# OSH inspector ratio to strengthen decent workplace safety and health: a cross-regional trend analysis research

**DOI:** 10.3389/fpubh.2026.1806383

**Published:** 2026-04-08

**Authors:** Arjun Kathayat, Mohd Zahirasri Mohd Tohir, Rabaaya Binti Daud, Mohd Rafee Baharudin

**Affiliations:** 1 Integrity Maintenance Ltd., Carlyle, SK, Canada; 2 Department of Community Health, Faculty of Medicine and Health Sciences, Universiti Putra Malaysia, Serdang, Malaysia; 3 International Research Institute of Occupational Safety and Health (IRIOSH), Manor, SK, Canada; 4 Safety Engineering Interest Group (SEIG), Department of Chemical and Environmental Engineering, Faculty of Engineering, Universiti Putra Malaysia, Serdang, Malaysia; 5 Policy, International and Research, Development Division, Department of Occupational Safety and Health (DOSH), Putrajaya, Malaysia

**Keywords:** decent workplace safety and health, fatal injuries, nonfatal injuries, occupational safety and health, OSH inspectors, SDG indicators 8.8.1, SDGs

## Abstract

**Introduction:**

Occupational safety and health (OSH) inspectors play significant roles in enforcing OSH policies and monitoring compliance with these policies to sustain decent workplace safety and health performance. However, there is contradictory or limited knowledge in the literature regarding the effectiveness as well as the standardization of the inspector ratios despite the International Labor Organization’s (ILO) initiatives to harmonize OSH policies and frameworks globally since its inception. Therefore, this cross-regional trend analysis research investigated the current inspector ratios to improve decent workplace safety and health performance in 85 ILO regions, including Malaysia and Saskatchewan.

**Methods:**

This retrospective ecological research utilized annual secondary datasets to analyze the effectiveness and feasibility of baseline inspector ratios in ILO regions. This study performed observational trends in research variables with Microsoft Excel graphs. It also computed a total of 20 plus multivariate regression analyses with 95% confidence intervals (CIs) and descriptive statistics using IBM SPSS 30.0 software.

**Results:**

The visual inspector ratio trends, along with standard error bars, provided additional tools not only for evaluating the effectiveness of the inspector ratios but also for allowing readers to compare these trends to the expectations set out by the ILO’s press release in 2006. This study also validated the literature associated with the effectiveness of inspector ratios. This research has real-world implications for OSH risk management policy, suggesting that the baseline inspector ratios assigned to each regional group may serve as preliminary OSH inspector capacity ratios for the continuous improvement of decent OSH performance.

**Conclusion:**

Five baseline ratios for the inspector ratios that warranted careful consideration and further investigation to improve OSH risk management policies and performance were group 1: 0.87 to 1.5, group 2: 0.44 to 1.5, groups 3 and 4: 0.49 to 1.5, and Malaysia: 0.75 to 1.50. Saskatchewan’s inspector ratio has been stable (1.0 to 1.5) for the past few years, so this region might also need to investigate other confounders, such as institutional or organizational behavior and OSH culture, to enhance OSH performance. Future researchers might explore or examine the gaps in this study.

## 1 Introduction

OSH inspectors, also called labor inspectors or simply *inspectors* in this study, are employees of regional OSH regulatory bodies and play crucial roles in enforcing OSH policies in the regions. Regional *regulatory capacity* associated with such inspectors impacts both proactive and reactive OSH activities. While strict or punitive OSH policies for non-compliance may *deter* or discourage (1, 2) workplaces and their employees from unsafe and unhealthy behaviors, the inspectors’ activities, such as job-site inspections (3) and regulatory compliance tools (4), may be instrumental in promoting OSH policy compliance in workplaces, thereby strengthening decent workplace safety and health performance.

OSH risk management literature is in consensus that multiple factors, such as OSH surveillance and evaluation of the OSH framework (5), routine OSH inspections (6), OSH policies (7, 8), social dimensions of OSH (e.g., OSH culture) (9), professional safety officers, and OSH standards (10), affect multi-level (local, regional, and global) OSH performance. Likewise, it is plausible to contend that inspector capacity, inspectors’ activities, OSH policies on deterrence, institutional OSH culture or behaviors, and OSH frameworks are inter-reliant for higher OSH performance in the regions. Therefore, the study created the theoretical framework, including three major interconnected theories aligned with ILO’s OSH guidelines: (1) Regulatory capacity theory, (2) Deterrence theory (i.e., responsive regulation theory), (3) Institutional theory, and (4) ILO’s regional guidelines (C-155/ILO-OSH 2001) (11).

**Figure 1:**
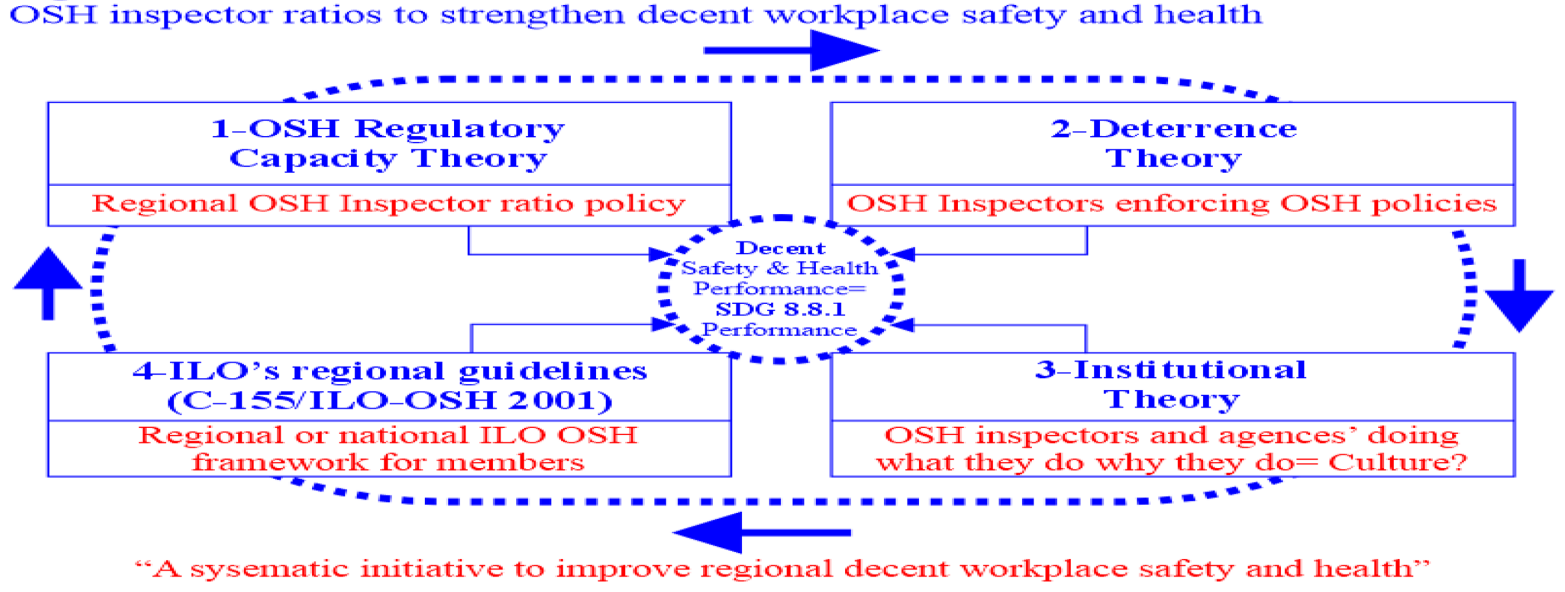
Theoretical framework–OSH inspector ratios to strengthen decent workplace safety and health. This study created in this figure, which shows three major theories and the OSH framework to systematically improve regional decent workplace safety and health. Above, C-155 stands for the International Labour Organization’s (ILO) Convention 155, which primarily focuses on the minimum standards for cross-regional Occupational Safety and Health (OSH) standards and sets the foundation for today’s ILO-OSH 2001 framework.

See Figure 1 Theoretical framework for details.

Figure 1 illustrates the relationship between each theory or OSH framework to generate and grow decent workplace OSH performance.

In Figure 1, the application of (regulatory) capacity theory (12) is not new in OSH policy contexts. There are multiple interdependent components, such as legal boundaries or authorities entrusted to the inspectors by the regional governments, the number of inspectors, and financial as well as other resources available to the OSH regulators, which may influence the OSH regulators’ ability to enforce the OSH laws. This study primarily investigates the inspector ratios, which represent a component of the regional capacity of the OSH regulatory agency to enforce OSH policies. This regulatory capacity theory can clarify the understanding of regional regulatory capacity (of ILO member nations or regions, including Saskatchewan). An adequate or higher number of inspectors may mean the region is more prepared or resourceful to enforce OSH policies by mobilizing the inspectors in proactive or reactive OSH activities like job-site inspections, issuing Summary of Offense (SOP) tickets (a practice in Saskatchewan), or fines and investigation of dangerous occurrences. The understaffing of inspectors may indicate that the region is not prepared to respond to OSH emergencies effectively. In other words, OSH laws set the minimum expectations and standards for workplaces and industries, but those laws unilaterally do not or cannot create a decent workplace environment. It is the regional regulators’ capacity to enforce and make the OSH stakeholders comply with the laws that has a more tangible impact on the organizations’ OSH behaviors (13, 14) in the creation or enhancement of OSH performance. In short, OSH regulations may grant legal authorities for workplace inspections and enforcement of the OSH laws, which compel workplaces and industries to readjust their OSH behaviors to comply with the laws, ensuring their due diligence, the wellbeing of the workers, and decent workplace environments.

Deterrence theory, in the context of workplace safety and health regulations, has two major components: enforcement of OSH laws and repercussions of violations of such laws, though this theory was initially explained in the criminal behavioral context (15). Deterrence theory emphasizes that OSH policies and inspectors’ job-site activities can impact multiple workplace stakeholders’ attitudes towards OSH policy compliance for decent workplace safety and health. For example, consequences (monetary penalties, jail time, or both) (16), or legal as well as non-legal punishments (e.g., public image or reputation) (15, p. 528) of serious violations of OSH policies or non-compliance may motivate the stakeholders—employers, supervisors, and employees—to create and participate in safer and healthier workplace environments for all.

Institutional theory, which originally explained how organizations or institutions would evolve similarly for legitimacy through “three isomorphic processes—coercive, mimetic, and normative” (17, p. 147), in the context of this study, *coercive* may represent regional OSH regulations or policies, *mimetic* indicates ILO’s regional regulatory bodies learning from each other’s institutional experiences to grow alike, and the ILO’s global or regional OSH guidelines or frameworks can be examples of the *normative* process to develop institutions alike. Additionally, institutional theory posits that every organization, including regional OSH policy enforcement agencies, has unique values, cultures, and leadership styles, which may influence their institutional or organizational behaviors (18). According to the literature (19), the institutional environment, regional OSH agencies’ organizational behaviors, and the perceptions of OSH policymakers or decision-makers (20) regarding OSH may impact how they allocate resources, as well as how they create, execute, and enforce OSH policies.

The ILO-OSH Framework-2001 (11) offers a systematic framework to ILO member nations by emphasizing national OSH policies, planning, implementation, and evaluation of the system to create and maintain decent workplace safety and health for all stakeholders. C-155 stands for the ILO’s Convention 155, which was primarily focused on the minimum standards for cross-regional OSH standards, setting the foundation for today’s ILO-OSH 2001 framework. This ILO-OSH framework can be incorporated at two levels: the national or regional level and the organizational or workplace level (11, p. 1). National OSH policies endorse the ILO-OSH framework at the national level and offer guidelines to improve compliance with the OSH policies at both national and workplace levels (11).

Thus, three theories and one OSH framework or guidelines in this study’s theoretical framework, as shown in Figure 1, are complementary to each other in improving compliance and enforcement of OSH policies and systematically growing OSH performance. The regulatory capacity of an ILO region influences the deterrence effectiveness of the inspectors, which is also interconnected or interdependent with the region’s institutional OSH cultures or behaviors. As a result, as a multi-level (global, regional, and workplace) OSH guideline, the ILO’s C-155 or ILO-OSH 2001 framework offers voluntary recommendations on how ILO regions and their workplaces can collectively improve decent workplace safety and health and promote sustainability.

However, on one hand, OSH laws rely on the OSH regulators or inspectors to enforce them in the regions. On the other hand, the literature remains divided on whether standard risk management regulations (21, 22), or in this study’s context, the *standardization* of the inspector ratios, are a good or detrimental risk management strategy. Evidence from a meta-analysis (23) reports that OSH policies and inspections can significantly reduce workplace injuries and fatalities and improve compliance with OSH regulations (23, p. 103). Certainly, how effectively a regional OSH regulatory agency can create deterrence, discourage non-compliance with OSH policies, and promote favorable institutional or organizational OSH behaviors to some extent depends on the region’s capacity or resources at its disposal. Thus, it is vital to audit or assess their regional capacity periodically and know, “What are *poor, average*, and *good* inspector ratios?”

Due to the scope of this research, this study primarily focused on investigating the trends and statistical feasibility of inspector ratios for the ILO’s regional members, rather than engaging in an in-depth debate about whether to support, oppose, or remain neutral on the standardization of these ratios. The ILO’s recently reported statistics, with datasets consisting of SDG 8.8.1 indicators, OSH inspectors, the number of workplace visits, and the numbers of job sites inspected by OSH inspectors, were complex, incomplete, and might not be visually reader-friendly for stakeholders.

See Table 1 OSH inspectors and their activities in ILO regions for details.

**Table 1:** Inspectors and their activities in ILO regions.

Income group	Regions	A	B	C	D	E	F	A1	G	H	I	J	K	Other
Group 2	Algeria	–	17.59	1002.7	–	–	–	–	–	–	–	–	–	–
Group 1	Andorra	–	–	–	–	–	–	–	8	4	4	50	–	–
Group 2	Argentina	0.25	3.015	3201.89	359.12	132,157	757,023	0.29	368	224	144	39.1	42.3455	–
Group 2	Armenia	1.1	4.19	29.4	–	–	–	–	126				53.32734	–
Group 1	Aruba	2.3	–	–	–	–	–	–	–	8	3	27.3	–	–
Group 1	Australia	0.99	1.416	944.783	–	–	–	–	–	–	–	–	–	–
Group 1	Austria	0.73	1.803	2322.598	156	49,253	260,659	0.71	315	223	92	29.2	47.10592	–
Group 2	Azerbaijan	0.45	4	22	1.9	11,338	71,188	0.45	212	201	11	5.2	49.27123	–
Group 1	Bahrain	0.26	0.59	109.02	104.811	5,555	81,551	0.26	53	–	–	–	21.15465	–
Group 1	Barbados	1.4	0.75	388.26	79	1,263	2029	1.4	18	6	12	66.7	50.16909	–
Group 2	Belarus	0.41	2.684	50.964	99.4	19,590	191,000	0.41	197	93	104	52.8	50.64676	–
Group 1	Belgium	0.56	0.12	1425.74	104.41	30,384	–	0.58	291	133	158	54.3	47.19394	–
Group 2	Belize	1.38	5.187	909.523	34.76	869	11,346	1.44	25	11	14	56	37.59143	–
Group 3	Bhutan	0.52	–	–	350	5,252	18,327	0.52	15	13	2	13.3	42.47717	–
Group 2	Brazil	0.31	7.433	1,374	99.75	275,139	3,837,000	0.31	2,775	1761	1,014	36.5	42.71707	–
Group 2	Bulgaria	1.41	3.507	86.517	120	42,396	34,988	1.13	355	109	246	69.3	46.45369	–
Group 4	Burkina Faso	0.31	4.4	1894.74	7.77	1920	17,148	0.47	247	212	35	14.2	45.06198	–
Group 4	Burundi		13.84	707	62	2,108	1,668	–	34	27	7	20.6	50.8722	–
Group 1	Canada	0.11	5.198	1347.6	9.15	2,323	43,316	0.12	230	90	140	60.9	47.50663	–
Group 1	Chile	0.91	3.058	3141.8	111.2	82,514	–	0.84	742	379	355	47.8	42.5802	8
Group 2	Colombia	0.51	6.46	4782.889	16.5	8,494	12,359	0.41	904	439	465	51.4	40.47492	–
Group 2	Costa Rica	0.58	9.72	9421.38	38	4,439	4,439	0.61	118	55	63	53.4	38.25794	–
Group 1	Croatia	1.1	2.58	548.69	77	14,484	274,384	1.1	188	82	106	56.4	46.63833	–
Group 2	Cuba	0.76	25	1,017	–	–	–	–	–	–	–	–	37.66953	–
Group 1	Cyprus	0.29	3.24	304.54	330.4	6,608	45,284	0.46	20	2	18		46.23625	–
Group 1	Czechia	–	–	–	36.4	18,144	2,627,378	0.95	498	254	244	49	43.9082	–
Group 1	Denmark	–	1.03	1613.14	–	–	–	–	–	–	–	–	–	–
Group 2	Dominican Republic	0.39	17.93	336.35	400.53	63,283	76,852	0.39	158	85	73	46.2	39.47609	–
Group 2	Ecuador	0.17	1	9	0.57	9,282	863,681	0.2	157	82	75	47.8	40.69014	–
Group 3	Egypt	–	2.9	371	–	–	–	–	–	–	–	–	–	–
Group 3	El Salvador	0.47	0.14	28.68	284	37	30,820	0.47	131	83	48	36.6	40.98009	–
Group 1	Estonia	0.66	1.4	472	100	4,638	54,803	0.68	46	25	21	45.7	49.39846	–
Group 3	Eswatini	–	–	–	–	110	–	–	–	–	–	–	–	–
Group 1	Finland	1.26	1.028	1261.504	64.66	22,500	226,491	1.4	348	200	148	42.5	48.79557	–

Income group	Regions	A	B	C	D	E	F	A1	G	H	I	J	K	Other
Group 1	France	0.8	6	8670.36	67	139,068	1,810,000	0.8	2,175	827	1,348	62	49.18573	–
Group 1	French Guiana	–	0	1057.33	–	–	–	–	–	–	–	–	–	–
Group 2	Georgia	0.43	2.35	18.32	116	3,755	230	0.65	84	52	32	38.1	47.90191	–
Group 1	Germany	1.42	0.71	1396.8	97	586,205	4,635,600	1.41	6,009				46.79487	–
Group 1	Greece	–	0.62	115.42	–	–	–	–	–	–	–	–	–	–
Group 1	Guadeloupe	–	6.75	1349.01	–	–	–	–	–	–	–	–	–	–
Group 2	Guatemala	0.25	0.14	27.9	214	32,528	55,264	0.22	152	91	61	40.1	33.89053	–
Group 4	Guinea	–	–	–	–	–	–	–	–	–	–	–	41.29955	–
Group 1	Hong Kong, China	0.5	6.782	1188.09	777	143,037		0.5	186	104	82	44.1	50.14027	–
Group 1	Hungary	0.57	1.6	429.1	81	22,040	1,992,415	0.58	273	149	124	45.4	46.50022	–
Group 1	Iceland	0.95	1.38	1028.305	–	–	–	–	–	–	–	–	46.83817	–
Group 3	India	–	116.8	325	–	–	–	–	–	3	1	25	–	–
Group 1	Ireland	0.23	1.44	808.69	62.587	3,943	202,060	0.25	63	–	–	–	–	–
Group 1	Isle of Man	–	2	390	70	280	2,500		4	–	–	–	–	–
Group 1	Israel	0.52	1.624	1073.66	215	23,528	82,135	0.52	203	80	123	60.6	47.63519	–
Group 1	Italy	–	2.01	1137.39	–	–	–	–	–	–	–	–	–	–
Group 2	Jamaica	–	–	–	–	4,725	–	–	–	–	–	–	–	–
Group 1	Japan	0.46	1.32	234.6	55.4	171,575		0.46	3,094	–	–	–	45.20044	–
Group 3	Jordan	0.69	13.15	2312.55	–	–	–	–	–	–	–	–	–	–
Group 2	Kazakhstan	–	4.3	41.5	–	–	–	–	–	–	–	–	–	–
Group 1	Korea, Republic of	0.75	3.86	–	12.8	27,180	2,146,156	0.75	2,126	1,154	972	45.7	43.46663	–
Group 3	Kyrgyzstan	–	4.1	22.1	–	–	–	–	–	–	–	–	–	–
Group 3	Lao People’s Democratic Republic	–	–	–	–	–	–	–	–	–	–	–	47.12159	–
Group 1	Latvia	1.15	3.53	288.72	92	9,897	887,716	1.3	116	32	84	72.4	50.17651	–
Group 1	Lithuania	1.46	2.4	473.4	47	6,779	374,100	1.01	143	82	61	42.7	49.53348	–
Group 1	Luxembourg	3.15	0.798	2551.402	118	10,171	46,882	2.76	86	52	34	39.5	46.16367	–
Group 1	Macao, China	2.65	6.93	1891.2	37.32	3,919	84,915	2.65	105	47	58	55.2	50.37247	–
Group 2	Malaysia	0.54	14.58	3229.69	57.4	47,634	1,116,093	0.54	830	376	454	54.7	38.86411	–
Group 1	Malta	0.59	2	684.698	62	377	–	0.21	6	2	4	66.7	41.74467	–
Group 2	Mauritius	2.47	3.7	45	35	4,740	6,895	2.58	134	49	85	63.4	38.60497	–
Group 2	Mexico	0.14	7.69	2,529	169.988	122,901	435,499	0.13	723	490	233	32.2	39.42576	–
Group 2	Moldova, Republic of	0.45	4.3	87.8	49	3,212	764,677	0.42	65	54	11	16.9	52.86693	–
Group 1	Monaco	–	–	–	–	–	–	–	3	2	1	33.3	–	–
Group 3	Mongolia	0.85	6.4	53.3	31.395	2,543	66	0.66	81	30	51	63	45.45507	–
Group 3	Morocco	–	–	–	–	–	–	–	–	–	–	–	22.1339	–
Group 3	Myanmar	0.07	3.146	11.83	213	33,114	25,974	0.07	155	45	110	71	37.73224	–
Group 2	Namibia	1.08	5	304.56	43	3,177	–	1.08	74	51	23	31.1	50.16101	–

Income group	Regions	A	B	C	D	E	F	A1	G	H	I	J	K	Other
Group 1	Netherlands	–	0.38	1038.11	–	–	–	–	–	–	–	–	–	–
Group 1	New Caledonia	–	7	5.59	–	–	–	–	–	–	–	–	–	–
Group 1	New Zealand	0.34	2.3	1,200	2.9	245	–	0.3	84	27	57	67.9	47.5164	–
Group 3	Nicaragua	–	7.95	4890.53	206	15,654	18,200		95	36	59	62.1	38.65511	–
Group 4	Niger	–	–	–	–	–	–	–	–	–	–	–	41.89458	–
Group 2	North Macedonia	1.72	–	–	89	11,532	9,746	1.7	129	50	79	61.2	41.22553	–
Group 1	Norway	1.1	1.1	392.28	30	10,586	250,000	1.21	346	159	187	54	47.12368	–
Group 3	Occupied Palestinian Territory	0.92	1	74	–	–	–	–	–	53	52	49.5	–	–
Group 1	Oman	–	–	–	–	–	–	–	282	258	24	8.5	16.12126	–
Group 3	Pakistan	–	44.25	2,691	–	–	–	–	–	–	–	–	–	–
Group 1	Panama	0.54	0.326	3.119	106.85	10,899	19,650	0.52	102	81	21	20.6	39.4096	–
Group 2	Paraguay	0.1			10.06	312	71,167	0.1	31	15	16	51.6	41.52701	–
Group 2	Peru	0.38			91.22	67	273,873	0.25	411	258	153	37.2	45.94968	–
Group 3	Philippines	0.13	3.2	227	130.3	81,314	95,305	0.13	624	379	245	39.3	38.7699	–
Group 1	Poland	0.87	1.19	483.6	39	59,570	1,064,164	0.92	1,539	907	632	41.1	45.59273	–
Group 1	Portugal	0.94	2.69	2493.8	95.4	39,786	318,254	0.87	417	139	278	66.7	49.64816	–
Group 1	Puerto Rico	–	8.18	–	–	–	–	–	–	–	–	–	–	–
Group 1	Qatar	1.28	2.958	39.908	190	43,366	80,850	1.28	270	203	67	24.8	17.07072	–
Group 1	Réunion	–	2.26	1988.58	–	–	–	–	–	–	–	–	–	–
Group 1	Romania	1.92	2.42	97.47	95	120,690	1,190,504	1.8	1,394	556	838	60.1	42.99008	–
Group 2	Russian Federation	–	5.032	96	–	–	–	–	–	–	–	–		–
Group 2	Saint Vincent and the Grenadines	–	2.62	851.092	–	–	–	–	–	–	–	–	42.453	–
Group 1	San Marino	3.89	0	2945.71	207.5	1,245	5,049	3.89	6	3	3	50	–	–
Group 1	Saudi Arabia	–	–	–	934	843,419	910,827	–	903	807	96	10.6	20.23361	–
Group 3	Senegal	0.17	–	–	57	5,112	–	0.2	89	–	–	–	38.65902	–
Group 1	Seychelles	1.18	4.79	165.24	38.4	538	5,000	3.09	14	3	11	78.6	–	–
Group 1	Singapore	0.32	0.99	622	175	23,223	43,320	1.11	261	185	76	29.1	42.30975	–
Group 1	Slovakia	1.12	1.12	359.98	185	54,282	710,210	1.13	294	156	138	46.9	46.97778	–
Group 1	Slovenia	0.94	2.2	1487.86	178.6	15,538	6,688	0.88	87	36	51	58.6	45.91895	–
Group 2	South Africa	1.08			161.984	312,792	298,104	1.08	1931	1,121	810	41.9	45.59473	–
Group 1	Spain	1.07	1.712	2319.639	105.9	223,982	1,332,390	1.07	2,115	787	1,328	62.8	45.95469	–
Group 3	Sri Lanka	0.58	0.48	21.2	205	79,076	251,169	0.58	470	261	209	44.5	31.89253	–
Group 1	Sweden	0.5	0.7	748	88	23,981	412,766	0.52	274	–	–	–	47.14893	–
Group 1	Switzerland	1.29	1.13	2344.97	61.36	38,532	338,219	1.33	628	–	–	–	46.61258	–
Group 1	Taiwan, China		4.5	432.8	–	–	–	–	––	––	–	–	–	–
Group 2	Thailand	0.18	4.9	718.08	54.64	783	443,266	0.18	674	–	–	–	45.86921	–

Income group	Regions	A	B	C	D	E	F	A1	G	H	I	J	K	Other
Group 4	Togo	0.39	16.3	484	7	922	2,311		132	102	30	22.7	48.7847	–
Group 1	Trinidad and Tobago	0.19	1.9	64.3	71	857	26,046	0.19	12	4	8	66.7	42.56078	–
Group 3	Tunisia	–	13.07	3639.11	–	–	–	–	–	–	–	–	–	–
Group 2	Türkiye	0.28	11.47	4409.18	35.58	26,434	2,139,496	0.3	933	711	222	23.8	32.23022	–
Group 4	Uganda	–	–	–	–	487	619	–	–	–	–	–	–	–
Group 3	Ukraine	0.75	7.6	166	20.3	4,362	1,956,248	0.75	885	331	554	62.6	–	–
Group 1	United Kingdom	–	–	–	55	13,212	3,219,980	0.29	934	–	–	–	–	–
Group 1	United Kingdom of Great Britain and Northern Ireland	0.41	0.78	691.65	–	–	–	–	–	582	352	37.7	47.70283	–
Group 1	United States	0.07	3.5	2,400	28	27,914	7,300,000	0.07	1,067	–	–	–	46.54482	–
Group 1	Uruguay	0.63	3.73	2,788	162	16,711	100,412	0.63	103	44	59	57.3	45.08348	–
Group 3	Uzbekistan	0.22	3.6	10	86	23,930	337	0.22	278	265	13	4.7	35.28608	–
Group 2	Venezuela (Bolivarian Republic of)	0.14	–	–	112.35	18,874	353,669	0.14	168	45	123	73.2	36.60446	–
Group 3	Zimbabwe	0.56	6	322	7.6	3,243	–	0.95	275	120	155	56.4	49.80612	–

This study created this table by retrieving/adapting the ILO’s table called “Occupational safety and health indicators” from see text footnote 1, the ILO’s table titled “Inspection visits to registered workplaces liable to labour inspection” from see text footnote 3, and the ILO’s table and chart called “Women’s share of labour inspectors and employment” from see text footnote 3.

In this table, groups are classified by ILO/World Bank: Group 1, High-income regions; group 2, upper-middle-income regions; group 3, lower-middle-income regions; group 4, lower-income regions.

Variables: A, Inspectors_per_10000_employed persons; B, SDG indicator 8.8.1–Fatal occupational injuries per 100′000 workers; C, SDG indicator 8.8.1–Non-fatal occupational injuries per 100,000 workers; A1, Inspectors_per_10000_employed persons (this “A1” column/statistics was included in this table to inform the readers and OSH stakeholders about potential discrepancies in self-reported secondary data from the regions).

Controlled variables: D, Visits_per_inspector; E, Visits_in_year; F, Workplaces_liable_to_inspection; G, Total Inspectors, Numbers of inspectors by sex; H, Inspectors (Male); I, Inspectors (Female); J, Share of Female Inspectors; K, Share of female employment; Others, Other genders.

(−), missing values in the ILO’s table called “Occupational safety and health indicators” retrieved from see text footnote 1, the ILO’s table titled “Inspection visits to registered workplaces liable to labour inspection” retrieved from see text footnote 3, and the ILO’s table and chart called “Women’s share of labour inspectors and employment” from see text footnote 3.

Studies by Gammarano (24) and Gómez-García et al. (25) alluded to the fact that there were no universal or standardized OSH ratios, even though, as also cited in Gómez-García et al. (25), the inspector ratio of one inspector per 10,000 workers might be a “helpful benchmark” (25, 26). Another ILO study pointed out that the number of inspectors required would depend on “the national context” (27, p. 20), such as the size of workplaces and the number of workers. International Labour Organization’s (26) consideration of benchmarking or binary classification of *“high” or “low”* inspector ratios was influenced by the ILO’s more than a decade-old literature titled “The informal economy and decent work: A policy resource guide supporting transition to formality,” published in 2013, and the data used for the quantitative analysis were from 2015 to 2019.

On one hand, no ILO conventions or guidelines, so far, have directly mandated a standard inspector ratio, but in 2006, ILO alluded that it might be “concerned” if *industrial economies*, *transition economies*, and *less developed regions exceeded* one OSH inspector per 10,000 workers, one OSH inspector per 20,000 workers, and one OSH inspector per 40,000 workers, respectively (28). This study was also an opportunity to evaluate if the ILO should or should not be *concerned* based on the currently trending inspector ratios across the ILO regions.

Additionally, a study from Europe by Williams et al. (29) investigated key performance indicators (KPI), strategic goals, and objectives to measure the effectiveness of inspectors. Nevertheless, Williams et al. (29) fell short of exploring or examining the inspector ratios that might be crucial to attaining such strategic goals or objectives. After all, inspectors also represent teams within the OSH policy enforcement agencies, and the inspectors do not perform their activities in isolation; generally, they work with other inspectors as teams. In other words, the inspector ratios have a significant impact on achieving the regulatory agencies’ OSH objectives. International Labour Organization (30) alludes to the fact that inspectors have a systematic influence on multiple types of OSH indicators, including inspection action, effectiveness, efficiency, and impact, as well as “*resource indicators, indicators of the work carried out, and efficiency and quality indicators,*” as also noted by De Wispelaere et al. (31), p. 19. A multinational report from Europe stated that the *effectiveness* of labor inspectors might be attained by *increasing the number of labor inspectors* (32, p. 22).

Moreover, literature (33) concurred that ineffective enforcement of OSH policies might promote a sense of *injustice* (p. 740), workplace tragedies (34), and effective OSH inspectors or agencies might succeed in prosecuting noncompliance with OSH policies (35) and also promote a sense of justice in the communities. Therefore, OSH policymakers and regulatory agencies may require evaluating if they are adequately *resourced* “in order for the inspection and enforcement system to be adequately designed and achieve the best balance of efficiency and effectiveness” (36, p. 14).

“Despite the consensus on the benefits of OSH inspectors” (25, p. 1), multiple studies associated with the inspector ratios or OSH inspectors, their roles, activities, tools available to OSH inspectors, or OSH inspectors’ effectiveness in creating decent workplaces, such as (8, 23–25, 37, 38), and ILO’s initiative to *harmonize* or develop universal labor inspection statistics (39), there is still limited knowledge on what a universal or standard regional inspector ratio is and its feasibility. Simply put, “more comprehensive studies are required to advance academic and institutional research” (7, p. 1) to develop more effective OSH *interventions,* and the inspector ratios are certainly one of those interventions that require further studies for decent workplace safety and health.

This study’s three major research questions are as follows:

*Research question 1 (RQ1)*: Visualize the inspector ratio trends across the ILO member regions, including Malaysia and Saskatchewan, and assess whether the regions lag, meet, or exceed the ILO’s expectations on the inspector ratios.

*Research question 2 (RQ2)*: Do regional inspector ratios (IV) have significant statistical relationships with SDG 8.8.1 (DV) or workplace fatal injury rates (DV1) and non-fatal injury rates (DV2) to justify a universal or regional inspector ratio?

*Research question 3 (RQ3)*: What is the evidence-based inspector ratio for each of ILO’s regional groups: group 1: high-income regions, group 2: upper-middle-income regions, group 3: lower-middle-income regions, and group 4: low-income regions, Malaysia, and Saskatchewan?

### 1.1 Operational definition of terms

OSH Policies: In this study, OSH policies, as defined in Kathayat et al. (8), are regional or national OSH laws, such as prescriptive, performance-based, or outcome-based, and system-based OSH risk management practices.

OSH Regulatory Capacity: In this study, OSH regulatory capacity refers to the capacity of the regional OSH regulatory agencies, including human resources capacity and the OSH inspector staffing ratio or capacity.

SDG Indicator 8.8.1: In this study, SDG Indicator 8.8.1, or SDG 8.8.1, is one of the United Nations Sustainability Development Goals (SDGs), which is quantified with SDG 8.8.1 fatal injury rates (DV1) and SDG 8.8.1 non-fatal injury rates (DV2) in the regions by ILO statistics. In this study, DV1 also refers to workplace fatal injury rates per 100,000 workers, and DV2 represents workplace non-fatal injury rates per 100,000 workers.

OSH Inspector Ratios: As in Kathayat et al. (8), the inspector ratios, an OSH leading indicator, represent the number of OSH inspectors or technical officers, as referred to in Malaysia, or labor inspectors, or simply *inspectors* per 10,000 employed persons in this study. The inspectors are regional OSH officers who can play a pertinent role in ensuring compliance with the regional OSH laws and risk management practices (8, 24). In other words, the inspector ratio is also known as the OSH inspector staffing or capacity ratio or labor inspector ratios, or simply *inspector ratios* in this study.

Income Levels: This study considered regional groups classified by the ILO and the World Bank based on regions’ economic strengths—groups 1 to 4; however, in this OSH issue-oriented research, GDPs of the regions were considered as controlled variables.

Decent Workplace Safety and Health: In this study, decent workplace safety and health, also referred to as sustainable workplace safety and health or sustainable OSH performance, represents one of the UN’s SDGs that is associated with healthier, safer, and more respectful workplaces with sustainable economic growth for stakeholders at workplaces and communities. In this research, this goal is measured with SDG 8.8.1.

## 2 Methods and materials

First, to find an answer to RQ1, this study explored OSH inspector trend analysis and created charts or graphs visualizing the inspector ratio trendlines across the ILO member regions, including Saskatchewan. These current inspector ratio trends were then compared to the ILO’s expectations for the inspector ratios, as outlined in the ILO’s press release titled “ILO calls for strengthening labor inspection worldwide” in 2006 (28).

Second, RQ2 was associated with a quantitative inferential analysis called multivariate regression analysis, and the hypothesis and null hypothesis of this RQ2 were as follows for this study:

*Hypothesis:* Regional inspector ratios (IV) have significant statistical relationships with SDG 8.8.1 (DV) or workplace fatal injury rates (DV1) and non-fatal injury rates (DV2) to justify a universal or regional benchmarking inspector ratio.

*Null hypothesis:* Regional inspector ratios (IV) do not have significant relationships with SDG 8.8.1 (DV) or workplace fatal injury rates (DV1) and non-fatal injury rates (DV2) to justify a universal or regional benchmarking inspector ratio.

A study of regression analysis to understand the response capacity of public healthcare systems, forecast the medical service demand (40), and examine the resiliency of nurse staffing (41, 42), human capital adequacy (43) in the healthcare sector, and even in higher education institutions (44) is common practice in scientific research.

**Figure 2:**
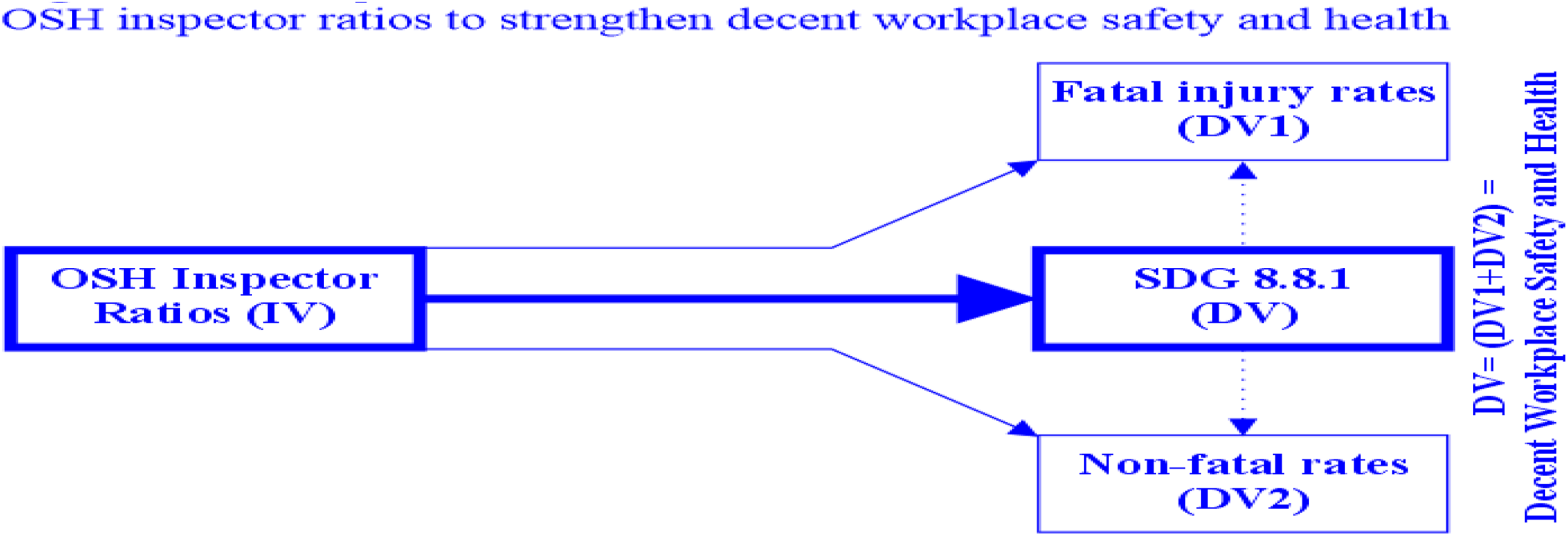
Conceptual framework-OSH inspector ratios to strengthen decent workplace safety and health. Conceptual framework-OSH inspector ratios to strengthen decent workplace safety and health. In this figure, independent variable (IV) = OSH inspector ratios, dependent variable (DV) = Sustainability Development Goal (SDG) 8.8.1 indicator, dependent variable (DV1) = fatal injury rates, and dependent variable (DV2) = non-fatal injury rates. DV = DV1 + DV2 = decent workplace safety and health = total injury rates.

See Figure 2 Conceptual framework of this study for details.

As illustrated in Figure 2 Conceptual framework, the only predictor variable was the regional inspector ratio (IV), and the major dependent variable was the regional SDG Indicator 8.8.1 (DV), which was further classified into fatal injury rates (DV1) and non-fatal injury rates (DV2) in this study. SDG 8.8.1 also represented annual total injury rates (i.e., DV1 + DV2).

Third, regarding RQ3, this study intended to examine the descriptive statistical evidence on the inspector ratio trends in each economic group and also for Malaysia and Saskatchewan separately to evaluate the feasibility of the baseline or initial inspector ratios using IBM SPSS 30.0 analytical software. An evidence-based study of descriptive statistics (45–49) in OSH research for the sake of benchmarking of OSH leading indicators is not a new approach.

### 2.1 Study setting and population

This research conducted a cross-regional study with secondary datasets in two ILO member nations: Saskatchewan (Canada) and Malaysia between 17 August 2025, and 4 February 2026. The participants in this cross-regional study are ILO’s member regions. Any ILO member region that had at least one complete set of statistics (i.e., IV, DV1, and DV2) was included in this study (see Tables 1–3). Any ILO region that had an incomplete set of statistics (missing values of either IV, DV1, or DV2) was removed from the multivariate regression analysis. This research conveniently invited Saskatchewan (a province in Canada, an ILO member nation) and Malaysia, another ILO member nation, to participate in this study. Thus, including Malaysia (see Table 2) and Saskatchewan (see Table 3), a total of 89 geographical regions’ annual inspector ratio trends and inspectors’ activities were investigated.

**Table 2:** Malaysia’s annual OSH performance (2014 to 2023).

					Variables used for regression analysis		
					OSH inspectors ratios (IV)	Dependent variables (SDG 8.8.1 Fatal and Non-Fatal Injuries)	
Study period	Total injury rates (/1,000 workers)	Total injury rates (/100,000 workers) (DV)	Total numbers of OSH Inspectors	Employed persons (millions)		Fatal injury rates (DV1)	Non-fatal injury rates (DV2)
Year					A	B	C
2014	3.10	310	–	13.85	–	4.21	305.79
2015	2.81	281	1,110	14.07	0.7894	4.84	276.16
2016	2.88	288	1,078	14.16	0.7615	4.84	283.16
2017	2.93	293	–	14.48	–	4.9	288.1
2018	2.40	240	1,114	14.78	0.7536	4.14	235.86
2019	2.71	271	1,111	15.07	0.7371	3.83	267.17
2020	2.22	222	1,093	14.72	0.7427	2.12	219.88
2021	1.45	145	1,145	14.83	0.7715	2.03	142.97
2022	2.26	226	1,122	15.16	0.7399	2.09	223.91
2023	2.46	246	1,130	15.81	0.7147	2.05	243.95

Retrieved/adapted from Department of Statistics Malaysia (50), Official website Department of Occupational Safety and Health–Archive publication (51), and Official website Department of Occupational Safety and Health–Annual report (52).

In this table, A, OSH inspector per 10,000 employed persons ratios, B, total fatal injury rates per 100,000, and C, total non-fatal injury rates per 100,000 workers.

The OSH inspectors or OSH officers or labor inspectors are also known as technical staff or enforcement staff or assistants under the engineering scheme in Malaysia. This does not include non-technical staffs as well as the “secondment officers” who are not the OSH enforcement officers but may be involved in specific workplace incidents or cases to support the tasks of OSH enforcement officers in Malaysia.

As shown in this table, this study converted the total injury rate, also known as the rate of occupational injuries per 1,000 workers reported by Official website Department of Occupational Safety and Health–Archive publication (51), Official website Department of Occupational Safety and Health–Annual report (52), to the total injury rates per 100,000 workers.

As shown in in this table, this study computed the total non-fatal injury rates (DV2) by subtracting the total fatal injury rates per 100,000 workers (DV1) from the total injury rates per 100,000 workers (DV) for each year in Malaysia.

As shown in in this table, this study converted the number of OSH inspectors per employed persons (in millions) to OSH inspectors per 10,000 employed persons (IV).

**Table 3:** Saskatchewan’s annual OSH performance (2017 to 2024).

Study period	Variables used for regression analysis		
	OSH inspectors ratios (IV)	Dependent variables (SDG 8.8.1 Fatal and Non-Fatal Injuries)	
		Fatal injury rates (DV1)	Non-fatal injury rates (DV2)
Year	A	B	C
2017	1.0488	7.28	5242.72
2018	1.1297	12.95	5427.05
2019	1.1267	9.71	4940.29
2020	1.2602	9.17	4450.83
2021	1.2284	8.36	4551.64
2022	1.1865	10.52	4319.48
2023	1.1482	7.82	3942.18
2024	1.1291	7.28	3902.72

Retrieved/adapted from Saskatchewan Workers’ Compensation Board (53). This study sought and obtained statistics associated with OSH inspector ratios (IV) from the Occupational Safety and Health (OSH) Division, Ministry of Labor Relations and Workplace Safety, Government of Saskatchewan, Canada.

In this table, A, OSH inspector per 10,000 employed persons ratios, B, Total Fatal Injury Rates per 100,000, and C, Total Non-Fatal Injury Rates per 100,000 workers.

### 2.2 Data source and measurements

There are three main data sources: (1) ILO’s publicly available statistics on OSH indicators, (2) Malaysia’s publicly available OSH indicator statistics, inspector numbers, and employed persons to calculate the inspector ratio, and (3) Saskatchewan’s workplace injuries statistics publicly shared by WCB-Saskatchewan, along with OSH inspector staffing ratios (annual) statistics sought and obtained from the OSH Division in Saskatchewan.

This study extracted statistics on the inspector ratios (IV), fatal injury rates (DV1), and non-fatal injury rates (DV2) from ILO’s OSH indicator table titled “Occupational safety and health indicators,” available at,^1^ by clicking on the “Get the Data” link right under the ILO’s table. There were OSH indicators related statistics associated with 108 regions; however, not all regions had complete datasets for the research variables—IV, DV1, and DV2.

Then, this study retrieved OSH statistics from the ILO’s table titled “Inspection visits to registered workplaces liable to labour inspections” from^2^ by clicking on the “Get the Data” link right under this table. There were statistics related to 88 regions. These 88 regions were divided into four categories according to the World Bank’s classification: high-income, upper-middle-income, lower-middle-income, and lower-income regions. Additionally, this ILO table included statistics related to visits per inspector, visits in the year, workplaces liable to labor inspection, inspector ratios, and the number of labor inspectors by sex (i.e., total inspectors). This study observed some discrepancies (see Table 1’s columns A and A1) in the inspector ratios in both ILO tables: “Occupational safety and health indicators” and “Inspection visits to registered workplaces liable to labour inspections.” Readers should be careful while interpreting the meaning of these datasets or while assessing the impact of the inspector ratios in theoretical or practical terms.

This study also retrieved ILO’s publicly available chart called “Women’s share of labour inspectors and employment” from^3^ by clicking on the “Get the Data” link right under the chart titled “Women’s share of labour inspectors and employment.” This ILO table included 94 names of countries or regions, year, total, other (other genders), male, female, share of female inspectors, and share of female employment in the regions. It was observed that not all statistics were current and some statistics were more than 10 years old. Multiple regions had missing statistics in this table as well.

Thus, this study extracted OSH-related statistics from ILO’s two tables and one chart, then merged the statistics and created a single table (see Table 1 OSH Inspectors and their activities in ILO regions), ensuring the inclusion of research variables. This research used the statistics retrieved from ILO to measure ILO regions’ IV, DV1, and DV2 as illustrated in Figure 2 of this study.

This study sought and obtained consent to retrieve and reuse (reprint) the ILO’s OSH indicators related statistics, especially Table 1, from the ILO Head Office, Switzerland, for this research on 30 January 2026.

In the case of Malaysia, this study retrieved annual employed persons statistics from Department of Statistics Malaysia (50) for the period of 2014 to 2023. Additionally, this study extracted the total number of inspectors from Official website Department of Occupational Safety and Health–Archive publication (51) and Official website Department of Occupational Safety and Health–Annual report (52) to compute the inspector ratios for Malaysia. This study also retrieved annual DV1 and DV2 stats from Official website Department of Occupational Safety and Health–Archive publication (51) and Official website Department of Occupational Safety and Health–Annual report (52). Since Malaysia used two different measurements—fatal injuries per 100,000 employed persons (DV1) and non-fatal injuries per 1,000 employed persons (DV2)—this study converted the annual non-fatal injuries per 1,000 employed persons to non-fatal injuries per 100,000 employed persons for standardization in this investigation. Note that those Malaysian annual reports were in the Malaysian language, and native Malaysian language speakers who were members of this study reviewed and extracted those stats. Thus, this study created Table 2 Malaysia’s annual OSH performance (2014 to 2023). Then, due to ambiguity in publicly available statistics, this study sought and obtained verification of the statistics used in Table 2 from the Department of Occupational Safety and Health (DOSH), Malaysia, for the descriptive and regression analysis in this study.

This study excluded Malaysia’s 2014 and 2017 research datasets from the descriptive statistics and regression analysis due to ambiguity in the annual reports for those years.

See Table 2 Malaysia’s Annual OSH Performance (2014 to 2023) for details.

In the case of Saskatchewan, this study retrieved SDG 8.8.1 datasets from Saskatchewan Workers’ Compensation Board (53). There were archived datasets for workplace fatalities and non-fatality rates from 2013 to 2024. Each year had a tab designated for workplace injuries under two separate files: “statistical supplement” and “industry summary statistics.” This study sought and obtained longitudinal statistics for the inspector ratios for Saskatchewan from the OSH Division, Ministry of Labour Relations and Workplace Safety, Saskatchewan, and populated the statistics related to Saskatchewan’s research variables in this study.

See Table 3 Saskatchewan’s Annual OSH Performance (2017 to 2024) for details.

### 2.3 Bias

The research data used in the observational analysis of the inspector ratios, multivariate regressions, and descriptive statistics were ecological secondary self-reported statistics. This study observed discrepancies or inconsistencies in those secondary datasets, as pointed out in the earlier section 2’s data sources and measurements. Therefore, to minimize bias in the skewed research datasets, this study assessed the credibility of statistics, especially those associated with research variables analyzing the sensitivities (such as potential discrepancies in data reporting as pointed out in Table 1), robustness (due to small sample sizes), functional form (log-transformation due to skewed data), and external validities (whether the findings of this study validate existing literature and similarities or differences between groups/ILO regions). Furthermore, to reduce biases in authorship, the authors reviewed and approved each other’s works in this research.

### 2.4 Data analysis

There were three major sections in the data analysis process.

**Figure 3:**
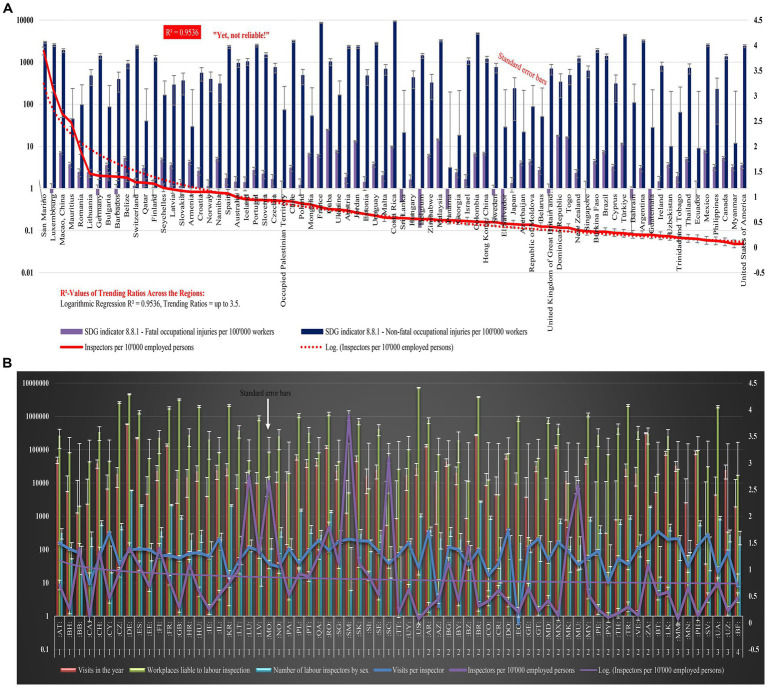
Cross-regional trends of OSH inspector ratios across 77 regions. This study created this graph by retrieving statistics from the ILO’s table named “Occupational safety and health indicators” available on the webpage: see text footnote 1. In **(A)**, the solid line represented the inspector ratio across the ILO regions, and the dotted line was the log-inspector ratio trendline. This study also added standard error bars (in both directions—minus and plus) on the inspector ratio trendline created with Microsoft Excel. Among 108 ILO regions, only 77 regions had complete statistics for the research variables; hence, this study included only those 77 regions with complete statistics in **(A)**. **(B)** Cross-regional trends of OSH inspector ratios, activities, and impact on SDG 8.8.1 indicators across 69 ILO regions. This study created this graph by retrieving statistics from the ILO’s tabled named “Inspection visits to registered workplaces liable to labour inspection,” available on the webpage (see text footnote 2) and also added standard error bars (both directions—minus and plus) on the inspector ratio trendline. Groups: 1, High-Income Regions; 2, Upper Middle-Income Regions; 3, Lower-Middle Income Regions; 4, Low-Income Regions. Countries/Regions: Austria, AT; Bahrain, BH; Barbados, BB; Canada, CA; Switzerland, CH; Cyprus, CY; Czechia, CZ; Germany, DE; Spain, ES: Estonia, EE; Finland, FI; France, FR; United Kingdom, GB; Croatia, HR; Hungary, HU; Ireland, IE; Israel, IL; Korea, Republic of, KR; Lithuania, LT; Luxembourg, LU; Latvia, LV; Macao, China, MO; Norway, NO; Panama, PA; Poland, PL; Portugal, PT; Qatar, QA; Romania, RO; Singapore, SG; San Marino, SM; Slovakia, SK; Slovenia, SI; Sweden, SE; Seychelles, SC; Trinidad and Tobago, TT; Uruguay, UY; United States, US; Argentina, AR; Azerbaijan, AZ; Bulgaria, BG; Belarus, BY; Belize, BZ; Brazil, BR; Colombia, CO; Costa Rica, CR; Dominican Republic, DO; Ecuador, EC; Georgia, GE; Guatemala, GT; Moldova, Republic of, MD; Mexico, MX; North Macedonia, MK; Mauritius, MU; Malaysia, MY; Peru, PE; Paraguay, PY; Thailand, TH; Türkiye, TR; Venezuela, VE; South Africa, ZA; Bhutan, BT; Sri Lanka, LK; Myanmar, MM; Mongolia, MN; Philippines, PH; El Salvador, SV; Ukraine, UA; Uzbekistan, UZ; Burkina Faso, BF. The ILO’s statistics associated with **(B)** had groups 1, 2, 3, and 4 assigned by the ILO.

First, this study created Figure 3A Trends of OSH inspector ratios across ILO regions, visualizing the trends of inspectors by utilizing statistics from the ILO’s table called “Occupational safety and health indicators,” retrieved from see text footnote 1. This study also created a visual representation, i.e., Figure 3B OSH inspector’s activities and impact on SDG 8.8.1, a graph of ILO’s statistics associated with the ILO’s table titled “Inspection visits to registered workplaces liable to labour inspection,” retrieved from see text footnote 3.

**Figure 4:**
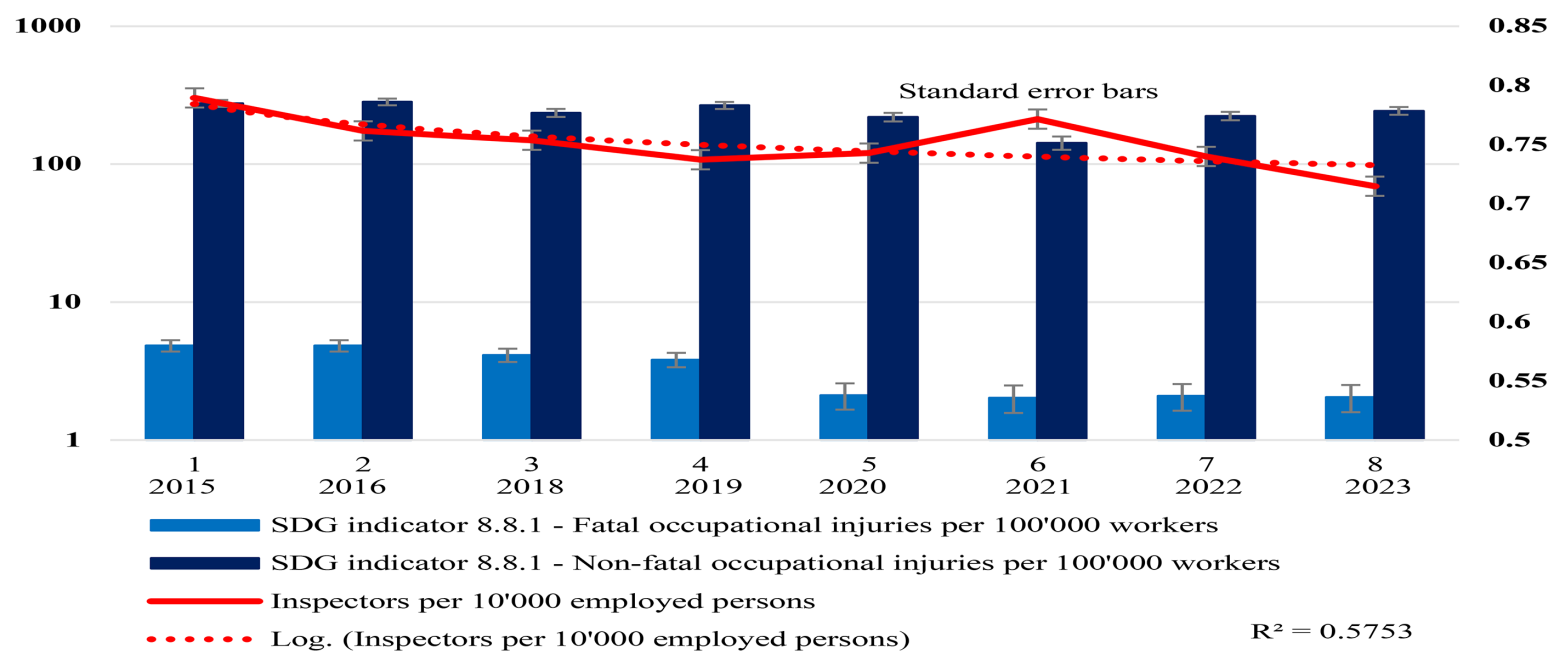
Malaysia’s OSH inspector ratios and impact. This study created in this figure to visualize Malaysia’s inspector ratio and its impact on decent workplace safety and health performance trends with standard error bars based on research variables included in Table 2. The solid line represents the inspector ratio in Malaysia, while the dotted line is the log-inspector ratio trendline (*R*^2^ = 0.5753). This study also added standard error bars (in both directions—minus and plus) on the inspector ratio trendline created with Microsoft Excel.

**Figure 5:**
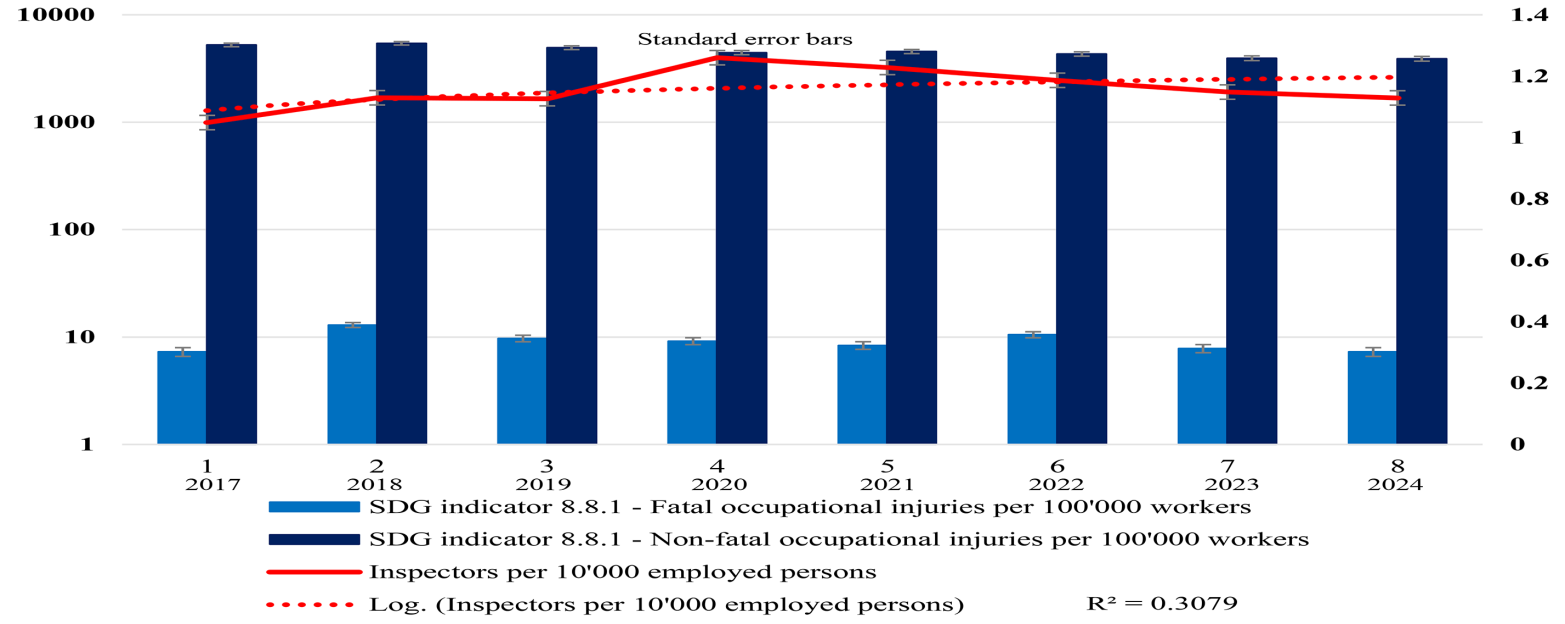
Saskatchewan’s OSH inspector ratios and impact. This study created in this figure to visualize Saskatchewan’s inspector ratio and its impact on decent workplace safety and health performance trends based on research variables included in Table 3. The solid line represents the inspector ratio in Saskatchewan, while the dotted line is the log-inspector ratio trendline (*R*^2^ = 0.3079). This study also added standard error bars (in both directions—minus and plus) on the inspector ratio trendline created with Microsoft Excel.

This study used the statistics in Tables 2, 3 to create visual graphs showing the inspector ratios’ annual trends in Malaysia and Saskatchewan, named Figures 4, 5, respectively.

This research exploited Microsoft Excel’s combo-clustered column-line, dual-axis with logarithmic scale functions, where applicable, to create Figures 3A,B, 4, 5.

Second, this study performed assumption tests associated with general linear multivariate (GLM) regression analysis with IBM SPSS 30.0, using statistics associated with research variables (IV, DV1, and DV2) as shown in Table 1, for all the ILO regions that had complete statistics for research variables. The application of log transformation of dependent variables (54) and the study of log-linear regression in retrospective research with publicly available secondary data (55) and log transformation of independent variables (56) in a general linear multivariate study were not new concepts in public safety and health research. On the other hand, the datasets, especially related to dependent variables, were ratios or rates, not count data, and were non-linear, as per normality tests computed with SPSS 30. Thus, this study evaluated the preliminary benchmarking of the inspector ratios for each group (groups 1, 2, 3, and 4) separately by comparing the observed inspector ratios with multivariate regression model-based expected inspector ratios via observed ratios-to-expected ratios and residual differences, which also was not a new concept in public safety and health research.

**Figure 6:**
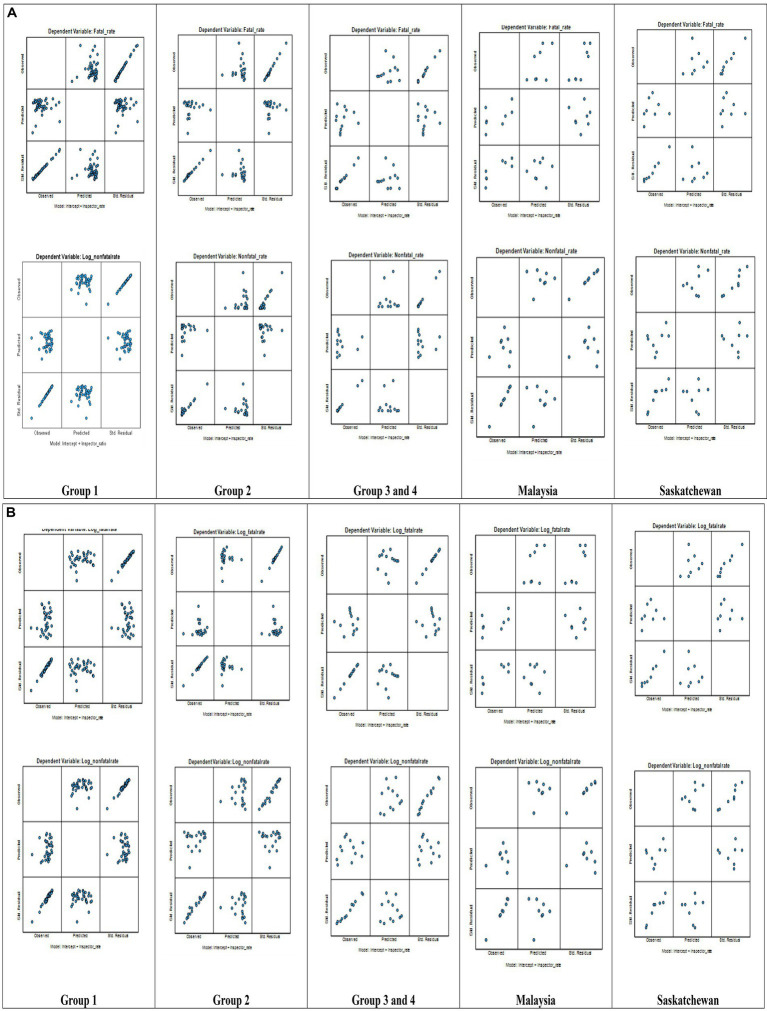
**(a)** Observed * Predicted * Std. Residual Plots. General Linear Multivariate Regression (Standard). **(a)** This study generated Observed*Predicted*Std. Residual Plots using IBM SPSS 30.0 for each group, including Malaysia and Saskatchewan as part of GLM analysis and external validity assessment across the groups. **(b)** Observed * Predicted * Std. Residual Plots. Log-General Linear Multivariate Regression. **(b)** This study generated Observed*Predicted*Std. Residual Plots using IBM SPSS 30.0 for each group, including Malaysia and Saskatchewan as part of Log-GLM analysis and external validity assessment across the groups.

See Figure 6a Observed * Predicted * Std. Residual Plots for details (for GLM regression), and see Figure 6b Observed * Predicted * Std. Residual Plots for details (for Log-GLM regression).

In the case of Malaysia and Saskatchewan, this study performed assumption tests associated with multivariate regression and multivariate regression tests using SPSS 30.0 software. This study used statistics in Table 2 to test the assumptions and regression design for Malaysia. Malaysia’s datasets include both domestic and foreign workers, as well as self-employed persons, as total employed persons. This research utilized statistics for the period of 2017 to 2024, as shown in Table 3 for Saskatchewan. In this study, Saskatchewan’s datasets exclude self-employed workers across the industry in the region.

Third, this study computed descriptive statistics, such as means, medians, and standard deviations for the inspector ratios across the ILO regions (groups 1, 2, 3, and 4), Malaysia, and Saskatchewan, to assess the evidence-based baseline inspector ratios. A study or inclusion of such descriptive statistics or *base* values, including a *benchmarking z-score* (57, p. 3) in evidence-based (OSH) policy decisions (58), was necessary to ensure the resource capacity of a regulatory agency was consistent with public safety and health research. This study selected median values of OSH inspector trends in the ILO’s regional groups as the preliminary benchmarking of inspectors for the respective groups since datasets were skewed, as also observed by Gammarano (24), in one direction (59), and the findings of regression analysis were not significant or adequate for benchmarking inspector ratios.

Further, this study recommended Saskatchewan’s inspector ratios as “good” compared to the inspector ratios for ILO’s regions, especially those that enjoy similar national or regional OSH culture, OSH policy regimes, and economic strengths, since Saskatchewan’s inspector ratio (1 to 1.26 inspectors per 10,000 employed persons) has been stable for about eight years and this region has also experienced declining workplace injury rates (see Table 3).

**Figure 7:**
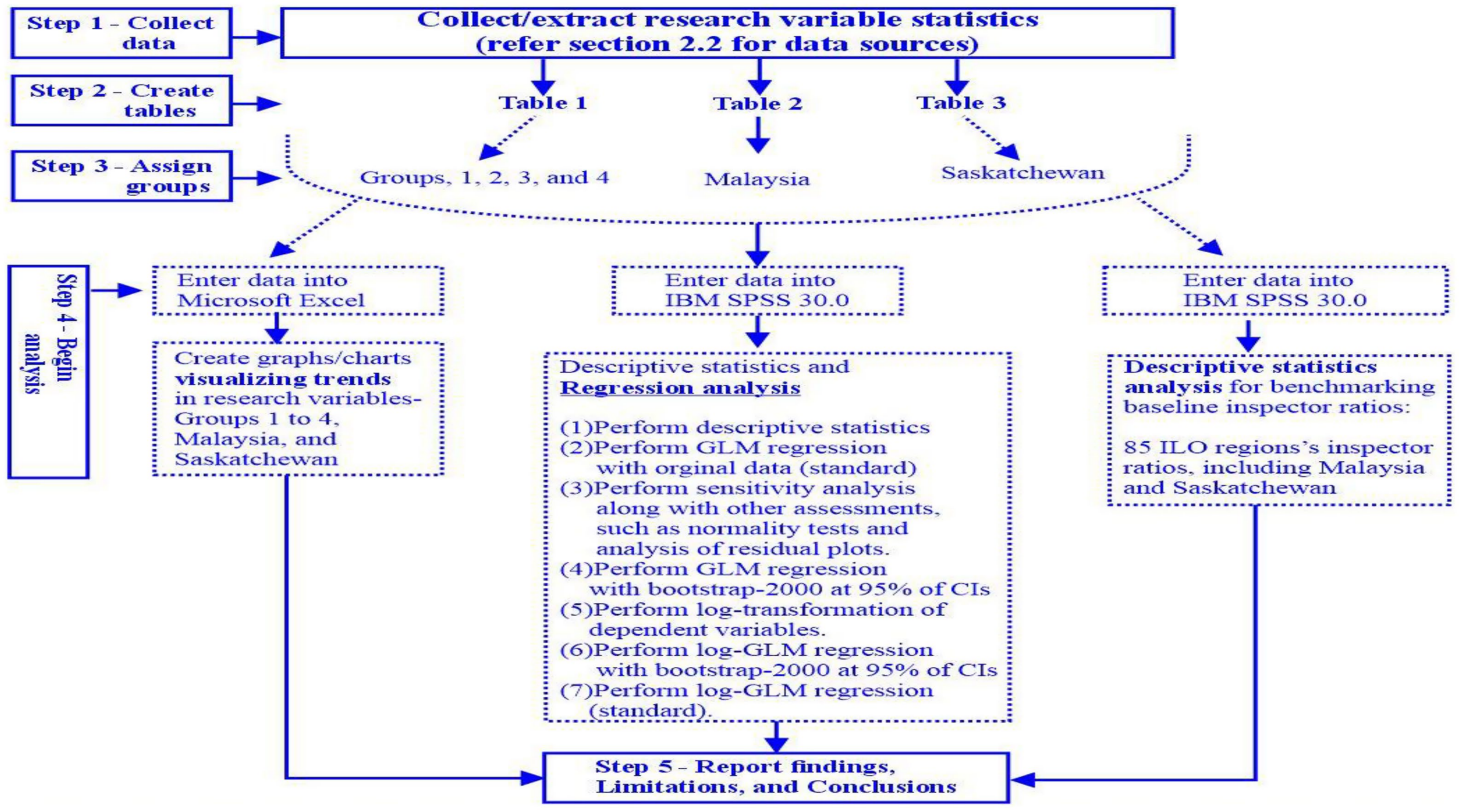
Overview of the major steps of the study plan and data analysis. This study created in this figure to provide a high-level overview of the major steps in the study plan and regression data analysis to readers and future researchers for better clarity and replicability. Something to consider in this exploratory ecological research–How credible were the ecological secondary data and associations between research variables?. *Assess sensitivities–Identify, investigate, and if required, remove influencial data before running the final analysis. *Assess Cook’s distance/leverage values while assessing sensitivity. *Normality of residuals. *Assess robustness–Perform Bootstrp-200 at interval of 95% CIs. *Functional form(s)–Log-regression due to skewed data. *External validity–Compare this study’s findings with similar studies, and also compares the findings between groups, Malaysia, and Saskatchewan for consistency, including directions. *Multicollinearity–No issues since inspector ratios is the only predictor.

See Figure 7 Overview of the major steps of the study plan and data analysis.

This study created Figure 7 as a framework for this exploratory ecological study to provide a high-level overview of the major steps taken from data collection to reporting the findings, limitations, and conclusions for better clarity and replicability.

The following section 3 will report the results of this research.

## 3 Results and discussion

This section summarizes the results of this study by discussing three major findings: first, the visuals/graphs/trends in section 3.1; second, the multivariate regression analysis in section 3.2; and finally, the descriptive statistics analysis in section 3.3, hereunder.

### 3.1 Visual/graphs/trends of inspector ratios

This study created graphs/charts illustrating the trends of the inspector ratios across ILO member regions, including Malaysia and Saskatchewan, and also visual trends of the inspector ratios and their activities across ILO regions: groups 1, 2, 3, and 4.

See Figures 3A,B for global trends for details.

Figures 3A,B visualize the global trends of the inspector ratios in the ILO member nations, their roles, and impact on OSH performances, such as SDG Indicator 8.8.1 fatal and non-fatal injury rates.

When the statistics were retrieved initially for Figure 3A with standard error bars, there were 108 regions in the ILO’s datasets. However, this study included only 77 regions in Figure 3A since only 77 regions had complete statistics for IV, DV1, and DV2. In Figure 3A, the United States of America and Myanmar have the lowest inspector ratios of 0.07, and San Marino has the highest inspector ratio of 3.89 among all the regions. San Marino also reports zero fatal injuries per 100,000 workers.

Figure 3B, with standard error bars, represents the OSH inspectors per 10,000 employed in 69 regions and four groups (1: high-income regions, 2: upper middle-income regions, 3: lower-middle-income regions, and 4: low-income regions) globally, and also illustrates the performance of the OSH inspectors, such as how many workplaces the OSH inspectors visited monthly and annually. Note that this does not include a total of 19 regions that did not have complete statistics (i.e., visits per inspector, visits in the year, workplaces liable to labor inspection, inspectors per 10,000 employed persons, and number of labor inspectors by sex) in the ILO’s table associated with Figure 3B.

The current trend analysis of inspector ratios indicates that not all regions or economies, categorized as group 1 (industrial economies), group 2 (transition economies), and groups 3 and 4 (less developed economies) according to the ILO’s press release (24), met the ILO’s expectations.

See Table 4 ILO’s expectations vs. current trends for details.

**Table 4:** ILO’s expectations v/s current trends.

ILO expectations	Group 1 (45)	Group 2 (26)	Group 3 and 4 (14)	Malaysia	Saskatchewan
Meet expectations	5	4	-	Yes	–
Exceed expectations	17	11	10	–	Yes
Lag expectations	23	11	4	–	–

This study created this table as per the ILO’s statistics, including Malaysia and Saskatchewan.

As shown in this table, groups 1 and 2 had 45 and 26 regions, respectively. Fourteen geographical regions participated in the combined groups of 3 and 4. Malaysia and Saskatchewan also participated in this comparison of ILO’s expectations v/s current inspector ratio trends.

In group 1, there were 5 regions meeting the minimum expectations with 0.90 to 1.0 inspector per 10,000 employed persons, 17 regions exceeded the ILO’s expectations on inspector ratios, and 23 regions appeared to be understaffed as per the ILO’s press release in 2006. In group 2, there were 4 regions meeting the minimum expectations with 0.40 to 0.50 inspector per 10,000 employed persons, 11 regions exceeded the expectations with greater than 0.50 inspector per 10,000 employed persons, and 11 regions were understaffed with less than 0.40 inspector per 10,000 employed persons. Interestingly, the majority (10) of regions in the combined group of 3 and 4 exceeded the expectations with greater than 0.25 inspector per employed person and 4 regions did not meet the expectations of inspector ratios. Also, Malaysia met the expectations, and Saskatchewan exceeded the expectations of the ILO press release in International Labour Organization (28).

In group 1, there were 5 regions meeting the minimum expectations with 0.90 to 1.0 inspector per 10,000 employed persons, 17 regions exceeded the ILO’s expectations on inspector ratios, and 23 regions appeared to be understaffed according to the ILO’s press release in 2006. In group 2, there were 4 regions meeting the minimum expectations with 0.40 to 0.50 inspector per 10,000 employed persons, 11 regions exceeded the expectations with greater than 0.50 inspectors per 10,000 employed persons, and 11 regions were understaffed with less than 0.40 inspectors per 10,000 employed persons. Interestingly, the majority (10) of regions in the combined group of 3 and 4 exceeded the expectations with greater than 0.25 inspectors per employed persons, while 4 regions did not meet the expectations for inspector ratios. Additionally, Malaysia met the expectations, and Saskatchewan exceeded the expectations of the ILO press release (28).

Thus, this study builds upon the recent study of Gammarano (24). With this study, readers have access to the ILO’s complex numerical statistics and the visual trends simultaneously on inspector ratio trends, including Saskatchewan, which was also not part of Gammarano (24).

Based on Table 3, this study created Malaysia’ longitudinal inspector ratios and trends of OSH injuries and fatalities.

See Figure 4 Malaysia’s OSH inspector ratios and impact for details.

Figure 4, with standard error bars, represents year-after-year inspector ratios and workplace human suffering or tragedies in Malaysia since 2015.

Then, this research used the statistics mentioned in Table 3 to visualize Saskatchewan’s inspector ratios and impact in the region.

See Figure 5 Saskatchewan’s inspector ratios and impact for details.

Figure 5, with standard error bars, represents Saskatchewan’s multi-year annual trends of inspector ratios and their impact on fatal and non-fatal workplace injuries. Figure 5 indicates that higher inspector ratios might not always improve sustainable OSH performance. While the inspector ratios were stable (1 to 1.30), recent workplace injuries showed a downward trend, as illustrated in Figure 5 for Saskatchewan.

The following section will report the results of the multivariate regression analysis.

### 3.2 Multivariate regression analysis

This section briefly describes and discusses the findings of general linear multivariate (GLM) assumption tests and the results of multivariate regression analysis performed with IBM SPSS 30.0. To address limitations or fallacies in the secondary ecological data used, this study performed:

*Sensitivity analysis:* This study observed outliers or extreme values in the research variables and conducted sensitivity analysis by analyzing Cook’s distance and leverage values associated with those particular outliers or extreme values. This study re-ran the regression(s) after removing any influential data from the regression analysis.

*Robustness:* Originally, groups 1, 2, 3, and 4 had 43, 21, and 12 (groups 3 and 4 combined = 12) data points of annual aggregated statistics. Additionally, Saskatchewan and Malaysia each had 8 years of annual aggregated statistics. Therefore, to address issues of robustness in the research, such as small sample sizes and non-normality, this study performed bootstrap-2000 with confidence intervals of 95% for those ILO groups, including Malaysia and Saskatchewan.

*Log-transformation of skewed data:* This study observed that the statistics in Table 1 were skewed. Therefore, this study performed data transformation, especially log-transformation of dependent variables, and included log-transformed research variables in the descriptive statistics and regression analysis.

*External validity:* This was another issue the ecological secondary statistics faced in this study. To address the external validity issue, this study not only compared the findings of regression analysis across the ILO groups, Malaysia, and Saskatchewan but also investigated whether the findings of this research validated any existing literature or vice versa.

The above approach to addressing the limitations in the ecological research data was not a new approach in scientific research, including public health and workplace safety and health.

#### 3.2.1 Assumptions tests for regression analysis

This study utilized the statistics associated with the inspector ratios (also known as “Inspector_ratio”), fatal injury rates (a.k.a. “Fatal_rate”), and non-fatal injury rates (also known as “Nonfatal_rate”) as outlined in Table 1 to compute group-specific multivariate regression analysis. There was a total of 120 regions listed in Table 1 that were further classified into four groups. However, due to missing statistics, this study was unable to include all the regions in the multivariate regression analysis to evaluate the feasibility of inspector ratios or to assess the impact of inspector ratios (IV) on dependent variables (DV1 and DV2).

This study completed the assumption tests and multivariate regression for each group, including Malaysia and Saskatchewan, in six major steps.

Step 1: After running initial descriptive statistics analysis (using the ‘explore’ option within SPSS 30.0), this study analyzed box plots, PP-Plots, QQ-Plots, scatter plots/residual normality, normality test scores of Shapiro–Wilk tests, and outliers. Despite observing outliers visually, this study did not remove them right away but performed sensitivity analysis and made an informed decision regarding when/if to remove any outliers or influential data points from the further regression analysis.
Step 2: Then, this study ran the first multivariate regression analysis and checked the Cook’s distance and leverage values as well. SPSS 30.0 did not output the Cook’s distance and leverage values for each regression but analyzed them in the ‘data view’ page of SPSS. Consistent with the literature, this study generally followed 4 divided by the number of samples (n) as the threshold values to accept or remove data points after the investigation of such values in further regression analysis. If any cases arose, this study removed data points due to higher Cook’s distance or leverage scores and ran and reran the regression with the reduced data.
Steps 3: Then, this study ran multivariate regression with bootstrap-2000 with confidence intervals of 95%.
Step 4: Then, this study log-transformed the dependent variables and ran descriptive statistics, repeating step 1 in this case.
Step 5: Then, this study ran the first log-multivariate regression with bootstrap-200 with confidence intervals of 95% and performed a similar analysis as in step 2.
Step 6: Then, this study ran log-multivariate regression analysis.

Detailed descriptive statistics and regression analysis on each group, including Malaysia, and Saskatchewan are accessible through Supplementary documents attached to this report.

For details, see Supplementary documents titled:

- Supplementary document: Group 1: Descriptive statistics and multivariate analysis,
- Supplementary document: Group 2: Descriptive statistics and multivariate analysis,
- Supplementary document: Groups 3 and 4: Descriptive statistics and multivariate analysis,
- Supplementary document: Malaysia: Descriptive statistics and multivariate analysis, and
- Supplementary document: Saskatchewan: Descriptive statistics and multivariate analysis.

See Table 5 Results of tests of normality for details.

**Table 5:** Results of test of normality.

Group 1 (Without and with log transformation of dependent variables)						
Before sensitivity analysis: *N* = 43	Kolmogorov–Smirnov^a^			Shapiro–Wilk		
	Statistic	df.	Sig.	Statistic	df.	Sig.
Inspector_ratio (IV)	0.171	43	0.003	0.804	43	<0.001
Fatal_rate (DV1)	0.144	43	0.025	0.867	43	<0.001
Nonfatal_rate (DV2)	0.194	43	<0.001	0.688	43	<0.001
After sensitivity analysis, total 9 data points were deleted. Log-transformed with reduced data for further analysis.						
Inspector_ratio (IV)	0.116	34	0.200^*^	0.948	34	0.106
Log_fatalrate (DV1)	0.113	34	0.200^*^	0.911	34	0.009
Log_nonfatalrate (DV2)	0.163	34	0.023	0.847	34	<0.001

Group 2 (Without and with log transformation of dependent variables)						
Normality test—before sensitivity analysis: *N* = 21.						
Inspector_ratio (IV)	0.260	21	<0.001	0.774	21	<0.001
Fatal_rate (DV1)	0.229	21	0.005	0.818	21	0.001
Nonfatal_rate (DV2)	0.257	21	<0.001	0.703	21	<0.001
After sensitivity analysis, total “1” data point was deleted. Log-transformed with reduced data for further analysis.						
Inspector_ratio (IV)	0.274	20	<0.001	0.774	20	<0.001
Log_fatalrate	0.182	20	0.081	0.875	20	0.014
Log_nonfatalrate	0.150	20	0.200^*^	0.916	20	0.082

Groups 3 and 4 (Without and with log transformation of dependent variables)						
Normality test—before sensitivity analysis: *N* = 12.						
Inspector_ratio (IV)	0.092	12	0.200^*^	0.966	12	0.868
Fatal_rate (DV1)	0.174	12	0.200^*^	0.878	12	0.082
Nonfatal_rate (DV2)	0.325	12	0.001	0.629	12	<0.001
After sensitivity analysis, but, log-transformed was performed with original data.						
Inspector_ratio (IV)	0.092	12	0.200^*^	0.966	12	0.868
Log_fatalrate	0.258	12	0.026	0.900	12	0.158
Log_nonfatalrate	0.112	12	0.200^*^	0.948	12	0.605

Malaysia (Without and with log transformation of dependent variables)						
Normality test—before sensitivity analysis: *N* = 8.						
Inspector_ratio (IV)	0.146	8	0.200^*^	0.985	8	0.983
Fatal_rate (DV1)	0.307	8	0.025	0.787	8	0.021
Nonfatal_rate (DV2)	0.229	8	0.200^*^	0.879	8	0.185
After sensitivity analysis, but, log-transformed was performed with original data for further analysis.						
Inspector_ratio (IV)	0.146	8	0.200^*^	0.985	8	0.983
Log_fatalrate	0.306	8	0.027	0.773	8	0.014
Log_nonfatalrate	0.276	8	0.074	0.811	8	0.038

Saskatchewan (Without and with log transformation of dependent variables)						
Inspector_ratio (IV)	0.198	8	0.200^*^	0.950	8	0.711
Fatal_rate (DV1)	0.168	8	0.200^*^	0.898	8	0.274
Nonfatal_rate (DV2)	0.157	8	0.200^*^	0.938	8	0.594
After sensitivity analysis, but, log-transformed was performed with original data for further analysis.						
Inspector_ratio (IV)	0.198	8	0.200^*^	0.950	8	0.711
Log_fatalrate	0.147	8	0.200^*^	0.929	8	0.504
Log_nonfatalrate	0.136	8	0.200^*^	0.941	8	0.619

Normality test results of groups 1, 2, 3, and 4, Malaysia, and Saskatchewan.

*This is a lower bound of the true significance.

^a^Lilliefors Significance Correction.

The following paragraphs will briefly describe the results of the normality tests.

*Group 1*: Among the total of 61 ILO regions in group 1, only 43 ILO regions had complete datasets for IV, DV1, and DV2, while 18 ILO regions had missing datasets that were removed from the final general linear multivariate (GLM) regression analysis. Additionally, five outliers were removed from the regression analysis. This study initially observed a few outliers; however, through the sensitivity analysis or analysis of Cook’s distance and leverage values, this study briefly investigated research data associated with Germany, Luxembourg, and Romania in Table 1 because these nations had a Cook’s distance greater than 0.0930 (i.e., 4/43 samples = 0.0930). This study removed Germany, Luxembourg, and Romania from further regression analysis, as each of these nations had incomplete data or discrepancies in reporting as per Table 1. Following a similar approach, after rerunning the analysis, this study also removed the data associated with Belgium, Finland, Lithuania, Seychelles, Qatar, and the United States, following a similar analysis and rerun of the regression. Thus, in total, 9 data points were removed in the final regression analysis based on the sensitivity analysis.

In the initial assessment of normality test scores with the original data (43 data points), the Shapiro–Wilk test value associated with Fatal_rate was 0.867, *p*-value = <0.001, and the Shapiro–Wilk test value associated with Nonfatal_rate was 0.688, *p*-value = <0.001. In contrast, with the reduced data (total of 34 data points) from the log-transformation of dependent variables, the Shapiro–Wilk test scores associated with Log_fatalrate = 0.911 (improved), *p*-value = 0.009, and the Shapiro–Wilk test scores associated with Log_nonfatalrate = 0.847 (improved), *p*-value = <0.001.

*Group 2*: The total number of regions participating in group 2 was 21, with 10 regions having missing statistics and six regions with outliers removed from the multivariate regression. Therefore, only 15 regions qualified to be part of the multivariate regression. As in group 1, this study observed several outliers and followed a similar sensitivity analysis for group 2. All the data points had a Cook’s distance value of less than 0.1904 (Cook’s distance threshold for this group B: 4/21 samples = 0.1904), but Costa Rica (0.29) and Cuba (0.24) had slightly higher values. This study briefly investigated these two countries in Table 1 and determined to remove Costa Rica from further regression analysis, while including Cuba in the further regression analysis because this study assumed that Cuba did not have reporting errors (A and A’ in Table 1) as per Table 1, and its Cook’s distance was much lower than 0.5, although certain statistics for this country were missing in Table 1.

In the initial assessment of normality test scores with the original data (21 data points), the Shapiro–Wilk test value associated with Fatal_rate was 0.818, *p*-value = 0.001, and the Shapiro–Wilk test value associated with Nonfatal_rate was 0.703, *p*-value = <0.001. In contrast, with the reduced data (total of 20 data points) from the log-transformation of dependent variables, the Shapiro–Wilk test scores associated with Log_fatalrate = 0.875 (improved), *p*-value = 0.014 (improved), and the Shapiro–Wilk test scores associated with Log_nonfatalrate = 0.916 (improved), *p*-value = <0.082 (improved).

*Groups 3 and 4*: Due to the small sample size in groups 3 and 4, this research combined both groups and performed a single normality test for groups 3 and 4. Among the 27 ILO regions in groups 3 and 4, a total of 15 regions with missing values were removed from the multivariate regression analysis. For these groups 3 and 4, the Cook’s distance threshold considered by this study was 0.3333 (4/12 samples = 0.3333) for sensitivity analysis. Despite several outliers, only one data point associated with Jordan had a higher Cook’s distance (0.42) than the threshold (0.3333). However, this data point was treated similarly to those in group B’s Cuba, Malaysia, and Saskatchewan, and, thus, was included in the further regression analysis.

In the initial assessment of normality test scores with the original data (12 data points), the Shapiro–Wilk test value associated with Fatal_rate was 0.878, *p*-value = 0.082, and the Shapiro–Wilk test value associated with Nonfatal_rate was 0.629, *p*-value = <0.001. In contrast, with the log-transformed original data points (total 12 data points) of dependent variables, the Shapiro–Wilk test scores associated with Log_fatalrate = 0.900 (improved), *p*-value = 0.158 (improved), and the Shapiro–Wilk test scores associated with Log_nonfatalrate = 0.948 (improved), *p*-value = <0.605 (improved).

*Malaysia*: There were eight valid datasets for eight different years, including 2023 (see Table 2), and all datasets were included in this region’s multivariate regression analysis.

For this region, the Cook’s distance threshold considered for sensitivity analysis by this study was 0.5 (4/8 samples = 0.5). This study observed two annual data points associated with 2017 (Cook’s distance = 0.87) and 2022 (Cook’s distance = 0.73) that exceeded the threshold for this region. Since this study sought and obtained verification of the research data associated with Malaysia from the Department of Occupational Safety and Health, Malaysia, it was decided not to remove any data points from the further regression analysis.

In the initial assessment of normality test scores with the original data (8 data points), the Shapiro–Wilk test value associated with Fatal_rate was 0.787, *p*-value = 0.021, and the Shapiro–Wilk test value associated with Nonfatal_rate was 0.879, *p*-value = 0.185. In contrast, with the log-transformed original data points (total 8 data points) of dependent variables, the Shapiro–Wilk test scores associated with Log_fatalrate = 0.773 (slightly reduced), *p*-value = 0.014 (reduced), and the Shapiro–Wilk test scores associated with Log_nonfatalrate = 0.811 (reduced), *p*-value = <0.038 (reduced).

*Saskatchewan*: There were eight valid datasets for eight different years, including 2024 (see Table 3), and all datasets were included in this region’s multivariate regression analysis. This study neither observed any outliers nor were the Cook’s distances higher than the threshold of 0.5 (4/8 samples = 0.5). Therefore, all data points were included in the regression analysis for this region. In the initial assessment of normality test scores with the original data (8 data points), the Shapiro–Wilk test value associated with Fatal_rate was 0.898, *p*-value = 0.274, and the Shapiro–Wilk test value associated with Nonfatal_rate was 0.938, *p*-value = 0.594. In contrast, with the log-transformed original data points (total 8 data points) of dependent variables, the Shapiro–Wilk test scores associated with Log_fatalrate = 0.929 (improved), *p*-value = 0.504 (improved), and the Shapiro–Wilk test scores associated with Log_nonfatalrate = 0.941 (slightly reduced), *p*-value = <0.619 (improved).

The following section 3.2.2 will report on the results and briefly discuss them.

#### 3.2.2 Results of multivariate regression analysis

This study performed four major regression models: (1) general linear multivariate regression, (2) general linear multivariate regression with bootstrap-2000 (95%CIs), (3) log-general linear multivariate regression with bootstrap-2000 (95%CIs), and (4) log-general linear multivariate regression. Additionally, this study performed multiple general linear multivariate regression analyses for group 1 as part of a sensitivity analysis, while for group 2, this study completed one additional general linear multivariate regression while analyzing research data sensitivity. Thus, a total of over 20 regression analyses were performed using IBM SPSS 30.0 (four regressions for each group—groups 1, 2, 3, and 4, Malaysia, and Saskatchewan) using the statistics from Table 1 for ILO regions of groups 1, 2, 3, and 4, while statistics in Tables 2, 3 were used for Malaysia and Saskatchewan, respectively.

The following paragraphs will briefly describe the results of the regression analysis.

*Group 1*: GLM regression, initially with a sample size (*N*) = 43, revealed that inspector ratios did not have a strong relationship with the combined variable (i.e., SDG 8.8.1) or fatal and non-fatal injury rates (Wilk’s Lambda = 0.949, *F*(2, 40) = 1.072, sig. = 0.352, power = 0.225). Additionally, the tests of between-subjects effects, such as the association between inspector ratios and fatal injury rates (adjusted *R*^2^ = −0.021, sig. = 0.721), and inspector ratios and non-fatal injury rates (adjusted *R*^2^ = −0.011, sig. = 0.234), indicated that inspector ratios alone might not be meaningful predictors of decent workplace safety and health in the case of group 1.

After the first regression, this study identified three influential data points during sensitivity analysis and then reran the regression analysis with a reduced sample (*N* = 40). The regression analysis did not result in any significant relationship between inspector ratios and SDG 8.8.1 (Wilk’s Lambda = 0.965, *F*(2, 37) = 0.662, sig. = 0.522, power = 0.153). Additionally, the tests of between-subjects effects—inspector ratios and fatal injury rates (adjusted *R*^2^ = −0.013, sig. = 0.487) and inspector ratios and non-fatal injury rates (adjusted *R*^2^ = −0.020, sig. = 0.639)—indicated a weak association between the subjects.

This study performed multiple rounds of sensitivity analysis and removed three additional data points during each rerun of the regression with SPSS 30.0 based on their potential influence. Each rerun of the regression (final regression sample size = 34) yielded similar results as reported above, showing a non-significant statistical relationship between inspector ratios and SDG 8.8.1, as well as an association between inspector ratios and fatal injury rates and inspector ratios and non-fatal injury rates for group 1.

Then, this study performed GLM regression with bootstrap-2000 (95% CIs) with the reduced data or sample size (*N*) = 34 to assess the robustness of the regression analysis, which also resulted in a nonsignificant impact of inspector ratios (Wilk’s Lambda = 0.964, *F*(2, 31) = 0.576, sig. = 0.568, power = 0.137) on decent workplace safety or both dependent variables. Additionally, the tests of between-subjects effects—inspector ratios and fatal injury rates (adjusted *R*^2^ = −0.021, sig. = 0.580), and inspector ratios and non-fatal injury rates (adjusted *R*^2^ = 0.003, sig. = 0.301)—indicated weak associations between the subjects.

Then, this study log-transformed the skewed reduced research data of dependent variables with a sample size (*N*) = 34 and performed log-GLM regression with bootstrap-2000 (95% CIs). The regression analysis produced similar results this time as well. In other words, there was a nonsignificant impact of inspector ratios (Wilk’s Lambda = 0.956, *F*(2, 31) = 0.719, sig. = 0.495, power = 0.160) on both log_fatal injury rates and log_nonfatal injury rates. The tests of between-subjects effects—inspector ratios and log_fatal injury rates (adjusted *R*^2^ = −0.013, sig. = 0.457), and inspector ratios and log_nonfatal injury rates (adjusted *R*^2^ = 0.006, sig. = 0.283)—indicated a still weak association between the subjects in group 1.

Finally, this study again performed log-GLM regression with a sample size (*N*) = 34 (without bootstrap-2000 at 95% CIs). This regression validated the previous regression results mentioned above for this group 1, and this multivariate model also did not significantly predict the effects of inspector ratios (sig. = 0.495) on both log_fatal injury rates and log_nonfatal injury rates. The tests of between-subjects effects—inspector ratios and log_fatal injury rates (sig. = 0.457), and inspector ratios and log_nonfatal injury rates (sig. = 0.283)—indicated a still weak association between the subjects in group 1.

*Group 2*: GLM regression, initially with a sample size (*N*) = 21, revealed that inspector ratios did not have a strong relationship with the combined variable (i.e., SDG 8.8.1), or fatal and non-fatal injury rates (Wilk’s Lambda = 0.956, *F*(2, 18) = 0.412, sig. = 0.668, power = 0.107). Additionally, the tests of between-subjects effects, such as the association between inspector ratios and fatal injury rates (adjusted *R*^2^ = −0.048, sig. = 0.767), and inspector ratios and non-fatal injury rates (adjusted *R*^2^ = −0.007, sig. = 0.363), indicated that inspector ratios alone might not be meaningful predictors of decent workplace safety and health in the case of group 2, as in group 1.

After the first regression, this study identified/removed one influential data point during sensitivity analysis and then reran the regression analysis with a reduced sample (*N*) = 20. Yet, the regression analysis did not result in any significant relationship between inspector ratios and SDG 8.8.1 (Wilk’s Lambda = 0.914, *F*(2, 17) = 0.796, sig. = 0.467, power = 0.163). Additionally, the tests of between-subjects effects—inspector ratios and fatal injury rates (adjusted *R*^2^ = −0.051, sig. = 0.781), and inspector ratios and non-fatal injury rates (adjusted *R*^2^ = −0.034, sig. = 0.212)—indicated weak associations between the subjects.

Then, this study performed GLM regression with bootstrap-2000 (95% CIs) with the reduced data or sample size (*N*) = 20 to assess the robustness of the regression analysis, which also resulted in a nonsignificant impact of inspector ratios (Wilk’s Lambda = 0.914, *F*(2, 17) = 0.796, sig. = 0.467, power = 0.163) on decent workplace safety or both dependent variables. Additionally, the tests of between-subjects effects—inspector ratios and fatal injury rates (adjusted *R*^2^ = 0.034, sig. = 0.212), and inspector ratios and non-fatal injury rates (adjusted *R*^2^ = −0.051, sig. = 0.781)—indicated a weak association between the subjects.

Then, this study log-transformed the skewed reduced research data of dependent variables with a sample size (*N*) = 20 and performed log-GLM regression with bootstrap-2000 (95% CIs). The regression analysis produced similar results this time, too, as above in groups 1 and 2. In other words, there was a nonsignificant impact of inspector ratios (Wilk’s Lambda = 0.892, *F*(2, 17) = 1.032, sig. = 0.378, power = 0.200) on both log_fatal injury rates and log_nonfatal injury rates. The tests of between-subjects effects—inspector ratios and log_fatal injury rates (adjusted *R*^2^ = −0.013, sig. = 0.741), and inspector ratios and log_nonfatal injury rates (adjusted *R*^2^ = −0.012, sig. = 0.392)—indicated a still weak association between the subjects in group 2.

Finally, this study again performed log-GLM regression with a sample size (*N*) = 20 (without bootstrap-2000 at 95% CIs). This regression validated the previous regression results mentioned above for this group 2, and this multivariate model also did not significantly predict effects of inspector ratios (sig. = 0.378) on both log_fatal injury rates and log_nonfatal injury rates. The tests of between-subjects effects—inspector ratios and log_fatal injury rates (sig. = 0.741), and inspector ratios and log_nonfatal injury rates (sig. = 0.392)—indicated a still weak association between the subjects in group 2.

*Groups 3 and 4*: An initial GLM regression with a sample size (*N*) = 12 revealed that inspector ratios did not have a strong relationship with the combined variable (i.e., SDG 8.8.1), or fatal and non-fatal injury rates (Wilk’s Lambda = 0.949, *F*(2, 9) = 0.053, sig. = 0.949, power = 0.056). Additionally, the tests of between-subjects effects, such as the association between inspector ratios and fatal injury rates (adjusted *R*^2^ = −0.088, sig. = 0.744), and inspector ratios and non-fatal injury rates (adjusted *R*^2^ = −0.099, sig. = 0.925), indicated that inspector ratios alone might not be meaningful predictors of decent workplace safety and health in the case of groups 3 and 4, as in groups 1 and 2.

After the first regression in groups 3 and 4, this study performed a sensitivity analysis but determined that no further re-runs of GLM regression were required for this group.

Then, this study performed GLM regression with bootstrap-2000 (95% CIs) with the original sample size (*N*) = 12 to assess the robustness of the regression analysis, which also resulted in a nonsignificant impact of inspector ratios (Wilk’s Lambda = 0.988, *F*(2, 9) = 0.053, sig. = 0.949, power = 0.056) on both dependent variables. Additionally, the tests of between-subjects effects—inspector ratios and fatal injury rates (adjusted *R*^2^ = −0.088, sig. = 0.744), and inspector ratios and non-fatal injury rates (adjusted *R*^2^ = −0.099, sig. = 0.925)—indicated a weak association between the subjects.

Then, this study log-transformed the skewed research data of dependent variables with a sample size (*N*) = 12 and performed log-GLM regression with bootstrap-2000 (95% CIs). The regression analysis produced similar results this time, too, as above in groups 1, 2, 3, and 4. In other words, there was a nonsignificant impact of inspector ratios (Wilk’s Lambda = 0.960, *F*(2, 9) = 0.187, sig. = 0.832, power = 0.071) on both log_fatal injury rates and log_nonfatal injury rates. The tests of between-subjects effects—inspector ratios and log_fatal injury rates (adjusted *R*^2^ = −0.100, sig. = 0.971), and inspector ratios and log_nonfatal injury rates (adjusted *R*^2^ = −0.072, sig. = 0.622)—indicated a still weak association between the subjects in groups 3 and 4.

Finally, this study again performed log-GLM regression with a sample size (*N*) = 12 (without bootstrap-2000 at 95% CIs). This regression also validated the previous regression results mentioned above for groups 3 and 4, and this multivariate model also did not significantly predict the effects of inspector ratios (sig. = 0.832) on both log_fatal injury rates and log_nonfatal injury rates. The tests of between-subjects effects—inspector ratios and log_fatal injury rates (sig. = 0.971), and inspector ratios and log_nonfatal injury rates (sig. = 0.622)—indicated a still weak association between the subjects in groups 3 and 4.

*Malaysia*: An initial GLM regression with a sample size (*N*) = 8 revealed that inspector ratios had a comparatively stronger relationship with the combined variable (i.e., SDG 8.8.1), or fatal and non-fatal injury rates (Wilk’s Lambda = 0.306, *F*(2, 5) = 5.677, sig. = 0.052, power = 0.587) than other groups discussed above. Additionally, the tests of between-subjects effects, such as the association between inspector ratios and fatal injury rates (adjusted *R*^2^ = 0.174, sig. = 0.167), and inspector ratios and non-fatal injury rates (adjusted *R*^2^ = −0.164, sig. = 0.915) indicated that inspector ratios alone might not be meaningful predictors of decent workplace safety and health in the case of groups 3 and 4, as in groups 1 and 2 above.

After the first regression for Malaysia, this study performed a sensitivity analysis but determined that no further re-runs of GLM regression were required for this group since the accuracy of the research data was verified by the Department of Occupational Safety and Health, Malaysia.

Then, this study performed GLM regression with bootstrap-2000 (95% CIs) with the original sample size (*N* = 8) to assess the robustness of the regression analysis, which also resulted in a significant impact of inspector ratios (sig. = 0.052) on both dependent variables. Additionally, the tests of between-subjects effects—inspector ratios and fatal injury rates (sig. = 0.167), and inspector ratios and non-fatal injury rates (sig. = 0.915)—indicated a weak association between the subjects.

Then, this study log-transformed the skewed original research data of dependent variables with a sample size (*N* = 8) and performed log-GLM regression with bootstrap-2000 (95% CIs). There was a nonsignificant impact of inspector ratios (Wilk’s Lambda = 0.356, *F*(2, 5) = 4.531, sig. = 0.832, power = 0.075) on both log_fatal injury rates and log_nonfatal injury rates. The tests of between-subjects effects—inspector ratios and log_fatal injury rates (adjusted *R*^2^ = 0.135, sig. = 0.198), and inspector ratios and log_nonfatal injury rates (adjusted *R*^2^ = −0.151, sig. = 0.786)—indicated a still weak and contradictory association between the subjects in groups 3 and 4.

Finally, this study again performed log-GLM regression with a sample size (*N* = 8) (without bootstrap-2000 at 95% CIs). This multivariate model also did not significantly predict effects of inspector ratios (sig. = 0.075) on both log_fatal injury rates and log_nonfatal injury rates. The tests of between-subjects effects—inspector ratios and log_fatal injury rates (sig. = 0.198), and inspector ratios and log_nonfatal injury rates (sig. = 0.786)—indicated a still weak association between the subjects in Malaysia.

*Saskatchewan*: An initial GLM regression with a sample size (*N* = 8) revealed that inspector ratios did not have a strong relationship with the combined variable (i.e., SDG 8.8.1) or fatal and non-fatal injury rates (Wilk’s Lambda = 0.671, *F*(2, 5) = 1.224, sig. = 0.369, power = 0.167). Additionally, the tests of between-subjects effects, such as the association between inspector ratios and fatal injury rates (adjusted *R*^2^ = −0.141, sig. = 0.728), and inspector ratios and non-fatal injury rates (adjusted *R*^2^ = 0.017, sig. = 0.330)—indicated that inspector ratios alone might not be meaningful predictors of decent workplace safety and health in the case of Saskatchewan.

After the first regression for Saskatchewan, this study performed a sensitivity analysis but determined that no further re-runs of GLM regression were required for Saskatchewan. Additionally, the internal inspector ratios data were provided by the Department of Occupational Safety and Health, Saskatchewan.

Then, this study performed GLM regression with bootstrap-2000 (95% CIs) with the original sample size (*N* = 8) to assess the robustness of the regression analysis, which also resulted in a nonsignificant impact of inspector ratios (sig. = 0.369) on both dependent variables. Additionally, the tests of between-subjects effects—inspector ratios and fatal injury rates (sig. = 0.728), and inspector ratios and non-fatal injury rates (sig. = 0.330)—indicated a weak association between the subjects.

Then, this study log-transformed the original research data of dependent variables with a sample size (N) of 8 and performed log-GLM regression with bootstrap-2000 (95% CIs). There was a nonsignificant impact of inspector ratios (Wilk’s Lambda = 0.666, *F*(2, 5) = 1.254, sig. = 0.362, power = 0.170) on both log_fatal injury rates and log_nonfatal injury rates. The tests of between-subjects effects—inspector ratios and log_fatal injury rates (adjusted *R*^2^ = −0.117, sig. = 0.623), and inspector ratios and log_nonfatal injury rates (adjusted *R*^2^ = −0.010, sig. = 0.371)—indicated a weak and contradictory association between the subjects in Saskatchewan’s regression analysis.

Finally, this study again performed log-GLM regression with a sample size (N) of 8 (without bootstrap-2000 at 95% CIs). This multivariate model also did not significantly predict the effects of inspector ratios (sig. = 0.362) on both log_fatal injury rates and log_nonfatal injury rates. The tests of between-subjects effects—inspector ratios and log_fatal injury rates (sig. = 0.623), and inspector ratios and log_nonfatal injury rates (sig. = 0.371)—indicated a weak association between the subjects in group 2.

For details of the GLM regression analysis for groups 1, 2, 3, 4, Malaysia, and Saskatchewan, see the Supplementary documents titled:

- Supplementary document: Group 1: Descriptive and multivariate analysis,
- Supplementary document: Group 2: Descriptive and multivariate analysis,
- Supplementary document: Groups 3 and 4: Descriptive and multivariate analysis,
- Supplementary document: Malaysia: Descriptive and multivariate analysis, and
- Supplementary document: Saskatchewan: Descriptive and multivariate analysis.

Thus, as discussed above, inspector ratios neither significantly influence SDG 8.8.1, also known as combined workplace injury rates, reliably, nor do the effects of inspector ratios consistently go in the same directions across all the groups. For perspectives, as per the parameter tables, GLM standard regression in group 1 (*B* = −0.123 for fatal_rate, and 354.412 for nonfatal_rate), group 2 (*B* = −0.732 for fatal_rate and −874.563 for nonfatal_rate), groups 3 and 4 (*B* = 1.875 for fatal_rate and 86.029 for nonfatal_rate), Malaysia (*B* = 30.378 for fatal_rate and −87.646 for nonfatal_rate), and Saskatchewan (*B* = 4.266 for fatal_rate and −3381.681 for nonfatal_rate). Additionally, refer to Figures 6a,b to compare the residual plots across the groups, including Malaysia and Saskatchewan. In other words, this study explored the associations between the research variables and fell short of claiming a causal relationship between them. Based on the findings of the GLM regression (multivariate or univariate associations) across the ILO regions investigated in this study, including Malaysia and Saskatchewan, the external validities across the groups were statistically weak and not reliable, which was ironically consistent with other literature, such as in Gómez-García et al. (25).

In conclusion, this study had limitations, such as the smaller sample sizes for the regression analysis, the ecological secondary nature of datasets, and observed discrepancies in the datasets, especially in the case of many ILO regions, including Malaysia. This research cautiously reported that the findings of the 20-plus multivariate regression analyses performed in this study were statistically nonsignificant, even though Malaysia’s GLM regression discovered, to some extent, a strong or significant association (sig. = 0.052) within the model for benchmarking the inspector ratios. Also, note that a pre- and post-regression power analysis would not have added any significant value to this study since it did not use “sample(s)” but the statistics associated with their populations as reported by the regional official sources. This report discussed the results of descriptive statistics briefly in the following section 3.3.

### 3.3 Descriptive statistics analysis

This study computed descriptive statistics, including *median values* associated with only the inspector ratios, as shown in Table 1, for group 1, group 2, group 3, group 4, Malaysia, and Saskatchewan, using IBM SPSS 30.0 software. A total of 85 ILO regions had inspector ratios in Table 1. Any ILO region that had the inspector ratio in Table 1, despite missing DV1 or DV2 values, was considered for descriptive statistics analysis. In other words, this research did not require consideration of any sensitivity analysis of this single descriptive variable (i.e., the inspector ratios), and the calculated descriptive statistics associated with this variable were fixed.

See Table 6 Results of descriptive statistics analysis for details.

**Table 6:** Results of descriptive statistics.

		Group 1	Group 2	Groups 3 and 4	Malaysia	Saskatchewan	Global_ILO_regions
N	Valid	45	26	14	8	8	85
Mean		0.986000	0.652308	0.473571	0.751300	1.157200	0.7995
Std. error of mean		0.1157838	0.1134200	0.0724880	0.0081439	0.0234691	0.07436
Median		0.870000	0.440000	0.495000	0.748150	1.138950	0.5800
Std. DEVIATION		0.7767017	0.5783308	0.2712253	0.0230344	0.0663805	0.68554
Skewness		1.925	1.642	0.090	0.165	0.087	2.089
Std. Error of Skewness		0.354	0.456	0.597	0.752	0.752	0.261
Kurtosis		4.515	2.684	−1.085	0.144	0.029	5.7424
Std. Error of Kurtosis		0.695	0.887	1.154	1.481	1.481	0.517

“N” stands for the total number of regions or annual data points. In the case of Malaysia and Saskatchewan, “N” represents the total annual data points used to calculate the descriptive statistics.

As shown in this table, groups 1 and 2 had 45 and 26 regions, respectively. Fourteen geographical regions participated in the combined groups of 3 and 4. Malaysia and Saskatchewan also participated in this descriptive statistics analysis. Global_ILO_regions represented a total of 85 ILO regions, excluding Saskatchewan. In the case of Malaysia, descriptive statistics analysis was performed based on Table 2, separately, and the Malaysian stats were also mentioned in Table 1, which included 85 ILO regions.

As shown in Table 6, though it was mathematically or theoretically possible to compute descriptive statistics associated with ILO’s 85 regions (also known as “Global_ILO_regions”) using SPSS 30.0 software, this study avoided recommending or practicing a standard inspector ratio based solely on descriptive statistics due to confounders like economic gaps and vast differences in regional or continental OSH cultures and demographics, and the fact that inspectors in many countries may not be adequately staffed, trained, or equipped to enforce standards in the informal economy (22, p. 216).

Based on the descriptive statistics computed in Table 6 and consistent with past literature in benchmarking, such as workplace safety scores (60), *the relationship between OSH inspectors and OSH injuries* (29), predicting future trends and consequences (61, 62), sustainability assessment (63), policy decision-making, evaluation of OSH performance (64), and assessment of nurse staffing coverage (65), this study recommended three ratings of the inspector ratios based on *median* value(s). This study’s three inspector ratio *ratings—poor, average, and* good—were inspired by OECD’s (36) approach on the “rating system” (p. 10), which was also distinct from Gómez-García et al. (25)’s binary classification: “high” and “low” ratios.

See Table 7 proposed baseline inspector ratios for ILO regions for details.

**Table 7:** Proposed baseline inspector ratios for ILO regions.

Regions	Poor capacity (≤25% of Median)	Average capacity (~ Median)	Good capacity (≥25% of Median)	Preliminary OSH inspector ratios per 10,000 employed persons
Global (85 ILO regions)	0.435	0.580	(0.58 of 25% + 0.58) to 1.50 = (0.725 to 1.50)	0.50 to 1.50 (due to vast disparity between group 1 and groups 3 and 4, global benchmarking may not sound feasible).
Group 1	0.652	0.870	(0.87 of 25% + 0.87) to 1.50 = (1.08–1.50)	0.87 to 1.50
Group 2	0.330	0.440	(0.44 of 25% + 0.44) to 1.50 = (0.55 to 1.5)	0.44 to 1.50
Groups 3and4	0.371	0.495	(0.495 of 25% + 0.495) to 1.50 = (0.61 to 1.50)	0.49 to 1.50
Malaysia	0.561	0.748	(0.748 of 25% + 0.748) to 1.50 = (0.93 to 1.50)	0.75 to 1.50 (or, group 2)
Saskatchewan	0.853	1.138	1.0 to 1.30 (Stable Value)	1.0 to 1.50 (or group 1)

Poor capacity of the inspector ratio means the regional inspector ratio that is 25% less than the median value in the group. Average capacity of the inspector ratio means closer to or equal to the median value in the group. Good capacity of the inspector ratio means 25% or more than the median value in the regions or group. In short, this study proposed: poor capacity ≤0.25 median, average capacity ~ median, and good capacity ≥25% of the median value of inspector trends in the group or peer level.

Thus, Table 7 recommended the evidence-based or current trends in inspector ratios for each region group studied in this research.

In Table 7, *poor capacity* of the inspector ratio means a regional inspector ratio that is 25% less than the median value in the group. Average capacity of the inspector ratio means closer to or equal to the median value in the group. *Good capacity* of the inspector ratio means 25% or more than the median value in the regions or group. In short, this research proposed: poor capacity≤0.25 median, average capacity ~ median, and good capacity ≥25% of the median value of inspector trends in the group or peer level.

This study’s approach to rank inspector ratios as above, equivalent to, or below 25% of the median value was not new, but also consistent with the literature in public health research, such as (66) ranked Australian hospitals—high adherence (≥ 25% above composite mean value) and low adherence (≥ 25% below composite mean value). With that, this study reminded readers that “practical considerations and intellectual reasoning” (22, p. 1) might have a greater impact on the effectiveness of the inspector ratios or policies than mere statistics.

### 3.4 Limitations and further research

Due to the availability of secondary research datasets, the study relied upon sample sizes to compute the inferential analysis. For example, geographical groups 3 and 4 collectively had only 12 data points from 12 geographical regions. Malaysia and Saskatchewan each had only 8 valid data points for the period of 8 years. Though the research datasets used in this study were secondary longitudinal datasets, this study had limited control over those datasets to avoid bias in the inferential analysis and descriptive statistics for trend analysis. This research also did not perform pre- or post-power analysis in the regression study.

This study did not remove all the outliers from the original data while performing the final regressions, especially regarding groups 1, 2, 3, and 4, but made decisions to include or exclude certain influential data based on sensitivity analysis with Cook’s distance. Future studies may investigate the strength of the associations between the research variables by excluding all outliers and extreme values in the group-level research data. This study log-transformed the reduced datasets for groups 1 and 2 to perform further regression analysis after the sensitivity analysis. Future research can log-transform the original datasets to conduct further multivariate regression analysis. Additionally, there are other functional forms, such as square root, cube root, and exponential transformations for skewed research data; however, this research only performed log-transformation of the dependent variables. While performing GLM regression with bootstrap-2000, this study selected simple bootstrapping at 95% CIs, but future research may explore other bootstrapping samples and methods. This study extracted annual aggregated statistics from ILO’s online webpages. Future studies can be conducted by extracting quarterly or monthly regional data from ILO’s webpages. Researchers can perform time-series or lag structure studies and explore how the associations between the research variables evolve over time. Although this study had eight different years of annual data associated with Malaysia and Saskatchewan, it did not include a lag structure study. This study had only one predictor variable and did not encounter issues with multicollinearity; however, future studies with multiple predictors or confounders, along with inspector ratios, may investigate multicollinearity in their regression models. This was a macro-level, ecological, or regional study, but future research may consider micro-level, industry-level, workplace-level, or team- or worker-level factors and their contributions, along with inspector ratios, to create and maintain regional sustainability.

Additionally, there were contradictory results of *R*^2^ values of the inspector ratio trendlines related to datasets generated by Microsoft Excel and IBM SPSS 30.0 in this study. For example, Figure 3 revealed higher *R*^2^ values (logarithmic regression *R*^2^ = 0.9536) generated by Microsoft Excel with the inspector ratio trending up to 3.5. However, in this research, *R*^2^ values computed via IBM SPSS 30.0 for inferential analysis were significantly lower. This study cautions readers not to base their OSH risk-informed decisions solely on *R*^2^ value(s) computed by Microsoft Excel because SPSS 30.0 is considered more credible regression analytical software than Microsoft Excel.

Any OSH risk management policies or policies related to OSH inspector staffing ratios should be judged or evaluated based on the effectiveness of such policies instead of mere intentions or the spirit of the policies. Therefore, future studies by OSH researchers and practitioners require them to evaluate the effectiveness of the proposed baseline inspector ratios. Further research requires comparing the inspector ratios suggested in this study with the lived experiences or expert opinions of inspectors, regional OSH division directors, and OSH recruiting managers, as well as evaluating the emergency response capacity of the regions. Regional practitioners may need to monitor the impact of the baseline inspector ratios on the performance of regional decent workplace safety and health after implementation. In this age of artificial intelligence (AI), OSH researchers may also explore machine learning (67–69) to predict and simulate the inspector ratios, as in Qiu et al. (42) on nurse staffing.

In this era of Industry 4.0 and AI, especially in developed economies and industries with resources, human workforces are beginning to share workplaces with humanoid AI robots and smart equipment. This new workplace environment has introduced new challenges for regional OSH policymakers and OSH inspectors to create decent workplace OSH environments. Therefore, future studies to improve the inspector ratios may need to consider not only employed human workforces but also the potential populations of humanoid AI robots or smart equipment as workforces, which may be capable of performing certain tasks independently or with minimal supervision in the workplace.

Issues of OSH performance are complex and multidimensional. Today’s OSH stakeholders’ aspiration for decent workplace safety and health and social justice may not be attainable without addressing or eliminating the root causes of the global crisis called child labor. There are about 54 million child laborers active in high-risk industries out of a total of 138 million child laborers (70). This study is also limited to the inspectors appointed by the Ministry of Human Resources under the Factory and Machinery Inspector Scheme of Service and excludes all other public officers serving as *secondment officers* in Malaysia.

Additionally, current literature in “safety theories and methods is still less integrated, multicultural, and global” (71, p. 2). Therefore, future studies of confounders, such as regional OSH culture or maturity, a region’s policy or attitude toward the UN’s sustainability development goals—especially SDG 3: Good Health & Well-Being and SDG 8: Decent Work and Economic Development—OSH policy styles (prescriptive, performance-based, and system-based), industrialization, economic development, competency of OSH inspectors, diversity among OSH inspectors, and the OSH purchasing power of the industries and workplaces in the regions may also reveal significant knowledge about the effectiveness and efficiency of the inspector ratios on regions’ sustainable OSH performance.

### 3.5 Conclusion

This cross-regional study evaluated the current inspector ratio trends across the regions based on the ILO’s expectations regarding inspector ratios. This study also validated or addressed gaps regarding the effectiveness of inspectors, regional or universal inspector ratios, and trends. Furthermore, this study observed the downward trend in Saskatchewan’s workplace injuries while inspector ratios remained stable for multiple years, which also validates the similar findings of (25)—“the number of accidents for every 10,000 workers has decreased while the number of inspectors has remained stable” (25, p. 1).

The study did not observe a strong statistical relationship between the predictor variable and dependent variable(s) in the regression analysis across groups 1, 2, 3, and 4 and Saskatchewan, contrary to the findings of (8) that used statistics from Singapore for their multivariate regression analysis. The findings of this study also validated the findings of (25), which reported, “contrary to the expectations, the results indicate that, in general, the number of inspectors is not associated with the number of accidents” (25, p. 1), and (27), which alluded to the idea that workplace specifics or regional context might impact OSH performance outcomes and inspector ratios.

This study also concluded that universal benchmarking of inspector ratios might not be practical or feasible due to various factors, such as regional or global socioeconomic variables, domestic and international migration of the working population, informal working populations, transparency in reporting OSH statistics, regional politics, and equal application of laws without prejudice while enforcing OSH policies.

Thus, despite the OSH risk management policy styles enforced within the same geographical region by regional inspectors, the OSH performances at the workplace, industry, and regional levels may vary. Industry-level or workforce-level factors or confounders, such as OSH leadership styles (72–74), job satisfaction (75), psychosocial safety climate (76, 77), safety behavior (77, 78), risk perception (79), and job demands or job characteristics (80), may influence OSH performance.

For perspective, the industry premium rates established by Saskatchewan’s Workers’ Compensation Board (WCB) for 2026, based on the regional industries’ OSH performance in 2025, varied even among high-risk industries, such as residential construction (B12), industrial construction (B13), oilwell servicing (D41), and service rigs and water well drilling (D51), which experienced premium rates of 1.62, 2.46, 1.55, and 1.85, respectively (81). In other words, in 2026, these industries would be responsible for the WCB-Saskatchewan premiums of $1,620 (residential construction), $2,460 (industrial construction), $1,550 (oilwell servicing), and $1,850 (service rigs and water well drilling) per $100,000 (CAD) payroll due to their decent workplace performances.

The UN calls upon regional governments to “accelerate comprehensive strategies” (70, p. 24) to address SDG 8.8.1. Since its inception, the ILO has played a significant role in promoting decent workplace safety and healthy environments for all. Today’s regional stakeholders have also taken initiatives to comply with the ILO’s expectations regarding inspector ratios and regional OSH frameworks. However, the question remains whether those initiatives are sufficient to ensure sustainable OSH environments and social justice for all stakeholders.

This study encourages the ILO regions, especially those that have under-resourced OSH policy enforcement agencies, to practice more OSH risk-informed decisions while allocating or curtailing resources and financial assistance for the regional OSH regulators and inspectors. This study provides the ILO regions, including Malaysia and Saskatchewan, with the options to cautiously implement the baseline inspector ratios as starting points for continuous improvement in inspector staffing or capacity ratios. In other words, these baseline inspector ratios may significantly contribute to the incremental changes, gradual refinements, or optimizations of inspector staffing ratios, thereby enhancing the enforcement and compliance of OSH risk management policies in those regions.

Finally, by systematically investigating the roles or effectiveness of inspectors to create safer and healthier regions, this study highlights the potential contributions of inspectors in today’s workplaces and communities and also renews the academic as well as real-world discussion among OSH regulators, policymakers, and other OSH stakeholders. This study is an addition to the literature related to public health because of its association with SDGs, especially in decent workplace safety and health, OSH risk management policies, inspector effectiveness, efficiency, and inspector ratios to create and sustain OSH performance and social justice. Therefore, as the 26th US president said, “Do what you can, with what you have, where you are,” it may be worth investigating or exploring the baseline inspector ratios pointed out in this research. With perseverance and continuous improvement, today’s public health and OSH stakeholders, including policymakers, may be able to create a more sustainable workplace safety and health environment for all generations of stakeholders.

## Data Availability

The original contributions presented in the study are included in the article/Supplementary material, further inquiries can be directed to the corresponding author.

## Footnotes

1
https://ilostat.ilo.org/topics/safety-and-health-at-work/

2
https://ilostat.ilo.org/blog/safety-in-numbers-what-labour-inspection-data-tells-us/

3
https://ilostat.ilo.org/safety-in-numbers-what-labour-inspection-data-tells-us
