# Supplementary material for: OSH inspector ratio to strengthen decent workplace safety and health: a cross-regional trend analysis research
Source: Front Public Health. 2026 Apr 8;14:1806383. doi: 10.3389/fpubh.2026.1806383 (PMC13099828; doi:10.3389/fpubh.2026.1806383)
Supplement: Supplementary file 1 [file Data_Sheet_1.zip › Supplementary document Group 1 – Descriptive and multivariate analysis.pdf]

Supplementary Material

Group 1 – Descriptive statistics and multivariate regression analysis

IBM SPSS 30.0 software was used to complete descriptive statistics and multivariate regression analysis for income group 1, which originally had International Labour Organization's (ILO) 43 nations or regions. There are six major sections in this supplementary material as listed hereunder:

1. Descriptive statistics with original data associated with the research variables (PP. 2-10),
2. General linear multivariate regression (with re-runs where applicable) (PP. 11-37),
3. General linear multivariate regression with bootstrap-2000 with Confidence Intervals of 95% (PP. 38-44),
4. Descriptive statistics with log-transformation of dependent variables (PP. 45-53),
5. Log-general linear multivariate regression with bootstrap-2000 with Confidence Intervals of 95% (PP. 54-60), and
6. Log-general linear multivariate regression analysis (PP. 61-67).

Note:

- Inspector ratio or inspector_ratio, or inspector_rate = Independent Variable (IV),
- Falta injury rate or fatal_rate = Dependent Variable (DV1), and
- Nonfatal injury rate or nonfatal_rate = Dependent Variable (DV2).

Descriptive statistics with original data associated with research variables (PP. 2-8)

Explore

Notes

Output Created		24-FEB-2026 11:09:06
Comments		
Input	Data	E:\Group 1 - Feb 24\Group 1 - Descriptive and regression analysis.sav
	Active Dataset	DataSet0
	Filter	<none>
	Weight	<none>
	Split File	<none>
	N of Rows in Working Data File	43
Missing Value Handling	Definition of Missing	User-defined missing values for dependent variables are treated as missing.
	Cases Used	Statistics are based on cases with no missing values for any dependent variable or factor used.
Syntax		EXAMINE VARIABLES=Inspector_ratio Fatal_rate Nonfatal_rate<br>/PLOT BOXPLOT HISTOGRAM NPPLOT<br>/COMPARE GROUPS<br>/STATISTICS DESCRIPTIVES EXTREME<br>/CINTERVAL 95<br>/MISSING LISTWISE<br>/NOTOTAL.
Resources	Processor Time	00:00:05.48
	Elapsed Time	00:00:01.89

Case Processing Summary

	Cases					
	Valid		Missing		Total	
	N	Percent	N	Percent	N	Percent
Inspector_ratio	43	100.0%	0	0.0%	43	100.0%
Fatal_rate	43	100.0%	0	0.0%	43	100.0%
Nonfatal_rate	43	100.0%	0	0.0%	43	100.0%

Descriptives

			Statistic	Std. Error
Inspector_ratio	Mean		.960930	.1170285
	95% Confidence Interval for Mean	Lower Bound	.724757	
		Upper Bound	1.197103	
	5% Trimmed Mean		.870129	
	Median		.870000	
	Variance		.589	
	Std. Deviation		.7674069	
	Minimum		.0700	
	Maximum		3.8900	
	Range		3.8200	
	Interquartile Range		.6800	
	Skewness		2.073	.361
	Kurtosis		5.382	.709
Fatal_rate	Mean		2.168209	.2575934
	95% Confidence Interval for Mean	Lower Bound	1.648365	
		Upper Bound	2.688054	
	5% Trimmed Mean		2.027186	
	Median		1.624000	
	Variance		2.853	
	Std. Deviation		1.6891527	
	Minimum		.0000	
	Maximum		6.9300	
	Range		6.9300	
	Interquartile Range		1.9300	
	Skewness		1.378	.361
	Kurtosis		1.556	.709
Nonfatal_rate	Mean		1270.536884	223.7488888
	95% Confidence Interval for Mean	Lower Bound	818.993345	
		Upper Bound	1722.080422	
	5% Trimmed Mean		1093.705362	
	Median		808.690000	
	Variance		2152733.305	
	Std. Deviation		1467.2195831	
	Minimum		3.1190	
	Maximum		8670.3600	
	Range		8667.2410	
	Interquartile Range		1502.9400	
	Skewness		3.245	.361
	Kurtosis		14.874	.709

Extreme Values

			Case Number	Value
Inspector_ratio	Highest	1	32	3.8900
		2	22	3.1500
		3	23	2.6500
		4	31	1.9200
		5	21	1.4600
	Lowest	1	42	.0700

		2	6	.1100
		3	40	.1900
		4	17	.2300
		5	3	.2600
Fatal_rate	Highest	1	23	6.9300
		2	14	6.7820
		3	12	6.0000
		4	6	5.1980
		5	33	4.7900
	Lowest	1	32	.0000
		2	5	.1200
		3	27	.3260
		4	3	.5900
		5	38	.7000
Nonfatal_rate	Highest	1	12	8670.3600
		2	7	3141.8000
		3	32	2945.7100
		4	43	2788.0000
		5	22	2551.4020
	Lowest	1	27	3.1190
		2	30	39.9080
		3	40	64.3000
		4	31	97.4700
		5	3	109.0200

Tests of Normality

	Kolmogorov-Smirnov[a]			Shapiro-Wilk		
	Statistic	df	Sig.	Statistic	df	Sig.
Inspector_ratio	.171	43	.003	.804	43	<.001
Fatal_rate	.144	43	.025	.867	43	<.001
Nonfatal_rate	.194	43	<.001	.688	43	<.001

a. Lilliefors Significance Correction

Inspector_ratio

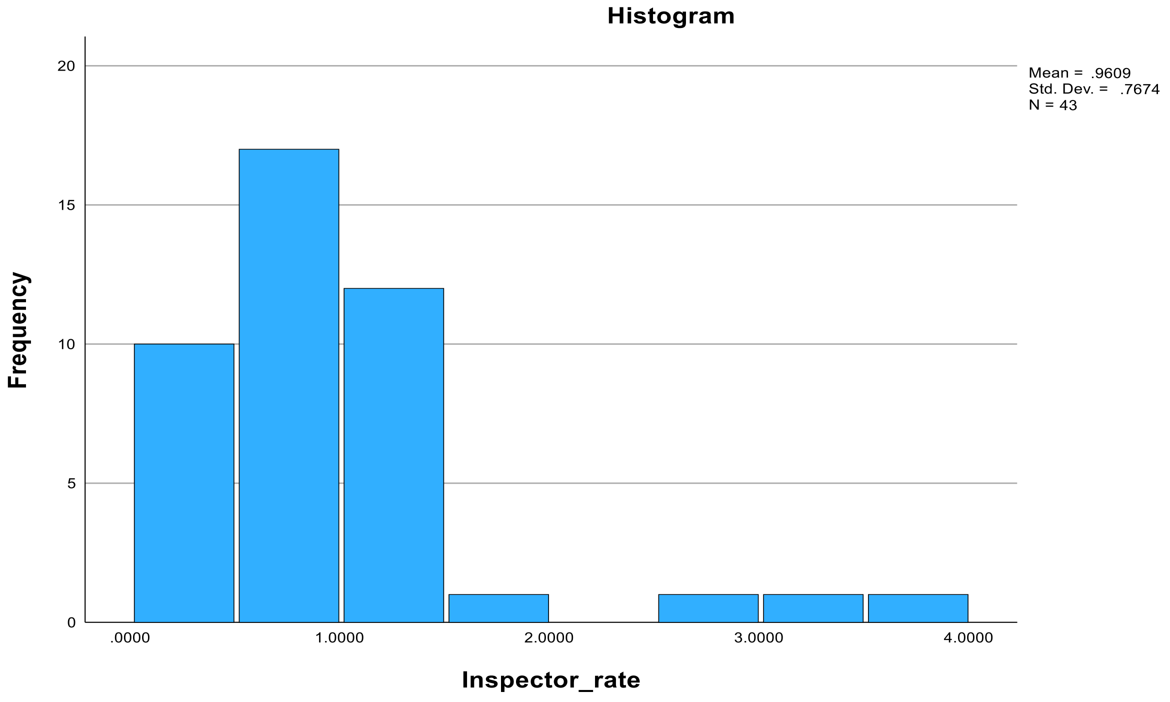

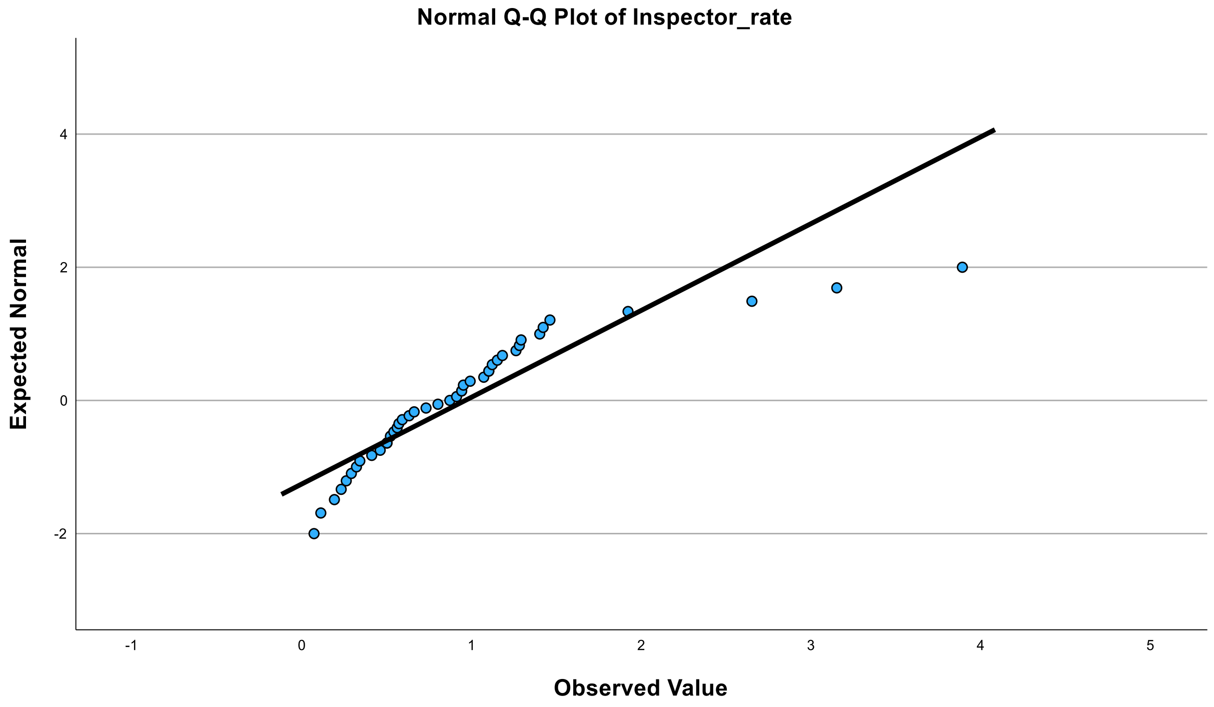

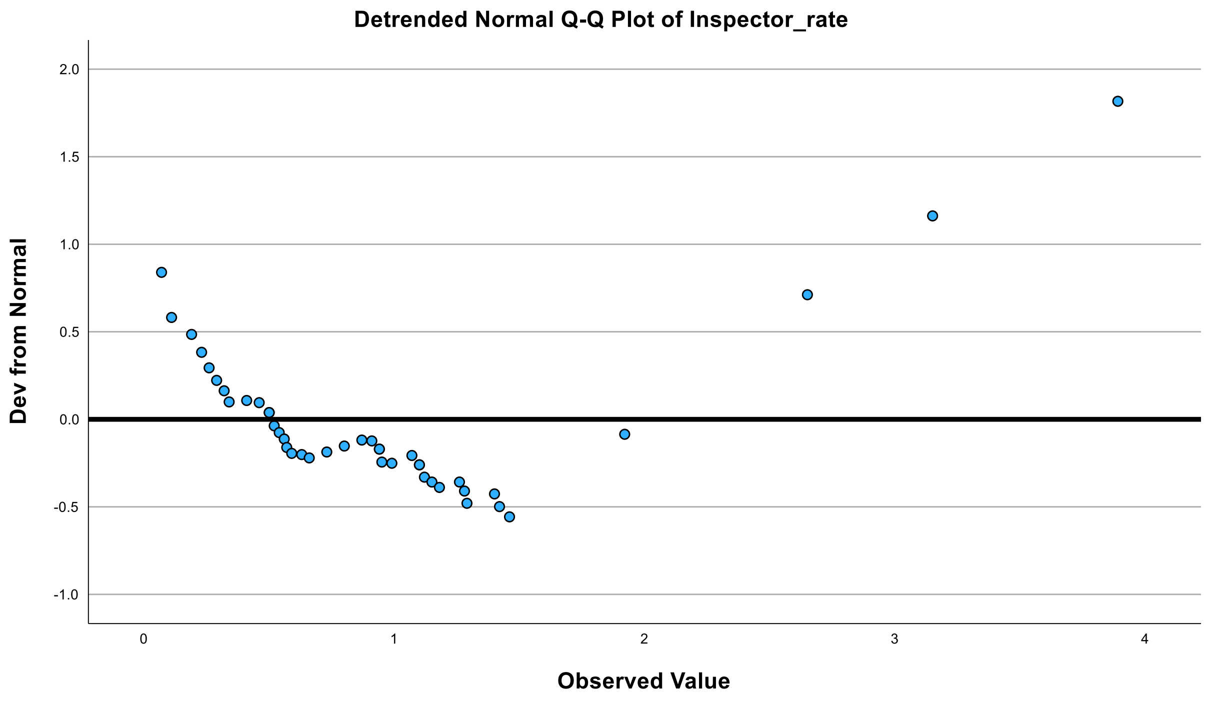
Detrended Normal Q-Q Plot of Inspector_rate
Dev from Normal
2.0
1.5
1.0
0.5
0.0
-0.5
-1.0
0
1
2
3
4
Observed Value

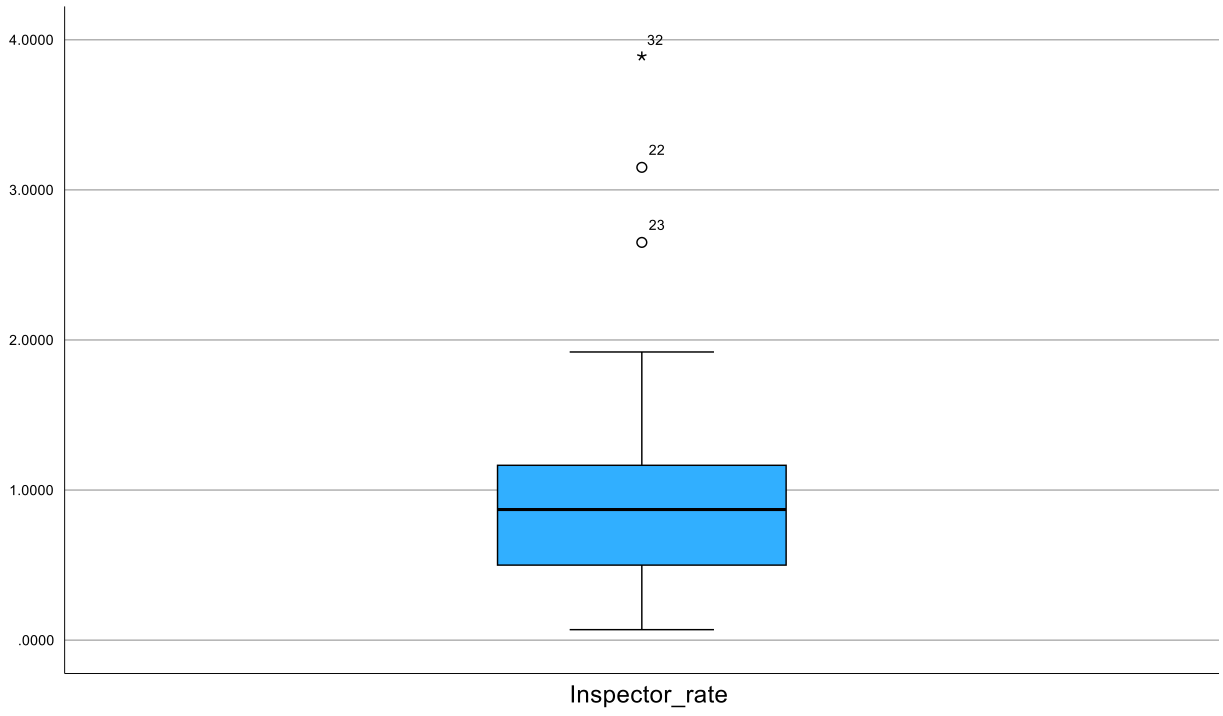
4.0000
3.0000
2.0000
1.0000
.0000
32
22
23
Inspector_rate

Fatal_rate

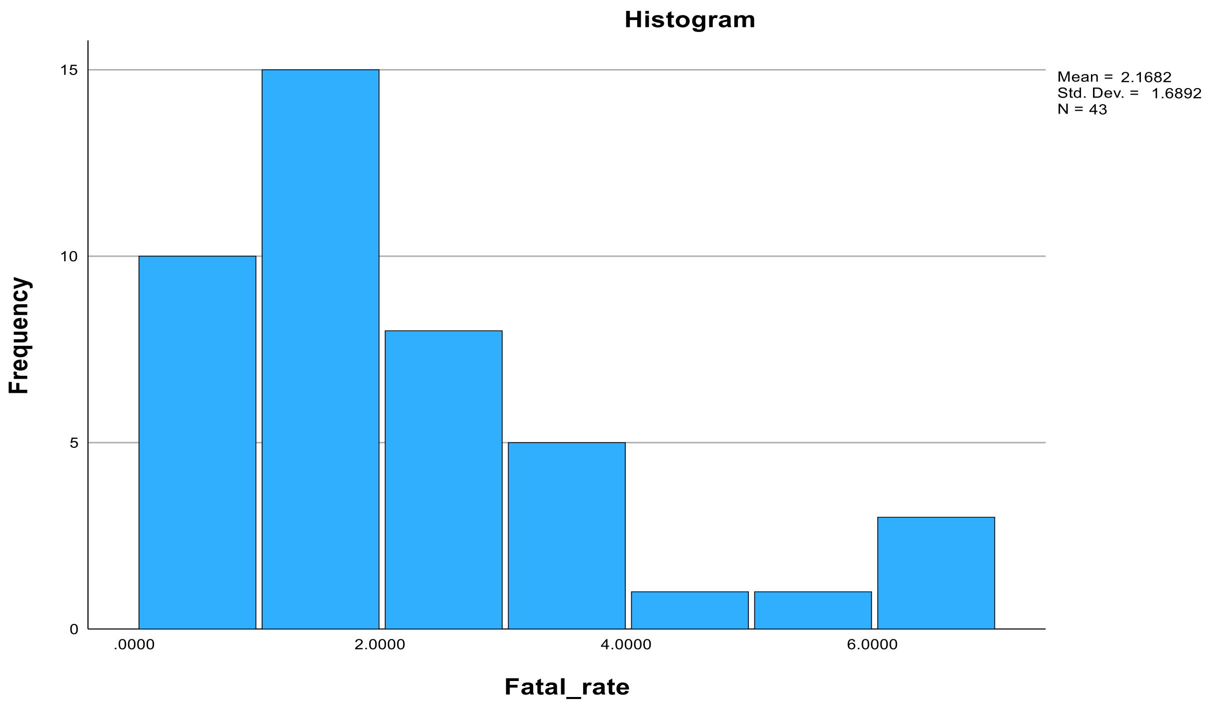
Histogram
Mean = 2.1682
Std. Dev. = 1.6892
N = 43
Frequency
15
10
5
0
.0000
2.0000
4.0000
6.0000
Fatal_rate

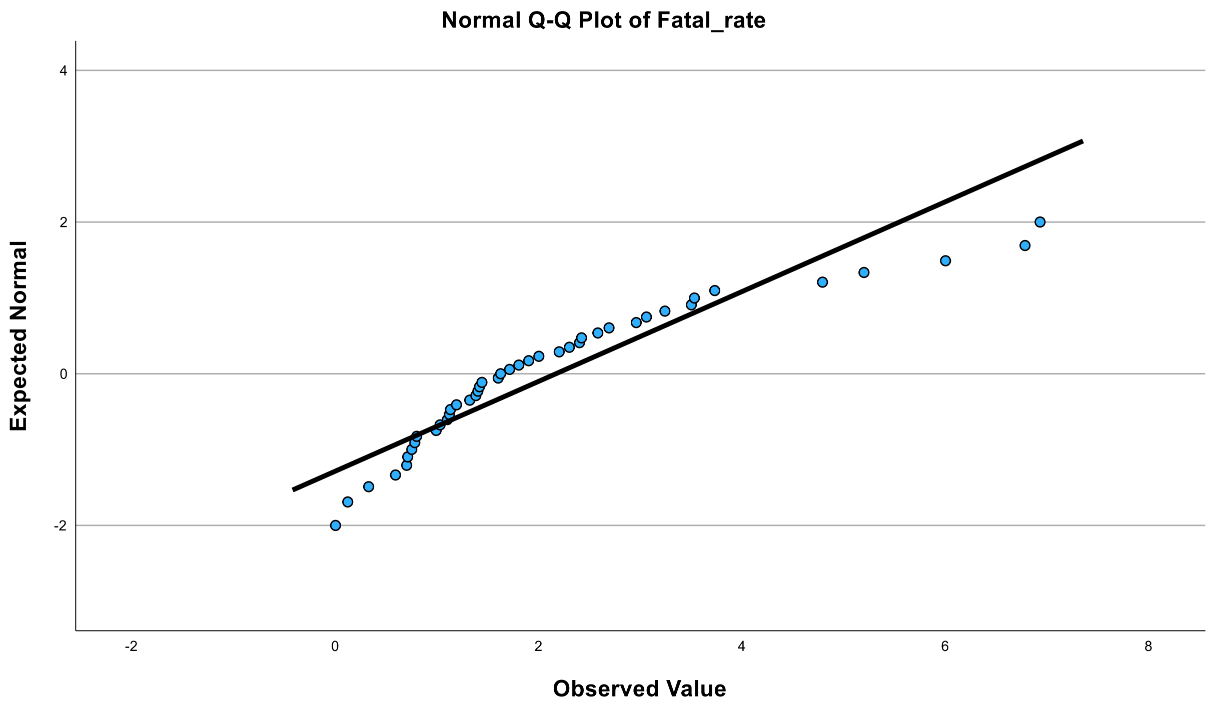
Normal Q-Q Plot of Fatal_rate
Expected Normal
4
2
0
-2
-2
0
2
4
6
8
Observed Value

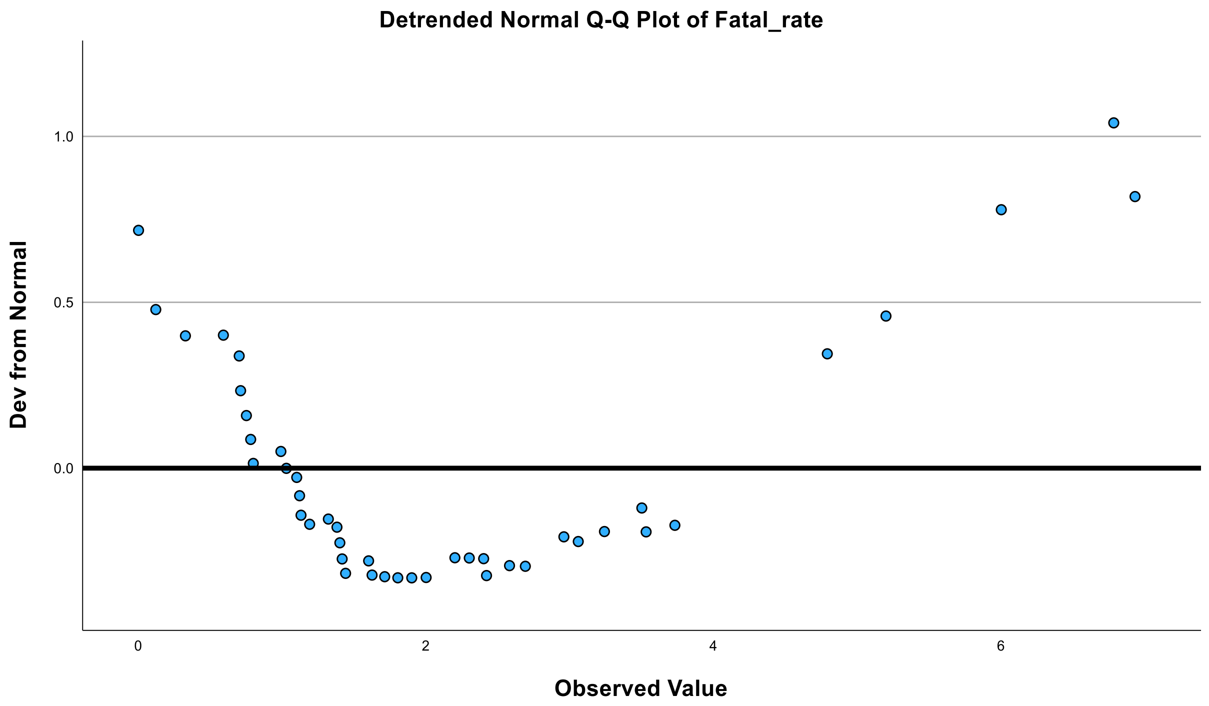
Detrended Normal Q-Q Plot of Fatal_rate
Dev from Normal
1.0
0.5
0.0
0
2
4
6
Observed Value

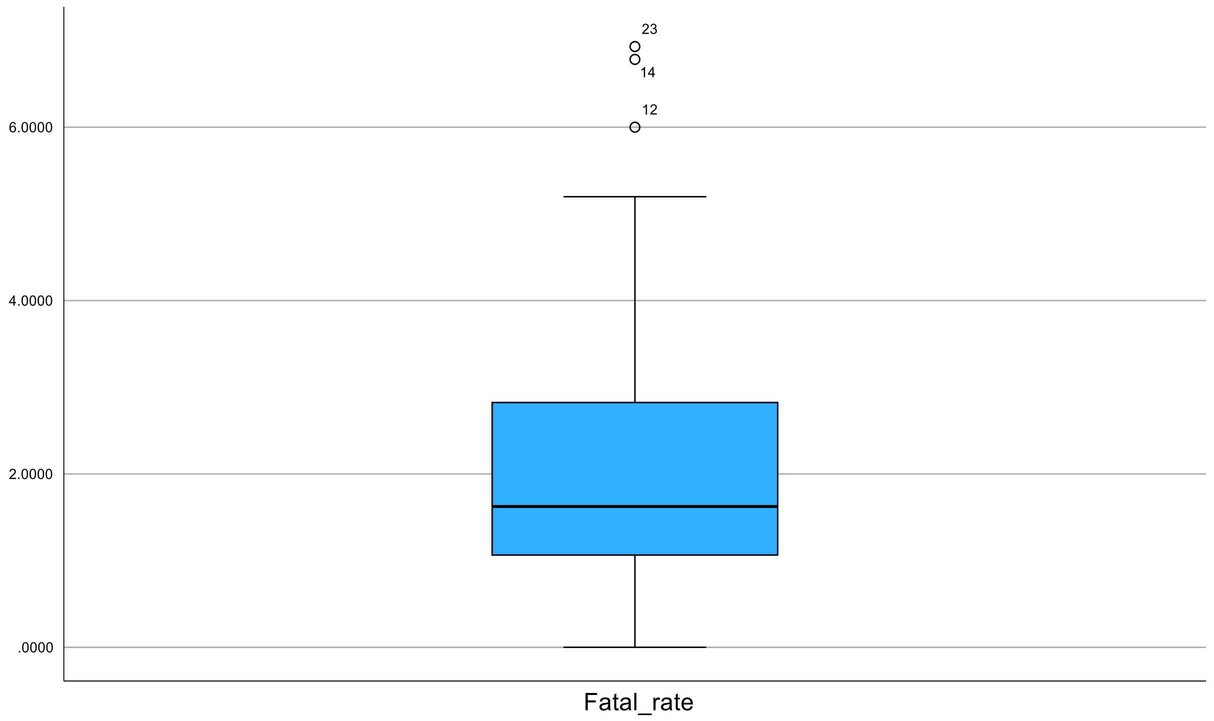
23
14
12
6.0000
4.0000
2.0000
.0000
Fatal_rate

Nonfatal_rate

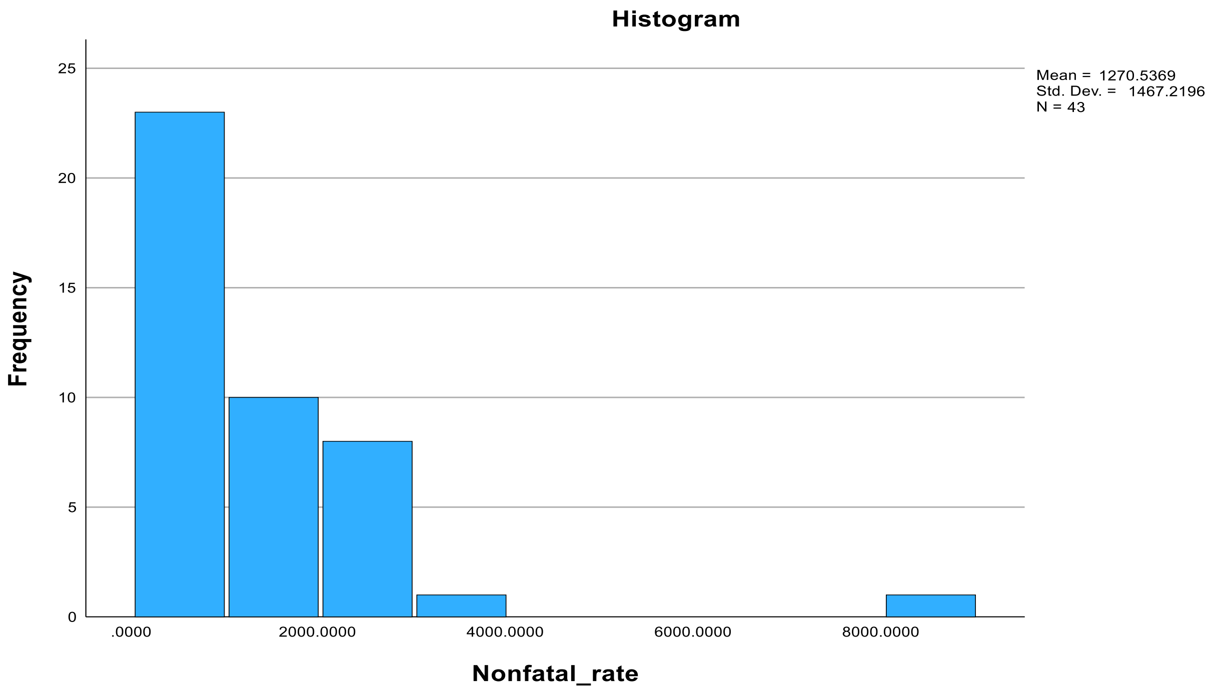

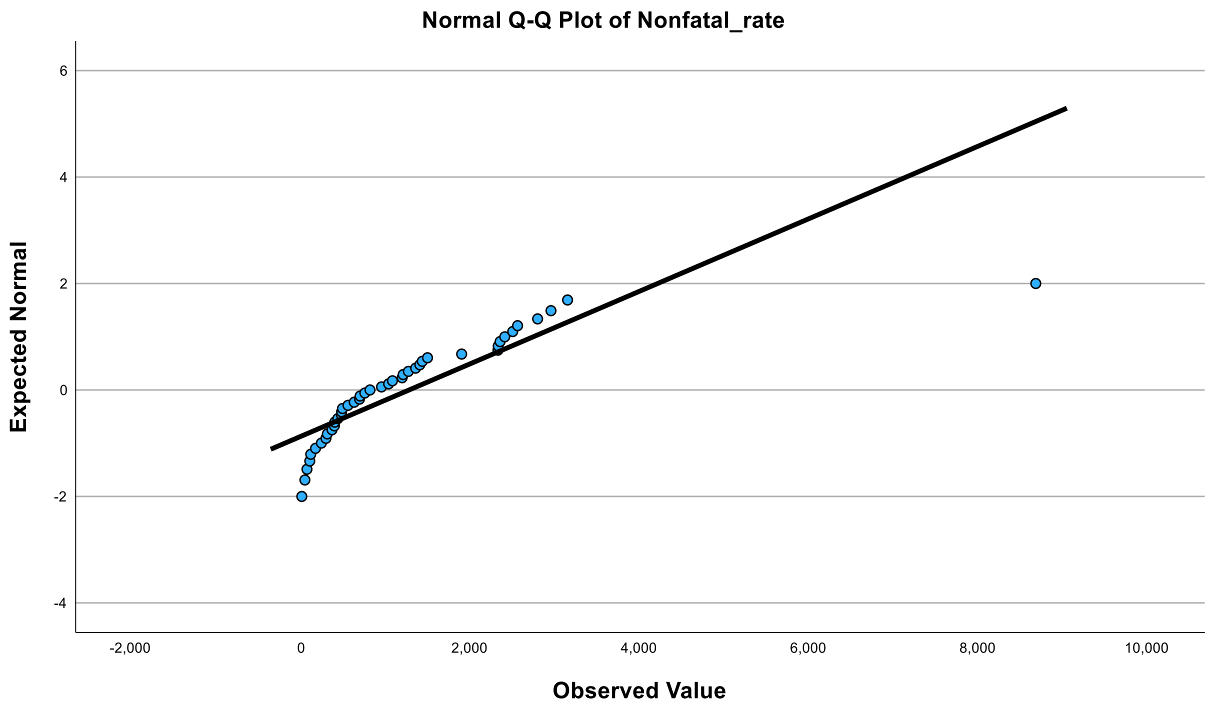

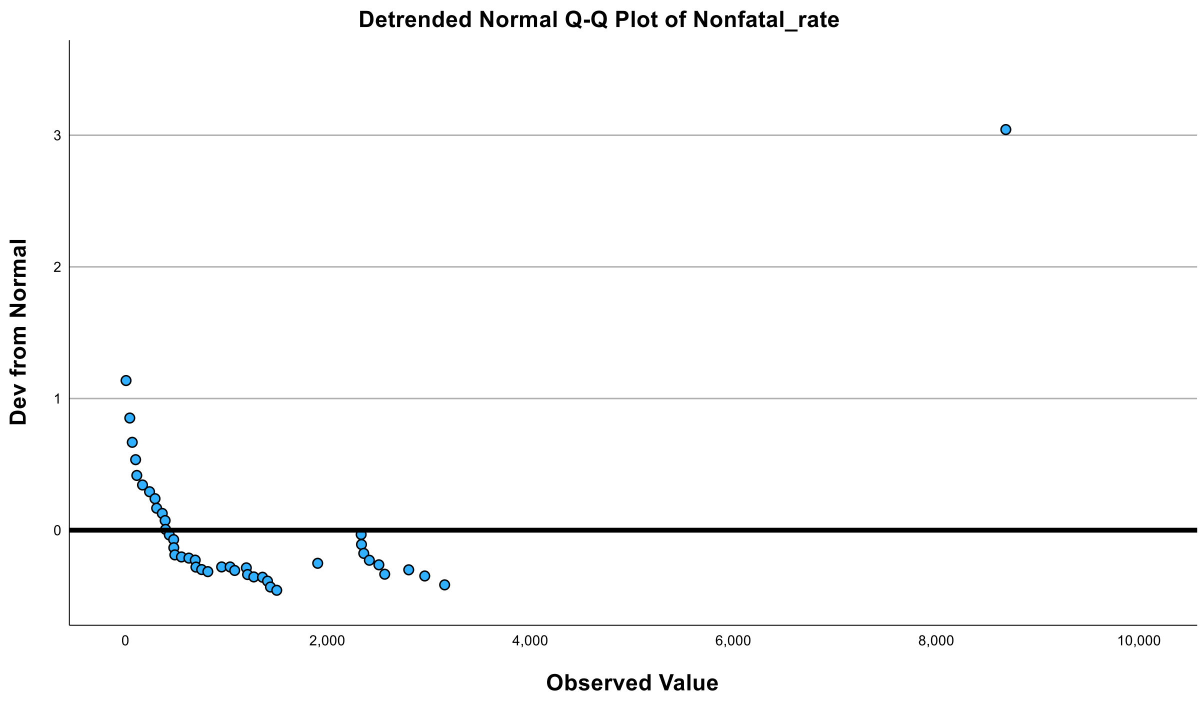
Detrended Normal Q-Q Plot of Nonfatal_rate
Dev from Normal
3
2
1
0
0
2,000
4,000
6,000
8,000
10,000
Observed Value

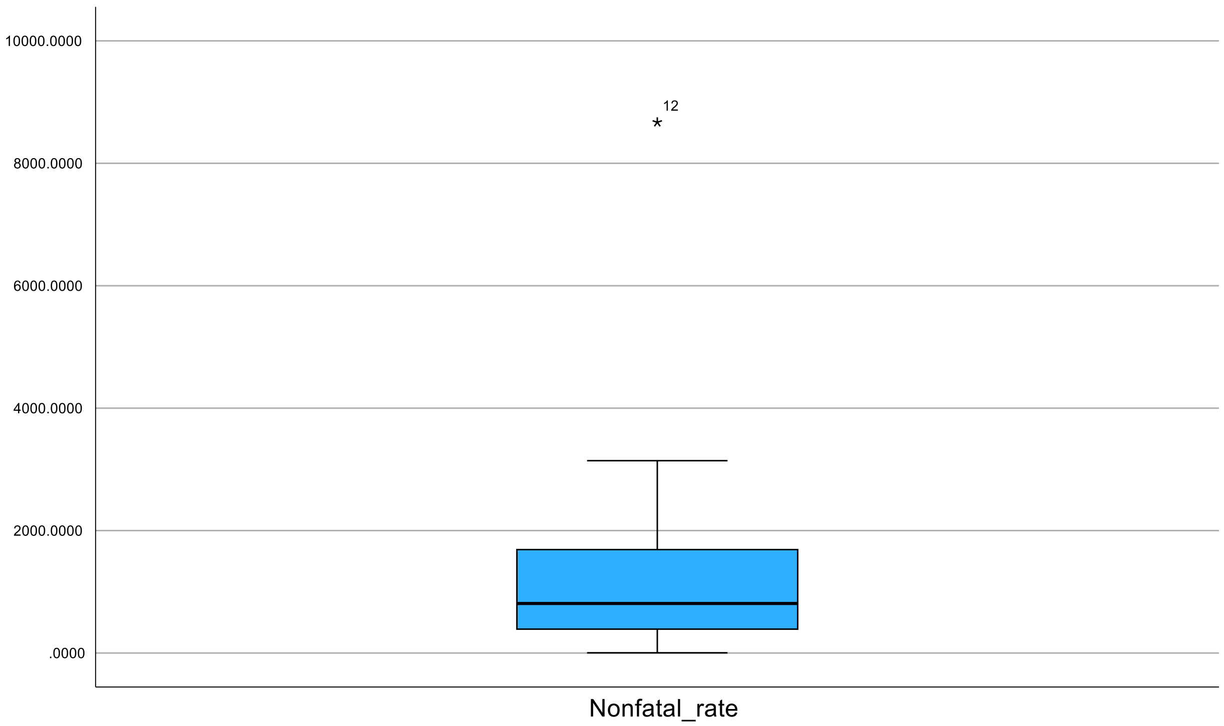
10000.0000
8000.0000
6000.0000
4000.0000
2000.0000
.0000
12
Nonfatal_rate

General linear multivariate regression (with re-runs where applicable) (PP. 11-35)

General Linear Model

Notes

Output Created		24-FEB-2026 11:11:03
Comments		
Input	Data	E:\Group 1 - Feb 24\Group 1 - Descriptive and regression analysis.sav
	Active Dataset	DataSet0
	Filter	<none>
	Weight	<none>
	Split File	<none>
	N of Rows in Working Data File	43
Missing Value Handling	Definition of Missing	User-defined missing values are treated as missing.
	Cases Used	Statistics are based on all cases with valid data for all variables in the model.
Syntax		GLM Fatal_rate Nonfatal_rate WITH Inspector_ratio<br>/METHOD=SSTYPE(3)<br>/INTERCEPT=INCLUDE<br>/SAVE=PRED SEPRED RESID ZRESID COOK LEVER<br>/EMMEANS=TABLES(OVERALL) WITH(Inspector_ratio=MEAN)<br>/PRINT=DESCRIPTIVE ETASQ OPOWER PARAMETER TEST(SSCP) RSSCP HOMOGENEITY LOF<br>/PLOT=SPREADLEVEL RESIDUALS<br>/CRITERIA=ALPHA(.05)<br>/DESIGN=Inspector_ratio.
Resources	Processor Time	00:00:00.73
	Elapsed Time	00:00:00.46
Variables Created or Modified	PRE_1	Predicted Value for Fatal_rate
	PRE_2	Predicted Value for Nonfatal_rate
	SEP_1	Standard Error of Predicted Value for Fatal_rate
	SEP_2	Standard Error of Predicted Value for Nonfatal_rate
	RES_1	Residual for Fatal_rate
	RES_2	Residual for Nonfatal_rate
	ZRE_1	Standardized Residual for Fatal_rate
	ZRE_2	Standardized Residual for Nonfatal_rate
	COO_1	Cook's Distance for Fatal_rate
	COO_2	Cook's Distance for Nonfatal_rate

	LEV_1	Uncentered Leverage Value for Fatal_rate
	LEV_2	Uncentered Leverage Value for Nonfatal_rate

Warnings

The HOMOGENEITY specification in the PRINT subcommand will be ignored because there are no between-subjects factors.
The SPREADLEVEL specification has been found in the PLOT subcommand, but the model includes no factors. The spread versus level plot will not be produced.

Descriptive Statistics

	Mean	Std. Deviation	N
Fatal_rate	2.168209	1.6891527	43
Nonfatal_rate	1270.536884	1467.2195831	43

Bartlett's Test of Sphericity[a]

Likelihood Ratio	.000
Approx. Chi-Square	490.332
df	2
Sig.	<.001

Tests the null hypothesis that the residual covariance matrix is proportional to an identity matrix.[a]

a. Design: Intercept + Inspector_ratio

Multivariate Tests[a]

Effect		Value	F	Hypothesis df	Error df	Sig.
Intercept	Pillai's Trace	.423	14.656[b]	2.000	40.000	<.001
	Wilks' Lambda	.577	14.656[b]	2.000	40.000	<.001
	Hotelling's Trace	.733	14.656[b]	2.000	40.000	<.001
	Roy's Largest Root	.733	14.656[b]	2.000	40.000	<.001
Inspector_ratio	Pillai's Trace	.051	1.072[b]	2.000	40.000	.352
	Wilks' Lambda	.949	1.072[b]	2.000	40.000	.352
	Hotelling's Trace	.054	1.072[b]	2.000	40.000	.352
	Roy's Largest Root	.054	1.072[b]	2.000	40.000	.352

Multivariate Tests[a]

Effect		Partial Eta Squared	Noncent. Parameter	Observed Power[c]
Intercept	Pillai's Trace	.423	29.312	.998
	Wilks' Lambda	.423	29.312	.998
	Hotelling's Trace	.423	29.312	.998
	Roy's Largest Root	.423	29.312	.998
Inspector_ratio	Pillai's Trace	.051	2.143	.225
	Wilks' Lambda	.051	2.143	.225
	Hotelling's Trace	.051	2.143	.225
	Roy's Largest Root	.051	2.143	.225

a. Design: Intercept + Inspector_ratio
b. Exact statistic
c. Computed using alpha = .05

Tests of Between-Subjects Effects

Source	Dependent Variable	Type III Sum of Squares	df	Mean Square	F
Corrected Model	Fatal_rate	.377[a]	1	.377	.129
	Nonfatal_rate	3106834.227[b]	1	3106834.227	1.459
Intercept	Fatal_rate	86.311	1	86.311	29.623
	Nonfatal_rate	14274237.079	1	14274237.079	6.703
Inspector_ratio	Fatal_rate	.377	1	.377	.129
	Nonfatal_rate	3106834.227	1	3106834.227	1.459
Error	Fatal_rate	119.459	41	2.914	
	Nonfatal_rate	87307964.579	41	2129462.551	
Total	Fatal_rate	321.985	43		
	Nonfatal_rate	159828149.641	43		
Corrected Total	Fatal_rate	119.836	42		
	Nonfatal_rate	90414798.806	42		

Tests of Between-Subjects Effects

Source	Dependent Variable	Sig.	Partial Eta Squared	Noncent. Parameter	Observed Power[c]
Corrected Model	Fatal_rate	.721	.003	.129	.064
	Nonfatal_rate	.234	.034	1.459	.218
Intercept	Fatal_rate	<.001	.419	29.623	1.000
	Nonfatal_rate	.013	.141	6.703	.715
Inspector_ratio	Fatal_rate	.721	.003	.129	.064
	Nonfatal_rate	.234	.034	1.459	.218
Error	Fatal_rate				
	Nonfatal_rate				
Total	Fatal_rate				
	Nonfatal_rate				
Corrected Total	Fatal_rate				
	Nonfatal_rate				

a. R Squared = .003 (Adjusted R Squared = -.021)
b. R Squared = .034 (Adjusted R Squared = .011)
c. Computed using alpha = .05

Parameter Estimates

Dependent Variable	Parameter	B	Std. Error	t	Sig.	95% Confidence Interval Lower Bound
Fatal_rate	Intercept	2.287	.420	5.443	<.001	1.438
	Inspector_ratio	-.123	.343	-.360	.721	-.817
Nonfatal_rate	Intercept	929.971	359.193	2.589	.013	204.566
	Inspector_ratio	354.412	293.417	1.208	.234	-238.155

Parameter Estimates

Dependent Variable	Parameter	95% Confidence Interval Upper Bound	Partial Eta Squared	Noncent. Parameter	Observed Power[a]
Fatal_rate	Intercept	3.135	.419	5.443	1.000
	Inspector_ratio	.570	.003	.360	.064
Nonfatal_rate	Intercept	1655.377	.141	2.589	.715
	Inspector_ratio	946.979	.034	1.208	.218

a. Computed using alpha = .05

Between-Subjects SSCP Matrix

			Fatal_rate	Nonfatal_rate
Hypothesis	Intercept	Fatal_rate	86.311	35100.239
		Nonfatal_rate	35100.239	14274237.079
	Inspector_ratio	Fatal_rate	.377	-1081.786
		Nonfatal_rate	-1081.786	3106834.227
Error		Fatal_rate	119.459	37231.546
		Nonfatal_rate	37231.546	87307964.579

Based on Type III Sum of Squares

Residual SSCP Matrix

		Fatal_rate	Nonfatal_rate
Sum-of-Squares and Cross-Products	Fatal_rate	119.459	37231.546
	Nonfatal_rate	37231.546	87307964.579
Covariance	Fatal_rate	2.914	908.086
	Nonfatal_rate	908.086	2129462.551
Correlation	Fatal_rate	1.000	.365
	Nonfatal_rate	.365	1.000

Based on Type III Sum of Squares

Lack of Fit

Multivariate Tests

Dependent Variables		Value	F	Hypothesis df	Error df
Fatal_rate, Nonfatal_rate	Pillai's Trace	1.816	.777	76.000	6.000
	Wilks' Lambda	.001	1.595[a]	76.000	4.000
	Hotelling's Trace	178.670	2.351	76.000	2.000
	Roy's Largest Root	174.074	13.743[b]	38.000	3.000
Fatal_rate	Pillai's Trace	.835	.400[a]	38.000	3.000
	Wilks' Lambda	.165	.400[a]	38.000	3.000
	Hotelling's Trace	5.061	.400[a]	38.000	3.000
	Roy's Largest Root	5.061	.400[a]	38.000	3.000
Nonfatal_rate	Pillai's Trace	.993	11.128[a]	38.000	3.000
	Wilks' Lambda	.007	11.128[a]	38.000	3.000
	Hotelling's Trace	140.957	11.128[a]	38.000	3.000
	Roy's Largest Root	140.957	11.128[a]	38.000	3.000

Multivariate Tests

Dependent Variables		Sig.	Partial Eta Squared	Noncent. Parameter
Fatal_rate, Nonfatal_rate	Pillai's Trace	.726	.908	59.072
	Wilks' Lambda	.355	.968	121.201
	Hotelling's Trace	.345	.989	178.670
	Roy's Largest Root	.026	.994	522.222
Fatal_rate	Pillai's Trace	.926	.835	15.182
	Wilks' Lambda	.926	.835	15.182
	Hotelling's Trace	.926	.835	15.182
	Roy's Largest Root	.926	.835	15.182
Nonfatal_rate	Pillai's Trace	.035	.993	422.872
	Wilks' Lambda	.035	.993	422.872
	Hotelling's Trace	.035	.993	422.872
	Roy's Largest Root	.035	.993	422.872

Multivariate Tests

Dependent Variables		Observed Power[c]
Fatal_rate, Nonfatal_rate	Pillai's Trace	.176
	Wilks' Lambda	.233
	Hotelling's Trace	.158
	Roy's Largest Root	.836
Fatal_rate	Pillai's Trace	.079
	Wilks' Lambda	.079
	Hotelling's Trace	.079
	Roy's Largest Root	.079
Nonfatal_rate	Pillai's Trace	.760
	Wilks' Lambda	.760
	Hotelling's Trace	.760
	Roy's Largest Root	.760

a. Exact statistic
b. The statistic is an upper bound on F that yields a lower bound on the significance level.
c. Computed using alpha = .05

Univariate Tests

Dependent Variable	Source	Sum of Squares	df	Mean Square	F	Sig.
Fatal_rate	Lack of Fit	99.749	38	2.625	.400	.926
	Pure Error	19.711	3	6.570		
Nonfatal_rate	Lack of Fit	86692935.289	38	2281393.034	11.128	.035
	Pure Error	615029.290	3	205009.763		

Univariate Tests

Dependent Variable	Source	Partial Eta Squared	Noncent. Parameter	Observed Power[a]
Fatal_rate	Lack of Fit	.835	15.182	.079
	Pure Error			
Nonfatal_rate	Lack of Fit	.993	422.872	.760
	Pure Error			

a. Computed using alpha = .05

SSCP Matrix

		Fatal_rate	Nonfatal_rate
Lack of Fit	Fatal_rate	99.749	35531.033
	Nonfatal_rate	35531.033	86692935.289
Pure Error	Fatal_rate	19.711	1700.512
	Nonfatal_rate	1700.512	615029.290

Estimated Marginal Means

Grand Mean

Dependent Variable	Mean	Std. Error	95% Confidence Interval Lower Bound	Upper Bound
Fatal_rate	2.168[a]	.260	1.643	2.694
Nonfatal_rate	1270.537[a]	222.536	821.116	1719.958

a. Covariates appearing in the model are evaluated at the following values: Inspector_ratio = .960930.

Observed * Predicted * Std. Residual Plots

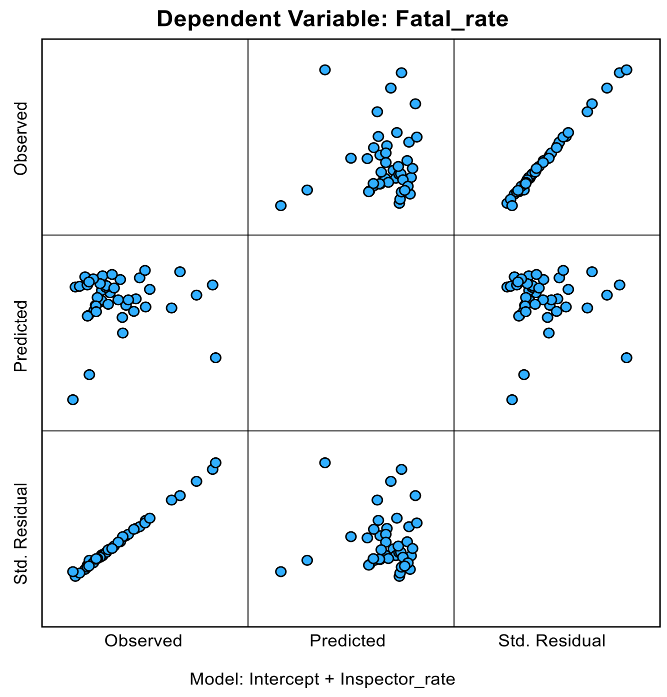

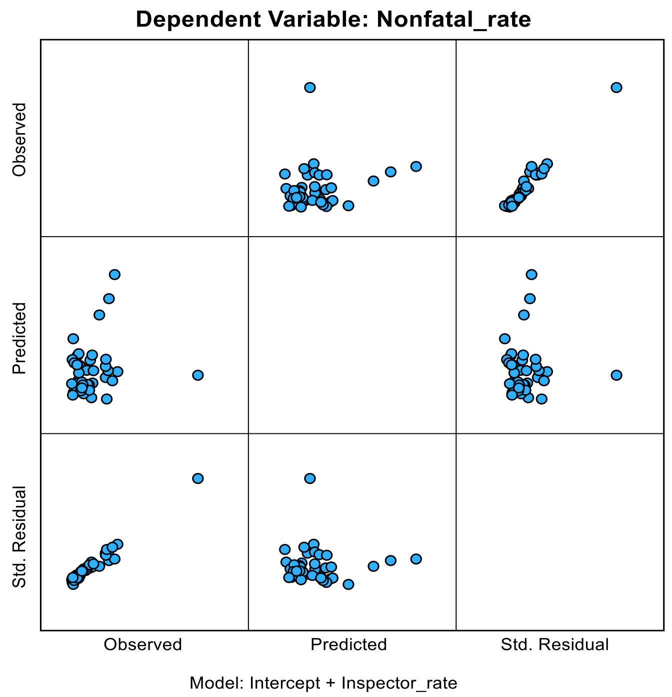

Re-run General Linear Model with reduced data

Notes

Output Created		24-FEB-2026 11:25:31
Comments		
Input	Data	E:\Group 1 - Feb 24\Group 1 - Descriptive and regression analysis -item 2 Rerun and all the rest.sav
	Active Dataset	DataSet2
	Filter	<none>
	Weight	<none>
	Split File	<none>
	N of Rows in Working Data File	40
Missing Value Handling	Definition of Missing	User-defined missing values are treated as missing.
	Cases Used	Statistics are based on all cases with valid data for all variables in the model.
Syntax		GLM Fatal_rate Nonfatal_rate WITH Inspector_ratio /METHOD=SSTYPE(3) /INTERCEPT=INCLUDE /SAVE=PRED SEPRED RESID ZRESID COOK LEVER /EMMEANS=TABLES(OVERALL) WITH(Inspector_ratio=MEAN)

		/PRINT=DESCRIPTIVE ETASQ OPOWER PARAMETER TEST(SSCP) RSSCP HOMOGENEITY LOF /PLOT=SPREADLEVEL RESIDUALS /CRITERIA=ALPHA(.05) /DESIGN=Inspector_ratio.
Resources	Processor Time	00:00:03.97
	Elapsed Time	00:00:00.82
Variables Created or Modified	PRE_1	Predicted Value for Fatal_rate
	PRE_2	Predicted Value for Nonfatal_rate
	SEP_1	Standard Error of Predicted Value for Fatal_rate
	SEP_2	Standard Error of Predicted Value for Nonfatal_rate
	RES_1	Residual for Fatal_rate
	RES_2	Residual for Nonfatal_rate
	ZRE_1	Standardized Residual for Fatal_rate
	ZRE_2	Standardized Residual for Nonfatal_rate
	COO_1	Cook's Distance for Fatal_rate
	COO_2	Cook's Distance for Nonfatal_rate
	LEV_1	Uncentered Leverage Value for Fatal_rate
	LEV_2	Uncentered Leverage Value for Nonfatal_rate

Warnings

The HOMOGENEITY specification in the PRINT subcommand will be ignored because there are no between-subjects factors.
The SPREADLEVEL specification has been found in the PLOT subcommand, but the model includes no factors. The spread versus level plot will not be produced.

Descriptive Statistics

	Mean	Std. Deviation	N
Fatal_rate	1.988025	1.3385262	40
Nonfatal_rate	1215.202150	1494.3834706	40

Bartlett's Test of Sphericity[a]

Likelihood Ratio	.000
Approx. Chi-Square	478.216
df	2
Sig.	<.001

Tests the null hypothesis that the residual covariance matrix is proportional to an identity matrix.[a]

a. Design: Intercept + Inspector_ratio

Multivariate Tests[a]

Effect		Value	F	Hypothesis df	Error df	Sig.
Intercept	Pillai's Trace	.463	15.950[b]	2.000	37.000	<.001
	Wilks' Lambda	.537	15.950[b]	2.000	37.000	<.001
	Hotelling's Trace	.862	15.950[b]	2.000	37.000	<.001
	Roy's Largest Root	.862	15.950[b]	2.000	37.000	<.001
Inspector_ratio	Pillai's Trace	.035	.662[b]	2.000	37.000	.522
	Wilks' Lambda	.965	.662[b]	2.000	37.000	.522
	Hotelling's Trace	.036	.662[b]	2.000	37.000	.522
	Roy's Largest Root	.036	.662[b]	2.000	37.000	.522

Multivariate Tests[a]

Effect		Partial Eta Squared	Noncent. Parameter	Observed Power[c]
Intercept	Pillai's Trace	.463	31.901	.999
	Wilks' Lambda	.463	31.901	.999
	Hotelling's Trace	.463	31.901	.999
	Roy's Largest Root	.463	31.901	.999
Inspector_ratio	Pillai's Trace	.035	1.325	.153
	Wilks' Lambda	.035	1.325	.153
	Hotelling's Trace	.035	1.325	.153
	Roy's Largest Root	.035	1.325	.153

a. Design: Intercept + Inspector_ratio
b. Exact statistic
c. Computed using alpha = .05

Tests of Between-Subjects Effects

Source	Dependent Variable	Type III Sum of Squares	df	Mean Square	F
Corrected Model	Fatal_rate	.896[a]	1	.896	.493
	Nonfatal_rate	510944.071[b]	1	510944.071	.224
Intercept	Fatal_rate	59.146	1	59.146	32.583
	Nonfatal_rate	13103093.218	1	13103093.218	5.751
Inspector_ratio	Fatal_rate	.896	1	.896	.493
	Nonfatal_rate	510944.071	1	510944.071	.224
Error	Fatal_rate	68.979	38	1.815	
	Nonfatal_rate	86583152.263	38	2278504.007	
Total	Fatal_rate	227.964	40		
	Nonfatal_rate	146162746.949	40		
Corrected Total	Fatal_rate	69.874	39		
	Nonfatal_rate	87094096.334	39		

Tests of Between-Subjects Effects

Source	Dependent Variable	Sig.	Partial Eta Squared	Noncent. Parameter	Observed Power[c]
Corrected Model	Fatal_rate	.487	.013	.493	.105
	Nonfatal_rate	.639	.006	.224	.075
Intercept	Fatal_rate	<.001	.462	32.583	1.000
	Nonfatal_rate	.021	.131	5.751	.647
Inspector_ratio	Fatal_rate	.487	.013	.493	.105

	Nonfatal_rate	.639	.006	.224	.075
Error	Fatal_rate				
	Nonfatal_rate				
Total	Fatal_rate				
	Nonfatal_rate				
Corrected Total	Fatal_rate				
	Nonfatal_rate				

a. R Squared = .013 (Adjusted R Squared = -.013)
b. R Squared = .006 (Adjusted R Squared = -.020)
c. Computed using alpha = .05

Parameter Estimates

Dependent Variable	Parameter	B	Std. Error	t	Sig.	95% Confidence Interval Lower Bound
Fatal_rate	Intercept	2.216	.388	5.708	<.001	1.430
	Inspector_ratio	-.266	.379	-.702	.487	-1.033
Nonfatal_rate	Intercept	1043.017	434.940	2.398	.021	162.527
	Inspector_ratio	200.916	424.279	.474	.639	-657.993

Parameter Estimates

Dependent Variable	Parameter	95% Confidence Interval Upper Bound	Partial Eta Squared	Noncent. Parameter	Observed Power[a]
Fatal_rate	Intercept	3.002	.462	5.708	1.000
	Inspector_ratio	.501	.013	.702	.105
Nonfatal_rate	Intercept	1923.508	.131	2.398	.647
	Inspector_ratio	1059.824	.006	.474	.075

a. Computed using alpha = .05

Between-Subjects SSCP Matrix

			Fatal_rate	Nonfatal_rate
Hypothesis	Intercept	Fatal_rate	59.146	27838.774
		Nonfatal_rate	27838.774	13103093.218
	Inspector_ratio	Fatal_rate	.896	-676.468
		Nonfatal_rate	-676.468	510944.071
Error		Fatal_rate	68.979	37484.461
		Nonfatal_rate	37484.461	86583152.263

Based on Type III Sum of Squares

Residual SSCP Matrix

		Fatal_rate	Nonfatal_rate
Sum-of-Squares and Cross-Products	Fatal_rate	68.979	37484.461
	Nonfatal_rate	37484.461	86583152.263
Covariance	Fatal_rate	1.815	986.433
	Nonfatal_rate	986.433	2278504.007
Correlation	Fatal_rate	1.000	.485
	Nonfatal_rate	.485	1.000

Based on Type III Sum of Squares

Lack of Fit

Multivariate Tests

Dependent Variables		Value	F	Hypothesis df	Error df
Fatal_rate, Nonfatal_rate	Pillai's Trace	1.975	4.403	72.000	4.000
	Wilks' Lambda	.000	2.631[a]	72.000	2.000
	Hotelling's Trace	226.287	.000	72.000	.000
	Roy's Largest Root	175.332	9.741[b]	36.000	2.000
Fatal_rate	Pillai's Trace	.982	3.098[a]	36.000	2.000
	Wilks' Lambda	.018	3.098[a]	36.000	2.000
	Hotelling's Trace	55.761	3.098[a]	36.000	2.000
	Roy's Largest Root	55.761	3.098[a]	36.000	2.000
Nonfatal_rate	Pillai's Trace	.994	9.227[a]	36.000	2.000
	Wilks' Lambda	.006	9.227[a]	36.000	2.000
	Hotelling's Trace	166.088	9.227[a]	36.000	2.000
	Roy's Largest Root	166.088	9.227[a]	36.000	2.000

Multivariate Tests

Dependent Variables		Sig.	Partial Eta Squared	Noncent. Parameter
Fatal_rate, Nonfatal_rate	Pillai's Trace	.078	.988	317.047
	Wilks' Lambda	.315	.990	189.430
	Hotelling's Trace	.	.991	.000
	Roy's Largest Root	.097	.994	350.663
Fatal_rate	Pillai's Trace	.274	.982	111.522
	Wilks' Lambda	.274	.982	111.522
	Hotelling's Trace	.274	.982	111.522
	Roy's Largest Root	.274	.982	111.522
Nonfatal_rate	Pillai's Trace	.102	.994	332.176
	Wilks' Lambda	.102	.994	332.176
	Hotelling's Trace	.102	.994	332.176
	Roy's Largest Root	.102	.994	332.176

Multivariate Tests

Dependent Variables		Observed Power[c]
Fatal_rate, Nonfatal_rate	Pillai's Trace	.565
	Wilks' Lambda	.170
	Hotelling's Trace	.
	Roy's Largest Root	.423
Fatal_rate	Pillai's Trace	.189
	Wilks' Lambda	.189
	Hotelling's Trace	.189
	Roy's Largest Root	.189
Nonfatal_rate	Pillai's Trace	.408
	Wilks' Lambda	.408
	Hotelling's Trace	.408
	Roy's Largest Root	.408

a. Exact statistic
b. The statistic is an upper bound on F that yields a lower bound on the significance level.
c. Computed using alpha = .05

Univariate Tests

Dependent Variable	Source	Sum of Squares	df	Mean Square	F	Sig.
Fatal_rate	Lack of Fit	67.764	36	1.882	3.098	.274
	Pure Error	1.215	2	.608		
Nonfatal_rate	Lack of Fit	86064962.577	36	2390693.405	9.227	.102
	Pure Error	518189.686	2	259094.843		

Univariate Tests

Dependent Variable	Source	Partial Eta Squared	Noncent. Parameter	Observed Power[a]
Fatal_rate	Lack of Fit	.982	111.522	.189
	Pure Error			
Nonfatal_rate	Lack of Fit	.994	332.176	.408
	Pure Error			

a. Computed using alpha = .05

SSCP Matrix

		Fatal_rate	Nonfatal_rate
Lack of Fit	Fatal_rate	67.764	37122.262
	Nonfatal_rate	37122.262	86064962.577
Pure Error	Fatal_rate	1.215	362.199
	Nonfatal_rate	362.199	518189.686

Estimated Marginal Means

Grand Mean

Dependent Variable	Mean	Std. Error	95% Confidence Interval Lower Bound	95% Confidence Interval Upper Bound
Fatal_rate	1.988[a]	.213	1.557	2.419
Nonfatal_rate	1215.202[a]	238.668	732.043	1698.361

a. Covariates appearing in the model are evaluated at the following values: Inspector_ratio = .857000.

Observed * Predicted * Std. Residual Plots

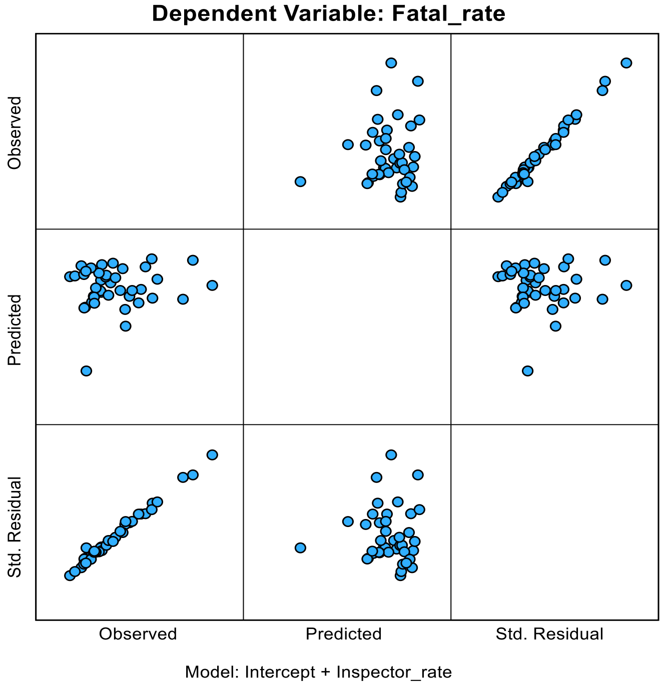

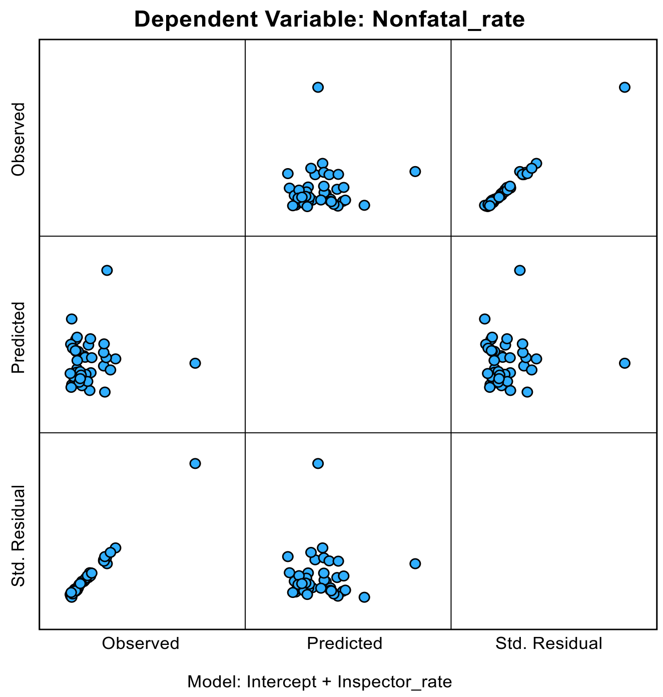

Re-run General Linear Model with reduced data

Notes

Output Created		24-FEB-2026 11:31:59
Comments		
Input	Data	E:\Group 1 - Feb 24\Group 1 - Descriptive and regression analysis -item 2 Rerun and all the rest.sav
	Active Dataset	DataSet2
	Filter	<none>
	Weight	<none>
	Split File	<none>
	N of Rows in Working Data File	37
Missing Value Handling	Definition of Missing	User-defined missing values are treated as missing.
	Cases Used	Statistics are based on all cases with valid data for all variables in the model.
Syntax		GLM Fatal_rate Nonfatal_rate WITH Inspector_ratio /METHOD=SSTYPE(3) /INTERCEPT=INCLUDE /SAVE=PRED SEPRED RESID ZRESID COOK LEVER /PRINT=DESCRIPTIVE ETASQ

		OPOWER PARAMETER TEST(SSCP) RSSCP HOMOGENEITY LOF /PLOT=SPREADLEVEL RESIDUALS /CRITERIA=ALPHA(.05) /DESIGN=Inspector_ratio.
Resources	Processor Time	00:00:00.74
	Elapsed Time	00:00:00.34
Variables Created or Modified	PRE_3	Predicted Value for Fatal_rate
	PRE_4	Predicted Value for Nonfatal_rate
	SEP_3	Standard Error of Predicted Value for Fatal_rate
	SEP_4	Standard Error of Predicted Value for Nonfatal_rate
	RES_3	Residual for Fatal_rate
	RES_4	Residual for Nonfatal_rate
	ZRE_3	Standardized Residual for Fatal_rate
	ZRE_4	Standardized Residual for Nonfatal_rate
	COO_3	Cook's Distance for Fatal_rate
	COO_4	Cook's Distance for Nonfatal_rate
	LEV_3	Uncentered Leverage Value for Fatal_rate
	LEV_4	Uncentered Leverage Value for Nonfatal_rate

Warnings

The HOMOGENEITY specification in the PRINT subcommand will be ignored because there are no between-subjects factors.
The SPREADLEVEL specification has been found in the PLOT subcommand, but the model includes no factors. The spread versus level plot will not be produced.

Descriptive Statistics

	Mean	Std. Deviation	N
Fatal_rate	1.825000	1.0681636	37
Nonfatal_rate	974.019568	874.9524452	37

Bartlett's Test of Sphericity[a]

Likelihood Ratio	.000
Approx. Chi-Square	410.769
df	2
Sig.	<.001

Tests the null hypothesis that the residual covariance matrix is proportional to an identity matrix.[a]

a. Design: Intercept + Inspector_ratio

Multivariate Tests[a]

Effect		Value	F	Hypothesis df	Error df	Sig.
Intercept	Pillai's Trace	.405	11.586[b]	2.000	34.000	<.001
	Wilks' Lambda	.595	11.586[b]	2.000	34.000	<.001
	Hotelling's Trace	.682	11.586[b]	2.000	34.000	<.001
	Roy's Largest Root	.682	11.586[b]	2.000	34.000	<.001
Inspector_ratio	Pillai's Trace	.013	.227[b]	2.000	34.000	.798
	Wilks' Lambda	.987	.227[b]	2.000	34.000	.798
	Hotelling's Trace	.013	.227[b]	2.000	34.000	.798
	Roy's Largest Root	.013	.227[b]	2.000	34.000	.798

Multivariate Tests[a]

Effect		Partial Eta Squared	Noncent. Parameter	Observed Power[c]
Intercept	Pillai's Trace	.405	23.171	.989
	Wilks' Lambda	.405	23.171	.989
	Hotelling's Trace	.405	23.171	.989
	Roy's Largest Root	.405	23.171	.989
Inspector_ratio	Pillai's Trace	.013	.455	.083
	Wilks' Lambda	.013	.455	.083
	Hotelling's Trace	.013	.455	.083
	Roy's Largest Root	.013	.455	.083

a. Design: Intercept + Inspector_ratio
b. Exact statistic
c. Computed using alpha = .05

Tests of Between-Subjects Effects

Source	Dependent Variable	Type III Sum of Squares	df	Mean Square	F	Sig.
Corrected Model	Fatal_rate	.450[a]	1	.450	.387	.538
	Nonfatal_rate	18707.963[b]	1	18707.963	.024	.878
Intercept	Fatal_rate	20.784	1	20.784	17.906	<.001
	Nonfatal_rate	8244042.234	1	8244042.234	10.477	.003
Inspector_ratio	Fatal_rate	.450	1	.450	.387	.538
	Nonfatal_rate	18707.963	1	18707.963	.024	.878
Error	Fatal_rate	40.626	35	1.161		
	Nonfatal_rate	27540796.164	35	786879.890		
Total	Fatal_rate	164.308	37			
	Nonfatal_rate	62661926.494	37			
Corrected Total	Fatal_rate	41.075	36			
	Nonfatal_rate	27559504.127	36			

Tests of Between-Subjects Effects

Source	Dependent Variable	Partial Eta Squared	Noncent. Parameter	Observed Power[c]
Corrected Model	Fatal_rate	.011	.387	.093
	Nonfatal_rate	.001	.024	.053
Intercept	Fatal_rate	.338	17.906	.984
	Nonfatal_rate	.230	10.477	.882
Inspector_ratio	Fatal_rate	.011	.387	.093
	Nonfatal_rate	.001	.024	.053
Error	Fatal_rate			
	Nonfatal_rate			
Total	Fatal_rate			
	Nonfatal_rate			
Corrected Total	Fatal_rate			
	Nonfatal_rate			

a. R Squared = .011 (Adjusted R Squared = -.017)
b. R Squared = .001 (Adjusted R Squared = -.028)
c. Computed using alpha = .05

Parameter Estimates

Dependent Variable	Parameter	B	Std. Error	t	Sig.	95% Confidence Interval Lower Bound
Fatal_rate	Intercept	1.615	.382	4.232	<.001	.840
	Inspector_ratio	.258	.414	.622	.538	-.583
Nonfatal_rate	Intercept	1016.928	314.177	3.237	.003	379.115
	Inspector_ratio	-52.535	340.714	-.154	.878	-744.222

Parameter Estimates

Dependent Variable	Parameter	95% Confidence Interval Upper Bound	Partial Eta Squared	Noncent. Parameter	Observed Power[a]
Fatal_rate	Intercept	2.389	.338	4.232	.984
	Inspector_ratio	1.098	.011	.622	.093
Nonfatal_rate	Intercept	1654.741	.230	3.237	.882
	Inspector_ratio	639.152	.001	.154	.053

a. Computed using alpha = .05

Between-Subjects SSCP Matrix

			Fatal_rate	Nonfatal_rate
Hypothesis	Intercept	Fatal_rate	20.784	13089.801
		Nonfatal_rate	13089.801	8244042.234
	Inspector_ratio	Fatal_rate	.450	-91.705
		Nonfatal_rate	-91.705	18707.963
Error		Fatal_rate	40.626	6700.112
		Nonfatal_rate	6700.112	27540796.164

Based on Type III Sum of Squares

Residual SSCP Matrix

		Fatal_rate	Nonfatal_rate
Sum-of-Squares and Cross-Products	Fatal_rate	40.626	6700.112
	Nonfatal_rate	6700.112	27540796.164
Covariance	Fatal_rate	1.161	191.432
	Nonfatal_rate	191.432	786879.890
Correlation	Fatal_rate	1.000	.200
	Nonfatal_rate	.200	1.000

Based on Type III Sum of Squares

Lack of Fit

Multivariate Tests

Dependent Variables		Value	F	Hypothesis df	Error df
Fatal_rate, Nonfatal_rate	Pillai's Trace	1.954	2.560	66.000	4.000
	Wilks' Lambda	.000	1.376[a]	66.000	2.000
	Hotelling's Trace	97.625	.000	66.000	.000
	Roy's Largest Root	66.897	4.054[b]	33.000	2.000
Fatal_rate	Pillai's Trace	.970	1.965[a]	33.000	2.000
	Wilks' Lambda	.030	1.965[a]	33.000	2.000
	Hotelling's Trace	32.430	1.965[a]	33.000	2.000
	Roy's Largest Root	32.430	1.965[a]	33.000	2.000
Nonfatal_rate	Pillai's Trace	.981	3.160[a]	33.000	2.000
	Wilks' Lambda	.019	3.160[a]	33.000	2.000
	Hotelling's Trace	52.148	3.160[a]	33.000	2.000
	Roy's Largest Root	52.148	3.160[a]	33.000	2.000

Multivariate Tests

Dependent Variables		Sig.	Partial Eta Squared	Noncent. Parameter
Fatal_rate, Nonfatal_rate	Pillai's Trace	.185	.977	168.986
	Wilks' Lambda	.513	.978	90.827
	Hotelling's Trace	.	.980	.000
	Roy's Largest Root	.217	.985	133.794
Fatal_rate	Pillai's Trace	.394	.970	64.860
	Wilks' Lambda	.394	.970	64.860
	Hotelling's Trace	.394	.970	64.860
	Roy's Largest Root	.394	.970	64.860
Nonfatal_rate	Pillai's Trace	.269	.981	104.296
	Wilks' Lambda	.269	.981	104.296
	Hotelling's Trace	.269	.981	104.296
	Roy's Largest Root	.269	.981	104.296

Multivariate Tests

Dependent Variables		Observed Power[c]
Fatal_rate, Nonfatal_rate	Pillai's Trace	.356
	Wilks' Lambda	.115
	Hotelling's Trace	.
	Roy's Largest Root	.228
Fatal_rate	Pillai's Trace	.141
	Wilks' Lambda	.141
	Hotelling's Trace	.141
	Roy's Largest Root	.141
Nonfatal_rate	Pillai's Trace	.192
	Wilks' Lambda	.192
	Hotelling's Trace	.192
	Roy's Largest Root	.192

a. Exact statistic
b. The statistic is an upper bound on F that yields a lower bound on the significance level.
c. Computed using alpha = .05

Univariate Tests

Dependent Variable	Source	Sum of Squares	df	Mean Square	F	Sig.
Fatal_rate	Lack of Fit	39.410	33	1.194	1.965	.394
	Pure Error	1.215	2	.608		
Nonfatal_rate	Lack of Fit	27022606.478	33	818866.863	3.160	.269
	Pure Error	518189.686	2	259094.843		

Univariate Tests

Dependent Variable	Source	Partial Eta Squared	Noncent. Parameter	Observed Power[a]
Fatal_rate	Lack of Fit	.970	64.860	.141
	Pure Error			
Nonfatal_rate	Lack of Fit	.981	104.296	.192
	Pure Error			

a. Computed using alpha = .05

SSCP Matrix

		Fatal_rate	Nonfatal_rate
Lack of Fit	Fatal_rate	39.410	6337.914
	Nonfatal_rate	6337.914	27022606.478
Pure Error	Fatal_rate	1.215	362.199
	Nonfatal_rate	362.199	518189.686

Observed * Predicted * Std. Residual Plots

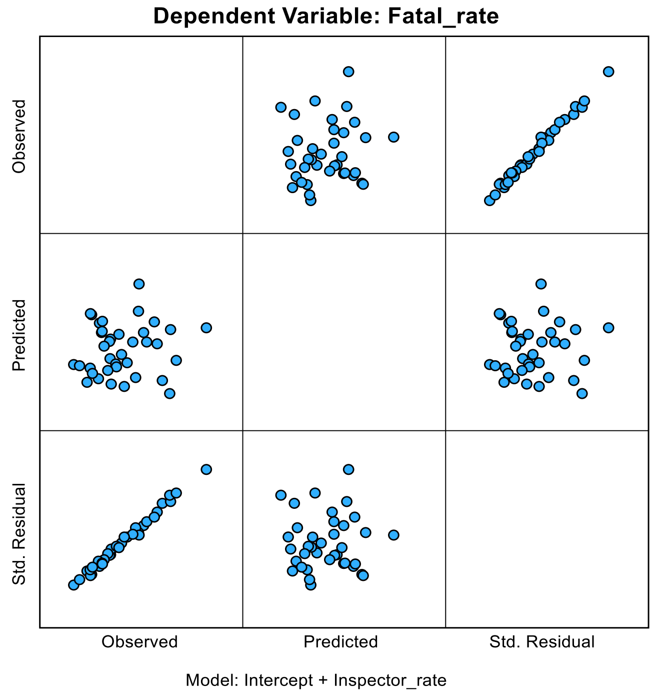

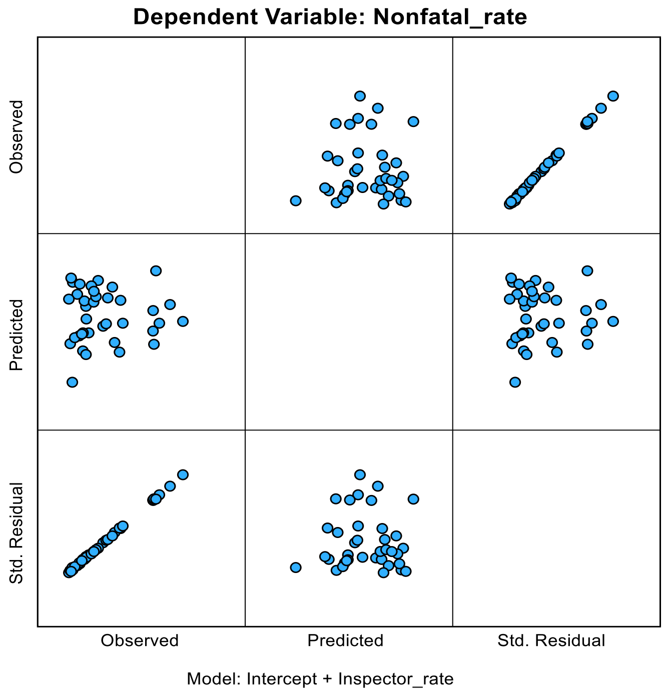

Re-run General Linear Model with reduced data

Notes

Output Created		24-FEB-2026 11:37:50
Comments		
Input	Data	E:\Group 1 - Feb 24\Group 1 - Descriptive and regression analysis -item 2 Rerun and all the rest.sav
	Active Dataset	DataSet2
	Filter	<none>
	Weight	<none>
	Split File	<none>
	N of Rows in Working Data File	34
Missing Value Handling	Definition of Missing	User-defined missing values are treated as missing.
	Cases Used	Statistics are based on all cases with valid data for all variables in the model.
Syntax		GLM Fatal_rate Nonfatal_rate WITH Inspector_ratio<br>/METHOD=SSTYPE(3)<br>/INTERCEPT=INCLUDE<br>/SAVE=PRED SEPRED RESID ZRESID COOK LEVER<br>/EMMEANS=TABLES(OVERALL) WITH(Inspector_ratio=MEAN)<br>/PRINT=DESCRIPTIVE ETASQ OPOWER PARAMETER TEST(SSCP) RSSCP HOMOGENEITY LOF<br>/PLOT=SPREADLEVEL RESIDUALS<br>/CRITERIA=ALPHA(.05)<br>/DESIGN=Inspector_ratio.
Resources	Processor Time	00:00:00.45
	Elapsed Time	00:00:00.31
Variables Created or Modified	PRE_5	Predicted Value for Fatal_rate
	PRE_6	Predicted Value for Nonfatal_rate
	SEP_5	Standard Error of Predicted Value for Fatal_rate
	SEP_6	Standard Error of Predicted Value for Nonfatal_rate
	RES_5	Residual for Fatal_rate
	RES_6	Residual for Nonfatal_rate
	ZRE_5	Standardized Residual for Fatal_rate
	ZRE_6	Standardized Residual for Nonfatal_rate
	COO_5	Cook's Distance for Fatal_rate
	COO_6	Cook's Distance for Nonfatal_rate
	LEV_5	Uncentered Leverage Value for Fatal_rate
	LEV_6	Uncentered Leverage Value for Nonfatal_rate

Warnings

The HOMOGENEITY specification in the PRINT subcommand will be ignored because there are no between-subjects factors.
The SPREADLEVEL specification has been found in the PLOT subcommand, but the model includes no factors. The spread versus level plot will not be produced.

Descriptive Statistics

	Mean	Std. Deviation	N
Fatal_rate	1.671029	.9263506	34
Nonfatal_rate	981.647471	854.6068051	34

Bartlett's Test of Sphericity[a]

Likelihood Ratio	.000
Approx. Chi-Square	381.772
df	2
Sig.	<.001

Tests the null hypothesis that the residual covariance matrix is proportional to an identity matrix.[a]

a. Design: Intercept + Inspector_ratio

Multivariate Tests[a]

Effect		Value	F	Hypothesis df	Error df	Sig.
Intercept	Pillai's Trace	.341	8.013[b]	2.000	31.000	.002
	Wilks' Lambda	.659	8.013[b]	2.000	31.000	.002
	Hotelling's Trace	.517	8.013[b]	2.000	31.000	.002
	Roy's Largest Root	.517	8.013[b]	2.000	31.000	.002
Inspector_ratio	Pillai's Trace	.036	.576[b]	2.000	31.000	.568
	Wilks' Lambda	.964	.576[b]	2.000	31.000	.568
	Hotelling's Trace	.037	.576[b]	2.000	31.000	.568
	Roy's Largest Root	.037	.576[b]	2.000	31.000	.568

Multivariate Tests[a]

Effect		Partial Eta Squared	Noncent. Parameter	Observed Power[c]
Intercept	Pillai's Trace	.341	16.027	.937
	Wilks' Lambda	.341	16.027	.937
	Hotelling's Trace	.341	16.027	.937
	Roy's Largest Root	.341	16.027	.937
Inspector_ratio	Pillai's Trace	.036	1.152	.137
	Wilks' Lambda	.036	1.152	.137
	Hotelling's Trace	.036	1.152	.137
	Roy's Largest Root	.036	1.152	.137

a. Design: Intercept + Inspector_ratio

b. Exact statistic

c. Computed using alpha = .05

Tests of Between-Subjects Effects

Source	Dependent Variable	Type III Sum of Squares	df	Mean Square	F	Sig.
Corrected Model	Fatal_rate	.273[a]	1	.273	.312	.580
	Nonfatal_rate	803249.301[b]	1	803249.301	1.103	.301
Intercept	Fatal_rate	13.754	1	13.754	15.694	<.001
	Nonfatal_rate	2711048.035	1	2711048.035	3.724	.063
Inspector_ratio	Fatal_rate	.273	1	.273	.312	.580
	Nonfatal_rate	803249.301	1	803249.301	1.103	.301
Error	Fatal_rate	28.045	32	.876		
	Nonfatal_rate	23298392.812	32	728074.775		
Total	Fatal_rate	123.258	34			
	Nonfatal_rate	56865121.835	34			
Corrected Total	Fatal_rate	28.318	33			
	Nonfatal_rate	24101642.114	33			

Tests of Between-Subjects Effects

Source	Dependent Variable	Partial Eta Squared	Noncent. Parameter	Observed Power[c]
Corrected Model	Fatal_rate	.010	.312	.084
	Nonfatal_rate	.033	1.103	.175
Intercept	Fatal_rate	.329	15.694	.970
	Nonfatal_rate	.104	3.724	.465
Inspector_ratio	Fatal_rate	.010	.312	.084
	Nonfatal_rate	.033	1.103	.175
Error	Fatal_rate			
	Nonfatal_rate			
Total	Fatal_rate			
	Nonfatal_rate			
Corrected Total	Fatal_rate			
	Nonfatal_rate			

a. R Squared = .010 (Adjusted R Squared = -.021)

b. R Squared = .033 (Adjusted R Squared = .003)

c. Computed using alpha = .05

Parameter Estimates

Dependent Variable	Parameter	B	Std. Error	t	Sig.	95% Confidence Interval Lower Bound
Fatal_rate	Intercept	1.482	.374	3.962	<.001	.720
	Inspector_ratio	.237	.425	.558	.580	-.628
Nonfatal_rate	Intercept	658.088	341.038	1.930	.063	-36.585
	Inspector_ratio	406.693	387.194	1.050	.301	-381.997

Parameter Estimates

Dependent Variable	Parameter	95% Confidence Interval Upper Bound	Partial Eta Squared	Noncent. Parameter	Observed Power[a]
Fatal_rate	Intercept	2.244	.329	3.962	.970
	Inspector_ratio	1.103	.010	.558	.084
Nonfatal_rate	Intercept	1352.760	.104	1.930	.465
	Inspector_ratio	1195.382	.033	1.050	.175

a. Computed using alpha = .05

Between-Subjects SSCP Matrix

			Fatal_rate	Nonfatal_rate
Hypothesis	Intercept	Fatal_rate	13.754	6106.393
		Nonfatal_rate	6106.393	2711048.035
	Inspector_ratio	Fatal_rate	.273	468.569
		Nonfatal_rate	468.569	803249.301
Error		Fatal_rate	28.045	6710.832
		Nonfatal_rate	6710.832	23298392.812

Based on Type III Sum of Squares

Residual SSCP Matrix

		Fatal_rate	Nonfatal_rate
Sum-of-Squares and Cross-Products	Fatal_rate	28.045	6710.832
	Nonfatal_rate	6710.832	23298392.812
Covariance	Fatal_rate	.876	209.714
	Nonfatal_rate	209.714	728074.775
Correlation	Fatal_rate	1.000	.263
	Nonfatal_rate	.263	1.000

Based on Type III Sum of Squares

Lack of Fit

Multivariate Tests

Dependent Variables		Value	F	Hypothesis df	Error df
Fatal_rate, Nonfatal_rate	Pillai's Trace	1.938	2.069	60.000	4.000
	Wilks' Lambda	.001	1.131[a]	60.000	2.000
	Hotelling's Trace	74.191	.000	60.000	.000
	Roy's Largest Root	52.294	3.486[b]	30.000	2.000
Fatal_rate	Pillai's Trace	.957	1.472[a]	30.000	2.000
	Wilks' Lambda	.043	1.472[a]	30.000	2.000
	Hotelling's Trace	22.077	1.472[a]	30.000	2.000
	Roy's Largest Root	22.077	1.472[a]	30.000	2.000
Nonfatal_rate	Pillai's Trace	.978	2.931[a]	30.000	2.000
	Wilks' Lambda	.022	2.931[a]	30.000	2.000
	Hotelling's Trace	43.961	2.931[a]	30.000	2.000
	Roy's Largest Root	43.961	2.931[a]	30.000	2.000

Multivariate Tests

Dependent Variables		Sig.	Partial Eta Squared	Noncent. Parameter
Fatal_rate, Nonfatal_rate	Pillai's Trace	.252	.969	124.128
	Wilks' Lambda	.582	.971	67.865
	Hotelling's Trace	.	.974	.000
	Roy's Largest Root	.247	.981	104.588
Fatal_rate	Pillai's Trace	.485	.957	44.155
	Wilks' Lambda	.485	.957	44.155
	Hotelling's Trace	.485	.957	44.155
	Roy's Largest Root	.485	.957	44.155
Nonfatal_rate	Pillai's Trace	.286	.978	87.922
	Wilks' Lambda	.286	.978	87.922
	Hotelling's Trace	.286	.978	87.922
	Roy's Largest Root	.286	.978	87.922

Multivariate Tests

Dependent Variables		Observed Power[c]
Fatal_rate, Nonfatal_rate	Pillai's Trace	.293
	Wilks' Lambda	.104
	Hotelling's Trace	.
	Roy's Largest Root	.205
Fatal_rate	Pillai's Trace	.119
	Wilks' Lambda	.119
	Hotelling's Trace	.119
	Roy's Largest Root	.119
Nonfatal_rate	Pillai's Trace	.182
	Wilks' Lambda	.182
	Hotelling's Trace	.182
	Roy's Largest Root	.182

a. Exact statistic

b. The statistic is an upper bound on F that yields a lower bound on the significance level.

c. Computed using alpha = .05

Univariate Tests

Dependent Variable	Source	Sum of Squares	df	Mean Square	F	Sig.
Fatal_rate	Lack of Fit	26.830	30	.894	1.472	.485
	Pure Error	1.215	2	.608		
Nonfatal_rate	Lack of Fit	22780203.126	30	759340.104	2.931	.286
	Pure Error	518189.686	2	259094.843		

Univariate Tests

Dependent Variable	Source	Partial Eta Squared	Noncent. Parameter	Observed Power[a]
Fatal_rate	Lack of Fit	.957	44.155	.119
	Pure Error			
Nonfatal_rate	Lack of Fit	.978	87.922	.182

	Pure Error			

a. Computed using alpha = .05

SSCP Matrix

		Fatal_rate	Nonfatal_rate
Lack of Fit	Fatal_rate	26.830	6348.634
	Nonfatal_rate	6348.634	22780203.126
Pure Error	Fatal_rate	1.215	362.199
	Nonfatal_rate	362.199	518189.686

Estimated Marginal Means

Grand Mean

Dependent Variable	Mean	Std. Error	95% Confidence Interval Lower Bound	95% Confidence Interval Upper Bound
Fatal_rate	1.671[a]	.161	1.344	1.998
Nonfatal_rate	981.647[a]	146.335	683.573	1279.722

a. Covariates appearing in the model are evaluated at the following values: Inspector_ratio = .795588.

Observed * Predicted * Std. Residual Plots

Dependent Variable: Fatal_rate

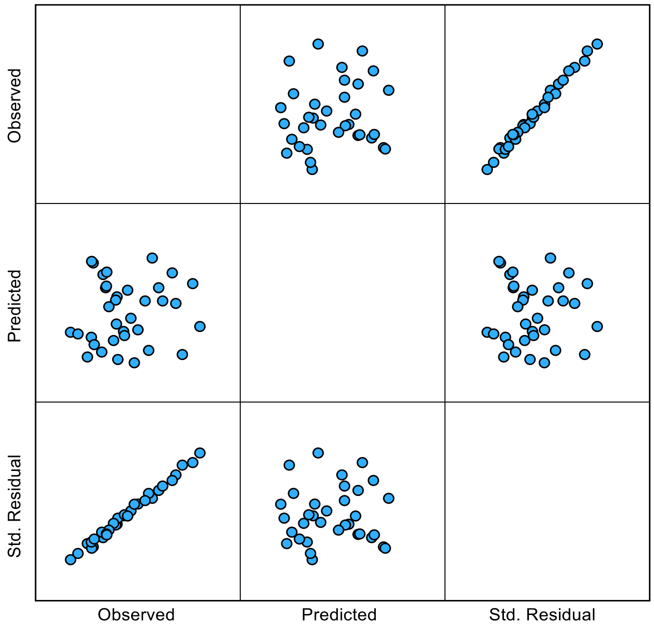

Model: Intercept + Inspector_rate

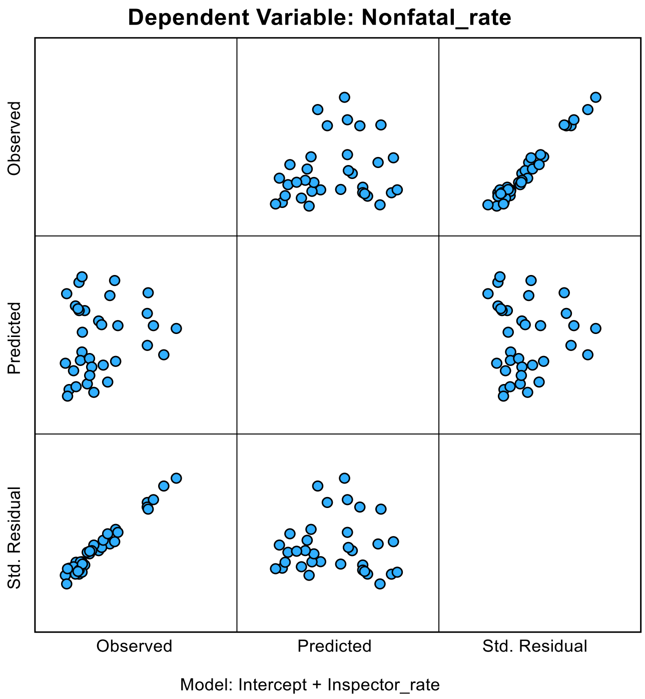

Dependent Variable: Nonfatal_rate
Observed
Predicted
Std. Residual
Observed
Predicted
Std. Residual
Model: Intercept + Inspector_rate

Continued.

General linear multivariate regression with bootstrap-2000 with Confidence Intervals of 95% (PP. 38-44)

Notes

Output Created		24-FEB-2026 11:39:45
Comments		
Input	Data	E:\Group 1 - Feb 24\Group 1 - Descriptive and regression analysis -item 2 Rerun and all the rest.sav
	Active Dataset	DataSet2
	Filter	<none>
	Weight	<none>
	Split File	<none>
	N of Rows in Working Data File	34
Syntax		BOOTSTRAP<br>/SAMPLING METHOD=SIMPLE<br>/VARIABLES TARGET=Fatal_rate Nonfatal_rate INPUT= Inspector_ratio<br>/CRITERIA CILEVEL=95 CITYPE=PERCENTILE NSAMPLES=2000<br>/MISSING USERMISSING=EXCLUDE.
Resources	Processor Time	00:00:00.02
	Elapsed Time	00:00:00.03

Bootstrap Specifications

Sampling Method	Simple
Number of Samples	2000
Confidence Interval Level	95.0%
Confidence Interval Type	Percentile

General Linear Model

Notes

Output Created		24-FEB-2026 11:39:45
Comments		
Input	Data	E:\Group 1 - Feb 24\Group 1 - Descriptive and regression analysis -item 2 Rerun and all the rest.sav
	Active Dataset	DataSet2
	Filter	<none>
	Weight	<none>
	Split File	<none>
	N of Rows in Working Data File	68034
Missing Value Handling	Definition of Missing	User-defined missing values are treated as missing.

	Cases Used	Statistics are based on all cases with valid data for all variables in the model.
Syntax		GLM Fatal_rate Nonfatal_rate WITH Inspector_ratio<br>/METHOD=SSTYPE(3)<br>/INTERCEPT=INCLUDE<br>/SAVE=PRED SEPRED RESID ZRESID COOK LEVER<br>/EMMEANS=TABLES(OVERALL) WITH(Inspector_ratio=MEAN)<br>/PRINT=DESCRIPTIVE ETASQ OPOWER PARAMETER TEST(SSCP) RSSCP HOMOGENEITY LOF<br>/PLOT=SPREADLEVEL RESIDUALS<br>/CRITERIA=ALPHA(.05)<br>/DESIGN=Inspector_ratio.
Resources	Processor Time	00:00:29.84
	Elapsed Time	00:00:28.61

Warnings

The HOMOGENEITY specification in the PRINT subcommand will be ignored because there are no between-subjects factors.
The SPREADLEVEL specification has been found in the PLOT subcommand, but the model includes no factors. The spread versus level plot will not be produced.
This command is trying to save new variables while bootstrapping is in effect. The new variables will be lost.<br>Execution of this command stops.

Descriptive Statistics

	Mean	Std. Deviation	N
Fatal_rate	1.671029	.9263506	34
Nonfatal_rate	981.647471	854.6068051	34

Bartlett's Test of Sphericity[a]

Likelihood Ratio	.000
Approx. Chi-Square	381.772
df	2
Sig.	<.001

Tests the null hypothesis that the residual covariance matrix is proportional to an identity matrix.[a]
a. Design: Intercept + Inspector_ratio

Multivariate Tests[a]

Effect		Value	F	Hypothesis df	Error df	Sig.
Intercept	Pillai's Trace	.341	8.013[b]	2.000	31.000	.002
	Wilks' Lambda	.659	8.013[b]	2.000	31.000	.002
	Hotelling's Trace	.517	8.013[b]	2.000	31.000	.002
	Roy's Largest Root	.517	8.013[b]	2.000	31.000	.002
Inspector_ratio	Pillai's Trace	.036	.576[b]	2.000	31.000	.568
	Wilks' Lambda	.964	.576[b]	2.000	31.000	.568
	Hotelling's Trace	.037	.576[b]	2.000	31.000	.568
	Roy's Largest Root	.037	.576[b]	2.000	31.000	.568

Multivariate Tests[a]

Effect		Partial Eta Squared	Noncent. Parameter	Observed Power[c]
Intercept	Pillai's Trace	.341	16.027	.937
	Wilks' Lambda	.341	16.027	.937
	Hotelling's Trace	.341	16.027	.937
	Roy's Largest Root	.341	16.027	.937
Inspector_ratio	Pillai's Trace	.036	1.152	.137
	Wilks' Lambda	.036	1.152	.137
	Hotelling's Trace	.036	1.152	.137
	Roy's Largest Root	.036	1.152	.137

a. Design: Intercept + Inspector_ratio
b. Exact statistic
c. Computed using alpha = .05

Tests of Between-Subjects Effects

Source	Dependent Variable	Type III Sum of Squares	df	Mean Square	F	Sig.
Corrected Model	Fatal_rate	.273[a]	1	.273	.312	.580
	Nonfatal_rate	803249.301[b]	1	803249.301	1.103	.301
Intercept	Fatal_rate	13.754	1	13.754	15.694	<.001
	Nonfatal_rate	2711048.035	1	2711048.035	3.724	.063
Inspector_ratio	Fatal_rate	.273	1	.273	.312	.580
	Nonfatal_rate	803249.301	1	803249.301	1.103	.301
Error	Fatal_rate	28.045	32	.876		
	Nonfatal_rate	23298392.812	32	728074.775		
Total	Fatal_rate	123.258	34			
	Nonfatal_rate	56865121.835	34			
Corrected Total	Fatal_rate	28.318	33			
	Nonfatal_rate	24101642.114	33			

Tests of Between-Subjects Effects

Source	Dependent Variable	Partial Eta Squared	Noncent. Parameter	Observed Power[c]
Corrected Model	Fatal_rate	.010	.312	.084
	Nonfatal_rate	.033	1.103	.175
Intercept	Fatal_rate	.329	15.694	.970
	Nonfatal_rate	.104	3.724	.465
Inspector_ratio	Fatal_rate	.010	.312	.084
	Nonfatal_rate	.033	1.103	.175
Error	Fatal_rate			
	Nonfatal_rate			
Total	Fatal_rate			
	Nonfatal_rate			
Corrected Total	Fatal_rate			
	Nonfatal_rate			

a. R Squared = .010 (Adjusted R Squared = -.021)
b. R Squared = .033 (Adjusted R Squared = .003)
c. Computed using alpha = .05

Parameter Estimates

Dependent Variable	Parameter	B	Std. Error	t	Sig.	95% Confidence Interval Lower Bound
Fatal_rate	Intercept	1.482	.374	3.962	<.001	.720
	Inspector_ratio	.237	.425	.558	.580	-.628
Nonfatal_rate	Intercept	658.088	341.038	1.930	.063	-36.585
	Inspector_ratio	406.693	387.194	1.050	.301	-381.997

Parameter Estimates

Dependent Variable	Parameter	95% Confidence Interval Upper Bound	Partial Eta Squared	Noncent. Parameter	Observed Power[a]
Fatal_rate	Intercept	2.244	.329	3.962	.970
	Inspector_ratio	1.103	.010	.558	.084
Nonfatal_rate	Intercept	1352.760	.104	1.930	.465
	Inspector_ratio	1195.382	.033	1.050	.175

a. Computed using alpha = .05

Bootstrap for Parameter Estimates

Dependent Variable	Parameter	B	Bootstrap[a] Bias	Std. Error	Sig. (2-tailed)	95% Confidence Interval Lower
Fatal_rate	Intercept	1.482	-.008	.378	.002	.751
	Inspector_ratio	.237	.012	.440	.597	-.577
Nonfatal_rate	Intercept	658.088	-3.441	263.784	.018	186.850
	Inspector_ratio	406.693	16.338	326.649	.218	-177.764

Bootstrap for Parameter Estimates

Dependent Variable	Parameter	Bootstrap 95% Confidence Interval Upper
Fatal_rate	Intercept	2.206
	Inspector_ratio	1.161
Nonfatal_rate	Intercept	1203.473
	Inspector_ratio	1118.837

a. Unless otherwise noted, bootstrap results are based on 2000 bootstrap samples

Between-Subjects SSCP Matrix

			Fatal_rate	Nonfatal_rate
Hypothesis	Intercept	Fatal_rate	13.754	6106.393
		Nonfatal_rate	6106.393	2711048.035
	Inspector_ratio	Fatal_rate	.273	468.569
		Nonfatal_rate	468.569	803249.301
Error		Fatal_rate	28.045	6710.832
		Nonfatal_rate	6710.832	23298392.812

Based on Type III Sum of Squares

Residual SSCP Matrix

		Fatal_rate	Nonfatal_rate
Sum-of-Squares and Cross-Products	Fatal_rate	28.045	6710.832
	Nonfatal_rate	6710.832	23298392.812
Covariance	Fatal_rate	.876	209.714
	Nonfatal_rate	209.714	728074.775
Correlation	Fatal_rate	1.000	.263
	Nonfatal_rate	.263	1.000

Based on Type III Sum of Squares

Lack of Fit

Multivariate Tests

Dependent Variables		Value	F	Hypothesis df	Error df
Fatal_rate, Nonfatal_rate	Pillai's Trace	1.938	2.069	60.000	4.000
	Wilks' Lambda	.001	1.131[a]	60.000	2.000
	Hotelling's Trace	74.191	.000	60.000	.000
	Roy's Largest Root	52.294	3.486[b]	30.000	2.000
Fatal_rate	Pillai's Trace	.957	1.472[a]	30.000	2.000
	Wilks' Lambda	.043	1.472[a]	30.000	2.000
	Hotelling's Trace	22.077	1.472[a]	30.000	2.000
	Roy's Largest Root	22.077	1.472[a]	30.000	2.000
Nonfatal_rate	Pillai's Trace	.978	2.931[a]	30.000	2.000
	Wilks' Lambda	.022	2.931[a]	30.000	2.000
	Hotelling's Trace	43.961	2.931[a]	30.000	2.000
	Roy's Largest Root	43.961	2.931[a]	30.000	2.000

Multivariate Tests

Dependent Variables		Sig.	Partial Eta Squared	Noncent. Parameter
Fatal_rate, Nonfatal_rate	Pillai's Trace	.252	.969	124.128
	Wilks' Lambda	.582	.971	67.865
	Hotelling's Trace	.	.974	.000
	Roy's Largest Root	.247	.981	104.588
Fatal_rate	Pillai's Trace	.485	.957	44.155
	Wilks' Lambda	.485	.957	44.155
	Hotelling's Trace	.485	.957	44.155
	Roy's Largest Root	.485	.957	44.155
Nonfatal_rate	Pillai's Trace	.286	.978	87.922
	Wilks' Lambda	.286	.978	87.922
	Hotelling's Trace	.286	.978	87.922
	Roy's Largest Root	.286	.978	87.922

Multivariate Tests

Dependent Variables		Observed Power[c]
Fatal_rate, Nonfatal_rate	Pillai's Trace	.293
	Wilks' Lambda	.104
	Hotelling's Trace	.
	Roy's Largest Root	.205
Fatal_rate	Pillai's Trace	.119
	Wilks' Lambda	.119
	Hotelling's Trace	.119
	Roy's Largest Root	.119
Nonfatal_rate	Pillai's Trace	.182
	Wilks' Lambda	.182
	Hotelling's Trace	.182
	Roy's Largest Root	.182

a. Exact statistic

b. The statistic is an upper bound on F that yields a lower bound on the significance level.
c. Computed using alpha = .05

Univariate Tests

Dependent Variable	Source	Sum of Squares	df	Mean Square	F	Sig.
Fatal_rate	Lack of Fit	26.830	30	.894	1.472	.485
	Pure Error	1.215	2	.608		
Nonfatal_rate	Lack of Fit	22780203.126	30	759340.104	2.931	.286
	Pure Error	518189.686	2	259094.843		

Univariate Tests

Dependent Variable	Source	Partial Eta Squared	Noncent. Parameter	Observed Power[a]
Fatal_rate	Lack of Fit	.957	44.155	.119
	Pure Error			
Nonfatal_rate	Lack of Fit	.978	87.922	.182
	Pure Error			

a. Computed using alpha = .05

SSCP Matrix

		Fatal_rate	Nonfatal_rate
Lack of Fit	Fatal_rate	26.830	6348.634
	Nonfatal_rate	6348.634	22780203.126
Pure Error	Fatal_rate	1.215	362.199
	Nonfatal_rate	362.199	518189.686

Estimated Marginal Means

Grand Mean

Dependent Variable	Mean	Std. Error	95% Confidence Interval Lower Bound	95% Confidence Interval Upper Bound
Fatal_rate	1.671[a]	.161	1.344	1.998
Nonfatal_rate	981.647[a]	146.335	683.573	1279.722

a. Covariates appearing in the model are evaluated at the following values: Inspector_ratio = .795588.

Descriptive statistics with Log-transformation of dependent variables reduced data (PP. 45-53)

Explore

Notes

Output Created		24-FEB-2026 11:42:27
Comments		
Input	Data	E:\Group 1 - Feb 24\Group 1 - Descriptive and regression analysis -item 2 Rerun and all the rest.sav
	Active Dataset	DataSet2
	Filter	<none>
	Weight	<none>
	Split File	<none>
	N of Rows in Working Data File	34
Missing Value Handling	Definition of Missing	User-defined missing values for dependent variables are treated as missing.
	Cases Used	Statistics are based on cases with no missing values for any dependent variable or factor used.
Syntax		EXAMINE VARIABLES=Inspector_ratio Log_fatalrate Log_nonfatalrate<br>/PLOT BOXPLOT HISTOGRAM NPPLOT<br>/COMPARE GROUPS<br>/STATISTICS DESCRIPTIVES EXTREME<br>/CINTERVAL 95<br>/MISSING LISTWISE<br>/NOTOTAL.
Resources	Processor Time	00:00:01.78
	Elapsed Time	00:00:01.32

Case Processing Summary

	Cases					
	Valid		Missing		Total	
	N	Percent	N	Percent	N	Percent
Inspector_ratio	34	100.0%	0	0.0%	34	100.0%
Log_fatalrate	34	100.0%	0	0.0%	34	100.0%
Log_nonfatalrate	34	100.0%	0	0.0%	34	100.0%

Descriptives

			Statistic	Std. Error
Inspector_ratio	Mean		.795588	.0657904
	95% Confidence Interval for Mean	Lower Bound	.661737	
		Upper Bound	.929440	
	5% Trimmed Mean		.792320	

	Median		.800000	
	Variance		.147	
	Std. Deviation		.3836206	
	Minimum		.1900	
	Maximum		1.4600	
	Range		1.2700	
	Interquartile Range		.6150	
	Skewness		.103	.403
	Kurtosis		-1.222	.788
Log_fatalrate	Mean		.1402	.05278
	95% Confidence Interval for Mean	Lower Bound	.0328	
		Upper Bound	.2475	
	5% Trimmed Mean		.1658	
	Median		.1547	
	Variance		.095	
	Std. Deviation		.30773	
	Minimum		-.92	
	Maximum		.57	
	Range		1.49	
	Interquartile Range		.36	
	Skewness		-1.347	.403
	Kurtosis		3.164	.788
Log_nonfatalrate	Mean		2.7577	.10258
	95% Confidence Interval for Mean	Lower Bound	2.5490	
		Upper Bound	2.9664	
	5% Trimmed Mean		2.8182	
	Median		2.8377	
	Variance		.358	
	Std. Deviation		.59815	
	Minimum		.49	
	Maximum		3.50	
	Range		3.00	
	Interquartile Range		.57	
	Skewness		-1.883	.403
	Kurtosis		5.336	.788

Extreme Values

			Case Number	Value
Inspector_ratio	Highest	1	18	1.4600
		2	11	1.4200
		3	4	1.4000
		4	31	1.2900
		5	25	1.2800
	Lowest	1	32	.1900
		2	14	.2300
		3	3	.2600
		4	8	.2900
		5	26	.3200
Log_fatalrate	Highest	1	34	.57

		2	17	.55
		3	8	.51
		4	6	.49
		5	25	.47
	Lowest	1	5	-.92
		2	22	-.49
		3	3	-.23
		4	30	-.15
		5	11	-.15
Log_nonfatalrate	Highest	1	6	3.50
		2	34	3.45
		3	24	3.40
		4	31	3.37
		5	2	3.37
	Lowest	1	22	.49
		2	25	1.60
		3	32	1.81
		4	3	2.04
		5	16	2.37

Tests of Normality

	Kolmogorov-Smirnov[a]			Shapiro-Wilk		
	Statistic	df	Sig.	Statistic	df	Sig.
Inspector_ratio	.116	34	.200*	.948	34	.106
Log_fatalrate	.113	34	.200*	.911	34	.009
Log_nonfatalrate	.163	34	.023	.847	34	<.001

*. This is a lower bound of the true significance.
a. Lilliefors Significance Correction

Inspector_ratio

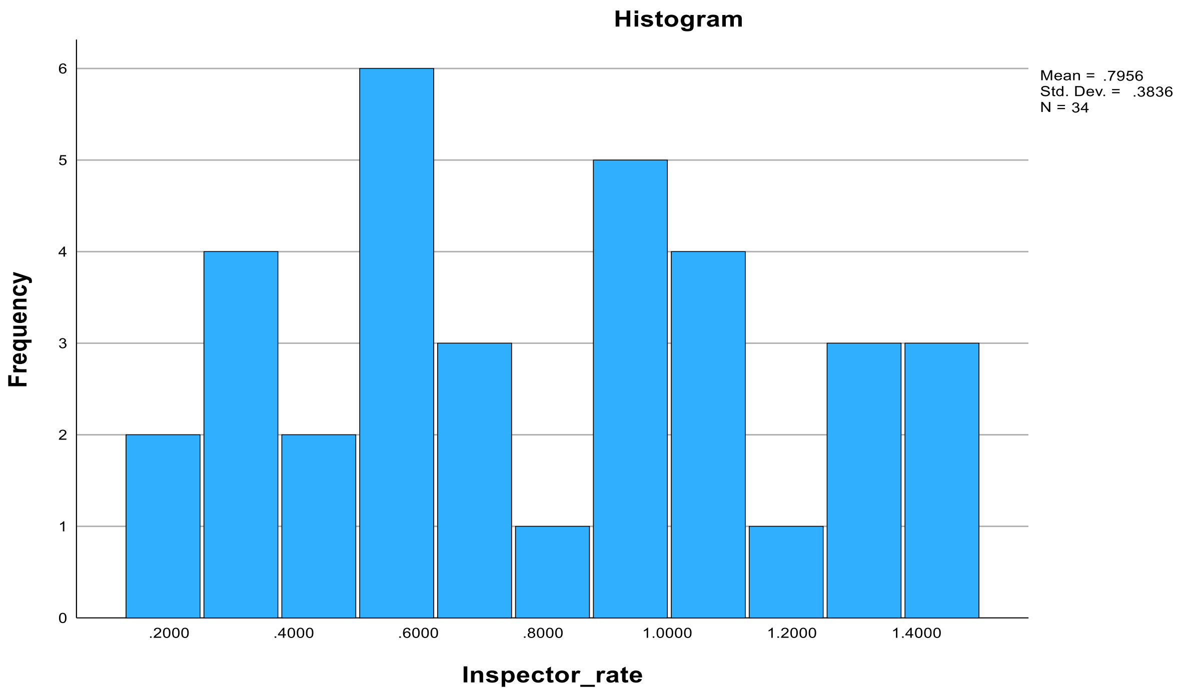

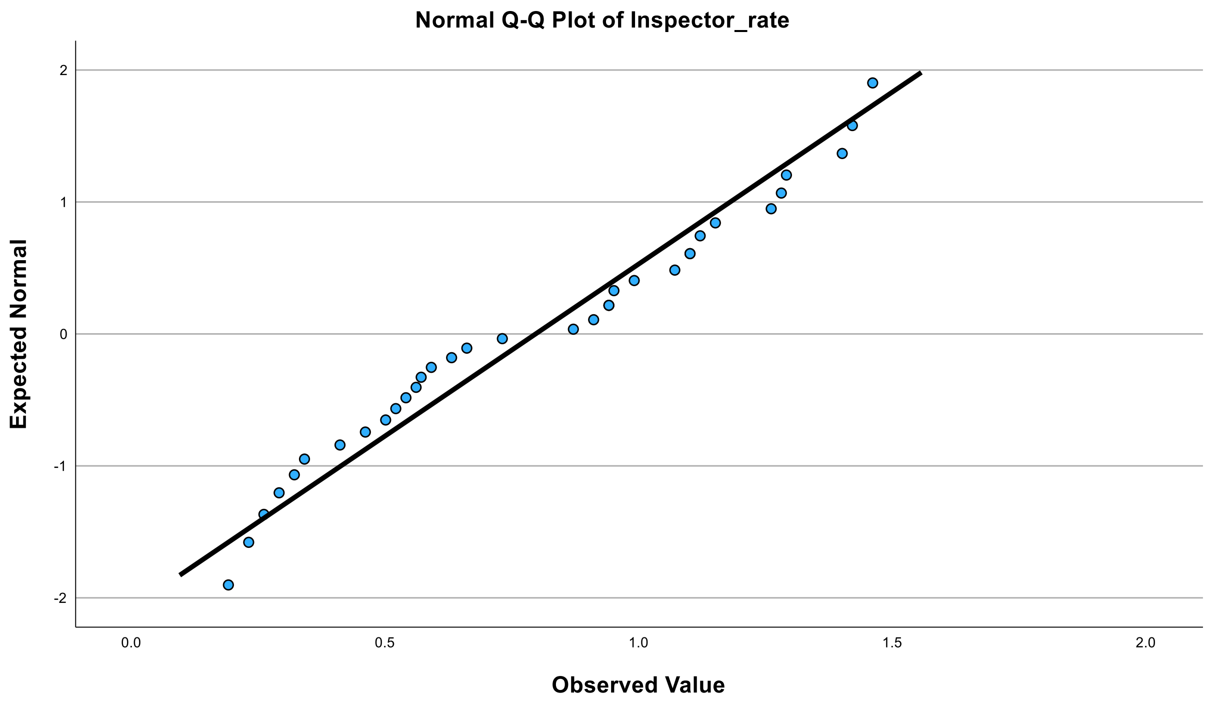

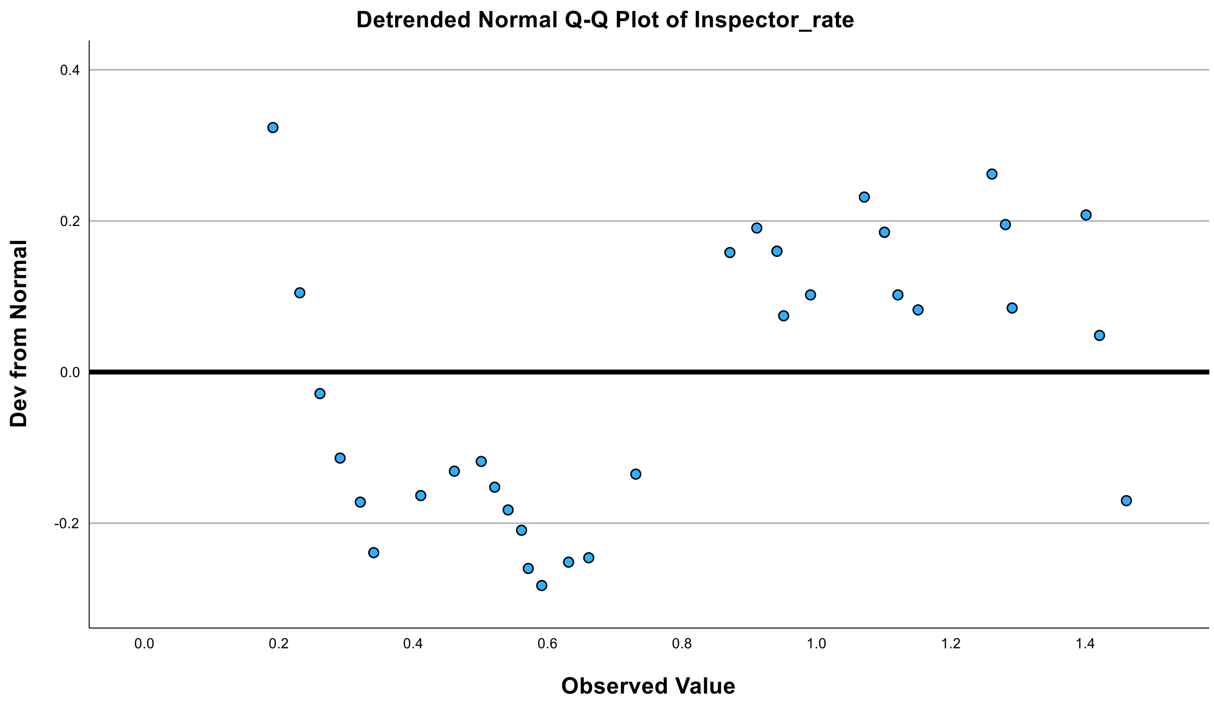
Detrended Normal Q-Q Plot of Inspector_rate
Dev from Normal
0.4
0.2
0.0
-0.2
0.0
0.2
0.4
0.6
0.8
1.0
1.2
1.4
Observed Value

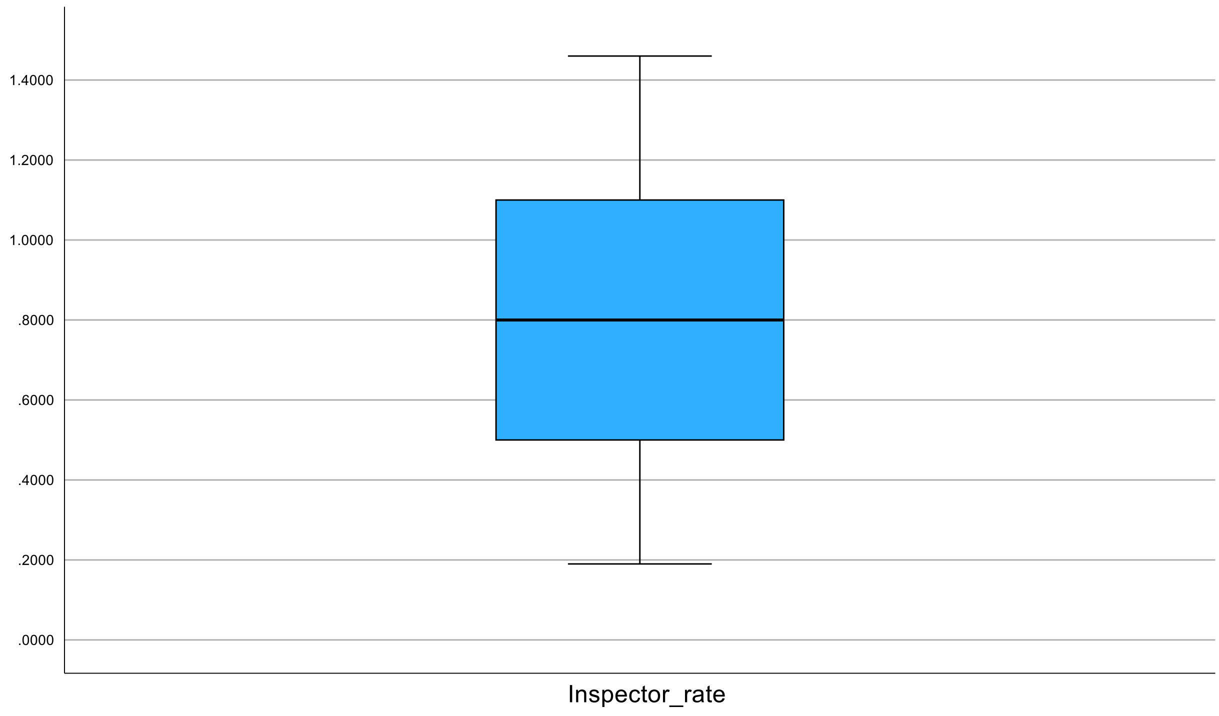
1.4000
1.2000
1.0000
.8000
.6000
.4000
.2000
.0000
Inspector_rate

Log_fatalrate

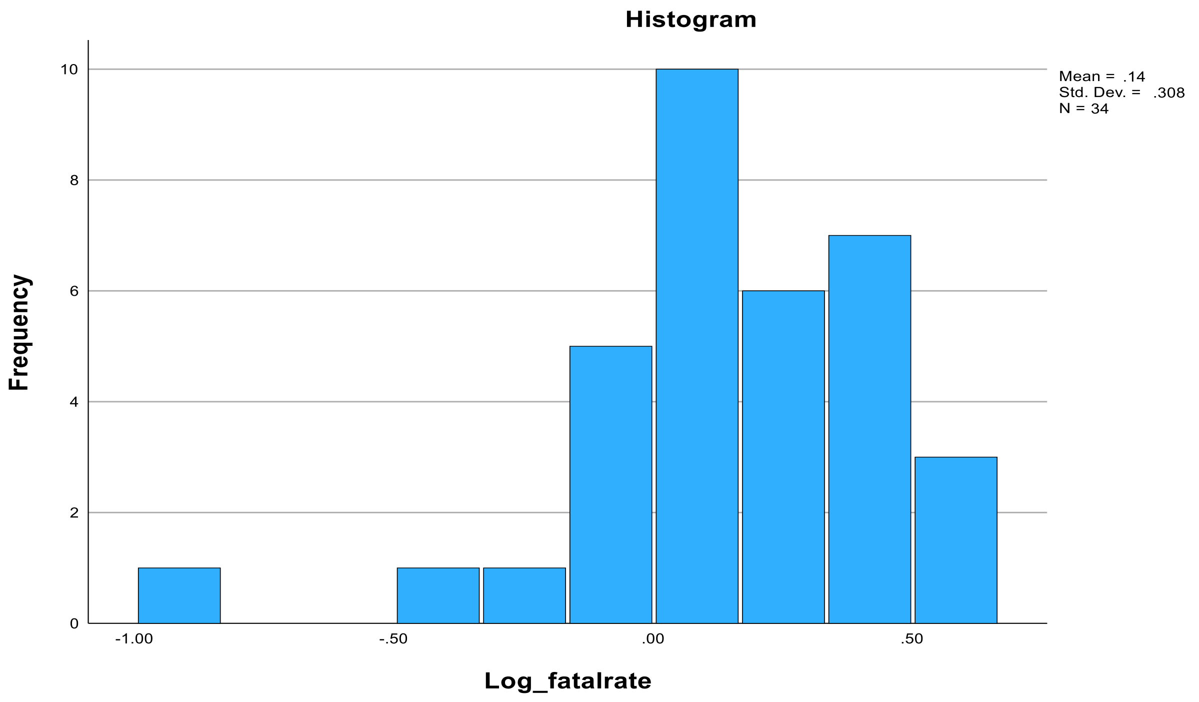

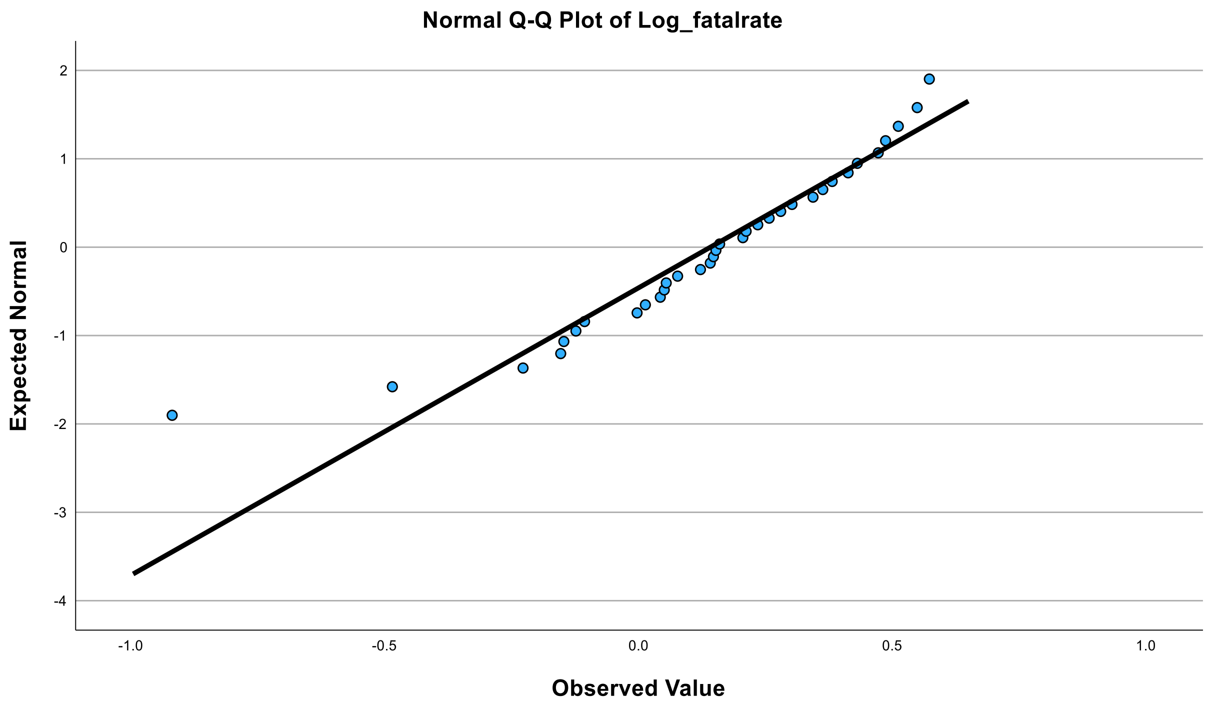

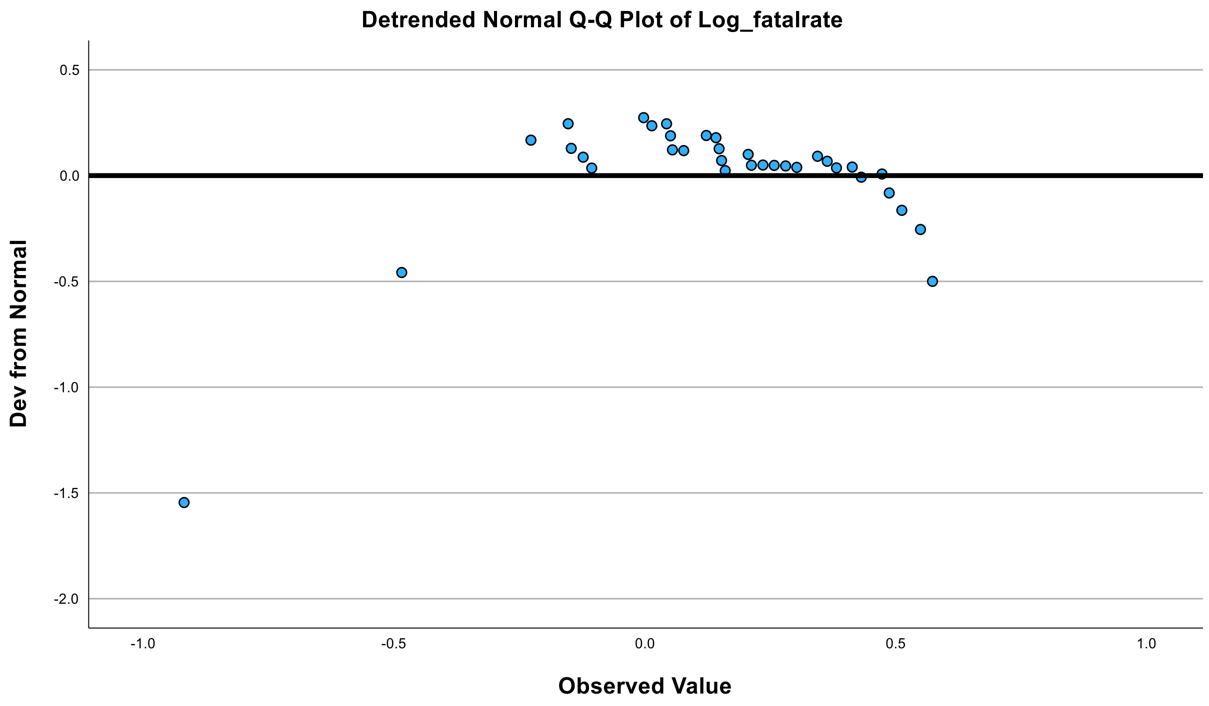
Detrended Normal Q-Q Plot of Log_fatalrate
Dev from Normal
Observed Value
0.5
0.0
-0.5
-1.0
-1.5
-2.0
-1.0
-0.5
0.0
0.5
1.0

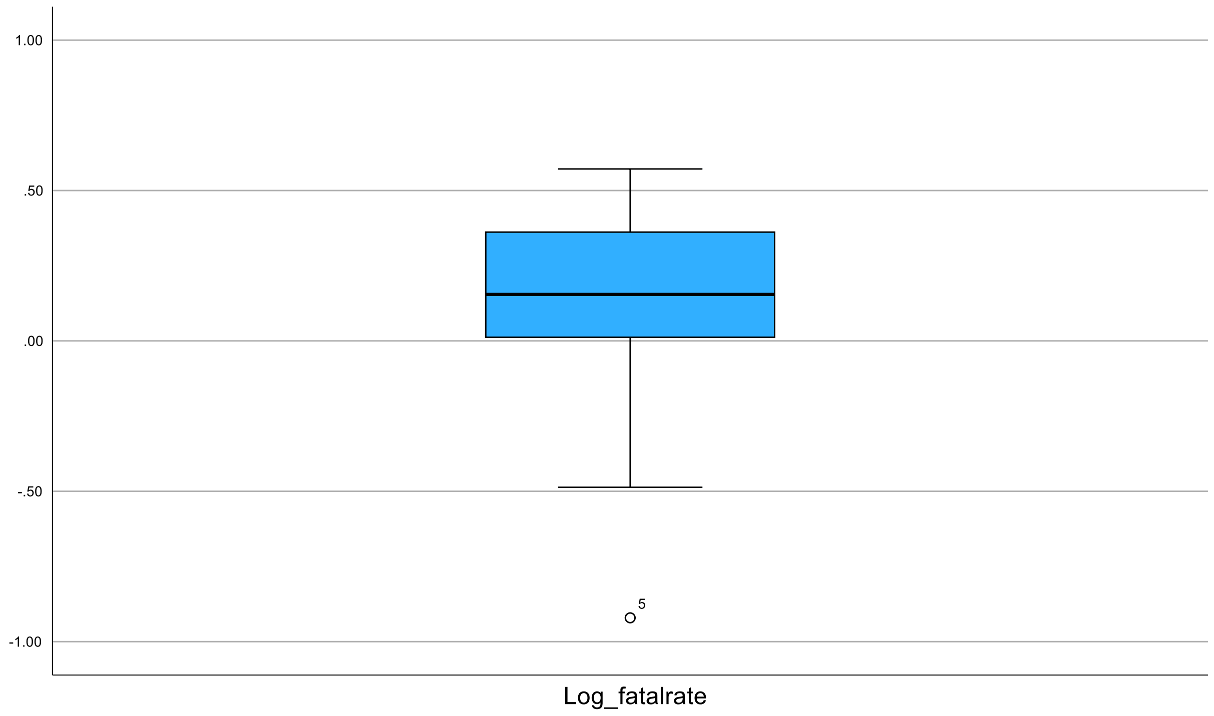
1.00
.50
.00
-.50
-1.00
5
Log_fatalrate

Log_nonfatalrate

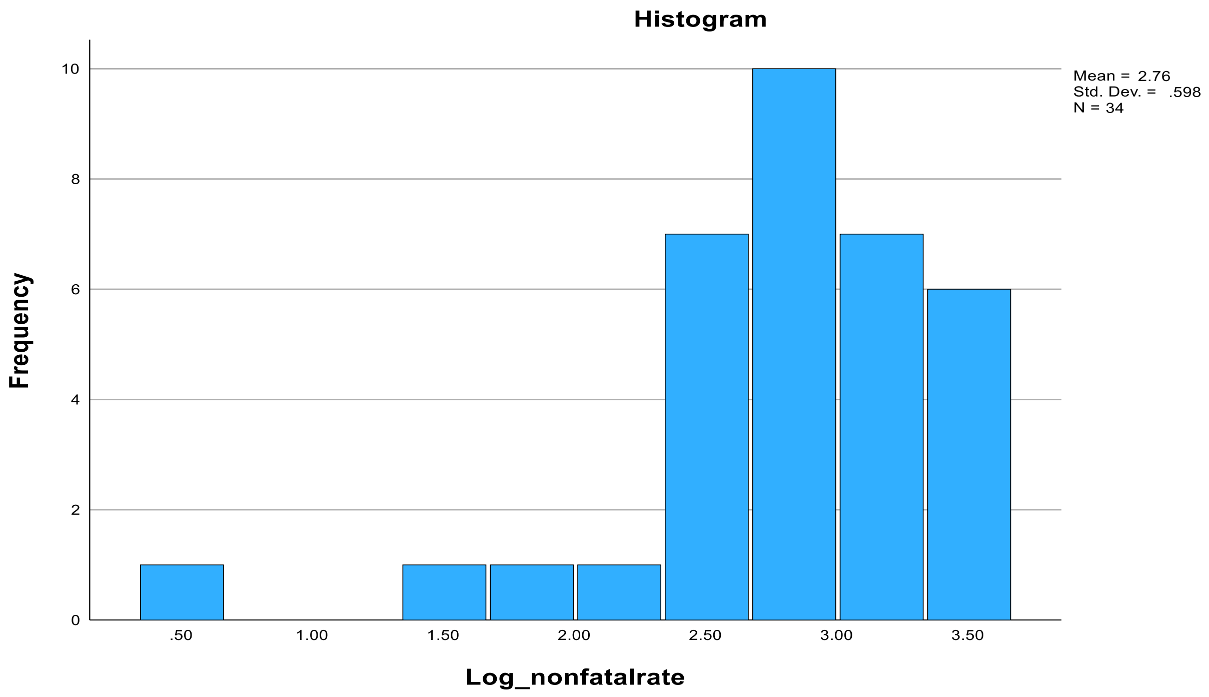

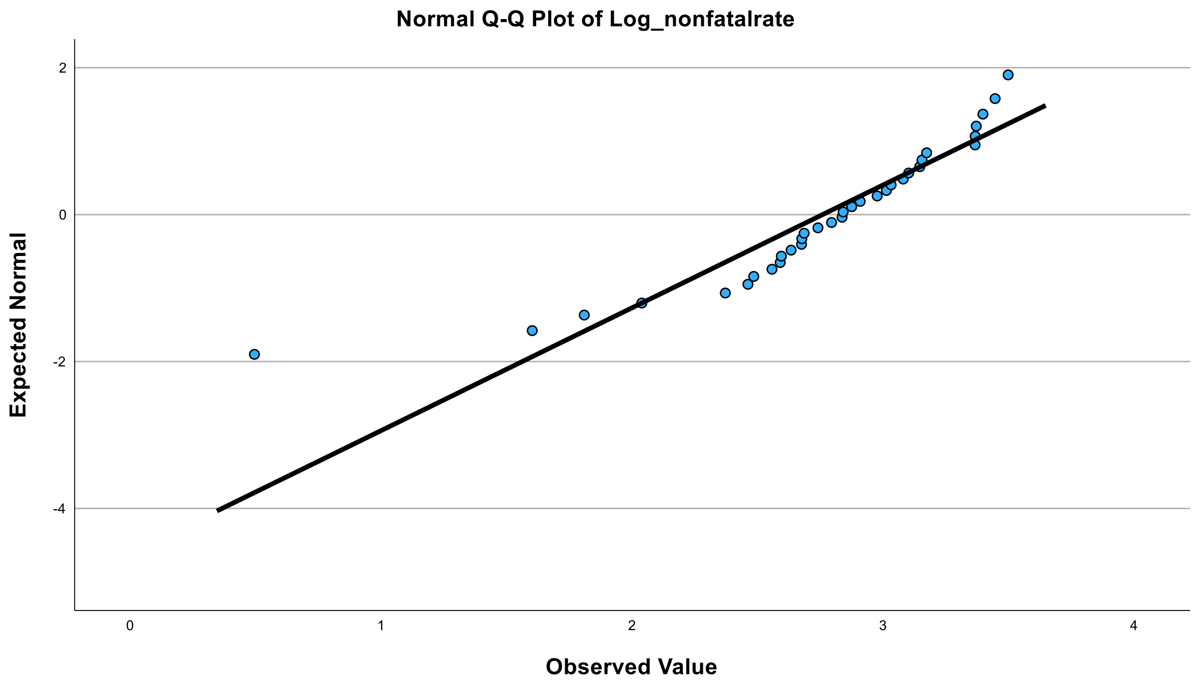

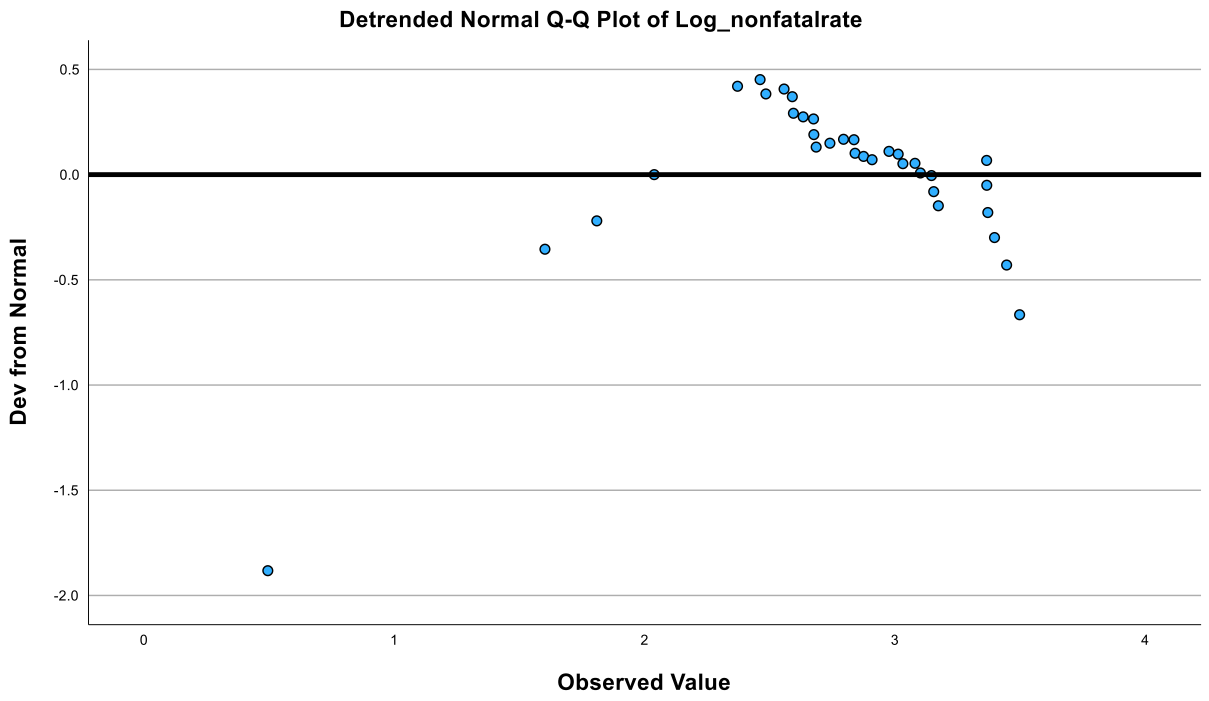
Detrended Normal Q-Q Plot of Log_nonfatalrate
Dev from Normal
0.5
0.0
-0.5
-1.0
-1.5
-2.0
0
1
2
3
4
Observed Value

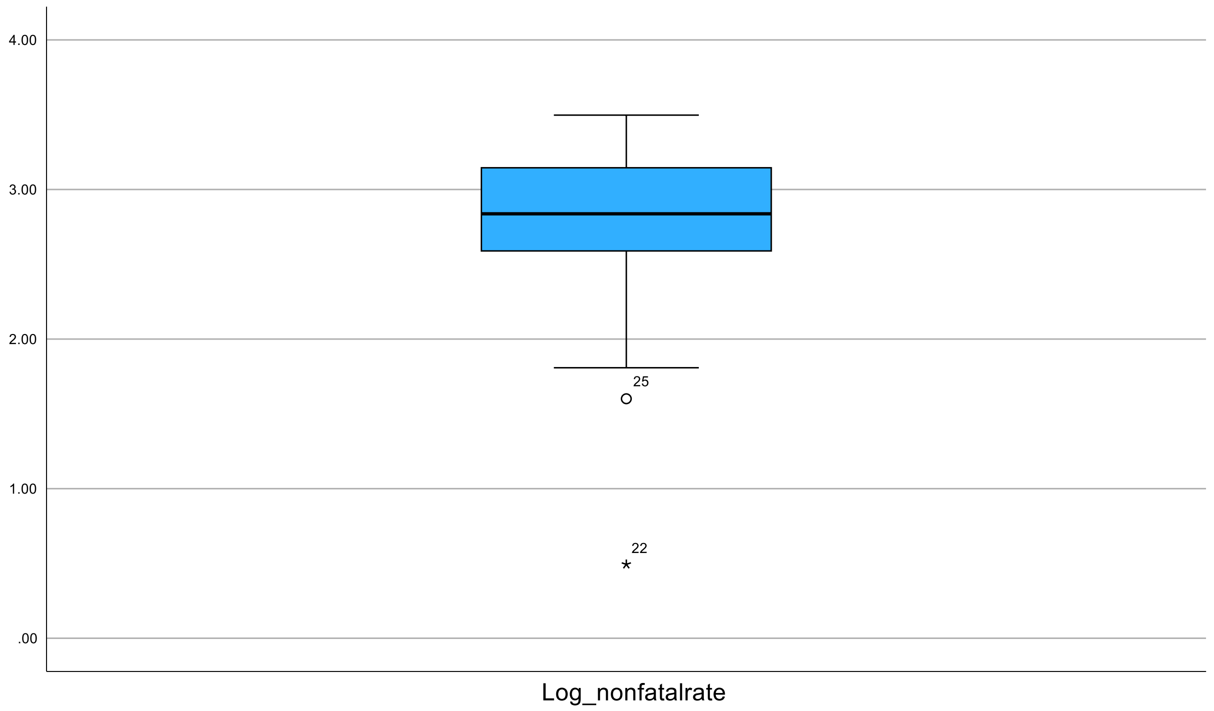
4.00
3.00
2.00
1.00
.00
25
22
Log_nonfatalrate

Log-multivariate general linear regression with bootstrap-2000 at 95% CIs with reduced data (PP.54-60)

Notes

Output Created		24-FEB-2026 11:46:43
Comments		
Input	Data	E:\Group 1 - Feb 24\Group 1 - Descriptive and regression analysis -item 2 Rerun and all the rest.sav
	Active Dataset	DataSet2
	Filter	<none>
	Weight	<none>
	Split File	<none>
Syntax		BOOTSTRAP<br>/SAMPLING METHOD=SIMPLE<br>/VARIABLES TARGET=Log_fatalrate Log_nonfatalrate INPUT= Inspector_ratio<br>/CRITERIA CILEVEL=95 CITYPE=PERCENTILE NSAMPLES=2000<br>/MISSING USERMISSING=EXCLUDE.
Resources	Processor Time	00:00:00.03
	Elapsed Time	00:00:00.02

Bootstrap Specifications

Sampling Method	Simple
Number of Samples	2000
Confidence Interval Level	95.0%
Confidence Interval Type	Percentile

General Linear Model

Notes

Output Created		24-FEB-2026 11:46:43
Comments		
Input	Data	E:\Group 1 - Feb 24\Group 1 - Descriptive and regression analysis -item 2 Rerun and all the rest.sav
	Active Dataset	DataSet2
	Filter	<none>
	Weight	<none>
	Split File	<none>
	N of Rows in Working Data File	68034
Missing Value Handling	Definition of Missing	User-defined missing values are treated as missing.

	Cases Used	Statistics are based on all cases with valid data for all variables in the model.
Syntax		GLM Log_fatalrate Log_nonfatalrate WITH Inspector_ratio<br>/METHOD=SSTYPE(3)<br>/INTERCEPT=INCLUDE<br>/SAVE=PRED SEPRED RESID ZRESID COOK LEVER<br>/PRINT=DESCRIPTIVE ETASQ OPOWER PARAMETER TEST(SSCP) RSSCP HOMOGENEITY LOF<br>/PLOT=SPREADLEVEL RESIDUALS<br>/CRITERIA=ALPHA(.05)<br>/DESIGN=Inspector_ratio.
Resources	Processor Time	00:00:27.23
	Elapsed Time	00:00:27.04

Warnings

The HOMOGENEITY specification in the PRINT subcommand will be ignored because there are no between-subjects factors.
The SPREADLEVEL specification has been found in the PLOT subcommand, but the model includes no factors. The spread versus level plot will not be produced.
This command is trying to save new variables while bootstrapping is in effect. The new variables will be lost.<br>Execution of this command stops.

Descriptive Statistics

	Mean	Std. Deviation	N
Log_fatalrate	.1402	.30773	34
Log_nonfatalrate	2.7577	.59815	34

Bartlett's Test of Sphericity[a]

Likelihood Ratio	.001
Approx. Chi-Square	13.800
df	2
Sig.	.001

Tests the null hypothesis that the residual covariance matrix is proportional to an identity matrix.[a]

a. Design: Intercept + Inspector_ratio

Multivariate Tests[a]

Effect		Value	F	Hypothesis df	Error df	Sig.
Intercept	Pillai's Trace	.783	55.766[b]	2.000	31.000	<.001
	Wilks' Lambda	.217	55.766[b]	2.000	31.000	<.001
	Hotelling's Trace	3.598	55.766[b]	2.000	31.000	<.001
	Roy's Largest Root	3.598	55.766[b]	2.000	31.000	<.001
Inspector_ratio	Pillai's Trace	.044	.719[b]	2.000	31.000	.495
	Wilks' Lambda	.956	.719[b]	2.000	31.000	.495
	Hotelling's Trace	.046	.719[b]	2.000	31.000	.495
	Roy's Largest Root	.046	.719[b]	2.000	31.000	.495

Multivariate Tests[a]

Effect		Partial Eta Squared	Noncent. Parameter	Observed Power[c]
Intercept	Pillai's Trace	.783	111.532	1.000
	Wilks' Lambda	.783	111.532	1.000
	Hotelling's Trace	.783	111.532	1.000
	Roy's Largest Root	.783	111.532	1.000
Inspector_ratio	Pillai's Trace	.044	1.437	.160
	Wilks' Lambda	.044	1.437	.160
	Hotelling's Trace	.044	1.437	.160
	Roy's Largest Root	.044	1.437	.160

a. Design: Intercept + Inspector_ratio
b. Exact statistic
c. Computed using alpha = .05

Tests of Between-Subjects Effects

Source	Dependent Variable	Type III Sum of Squares	df	Mean Square	F	Sig.
Corrected Model	Log_fatalrate	.054[a]	1	.054	.568	.457
	Log_nonfatalrate	.424[b]	1	.424	1.192	.283
Intercept	Log_fatalrate	.020	1	.020	.204	.655
	Log_nonfatalrate	39.835	1	39.835	111.988	<.001
Inspector_ratio	Log_fatalrate	.054	1	.054	.568	.457
	Log_nonfatalrate	.424	1	.424	1.192	.283
Error	Log_fatalrate	3.071	32	.096		
	Log_nonfatalrate	11.383	32	.356		
Total	Log_fatalrate	3.793	34			
	Log_nonfatalrate	270.373	34			
Corrected Total	Log_fatalrate	3.125	33			
	Log_nonfatalrate	11.807	33			

Tests of Between-Subjects Effects

Source	Dependent Variable	Partial Eta Squared	Noncent. Parameter	Observed Power[c]
Corrected Model	Log_fatalrate	.017	.568	.113
	Log_nonfatalrate	.036	1.192	.185
Intercept	Log_fatalrate	.006	.204	.072
	Log_nonfatalrate	.778	111.988	1.000
Inspector_ratio	Log_fatalrate	.017	.568	.113
	Log_nonfatalrate	.036	1.192	.185
Error	Log_fatalrate			
	Log_nonfatalrate			
Total	Log_fatalrate			
	Log_nonfatalrate			
Corrected Total	Log_fatalrate			
	Log_nonfatalrate			

a. R Squared = .017 (Adjusted R Squared = -.013)
b. R Squared = .036 (Adjusted R Squared = .006)
c. Computed using alpha = .05

Parameter Estimates

Dependent Variable	Parameter	B	Std. Error	t	Sig.	95% Confidence Interval Lower Bound
Log_fatalrate	Intercept	.056	.124	.451	.655	-.196
	Inspector_ratio	.106	.141	.754	.457	-.180
Log_nonfatalrate	Intercept	2.523	.238	10.582	<.001	2.037
	Inspector_ratio	.296	.271	1.092	.283	-.256

Parameter Estimates

Dependent Variable	Parameter	95% Confidence Interval Upper Bound	Partial Eta Squared	Noncent. Parameter	Observed Power[a]
Log_fatalrate	Intercept	.308	.006	.451	.072
	Inspector_ratio	.392	.017	.754	.113
Log_nonfatalrate	Intercept	3.008	.778	10.582	1.000
	Inspector_ratio	.847	.036	1.092	.185

a. Computed using alpha = .05

Bootstrap for Parameter Estimates

Dependent Variable	Parameter	B	Bootstrap[a] Bias	Std. Error	Sig. (2-tailed)	95% Confidence Interval Lower
Log_fatalrate	Intercept	.056	-.003	.128	.672	-.217
	Inspector_ratio	.106	.003	.130	.428	-.137
Log_nonfatalrate	Intercept	2.523	.002	.244	<.001	2.020
	Inspector_ratio	.296	.007	.258	.272	-.218

Bootstrap for Parameter Estimates

Dependent Variable	Parameter	Bootstrap 95% Confidence Interval Upper
Log_fatalrate	Intercept	.292
	Inspector_ratio	.384
Log_nonfatalrate	Intercept	3.008
	Inspector_ratio	.833

a. Unless otherwise noted, bootstrap results are based on 2000 bootstrap samples

Between-Subjects SSCP Matrix

			Log_fatalrate	Log_nonfatalrate
Hypothesis	Intercept	Log_fatalrate	.020	.883
		Log_nonfatalrate	.883	39.835
	Inspector_ratio	Log_fatalrate	.054	.152
		Log_nonfatalrate	.152	.424
Error		Log_fatalrate	3.071	1.221
		Log_nonfatalrate	1.221	11.383

Based on Type III Sum of Squares

Residual SSCP Matrix

		Log_fatalrate	Log_nonfatalrate
Sum-of-Squares and Cross-Products	Log_fatalrate	3.071	1.221
	Log_nonfatalrate	1.221	11.383
Covariance	Log_fatalrate	.096	.038
	Log_nonfatalrate	.038	.356
Correlation	Log_fatalrate	1.000	.207
	Log_nonfatalrate	.207	1.000

Based on Type III Sum of Squares

Lack of Fit

Multivariate Tests

Dependent Variables		Value	F	Hypothesis df	Error df
Log_fatalrate, Log_nonfatalrate	Pillai's Trace	1.975	5.222	60.000	4.000
	Wilks' Lambda	.000	5.451[a]	60.000	2.000
	Hotelling's Trace	680.453	.000	60.000	.000
	Roy's Largest Root	639.166	42.611[b]	30.000	2.000
Log_fatalrate	Pillai's Trace	.976	2.763[a]	30.000	2.000
	Wilks' Lambda	.024	2.763[a]	30.000	2.000
	Hotelling's Trace	41.443	2.763[a]	30.000	2.000
	Roy's Largest Root	41.443	2.763[a]	30.000	2.000
Log_nonfatalrate	Pillai's Trace	.997	21.146[a]	30.000	2.000
	Wilks' Lambda	.003	21.146[a]	30.000	2.000
	Hotelling's Trace	317.183	21.146[a]	30.000	2.000
	Roy's Largest Root	317.183	21.146[a]	30.000	2.000

Multivariate Tests

Dependent Variables		Sig.	Partial Eta Squared	Noncent. Parameter
Log_fatalrate, Log_nonfatalrate	Pillai's Trace	.058	.987	313.332
	Wilks' Lambda	.167	.994	327.062
	Hotelling's Trace	.	.997	.000
	Roy's Largest Root	.023	.998	1278.332
Log_fatalrate	Pillai's Trace	.301	.976	82.886
	Wilks' Lambda	.301	.976	82.886
	Hotelling's Trace	.301	.976	82.886
	Roy's Largest Root	.301	.976	82.886
Log_nonfatalrate	Pillai's Trace	.046	.997	634.365
	Wilks' Lambda	.046	.997	634.365
	Hotelling's Trace	.046	.997	634.365
	Roy's Largest Root	.046	.997	634.365

Multivariate Tests

Dependent Variables		Observed Power[c]
Log_fatalrate, Log_nonfatalrate	Pillai's Trace	.639
	Wilks' Lambda	.282
	Hotelling's Trace	.
	Roy's Largest Root	.893
Log_fatalrate	Pillai's Trace	.175
	Wilks' Lambda	.175
	Hotelling's Trace	.175
	Roy's Largest Root	.175
Log_nonfatalrate	Pillai's Trace	.678
	Wilks' Lambda	.678
	Hotelling's Trace	.678
	Roy's Largest Root	.678

a. Exact statistic
b. The statistic is an upper bound on F that yields a lower bound on the significance level.
c. Computed using alpha = .05

Univariate Tests

Dependent Variable	Source	Sum of Squares	df	Mean Square	F	Sig.
Log_fatalrate	Lack of Fit	2.998	30	.100	2.763	.301
	Pure Error	.072	2	.036		
Log_nonfatalrate	Lack of Fit	11.347	30	.378	21.146	.046
	Pure Error	.036	2	.018		

Univariate Tests

Dependent Variable	Source	Partial Eta Squared	Noncent. Parameter	Observed Power[a]
Log_fatalrate	Lack of Fit	.976	82.886	.175
	Pure Error			
Log_nonfatalrate	Lack of Fit	.997	634.365	.678
	Pure Error			

a. Computed using alpha = .05

SSCP Matrix

		Log_fatalrate	Log_nonfatalrate
Lack of Fit	Log_fatalrate	2.998	1.184
	Log_nonfatalrate	1.184	11.347
Pure Error	Log_fatalrate	.072	.037
	Log_nonfatalrate	.037	.036

Continued.

Log-General linear multivariate regression with reduced data (PP. 61-67)

Notes

Output Created		24-FEB-2026 11:47:59
Comments		
Input	Data	E:\Group 1 - Feb 24\Group 1 - Descriptive and regression analysis -item 2 Rerun and all the rest.sav
	Active Dataset	DataSet2
	Filter	<none>
	Weight	<none>
	Split File	<none>
	N of Rows in Working Data File	34
Missing Value Handling	Definition of Missing	User-defined missing values are treated as missing.
	Cases Used	Statistics are based on all cases with valid data for all variables in the model.
Syntax		GLM Log_fatalrate Log_nonfatalrate WITH Inspector_ratio /METHOD=SSTYPE(3) /INTERCEPT=INCLUDE /SAVE=PRED SEPRED RESID ZRESID COOK LEVER /EMMEANS=TABLES(OVERALL) WITH(Inspector_ratio=MEAN) /PRINT=DESCRIPTIVE ETASQ OPOWER PARAMETER TEST(SSCP) RSSCP HOMOGENEITY LOF /PLOT=SPREADLEVEL RESIDUALS /CRITERIA=ALPHA(.05) /DESIGN=Inspector_ratio.
Resources	Processor Time	00:00:00.31
	Elapsed Time	00:00:00.27
Variables Created or Modified	PRE_7	Predicted Value for Log_fatalrate
	PRE_8	Predicted Value for Log_nonfatalrate
	SEP_7	Standard Error of Predicted Value for Log_fatalrate
	SEP_8	Standard Error of Predicted Value for Log_nonfatalrate
	RES_7	Residual for Log_fatalrate
	RES_8	Residual for Log_nonfatalrate
	ZRE_7	Standardized Residual for Log_fatalrate
	ZRE_8	Standardized Residual for Log_nonfatalrate
	COO_7	Cook's Distance for Log_fatalrate
	COO_8	Cook's Distance for Log_nonfatalrate

	LEV_7	Uncentered Leverage Value for Log_fatalrate
	LEV_8	Uncentered Leverage Value for Log_nonfatalrate

Warnings

The HOMOGENEITY specification in the PRINT subcommand will be ignored because there are no between-subjects factors.
The SPREADLEVEL specification has been found in the PLOT subcommand, but the model includes no factors. The spread versus level plot will not be produced.

Descriptive Statistics

	Mean	Std. Deviation	N
Log_fatalrate	.1402	.30773	34
Log_nonfatalrate	2.7577	.59815	34

Bartlett's Test of Sphericity[a]

Likelihood Ratio	.001
Approx. Chi-Square	13.800
df	2
Sig.	.001

Tests the null hypothesis that the residual covariance matrix is proportional to an identity matrix.[a]
a. Design: Intercept + Inspector_ratio

Multivariate Tests[a]

Effect		Value	F	Hypothesis df	Error df	Sig.
Intercept	Pillai's Trace	.783	55.766[b]	2.000	31.000	<.001
	Wilks' Lambda	.217	55.766[b]	2.000	31.000	<.001
	Hotelling's Trace	3.598	55.766[b]	2.000	31.000	<.001
	Roy's Largest Root	3.598	55.766[b]	2.000	31.000	<.001
Inspector_ratio	Pillai's Trace	.044	.719[b]	2.000	31.000	.495
	Wilks' Lambda	.956	.719[b]	2.000	31.000	.495
	Hotelling's Trace	.046	.719[b]	2.000	31.000	.495
	Roy's Largest Root	.046	.719[b]	2.000	31.000	.495

Multivariate Tests[a]

Effect		Partial Eta Squared	Noncent. Parameter	Observed Power[c]
Intercept	Pillai's Trace	.783	111.532	1.000
	Wilks' Lambda	.783	111.532	1.000
	Hotelling's Trace	.783	111.532	1.000
	Roy's Largest Root	.783	111.532	1.000
Inspector_ratio	Pillai's Trace	.044	1.437	.160
	Wilks' Lambda	.044	1.437	.160
	Hotelling's Trace	.044	1.437	.160
	Roy's Largest Root	.044	1.437	.160

a. Design: Intercept + Inspector_ratio

b. Exact statistic
c. Computed using alpha = .05

Tests of Between-Subjects Effects

Source	Dependent Variable	Type III Sum of Squares	df	Mean Square	F	Sig.
Corrected Model	Log_fatalrate	.054[a]	1	.054	.568	.457
	Log_nonfatalrate	.424[b]	1	.424	1.192	.283
Intercept	Log_fatalrate	.020	1	.020	.204	.655
	Log_nonfatalrate	39.835	1	39.835	111.988	<.001
Inspector_ratio	Log_fatalrate	.054	1	.054	.568	.457
	Log_nonfatalrate	.424	1	.424	1.192	.283
Error	Log_fatalrate	3.071	32	.096		
	Log_nonfatalrate	11.383	32	.356		
Total	Log_fatalrate	3.793	34			
	Log_nonfatalrate	270.373	34			
Corrected Total	Log_fatalrate	3.125	33			
	Log_nonfatalrate	11.807	33			

Tests of Between-Subjects Effects

Source	Dependent Variable	Partial Eta Squared	Noncent. Parameter	Observed Power[c]
Corrected Model	Log_fatalrate	.017	.568	.113
	Log_nonfatalrate	.036	1.192	.185
Intercept	Log_fatalrate	.006	.204	.072
	Log_nonfatalrate	.778	111.988	1.000
Inspector_ratio	Log_fatalrate	.017	.568	.113
	Log_nonfatalrate	.036	1.192	.185
Error	Log_fatalrate			
	Log_nonfatalrate			
Total	Log_fatalrate			
	Log_nonfatalrate			
Corrected Total	Log_fatalrate			
	Log_nonfatalrate			

a. R Squared = .017 (Adjusted R Squared = -.013)
b. R Squared = .036 (Adjusted R Squared = .006)
c. Computed using alpha = .05

Parameter Estimates

Dependent Variable	Parameter	B	Std. Error	t	Sig.	95% Confidence Interval Lower Bound
Log_fatalrate	Intercept	.056	.124	.451	.655	-.196
	Inspector_ratio	.106	.141	.754	.457	-.180
Log_nonfatalrate	Intercept	2.523	.238	10.582	<.001	2.037
	Inspector_ratio	.296	.271	1.092	.283	-.256

Parameter Estimates

Dependent Variable	Parameter	95% Confidence Interval Upper Bound	Partial Eta Squared	Noncent. Parameter	Observed Power[a]
Log_fatalrate	Intercept	.308	.006	.451	.072
	Inspector_ratio	.392	.017	.754	.113
Log_nonfatalrate	Intercept	3.008	.778	10.582	1.000
	Inspector_ratio	.847	.036	1.092	.185

a. Computed using alpha = .05

Between-Subjects SSCP Matrix

			Log_fatalrate	Log_nonfatalrate
Hypothesis	Intercept	Log_fatalrate	.020	.883
		Log_nonfatalrate	.883	39.835
	Inspector_ratio	Log_fatalrate	.054	.152
		Log_nonfatalrate	.152	.424
Error		Log_fatalrate	3.071	1.221
		Log_nonfatalrate	1.221	11.383

Based on Type III Sum of Squares

Residual SSCP Matrix

		Log_fatalrate	Log_nonfatalrate
Sum-of-Squares and Cross-Products	Log_fatalrate	3.071	1.221
	Log_nonfatalrate	1.221	11.383
Covariance	Log_fatalrate	.096	.038
	Log_nonfatalrate	.038	.356
Correlation	Log_fatalrate	1.000	.207
	Log_nonfatalrate	.207	1.000

Based on Type III Sum of Squares

Lack of Fit

Multivariate Tests

Dependent Variables		Value	F	Hypothesis df	Error df
Log_fatalrate, Log_nonfatalrate	Pillai's Trace	1.975	5.222	60.000	4.000
	Wilks' Lambda	.000	5.451[a]	60.000	2.000
	Hotelling's Trace	680.453	.000	60.000	.000
	Roy's Largest Root	639.166	42.611[b]	30.000	2.000
Log_fatalrate	Pillai's Trace	.976	2.763[a]	30.000	2.000
	Wilks' Lambda	.024	2.763[a]	30.000	2.000
	Hotelling's Trace	41.443	2.763[a]	30.000	2.000
	Roy's Largest Root	41.443	2.763[a]	30.000	2.000
Log_nonfatalrate	Pillai's Trace	.997	21.146[a]	30.000	2.000
	Wilks' Lambda	.003	21.146[a]	30.000	2.000
	Hotelling's Trace	317.183	21.146[a]	30.000	2.000
	Roy's Largest Root	317.183	21.146[a]	30.000	2.000

Multivariate Tests

Dependent Variables		Sig.	Partial Eta Squared	Noncent. Parameter
Log_fatalrate, Log_nonfatalrate	Pillai's Trace	.058	.987	313.332
	Wilks' Lambda	.167	.994	327.062
	Hotelling's Trace	.	.997	.000
	Roy's Largest Root	.023	.998	1278.332
Log_fatalrate	Pillai's Trace	.301	.976	82.886
	Wilks' Lambda	.301	.976	82.886
	Hotelling's Trace	.301	.976	82.886
	Roy's Largest Root	.301	.976	82.886
Log_nonfatalrate	Pillai's Trace	.046	.997	634.365
	Wilks' Lambda	.046	.997	634.365
	Hotelling's Trace	.046	.997	634.365
	Roy's Largest Root	.046	.997	634.365

Multivariate Tests

Dependent Variables		Observed Power[c]
Log_fatalrate, Log_nonfatalrate	Pillai's Trace	.639
	Wilks' Lambda	.282
	Hotelling's Trace	.
	Roy's Largest Root	.893
Log_fatalrate	Pillai's Trace	.175
	Wilks' Lambda	.175
	Hotelling's Trace	.175
	Roy's Largest Root	.175
Log_nonfatalrate	Pillai's Trace	.678
	Wilks' Lambda	.678
	Hotelling's Trace	.678
	Roy's Largest Root	.678

a. Exact statistic

b. The statistic is an upper bound on F that yields a lower bound on the significance level.

c. Computed using alpha = .05

Univariate Tests

Dependent Variable	Source	Sum of Squares	df	Mean Square	F	Sig.
Log_fatalrate	Lack of Fit	2.998	30	.100	2.763	.301
	Pure Error	.072	2	.036		
Log_nonfatalrate	Lack of Fit	11.347	30	.378	21.146	.046
	Pure Error	.036	2	.018		

Univariate Tests

Dependent Variable	Source	Partial Eta Squared	Noncent. Parameter	Observed Power[a]
Log_fatalrate	Lack of Fit	.976	82.886	.175
	Pure Error			
Log_nonfatalrate	Lack of Fit	.997	634.365	.678
	Pure Error			

a. Computed using alpha = .05

SSCP Matrix

		Log_fatalrate	Log_nonfatalrate
Lack of Fit	Log_fatalrate	2.998	1.184
	Log_nonfatalrate	1.184	11.347
Pure Error	Log_fatalrate	.072	.037
	Log_nonfatalrate	.037	.036

Estimated Marginal Means

Grand Mean

Dependent Variable	Mean	Std. Error	95% Confidence Interval Lower Bound	95% Confidence Interval Upper Bound
Log_fatalrate	.140[a]	.053	.032	.248
Log_nonfatalrate	2.758[a]	.102	2.549	2.966

a. Covariates appearing in the model are evaluated at the following values: Inspector_ratio = .795588.

Observed * Predicted * Std. Residual Plots

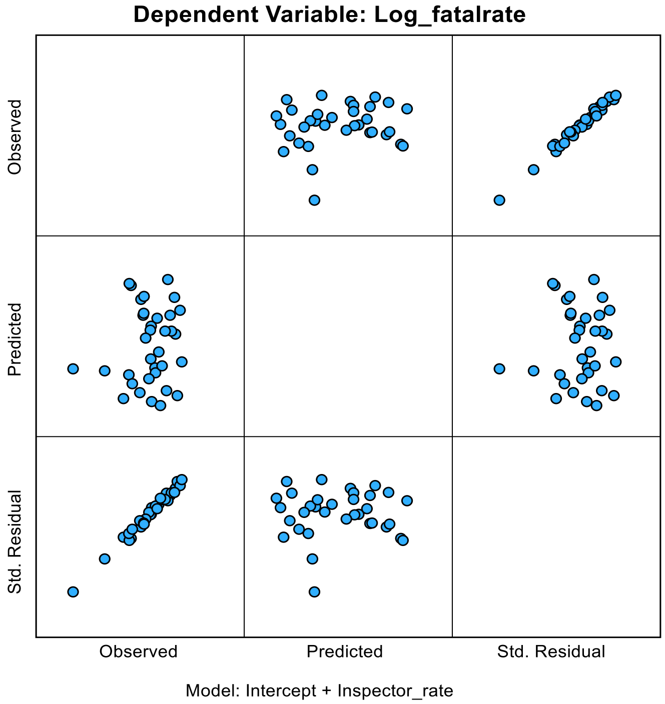

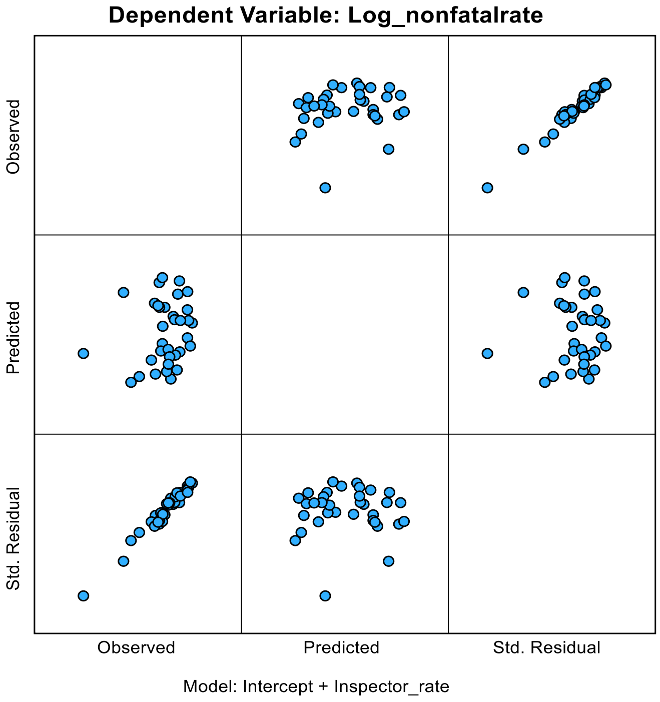

Thus, following analysis have been completed with IBM SPSS 30.0 for ILO’s group 1 as hereunder-

- Descriptive statistics with original data associated with the research variables,
- General linear multivariate regression (with re-runs where applicable),
- General linear multivariate regression with bootstrap-2000 with Confidence Intervals of 95%,
- Descriptive statistics with log-transformation of dependent variables,
- Log-general linear multivariate regression with bootstrap-2000 with Confidence Intervals of 95% and
- Log-general linear multivariate regression analysis.
