# Supplementary material for: OSH inspector ratio to strengthen decent workplace safety and health: a cross-regional trend analysis research
Source: Front Public Health. 2026 Apr 8;14:1806383. doi: 10.3389/fpubh.2026.1806383 (PMC13099828; doi:10.3389/fpubh.2026.1806383)
Supplement: Supplementary file 1 [file Data_Sheet_1.zip › Supplementary document Group 2 - Descriptive and regression analysis.pdf]

Supplementary Material

Group 2 – Descriptive and multivariate regression analysis

IBM SPSS 30.0 software was used to complete descriptive statistics and multivariate regression analysis for income group 2, which originally had International Labour Organization's (ILO) 21 nations or regions. There are six major sections in this supplementary material as listed hereunder:

1. Descriptive statistics with original data associated with the research variables (PP. 2-8),
2. General linear multivariate regression (with re-runs where applicable) (PP. 11-24),
3. General linear multivariate regression with bootstrap-2000 with Confidence Intervals of 95% (PP. 25-31),
4. Descriptive statistics with log-transformation of dependent variables (PP. 32-40),
5. Log-general linear multivariate regression with bootstrap-2000 with Confidence Intervals of 95% (PP. 41-47), and
6. Log-general linear multivariate regression analysis (PP. 48-54).

Note:

- Inspector ratio or inspector_ratio, or inspector_rate = Independent Variable (IV),
- Falta injury rate or fatal_rate = Dependent Variable (DV1), and
- Nonfatal injury rate or nonfatal_rate = Dependent Variable (DV2).

Descriptive statistics with original data associated with the research variables (PP. 2-10)

Explore

Notes

Output Created		24-FEB-2026 10:54:35
Comments		
Input	Data	E:\Group 2 - Feb 24\Group 2 - Descriptive and regression analysis.sav
	Active Dataset	DataSet0
	Filter	<none>
	Weight	<none>
	Split File	<none>
	N of Rows in Working Data File	21
Missing Value Handling	Definition of Missing	User-defined missing values for dependent variables are treated as missing.
	Cases Used	Statistics are based on cases with no missing values for any dependent variable or factor used.
Syntax		EXAMINE VARIABLES=Inspector_ratio Fatal_rate Nonfatal_rate<br>/PLOT BOXPLOT HISTOGRAM NPPLOT<br>/COMPARE GROUPS<br>/STATISTICS DESCRIPTIVES EXTREME<br>/CINTERVAL 95<br>/MISSING LISTWISE<br>/NOTOTAL.
Resources	Processor Time	00:00:05.50
	Elapsed Time	00:00:01.92

Case Processing Summary

	Cases					
	Valid		Missing		Total	
	N	Percent	N	Percent	N	Percent
Inspector_ratio	21	100.0%	0	0.0%	21	100.0%
Fatal_rate	21	100.0%	0	0.0%	21	100.0%
Nonfatal_rate	21	100.0%	0	0.0%	21	100.0%

Descriptives

			Statistic	Std. Error
Inspector_ratio	Mean		.644762	.1234034
	95% Confidence Interval for Mean	Lower Bound	.387347	
		Upper Bound	.902177	
	5% Trimmed Mean		.574127	

	Median		.450000	
	Variance		.320	
	Std. Deviation		.5655053	
	Minimum		.1400	
	Maximum		2.4700	
	Range		2.3300	
	Interquartile Range		.6550	
	Skewness		1.987	.501
	Kurtosis		4.461	.972
Fatal_rate	Mean		6.869333	1.3157123
	95% Confidence Interval for Mean	Lower Bound	4.124806	
		Upper Bound	9.613861	
	5% Trimmed Mean		6.252354	
	Median		4.900000	
	Variance		36.353	
	Std. Deviation		6.0293513	
	Minimum		.1400	
	Maximum		25.0000	
	Range		24.8600	
	Interquartile Range		5.4440	
	Skewness		1.769	.501
	Kurtosis		3.221	.972
Nonfatal_rate	Mean		1552.878238	516.4239934
	95% Confidence Interval for Mean	Lower Bound	475.636665	
		Upper Bound	2630.119812	
	5% Trimmed Mean		1213.756749	
	Median		336.350000	
	Variance		5600568.560	
	Std. Deviation		2366.5520405	
	Minimum		9.0000	
	Maximum		9421.3800	
	Range		9412.3800	
	Interquartile Range		2828.2450	
	Skewness		2.162	.501
	Kurtosis		5.271	.972

Extreme Values

			Case Number	Value
Inspector_ratio	Highest	1	16	2.4700
		2	7	1.4100
		3	5	1.3800
		4	2	1.1000
		5	19	1.0800
	Lowest	1	17	.1400
		2	12	.1700
		3	20	.1800
		4	14	.2500
		5	1	.2500
Fatal_rate	Highest	1	10	25.0000
		2	11	17.9300

		3	15	14.5800
		4	21	11.4700
		5	9	9.7200
	Lowest	1	14	.1400
		2	12	1.0000
		3	13	2.3500
		4	4	2.6840
		5	1	3.0150
Nonfatal_rate	Highest	1	9	9421.3800
		2	8	4782.8890
		3	21	4409.1800
		4	15	3229.6900
		5	1	3201.8900
	Lowest	1	12	9.0000
		2	13	18.3200
		3	3	22.0000
		4	14	27.9000
		5	2	29.4000

Tests of Normality

	Kolmogorov-Smirnov[a]			Shapiro-Wilk		
	Statistic	df	Sig.	Statistic	df	Sig.
Inspector_ratio	.260	21	<.001	.774	21	<.001
Fatal_rate	.229	21	.005	.818	21	.001
Nonfatal_rate	.257	21	<.001	.703	21	<.001

a. Lilliefors Significance Correction

Inspector_ratio

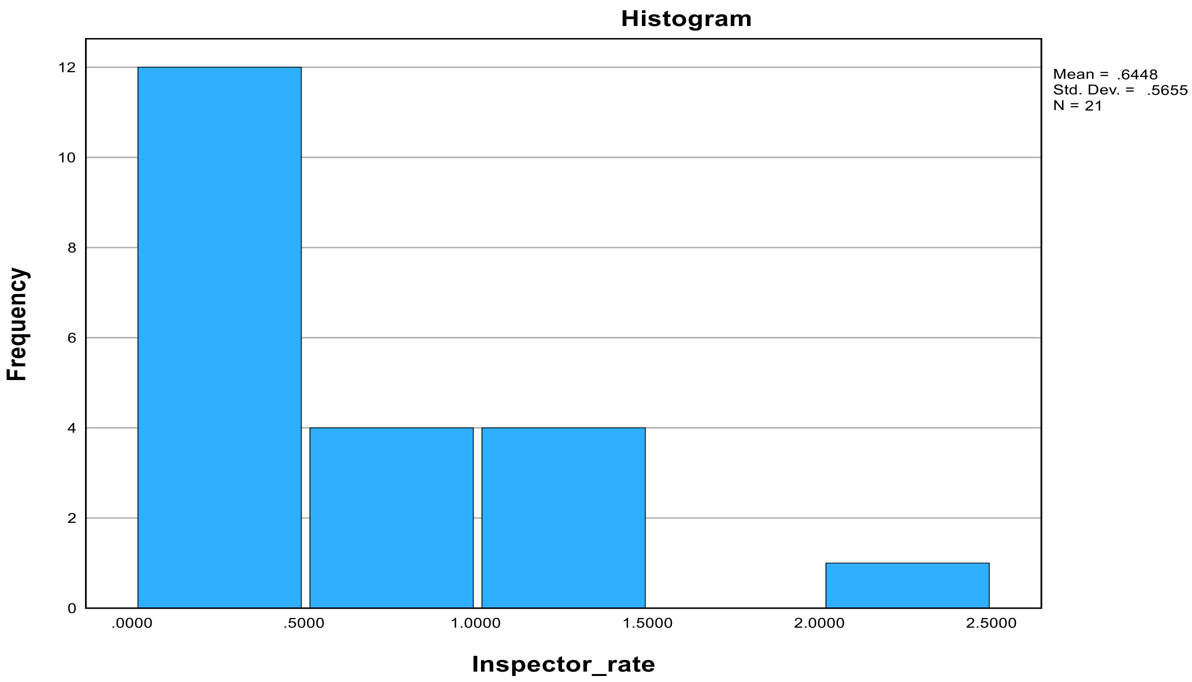

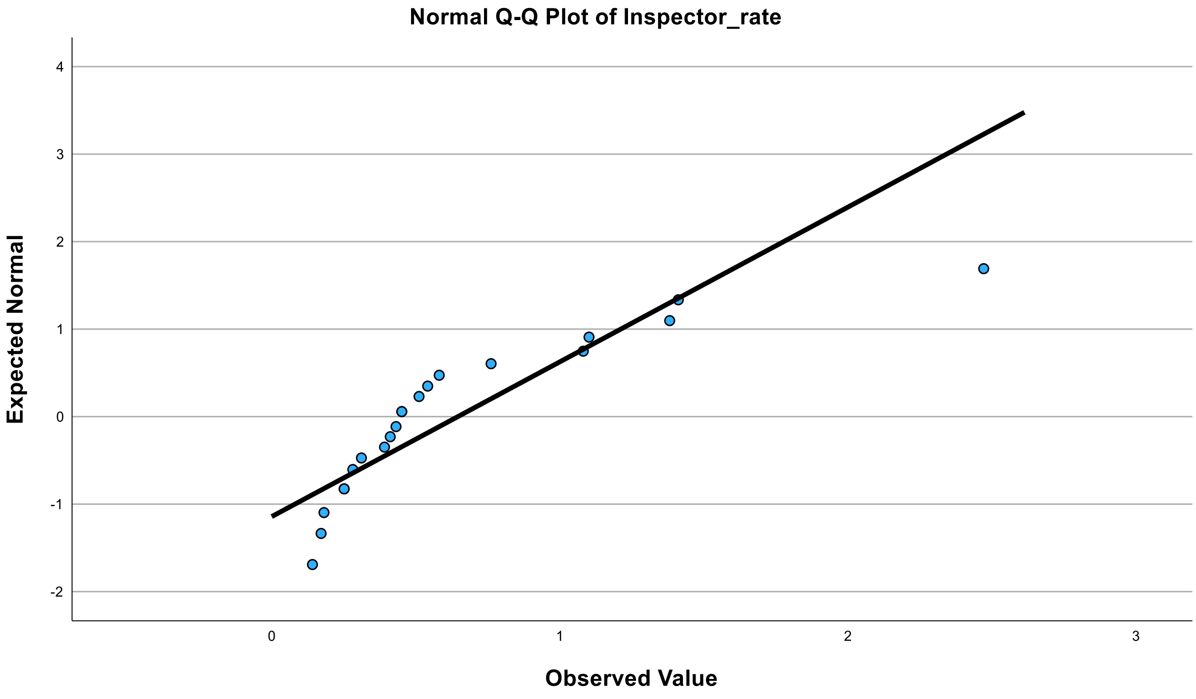

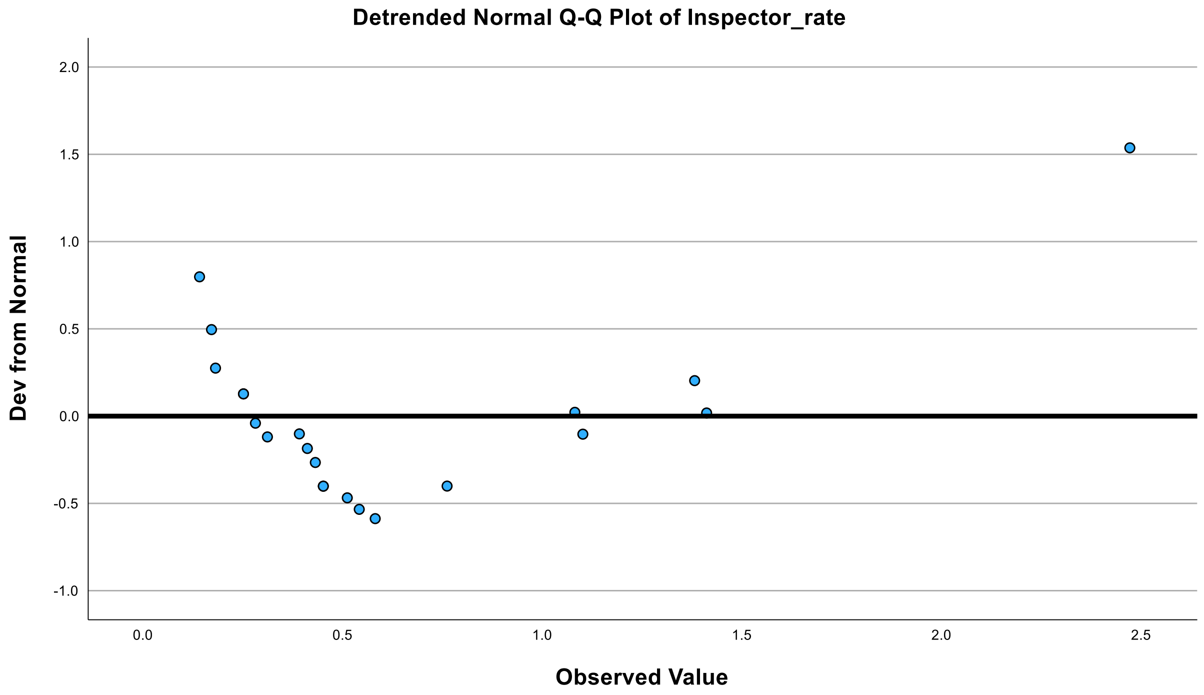
Detrended Normal Q-Q Plot of Inspector_rate
Dev from Normal
2.0
1.5
1.0
0.5
0.0
-0.5
-1.0
0.0
0.5
1.0
1.5
2.0
2.5
Observed Value

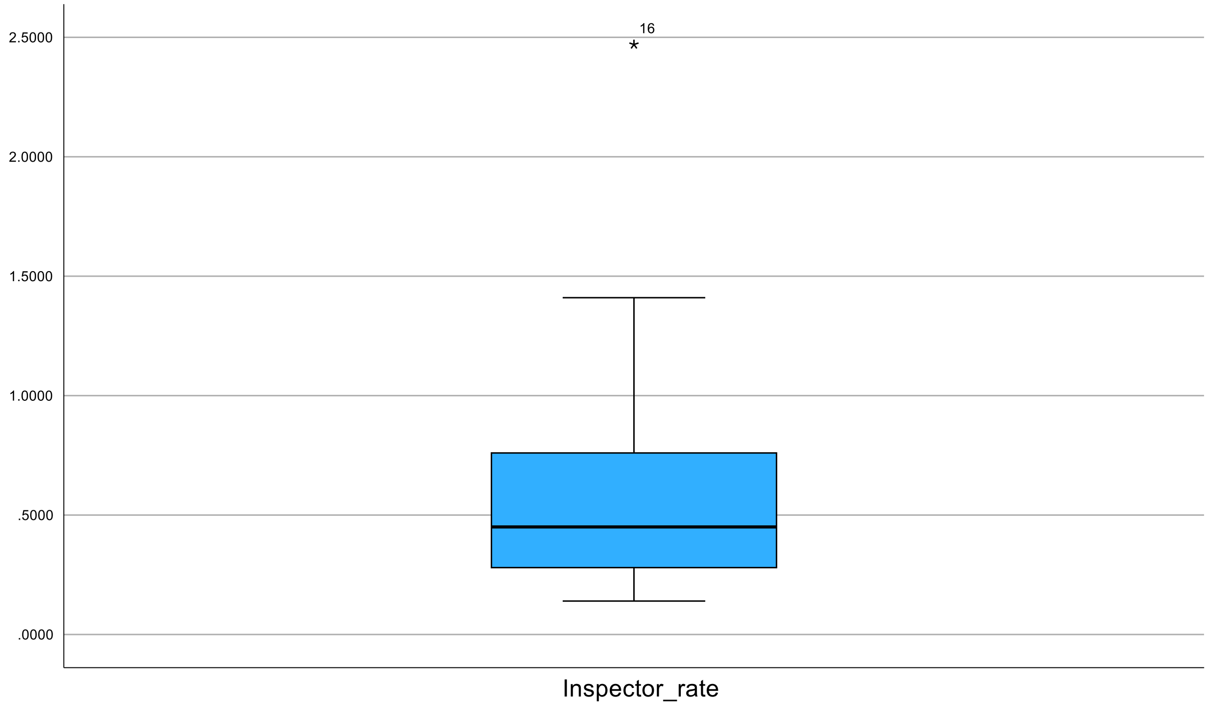
16
2.5000
2.0000
1.5000
1.0000
.5000
.0000
Inspector_rate

Fatal_rate

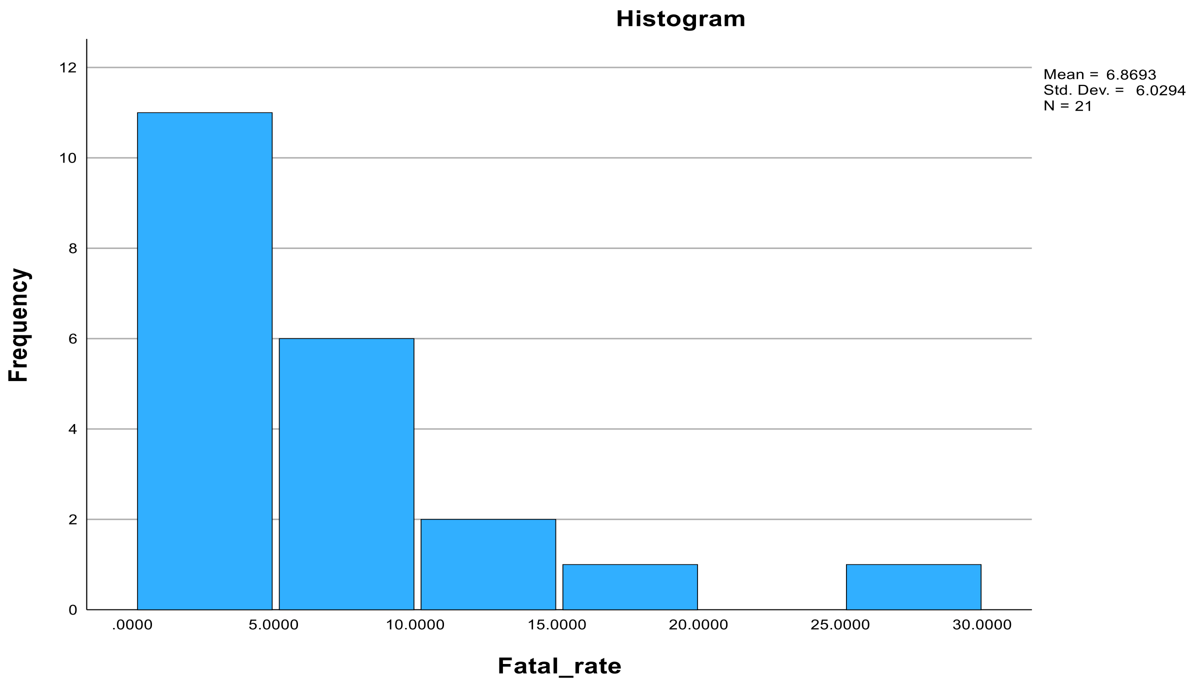
Histogram
Mean = 6.8693
Std. Dev. = 6.0294
N = 21
Frequency
12
10
8
6
4
2
0
.0000
5.0000
10.0000
15.0000
20.0000
25.0000
30.0000
Fatal_rate

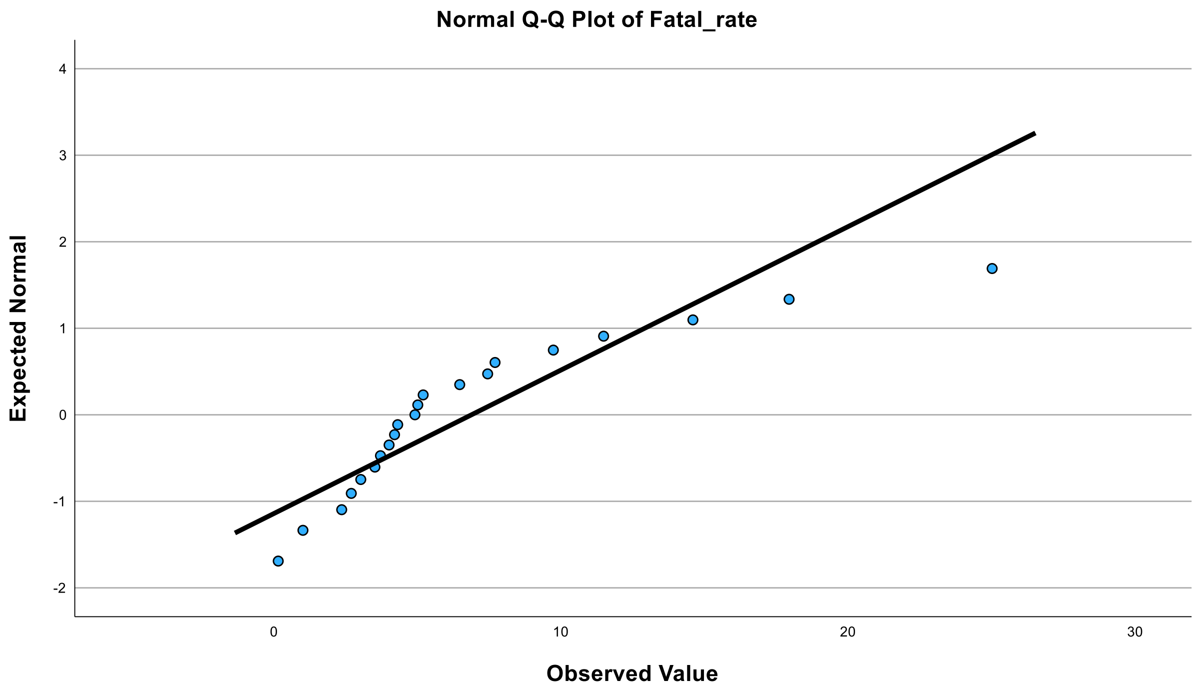
Normal Q-Q Plot of Fatal_rate
Expected Normal
4
3
2
1
0
-1
-2
0
10
20
30
Observed Value

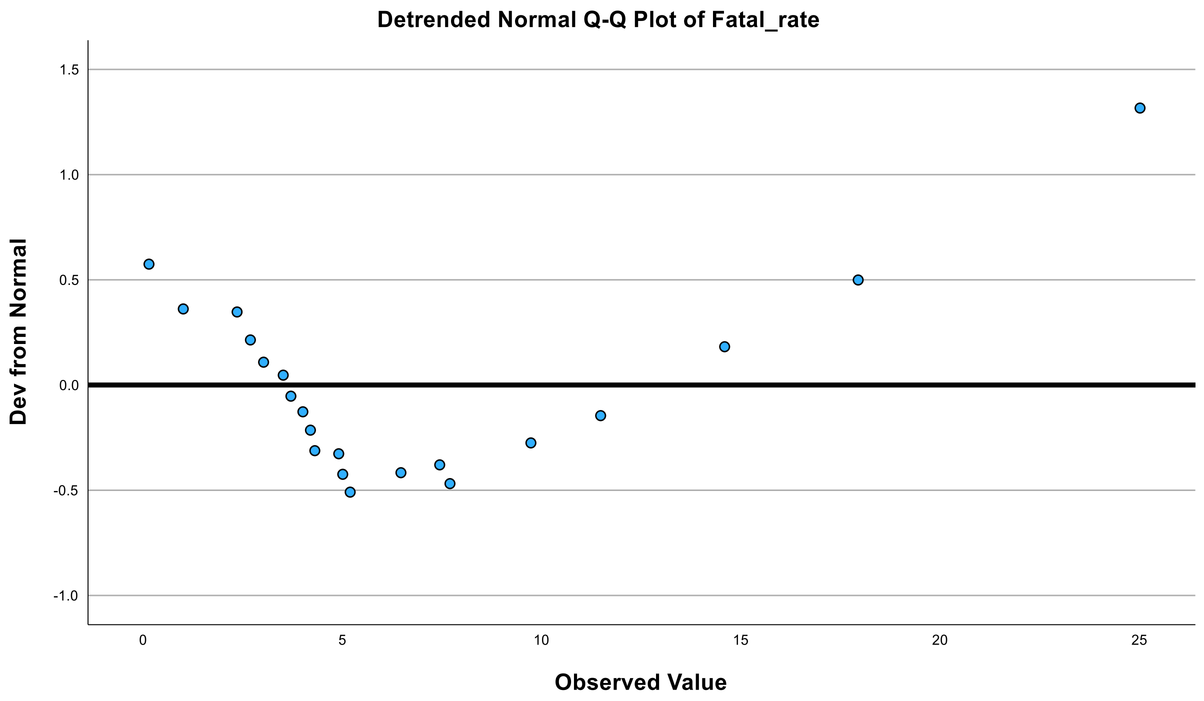

Detrended Normal Q-Q Plot of Fatal_rate
Dev from Normal
1.5
1.0
0.5
0.0
-0.5
-1.0
0
5
10
15
20
25
Observed Value

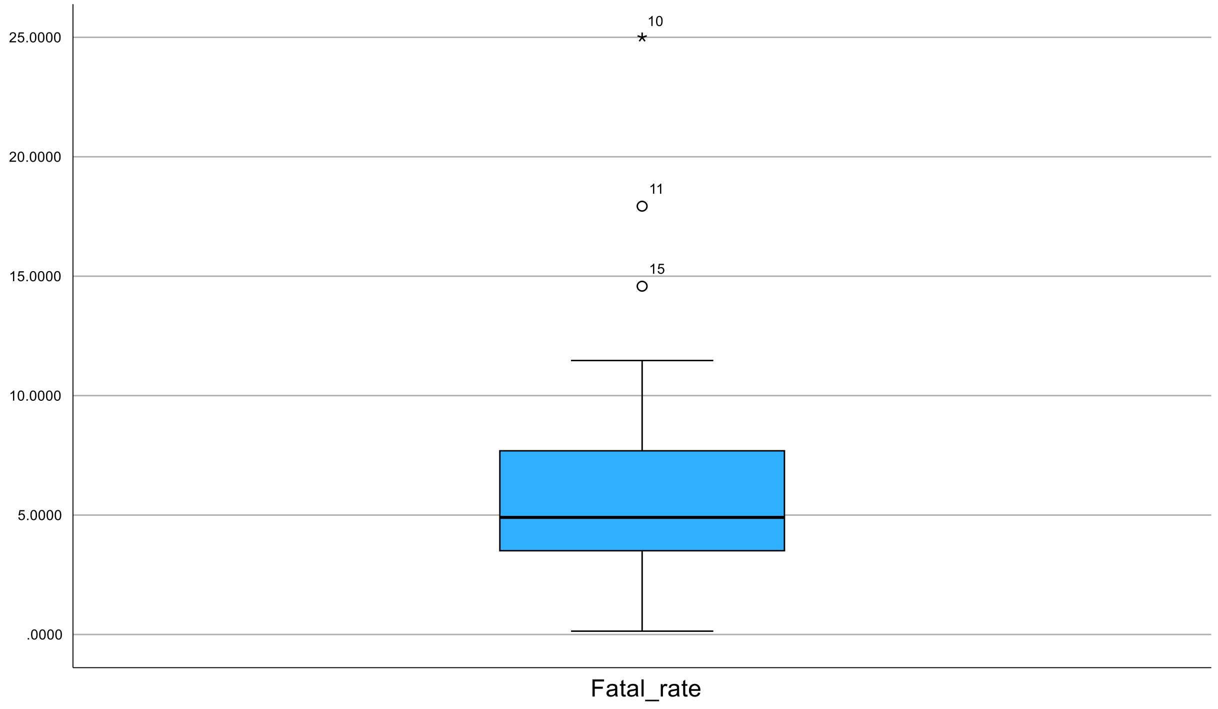

25.0000
20.0000
15.0000
10.0000
5.0000
.0000
10
11
15
Fatal_rate

Nonfatal_rate

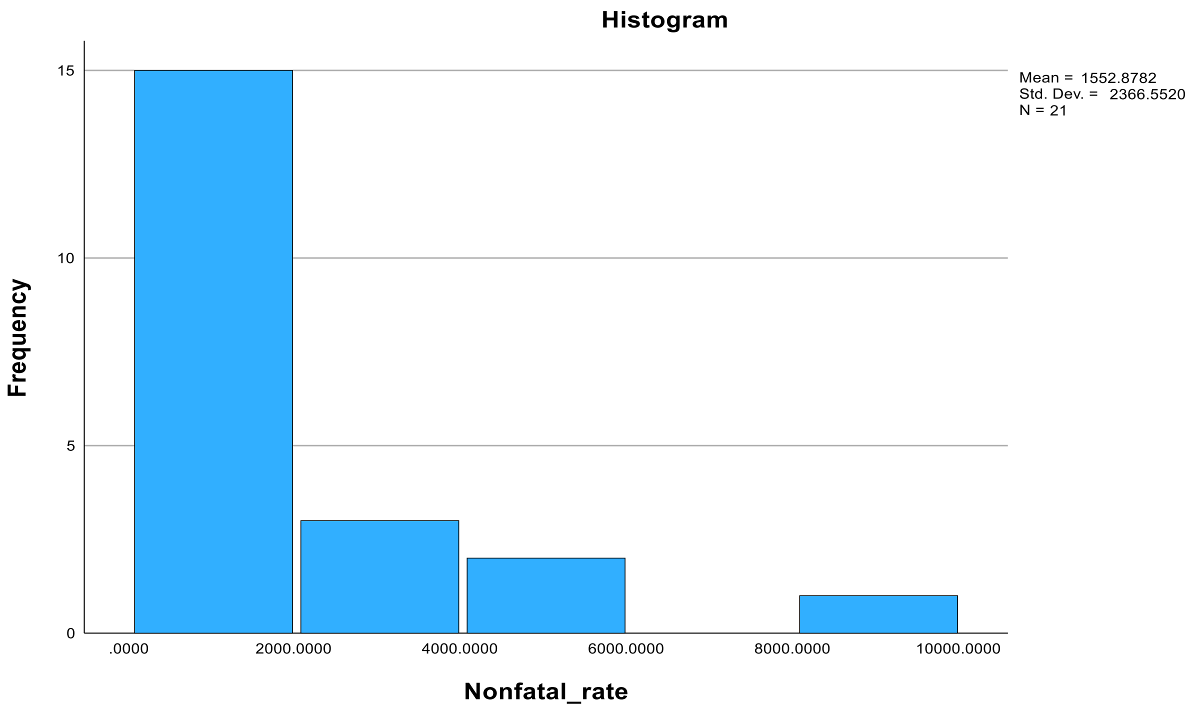

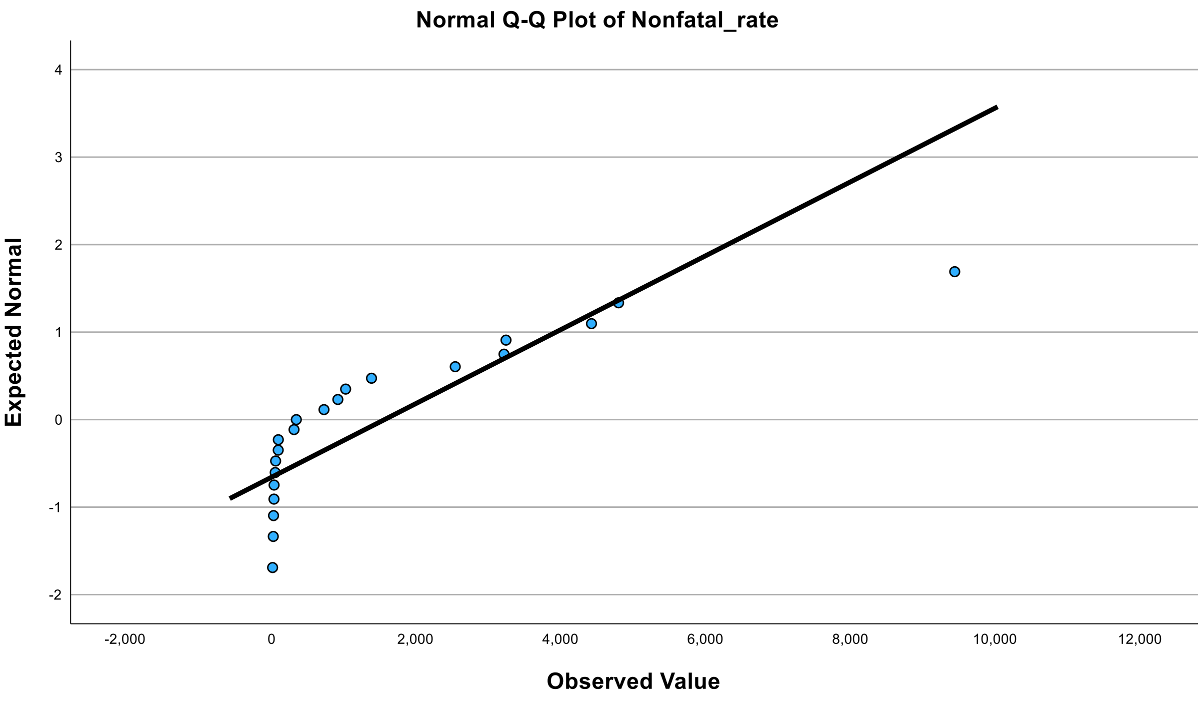

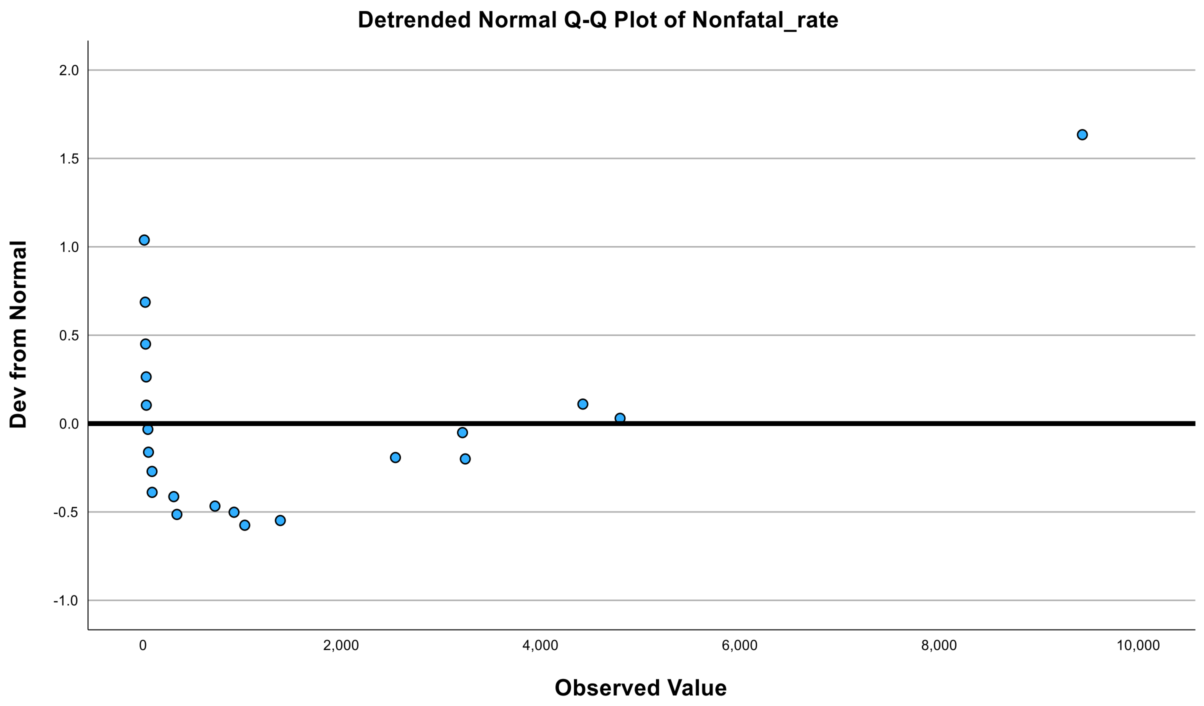
Detrended Normal Q-Q Plot of Nonfatal_rate
Dev from Normal
2.0
1.5
1.0
0.5
0.0
-0.5
-1.0
0
2,000
4,000
6,000
8,000
10,000
Observed Value

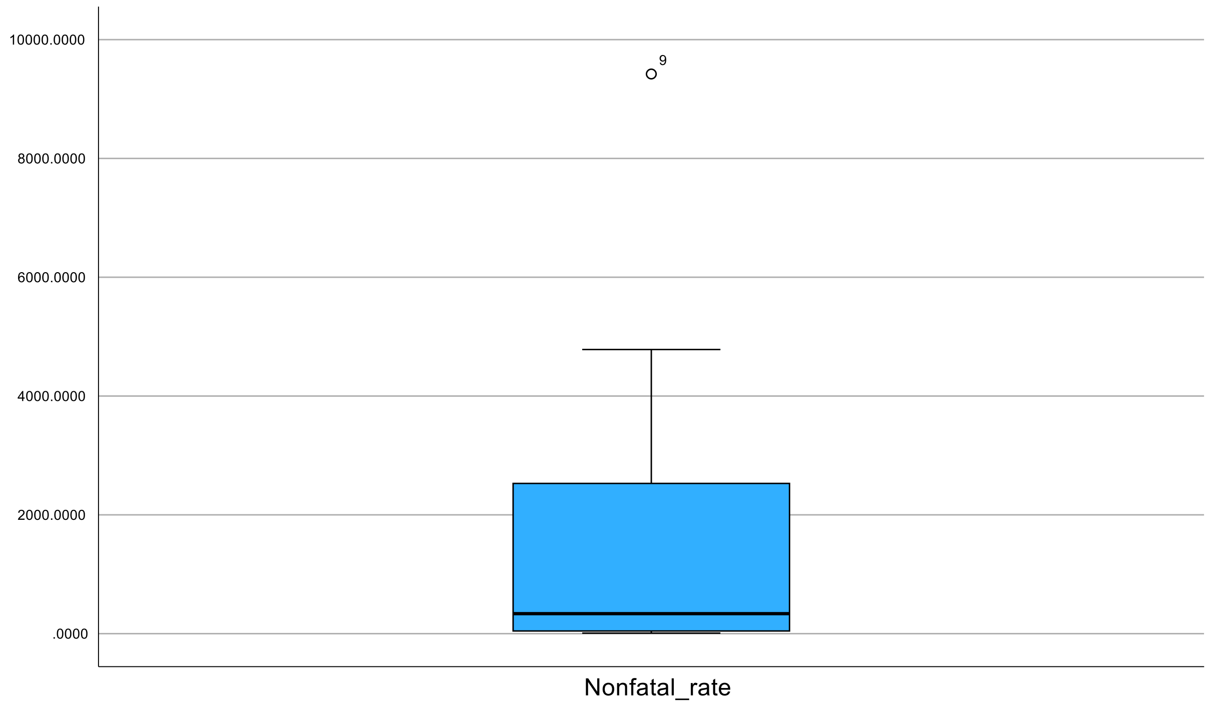
10000.0000
8000.0000
6000.0000
4000.0000
2000.0000
.0000
9
Nonfatal_rate

General linear multivariate regression (with re-runs where applicable) (PP. 11-24)

Notes

Output Created		24-FEB-2026 10:55:49
Comments		
Input	Data	E:\Group 2 - Feb 24\Group 2 - Descriptive and regression analysis.sav
	Active Dataset	DataSet0
	Filter	<none>
	Weight	<none>
	Split File	<none>
	N of Rows in Working Data File	21
Missing Value Handling	Definition of Missing	User-defined missing values are treated as missing.
	Cases Used	Statistics are based on all cases with valid data for all variables in the model.
Syntax		GLM Fatal_rate Nonfatal_rate WITH Inspector_ratio<br>/METHOD=SSTYPE(3)<br>/INTERCEPT=INCLUDE<br>/SAVE=PRED SEPRED RESID ZRESID COOK LEVER<br>/EMMEANS=TABLES(OVERALL) WITH(Inspector_ratio=MEAN)<br>/PRINT=DESCRIPTIVE ETASQ OPOWER PARAMETER TEST(SSCP) RSSCP HOMOGENEITY LOF<br>/PLOT=SPREADLEVEL RESIDUALS<br>/CRITERIA=ALPHA(.05)<br>/DESIGN=Inspector_ratio.
Resources	Processor Time	00:00:00.56
	Elapsed Time	00:00:00.29
Variables Created or Modified	PRE_1	Predicted Value for Fatal_rate
	PRE_2	Predicted Value for Nonfatal_rate
	SEP_1	Standard Error of Predicted Value for Fatal_rate
	SEP_2	Standard Error of Predicted Value for Nonfatal_rate
	RES_1	Residual for Fatal_rate
	RES_2	Residual for Nonfatal_rate
	ZRE_1	Standardized Residual for Fatal_rate
	ZRE_2	Standardized Residual for Nonfatal_rate
	COO_1	Cook's Distance for Fatal_rate
	COO_2	Cook's Distance for Nonfatal_rate
	LEV_1	Uncentered Leverage Value for Fatal_rate
	LEV_2	Uncentered Leverage Value for Nonfatal_rate

Warnings

The HOMOGENEITY specification in the PRINT subcommand will be ignored because there are no between-subjects factors.
The SPREADLEVEL specification has been found in the PLOT subcommand, but the model includes no factors. The spread versus level plot will not be produced.

Descriptive Statistics

	Mean	Std. Deviation	N
Fatal_rate	6.869333	6.0293513	21
Nonfatal_rate	1552.878238	2366.5520405	21

Bartlett's Test of Sphericity[a]

Likelihood Ratio	.000
Approx. Chi-Square	190.652
df	2
Sig.	<.001

Tests the null hypothesis that the residual covariance matrix is proportional to an identity matrix.[a]

a. Design: Intercept + Inspector_ratio

Multivariate Tests[a]

Effect		Value	F	Hypothesis df	Error df	Sig.
Intercept	Pillai's Trace	.453	7.454[b]	2.000	18.000	.004
	Wilks' Lambda	.547	7.454[b]	2.000	18.000	.004
	Hotelling's Trace	.828	7.454[b]	2.000	18.000	.004
	Roy's Largest Root	.828	7.454[b]	2.000	18.000	.004
Inspector_ratio	Pillai's Trace	.044	.412[b]	2.000	18.000	.668
	Wilks' Lambda	.956	.412[b]	2.000	18.000	.668
	Hotelling's Trace	.046	.412[b]	2.000	18.000	.668
	Roy's Largest Root	.046	.412[b]	2.000	18.000	.668

Multivariate Tests[a]

Effect		Partial Eta Squared	Noncent. Parameter	Observed Power[c]
Intercept	Pillai's Trace	.453	14.908	.897
	Wilks' Lambda	.453	14.908	.897
	Hotelling's Trace	.453	14.908	.897
	Roy's Largest Root	.453	14.908	.897
Inspector_ratio	Pillai's Trace	.044	.825	.107
	Wilks' Lambda	.044	.825	.107
	Hotelling's Trace	.044	.825	.107
	Roy's Largest Root	.044	.825	.107

a. Design: Intercept + Inspector_ratio

b. Exact statistic

c. Computed using alpha = .05

Tests of Between-Subjects Effects

Source	Dependent Variable	Type III Sum of Squares	df	Mean Square	F
Corrected Model	Fatal_rate	3.428[a]	1	3.428	.090
	Nonfatal_rate	4891988.212[b]	1	4891988.212	.868
Intercept	Fatal_rate	478.579	1	478.579	12.566
	Nonfatal_rate	39787164.498	1	39787164.498	7.057
Inspector_ratio	Fatal_rate	3.428	1	3.428	.090
	Nonfatal_rate	4891988.212	1	4891988.212	.868
Error	Fatal_rate	723.633	19	38.086	
	Nonfatal_rate	107119382.993	19	5637862.263	
Total	Fatal_rate	1718.004	21		
	Nonfatal_rate	162651418.474	21		
Corrected Total	Fatal_rate	727.062	20		
	Nonfatal_rate	112011371.205	20		

Tests of Between-Subjects Effects

Source	Dependent Variable	Sig.	Partial Eta Squared	Noncent. Parameter	Observed Power[c]
Corrected Model	Fatal_rate	.767	.005	.090	.059
	Nonfatal_rate	.363	.044	.868	.143
Intercept	Fatal_rate	.002	.398	12.566	.919
	Nonfatal_rate	.016	.271	7.057	.712
Inspector_ratio	Fatal_rate	.767	.005	.090	.059
	Nonfatal_rate	.363	.044	.868	.143
Error	Fatal_rate				
	Nonfatal_rate				
Total	Fatal_rate				
	Nonfatal_rate				
Corrected Total	Fatal_rate				
	Nonfatal_rate				

a. R Squared = .005 (Adjusted R Squared = -.048)
b. R Squared = .044 (Adjusted R Squared = -.007)
c. Computed using alpha = .05

Parameter Estimates

Dependent Variable	Parameter	B	Std. Error	t	Sig.	95% Confidence Interval Lower Bound
Fatal_rate	Intercept	7.341	2.071	3.545	.002	3.007
	Inspector_ratio	-.732	2.440	-.300	.767	-5.840
Nonfatal_rate	Intercept	2116.763	796.816	2.657	.016	449.008
	Inspector_ratio	-874.563	938.870	-.932	.363	-2839.641

Parameter Estimates

Dependent Variable	Parameter	95% Confidence Interval Upper Bound	Partial Eta Squared	Noncent. Parameter	Observed Power[a]
Fatal_rate	Intercept	11.676	.398	3.545	.919
	Inspector_ratio	4.375	.005	.300	.059
Nonfatal_rate	Intercept	3784.518	.271	2.657	.712
	Inspector_ratio	1090.515	.044	.932	.143

a. Computed using alpha = .05

Between-Subjects SSCP Matrix

			Fatal_rate	Nonfatal_rate
Hypothesis	Intercept	Fatal_rate	478.579	137990.270
		Nonfatal_rate	137990.270	39787164.498
	Inspector_ratio	Fatal_rate	3.428	4095.249
		Nonfatal_rate	4095.249	4891988.212
Error		Fatal_rate	723.633	73809.080
		Nonfatal_rate	73809.080	107119382.993

Based on Type III Sum of Squares

Residual SSCP Matrix

		Fatal_rate	Nonfatal_rate
Sum-of-Squares and Cross-Products	Fatal_rate	723.633	73809.080
	Nonfatal_rate	73809.080	107119382.993
Covariance	Fatal_rate	38.086	3884.688
	Nonfatal_rate	3884.688	5637862.263
Correlation	Fatal_rate	1.000	.265
	Nonfatal_rate	.265	1.000

Based on Type III Sum of Squares

Lack of Fit

Multivariate Tests

Dependent Variables		Value	F	Hypothesis df	Error df
Fatal_rate, Nonfatal_rate	Pillai's Trace	1.953	4.842	34.000	4.000
	Wilks' Lambda	.000	41.333[a]	34.000	2.000
	Hotelling's Trace	23488.983	.000	34.000	.000
	Roy's Largest Root	23468.887	2761.045[b]	17.000	2.000
Fatal_rate	Pillai's Trace	.994	20.260[a]	17.000	2.000
	Wilks' Lambda	.006	20.260[a]	17.000	2.000
	Hotelling's Trace	172.209	20.260[a]	17.000	2.000
	Roy's Largest Root	172.209	20.260[a]	17.000	2.000
Nonfatal_rate	Pillai's Trace	.953	2.383[a]	17.000	2.000
	Wilks' Lambda	.047	2.383[a]	17.000	2.000
	Hotelling's Trace	20.257	2.383[a]	17.000	2.000
	Roy's Largest Root	20.257	2.383[a]	17.000	2.000

Multivariate Tests

Dependent Variables		Sig.	Partial Eta Squared	Noncent. Parameter
Fatal_rate, Nonfatal_rate	Pillai's Trace	.067	.976	164.622
	Wilks' Lambda	.024	.999	1405.318
	Hotelling's Trace	.	1.000	.000
	Roy's Largest Root	<.001	1.000	46937.773
Fatal_rate	Pillai's Trace	.048	.994	344.417
	Wilks' Lambda	.048	.994	344.417
	Hotelling's Trace	.048	.994	344.417
	Roy's Largest Root	.048	.994	344.417
Nonfatal_rate	Pillai's Trace	.336	.953	40.514
	Wilks' Lambda	.336	.953	40.514
	Hotelling's Trace	.336	.953	40.514
	Roy's Largest Root	.336	.953	40.514

Multivariate Tests

Dependent Variables		Observed Power[c]
Fatal_rate, Nonfatal_rate	Pillai's Trace	.599
	Wilks' Lambda	.886
	Hotelling's Trace	.
	Roy's Largest Root	1.000
Fatal_rate	Pillai's Trace	.663
	Wilks' Lambda	.663
	Hotelling's Trace	.663
	Roy's Largest Root	.663
Nonfatal_rate	Pillai's Trace	.159
	Wilks' Lambda	.159
	Hotelling's Trace	.159
	Roy's Largest Root	.159

a. Exact statistic

b. The statistic is an upper bound on F that yields a lower bound on the significance level.

c. Computed using alpha = .05

Univariate Tests

Dependent Variable	Source	Sum of Squares	df	Mean Square	F	Sig.
Fatal_rate	Lack of Fit	719.455	17	42.321	20.260	.048
	Pure Error	4.178	2	2.089		
Nonfatal_rate	Lack of Fit	102080111.913	17	6004712.465	2.383	.336
	Pure Error	5039271.080	2	2519635.540		

Univariate Tests

Dependent Variable	Source	Partial Eta Squared	Noncent. Parameter	Observed Power[a]
Fatal_rate	Lack of Fit	.994	344.417	.663
	Pure Error			
Nonfatal_rate	Lack of Fit	.953	40.514	.159
	Pure Error			

a. Computed using alpha = .05

SSCP Matrix

		Fatal_rate	Nonfatal_rate
Lack of Fit	Fatal_rate	719.455	69236.599
	Nonfatal_rate	69236.599	102080111.913
Pure Error	Fatal_rate	4.178	4572.481
	Nonfatal_rate	4572.481	5039271.080

Estimated Marginal Means

Grand Mean

Dependent Variable	Mean	Std. Error	95% Confidence Interval Lower Bound	95% Confidence Interval Upper Bound
Fatal_rate	6.869[a]	1.347	4.051	9.688
Nonfatal_rate	1552.878[a]	518.141	468.398	2637.359

a. Covariates appearing in the model are evaluated at the following values: Inspector_ratio = .644762.

Observed * Predicted * Std. Residual Plots

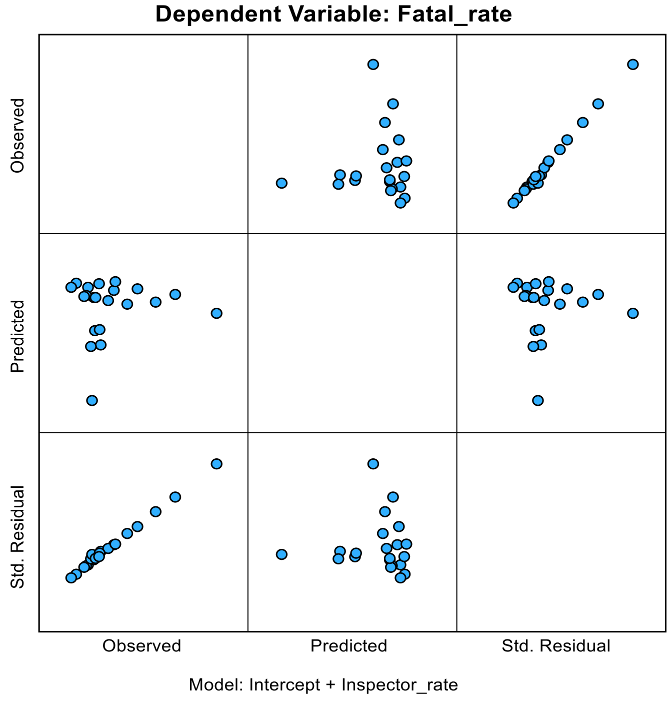

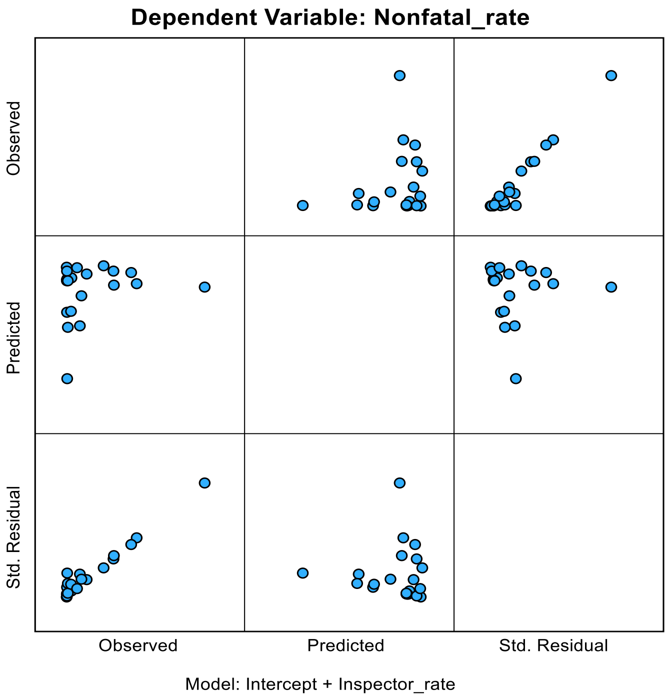
Dependent Variable: Nonfatal_rate
Observed
Predicted
Std. Residual
Observed
Predicted
Std. Residual
Model: Intercept + Inspector_rate

Re-run General Linear Model with reduced data

Notes

Output Created		24-FEB-2026 10:57:09
Comments		
Input	Data	E:\Group 2 - Feb 24\Group 2 - Descriptive and regression analysis.sav
	Active Dataset	DataSet0
	Filter	<none>
	Weight	<none>
	Split File	<none>
	N of Rows in Working Data File	20
Missing Value Handling	Definition of Missing	User-defined missing values are treated as missing.
	Cases Used	Statistics are based on all cases with valid data for all variables in the model.
Syntax		GLM Fatal_rate Nonfatal_rate WITH Inspector_ratio<br>/METHOD=SSTYPE(3)<br>/INTERCEPT=INCLUDE<br>/SAVE=PRED SEPRED RESID ZRESID COOK LEVER<br>/PRINT=DESCRIPTIVE ETASQ OPOWER PARAMETER TEST(SSCP) RSSCP HOMOGENEITY LOF<br>/PLOT=SPREADLEVEL RESIDUALS<br>/CRITERIA=ALPHA(.05)<br>/DESIGN=Inspector_ratio.
Resources	Processor Time	00:00:00.47
	Elapsed Time	00:00:00.29
Variables Created or Modified	PRE_3	Predicted Value for Fatal_rate
	PRE_4	Predicted Value for Nonfatal_rate
	SEP_3	Standard Error of Predicted Value for Fatal_rate
	SEP_4	Standard Error of Predicted Value for Nonfatal_rate
	RES_3	Residual for Fatal_rate
	RES_4	Residual for Nonfatal_rate
	ZRE_3	Standardized Residual for Fatal_rate
	ZRE_4	Standardized Residual for Nonfatal_rate
	COO_3	Cook's Distance for Fatal_rate
	COO_4	Cook's Distance for Nonfatal_rate
	LEV_3	Uncentered Leverage Value for Fatal_rate
	LEV_4	Uncentered Leverage Value for Nonfatal_rate

Warnings

The HOMOGENEITY specification in the PRINT subcommand will be ignored because there are no between-subjects factors.
The SPREADLEVEL specification has been found in the PLOT subcommand, but the model includes no factors. The spread versus level plot will not be produced.

Descriptive Statistics

	Mean	Std. Deviation	N
Fatal_rate	6.726800	6.1495782	20
Nonfatal_rate	1159.453150	1572.8349401	20

Bartlett's Test of Sphericity[a]

Likelihood Ratio	.000
Approx. Chi-Square	164.989
df	2
Sig.	<.001

Tests the null hypothesis that the residual covariance matrix is proportional to an identity matrix.[a]
a. Design: Intercept + Inspector_ratio

Multivariate Tests[a]

Effect		Value	F	Hypothesis df	Error df	Sig.
Intercept	Pillai's Trace	.479	7.817[b]	2.000	17.000	.004
	Wilks' Lambda	.521	7.817[b]	2.000	17.000	.004
	Hotelling's Trace	.920	7.817[b]	2.000	17.000	.004
	Roy's Largest Root	.920	7.817[b]	2.000	17.000	.004
Inspector_ratio	Pillai's Trace	.086	.796[b]	2.000	17.000	.467
	Wilks' Lambda	.914	.796[b]	2.000	17.000	.467
	Hotelling's Trace	.094	.796[b]	2.000	17.000	.467
	Roy's Largest Root	.094	.796[b]	2.000	17.000	.467

Multivariate Tests[a]

Effect		Partial Eta Squared	Noncent. Parameter	Observed Power[c]
Intercept	Pillai's Trace	.479	15.634	.908
	Wilks' Lambda	.479	15.634	.908
	Hotelling's Trace	.479	15.634	.908
	Roy's Largest Root	.479	15.634	.908
Inspector_ratio	Pillai's Trace	.086	1.592	.163
	Wilks' Lambda	.086	1.592	.163
	Hotelling's Trace	.086	1.592	.163
	Roy's Largest Root	.086	1.592	.163

a. Design: Intercept + Inspector_ratio
b. Exact statistic
c. Computed using alpha = .05

Tests of Between-Subjects Effects

Source	Dependent Variable	Type III Sum of Squares	df	Mean Square	F
Corrected Model	Fatal_rate	3.152[a]	1	3.152	.079
	Nonfatal_rate	4003621.739[b]	1	4003621.739	1.676
Intercept	Fatal_rate	445.816	1	445.816	11.217
	Nonfatal_rate	24172040.333	1	24172040.333	10.119
Inspector_ratio	Fatal_rate	3.152	1	3.152	.079
	Nonfatal_rate	4003621.739	1	4003621.739	1.676
Error	Fatal_rate	715.376	18	39.743	
	Nonfatal_rate	42998763.490	18	2388820.194	
Total	Fatal_rate	1623.526	20		
	Nonfatal_rate	73889017.370	20		
Corrected Total	Fatal_rate	718.529	19		
	Nonfatal_rate	47002385.229	19		

Tests of Between-Subjects Effects

Source	Dependent Variable	Sig.	Partial Eta Squared	Noncent. Parameter	Observed Power[c]
Corrected Model	Fatal_rate	.781	.004	.079	.058
	Nonfatal_rate	.212	.085	1.676	.232
Intercept	Fatal_rate	.004	.384	11.217	.886
	Nonfatal_rate	.005	.360	10.119	.853
Inspector_ratio	Fatal_rate	.781	.004	.079	.058
	Nonfatal_rate	.212	.085	1.676	.232
Error	Fatal_rate				
	Nonfatal_rate				
Total	Fatal_rate				
	Nonfatal_rate				
Corrected Total	Fatal_rate				
	Nonfatal_rate				

a. R Squared = .004 (Adjusted R Squared = -.051)
b. R Squared = .085 (Adjusted R Squared = .034)
c. Computed using alpha = .05

Parameter Estimates

Dependent Variable	Parameter	B	Std. Error	t	Sig.	95% Confidence Interval Lower Bound
Fatal_rate	Intercept	7.182	2.144	3.349	.004	2.677
	Inspector_ratio	-.702	2.494	-.282	.781	-5.941
Nonfatal_rate	Intercept	1672.314	525.718	3.181	.005	567.821
	Inspector_ratio	-791.452	611.350	-1.295	.212	-2075.850

Parameter Estimates

Dependent Variable	Parameter	95% Confidence Interval Upper Bound	Partial Eta Squared	Noncent. Parameter	Observed Power[a]
Fatal_rate	Intercept	11.687	.384	3.349	.886
	Inspector_ratio	4.537	.004	.282	.058
Nonfatal_rate	Intercept	2776.806	.360	3.181	.853
	Inspector_ratio	492.946	.085	1.295	.232

a. Computed using alpha = .05

Between-Subjects SSCP Matrix

			Fatal_rate	Nonfatal_rate
Hypothesis	Intercept	Fatal_rate	445.816	103808.848
		Nonfatal_rate	103808.848	24172040.333
	Inspector_ratio	Fatal_rate	3.152	3552.651
		Nonfatal_rate	3552.651	4003621.739
Error		Fatal_rate	715.376	50799.678
		Nonfatal_rate	50799.678	42998763.490

Based on Type III Sum of Squares

Residual SSCP Matrix

		Fatal_rate	Nonfatal_rate
Sum-of-Squares and Cross-Products	Fatal_rate	715.376	50799.678
	Nonfatal_rate	50799.678	42998763.490
Covariance	Fatal_rate	39.743	2822.204
	Nonfatal_rate	2822.204	2388820.194
Correlation	Fatal_rate	1.000	.290
	Nonfatal_rate	.290	1.000

Based on Type III Sum of Squares

Lack of Fit

Multivariate Tests

Dependent Variables		Value	F	Hypothesis df	Error df
Fatal_rate, Nonfatal_rate	Pillai's Trace	1.882	1.997	32.000	4.000
	Wilks' Lambda	.000	27.438[a]	32.000	2.000
	Hotelling's Trace	22808.312	.000	32.000	.000
	Roy's Largest Root	22800.821	2850.103[b]	16.000	2.000
Fatal_rate	Pillai's Trace	.994	21.279[a]	16.000	2.000
	Wilks' Lambda	.006	21.279[a]	16.000	2.000
	Hotelling's Trace	170.232	21.279[a]	16.000	2.000
	Roy's Largest Root	170.232	21.279[a]	16.000	2.000
Nonfatal_rate	Pillai's Trace	.883	.942[a]	16.000	2.000
	Wilks' Lambda	.117	.942[a]	16.000	2.000
	Hotelling's Trace	7.533	.942[a]	16.000	2.000
	Roy's Largest Root	7.533	.942[a]	16.000	2.000

Multivariate Tests

Dependent Variables		Sig.	Partial Eta Squared	Noncent. Parameter
Fatal_rate, Nonfatal_rate	Pillai's Trace	.265	.941	63.902
	Wilks' Lambda	.036	.998	878.017
	Hotelling's Trace	.	1.000	.000
	Roy's Largest Root	<.001	1.000	45601.641
Fatal_rate	Pillai's Trace	.046	.994	340.465
	Wilks' Lambda	.046	.994	340.465
	Hotelling's Trace	.046	.994	340.465
	Roy's Largest Root	.046	.994	340.465
Nonfatal_rate	Pillai's Trace	.631	.883	15.065
	Wilks' Lambda	.631	.883	15.065
	Hotelling's Trace	.631	.883	15.065
	Roy's Largest Root	.631	.883	15.065

Multivariate Tests

Dependent Variables		Observed Power[c]
Fatal_rate, Nonfatal_rate	Pillai's Trace	.280
	Wilks' Lambda	.767
	Hotelling's Trace	.
	Roy's Largest Root	1.000
Fatal_rate	Pillai's Trace	.680
	Wilks' Lambda	.680
	Hotelling's Trace	.680
	Roy's Largest Root	.680
Nonfatal_rate	Pillai's Trace	.095
	Wilks' Lambda	.095
	Hotelling's Trace	.095
	Roy's Largest Root	.095

a. Exact statistic
b. The statistic is an upper bound on F that yields a lower bound on the significance level.
c. Computed using alpha = .05

Univariate Tests

Dependent Variable	Source	Sum of Squares	df	Mean Square	F	Sig.
Fatal_rate	Lack of Fit	711.199	16	44.450	21.279	.046
	Pure Error	4.178	2	2.089		
Nonfatal_rate	Lack of Fit	37959492.410	16	2372468.276	.942	.631
	Pure Error	5039271.080	2	2519635.540		

Univariate Tests

Dependent Variable	Source	Partial Eta Squared	Noncent. Parameter	Observed Power[a]
Fatal_rate	Lack of Fit	.994	340.465	.680
	Pure Error			
Nonfatal_rate	Lack of Fit	.883	15.065	.095
	Pure Error			

a. Computed using alpha = .05

SSCP Matrix

		Fatal_rate	Nonfatal_rate
Lack of Fit	Fatal_rate	711.199	46227.197
	Nonfatal_rate	46227.197	37959492.410
Pure Error	Fatal_rate	4.178	4572.481
	Nonfatal_rate	4572.481	5039271.080

Observed * Predicted * Std. Residual Plots

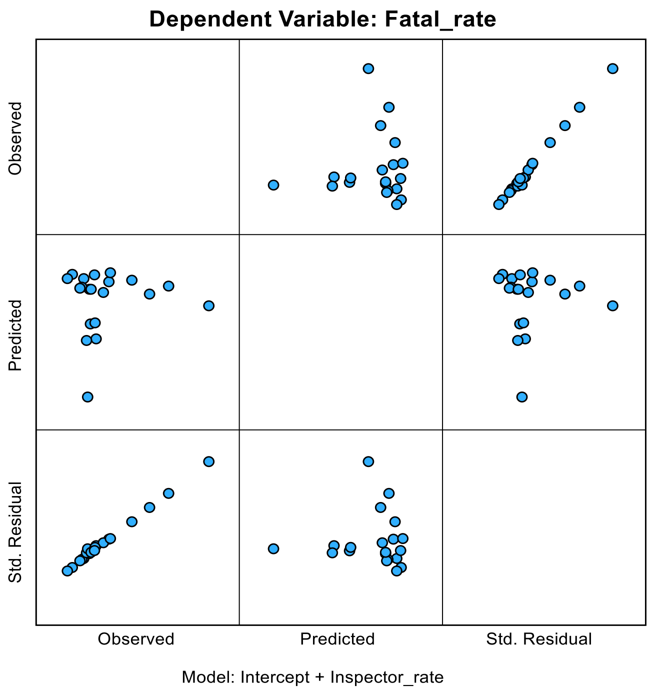

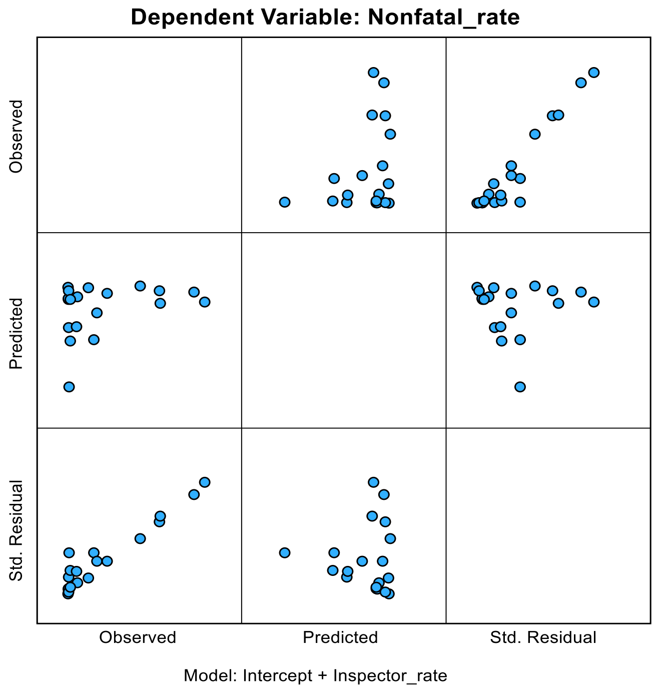

Dependent Variable: Nonfatal_rate
Observed
Predicted
Std. Residual
Observed
Predicted
Std. Residual
Model: Intercept + Inspector_rate

Continued.

General linear multivariate regression with bootstrap-2000 with Confidence Intervals of 95% with reduced data (PP. 25-31)

Notes

Output Created		24-FEB-2026 10:58:25
Comments		
Input	Data	E:\Group 2 - Feb 24\Group 2 - Descriptive and regression analysis.sav
	Active Dataset	DataSet0
	Filter	<none>
	Weight	<none>
	Split File	<none>
	N of Rows in Working Data File	20
Syntax		BOOTSTRAP<br>/SAMPLING METHOD=SIMPLE<br>/VARIABLES TARGET=Nonfatal_rate Fatal_rate INPUT= Inspector_ratio<br>/CRITERIA CILEVEL=95 CITYPE=PERCENTILE NSAMPLES=2000<br>/MISSING USERMISSING=EXCLUDE.
Resources	Processor Time	00:00:00.02
	Elapsed Time	00:00:00.02

Bootstrap Specifications

Sampling Method	Simple
Number of Samples	2000
Confidence Interval Level	95.0%
Confidence Interval Type	Percentile

General Linear Model

Notes

Output Created		24-FEB-2026 10:58:25
Comments		
Input	Data	E:\Group 2 - Feb 24\Group 2 - Descriptive and regression analysis.sav
	Active Dataset	DataSet0
	Filter	<none>
	Weight	<none>
	Split File	<none>
	N of Rows in Working Data File	40020
Missing Value Handling	Definition of Missing	User-defined missing values are treated as missing.
	Cases Used	Statistics are based on all cases with valid data for all variables in the model.

Syntax		GLM Nonfatal_rate Fatal_rate WITH Inspector_ratio<br>/METHOD=SSTYPE(3)<br>/INTERCEPT=INCLUDE<br>/SAVE=PRED SEPRED RESID ZRESID COOK LEVER<br>/EMMEANS=TABLES(OVERALL) WITH(Inspector_ratio=MEAN)<br>/PRINT=DESCRIPTIVE ETASQ OPOWER PARAMETER TEST(SSCP) RSSCP HOMOGENEITY LOF<br>/PLOT=SPREADLEVEL RESIDUALS<br>/CRITERIA=ALPHA(.05)<br>/DESIGN=Inspector_ratio.
Resources	Processor Time	00:00:28.50
	Elapsed Time	00:00:34.57

Warnings

The HOMOGENEITY specification in the PRINT subcommand will be ignored because there are no between-subjects factors.
The SPREADLEVEL specification has been found in the PLOT subcommand, but the model includes no factors. The spread versus level plot will not be produced.
This command is trying to save new variables while bootstrapping is in effect. The new variables will be lost.<br>Execution of this command stops.

Descriptive Statistics

	Mean	Std. Deviation	N
Nonfatal_rate	1159.453150	1572.8349401	20
Fatal_rate	6.726800	6.1495782	20

Bartlett's Test of Sphericity[a]

Likelihood Ratio	.000
Approx. Chi-Square	164.989
df	2
Sig.	<.001

Tests the null hypothesis that the residual covariance matrix is proportional to an identity matrix.[a]

a. Design: Intercept + Inspector_ratio

Multivariate Tests[a]

Effect		Value	F	Hypothesis df	Error df	Sig.
Intercept	Pillai's Trace	.479	7.817[b]	2.000	17.000	.004
	Wilks' Lambda	.521	7.817[b]	2.000	17.000	.004
	Hotelling's Trace	.920	7.817[b]	2.000	17.000	.004
	Roy's Largest Root	.920	7.817[b]	2.000	17.000	.004

Inspector_ratio	Pillai's Trace	.086	.796[b]	2.000	17.000	.467
	Wilks' Lambda	.914	.796[b]	2.000	17.000	.467
	Hotelling's Trace	.094	.796[b]	2.000	17.000	.467
	Roy's Largest Root	.094	.796[b]	2.000	17.000	.467

Multivariate Tests[a]

Effect		Partial Eta Squared	Noncent. Parameter	Observed Power[c]
Intercept	Pillai's Trace	.479	15.634	.908
	Wilks' Lambda	.479	15.634	.908
	Hotelling's Trace	.479	15.634	.908
	Roy's Largest Root	.479	15.634	.908
Inspector_ratio	Pillai's Trace	.086	1.592	.163
	Wilks' Lambda	.086	1.592	.163
	Hotelling's Trace	.086	1.592	.163
	Roy's Largest Root	.086	1.592	.163

a. Design: Intercept + Inspector_ratio
b. Exact statistic
c. Computed using alpha = .05

Tests of Between-Subjects Effects

Source	Dependent Variable	Type III Sum of Squares	df	Mean Square	F
Corrected Model	Nonfatal_rate	4003621.739[a]	1	4003621.739	1.676
	Fatal_rate	3.152[b]	1	3.152	.079
Intercept	Nonfatal_rate	24172040.333	1	24172040.333	10.119
	Fatal_rate	445.816	1	445.816	11.217
Inspector_ratio	Nonfatal_rate	4003621.739	1	4003621.739	1.676
	Fatal_rate	3.152	1	3.152	.079
Error	Nonfatal_rate	42998763.490	18	2388820.194	
	Fatal_rate	715.376	18	39.743	
Total	Nonfatal_rate	73889017.370	20		
	Fatal_rate	1623.526	20		
Corrected Total	Nonfatal_rate	47002385.229	19		
	Fatal_rate	718.529	19		

Tests of Between-Subjects Effects

Source	Dependent Variable	Sig.	Partial Eta Squared	Noncent. Parameter	Observed Power[c]
Corrected Model	Nonfatal_rate	.212	.085	1.676	.232
	Fatal_rate	.781	.004	.079	.058
Intercept	Nonfatal_rate	.005	.360	10.119	.853
	Fatal_rate	.004	.384	11.217	.886
Inspector_ratio	Nonfatal_rate	.212	.085	1.676	.232
	Fatal_rate	.781	.004	.079	.058
Error	Nonfatal_rate				
	Fatal_rate				
Total	Nonfatal_rate				
	Fatal_rate				
Corrected Total	Nonfatal_rate				
	Fatal_rate				

a. R Squared = .085 (Adjusted R Squared = .034)
b. R Squared = .004 (Adjusted R Squared = -.051)
c. Computed using alpha = .05

Parameter Estimates

Dependent Variable	Parameter	B	Std. Error	t	Sig.	95% Confidence Interval Lower Bound
Nonfatal_rate	Intercept	1672.314	525.718	3.181	.005	567.821
	Inspector_ratio	-791.452	611.350	-1.295	.212	-2075.850
Fatal_rate	Intercept	7.182	2.144	3.349	.004	2.677
	Inspector_ratio	-.702	2.494	-.282	.781	-5.941

Parameter Estimates

Dependent Variable	Parameter	95% Confidence Interval Upper Bound	Partial Eta Squared	Noncent. Parameter	Observed Power[a]
Nonfatal_rate	Intercept	2776.806	.360	3.181	.853
	Inspector_ratio	492.946	.085	1.295	.232
Fatal_rate	Intercept	11.687	.384	3.349	.886
	Inspector_ratio	4.537	.004	.282	.058

a. Computed using alpha = .05

Bootstrap for Parameter Estimates

Dependent Variable	Parameter	B	Bootstrap[a] Bias	Bootstrap[a] Std. Error	Bootstrap[a] Sig. (2-tailed)	Bootstrap[a] 95% Confidence Interval Lower
Nonfatal_rate	Intercept	1672.314	30.927	528.615	.018	737.453
	Inspector_ratio	-791.452	-48.617	441.473	.055	-1847.645
Fatal_rate	Intercept	7.182	-.179	1.851	.018	3.551
	Inspector_ratio	-.702	.512	2.255	.609	-2.946

Bootstrap for Parameter Estimates

Dependent Variable	Parameter	Bootstrap 95% Confidence Interval Upper
Nonfatal_rate	Intercept	2849.792
	Inspector_ratio	-117.451
Fatal_rate	Intercept	10.946
	Inspector_ratio	5.751

a. Unless otherwise noted, bootstrap results are based on 2000 bootstrap samples

Between-Subjects SSCP Matrix

			Nonfatal_rate	Fatal_rate
Hypothesis	Intercept	Nonfatal_rate	24172040.333	103808.848
		Fatal_rate	103808.848	445.816
	Inspector_ratio	Nonfatal_rate	4003621.739	3552.651
		Fatal_rate	3552.651	3.152
Error		Nonfatal_rate	42998763.490	50799.678
		Fatal_rate	50799.678	715.376

Based on Type III Sum of Squares

Residual SSCP Matrix

		Nonfatal_rate	Fatal_rate
Sum-of-Squares and Cross-Products	Nonfatal_rate	42998763.490	50799.678
	Fatal_rate	50799.678	715.376
Covariance	Nonfatal_rate	2388820.194	2822.204
	Fatal_rate	2822.204	39.743
Correlation	Nonfatal_rate	1.000	.290
	Fatal_rate	.290	1.000

Based on Type III Sum of Squares

Lack of Fit

Multivariate Tests

Dependent Variables		Value	F	Hypothesis df	Error df
Nonfatal_rate, Fatal_rate	Pillai's Trace	1.882	1.997	32.000	4.000
	Wilks' Lambda	.000	27.438[a]	32.000	2.000
	Hotelling's Trace	22808.312	.000	32.000	.000
	Roy's Largest Root	22800.821	2850.103[b]	16.000	2.000
Nonfatal_rate	Pillai's Trace	.883	.942[a]	16.000	2.000
	Wilks' Lambda	.117	.942[a]	16.000	2.000
	Hotelling's Trace	7.533	.942[a]	16.000	2.000
	Roy's Largest Root	7.533	.942[a]	16.000	2.000
Fatal_rate	Pillai's Trace	.994	21.279[a]	16.000	2.000
	Wilks' Lambda	.006	21.279[a]	16.000	2.000
	Hotelling's Trace	170.232	21.279[a]	16.000	2.000
	Roy's Largest Root	170.232	21.279[a]	16.000	2.000

Multivariate Tests

Dependent Variables		Sig.	Partial Eta Squared	Noncent. Parameter
Nonfatal_rate, Fatal_rate	Pillai's Trace	.265	.941	63.902
	Wilks' Lambda	.036	.998	878.017
	Hotelling's Trace	.	1.000	.000
	Roy's Largest Root	<.001	1.000	45601.641
Nonfatal_rate	Pillai's Trace	.631	.883	15.065
	Wilks' Lambda	.631	.883	15.065
	Hotelling's Trace	.631	.883	15.065
	Roy's Largest Root	.631	.883	15.065
Fatal_rate	Pillai's Trace	.046	.994	340.465
	Wilks' Lambda	.046	.994	340.465
	Hotelling's Trace	.046	.994	340.465
	Roy's Largest Root	.046	.994	340.465

Multivariate Tests

Dependent Variables		Observed Power[c]
Nonfatal_rate, Fatal_rate	Pillai's Trace	.280
	Wilks' Lambda	.767
	Hotelling's Trace	.
	Roy's Largest Root	1.000
Nonfatal_rate	Pillai's Trace	.095
	Wilks' Lambda	.095
	Hotelling's Trace	.095
	Roy's Largest Root	.095
Fatal_rate	Pillai's Trace	.680
	Wilks' Lambda	.680
	Hotelling's Trace	.680
	Roy's Largest Root	.680

a. Exact statistic

b. The statistic is an upper bound on F that yields a lower bound on the significance level.

c. Computed using alpha = .05

Univariate Tests

Dependent Variable	Source	Sum of Squares	df	Mean Square	F	Sig.
Nonfatal_rate	Lack of Fit	37959492.410	16	2372468.276	.942	.631
	Pure Error	5039271.080	2	2519635.540		
Fatal_rate	Lack of Fit	711.199	16	44.450	21.279	.046
	Pure Error	4.178	2	2.089		

Univariate Tests

Dependent Variable	Source	Partial Eta Squared	Noncent. Parameter	Observed Power[a]
Nonfatal_rate	Lack of Fit	.883	15.065	.095
	Pure Error			
Fatal_rate	Lack of Fit	.994	340.465	.680
	Pure Error			

a. Computed using alpha = .05

SSCP Matrix

		Nonfatal_rate	Fatal_rate
Lack of Fit	Nonfatal_rate	37959492.410	46227.197
	Fatal_rate	46227.197	711.199
Pure Error	Nonfatal_rate	5039271.080	4572.481
	Fatal_rate	4572.481	4.178

Estimated Marginal Means

Grand Mean

			95% Confidence Interval	
Dependent Variable	Mean	Std. Error	Lower Bound	Upper Bound
Nonfatal_rate	1159.453[a]	345.602	433.369	1885.537
Fatal_rate	6.727[a]	1.410	3.765	9.688

a. Covariates appearing in the model are evaluated at the following values: Inspector_ratio = .648000.

Continued.

Descriptive statistics with log-transformation of dependent variables with reduced data (PP. 32-40)

Explore

Notes

Output Created		24-FEB-2026 11:00:27
Comments		
Input	Data	E:\Group 2 - Feb 24\Group 2 - Descriptive and regression analysis.sav
	Active Dataset	DataSet0
	Filter	<none>
	Weight	<none>
	Split File	<none>
	N of Rows in Working Data File	20
Missing Value Handling	Definition of Missing	User-defined missing values for dependent variables are treated as missing.
	Cases Used	Statistics are based on cases with no missing values for any dependent variable or factor used.
Syntax		EXAMINE VARIABLES=Inspector_ratio Log_fatalrate Log_nonfatalrate<br>/PLOT BOXPLOT HISTOGRAM NPPLOT<br>/COMPARE GROUPS<br>/STATISTICS DESCRIPTIVES EXTREME<br>/CINTERVAL 95<br>/MISSING LISTWISE<br>/NOTOTAL.
Resources	Processor Time	00:00:01.47
	Elapsed Time	00:00:01.29

Case Processing Summary

	Cases					
	Valid		Missing		Total	
	N	Percent	N	Percent	N	Percent
Inspector_ratio	20	100.0%	0	0.0%	20	100.0%
Log_fatalrate	20	100.0%	0	0.0%	20	100.0%
Log_nonfatalrate	20	100.0%	0	0.0%	20	100.0%

Descriptives

			Statistic	Std. Error
Inspector_ratio	Mean		.648000	.1296911
	95% Confidence Interval for Mean	Lower Bound	.376553	
		Upper Bound	.919447	
	5% Trimmed Mean		.575000	
	Median		.440000	
	Variance		.336	
	Std. Deviation		.5799964	
	Minimum		.1400	

	Maximum		2.4700	
	Range		2.3300	
	Interquartile Range		.7425	
	Skewness		1.930	.512
	Kurtosis		4.110	.992
Log_fatalrate	Mean		.6471	.10705
	95% Confidence Interval for Mean	Lower Bound	.4231	
		Upper Bound	.8712	
	5% Trimmed Mean		.6888	
	Median		.6618	
	Variance		.229	
	Std. Deviation		.47875	
	Minimum		-.85	
	Maximum		1.40	
	Range		2.25	
	Interquartile Range		.39	
	Skewness		-1.496	.512
	Kurtosis		4.464	.992
Log_nonfatalrate	Mean		2.4213	.20412
	95% Confidence Interval for Mean	Lower Bound	1.9941	
		Upper Bound	2.8486	
	5% Trimmed Mean		2.4329	
	Median		2.5052	
	Variance		.833	
	Std. Deviation		.91285	
	Minimum		.95	
	Maximum		3.68	
	Range		2.73	
	Interquartile Range		1.82	
	Skewness		-.051	.512
	Kurtosis		-1.543	.992

Extreme Values

			Case Number	Value
Inspector_ratio	Highest	1	15	2.4700
		2	7	1.4100
		3	5	1.3800
		4	2	1.1000
		5	18	1.0800
	Lowest	1	16	.1400
		2	11	.1700
		3	19	.1800
		4	13	.2500
		5	1	.2500
Log_fatalrate	Highest	1	9	1.40
		2	10	1.25
		3	14	1.16
		4	20	1.06
		5	16	.89
	Lowest	1	13	-.85

		2	11	.00
		3	12	.37
		4	4	.43
		5	1	.48
Log_nonfatalrate	Highest	1	8	3.68
		2	20	3.64
		3	14	3.51
		4	1	3.51
		5	16	3.40
	Lowest	1	11	.95
		2	12	1.26
		3	3	1.34
		4	13	1.45
		5	2	1.47

Tests of Normality

	Kolmogorov-Smirnov[a]			Shapiro-Wilk		
	Statistic	df	Sig.	Statistic	df	Sig.
Inspector_ratio	.274	20	<.001	.774	20	<.001
Log_fatalrate	.182	20	.081	.875	20	.014
Log_nonfatalrate	.150	20	.200*	.916	20	.082

*. This is a lower bound of the true significance.

a. Lilliefors Significance Correction

Inspector_ratio

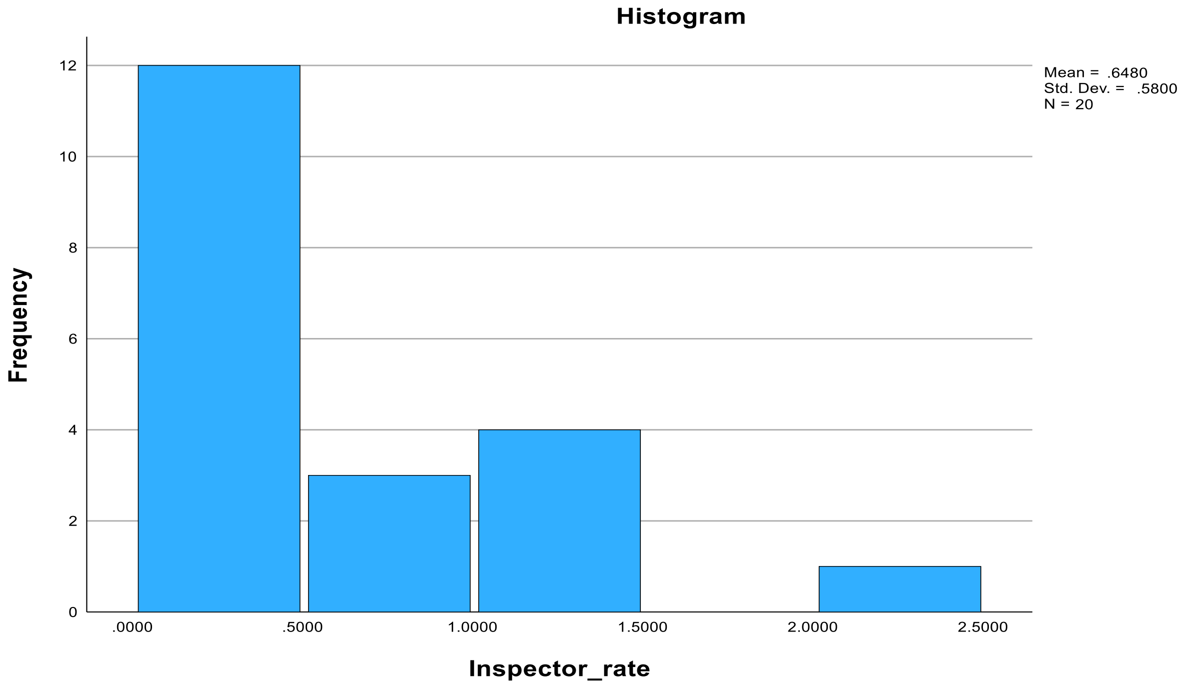

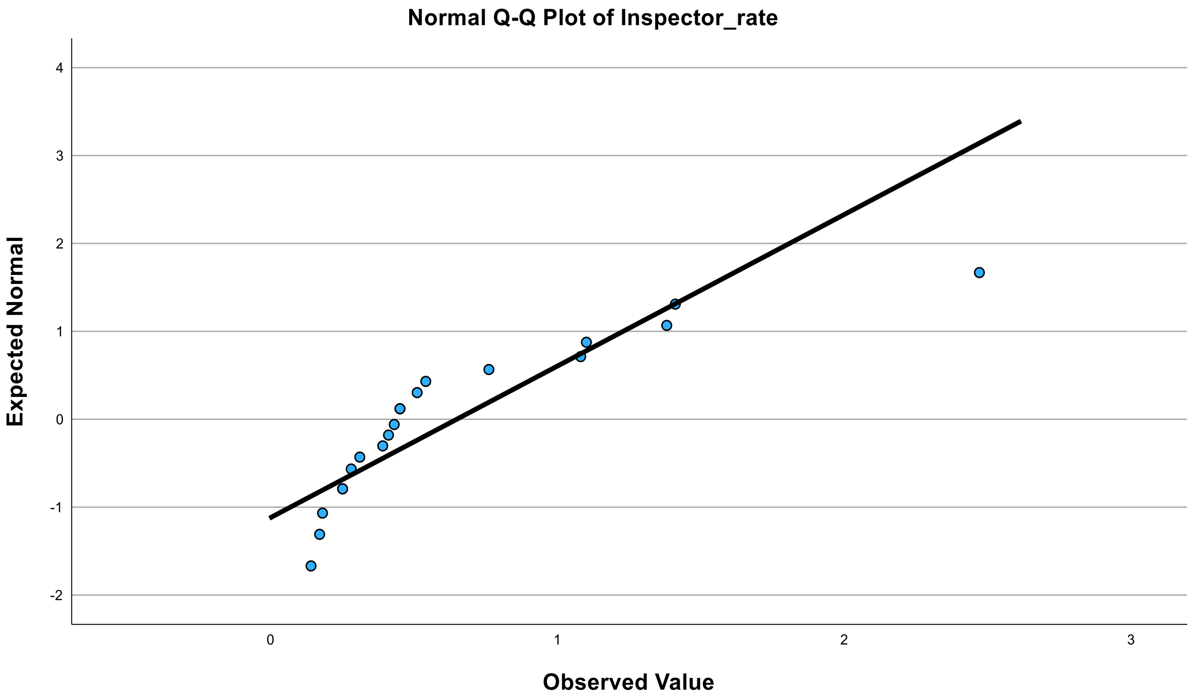
Normal Q-Q Plot of Inspector_rate
Expected Normal
4
3
2
1
0
-1
-2
0
1
2
3
Observed Value

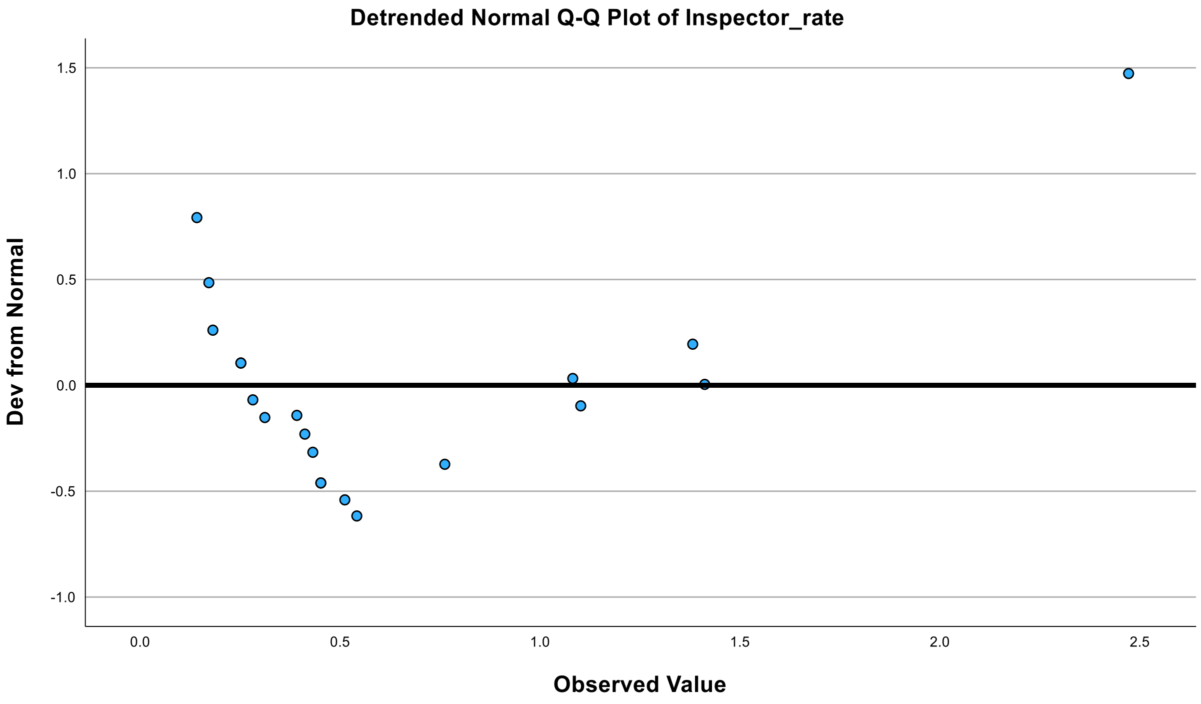
Detrended Normal Q-Q Plot of Inspector_rate
Dev from Normal
1.5
1.0
0.5
0.0
-0.5
-1.0
0.0
0.5
1.0
1.5
2.0
2.5
Observed Value

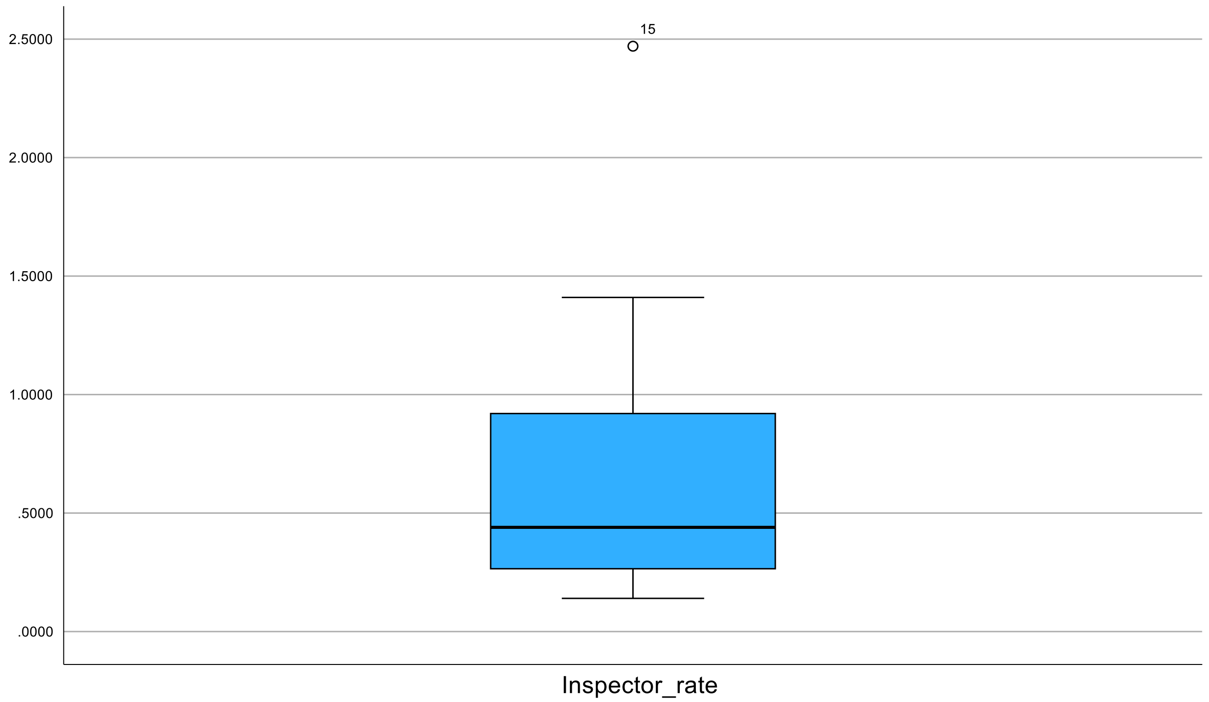

Log_fatalrate

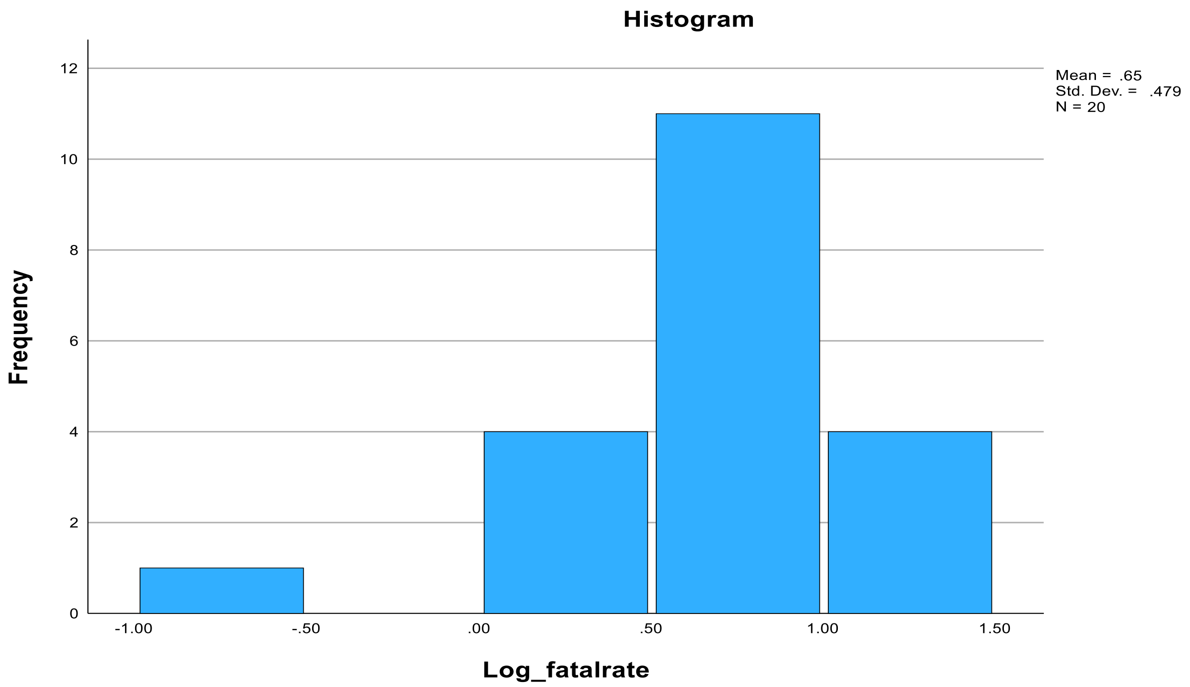

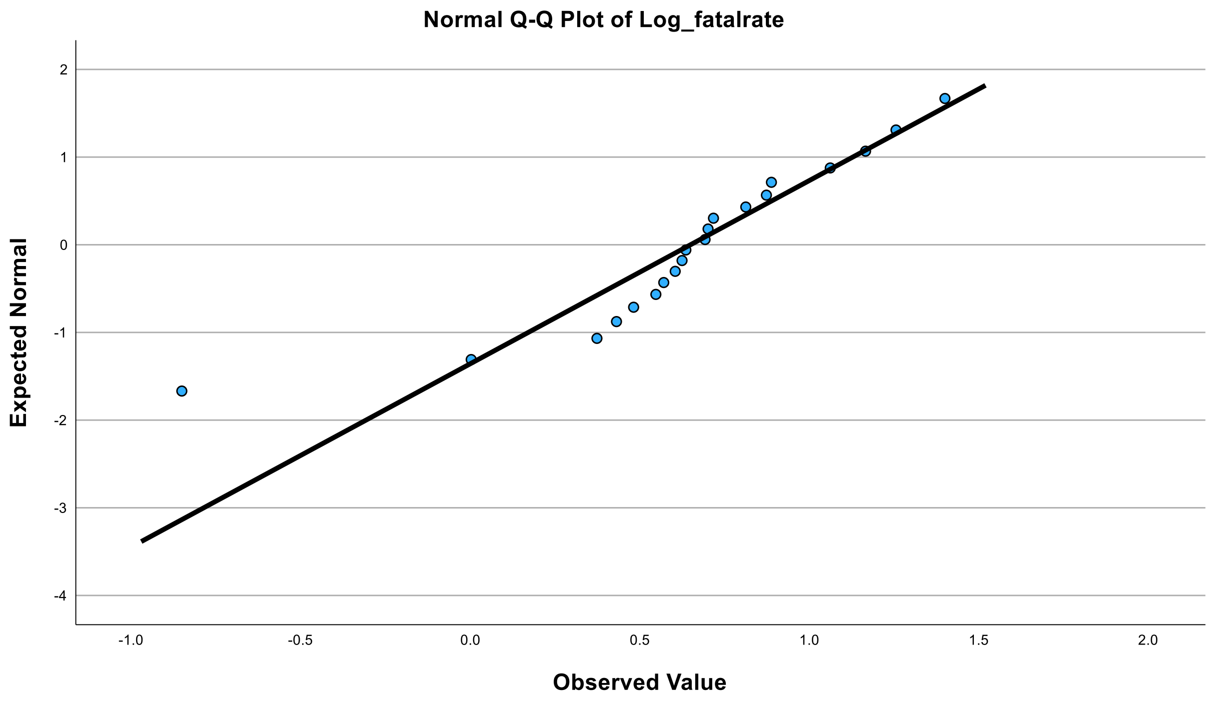
Normal Q-Q Plot of Log_fatalrate
Expected Normal
Observed Value
2
1
0
-1
-2
-3
-4
-1.0
-0.5
0.0
0.5
1.0
1.5
2.0

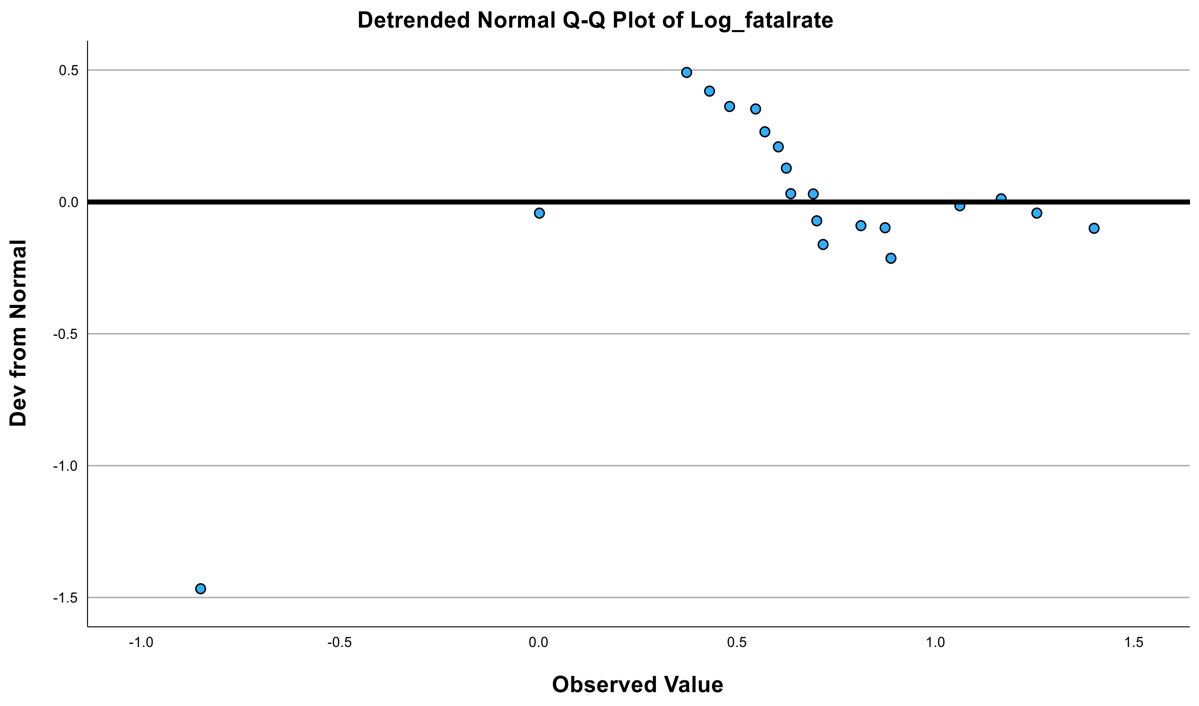
Detrended Normal Q-Q Plot of Log_fatalrate
Dev from Normal
Observed Value
0.5
0.0
-0.5
-1.0
-1.5
-1.0
-0.5
0.0
0.5
1.0
1.5

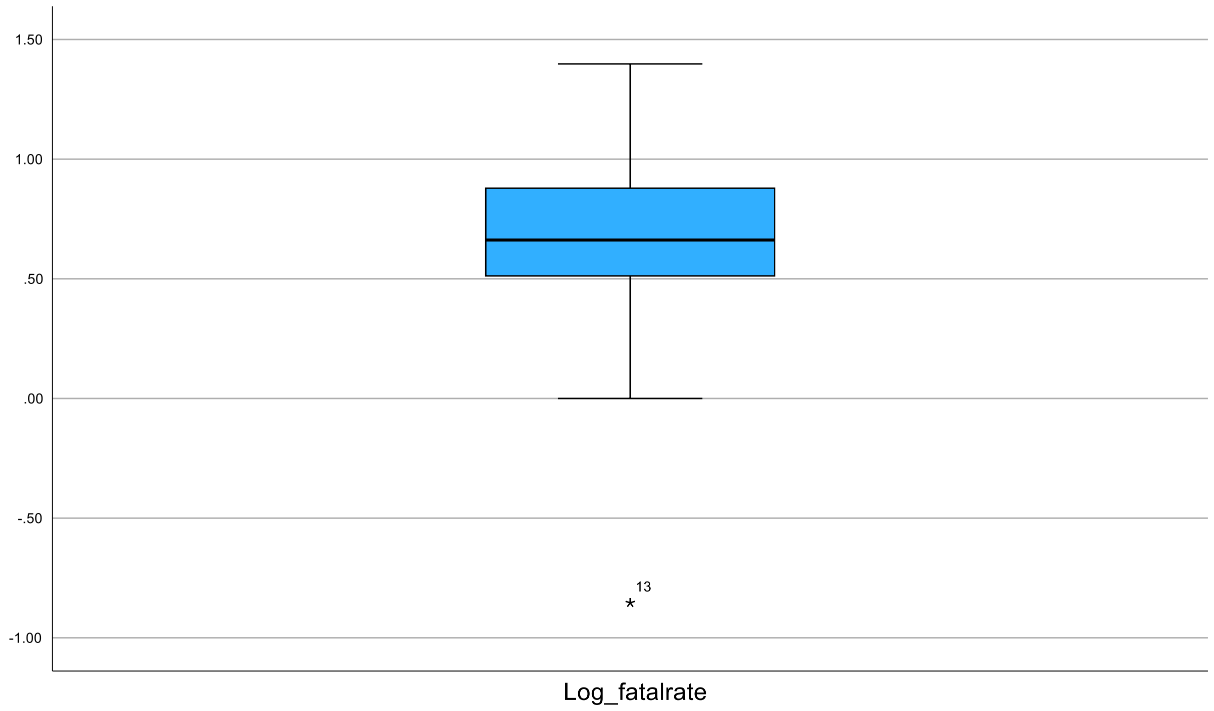

Log_nonfatalrate

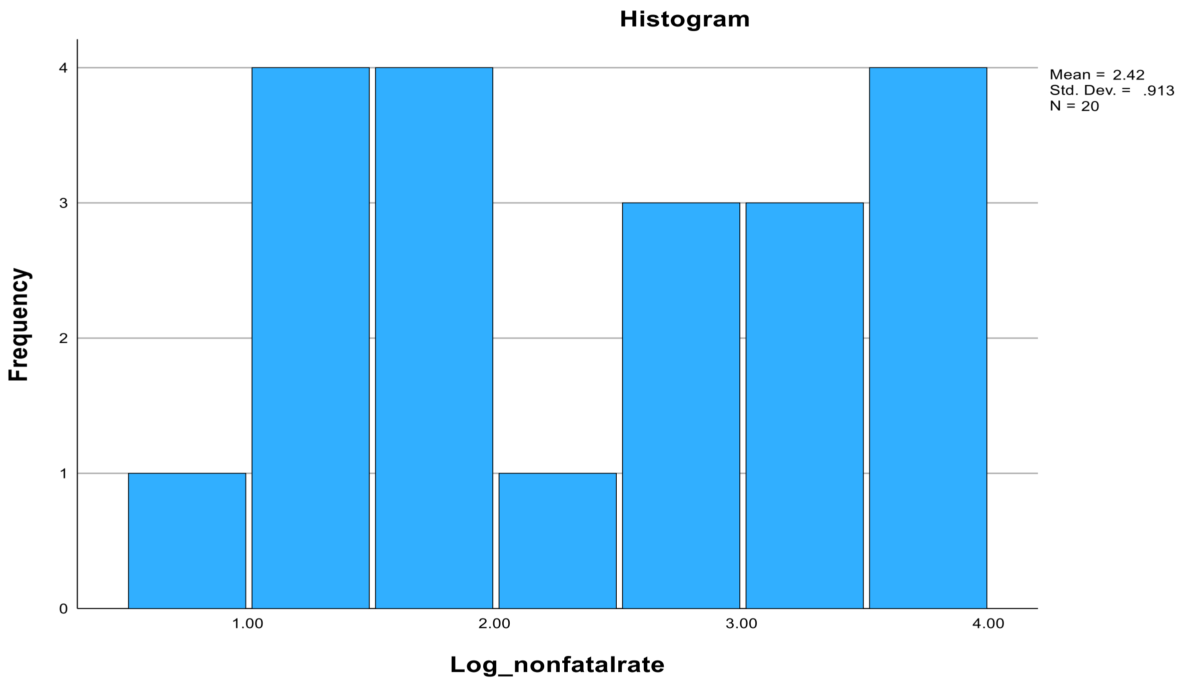

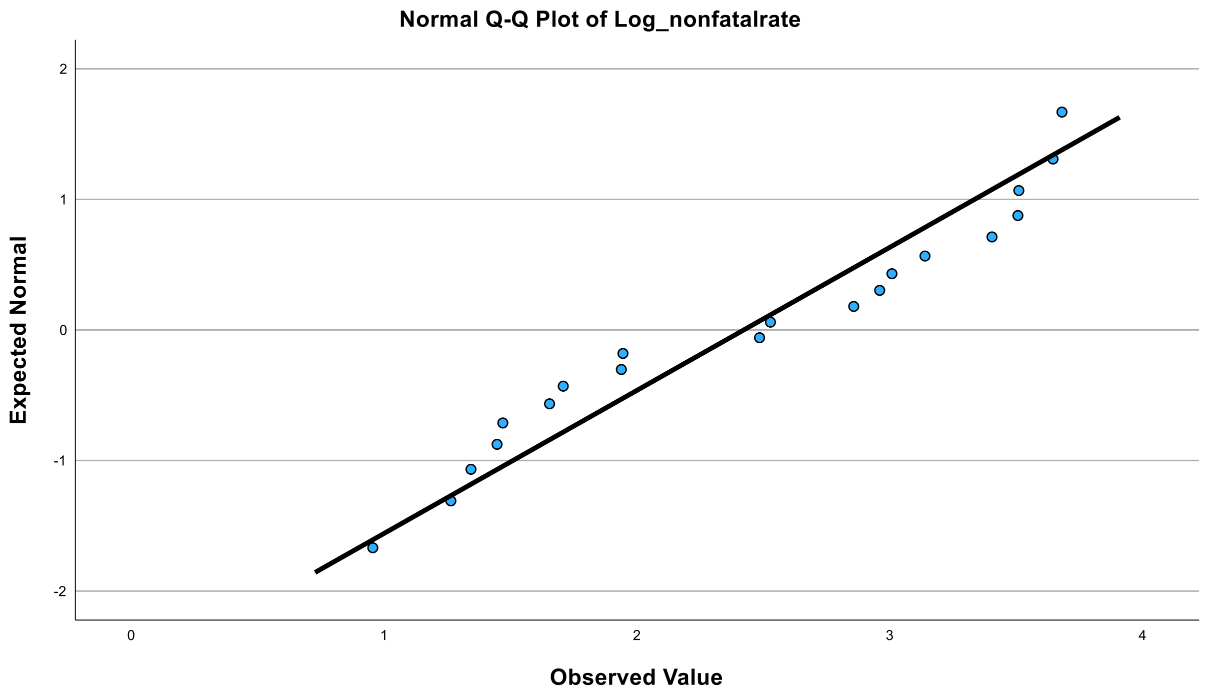
Normal Q-Q Plot of Log_nonfatalrate
Expected Normal
Observed Value
2
1
0
-1
-2
0
1
2
3
4

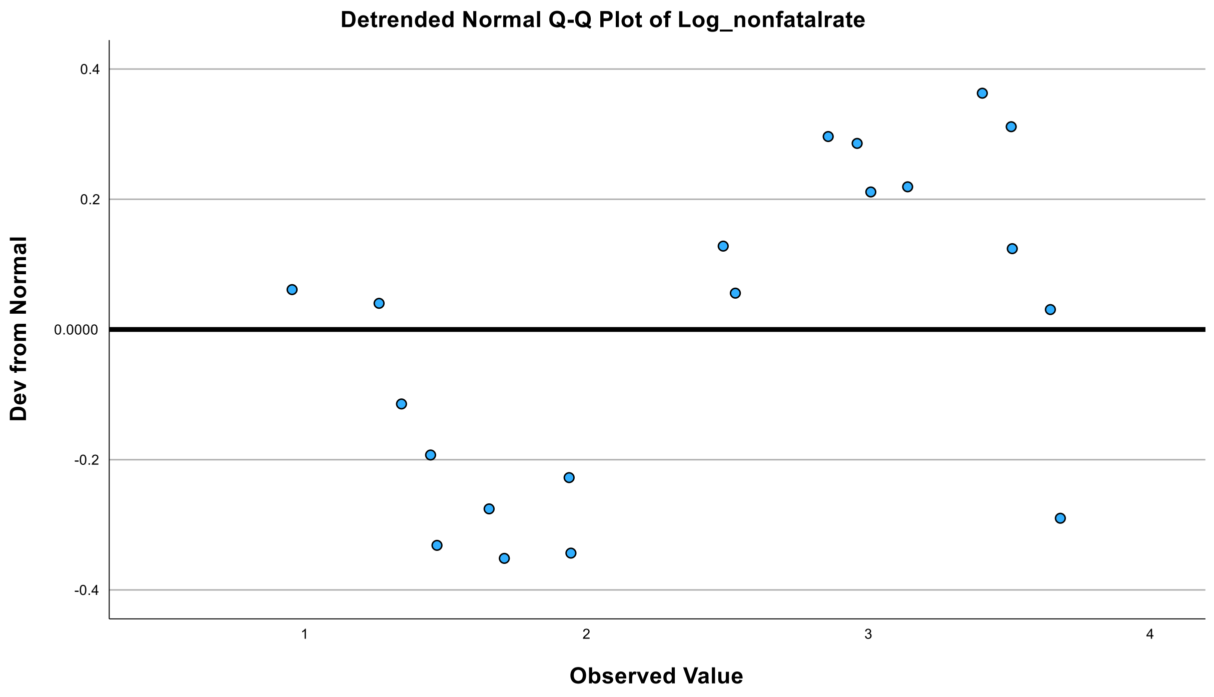
Detrended Normal Q-Q Plot of Log_nonfatalrate
Dev from Normal
Observed Value
0.4
0.2
0.0000
-0.2
-0.4
1
2
3
4

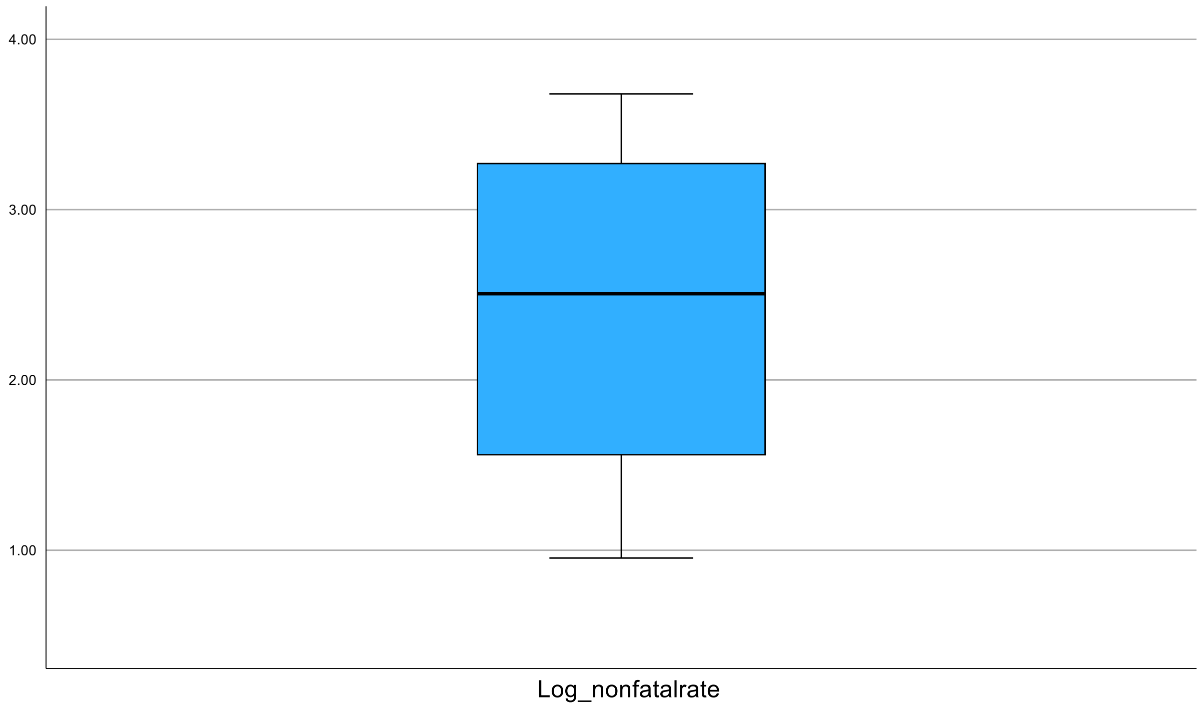

Continued.

Log-general linear multivariate regression with bootstrap-2000 with Confidence Intervals of 95% with reduced data (PP. 41-47)

Bootstrap

Notes

Output Created		24-FEB-2026 11:01:22
Comments		
Input	Data	E:\Group 2 - Feb 24\Group 2 - Descriptive and regression analysis.sav
	Active Dataset	DataSet0
	Filter	<none>
	Weight	<none>
	Split File	<none>
	N of Rows in Working Data File	20
Syntax		BOOTSTRAP<br>/SAMPLING METHOD=SIMPLE<br>/VARIABLES TARGET=Log_fatalrate Log_nonfatalrate INPUT= Inspector_ratio<br>/CRITERIA CILEVEL=95 CITYPE=PERCENTILE NSAMPLES=2000<br>/MISSING USERMISSING=EXCLUDE.
Resources	Processor Time	00:00:00.02
	Elapsed Time	00:00:00.01

Bootstrap Specifications

Sampling Method	Simple
Number of Samples	2000
Confidence Interval Level	95.0%
Confidence Interval Type	Percentile

General Linear Model

Notes

Output Created		24-FEB-2026 11:01:22
Comments		
Input	Data	E:\Group 2 - Feb 24\Group 2 - Descriptive and regression analysis.sav
	Active Dataset	DataSet0
	Filter	<none>
	Weight	<none>
	Split File	<none>
	N of Rows in Working Data File	40020
Missing Value Handling	Definition of Missing	User-defined missing values are treated as

		missing.
	Cases Used	Statistics are based on all cases with valid data for all variables in the model.
Syntax		GLM Log_fatalrate Log_nonfatalrate WITH Inspector_ratio<br>/METHOD=SSTYPE(3)<br>/INTERCEPT=INCLUDE<br>/SAVE=PRED SEPRED RESID ZRESID COOK LEVER<br>/EMMEANS=TABLES(OVERALL) WITH(Inspector_ratio=MEAN)<br>/PRINT=DESCRIPTIVE ETASQ OPOWER PARAMETER TEST(SSCP) RSSCP HOMOGENEITY LOF<br>/PLOT=SPREADLEVEL RESIDUALS<br>/CRITERIA=ALPHA(.05)<br>/DESIGN=Inspector_ratio.
Resources	Processor Time	00:00:28.56
	Elapsed Time	00:00:27.74

Warnings

The HOMOGENEITY specification in the PRINT subcommand will be ignored because there are no between-subjects factors.
The SPREADLEVEL specification has been found in the PLOT subcommand, but the model includes no factors. The spread versus level plot will not be produced.
This command is trying to save new variables while bootstrapping is in effect. The new variables will be lost.<br>Execution of this command stops.

Descriptive Statistics

	Mean	Std. Deviation	N
Log_fatalrate	.6471	.47875	20
Log_nonfatalrate	2.4213	.91285	20

Bartlett's Test of Sphericity[a]

Likelihood Ratio	.000
Approx. Chi-Square	15.582
df	2
Sig.	<.001

Tests the null hypothesis that the residual covariance matrix is proportional to an identity matrix.[a]

a. Design: Intercept + Inspector_ratio

Multivariate Tests[a]

Effect		Value	F	Hypothesis df	Error df	Sig.
Intercept	Pillai's Trace	.810	36.147[b]	2.000	17.000	<.001
	Wilks' Lambda	.190	36.147[b]	2.000	17.000	<.001
	Hotelling's Trace	4.253	36.147[b]	2.000	17.000	<.001
	Roy's Largest Root	4.253	36.147[b]	2.000	17.000	<.001
Inspector_ratio	Pillai's Trace	.108	1.032[b]	2.000	17.000	.378
	Wilks' Lambda	.892	1.032[b]	2.000	17.000	.378
	Hotelling's Trace	.121	1.032[b]	2.000	17.000	.378
	Roy's Largest Root	.121	1.032[b]	2.000	17.000	.378

Multivariate Tests[a]

Effect		Partial Eta Squared	Noncent. Parameter	Observed Power[c]
Intercept	Pillai's Trace	.810	72.293	1.000
	Wilks' Lambda	.810	72.293	1.000
	Hotelling's Trace	.810	72.293	1.000
	Roy's Largest Root	.810	72.293	1.000
Inspector_ratio	Pillai's Trace	.108	2.064	.200
	Wilks' Lambda	.108	2.064	.200
	Hotelling's Trace	.108	2.064	.200
	Roy's Largest Root	.108	2.064	.200

a. Design: Intercept + Inspector_ratio
b. Exact statistic
c. Computed using alpha = .05

Tests of Between-Subjects Effects

Source	Dependent Variable	Type III Sum of Squares	df	Mean Square	F	Sig.
Corrected Model	Log_fatalrate	.027[a]	1	.027	.113	.741
	Log_nonfatalrate	.649[b]	1	.649	.770	.392
Intercept	Log_fatalrate	3.162	1	3.162	13.153	.002
	Log_nonfatalrate	59.689	1	59.689	70.762	<.001
Inspector_ratio	Log_fatalrate	.027	1	.027	.113	.741
	Log_nonfatalrate	.649	1	.649	.770	.392
Error	Log_fatalrate	4.328	18	.240		
	Log_nonfatalrate	15.183	18	.844		
Total	Log_fatalrate	12.730	20			
	Log_nonfatalrate	133.091	20			
Corrected Total	Log_fatalrate	4.355	19			
	Log_nonfatalrate	15.833	19			

Tests of Between-Subjects Effects

Source	Dependent Variable	Partial Eta Squared	Noncent. Parameter	Observed Power[c]
Corrected Model	Log_fatalrate	.006	.113	.062
	Log_nonfatalrate	.041	.770	.132
Intercept	Log_fatalrate	.422	13.153	.929
	Log_nonfatalrate	.797	70.762	1.000
Inspector_ratio	Log_fatalrate	.006	.113	.062

	Log_nonfatalrate	.041	.770	.132
Error	Log_fatalrate			
	Log_nonfatalrate			
Total	Log_fatalrate			
	Log_nonfatalrate			
Corrected Total	Log_fatalrate			
	Log_nonfatalrate			

a. R Squared = .006 (Adjusted R Squared = -.049)
b. R Squared = .041 (Adjusted R Squared = -.012)
c. Computed using alpha = .05

Parameter Estimates

Dependent Variable	Parameter	B	Std. Error	t	Sig.	95% Confidence Interval Lower Bound
Log_fatalrate	Intercept	.605	.167	3.627	.002	.254
	Inspector_ratio	.065	.194	.336	.741	-.342
Log_nonfatalrate	Intercept	2.628	.312	8.412	<.001	1.972
	Inspector_ratio	-.319	.363	-.877	.392	-1.082

Parameter Estimates

Dependent Variable	Parameter	95% Confidence Interval Upper Bound	Partial Eta Squared	Noncent. Parameter	Observed Power[a]
Log_fatalrate	Intercept	.955	.422	3.627	.929
	Inspector_ratio	.473	.006	.336	.062
Log_nonfatalrate	Intercept	3.284	.797	8.412	1.000
	Inspector_ratio	.444	.041	.877	.132

a. Computed using alpha = .05

Bootstrap for Parameter Estimates

			Bootstrap[a]			
Dependent Variable	Parameter	B	Bias	Std. Error	Sig. (2-tailed)	95% Confidence Interval Lower
Log_fatalrate	Intercept	.605	-.011	.192	.013	.179
	Inspector_ratio	.065	.045	.223	.643	-.151
Log_nonfatalrate	Intercept	2.628	-.008	.338	<.001	1.927
	Inspector_ratio	-.319	.026	.351	.226	-.919

Bootstrap for Parameter Estimates

Dependent Variable	Parameter	Bootstrap 95% Confidence Interval Upper
Log_fatalrate	Intercept	.923
	Inspector_ratio	.712
Log_nonfatalrate	Intercept	3.268

Inspector_ratio	.551

a. Unless otherwise noted, bootstrap results are based on 2000 bootstrap samples

Between-Subjects SSCP Matrix

			Log_fatalrate	Log_nonfatalrate
Hypothesis	Intercept	Log_fatalrate	3.162	13.739
		Log_nonfatalrate	13.739	59.689
	Inspector_ratio	Log_fatalrate	.027	-.133
		Log_nonfatalrate	-.133	.649
Error		Log_fatalrate	4.328	5.258
		Log_nonfatalrate	5.258	15.183

Based on Type III Sum of Squares

Residual SSCP Matrix

		Log_fatalrate	Log_nonfatalrate
Sum-of-Squares and Cross-Products	Log_fatalrate	4.328	5.258
	Log_nonfatalrate	5.258	15.183
Covariance	Log_fatalrate	.240	.292
	Log_nonfatalrate	.292	.844
Correlation	Log_fatalrate	1.000	.649
	Log_nonfatalrate	.649	1.000

Based on Type III Sum of Squares

Lack of Fit

Multivariate Tests

Dependent Variables		Value	F	Hypothesis df	Error df
Log_fatalrate, Log_nonfatalrate	Pillai's Trace	1.766	.941	32.000	4.000
	Wilks' Lambda	.004	.984[a]	32.000	2.000
	Hotelling's Trace	63.779	.000	32.000	.000
	Roy's Largest Root	60.194	7.524[b]	16.000	2.000
Log_fatalrate	Pillai's Trace	.795	.483[a]	16.000	2.000
	Wilks' Lambda	.205	.483[a]	16.000	2.000
	Hotelling's Trace	3.867	.483[a]	16.000	2.000
	Roy's Largest Root	3.867	.483[a]	16.000	2.000
Log_nonfatalrate	Pillai's Trace	.848	.699[a]	16.000	2.000
	Wilks' Lambda	.152	.699[a]	16.000	2.000
	Hotelling's Trace	5.596	.699[a]	16.000	2.000
	Roy's Largest Root	5.596	.699[a]	16.000	2.000

Multivariate Tests

Dependent Variables		Sig.	Partial Eta Squared	Noncent. Parameter
Log_fatalrate, Log_nonfatalrate	Pillai's Trace	.609	.883	30.118
	Wilks' Lambda	.627	.940	31.498
	Hotelling's Trace	.	.970	.000
	Roy's Largest Root	.123	.984	120.389

Log_fatalrate	Pillai's Trace	.841	.795	7.734
	Wilks' Lambda	.841	.795	7.734
	Hotelling's Trace	.841	.795	7.734
	Roy's Largest Root	.841	.795	7.734
Log_nonfatalrate	Pillai's Trace	.732	.848	11.191
	Wilks' Lambda	.732	.848	11.191
	Hotelling's Trace	.732	.848	11.191
	Roy's Largest Root	.732	.848	11.191

Multivariate Tests

Dependent Variables		Observed Power[c]
Log_fatalrate, Log_nonfatalrate	Pillai's Trace	.149
	Wilks' Lambda	.097
	Hotelling's Trace	.
	Roy's Largest Root	.353
Log_fatalrate	Pillai's Trace	.073
	Wilks' Lambda	.073
	Hotelling's Trace	.073
	Roy's Largest Root	.073
Log_nonfatalrate	Pillai's Trace	.083
	Wilks' Lambda	.083
	Hotelling's Trace	.083
	Roy's Largest Root	.083

a. Exact statistic
b. The statistic is an upper bound on F that yields a lower bound on the significance level.
c. Computed using alpha = .05

Univariate Tests

Dependent Variable	Source	Sum of Squares	df	Mean Square	F	Sig.
Log_fatalrate	Lack of Fit	3.438	16	.215	.483	.841
	Pure Error	.889	2	.445		
Log_nonfatalrate	Lack of Fit	12.881	16	.805	.699	.732
	Pure Error	2.302	2	1.151		

Univariate Tests

Dependent Variable	Source	Partial Eta Squared	Noncent. Parameter	Observed Power[a]
Log_fatalrate	Lack of Fit	.795	7.734	.073
	Pure Error			
Log_nonfatalrate	Lack of Fit	.848	11.191	.083
	Pure Error			

a. Computed using alpha = .05

SSCP Matrix

		Log_fatalrate	Log_nonfatalrate
Lack of Fit	Log_fatalrate	3.438	3.876
	Log_nonfatalrate	3.876	12.881
Pure Error	Log_fatalrate	.889	1.382
	Log_nonfatalrate	1.382	2.302

Estimated Marginal Means

Grand Mean

Dependent Variable	Mean	Std. Error	95% Confidence Interval Lower Bound	95% Confidence Interval Upper Bound
Log_fatalrate	.647[a]	.110	.417	.877
Log_nonfatalrate	2.421[a]	.205	1.990	2.853

a. Covariates appearing in the model are evaluated at the following values: Inspector_ratio = .648000.

Continued.

Log-general linear multivariate regression analysis with reduced data (PP. 48-54)

Notes

Output Created		24-FEB-2026 11:02:37
Comments		
Input	Data	E:\Group 2 - Feb 24\Group 2 - Descriptive and regression analysis.sav
	Active Dataset	DataSet0
	Filter	<none>
	Weight	<none>
	Split File	<none>
	N of Rows in Working Data File	20
Missing Value Handling	Definition of Missing	User-defined missing values are treated as missing.
	Cases Used	Statistics are based on all cases with valid data for all variables in the model.
Syntax		GLM Log_fatalrate Log_nonfatalrate WITH Inspector_ratio /METHOD=SSTYPE(3) /INTERCEPT=INCLUDE /SAVE=PRED SEPRED RESID ZRESID COOK LEVER /EMMEANS=TABLES(OVERALL) WITH(Inspector_ratio=MEAN) /PRINT=DESCRIPTIVE ETASQ OPOWER PARAMETER TEST(SSCP) RSSCP HOMOGENEITY LOF /PLOT=SPREADLEVEL RESIDUALS /CRITERIA=ALPHA(.05) /DESIGN=Inspector_ratio.
Resources	Processor Time	00:00:00.28
	Elapsed Time	00:00:00.27
Variables Created or Modified	PRE_5	Predicted Value for Log_fatalrate
	PRE_6	Predicted Value for Log_nonfatalrate
	SEP_5	Standard Error of Predicted Value for Log_fatalrate
	SEP_6	Standard Error of Predicted Value for Log_nonfatalrate
	RES_5	Residual for Log_fatalrate
	RES_6	Residual for Log_nonfatalrate
	ZRE_5	Standardized Residual for Log_fatalrate
	ZRE_6	Standardized Residual for Log_nonfatalrate
	COO_5	Cook's Distance for Log_fatalrate
	COO_6	Cook's Distance for Log_nonfatalrate
	LEV_5	Uncentered Leverage Value for Log_fatalrate

LEV_6	Uncentered Leverage Value for Log_nonfatalrate

Warnings

The HOMOGENEITY specification in the PRINT subcommand will be ignored because there are no between-subjects factors.
The SPREADLEVEL specification has been found in the PLOT subcommand, but the model includes no factors. The spread versus level plot will not be produced.

Descriptive Statistics

	Mean	Std. Deviation	N
Log_fatalrate	.6471	.47875	20
Log_nonfatalrate	2.4213	.91285	20

Bartlett's Test of Sphericity[a]

Likelihood Ratio	.000
Approx. Chi-Square	15.582
df	2
Sig.	<.001

Tests the null hypothesis that the residual covariance matrix is proportional to an identity matrix.[a]
a. Design: Intercept + Inspector_ratio

Multivariate Tests[a]

Effect		Value	F	Hypothesis df	Error df	Sig.
Intercept	Pillai's Trace	.810	36.147[b]	2.000	17.000	<.001
	Wilks' Lambda	.190	36.147[b]	2.000	17.000	<.001
	Hotelling's Trace	4.253	36.147[b]	2.000	17.000	<.001
	Roy's Largest Root	4.253	36.147[b]	2.000	17.000	<.001
Inspector_ratio	Pillai's Trace	.108	1.032[b]	2.000	17.000	.378
	Wilks' Lambda	.892	1.032[b]	2.000	17.000	.378
	Hotelling's Trace	.121	1.032[b]	2.000	17.000	.378
	Roy's Largest Root	.121	1.032[b]	2.000	17.000	.378

Multivariate Tests[a]

Effect		Partial Eta Squared	Noncent. Parameter	Observed Power[c]
Intercept	Pillai's Trace	.810	72.293	1.000
	Wilks' Lambda	.810	72.293	1.000
	Hotelling's Trace	.810	72.293	1.000
	Roy's Largest Root	.810	72.293	1.000
Inspector_ratio	Pillai's Trace	.108	2.064	.200
	Wilks' Lambda	.108	2.064	.200
	Hotelling's Trace	.108	2.064	.200
	Roy's Largest Root	.108	2.064	.200

a. Design: Intercept + Inspector_ratio
b. Exact statistic
c. Computed using alpha = .05

Tests of Between-Subjects Effects

Source	Dependent Variable	Type III Sum of Squares	df	Mean Square	F	Sig.
Corrected Model	Log_fatalrate	.027[a]	1	.027	.113	.741
	Log_nonfatalrate	.649[b]	1	.649	.770	.392
Intercept	Log_fatalrate	3.162	1	3.162	13.153	.002
	Log_nonfatalrate	59.689	1	59.689	70.762	<.001
Inspector_ratio	Log_fatalrate	.027	1	.027	.113	.741
	Log_nonfatalrate	.649	1	.649	.770	.392
Error	Log_fatalrate	4.328	18	.240		
	Log_nonfatalrate	15.183	18	.844		
Total	Log_fatalrate	12.730	20			
	Log_nonfatalrate	133.091	20			
Corrected Total	Log_fatalrate	4.355	19			
	Log_nonfatalrate	15.833	19			

Tests of Between-Subjects Effects

Source	Dependent Variable	Partial Eta Squared	Noncent. Parameter	Observed Power[c]
Corrected Model	Log_fatalrate	.006	.113	.062
	Log_nonfatalrate	.041	.770	.132
Intercept	Log_fatalrate	.422	13.153	.929
	Log_nonfatalrate	.797	70.762	1.000
Inspector_ratio	Log_fatalrate	.006	.113	.062
	Log_nonfatalrate	.041	.770	.132
Error	Log_fatalrate			
	Log_nonfatalrate			
Total	Log_fatalrate			
	Log_nonfatalrate			
Corrected Total	Log_fatalrate			
	Log_nonfatalrate			

a. R Squared = .006 (Adjusted R Squared = -.049)
b. R Squared = .041 (Adjusted R Squared = -.012)
c. Computed using alpha = .05

Parameter Estimates

Dependent Variable	Parameter	B	Std. Error	t	Sig.	95% Confidence Interval Lower Bound
Log_fatalrate	Intercept	.605	.167	3.627	.002	.254
	Inspector_ratio	.065	.194	.336	.741	-.342
Log_nonfatalrate	Intercept	2.628	.312	8.412	<.001	1.972
	Inspector_ratio	-.319	.363	-.877	.392	-1.082

Parameter Estimates

Dependent Variable	Parameter	95% Confidence Interval Upper Bound	Partial Eta Squared	Noncent. Parameter	Observed Power[a]
Log_fatalrate	Intercept	.955	.422	3.627	.929
	Inspector_ratio	.473	.006	.336	.062
Log_nonfatalrate	Intercept	3.284	.797	8.412	1.000
	Inspector_ratio	.444	.041	.877	.132

a. Computed using alpha = .05

Between-Subjects SSCP Matrix

			Log_fatalrate	Log_nonfatalrate
Hypothesis	Intercept	Log_fatalrate	3.162	13.739
		Log_nonfatalrate	13.739	59.689
	Inspector_ratio	Log_fatalrate	.027	-.133
		Log_nonfatalrate	-.133	.649
Error		Log_fatalrate	4.328	5.258
		Log_nonfatalrate	5.258	15.183

Based on Type III Sum of Squares

Residual SSCP Matrix

		Log_fatalrate	Log_nonfatalrate
Sum-of-Squares and Cross-Products	Log_fatalrate	4.328	5.258
	Log_nonfatalrate	5.258	15.183
Covariance	Log_fatalrate	.240	.292
	Log_nonfatalrate	.292	.844
Correlation	Log_fatalrate	1.000	.649
	Log_nonfatalrate	.649	1.000

Based on Type III Sum of Squares

Lack of Fit

Multivariate Tests

Dependent Variables		Value	F	Hypothesis df	Error df
Log_fatalrate, Log_nonfatalrate	Pillai's Trace	1.766	.941	32.000	4.000
	Wilks' Lambda	.004	.984[a]	32.000	2.000
	Hotelling's Trace	63.779	.000	32.000	.000
	Roy's Largest Root	60.194	7.524[b]	16.000	2.000
Log_fatalrate	Pillai's Trace	.795	.483[a]	16.000	2.000
	Wilks' Lambda	.205	.483[a]	16.000	2.000
	Hotelling's Trace	3.867	.483[a]	16.000	2.000
	Roy's Largest Root	3.867	.483[a]	16.000	2.000
Log_nonfatalrate	Pillai's Trace	.848	.699[a]	16.000	2.000
	Wilks' Lambda	.152	.699[a]	16.000	2.000
	Hotelling's Trace	5.596	.699[a]	16.000	2.000
	Roy's Largest Root	5.596	.699[a]	16.000	2.000

Multivariate Tests

Dependent Variables		Sig.	Partial Eta Squared	Noncent. Parameter
Log_fatalrate, Log_nonfatalrate	Pillai's Trace	.609	.883	30.118
	Wilks' Lambda	.627	.940	31.498
	Hotelling's Trace	.	.970	.000
	Roy's Largest Root	.123	.984	120.389
Log_fatalrate	Pillai's Trace	.841	.795	7.734
	Wilks' Lambda	.841	.795	7.734
	Hotelling's Trace	.841	.795	7.734
	Roy's Largest Root	.841	.795	7.734
Log_nonfatalrate	Pillai's Trace	.732	.848	11.191
	Wilks' Lambda	.732	.848	11.191
	Hotelling's Trace	.732	.848	11.191
	Roy's Largest Root	.732	.848	11.191

Multivariate Tests

Dependent Variables		Observed Power[c]
Log_fatalrate, Log_nonfatalrate	Pillai's Trace	.149
	Wilks' Lambda	.097
	Hotelling's Trace	.
	Roy's Largest Root	.353
Log_fatalrate	Pillai's Trace	.073
	Wilks' Lambda	.073
	Hotelling's Trace	.073
	Roy's Largest Root	.073
Log_nonfatalrate	Pillai's Trace	.083
	Wilks' Lambda	.083
	Hotelling's Trace	.083
	Roy's Largest Root	.083

a. Exact statistic
b. The statistic is an upper bound on F that yields a lower bound on the significance level.
c. Computed using alpha = .05

Univariate Tests

Dependent Variable	Source	Sum of Squares	df	Mean Square	F	Sig.
Log_fatalrate	Lack of Fit	3.438	16	.215	.483	.841
	Pure Error	.889	2	.445		
Log_nonfatalrate	Lack of Fit	12.881	16	.805	.699	.732
	Pure Error	2.302	2	1.151		

Univariate Tests

Dependent Variable	Source	Partial Eta Squared	Noncent. Parameter	Observed Power[a]
Log_fatalrate	Lack of Fit	.795	7.734	.073
	Pure Error			
Log_nonfatalrate	Lack of Fit	.848	11.191	.083
	Pure Error			

a. Computed using alpha = .05

SSCP Matrix

		Log_fatalrate	Log_nonfatalrate
Lack of Fit	Log_fatalrate	3.438	3.876
	Log_nonfatalrate	3.876	12.881
Pure Error	Log_fatalrate	.889	1.382
	Log_nonfatalrate	1.382	2.302

Estimated Marginal Means

Grand Mean

Dependent Variable	Mean	Std. Error	95% Confidence Interval Lower Bound	95% Confidence Interval Upper Bound
Log_fatalrate	.647[a]	.110	.417	.877
Log_nonfatalrate	2.421[a]	.205	1.990	2.853

a. Covariates appearing in the model are evaluated at the following values: Inspector_ratio = .648000.

Observed * Predicted * Std. Residual Plots

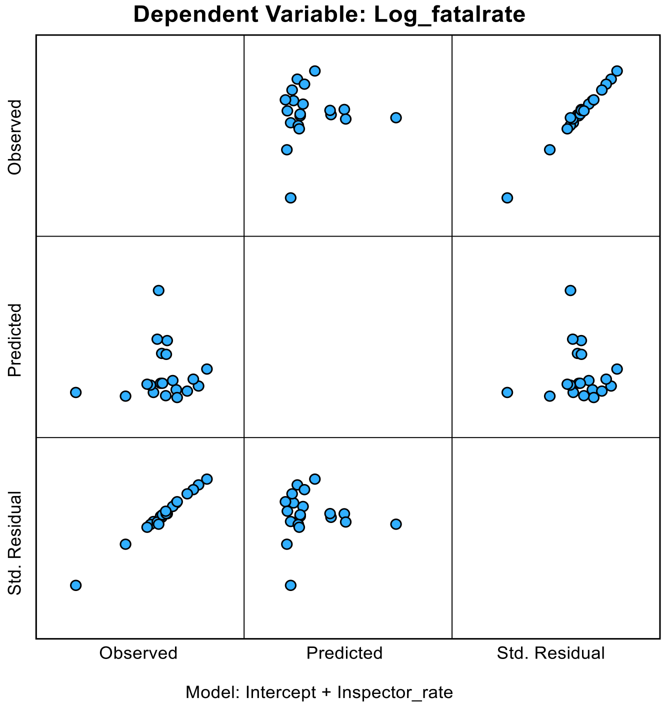

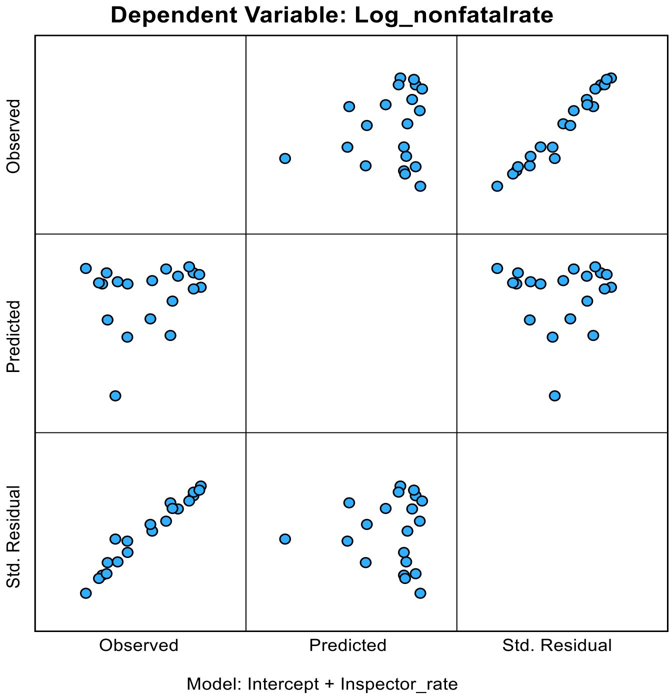

Thus, following analysis have been completed with IBM SPSS 30.0 for ILO's group 2, as hereunder-

- Descriptive statistics with original data associated with the research variables,
- General linear multivariate regression (with re-runs where applicable),
- General linear multivariate regression with bootstrap-2000 with Confidence Intervals of 95%,
- Descriptive statistics with log-transformation of dependent variables,
- Log-general linear multivariate regression with bootstrap-2000 with Confidence Intervals of 95% and
- Log-general linear multivariate regression analysis.
