# Supplementary material for: OSH inspector ratio to strengthen decent workplace safety and health: a cross-regional trend analysis research
Source: Front Public Health. 2026 Apr 8;14:1806383. doi: 10.3389/fpubh.2026.1806383 (PMC13099828; doi:10.3389/fpubh.2026.1806383)
Supplement: Supplementary file 1 [file Data_Sheet_1.zip › Supplementary document Groups 3 and 4 - Descriptive and regression analysis.docx]

Supplementary Material

**Groups 3 and 4 – Descriptive and multivariate regression analysis**

IBM SPSS 30.0 software was used to complete descriptive statistics and multivariate regression analysis for income groups 3 and 4, which originally had International Labour Organization’s (ILO) 12 nations or regions. There are six major sections in this supplementary material as listed hereunder:

1. Descriptive statistics with original data associated with the research variables (PP. 2-10),
2. General linear multivariate regression (PP. 11-17),
3. General linear multivariate regression with bootstrap-2000 with Confidence Intervals of 95% (PP. 18-24),
4. Descriptive statistics with log-transformation of dependent variables (PP. 25-33),
5. Log-general linear multivariate regression with bootstrap-2000 with Confidence Intervals of 95% (PP. 34-40), and
6. Log-general linear multivariate regression analysis (PP. 41-47).

**Note:**

- Inspector ratio or inspector_ratio, or inspector_rate = Independent Variable (IV),
- Falta injury rate or fatal_rate = Dependent Variable (DV1), and
- Nonfatal injury rate or nonfatal_rate = Dependent Variable (DV2).

**Descriptive statistics with original data associated with the research variables (PP. 2-10)**

**Explore**

| **Notes** | | |
| --- | --- | --- |
| Output Created | | 24-FEB-2026 09:34:05 |
| Comments | |  |
| Input | Data | E:\Group 3 and 4 - Feb 24\Groups 3 and 4 - Descriptive and regression analysis.sav |
|  | Active Dataset | DataSet0 |
|  | Filter | <none> |
|  | Weight | <none> |
|  | Split File | <none> |
|  | N of Rows in Working Data File | 12 |
| Missing Value Handling | Definition of Missing | User-defined missing values for dependent variables are treated as missing. |
|  | Cases Used | Statistics are based on cases with no missing values for any dependent variable or factor used. |
| Syntax | | EXAMINE VARIABLES=Inspector_ratio Fatal_rate Nonfatal_rate /PLOT BOXPLOT HISTOGRAM NPPLOT /COMPARE GROUPS /STATISTICS DESCRIPTIVES EXTREME /CINTERVAL 95 /MISSING LISTWISE /NOTOTAL. |
| Resources | Processor Time | 00:00:05.55 |
|  | Elapsed Time | 00:00:01.88 |

| **Case Processing Summary** | | | | | | |
| --- | --- | --- | --- | --- | --- | --- |
|  | Cases | | | | | |
|  | Valid | | Missing | | Total | |
|  | N | Percent | N | Percent | N | Percent |
| Inspector_ratio | 12 | 100.0% | 0 | 0.0% | 12 | 100.0% |
| Fatal_rate | 12 | 100.0% | 0 | 0.0% | 12 | 100.0% |
| Nonfatal_rate | 12 | 100.0% | 0 | 0.0% | 12 | 100.0% |

| **Descriptives** | | | | |
| --- | --- | --- | --- | --- |
|  | | | Statistic | Std. Error |
| Inspector_ratio | Mean | | .495000 | .0805521 |
|  | 95% Confidence Interval for Mean | Lower Bound | .317706 |  |
|  |  | Upper Bound | .672294 |  |
|  | 5% Trimmed Mean | | .495000 |  |
|  | Median | | .515000 |  |
|  | Variance | | .078 |  |
|  | Std. Deviation | | .2790406 |  |
|  | Minimum | | .0700 |  |
|  | Maximum | | .9200 |  |
|  | Range | | .8500 |  |
|  | Interquartile Range | | .4925 |  |
|  | Skewness | | -.037 | .637 |
|  | Kurtosis | | -1.132 | 1.232 |
| Fatal_rate | Mean | | 5.451333 | 1.4313466 |
|  | 95% Confidence Interval for Mean | Lower Bound | 2.300961 |  |
|  |  | Upper Bound | 8.601706 |  |
|  | 5% Trimmed Mean | | 5.143704 |  |
|  | Median | | 4.000000 |  |
|  | Variance | | 24.585 |  |
|  | Std. Deviation | | 4.9583299 |  |
|  | Minimum | | .1400 |  |
|  | Maximum | | 16.3000 |  |
|  | Range | | 16.1600 |  |
|  | Interquartile Range | | 5.7635 |  |
|  | Skewness | | 1.210 | .637 |
|  | Kurtosis | | .986 | 1.232 |
| Nonfatal_rate | Mean | | 467.108333 | 226.0748510 |
|  | 95% Confidence Interval for Mean | Lower Bound | -30.479059 |  |
|  |  | Upper Bound | 964.695725 |  |
|  | 5% Trimmed Mean | | 389.978704 |  |
|  | Median | | 120.000000 |  |
|  | Variance | | 613318.059 |  |
|  | Std. Deviation | | 783.1462565 |  |
|  | Minimum | | 10.0000 |  |
|  | Maximum | | 2312.5500 |  |
|  | Range | | 2302.5500 |  |
|  | Interquartile Range | | 420.4300 |  |
|  | Skewness | | 1.966 | .637 |
|  | Kurtosis | | 2.661 | 1.232 |

| **Extreme Values** | | | | |
| --- | --- | --- | --- | --- |
|  | | | Case Number | Value |
| Inspector_ratio | Highest | 1 | 5 | .9200 |
|  |  | 2 | 3 | .8500 |
|  |  | 3 | 8 | .7500 |
|  |  | 4 | 2 | .6900 |
|  |  | 5 | 7 | .5800 |
|  | Lowest | 1 | 4 | .0700 |
|  |  | 2 | 6 | .1300 |
|  |  | 3 | 9 | .2200 |
|  |  | 4 | 11 | .3100 |
|  |  | 5 | 12 | .3900 |
| Fatal_rate | Highest | 1 | 12 | 16.3000 |
|  |  | 2 | 2 | 13.1500 |
|  |  | 3 | 8 | 7.6000 |
|  |  | 4 | 3 | 6.4000 |
|  |  | 5 | 10 | 6.0000 |
|  | Lowest | 1 | 1 | .1400 |
|  |  | 2 | 7 | .4800 |
|  |  | 3 | 5 | 1.0000 |
|  |  | 4 | 4 | 3.1460 |
|  |  | 5 | 6 | 3.2000 |
| Nonfatal_rate | Highest | 1 | 2 | 2312.5500 |
|  |  | 2 | 11 | 1894.7400 |
|  |  | 3 | 12 | 484.0000 |
|  |  | 4 | 10 | 322.0000 |
|  |  | 5 | 6 | 227.0000 |
|  | Lowest | 1 | 9 | 10.0000 |
|  |  | 2 | 4 | 11.8300 |
|  |  | 3 | 7 | 21.2000 |
|  |  | 4 | 1 | 28.6800 |
|  |  | 5 | 3 | 53.3000 |

| **Tests of Normality** | | | | | | |
| --- | --- | --- | --- | --- | --- | --- |
|  | Kolmogorov-Smirnov^a^ | | | Shapiro-Wilk | | |
|  | Statistic | df | Sig. | Statistic | df | Sig. |
| Inspector_ratio | .092 | 12 | .200^*^ | .966 | 12 | .868 |
| Fatal_rate | .174 | 12 | .200^*^ | .878 | 12 | .082 |
| Nonfatal_rate | .325 | 12 | .001 | .629 | 12 | <.001 |
| *. This is a lower bound of the true significance. | | | | | | |
| a. Lilliefors Significance Correction | | | | | | |

**Inspector_ratio**

**Fatal_rate**

**Nonfatal_rate**

**General linear multivariate regression (PP. 11-17)**

| **Notes** | | |
| --- | --- | --- |
| Output Created | | 24-FEB-2026 09:34:55 |
| Comments | |  |
| Input | Data | E:\Group 3 and 4 - Feb 24\Groups 3 and 4 - Descriptive and regression analysis.sav |
|  | Active Dataset | DataSet0 |
|  | Filter | <none> |
|  | Weight | <none> |
|  | Split File | <none> |
|  | N of Rows in Working Data File | 12 |
| Missing Value Handling | Definition of Missing | User-defined missing values are treated as missing. |
|  | Cases Used | Statistics are based on all cases with valid data for all variables in the model. |
| Syntax | | GLM Fatal_rate Nonfatal_rate WITH Inspector_ratio /METHOD=SSTYPE(3) /INTERCEPT=INCLUDE /SAVE=PRED SEPRED RESID ZRESID COOK LEVER /EMMEANS=TABLES(OVERALL) WITH(Inspector_ratio=MEAN) /PRINT=DESCRIPTIVE ETASQ OPOWER PARAMETER TEST(SSCP) RSSCP HOMOGENEITY LOF /PLOT=SPREADLEVEL RESIDUALS /CRITERIA=ALPHA(.05) /DESIGN=Inspector_ratio. |
| Resources | Processor Time | 00:00:00.53 |
|  | Elapsed Time | 00:00:00.27 |
| Variables Created or Modified | PRE_1 | Predicted Value for Fatal_rate |
|  | PRE_2 | Predicted Value for Nonfatal_rate |
|  | SEP_1 | Standard Error of Predicted Value for Fatal_rate |
|  | SEP_2 | Standard Error of Predicted Value for Nonfatal_rate |
|  | RES_1 | Residual for Fatal_rate |
|  | RES_2 | Residual for Nonfatal_rate |
|  | ZRE_1 | Standardized Residual for Fatal_rate |
|  | ZRE_2 | Standardized Residual for Nonfatal_rate |
|  | COO_1 | Cook's Distance for Fatal_rate |
|  | COO_2 | Cook's Distance for Nonfatal_rate |
|  | LEV_1 | Uncentered Leverage Value for Fatal_rate |
|  | LEV_2 | Uncentered Leverage Value for Nonfatal_rate |

| **Warnings** |
| --- |
| The HOMOGENEITY specification in the PRINT subcommand will be ignored because there are no between-subjects factors. |
| The SPREADLEVEL specification has been found in the PLOT subcommand, but the model includes no factors. The spread versus level plot will not be produced. |

| **Descriptive Statistics** | | | |
| --- | --- | --- | --- |
|  | Mean | Std. Deviation | N |
| Fatal_rate | 5.451333 | 4.9583299 | 12 |
| Nonfatal_rate | 467.108333 | 783.1462565 | 12 |

| **Bartlett's Test of Sphericity**^a^ | |
| --- | --- |
| Likelihood Ratio | .000 |
| Approx. Chi-Square | 81.090 |
| df | 2 |
| Sig. | <.001 |
| Tests the null hypothesis that the residual covariance matrix is proportional to an identity matrix.^a^  a. Design: Intercept + Inspector_ratio | |

| **Multivariate Tests**^a^ | | | | | | |
| --- | --- | --- | --- | --- | --- | --- |
| Effect | | Value | F | Hypothesis df | Error df | Sig. |
| Intercept | Pillai's Trace | .174 | .947^b^ | 2.000 | 9.000 | .423 |
|  | Wilks' Lambda | .826 | .947^b^ | 2.000 | 9.000 | .423 |
|  | Hotelling's Trace | .210 | .947^b^ | 2.000 | 9.000 | .423 |
|  | Roy's Largest Root | .210 | .947^b^ | 2.000 | 9.000 | .423 |
| Inspector_ratio | Pillai's Trace | .012 | .053^b^ | 2.000 | 9.000 | .949 |
|  | Wilks' Lambda | .988 | .053^b^ | 2.000 | 9.000 | .949 |
|  | Hotelling's Trace | .012 | .053^b^ | 2.000 | 9.000 | .949 |
|  | Roy's Largest Root | .012 | .053^b^ | 2.000 | 9.000 | .949 |

| **Multivariate Tests**^a^ | | | | |
| --- | --- | --- | --- | --- |
| Effect | | Partial Eta Squared | Noncent. Parameter | Observed Power^c^ |
| Intercept | Pillai's Trace | .174 | 1.894 | .166 |
|  | Wilks' Lambda | .174 | 1.894 | .166 |
|  | Hotelling's Trace | .174 | 1.894 | .166 |
|  | Roy's Largest Root | .174 | 1.894 | .166 |
| Inspector_ratio | Pillai's Trace | .012 | .106 | .056 |
|  | Wilks' Lambda | .012 | .106 | .056 |
|  | Hotelling's Trace | .012 | .106 | .056 |
|  | Roy's Largest Root | .012 | .106 | .056 |
| a. Design: Intercept + Inspector_ratio | | | | |
| b. Exact statistic | | | | |
| c. Computed using alpha = .05 | | | | |

| **Tests of Between-Subjects Effects** | | | | | | |
| --- | --- | --- | --- | --- | --- | --- |
| Source | Dependent Variable | Type III Sum of Squares | df | Mean Square | F | Sig. |
| Corrected Model | Fatal_rate | 3.011^a^ | 1 | 3.011 | .113 | .744 |
|  | Nonfatal_rate | 6338.992^b^ | 1 | 6338.992 | .009 | .925 |
| Intercept | Fatal_rate | 55.383 | 1 | 55.383 | 2.071 | .181 |
|  | Nonfatal_rate | 487859.801 | 1 | 487859.801 | .724 | .415 |
| Inspector_ratio | Fatal_rate | 3.011 | 1 | 3.011 | .113 | .744 |
|  | Nonfatal_rate | 6338.992 | 1 | 6338.992 | .009 | .925 |
| Error | Fatal_rate | 267.424 | 10 | 26.742 |  |  |
|  | Nonfatal_rate | 6740159.659 | 10 | 674015.966 |  |  |
| Total | Fatal_rate | 627.040 | 12 |  |  |  |
|  | Nonfatal_rate | 9364780.991 | 12 |  |  |  |
| Corrected Total | Fatal_rate | 270.435 | 11 |  |  |  |
|  | Nonfatal_rate | 6746498.651 | 11 |  |  |  |

| **Tests of Between-Subjects Effects** | | | | |
| --- | --- | --- | --- | --- |
| Source | Dependent Variable | Partial Eta Squared | Noncent. Parameter | Observed Power^c^ |
| Corrected Model | Fatal_rate | .011 | .113 | .061 |
|  | Nonfatal_rate | .001 | .009 | .051 |
| Intercept | Fatal_rate | .172 | 2.071 | .256 |
|  | Nonfatal_rate | .067 | .724 | .120 |
| Inspector_ratio | Fatal_rate | .011 | .113 | .061 |
|  | Nonfatal_rate | .001 | .009 | .051 |
| Error | Fatal_rate |  |  |  |
|  | Nonfatal_rate |  |  |  |
| Total | Fatal_rate |  |  |  |
|  | Nonfatal_rate |  |  |  |
| Corrected Total | Fatal_rate |  |  |  |
|  | Nonfatal_rate |  |  |  |
| a. R Squared = .011 (Adjusted R Squared = -.088) | | | | |
| b. R Squared = .001 (Adjusted R Squared = -.099) | | | | |
| c. Computed using alpha = .05 | | | | |

| **Parameter Estimates** | | | | | | |
| --- | --- | --- | --- | --- | --- | --- |
| Dependent Variable | Parameter | B | Std. Error | t | Sig. | 95% Confidence Interval |
|  |  |  |  |  |  | Lower Bound |
| Fatal_rate | Intercept | 4.523 | 3.143 | 1.439 | .181 | -2.480 |
|  | Inspector_ratio | 1.875 | 5.588 | .336 | .744 | -10.575 |
| Nonfatal_rate | Intercept | 424.524 | 498.987 | .851 | .415 | -687.290 |
|  | Inspector_ratio | 86.029 | 887.098 | .097 | .925 | -1890.547 |

| **Parameter Estimates** | | | | | |
| --- | --- | --- | --- | --- | --- |
| Dependent Variable | Parameter | 95% Confidence Interval | Partial Eta Squared | Noncent. Parameter | Observed Power^a^ |
|  |  | Upper Bound |  |  |  |
| Fatal_rate | Intercept | 11.526 | .172 | 1.439 | .256 |
|  | Inspector_ratio | 14.325 | .011 | .336 | .061 |
| Nonfatal_rate | Intercept | 1536.337 | .067 | .851 | .120 |
|  | Inspector_ratio | 2062.606 | .001 | .097 | .051 |
| a. Computed using alpha = .05 | | | | | |

| **Between-Subjects SSCP Matrix** | | | | |
| --- | --- | --- | --- | --- |
|  | | | Fatal_rate | Nonfatal_rate |
| Hypothesis | Intercept | Fatal_rate | 55.383 | 5197.998 |
|  |  | Nonfatal_rate | 5197.998 | 487859.801 |
|  | Inspector_ratio | Fatal_rate | 3.011 | 138.163 |
|  |  | Nonfatal_rate | 138.163 | 6338.992 |
| Error | | Fatal_rate | 267.424 | 20364.095 |
|  |  | Nonfatal_rate | 20364.095 | 6740159.659 |
| Based on Type III Sum of Squares | | | | |

| **Residual SSCP Matrix** | | | |
| --- | --- | --- | --- |
|  | | Fatal_rate | Nonfatal_rate |
| Sum-of-Squares and Cross-Products | Fatal_rate | 267.424 | 20364.095 |
|  | Nonfatal_rate | 20364.095 | 6740159.659 |
| Covariance | Fatal_rate | 26.742 | 2036.410 |
|  | Nonfatal_rate | 2036.410 | 674015.966 |
| Correlation | Fatal_rate | 1.000 | .480 |
|  | Nonfatal_rate | .480 | 1.000 |
| Based on Type III Sum of Squares | | | |

**Lack of Fit**

| **Multivariate Tests** | | | | | |
| --- | --- | --- | --- | --- | --- |
| Dependent Variables | | Value | F | Hypothesis df | Error df |
| Fatal_rate, Nonfatal_rate | Pillai's Trace | .^a^ | . | . | . |
|  | Wilks' Lambda | .^a^ | . | . | . |
|  | Hotelling's Trace | .^a^ | . | . | . |
|  | Roy's Largest Root | .^a^ | . | . | . |
| Fatal_rate | Pillai's Trace | .^a^ | . | . | . |
|  | Wilks' Lambda | .^a^ | . | . | . |
|  | Hotelling's Trace | .^a^ | . | . | . |
|  | Roy's Largest Root | .^a^ | . | . | . |
| Nonfatal_rate | Pillai's Trace | .^a^ | . | . | . |
|  | Wilks' Lambda | .^a^ | . | . | . |
|  | Hotelling's Trace | .^a^ | . | . | . |
|  | Roy's Largest Root | .^a^ | . | . | . |

| **Multivariate Tests** | | | | |
| --- | --- | --- | --- | --- |
| Dependent Variables | | Sig. | Partial Eta Squared | Noncent. Parameter |
| Fatal_rate, Nonfatal_rate | Pillai's Trace | . | . | . |
|  | Wilks' Lambda | . | . | . |
|  | Hotelling's Trace | . | . | . |
|  | Roy's Largest Root | . | . | . |
| Fatal_rate | Pillai's Trace | . | . | . |
|  | Wilks' Lambda | . | . | . |
|  | Hotelling's Trace | . | . | . |
|  | Roy's Largest Root | . | . | . |
| Nonfatal_rate | Pillai's Trace | . | . | . |
|  | Wilks' Lambda | . | . | . |
|  | Hotelling's Trace | . | . | . |
|  | Roy's Largest Root | . | . | . |

| **Multivariate Tests** | | |
| --- | --- | --- |
| Dependent Variables | | Observed Power^b^ |
| Fatal_rate, Nonfatal_rate | Pillai's Trace | . |
|  | Wilks' Lambda | . |
|  | Hotelling's Trace | . |
|  | Roy's Largest Root | . |
| Fatal_rate | Pillai's Trace | . |
|  | Wilks' Lambda | . |
|  | Hotelling's Trace | . |
|  | Roy's Largest Root | . |
| Nonfatal_rate | Pillai's Trace | . |
|  | Wilks' Lambda | . |
|  | Hotelling's Trace | . |
|  | Roy's Largest Root | . |
| a. The pure error sub-matrix corresponding to the selected dependent variables is a zero matrix. | | |
| b. Computed using alpha = .05 | | |

| **Univariate Tests** | | | | | | |
| --- | --- | --- | --- | --- | --- | --- |
| Dependent Variable | Source | Sum of Squares | df | Mean Square | F | Sig. |
| Fatal_rate | Lack of Fit | 267.424 | 10 | 26.742 | . | . |
|  | Pure Error | .000 | 0 | . |  |  |
| Nonfatal_rate | Lack of Fit | 6740159.659 | 10 | 674015.966 | . | . |
|  | Pure Error | .000 | 0 | . |  |  |

| **Univariate Tests** | | | | |
| --- | --- | --- | --- | --- |
| Dependent Variable | Source | Partial Eta Squared | Noncent. Parameter | Observed Power^a^ |
| Fatal_rate | Lack of Fit | 1.000 | . | . |
|  | Pure Error |  |  |  |
| Nonfatal_rate | Lack of Fit | 1.000 | . | . |
|  | Pure Error |  |  |  |
| a. Computed using alpha = .05 | | | | |

| **SSCP Matrix** | | | |
| --- | --- | --- | --- |
|  | | Fatal_rate | Nonfatal_rate |
| Lack of Fit | Fatal_rate | 267.424 | 20364.095 |
|  | Nonfatal_rate | 20364.095 | 6740159.659 |
| Pure Error | Fatal_rate | .000 | .000 |
|  | Nonfatal_rate | .000 | .000 |

**Estimated Marginal Means**

| **Grand Mean** | | | | |
| --- | --- | --- | --- | --- |
| Dependent Variable | Mean | Std. Error | 95% Confidence Interval | |
|  |  |  | Lower Bound | Upper Bound |
| Fatal_rate | 5.451^a^ | 1.493 | 2.125 | 8.778 |
| Nonfatal_rate | 467.108^a^ | 236.998 | -60.956 | 995.173 |
| a. Covariates appearing in the model are evaluated at the following values: Inspector_ratio = .495000. | | | | |

**Observed * Predicted * Std. Residual Plots**

**General linear multivariate regression with bootstrap-2000 with Confidence Intervals of 95% (PP. 18-24)
Bootstrap**

| **Notes** | | |
| --- | --- | --- |
| Output Created | | 24-FEB-2026 09:35:58 |
| Comments | |  |
| Input | Data | E:\Group 3 and 4 - Feb 24\Groups 3 and 4 - Descriptive and regression analysis.sav |
|  | Active Dataset | DataSet0 |
|  | Filter | <none> |
|  | Weight | <none> |
|  | Split File | <none> |
|  | N of Rows in Working Data File | 12 |
| Syntax | | BOOTSTRAP /SAMPLING METHOD=SIMPLE /VARIABLES TARGET=Fatal_rate Nonfatal_rate INPUT= Inspector_ratio /CRITERIA CILEVEL=95 CITYPE=PERCENTILE NSAMPLES=2000 /MISSING USERMISSING=EXCLUDE. |
| Resources | Processor Time | 00:00:00.02 |
|  | Elapsed Time | 00:00:00.02 |

| **Bootstrap Specifications** | |
| --- | --- |
| Sampling Method | Simple |
| Number of Samples | 2000 |
| Confidence Interval Level | 95.0% |
| Confidence Interval Type | Percentile |

**General Linear Model**

| **Notes** | | |
| --- | --- | --- |
| Output Created | | 24-FEB-2026 09:35:58 |
| Comments | |  |
| Input | Data | E:\Group 3 and 4 - Feb 24\Groups 3 and 4 - Descriptive and regression analysis.sav |
|  | Active Dataset | DataSet0 |
|  | Filter | <none> |
|  | Weight | <none> |
|  | Split File | <none> |
|  | N of Rows in Working Data File | 24012 |
| Missing Value Handling | Definition of Missing | User-defined missing values are treated as missing. |
|  | Cases Used | Statistics are based on all cases with valid data for all variables in the model. |
| Syntax | | GLM Fatal_rate Nonfatal_rate WITH Inspector_ratio /METHOD=SSTYPE(3) /INTERCEPT=INCLUDE /SAVE=PRED SEPRED RESID ZRESID COOK LEVER /EMMEANS=TABLES(OVERALL) WITH(Inspector_ratio=MEAN) /PRINT=DESCRIPTIVE ETASQ OPOWER PARAMETER TEST(SSCP) RSSCP HOMOGENEITY LOF /PLOT=SPREADLEVEL RESIDUALS /CRITERIA=ALPHA(.05) /DESIGN=Inspector_ratio. |
| Resources | Processor Time | 00:00:25.98 |
|  | Elapsed Time | 00:00:25.65 |

| **Warnings** |
| --- |
| The HOMOGENEITY specification in the PRINT subcommand will be ignored because there are no between-subjects factors. |
| The SPREADLEVEL specification has been found in the PLOT subcommand, but the model includes no factors. The spread versus level plot will not be produced. |
| This command is trying to save new variables while bootstrapping is in effect. The new variables will be lost. Execution of this command stops. |

| **Descriptive Statistics** | | | |
| --- | --- | --- | --- |
|  | Mean | Std. Deviation | N |
| Fatal_rate | 5.451333 | 4.9583299 | 12 |
| Nonfatal_rate | 467.108333 | 783.1462565 | 12 |

| **Bartlett's Test of Sphericity**^a^ | |
| --- | --- |
| Likelihood Ratio | .000 |
| Approx. Chi-Square | 81.090 |
| df | 2 |
| Sig. | <.001 |
| Tests the null hypothesis that the residual covariance matrix is proportional to an identity matrix.^a^  a. Design: Intercept + Inspector_ratio | |

| **Multivariate Tests**^a^ | | | | | | |
| --- | --- | --- | --- | --- | --- | --- |
| Effect | | Value | F | Hypothesis df | Error df | Sig. |
| Intercept | Pillai's Trace | .174 | .947^b^ | 2.000 | 9.000 | .423 |
|  | Wilks' Lambda | .826 | .947^b^ | 2.000 | 9.000 | .423 |
|  | Hotelling's Trace | .210 | .947^b^ | 2.000 | 9.000 | .423 |
|  | Roy's Largest Root | .210 | .947^b^ | 2.000 | 9.000 | .423 |
| Inspector_ratio | Pillai's Trace | .012 | .053^b^ | 2.000 | 9.000 | .949 |
|  | Wilks' Lambda | .988 | .053^b^ | 2.000 | 9.000 | .949 |
|  | Hotelling's Trace | .012 | .053^b^ | 2.000 | 9.000 | .949 |
|  | Roy's Largest Root | .012 | .053^b^ | 2.000 | 9.000 | .949 |

| **Multivariate Tests**^a^ | | | | |
| --- | --- | --- | --- | --- |
| Effect | | Partial Eta Squared | Noncent. Parameter | Observed Power^c^ |
| Intercept | Pillai's Trace | .174 | 1.894 | .166 |
|  | Wilks' Lambda | .174 | 1.894 | .166 |
|  | Hotelling's Trace | .174 | 1.894 | .166 |
|  | Roy's Largest Root | .174 | 1.894 | .166 |
| Inspector_ratio | Pillai's Trace | .012 | .106 | .056 |
|  | Wilks' Lambda | .012 | .106 | .056 |
|  | Hotelling's Trace | .012 | .106 | .056 |
|  | Roy's Largest Root | .012 | .106 | .056 |
| a. Design: Intercept + Inspector_ratio | | | | |
| b. Exact statistic | | | | |
| c. Computed using alpha = .05 | | | | |

| **Tests of Between-Subjects Effects** | | | | | | |
| --- | --- | --- | --- | --- | --- | --- |
| Source | Dependent Variable | Type III Sum of Squares | df | Mean Square | F | Sig. |
| Corrected Model | Fatal_rate | 3.011^a^ | 1 | 3.011 | .113 | .744 |
|  | Nonfatal_rate | 6338.992^b^ | 1 | 6338.992 | .009 | .925 |
| Intercept | Fatal_rate | 55.383 | 1 | 55.383 | 2.071 | .181 |
|  | Nonfatal_rate | 487859.801 | 1 | 487859.801 | .724 | .415 |
| Inspector_ratio | Fatal_rate | 3.011 | 1 | 3.011 | .113 | .744 |
|  | Nonfatal_rate | 6338.992 | 1 | 6338.992 | .009 | .925 |
| Error | Fatal_rate | 267.424 | 10 | 26.742 |  |  |
|  | Nonfatal_rate | 6740159.659 | 10 | 674015.966 |  |  |
| Total | Fatal_rate | 627.040 | 12 |  |  |  |
|  | Nonfatal_rate | 9364780.991 | 12 |  |  |  |
| Corrected Total | Fatal_rate | 270.435 | 11 |  |  |  |
|  | Nonfatal_rate | 6746498.651 | 11 |  |  |  |

| **Tests of Between-Subjects Effects** | | | | |
| --- | --- | --- | --- | --- |
| Source | Dependent Variable | Partial Eta Squared | Noncent. Parameter | Observed Power^c^ |
| Corrected Model | Fatal_rate | .011 | .113 | .061 |
|  | Nonfatal_rate | .001 | .009 | .051 |
| Intercept | Fatal_rate | .172 | 2.071 | .256 |
|  | Nonfatal_rate | .067 | .724 | .120 |
| Inspector_ratio | Fatal_rate | .011 | .113 | .061 |
|  | Nonfatal_rate | .001 | .009 | .051 |
| Error | Fatal_rate |  |  |  |
|  | Nonfatal_rate |  |  |  |
| Total | Fatal_rate |  |  |  |
|  | Nonfatal_rate |  |  |  |
| Corrected Total | Fatal_rate |  |  |  |
|  | Nonfatal_rate |  |  |  |
| a. R Squared = .011 (Adjusted R Squared = -.088) | | | | |
| b. R Squared = .001 (Adjusted R Squared = -.099) | | | | |
| c. Computed using alpha = .05 | | | | |

| **Parameter Estimates** | | | | | | |
| --- | --- | --- | --- | --- | --- | --- |
| Dependent Variable | Parameter | B | Std. Error | t | Sig. | 95% Confidence Interval |
|  |  |  |  |  |  | Lower Bound |
| Fatal_rate | Intercept | 4.523 | 3.143 | 1.439 | .181 | -2.480 |
|  | Inspector_ratio | 1.875 | 5.588 | .336 | .744 | -10.575 |
| Nonfatal_rate | Intercept | 424.524 | 498.987 | .851 | .415 | -687.290 |
|  | Inspector_ratio | 86.029 | 887.098 | .097 | .925 | -1890.547 |

| **Parameter Estimates** | | | | | |
| --- | --- | --- | --- | --- | --- |
| Dependent Variable | Parameter | 95% Confidence Interval | Partial Eta Squared | Noncent. Parameter | Observed Power^a^ |
|  |  | Upper Bound |  |  |  |
| Fatal_rate | Intercept | 11.526 | .172 | 1.439 | .256 |
|  | Inspector_ratio | 14.325 | .011 | .336 | .061 |
| Nonfatal_rate | Intercept | 1536.337 | .067 | .851 | .120 |
|  | Inspector_ratio | 2062.606 | .001 | .097 | .051 |
| a. Computed using alpha = .05 | | | | | |

| **Bootstrap for Parameter Estimates** | | | | | | |
| --- | --- | --- | --- | --- | --- | --- |
| Dependent Variable | Parameter | B | Bootstrap^a^ | | | |
|  |  |  | Bias | Std. Error | Sig. (2-tailed) | 95% Confidence Interval |
|  |  |  |  |  |  | Lower |
| Fatal_rate | Intercept | 4.523 | -.014 | 2.519 | .083 | .702 |
|  | Inspector_ratio | 1.875 | .208 | 4.513 | .673 | -7.419 |
| Nonfatal_rate | Intercept | 424.524 | 17.262 | 430.825 | .342 | -213.115 |
|  | Inspector_ratio | 86.029 | 12.842 | 833.376 | .918 | -1567.512 |

| **Bootstrap for Parameter Estimates** | | |
| --- | --- | --- |
| Dependent Variable | Parameter | Bootstrap |
|  |  | 95% Confidence Interval |
|  |  | Upper |
| Fatal_rate | Intercept | 10.467 |
|  | Inspector_ratio | 11.215 |
| Nonfatal_rate | Intercept | 1527.978 |
|  | Inspector_ratio | 2020.185 |
| a. Unless otherwise noted, bootstrap results are based on 2000 bootstrap samples | | |

| **Between-Subjects SSCP Matrix** | | | | |
| --- | --- | --- | --- | --- |
|  | | | Fatal_rate | Nonfatal_rate |
| Hypothesis | Intercept | Fatal_rate | 55.383 | 5197.998 |
|  |  | Nonfatal_rate | 5197.998 | 487859.801 |
|  | Inspector_ratio | Fatal_rate | 3.011 | 138.163 |
|  |  | Nonfatal_rate | 138.163 | 6338.992 |
| Error | | Fatal_rate | 267.424 | 20364.095 |
|  |  | Nonfatal_rate | 20364.095 | 6740159.659 |
| Based on Type III Sum of Squares | | | | |

| **Residual SSCP Matrix** | | | |
| --- | --- | --- | --- |
|  | | Fatal_rate | Nonfatal_rate |
| Sum-of-Squares and Cross-Products | Fatal_rate | 267.424 | 20364.095 |
|  | Nonfatal_rate | 20364.095 | 6740159.659 |
| Covariance | Fatal_rate | 26.742 | 2036.410 |
|  | Nonfatal_rate | 2036.410 | 674015.966 |
| Correlation | Fatal_rate | 1.000 | .480 |
|  | Nonfatal_rate | .480 | 1.000 |
| Based on Type III Sum of Squares | | | |

**Lack of Fit**

| **Multivariate Tests** | | | | | |
| --- | --- | --- | --- | --- | --- |
| Dependent Variables | | Value | F | Hypothesis df | Error df |
| Fatal_rate, Nonfatal_rate | Pillai's Trace | .^a^ | . | . | . |
|  | Wilks' Lambda | .^a^ | . | . | . |
|  | Hotelling's Trace | .^a^ | . | . | . |
|  | Roy's Largest Root | .^a^ | . | . | . |
| Fatal_rate | Pillai's Trace | .^a^ | . | . | . |
|  | Wilks' Lambda | .^a^ | . | . | . |
|  | Hotelling's Trace | .^a^ | . | . | . |
|  | Roy's Largest Root | .^a^ | . | . | . |
| Nonfatal_rate | Pillai's Trace | .^a^ | . | . | . |
|  | Wilks' Lambda | .^a^ | . | . | . |
|  | Hotelling's Trace | .^a^ | . | . | . |
|  | Roy's Largest Root | .^a^ | . | . | . |

| **Multivariate Tests** | | | | |
| --- | --- | --- | --- | --- |
| Dependent Variables | | Sig. | Partial Eta Squared | Noncent. Parameter |
| Fatal_rate, Nonfatal_rate | Pillai's Trace | . | . | . |
|  | Wilks' Lambda | . | . | . |
|  | Hotelling's Trace | . | . | . |
|  | Roy's Largest Root | . | . | . |
| Fatal_rate | Pillai's Trace | . | . | . |
|  | Wilks' Lambda | . | . | . |
|  | Hotelling's Trace | . | . | . |
|  | Roy's Largest Root | . | . | . |
| Nonfatal_rate | Pillai's Trace | . | . | . |
|  | Wilks' Lambda | . | . | . |
|  | Hotelling's Trace | . | . | . |
|  | Roy's Largest Root | . | . | . |

| **Multivariate Tests** | | |
| --- | --- | --- |
| Dependent Variables | | Observed Power^b^ |
| Fatal_rate, Nonfatal_rate | Pillai's Trace | . |
|  | Wilks' Lambda | . |
|  | Hotelling's Trace | . |
|  | Roy's Largest Root | . |
| Fatal_rate | Pillai's Trace | . |
|  | Wilks' Lambda | . |
|  | Hotelling's Trace | . |
|  | Roy's Largest Root | . |
| Nonfatal_rate | Pillai's Trace | . |
|  | Wilks' Lambda | . |
|  | Hotelling's Trace | . |
|  | Roy's Largest Root | . |
| a. The pure error sub-matrix corresponding to the selected dependent variables is a zero matrix. | | |
| b. Computed using alpha = .05 | | |

| **Univariate Tests** | | | | | | |
| --- | --- | --- | --- | --- | --- | --- |
| Dependent Variable | Source | Sum of Squares | df | Mean Square | F | Sig. |
| Fatal_rate | Lack of Fit | 267.424 | 10 | 26.742 | . | . |
|  | Pure Error | .000 | 0 | . |  |  |
| Nonfatal_rate | Lack of Fit | 6740159.659 | 10 | 674015.966 | . | . |
|  | Pure Error | .000 | 0 | . |  |  |

| **Univariate Tests** | | | | |
| --- | --- | --- | --- | --- |
| Dependent Variable | Source | Partial Eta Squared | Noncent. Parameter | Observed Power^a^ |
| Fatal_rate | Lack of Fit | 1.000 | . | . |
|  | Pure Error |  |  |  |
| Nonfatal_rate | Lack of Fit | 1.000 | . | . |
|  | Pure Error |  |  |  |
| a. Computed using alpha = .05 | | | | |

| **SSCP Matrix** | | | |
| --- | --- | --- | --- |
|  | | Fatal_rate | Nonfatal_rate |
| Lack of Fit | Fatal_rate | 267.424 | 20364.095 |
|  | Nonfatal_rate | 20364.095 | 6740159.659 |
| Pure Error | Fatal_rate | .000 | .000 |
|  | Nonfatal_rate | .000 | .000 |

**Estimated Marginal Means**

| **Grand Mean** | | | | |
| --- | --- | --- | --- | --- |
| Dependent Variable | Mean | Std. Error | 95% Confidence Interval | |
|  |  |  | Lower Bound | Upper Bound |
| Fatal_rate | 5.451^a^ | 1.493 | 2.125 | 8.778 |
| Nonfatal_rate | 467.108^a^ | 236.998 | -60.956 | 995.173 |
| a. Covariates appearing in the model are evaluated at the following values: Inspector_ratio = .495000. | | | | |

**Continued.**

**Descriptive statistics with log-transformation of dependent variables (PP. 25-33)**

**Explore**

| **Notes** | | |
| --- | --- | --- |
| Output Created | | 24-FEB-2026 09:38:35 |
| Comments | |  |
| Input | Data | E:\Group 3 and 4 - Feb 24\Groups 3 and 4 - Descriptive and regression analysis.sav |
|  | Active Dataset | DataSet0 |
|  | Filter | <none> |
|  | Weight | <none> |
|  | Split File | <none> |
|  | N of Rows in Working Data File | 12 |
| Missing Value Handling | Definition of Missing | User-defined missing values for dependent variables are treated as missing. |
|  | Cases Used | Statistics are based on cases with no missing values for any dependent variable or factor used. |
| Syntax | | EXAMINE VARIABLES=Inspector_ratio Log_fatalrate Log_nonfatalrate /PLOT BOXPLOT HISTOGRAM NPPLOT /COMPARE GROUPS /STATISTICS DESCRIPTIVES EXTREME /CINTERVAL 95 /MISSING LISTWISE /NOTOTAL. |
| Resources | Processor Time | 00:00:01.64 |
|  | Elapsed Time | 00:00:01.26 |

| **Case Processing Summary** | | | | | | |
| --- | --- | --- | --- | --- | --- | --- |
|  | Cases | | | | | |
|  | Valid | | Missing | | Total | |
|  | N | Percent | N | Percent | N | Percent |
| Inspector_ratio | 12 | 100.0% | 0 | 0.0% | 12 | 100.0% |
| Log_fatalrate | 12 | 100.0% | 0 | 0.0% | 12 | 100.0% |
| Log_nonfatalrate | 12 | 100.0% | 0 | 0.0% | 12 | 100.0% |

| **Descriptives** | | | | |
| --- | --- | --- | --- | --- |
|  | | | Statistic | Std. Error |
| Inspector_ratio | Mean | | .495000 | .0805521 |
|  | 95% Confidence Interval for Mean | Lower Bound | .317706 |  |
|  |  | Upper Bound | .672294 |  |
|  | 5% Trimmed Mean | | .495000 |  |
|  | Median | | .515000 |  |
|  | Variance | | .078 |  |
|  | Std. Deviation | | .2790406 |  |
|  | Minimum | | .0700 |  |
|  | Maximum | | .9200 |  |
|  | Range | | .8500 |  |
|  | Interquartile Range | | .4925 |  |
|  | Skewness | | -.037 | .637 |
|  | Kurtosis | | -1.132 | 1.232 |
| Log_fatalrate | Mean | | .4855 | .17366 |
|  | 95% Confidence Interval for Mean | Lower Bound | .1033 |  |
|  |  | Upper Bound | .8678 |  |
|  | 5% Trimmed Mean | | .5196 |  |
|  | Median | | .5999 |  |
|  | Variance | | .362 |  |
|  | Std. Deviation | | .60159 |  |
|  | Minimum | | -.85 |  |
|  | Maximum | | 1.21 |  |
|  | Range | | 2.07 |  |
|  | Interquartile Range | | .74 |  |
|  | Skewness | | -1.140 | .637 |
|  | Kurtosis | | .990 | 1.232 |
| Log_nonfatalrate | Mean | | 2.0719 | .23081 |
|  | 95% Confidence Interval for Mean | Lower Bound | 1.5639 |  |
|  |  | Upper Bound | 2.5800 |  |
|  | 5% Trimmed Mean | | 2.0597 |  |
|  | Median | | 2.0447 |  |
|  | Variance | | .639 |  |
|  | Std. Deviation | | .79955 |  |
|  | Minimum | | 1.00 |  |
|  | Maximum | | 3.36 |  |
|  | Range | | 2.36 |  |
|  | Interquartile Range | | 1.28 |  |
|  | Skewness | | .283 | .637 |
|  | Kurtosis | | -1.000 | 1.232 |

| **Extreme Values** | | | | |
| --- | --- | --- | --- | --- |
|  | | | Case Number | Value |
| Inspector_ratio | Highest | 1 | 5 | .9200 |
|  |  | 2 | 3 | .8500 |
|  |  | 3 | 8 | .7500 |
|  |  | 4 | 2 | .6900 |
|  |  | 5 | 7 | .5800 |
|  | Lowest | 1 | 4 | .0700 |
|  |  | 2 | 6 | .1300 |
|  |  | 3 | 9 | .2200 |
|  |  | 4 | 11 | .3100 |
|  |  | 5 | 12 | .3900 |
| Log_fatalrate | Highest | 1 | 12 | 1.21 |
|  |  | 2 | 2 | 1.12 |
|  |  | 3 | 8 | .88 |
|  |  | 4 | 3 | .81 |
|  |  | 5 | 10 | .78 |
|  | Lowest | 1 | 1 | -.85 |
|  |  | 2 | 7 | -.32 |
|  |  | 3 | 5 | .00 |
|  |  | 4 | 4 | .50 |
|  |  | 5 | 6 | .51 |
| Log_nonfatalrate | Highest | 1 | 2 | 3.36 |
|  |  | 2 | 11 | 3.28 |
|  |  | 3 | 12 | 2.68 |
|  |  | 4 | 10 | 2.51 |
|  |  | 5 | 6 | 2.36 |
|  | Lowest | 1 | 9 | 1.00 |
|  |  | 2 | 4 | 1.07 |
|  |  | 3 | 7 | 1.33 |
|  |  | 4 | 1 | 1.46 |
|  |  | 5 | 3 | 1.73 |

| **Tests of Normality** | | | | | | |
| --- | --- | --- | --- | --- | --- | --- |
|  | Kolmogorov-Smirnov^a^ | | | Shapiro-Wilk | | |
|  | Statistic | df | Sig. | Statistic | df | Sig. |
| Inspector_ratio | .092 | 12 | .200^*^ | .966 | 12 | .868 |
| Log_fatalrate | .258 | 12 | .026 | .900 | 12 | .158 |
| Log_nonfatalrate | .112 | 12 | .200^*^ | .948 | 12 | .605 |
| *. This is a lower bound of the true significance. | | | | | | |
| a. Lilliefors Significance Correction | | | | | | |

**Inspector_ratio**

**Log_fatalrate**

**Log_nonfatalrate**

**Log-general linear multivariate regression with bootstrap-2000 with Confidence Intervals of 95% (PP. 34-40)**

**Bootstrap**

| **Notes** | | |
| --- | --- | --- |
| Output Created | | 24-FEB-2026 09:43:08 |
| Comments | |  |
| Input | Data | E:\Group 3 and 4 - Feb 24\Groups 3 and 4 - Descriptive and regression analysis.sav |
|  | Active Dataset | DataSet0 |
|  | Filter | <none> |
|  | Weight | <none> |
|  | Split File | <none> |
|  | N of Rows in Working Data File | 12 |
| Syntax | | BOOTSTRAP /SAMPLING METHOD=SIMPLE /VARIABLES TARGET=Log_fatalrate Log_nonfatalrate INPUT= Inspector_ratio /CRITERIA CILEVEL=95 CITYPE=PERCENTILE NSAMPLES=2000 /MISSING USERMISSING=EXCLUDE. |
| Resources | Processor Time | 00:00:00.02 |
|  | Elapsed Time | 00:00:00.01 |

| **Bootstrap Specifications** | |
| --- | --- |
| Sampling Method | Simple |
| Number of Samples | 2000 |
| Confidence Interval Level | 95.0% |
| Confidence Interval Type | Percentile |

**General Linear Model**

| **Notes** | | |
| --- | --- | --- |
| Output Created | | 24-FEB-2026 09:43:09 |
| Comments | |  |
| Input | Data | E:\Group 3 and 4 - Feb 24\Groups 3 and 4 - Descriptive and regression analysis.sav |
|  | Active Dataset | DataSet0 |
|  | Filter | <none> |
|  | Weight | <none> |
|  | Split File | <none> |
|  | N of Rows in Working Data File | 24012 |
| Missing Value Handling | Definition of Missing | User-defined missing values are treated as missing. |
|  | Cases Used | Statistics are based on all cases with valid data for all variables in the model. |
| Syntax | | GLM Log_fatalrate Log_nonfatalrate WITH Inspector_ratio /METHOD=SSTYPE(3) /INTERCEPT=INCLUDE /SAVE=PRED SEPRED RESID ZRESID COOK LEVER /EMMEANS=TABLES(OVERALL) WITH(Inspector_ratio=MEAN) /PRINT=DESCRIPTIVE ETASQ OPOWER PARAMETER TEST(SSCP) RSSCP HOMOGENEITY LOF /PLOT=SPREADLEVEL RESIDUALS /CRITERIA=ALPHA(.05) /DESIGN=Inspector_ratio. |
| Resources | Processor Time | 00:00:26.34 |
|  | Elapsed Time | 00:00:26.22 |

| **Warnings** |
| --- |
| The HOMOGENEITY specification in the PRINT subcommand will be ignored because there are no between-subjects factors. |
| The SPREADLEVEL specification has been found in the PLOT subcommand, but the model includes no factors. The spread versus level plot will not be produced. |
| This command is trying to save new variables while bootstrapping is in effect. The new variables will be lost. Execution of this command stops. |

| **Descriptive Statistics** | | | |
| --- | --- | --- | --- |
|  | Mean | Std. Deviation | N |
| Log_fatalrate | .4855 | .60159 | 12 |
| Log_nonfatalrate | 2.0719 | .79955 | 12 |

| **Bartlett's Test of Sphericity**^a^ | |
| --- | --- |
| Likelihood Ratio | .061 |
| Approx. Chi-Square | 4.194 |
| df | 2 |
| Sig. | .123 |
| Tests the null hypothesis that the residual covariance matrix is proportional to an identity matrix.^a^  a. Design: Intercept + Inspector_ratio | |

| **Multivariate Tests**^a^ | | | | | | |
| --- | --- | --- | --- | --- | --- | --- |
| Effect | | Value | F | Hypothesis df | Error df | Sig. |
| Intercept | Pillai's Trace | .590 | 6.481^b^ | 2.000 | 9.000 | .018 |
|  | Wilks' Lambda | .410 | 6.481^b^ | 2.000 | 9.000 | .018 |
|  | Hotelling's Trace | 1.440 | 6.481^b^ | 2.000 | 9.000 | .018 |
|  | Roy's Largest Root | 1.440 | 6.481^b^ | 2.000 | 9.000 | .018 |
| Inspector_ratio | Pillai's Trace | .040 | .187^b^ | 2.000 | 9.000 | .832 |
|  | Wilks' Lambda | .960 | .187^b^ | 2.000 | 9.000 | .832 |
|  | Hotelling's Trace | .042 | .187^b^ | 2.000 | 9.000 | .832 |
|  | Roy's Largest Root | .042 | .187^b^ | 2.000 | 9.000 | .832 |

| **Multivariate Tests**^a^ | | | | |
| --- | --- | --- | --- | --- |
| Effect | | Partial Eta Squared | Noncent. Parameter | Observed Power^c^ |
| Intercept | Pillai's Trace | .590 | 12.962 | .777 |
|  | Wilks' Lambda | .590 | 12.962 | .777 |
|  | Hotelling's Trace | .590 | 12.962 | .777 |
|  | Roy's Largest Root | .590 | 12.962 | .777 |
| Inspector_ratio | Pillai's Trace | .040 | .375 | .071 |
|  | Wilks' Lambda | .040 | .375 | .071 |
|  | Hotelling's Trace | .040 | .375 | .071 |
|  | Roy's Largest Root | .040 | .375 | .071 |
| a. Design: Intercept + Inspector_ratio | | | | |
| b. Exact statistic | | | | |
| c. Computed using alpha = .05 | | | | |

| **Tests of Between-Subjects Effects** | | | | | | |
| --- | --- | --- | --- | --- | --- | --- |
| Source | Dependent Variable | Type III Sum of Squares | df | Mean Square | F | Sig. |
| Corrected Model | Log_fatalrate | .001^a^ | 1 | .001 | .001 | .971 |
|  | Log_nonfatalrate | .177^b^ | 1 | .177 | .259 | .622 |
| Intercept | Log_fatalrate | .671 | 1 | .671 | 1.686 | .223 |
|  | Log_nonfatalrate | 9.232 | 1 | 9.232 | 13.468 | .004 |
| Inspector_ratio | Log_fatalrate | .001 | 1 | .001 | .001 | .971 |
|  | Log_nonfatalrate | .177 | 1 | .177 | .259 | .622 |
| Error | Log_fatalrate | 3.980 | 10 | .398 |  |  |
|  | Log_nonfatalrate | 6.855 | 10 | .685 |  |  |
| Total | Log_fatalrate | 6.810 | 12 |  |  |  |
|  | Log_nonfatalrate | 58.548 | 12 |  |  |  |
| Corrected Total | Log_fatalrate | 3.981 | 11 |  |  |  |
|  | Log_nonfatalrate | 7.032 | 11 |  |  |  |

| **Tests of Between-Subjects Effects** | | | | |
| --- | --- | --- | --- | --- |
| Source | Dependent Variable | Partial Eta Squared | Noncent. Parameter | Observed Power^c^ |
| Corrected Model | Log_fatalrate | .000 | .001 | .050 |
|  | Log_nonfatalrate | .025 | .259 | .075 |
| Intercept | Log_fatalrate | .144 | 1.686 | .217 |
|  | Log_nonfatalrate | .574 | 13.468 | .910 |
| Inspector_ratio | Log_fatalrate | .000 | .001 | .050 |
|  | Log_nonfatalrate | .025 | .259 | .075 |
| Error | Log_fatalrate |  |  |  |
|  | Log_nonfatalrate |  |  |  |
| Total | Log_fatalrate |  |  |  |
|  | Log_nonfatalrate |  |  |  |
| Corrected Total | Log_fatalrate |  |  |  |
|  | Log_nonfatalrate |  |  |  |
| a. R Squared = .000 (Adjusted R Squared = -.100) | | | | |
| b. R Squared = .025 (Adjusted R Squared = -.072) | | | | |
| c. Computed using alpha = .05 | | | | |

| **Parameter Estimates** | | | | | | |
| --- | --- | --- | --- | --- | --- | --- |
| Dependent Variable | Parameter | B | Std. Error | t | Sig. | 95% Confidence Interval |
|  |  |  |  |  |  | Lower Bound |
| Log_fatalrate | Intercept | .498 | .383 | 1.298 | .223 | -.357 |
|  | Inspector_ratio | -.025 | .682 | -.037 | .971 | -1.544 |
| Log_nonfatalrate | Intercept | 1.847 | .503 | 3.670 | .004 | .726 |
|  | Inspector_ratio | .455 | .895 | .508 | .622 | -1.538 |

| **Parameter Estimates** | | | | | |
| --- | --- | --- | --- | --- | --- |
| Dependent Variable | Parameter | 95% Confidence Interval | Partial Eta Squared | Noncent. Parameter | Observed Power^a^ |
|  |  | Upper Bound |  |  |  |
| Log_fatalrate | Intercept | 1.352 | .144 | 1.298 | .217 |
|  | Inspector_ratio | 1.494 | .000 | .037 | .050 |
| Log_nonfatalrate | Intercept | 2.968 | .574 | 3.670 | .910 |
|  | Inspector_ratio | 2.448 | .025 | .508 | .075 |
| a. Computed using alpha = .05 | | | | | |

| **Bootstrap for Parameter Estimates** | | | | | | |
| --- | --- | --- | --- | --- | --- | --- |
| Dependent Variable | Parameter | B | Bootstrap^a^ | | | |
|  |  |  | Bias | Std. Error | Sig. (2-tailed) | 95% Confidence Interval |
|  |  |  |  |  |  | Lower |
| Log_fatalrate | Intercept | .498 | -.012 | .261 | .047 | -.102 |
|  | Inspector_ratio | -.025 | .025 | .474 | .953 | -.917 |
| Log_nonfatalrate | Intercept | 1.847 | .028 | .522 | .015 | .965 |
|  | Inspector_ratio | .455 | -.004 | .853 | .582 | -1.354 |

| **Bootstrap for Parameter Estimates** | | |
| --- | --- | --- |
| Dependent Variable | Parameter | Bootstrap |
|  |  | 95% Confidence Interval |
|  |  | Upper |
| Log_fatalrate | Intercept | .943 |
|  | Inspector_ratio | .894 |
| Log_nonfatalrate | Intercept | 3.018 |
|  | Inspector_ratio | 2.199 |
| a. Unless otherwise noted, bootstrap results are based on 2000 bootstrap samples | | |

| **Between-Subjects SSCP Matrix** | | | | |
| --- | --- | --- | --- | --- |
|  | | | Log_fatalrate | Log_nonfatalrate |
| Hypothesis | Intercept | Log_fatalrate | .671 | 2.489 |
|  |  | Log_nonfatalrate | 2.489 | 9.232 |
|  | Inspector_ratio | Log_fatalrate | .001 | -.010 |
|  |  | Log_nonfatalrate | -.010 | .177 |
| Error | | Log_fatalrate | 3.980 | 2.978 |
|  |  | Log_nonfatalrate | 2.978 | 6.855 |
| Based on Type III Sum of Squares | | | | |

| **Residual SSCP Matrix** | | | |
| --- | --- | --- | --- |
|  | | Log_fatalrate | Log_nonfatalrate |
| Sum-of-Squares and Cross-Products | Log_fatalrate | 3.980 | 2.978 |
|  | Log_nonfatalrate | 2.978 | 6.855 |
| Covariance | Log_fatalrate | .398 | .298 |
|  | Log_nonfatalrate | .298 | .685 |
| Correlation | Log_fatalrate | 1.000 | .570 |
|  | Log_nonfatalrate | .570 | 1.000 |
| Based on Type III Sum of Squares | | | |

**Lack of Fit**

| **Multivariate Tests** | | | | | |
| --- | --- | --- | --- | --- | --- |
| Dependent Variables | | Value | F | Hypothesis df | Error df |
| Log_fatalrate, Log_nonfatalrate | Pillai's Trace | .^a^ | . | . | . |
|  | Wilks' Lambda | .^a^ | . | . | . |
|  | Hotelling's Trace | .^a^ | . | . | . |
|  | Roy's Largest Root | .^a^ | . | . | . |
| Log_fatalrate | Pillai's Trace | .^a^ | . | . | . |
|  | Wilks' Lambda | .^a^ | . | . | . |
|  | Hotelling's Trace | .^a^ | . | . | . |
|  | Roy's Largest Root | .^a^ | . | . | . |
| Log_nonfatalrate | Pillai's Trace | .^a^ | . | . | . |
|  | Wilks' Lambda | .^a^ | . | . | . |
|  | Hotelling's Trace | .^a^ | . | . | . |
|  | Roy's Largest Root | .^a^ | . | . | . |

| **Multivariate Tests** | | | | |
| --- | --- | --- | --- | --- |
| Dependent Variables | | Sig. | Partial Eta Squared | Noncent. Parameter |
| Log_fatalrate, Log_nonfatalrate | Pillai's Trace | . | . | . |
|  | Wilks' Lambda | . | . | . |
|  | Hotelling's Trace | . | . | . |
|  | Roy's Largest Root | . | . | . |
| Log_fatalrate | Pillai's Trace | . | . | . |
|  | Wilks' Lambda | . | . | . |
|  | Hotelling's Trace | . | . | . |
|  | Roy's Largest Root | . | . | . |
| Log_nonfatalrate | Pillai's Trace | . | . | . |
|  | Wilks' Lambda | . | . | . |
|  | Hotelling's Trace | . | . | . |
|  | Roy's Largest Root | . | . | . |

| **Multivariate Tests** | | |
| --- | --- | --- |
| Dependent Variables | | Observed Power^b^ |
| Log_fatalrate, Log_nonfatalrate | Pillai's Trace | . |
|  | Wilks' Lambda | . |
|  | Hotelling's Trace | . |
|  | Roy's Largest Root | . |
| Log_fatalrate | Pillai's Trace | . |
|  | Wilks' Lambda | . |
|  | Hotelling's Trace | . |
|  | Roy's Largest Root | . |
| Log_nonfatalrate | Pillai's Trace | . |
|  | Wilks' Lambda | . |
|  | Hotelling's Trace | . |
|  | Roy's Largest Root | . |
| a. The pure error sub-matrix corresponding to the selected dependent variables is a zero matrix. | | |
| b. Computed using alpha = .05 | | |

| **Univariate Tests** | | | | | | |
| --- | --- | --- | --- | --- | --- | --- |
| Dependent Variable | Source | Sum of Squares | df | Mean Square | F | Sig. |
| Log_fatalrate | Lack of Fit | 3.980 | 10 | .398 | . | . |
|  | Pure Error | .000 | 0 | . |  |  |
| Log_nonfatalrate | Lack of Fit | 6.855 | 10 | .685 | . | . |
|  | Pure Error | .000 | 0 | . |  |  |

| **Univariate Tests** | | | | |
| --- | --- | --- | --- | --- |
| Dependent Variable | Source | Partial Eta Squared | Noncent. Parameter | Observed Power^a^ |
| Log_fatalrate | Lack of Fit | 1.000 | . | . |
|  | Pure Error |  |  |  |
| Log_nonfatalrate | Lack of Fit | 1.000 | . | . |
|  | Pure Error |  |  |  |
| a. Computed using alpha = .05 | | | | |

| **SSCP Matrix** | | | |
| --- | --- | --- | --- |
|  | | Log_fatalrate | Log_nonfatalrate |
| Lack of Fit | Log_fatalrate | 3.980 | 2.978 |
|  | Log_nonfatalrate | 2.978 | 6.855 |
| Pure Error | Log_fatalrate | .000 | .000 |
|  | Log_nonfatalrate | .000 | .000 |

**Estimated Marginal Means**

| **Grand Mean** | | | | |
| --- | --- | --- | --- | --- |
| Dependent Variable | Mean | Std. Error | 95% Confidence Interval | |
|  |  |  | Lower Bound | Upper Bound |
| Log_fatalrate | .486^a^ | .182 | .080 | .891 |
| Log_nonfatalrate | 2.072^a^ | .239 | 1.539 | 2.604 |
| a. Covariates appearing in the model are evaluated at the following values: Inspector_ratio = .495000. | | | | |

**Continued.**

**Log-general linear multivariate regression analysis (PP. 41-47)**

| **Notes** | | |
| --- | --- | --- |
| Output Created | | 24-FEB-2026 09:45:39 |
| Comments | |  |
| Input | Data | E:\Group 3 and 4 - Feb 24\Groups 3 and 4 - Descriptive and regression analysis.sav |
|  | Active Dataset | DataSet0 |
|  | Filter | <none> |
|  | Weight | <none> |
|  | Split File | <none> |
|  | N of Rows in Working Data File | 12 |
| Missing Value Handling | Definition of Missing | User-defined missing values are treated as missing. |
|  | Cases Used | Statistics are based on all cases with valid data for all variables in the model. |
| Syntax | | GLM Log_fatalrate Log_nonfatalrate WITH Inspector_ratio /METHOD=SSTYPE(3) /INTERCEPT=INCLUDE /SAVE=PRED SEPRED RESID ZRESID COOK LEVER /EMMEANS=TABLES(OVERALL) WITH(Inspector_ratio=MEAN) /PRINT=DESCRIPTIVE ETASQ OPOWER PARAMETER TEST(SSCP) RSSCP HOMOGENEITY LOF /PLOT=SPREADLEVEL RESIDUALS /CRITERIA=ALPHA(.05) /DESIGN=Inspector_ratio. |
| Resources | Processor Time | 00:00:00.34 |
|  | Elapsed Time | 00:00:00.26 |
| Variables Created or Modified | PRE_3 | Predicted Value for Log_fatalrate |
|  | PRE_4 | Predicted Value for Log_nonfatalrate |
|  | SEP_3 | Standard Error of Predicted Value for Log_fatalrate |
|  | SEP_4 | Standard Error of Predicted Value for Log_nonfatalrate |
|  | RES_3 | Residual for Log_fatalrate |
|  | RES_4 | Residual for Log_nonfatalrate |
|  | ZRE_3 | Standardized Residual for Log_fatalrate |
|  | ZRE_4 | Standardized Residual for Log_nonfatalrate |
|  | COO_3 | Cook's Distance for Log_fatalrate |
|  | COO_4 | Cook's Distance for Log_nonfatalrate |
|  | LEV_3 | Uncentered Leverage Value for Log_fatalrate |
|  | LEV_4 | Uncentered Leverage Value for Log_nonfatalrate |

| **Warnings** |
| --- |
| The HOMOGENEITY specification in the PRINT subcommand will be ignored because there are no between-subjects factors. |
| The SPREADLEVEL specification has been found in the PLOT subcommand, but the model includes no factors. The spread versus level plot will not be produced. |

| **Descriptive Statistics** | | | |
| --- | --- | --- | --- |
|  | Mean | Std. Deviation | N |
| Log_fatalrate | .4855 | .60159 | 12 |
| Log_nonfatalrate | 2.0719 | .79955 | 12 |

| **Bartlett's Test of Sphericity**^a^ | |
| --- | --- |
| Likelihood Ratio | .061 |
| Approx. Chi-Square | 4.194 |
| df | 2 |
| Sig. | .123 |
| Tests the null hypothesis that the residual covariance matrix is proportional to an identity matrix.^a^  a. Design: Intercept + Inspector_ratio | |

| **Multivariate Tests**^a^ | | | | | | |
| --- | --- | --- | --- | --- | --- | --- |
| Effect | | Value | F | Hypothesis df | Error df | Sig. |
| Intercept | Pillai's Trace | .590 | 6.481^b^ | 2.000 | 9.000 | .018 |
|  | Wilks' Lambda | .410 | 6.481^b^ | 2.000 | 9.000 | .018 |
|  | Hotelling's Trace | 1.440 | 6.481^b^ | 2.000 | 9.000 | .018 |
|  | Roy's Largest Root | 1.440 | 6.481^b^ | 2.000 | 9.000 | .018 |
| Inspector_ratio | Pillai's Trace | .040 | .187^b^ | 2.000 | 9.000 | .832 |
|  | Wilks' Lambda | .960 | .187^b^ | 2.000 | 9.000 | .832 |
|  | Hotelling's Trace | .042 | .187^b^ | 2.000 | 9.000 | .832 |
|  | Roy's Largest Root | .042 | .187^b^ | 2.000 | 9.000 | .832 |

| **Multivariate Tests**^a^ | | | | |
| --- | --- | --- | --- | --- |
| Effect | | Partial Eta Squared | Noncent. Parameter | Observed Power^c^ |
| Intercept | Pillai's Trace | .590 | 12.962 | .777 |
|  | Wilks' Lambda | .590 | 12.962 | .777 |
|  | Hotelling's Trace | .590 | 12.962 | .777 |
|  | Roy's Largest Root | .590 | 12.962 | .777 |
| Inspector_ratio | Pillai's Trace | .040 | .375 | .071 |
|  | Wilks' Lambda | .040 | .375 | .071 |
|  | Hotelling's Trace | .040 | .375 | .071 |
|  | Roy's Largest Root | .040 | .375 | .071 |
| a. Design: Intercept + Inspector_ratio | | | | |
| b. Exact statistic | | | | |
| c. Computed using alpha = .05 | | | | |

| **Tests of Between-Subjects Effects** | | | | | | |
| --- | --- | --- | --- | --- | --- | --- |
| Source | Dependent Variable | Type III Sum of Squares | df | Mean Square | F | Sig. |
| Corrected Model | Log_fatalrate | .001^a^ | 1 | .001 | .001 | .971 |
|  | Log_nonfatalrate | .177^b^ | 1 | .177 | .259 | .622 |
| Intercept | Log_fatalrate | .671 | 1 | .671 | 1.686 | .223 |
|  | Log_nonfatalrate | 9.232 | 1 | 9.232 | 13.468 | .004 |
| Inspector_ratio | Log_fatalrate | .001 | 1 | .001 | .001 | .971 |
|  | Log_nonfatalrate | .177 | 1 | .177 | .259 | .622 |
| Error | Log_fatalrate | 3.980 | 10 | .398 |  |  |
|  | Log_nonfatalrate | 6.855 | 10 | .685 |  |  |
| Total | Log_fatalrate | 6.810 | 12 |  |  |  |
|  | Log_nonfatalrate | 58.548 | 12 |  |  |  |
| Corrected Total | Log_fatalrate | 3.981 | 11 |  |  |  |
|  | Log_nonfatalrate | 7.032 | 11 |  |  |  |

| **Tests of Between-Subjects Effects** | | | | |
| --- | --- | --- | --- | --- |
| Source | Dependent Variable | Partial Eta Squared | Noncent. Parameter | Observed Power^c^ |
| Corrected Model | Log_fatalrate | .000 | .001 | .050 |
|  | Log_nonfatalrate | .025 | .259 | .075 |
| Intercept | Log_fatalrate | .144 | 1.686 | .217 |
|  | Log_nonfatalrate | .574 | 13.468 | .910 |
| Inspector_ratio | Log_fatalrate | .000 | .001 | .050 |
|  | Log_nonfatalrate | .025 | .259 | .075 |
| Error | Log_fatalrate |  |  |  |
|  | Log_nonfatalrate |  |  |  |
| Total | Log_fatalrate |  |  |  |
|  | Log_nonfatalrate |  |  |  |
| Corrected Total | Log_fatalrate |  |  |  |
|  | Log_nonfatalrate |  |  |  |
| a. R Squared = .000 (Adjusted R Squared = -.100) | | | | |
| b. R Squared = .025 (Adjusted R Squared = -.072) | | | | |
| c. Computed using alpha = .05 | | | | |

| **Parameter Estimates** | | | | | | |
| --- | --- | --- | --- | --- | --- | --- |
| Dependent Variable | Parameter | B | Std. Error | t | Sig. | 95% Confidence Interval |
|  |  |  |  |  |  | Lower Bound |
| Log_fatalrate | Intercept | .498 | .383 | 1.298 | .223 | -.357 |
|  | Inspector_ratio | -.025 | .682 | -.037 | .971 | -1.544 |
| Log_nonfatalrate | Intercept | 1.847 | .503 | 3.670 | .004 | .726 |
|  | Inspector_ratio | .455 | .895 | .508 | .622 | -1.538 |

| **Parameter Estimates** | | | | | |
| --- | --- | --- | --- | --- | --- |
| Dependent Variable | Parameter | 95% Confidence Interval | Partial Eta Squared | Noncent. Parameter | Observed Power^a^ |
|  |  | Upper Bound |  |  |  |
| Log_fatalrate | Intercept | 1.352 | .144 | 1.298 | .217 |
|  | Inspector_ratio | 1.494 | .000 | .037 | .050 |
| Log_nonfatalrate | Intercept | 2.968 | .574 | 3.670 | .910 |
|  | Inspector_ratio | 2.448 | .025 | .508 | .075 |
| a. Computed using alpha = .05 | | | | | |

| **Between-Subjects SSCP Matrix** | | | | |
| --- | --- | --- | --- | --- |
|  | | | Log_fatalrate | Log_nonfatalrate |
| Hypothesis | Intercept | Log_fatalrate | .671 | 2.489 |
|  |  | Log_nonfatalrate | 2.489 | 9.232 |
|  | Inspector_ratio | Log_fatalrate | .001 | -.010 |
|  |  | Log_nonfatalrate | -.010 | .177 |
| Error | | Log_fatalrate | 3.980 | 2.978 |
|  |  | Log_nonfatalrate | 2.978 | 6.855 |
| Based on Type III Sum of Squares | | | | |

| **Residual SSCP Matrix** | | | |
| --- | --- | --- | --- |
|  | | Log_fatalrate | Log_nonfatalrate |
| Sum-of-Squares and Cross-Products | Log_fatalrate | 3.980 | 2.978 |
|  | Log_nonfatalrate | 2.978 | 6.855 |
| Covariance | Log_fatalrate | .398 | .298 |
|  | Log_nonfatalrate | .298 | .685 |
| Correlation | Log_fatalrate | 1.000 | .570 |
|  | Log_nonfatalrate | .570 | 1.000 |
| Based on Type III Sum of Squares | | | |

**Lack of Fit**

| **Multivariate Tests** | | | | | |
| --- | --- | --- | --- | --- | --- |
| Dependent Variables | | Value | F | Hypothesis df | Error df |
| Log_fatalrate, Log_nonfatalrate | Pillai's Trace | .^a^ | . | . | . |
|  | Wilks' Lambda | .^a^ | . | . | . |
|  | Hotelling's Trace | .^a^ | . | . | . |
|  | Roy's Largest Root | .^a^ | . | . | . |
| Log_fatalrate | Pillai's Trace | .^a^ | . | . | . |
|  | Wilks' Lambda | .^a^ | . | . | . |
|  | Hotelling's Trace | .^a^ | . | . | . |
|  | Roy's Largest Root | .^a^ | . | . | . |
| Log_nonfatalrate | Pillai's Trace | .^a^ | . | . | . |
|  | Wilks' Lambda | .^a^ | . | . | . |
|  | Hotelling's Trace | .^a^ | . | . | . |
|  | Roy's Largest Root | .^a^ | . | . | . |

| **Multivariate Tests** | | | | |
| --- | --- | --- | --- | --- |
| Dependent Variables | | Sig. | Partial Eta Squared | Noncent. Parameter |
| Log_fatalrate, Log_nonfatalrate | Pillai's Trace | . | . | . |
|  | Wilks' Lambda | . | . | . |
|  | Hotelling's Trace | . | . | . |
|  | Roy's Largest Root | . | . | . |
| Log_fatalrate | Pillai's Trace | . | . | . |
|  | Wilks' Lambda | . | . | . |
|  | Hotelling's Trace | . | . | . |
|  | Roy's Largest Root | . | . | . |
| Log_nonfatalrate | Pillai's Trace | . | . | . |
|  | Wilks' Lambda | . | . | . |
|  | Hotelling's Trace | . | . | . |
|  | Roy's Largest Root | . | . | . |

| **Multivariate Tests** | | |
| --- | --- | --- |
| Dependent Variables | | Observed Power^b^ |
| Log_fatalrate, Log_nonfatalrate | Pillai's Trace | . |
|  | Wilks' Lambda | . |
|  | Hotelling's Trace | . |
|  | Roy's Largest Root | . |
| Log_fatalrate | Pillai's Trace | . |
|  | Wilks' Lambda | . |
|  | Hotelling's Trace | . |
|  | Roy's Largest Root | . |
| Log_nonfatalrate | Pillai's Trace | . |
|  | Wilks' Lambda | . |
|  | Hotelling's Trace | . |
|  | Roy's Largest Root | . |
| a. The pure error sub-matrix corresponding to the selected dependent variables is a zero matrix. | | |
| b. Computed using alpha = .05 | | |

| **Univariate Tests** | | | | | | |
| --- | --- | --- | --- | --- | --- | --- |
| Dependent Variable | Source | Sum of Squares | df | Mean Square | F | Sig. |
| Log_fatalrate | Lack of Fit | 3.980 | 10 | .398 | . | . |
|  | Pure Error | .000 | 0 | . |  |  |
| Log_nonfatalrate | Lack of Fit | 6.855 | 10 | .685 | . | . |
|  | Pure Error | .000 | 0 | . |  |  |

| **Univariate Tests** | | | | |
| --- | --- | --- | --- | --- |
| Dependent Variable | Source | Partial Eta Squared | Noncent. Parameter | Observed Power^a^ |
| Log_fatalrate | Lack of Fit | 1.000 | . | . |
|  | Pure Error |  |  |  |
| Log_nonfatalrate | Lack of Fit | 1.000 | . | . |
|  | Pure Error |  |  |  |
| a. Computed using alpha = .05 | | | | |

| **SSCP Matrix** | | | |
| --- | --- | --- | --- |
|  | | Log_fatalrate | Log_nonfatalrate |
| Lack of Fit | Log_fatalrate | 3.980 | 2.978 |
|  | Log_nonfatalrate | 2.978 | 6.855 |
| Pure Error | Log_fatalrate | .000 | .000 |
|  | Log_nonfatalrate | .000 | .000 |

**Estimated Marginal Means**

| **Grand Mean** | | | | |
| --- | --- | --- | --- | --- |
| Dependent Variable | Mean | Std. Error | 95% Confidence Interval | |
|  |  |  | Lower Bound | Upper Bound |
| Log_fatalrate | .486^a^ | .182 | .080 | .891 |
| Log_nonfatalrate | 2.072^a^ | .239 | 1.539 | 2.604 |
| a. Covariates appearing in the model are evaluated at the following values: Inspector_ratio = .495000. | | | | |

**Observed * Predicted * Std. Residual Plots**

Thus, following analysis have been completed with IBM SPSS 30.0 for ILO’s groups 3 and 4, as hereunder-

- Descriptive statistics with original data associated with the research variables,
- General linear multivariate regression,
- General linear multivariate regression with bootstrap-2000 with Confidence Intervals of 95%,
- Descriptive statistics with log-transformation of dependent variables,
- Log-general linear multivariate regression with bootstrap-2000 with Confidence Intervals of 95% and
- Log-general linear multivariate regression analysis.
