# Supplementary material for: OSH inspector ratio to strengthen decent workplace safety and health: a cross-regional trend analysis research
Source: Front Public Health. 2026 Apr 8;14:1806383. doi: 10.3389/fpubh.2026.1806383 (PMC13099828; doi:10.3389/fpubh.2026.1806383)
Supplement: Supplementary file 1 [file Data_Sheet_1.zip › Supplementary document Groups 3 and 4 - Descriptive and regression analysis.pdf]

Supplementary Material

Groups 3 and 4 – Descriptive and multivariate regression analysis

IBM SPSS 30.0 software was used to complete descriptive statistics and multivariate regression analysis for income groups 3 and 4, which originally had International Labour Organization's (ILO) 12 nations or regions. There are six major sections in this supplementary material as listed hereunder:

1. Descriptive statistics with original data associated with the research variables (PP. 2-10),
2. General linear multivariate regression (PP. 11-17),
3. General linear multivariate regression with bootstrap-2000 with Confidence Intervals of 95% (PP. 18-24),
4. Descriptive statistics with log-transformation of dependent variables (PP. 25-33),
5. Log-general linear multivariate regression with bootstrap-2000 with Confidence Intervals of 95% (PP. 34-40), and
6. Log-general linear multivariate regression analysis (PP. 41-47).

Note:

- Inspector ratio or inspector_ratio, or inspector_rate = Independent Variable (IV),
- Falta injury rate or fatal_rate = Dependent Variable (DV1), and
- Nonfatal injury rate or nonfatal_rate = Dependent Variable (DV2).

Descriptive statistics with original data associated with the research variables (PP. 2-10)

Explore

Notes

Output Created		24-FEB-2026 09:34:05
Comments		
Input	Data	E:\Group 3 and 4 - Feb 24\Groups 3 and 4 - Descriptive and regression analysis.sav
	Active Dataset	DataSet0
	Filter	<none>
	Weight	<none>
	Split File	<none>
	N of Rows in Working Data File	12
Missing Value Handling	Definition of Missing	User-defined missing values for dependent variables are treated as missing.
	Cases Used	Statistics are based on cases with no missing values for any dependent variable or factor used.
Syntax		EXAMINE VARIABLES=Inspector_ratio Fatal_rate Nonfatal_rate /PLOT BOXPLOT HISTOGRAM NPPLOT /COMPARE GROUPS /STATISTICS DESCRIPTIVES EXTREME /CINTERVAL 95 /MISSING LISTWISE /NOTOTAL.
Resources	Processor Time	00:00:05.55
	Elapsed Time	00:00:01.88

Case Processing Summary

	Cases					
	Valid		Missing		Total	
	N	Percent	N	Percent	N	Percent
Inspector_ratio	12	100.0%	0	0.0%	12	100.0%
Fatal_rate	12	100.0%	0	0.0%	12	100.0%
Nonfatal_rate	12	100.0%	0	0.0%	12	100.0%

Descriptives

			Statistic	Std. Error
Inspector_ratio	Mean		.495000	.0805521
	95% Confidence Interval for Mean	Lower Bound	.317706	
		Upper Bound	.672294	
	5% Trimmed Mean		.495000	
	Median		.515000	
	Variance		.078	
	Std. Deviation		.2790406	
	Minimum		.0700	
	Maximum		.9200	
	Range		.8500	
	Interquartile Range		.4925	
	Skewness		-.037	.637
	Kurtosis		-1.132	1.232
Fatal_rate	Mean		5.451333	1.4313466
	95% Confidence Interval for Mean	Lower Bound	2.300961	
		Upper Bound	8.601706	
	5% Trimmed Mean		5.143704	
	Median		4.000000	
	Variance		24.585	
	Std. Deviation		4.9583299	
	Minimum		.1400	
	Maximum		16.3000	
	Range		16.1600	
	Interquartile Range		5.7635	
	Skewness		1.210	.637
	Kurtosis		.986	1.232
Nonfatal_rate	Mean		467.108333	226.0748510
	95% Confidence Interval for Mean	Lower Bound	-30.479059	
		Upper Bound	964.695725	
	5% Trimmed Mean		389.978704	
	Median		120.000000	
	Variance		613318.059	
	Std. Deviation		783.1462565	
	Minimum		10.0000	
	Maximum		2312.5500	
	Range		2302.5500	
	Interquartile Range		420.4300	
	Skewness		1.966	.637
	Kurtosis		2.661	1.232

Extreme Values

			Case Number	Value
Inspector_ratio	Highest	1	5	.9200
		2	3	.8500
		3	8	.7500
		4	2	.6900
		5	7	.5800
	Lowest	1	4	.0700

		2	6	.1300
		3	9	.2200
		4	11	.3100
		5	12	.3900
Fatal_rate	Highest	1	12	16.3000
		2	2	13.1500
		3	8	7.6000
		4	3	6.4000
		5	10	6.0000
	Lowest	1	1	.1400
		2	7	.4800
		3	5	1.0000
		4	4	3.1460
		5	6	3.2000
Nonfatal_rate	Highest	1	2	2312.5500
		2	11	1894.7400
		3	12	484.0000
		4	10	322.0000
		5	6	227.0000
	Lowest	1	9	10.0000
		2	4	11.8300
		3	7	21.2000
		4	1	28.6800
		5	3	53.3000

Tests of Normality

	Kolmogorov-Smirnov[a]			Shapiro-Wilk		
	Statistic	df	Sig.	Statistic	df	Sig.
Inspector_ratio	.092	12	.200*	.966	12	.868
Fatal_rate	.174	12	.200*	.878	12	.082
Nonfatal_rate	.325	12	.001	.629	12	<.001

*. This is a lower bound of the true significance.

a. Lilliefors Significance Correction

Inspector_ratio

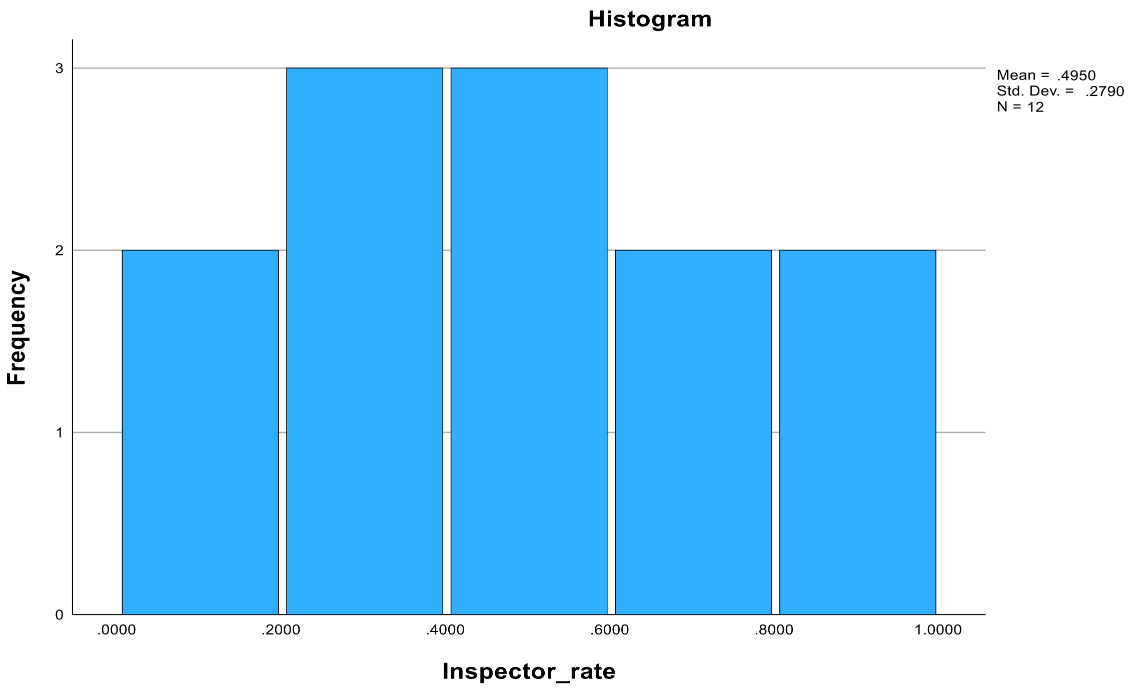

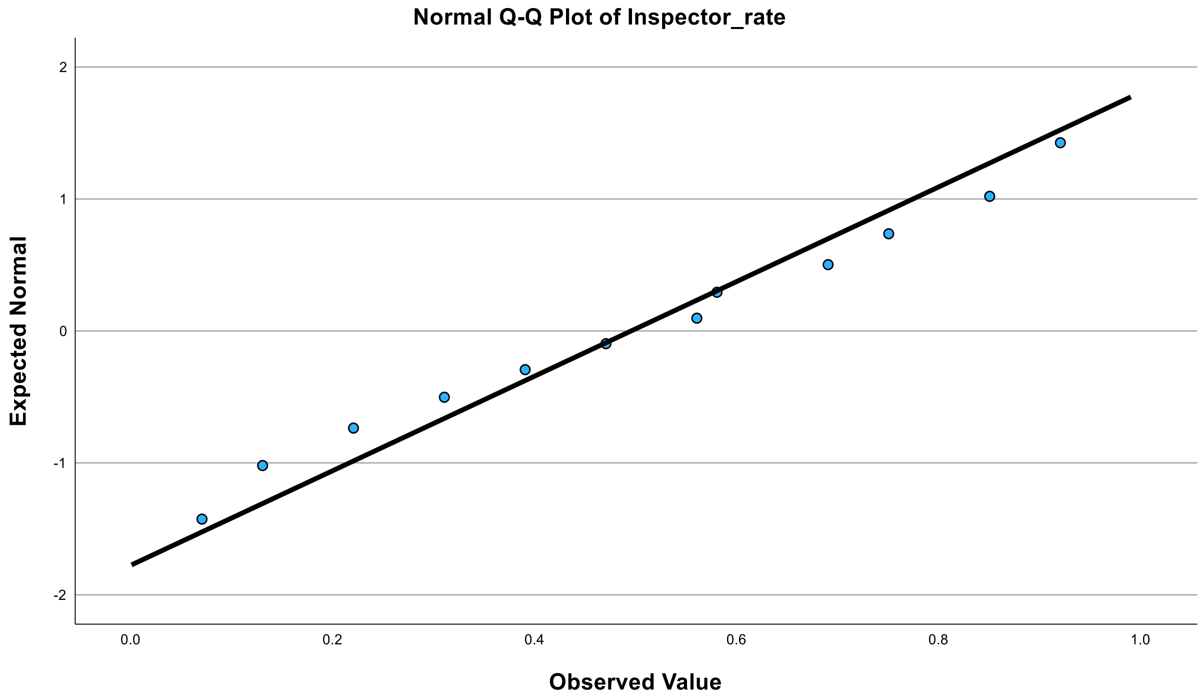

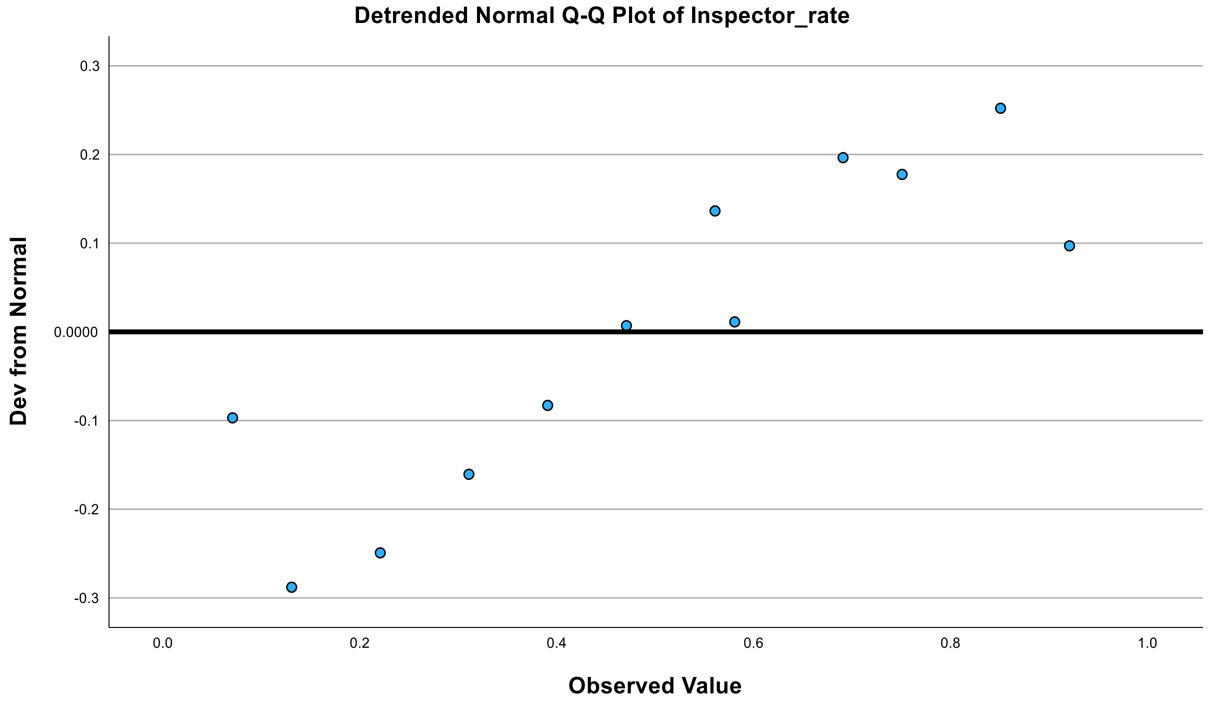
Detrended Normal Q-Q Plot of Inspector_rate
Dev from Normal
0.3
0.2
0.1
0.0000
-0.1
-0.2
-0.3
0.0
0.2
0.4
0.6
0.8
1.0
Observed Value

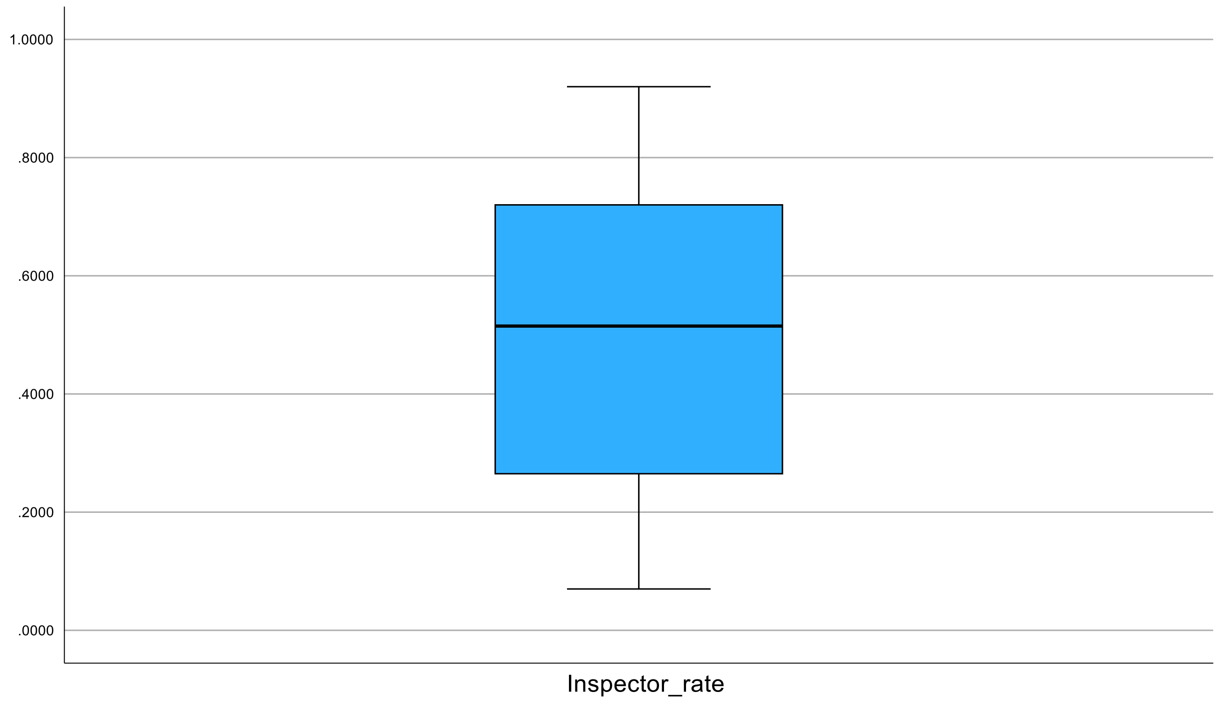
1.0000
.8000
.6000
.4000
.2000
.0000
Inspector_rate

Fatal_rate

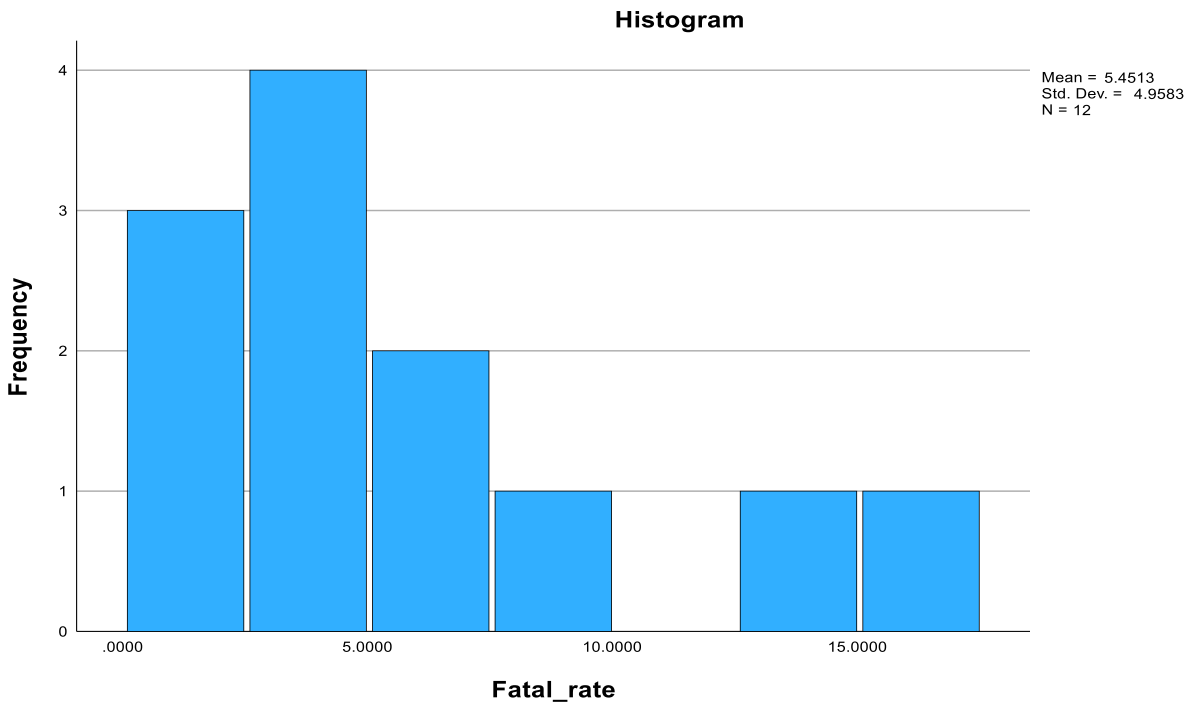
Histogram
Mean = 5.4513
Std. Dev. = 4.9583
N = 12
Frequency
0
1
2
3
4
.0000
5.0000
10.0000
15.0000
Fatal_rate

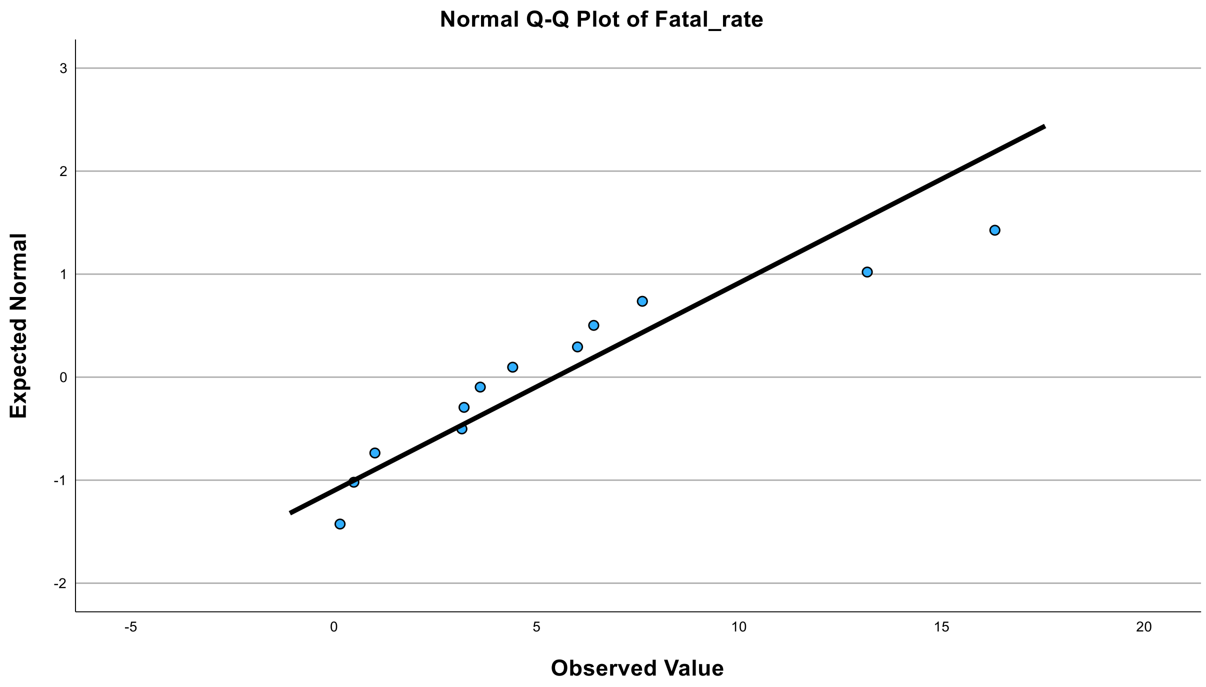
Normal Q-Q Plot of Fatal_rate
Expected Normal
3
2
1
0
-1
-2
-5
0
5
10
15
20
Observed Value

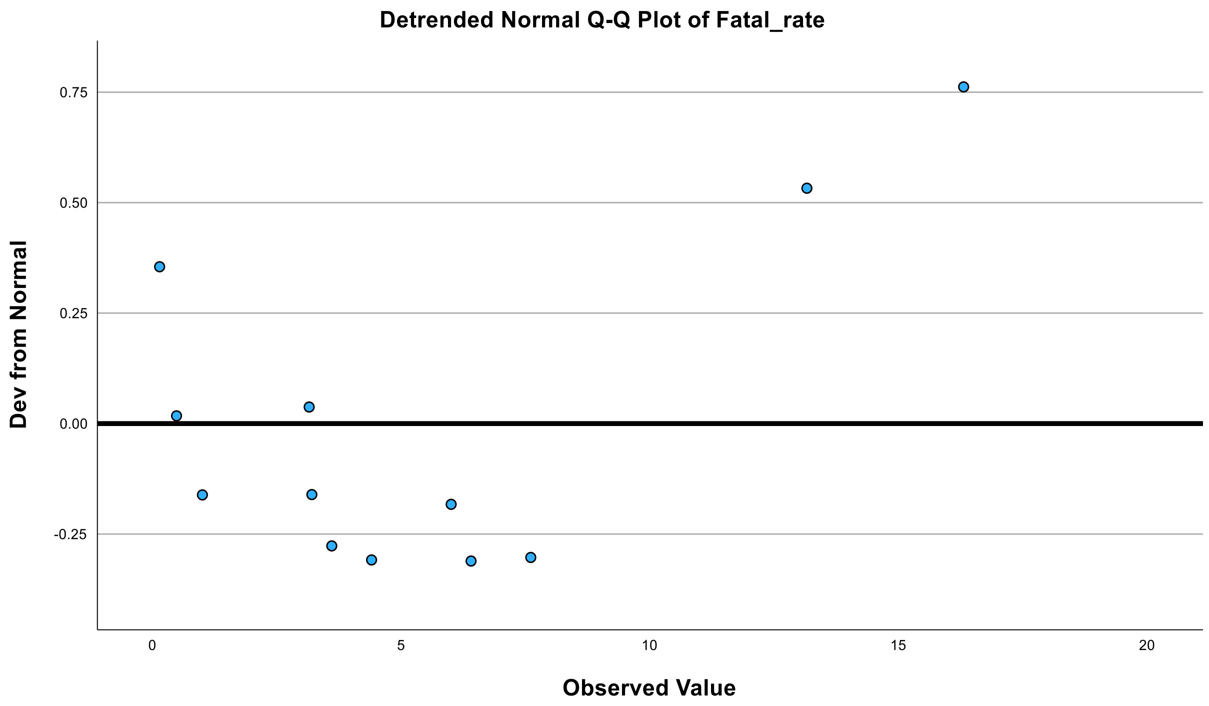
Detrended Normal Q-Q Plot of Fatal_rate
Dev from Normal
0.75
0.50
0.25
0.00
-0.25
0
5
10
15
20
Observed Value

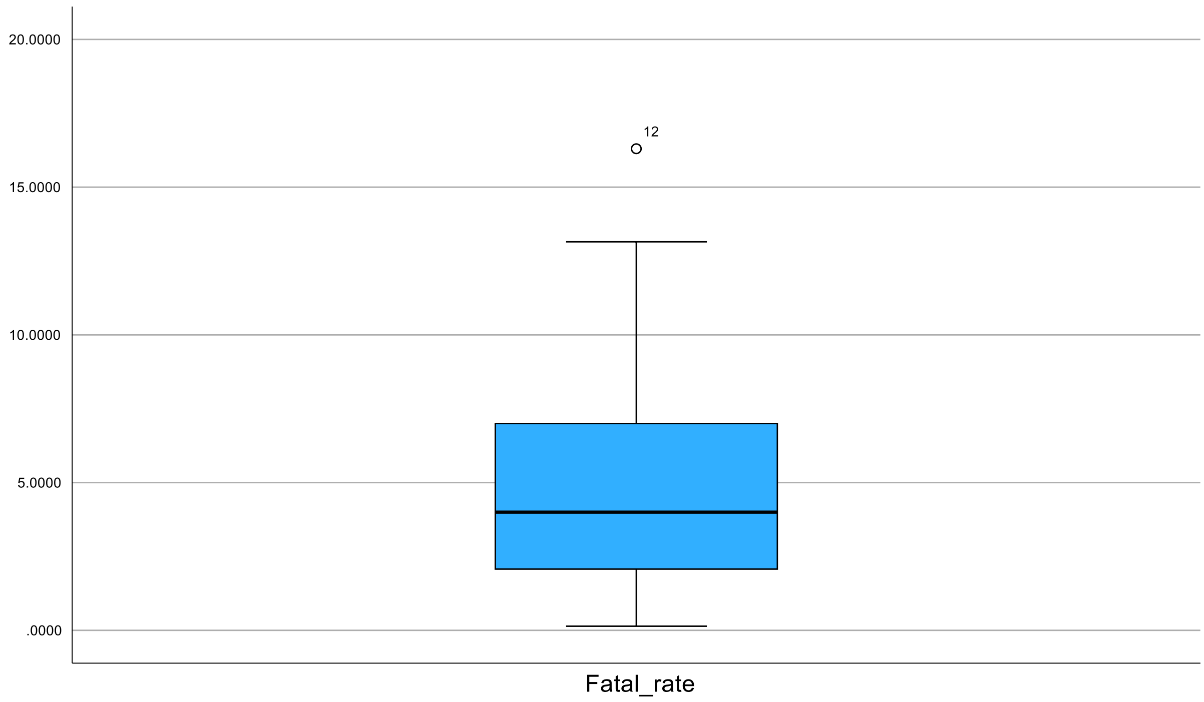
20.0000
15.0000
10.0000
5.0000
.0000
12
Fatal_rate

Nonfatal_rate

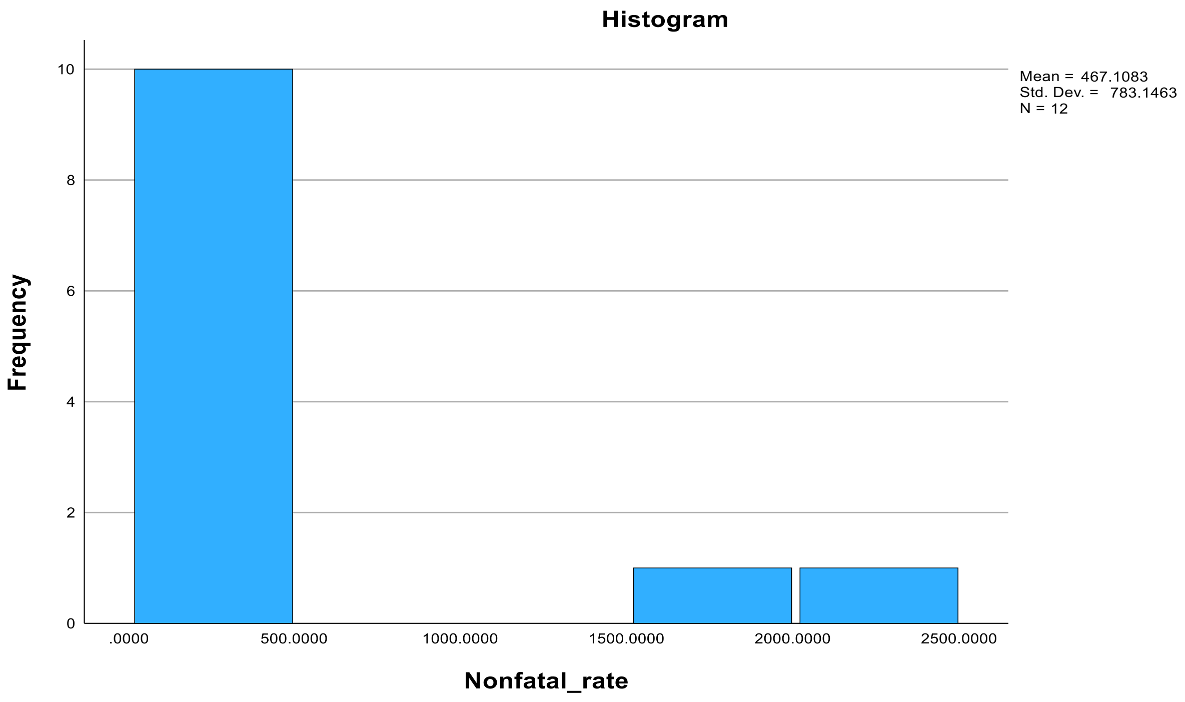

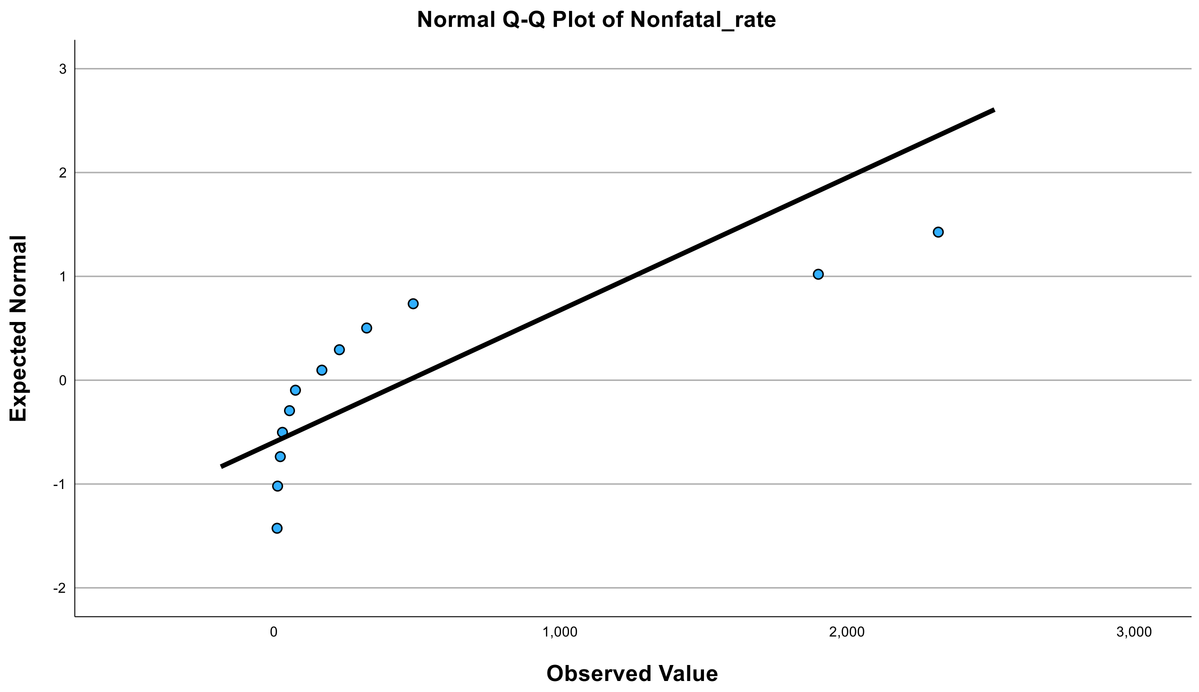

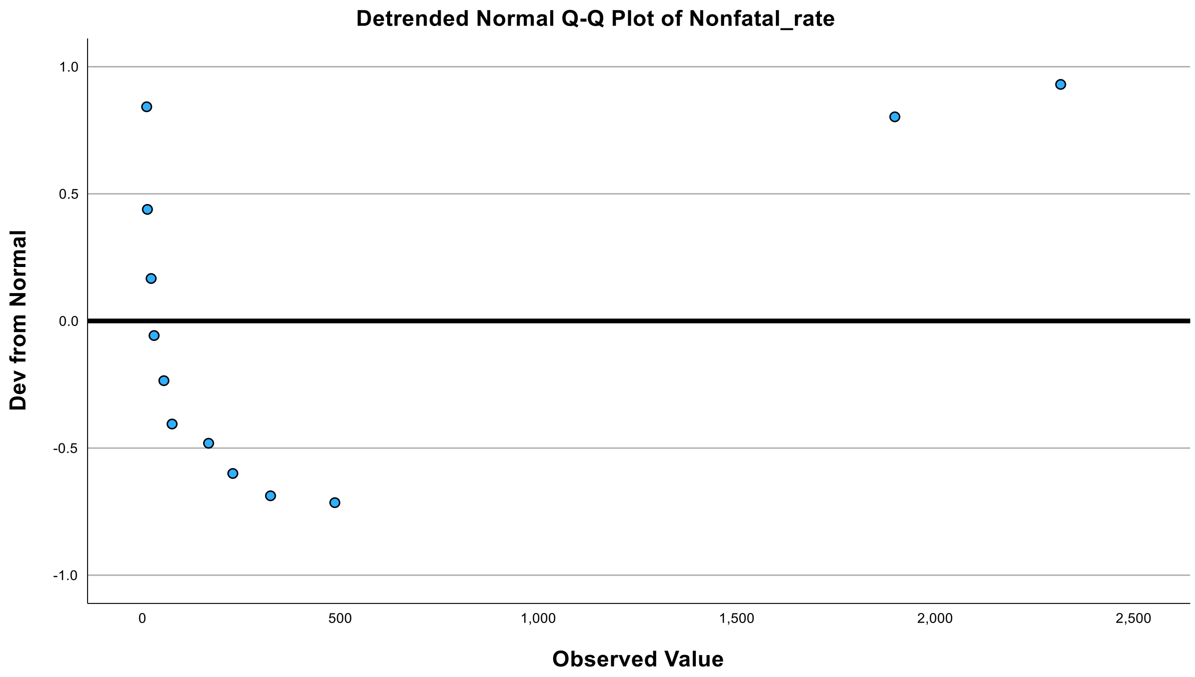
Detrended Normal Q-Q Plot of Nonfatal_rate
Dev from Normal
1.0
0.5
0.0
-0.5
-1.0
0
500
1,000
1,500
2,000
2,500
Observed Value

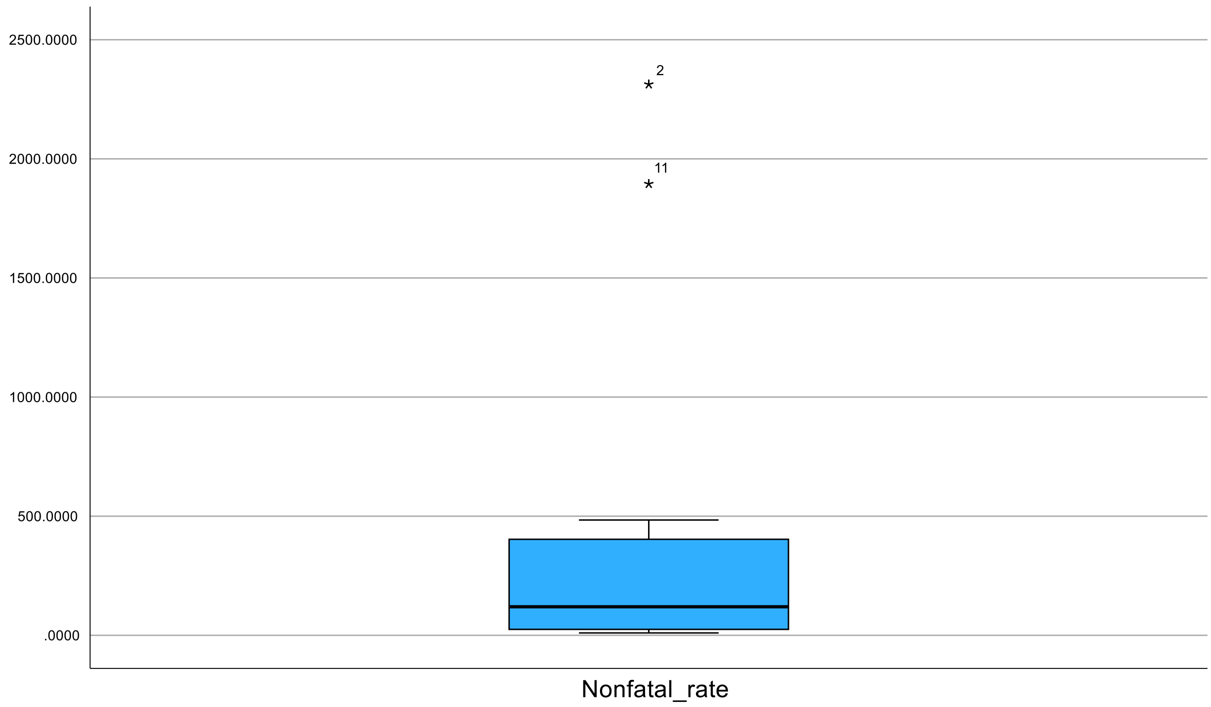
2500.0000
2000.0000
1500.0000
1000.0000
500.0000
.0000
2
11
Nonfatal_rate

General linear multivariate regression (PP. 11-17)

Notes

Output Created		24-FEB-2026 09:34:55
Comments		
Input	Data	E:\Group 3 and 4 - Feb 24\Groups 3 and 4 - Descriptive and regression analysis.sav
	Active Dataset	DataSet0
	Filter	<none>
	Weight	<none>
	Split File	<none>
	N of Rows in Working Data File	12
Missing Value Handling	Definition of Missing	User-defined missing values are treated as missing.
	Cases Used	Statistics are based on all cases with valid data for all variables in the model.
Syntax		GLM Fatal_rate Nonfatal_rate WITH Inspector_ratio<br>/METHOD=SSTYPE(3)<br>/INTERCEPT=INCLUDE<br>/SAVE=PRED SEPRED RESID ZRESID COOK LEVER<br>/EMMEANS=TABLES(OVERALL) WITH(Inspector_ratio=MEAN)<br>/PRINT=DESCRIPTIVE ETASQ OPOWER PARAMETER TEST(SSCP) RSSCP HOMOGENEITY LOF<br>/PLOT=SPREADLEVEL RESIDUALS<br>/CRITERIA=ALPHA(.05)<br>/DESIGN=Inspector_ratio.
Resources	Processor Time	00:00:00.53
	Elapsed Time	00:00:00.27
Variables Created or Modified	PRE_1	Predicted Value for Fatal_rate
	PRE_2	Predicted Value for Nonfatal_rate
	SEP_1	Standard Error of Predicted Value for Fatal_rate
	SEP_2	Standard Error of Predicted Value for Nonfatal_rate
	RES_1	Residual for Fatal_rate
	RES_2	Residual for Nonfatal_rate
	ZRE_1	Standardized Residual for Fatal_rate
	ZRE_2	Standardized Residual for Nonfatal_rate
	COO_1	Cook's Distance for Fatal_rate
	COO_2	Cook's Distance for Nonfatal_rate
	LEV_1	Uncentered Leverage Value for

		Fatal_rate
	LEV_2	Uncentered Leverage Value for Nonfatal_rate

Warnings

The HOMOGENEITY specification in the PRINT subcommand will be ignored because there are no between-subjects factors.
The SPREADLEVEL specification has been found in the PLOT subcommand, but the model includes no factors. The spread versus level plot will not be produced.

Descriptive Statistics

	Mean	Std. Deviation	N
Fatal_rate	5.451333	4.9583299	12
Nonfatal_rate	467.108333	783.1462565	12

Bartlett's Test of Sphericity[a]

Likelihood Ratio	.000
Approx. Chi-Square	81.090
df	2
Sig.	<.001

Tests the null hypothesis that the residual covariance matrix is proportional to an identity matrix.[a]

a. Design: Intercept + Inspector_ratio

Multivariate Tests[a]

Effect		Value	F	Hypothesis df	Error df	Sig.
Intercept	Pillai's Trace	.174	.947[b]	2.000	9.000	.423
	Wilks' Lambda	.826	.947[b]	2.000	9.000	.423
	Hotelling's Trace	.210	.947[b]	2.000	9.000	.423
	Roy's Largest Root	.210	.947[b]	2.000	9.000	.423
Inspector_ratio	Pillai's Trace	.012	.053[b]	2.000	9.000	.949
	Wilks' Lambda	.988	.053[b]	2.000	9.000	.949
	Hotelling's Trace	.012	.053[b]	2.000	9.000	.949
	Roy's Largest Root	.012	.053[b]	2.000	9.000	.949

Multivariate Tests[a]

Effect		Partial Eta Squared	Noncent. Parameter	Observed Power[c]
Intercept	Pillai's Trace	.174	1.894	.166
	Wilks' Lambda	.174	1.894	.166
	Hotelling's Trace	.174	1.894	.166
	Roy's Largest Root	.174	1.894	.166
Inspector_ratio	Pillai's Trace	.012	.106	.056
	Wilks' Lambda	.012	.106	.056
	Hotelling's Trace	.012	.106	.056
	Roy's Largest Root	.012	.106	.056

a. Design: Intercept + Inspector_ratio

b. Exact statistic

c. Computed using alpha = .05

Tests of Between-Subjects Effects

Source	Dependent Variable	Type III Sum of Squares	df	Mean Square	F	Sig.
Corrected Model	Fatal_rate	3.011[a]	1	3.011	.113	.744
	Nonfatal_rate	6338.992[b]	1	6338.992	.009	.925
Intercept	Fatal_rate	55.383	1	55.383	2.071	.181
	Nonfatal_rate	487859.801	1	487859.801	.724	.415
Inspector_ratio	Fatal_rate	3.011	1	3.011	.113	.744
	Nonfatal_rate	6338.992	1	6338.992	.009	.925
Error	Fatal_rate	267.424	10	26.742		
	Nonfatal_rate	6740159.659	10	674015.966		
Total	Fatal_rate	627.040	12			
	Nonfatal_rate	9364780.991	12			
Corrected Total	Fatal_rate	270.435	11			
	Nonfatal_rate	6746498.651	11			

Tests of Between-Subjects Effects

Source	Dependent Variable	Partial Eta Squared	Noncent. Parameter	Observed Power[c]
Corrected Model	Fatal_rate	.011	.113	.061
	Nonfatal_rate	.001	.009	.051
Intercept	Fatal_rate	.172	2.071	.256
	Nonfatal_rate	.067	.724	.120
Inspector_ratio	Fatal_rate	.011	.113	.061
	Nonfatal_rate	.001	.009	.051
Error	Fatal_rate			
	Nonfatal_rate			
Total	Fatal_rate			
	Nonfatal_rate			
Corrected Total	Fatal_rate			
	Nonfatal_rate			

a. R Squared = .011 (Adjusted R Squared = -.088)
b. R Squared = .001 (Adjusted R Squared = -.099)
c. Computed using alpha = .05

Parameter Estimates

Dependent Variable	Parameter	B	Std. Error	t	Sig.	95% Confidence Interval Lower Bound
Fatal_rate	Intercept	4.523	3.143	1.439	.181	-2.480
	Inspector_ratio	1.875	5.588	.336	.744	-10.575
Nonfatal_rate	Intercept	424.524	498.987	.851	.415	-687.290
	Inspector_ratio	86.029	887.098	.097	.925	-1890.547

Parameter Estimates

Dependent Variable	Parameter	95% Confidence Interval Upper Bound	Partial Eta Squared	Noncent. Parameter	Observed Power[a]
Fatal_rate	Intercept	11.526	.172	1.439	.256
	Inspector_ratio	14.325	.011	.336	.061
Nonfatal_rate	Intercept	1536.337	.067	.851	.120
	Inspector_ratio	2062.606	.001	.097	.051

a. Computed using alpha = .05

Between-Subjects SSCP Matrix

			Fatal_rate	Nonfatal_rate
Hypothesis	Intercept	Fatal_rate	55.383	5197.998
		Nonfatal_rate	5197.998	487859.801
	Inspector_ratio	Fatal_rate	3.011	138.163
		Nonfatal_rate	138.163	6338.992
Error		Fatal_rate	267.424	20364.095
		Nonfatal_rate	20364.095	6740159.659

Based on Type III Sum of Squares

Residual SSCP Matrix

		Fatal_rate	Nonfatal_rate
Sum-of-Squares and Cross-Products	Fatal_rate	267.424	20364.095
	Nonfatal_rate	20364.095	6740159.659
Covariance	Fatal_rate	26.742	2036.410
	Nonfatal_rate	2036.410	674015.966
Correlation	Fatal_rate	1.000	.480
	Nonfatal_rate	.480	1.000

Based on Type III Sum of Squares

Lack of Fit

Multivariate Tests

Dependent Variables		Value	F	Hypothesis df	Error df
Fatal_rate, Nonfatal_rate	Pillai's Trace	.[a]	.	.	.
	Wilks' Lambda	.[a]	.	.	.
	Hotelling's Trace	.[a]	.	.	.
	Roy's Largest Root	.[a]	.	.	.
Fatal_rate	Pillai's Trace	.[a]	.	.	.
	Wilks' Lambda	.[a]	.	.	.
	Hotelling's Trace	.[a]	.	.	.
	Roy's Largest Root	.[a]	.	.	.
Nonfatal_rate	Pillai's Trace	.[a]	.	.	.
	Wilks' Lambda	.[a]	.	.	.
	Hotelling's Trace	.[a]	.	.	.
	Roy's Largest Root	.[a]	.	.	.

Multivariate Tests

Dependent Variables		Sig.	Partial Eta Squared	Noncent. Parameter
Fatal_rate, Nonfatal_rate	Pillai's Trace	.	.	.
	Wilks' Lambda	.	.	.
	Hotelling's Trace	.	.	.
	Roy's Largest Root	.	.	.
Fatal_rate	Pillai's Trace	.	.	.
	Wilks' Lambda	.	.	.
	Hotelling's Trace	.	.	.
	Roy's Largest Root	.	.	.
Nonfatal_rate	Pillai's Trace	.	.	.
	Wilks' Lambda	.	.	.
	Hotelling's Trace	.	.	.
	Roy's Largest Root	.	.	.

Multivariate Tests

Dependent Variables		Observed Power[b]
Fatal_rate, Nonfatal_rate	Pillai's Trace	.
	Wilks' Lambda	.
	Hotelling's Trace	.
	Roy's Largest Root	.
Fatal_rate	Pillai's Trace	.
	Wilks' Lambda	.
	Hotelling's Trace	.
	Roy's Largest Root	.
Nonfatal_rate	Pillai's Trace	.
	Wilks' Lambda	.
	Hotelling's Trace	.
	Roy's Largest Root	.

a. The pure error sub-matrix corresponding to the selected dependent variables is a zero matrix.
b. Computed using alpha = .05

Univariate Tests

Dependent Variable	Source	Sum of Squares	df	Mean Square	F	Sig.
Fatal_rate	Lack of Fit	267.424	10	26.742	.	.
	Pure Error	.000	0	.		
Nonfatal_rate	Lack of Fit	6740159.659	10	674015.966	.	.
	Pure Error	.000	0	.		

Univariate Tests

Dependent Variable	Source	Partial Eta Squared	Noncent. Parameter	Observed Power[a]
Fatal_rate	Lack of Fit	1.000	.	.
	Pure Error			
Nonfatal_rate	Lack of Fit	1.000	.	.
	Pure Error			

a. Computed using alpha = .05

SSCP Matrix

		Fatal_rate	Nonfatal_rate
Lack of Fit	Fatal_rate	267.424	20364.095
	Nonfatal_rate	20364.095	6740159.659
Pure Error	Fatal_rate	.000	.000
	Nonfatal_rate	.000	.000

Estimated Marginal Means

Grand Mean

Dependent Variable	Mean	Std. Error	95% Confidence Interval Lower Bound	95% Confidence Interval Upper Bound
Fatal_rate	5.451[a]	1.493	2.125	8.778
Nonfatal_rate	467.108[a]	236.998	-60.956	995.173

a. Covariates appearing in the model are evaluated at the following values: Inspector_ratio = .495000.

Observed * Predicted * Std. Residual Plots

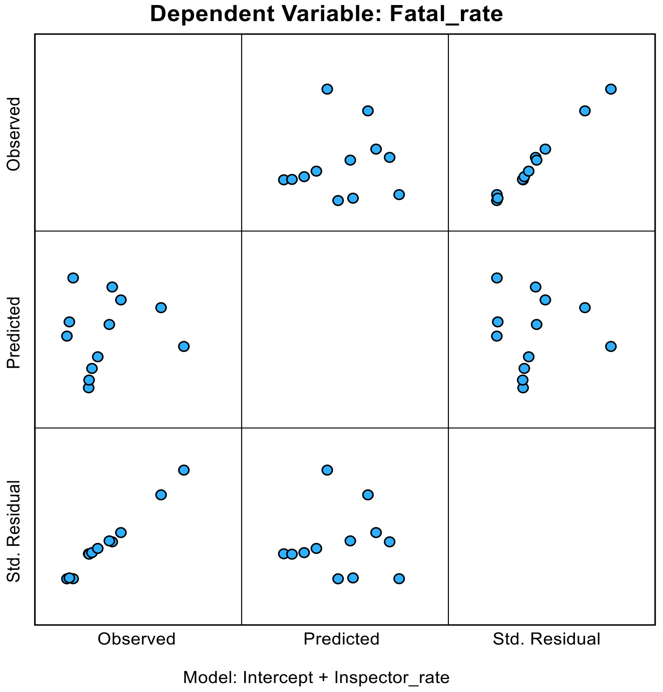

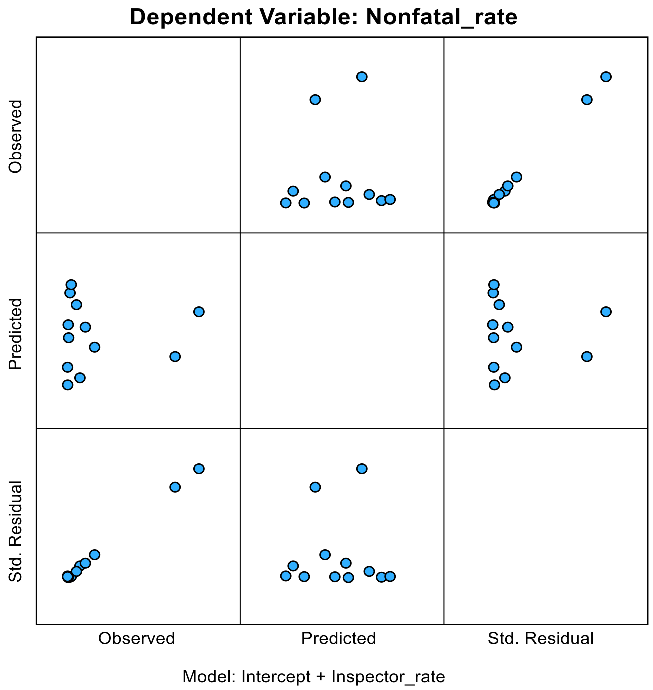
Dependent Variable: Nonfatal_rate
Observed
Predicted
Std. Residual
Observed
Predicted
Std. Residual
Model: Intercept + Inspector_rate

General linear multivariate regression with bootstrap-2000 with Confidence Intervals of 95% (PP. 18-24)

Bootstrap

Notes

Output Created		24-FEB-2026 09:35:58
Comments		
Input	Data	E:\Group 3 and 4 - Feb 24\Groups 3 and 4 - Descriptive and regression analysis.sav
	Active Dataset	DataSet0
	Filter	<none>
	Weight	<none>
	Split File	<none>
	N of Rows in Working Data File	12
Syntax		BOOTSTRAP<br>/SAMPLING METHOD=SIMPLE<br>/VARIABLES TARGET=Fatal_rate Nonfatal_rate INPUT= Inspector_ratio<br>/CRITERIA CILEVEL=95 CITYPE=PERCENTILE NSAMPLES=2000<br>/MISSING USERMISSING=EXCLUDE.
Resources	Processor Time	00:00:00.02
	Elapsed Time	00:00:00.02

Bootstrap Specifications

Sampling Method	Simple
Number of Samples	2000
Confidence Interval Level	95.0%
Confidence Interval Type	Percentile

General Linear Model

Notes

Output Created		24-FEB-2026 09:35:58
Comments		
Input	Data	E:\Group 3 and 4 - Feb 24\Groups 3 and 4 - Descriptive and regression analysis.sav
	Active Dataset	DataSet0
	Filter	<none>
	Weight	<none>
	Split File	<none>
	N of Rows in Working Data File	24012
Missing Value Handling	Definition of Missing	User-defined missing values are treated as missing.
	Cases Used	Statistics are based on all cases with valid

		data for all variables in the model.
Syntax		GLM Fatal_rate Nonfatal_rate WITH Inspector_ratio<br>/METHOD=SSTYPE(3)<br>/INTERCEPT=INCLUDE<br>/SAVE=PRED SEPRED RESID ZRESID COOK LEVER<br>/EMMEANS=TABLES(OVERALL) WITH(Inspector_ratio=MEAN)<br>/PRINT=DESCRIPTIVE ETASQ OPOWER PARAMETER TEST(SSCP) RSSCP HOMOGENEITY LOF<br>/PLOT=SPREADLEVEL RESIDUALS<br>/CRITERIA=ALPHA(.05)<br>/DESIGN=Inspector_ratio.
Resources	Processor Time	00:00:25.98
	Elapsed Time	00:00:25.65

Warnings

The HOMOGENEITY specification in the PRINT subcommand will be ignored because there are no between-subjects factors.
The SPREADLEVEL specification has been found in the PLOT subcommand, but the model includes no factors. The spread versus level plot will not be produced.
This command is trying to save new variables while bootstrapping is in effect. The new variables will be lost.<br>Execution of this command stops.

Descriptive Statistics

	Mean	Std. Deviation	N
Fatal_rate	5.451333	4.9583299	12
Nonfatal_rate	467.108333	783.1462565	12

Bartlett's Test of Sphericity[a]

Likelihood Ratio	.000
Approx. Chi-Square	81.090
df	2
Sig.	<.001

Tests the null hypothesis that the residual covariance matrix is proportional to an identity matrix.[a]
a. Design: Intercept + Inspector_ratio

Multivariate Tests[a]

Effect		Value	F	Hypothesis df	Error df	Sig.
Intercept	Pillai's Trace	.174	.947[b]	2.000	9.000	.423
	Wilks' Lambda	.826	.947[b]	2.000	9.000	.423
	Hotelling's Trace	.210	.947[b]	2.000	9.000	.423
	Roy's Largest Root	.210	.947[b]	2.000	9.000	.423

Inspector_ratio	Pillai's Trace	.012	.053[b]	2.000	9.000	.949
	Wilks' Lambda	.988	.053[b]	2.000	9.000	.949
	Hotelling's Trace	.012	.053[b]	2.000	9.000	.949
	Roy's Largest Root	.012	.053[b]	2.000	9.000	.949

Multivariate Tests[a]

Effect		Partial Eta Squared	Noncent. Parameter	Observed Power[c]
Intercept	Pillai's Trace	.174	1.894	.166
	Wilks' Lambda	.174	1.894	.166
	Hotelling's Trace	.174	1.894	.166
	Roy's Largest Root	.174	1.894	.166
Inspector_ratio	Pillai's Trace	.012	.106	.056
	Wilks' Lambda	.012	.106	.056
	Hotelling's Trace	.012	.106	.056
	Roy's Largest Root	.012	.106	.056

a. Design: Intercept + Inspector_ratio
b. Exact statistic
c. Computed using alpha = .05

Tests of Between-Subjects Effects

Source	Dependent Variable	Type III Sum of Squares	df	Mean Square	F	Sig.
Corrected Model	Fatal_rate	3.011[a]	1	3.011	.113	.744
	Nonfatal_rate	6338.992[b]	1	6338.992	.009	.925
Intercept	Fatal_rate	55.383	1	55.383	2.071	.181
	Nonfatal_rate	487859.801	1	487859.801	.724	.415
Inspector_ratio	Fatal_rate	3.011	1	3.011	.113	.744
	Nonfatal_rate	6338.992	1	6338.992	.009	.925
Error	Fatal_rate	267.424	10	26.742		
	Nonfatal_rate	6740159.659	10	674015.966		
Total	Fatal_rate	627.040	12			
	Nonfatal_rate	9364780.991	12			
Corrected Total	Fatal_rate	270.435	11			
	Nonfatal_rate	6746498.651	11			

Tests of Between-Subjects Effects

Source	Dependent Variable	Partial Eta Squared	Noncent. Parameter	Observed Power[c]
Corrected Model	Fatal_rate	.011	.113	.061
	Nonfatal_rate	.001	.009	.051
Intercept	Fatal_rate	.172	2.071	.256
	Nonfatal_rate	.067	.724	.120
Inspector_ratio	Fatal_rate	.011	.113	.061
	Nonfatal_rate	.001	.009	.051
Error	Fatal_rate			
	Nonfatal_rate			
Total	Fatal_rate			
	Nonfatal_rate			
Corrected Total	Fatal_rate			

Nonfatal_rate			

a. R Squared = .011 (Adjusted R Squared = -.088)
b. R Squared = .001 (Adjusted R Squared = -.099)
c. Computed using alpha = .05

Parameter Estimates

Dependent Variable	Parameter	B	Std. Error	t	Sig.	95% Confidence Interval Lower Bound
Fatal_rate	Intercept	4.523	3.143	1.439	.181	-2.480
	Inspector_ratio	1.875	5.588	.336	.744	-10.575
Nonfatal_rate	Intercept	424.524	498.987	.851	.415	-687.290
	Inspector_ratio	86.029	887.098	.097	.925	-1890.547

Parameter Estimates

Dependent Variable	Parameter	95% Confidence Interval Upper Bound	Partial Eta Squared	Noncent. Parameter	Observed Power[a]
Fatal_rate	Intercept	11.526	.172	1.439	.256
	Inspector_ratio	14.325	.011	.336	.061
Nonfatal_rate	Intercept	1536.337	.067	.851	.120
	Inspector_ratio	2062.606	.001	.097	.051

a. Computed using alpha = .05

Bootstrap for Parameter Estimates

			Bootstrap[a]			
Dependent Variable	Parameter	B	Bias	Std. Error	Sig. (2-tailed)	95% Confidence Interval Lower
Fatal_rate	Intercept	4.523	-.014	2.519	.083	.702
	Inspector_ratio	1.875	.208	4.513	.673	-7.419
Nonfatal_rate	Intercept	424.524	17.262	430.825	.342	-213.115
	Inspector_ratio	86.029	12.842	833.376	.918	-1567.512

Bootstrap for Parameter Estimates

Dependent Variable	Parameter	Bootstrap 95% Confidence Interval Upper
Fatal_rate	Intercept	10.467
	Inspector_ratio	11.215
Nonfatal_rate	Intercept	1527.978
	Inspector_ratio	2020.185

a. Unless otherwise noted, bootstrap results are based on 2000 bootstrap samples

Between-Subjects SSCP Matrix

			Fatal_rate	Nonfatal_rate
Hypothesis	Intercept	Fatal_rate	55.383	5197.998
		Nonfatal_rate	5197.998	487859.801
	Inspector_ratio	Fatal_rate	3.011	138.163
		Nonfatal_rate	138.163	6338.992
Error		Fatal_rate	267.424	20364.095
		Nonfatal_rate	20364.095	6740159.659

Based on Type III Sum of Squares

Residual SSCP Matrix

		Fatal_rate	Nonfatal_rate
Sum-of-Squares and Cross-Products	Fatal_rate	267.424	20364.095
	Nonfatal_rate	20364.095	6740159.659
Covariance	Fatal_rate	26.742	2036.410
	Nonfatal_rate	2036.410	674015.966
Correlation	Fatal_rate	1.000	.480
	Nonfatal_rate	.480	1.000

Based on Type III Sum of Squares

Lack of Fit

Multivariate Tests

Dependent Variables		Value	F	Hypothesis df	Error df
Fatal_rate, Nonfatal_rate	Pillai's Trace	.[a]	.	.	.
	Wilks' Lambda	.[a]	.	.	.
	Hotelling's Trace	.[a]	.	.	.
	Roy's Largest Root	.[a]	.	.	.
Fatal_rate	Pillai's Trace	.[a]	.	.	.
	Wilks' Lambda	.[a]	.	.	.
	Hotelling's Trace	.[a]	.	.	.
	Roy's Largest Root	.[a]	.	.	.
Nonfatal_rate	Pillai's Trace	.[a]	.	.	.
	Wilks' Lambda	.[a]	.	.	.
	Hotelling's Trace	.[a]	.	.	.
	Roy's Largest Root	.[a]	.	.	.

Multivariate Tests

Dependent Variables		Sig.	Partial Eta Squared	Noncent. Parameter
Fatal_rate, Nonfatal_rate	Pillai's Trace	.	.	.
	Wilks' Lambda	.	.	.
	Hotelling's Trace	.	.	.
	Roy's Largest Root	.	.	.
Fatal_rate	Pillai's Trace	.	.	.
	Wilks' Lambda	.	.	.
	Hotelling's Trace	.	.	.

	Roy's Largest Root	.	.	.
Nonfatal_rate	Pillai's Trace	.	.	.
	Wilks' Lambda	.	.	.
	Hotelling's Trace	.	.	.
	Roy's Largest Root	.	.	.

Multivariate Tests

Dependent Variables		Observed Power[b]
Fatal_rate, Nonfatal_rate	Pillai's Trace	.
	Wilks' Lambda	.
	Hotelling's Trace	.
	Roy's Largest Root	.
Fatal_rate	Pillai's Trace	.
	Wilks' Lambda	.
	Hotelling's Trace	.
	Roy's Largest Root	.
Nonfatal_rate	Pillai's Trace	.
	Wilks' Lambda	.
	Hotelling's Trace	.
	Roy's Largest Root	.

a. The pure error sub-matrix corresponding to the selected dependent variables is a zero matrix.
b. Computed using alpha = .05

Univariate Tests

Dependent Variable	Source	Sum of Squares	df	Mean Square	F	Sig.
Fatal_rate	Lack of Fit	267.424	10	26.742	.	.
	Pure Error	.000	0	.		
Nonfatal_rate	Lack of Fit	6740159.659	10	674015.966	.	.
	Pure Error	.000	0	.		

Univariate Tests

Dependent Variable	Source	Partial Eta Squared	Noncent. Parameter	Observed Power[a]
Fatal_rate	Lack of Fit	1.000	.	.
	Pure Error			
Nonfatal_rate	Lack of Fit	1.000	.	.
	Pure Error			

a. Computed using alpha = .05

SSCP Matrix

		Fatal_rate	Nonfatal_rate
Lack of Fit	Fatal_rate	267.424	20364.095
	Nonfatal_rate	20364.095	6740159.659
Pure Error	Fatal_rate	.000	.000
	Nonfatal_rate	.000	.000

Estimated Marginal Means

Grand Mean

Dependent Variable	Mean	Std. Error	95% Confidence Interval Lower Bound	95% Confidence Interval Upper Bound
Fatal_rate	5.451[a]	1.493	2.125	8.778
Nonfatal_rate	467.108[a]	236.998	-60.956	995.173

a. Covariates appearing in the model are evaluated at the following values: Inspector_ratio = .495000.

Continued.

Descriptive statistics with log-transformation of dependent variables (PP. 25-33)

Explore

Notes

Output Created		24-FEB-2026 09:38:35
Comments		
Input	Data	E:\Group 3 and 4 - Feb 24\Groups 3 and 4 - Descriptive and regression analysis.sav
	Active Dataset	DataSet0
	Filter	<none>
	Weight	<none>
	Split File	<none>
	N of Rows in Working Data File	12
Missing Value Handling	Definition of Missing	User-defined missing values for dependent variables are treated as missing.
	Cases Used	Statistics are based on cases with no missing values for any dependent variable or factor used.
Syntax		EXAMINE VARIABLES=Inspector_ratio Log_fatalrate Log_nonfatalrate<br>/PLOT BOXPLOT HISTOGRAM NPPLOT<br>/COMPARE GROUPS<br>/STATISTICS DESCRIPTIVES EXTREME<br>/CINTERVAL 95<br>/MISSING LISTWISE<br>/NOTOTAL.
Resources	Processor Time	00:00:01.64
	Elapsed Time	00:00:01.26

Case Processing Summary

	Cases					
	Valid		Missing		Total	
	N	Percent	N	Percent	N	Percent
Inspector_ratio	12	100.0%	0	0.0%	12	100.0%
Log_fatalrate	12	100.0%	0	0.0%	12	100.0%
Log_nonfatalrate	12	100.0%	0	0.0%	12	100.0%

Descriptives

			Statistic	Std. Error
Inspector_ratio	Mean		.495000	.0805521
	95% Confidence Interval for Mean	Lower Bound	.317706	
		Upper Bound	.672294	
	5% Trimmed Mean		.495000	
	Median		.515000	
	Variance		.078	
	Std. Deviation		.2790406	
	Minimum		.0700	
	Maximum		.9200	
	Range		.8500	
	Interquartile Range		.4925	
	Skewness		-.037	.637
	Kurtosis		-1.132	1.232
Log_fatalrate	Mean		.4855	.17366
	95% Confidence Interval for Mean	Lower Bound	.1033	
		Upper Bound	.8678	
	5% Trimmed Mean		.5196	
	Median		.5999	
	Variance		.362	
	Std. Deviation		.60159	
	Minimum		-.85	
	Maximum		1.21	
	Range		2.07	
	Interquartile Range		.74	
	Skewness		-1.140	.637
	Kurtosis		.990	1.232
Log_nonfatalrate	Mean		2.0719	.23081
	95% Confidence Interval for Mean	Lower Bound	1.5639	
		Upper Bound	2.5800	
	5% Trimmed Mean		2.0597	
	Median		2.0447	
	Variance		.639	
	Std. Deviation		.79955	
	Minimum		1.00	
	Maximum		3.36	
	Range		2.36	
	Interquartile Range		1.28	
	Skewness		.283	.637
	Kurtosis		-1.000	1.232

Extreme Values

			Case Number	Value
Inspector_ratio	Highest	1	5	.9200
		2	3	.8500
		3	8	.7500
		4	2	.6900
		5	7	.5800
	Lowest	1	4	.0700
		2	6	.1300
		3	9	.2200
		4	11	.3100
		5	12	.3900
Log_fatalrate	Highest	1	12	1.21
		2	2	1.12
		3	8	.88
		4	3	.81
		5	10	.78
	Lowest	1	1	-.85
		2	7	-.32
		3	5	.00
		4	4	.50
		5	6	.51
Log_nonfatalrate	Highest	1	2	3.36
		2	11	3.28
		3	12	2.68
		4	10	2.51
		5	6	2.36
	Lowest	1	9	1.00
		2	4	1.07
		3	7	1.33
		4	1	1.46
		5	3	1.73

Tests of Normality

	Kolmogorov-Smirnov[a]			Shapiro-Wilk		
	Statistic	df	Sig.	Statistic	df	Sig.
Inspector_ratio	.092	12	.200*	.966	12	.868
Log_fatalrate	.258	12	.026	.900	12	.158
Log_nonfatalrate	.112	12	.200*	.948	12	.605

*. This is a lower bound of the true significance.

a. Lilliefors Significance Correction

Inspector_ratio

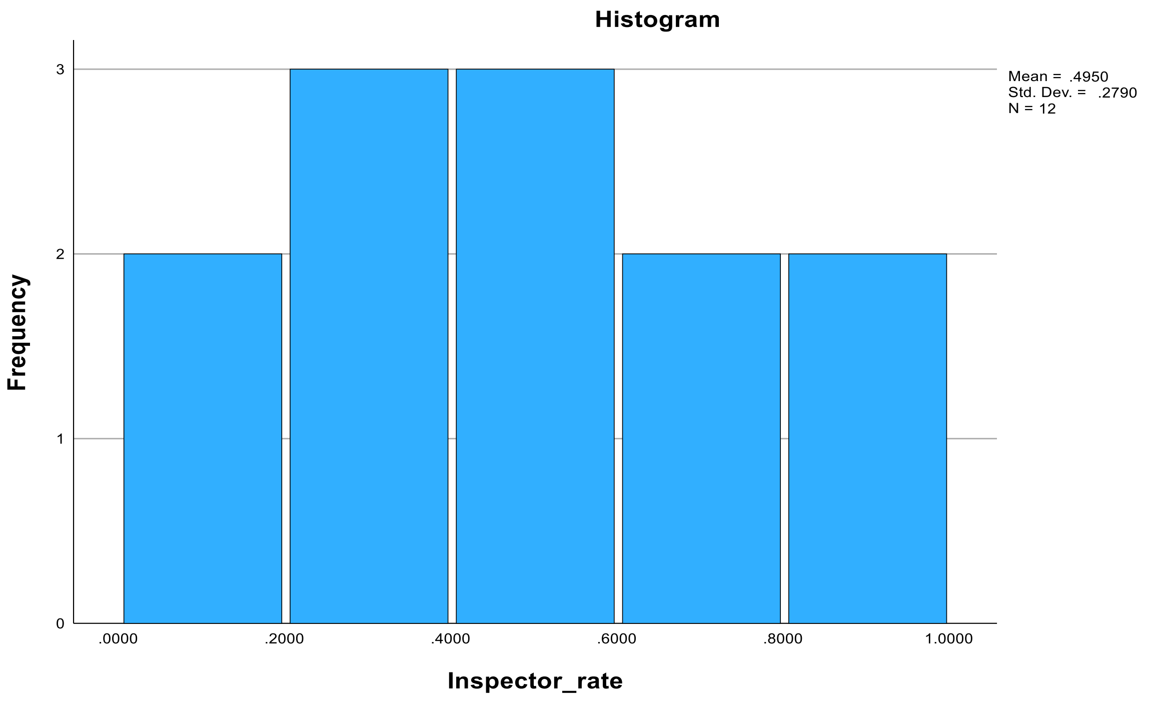

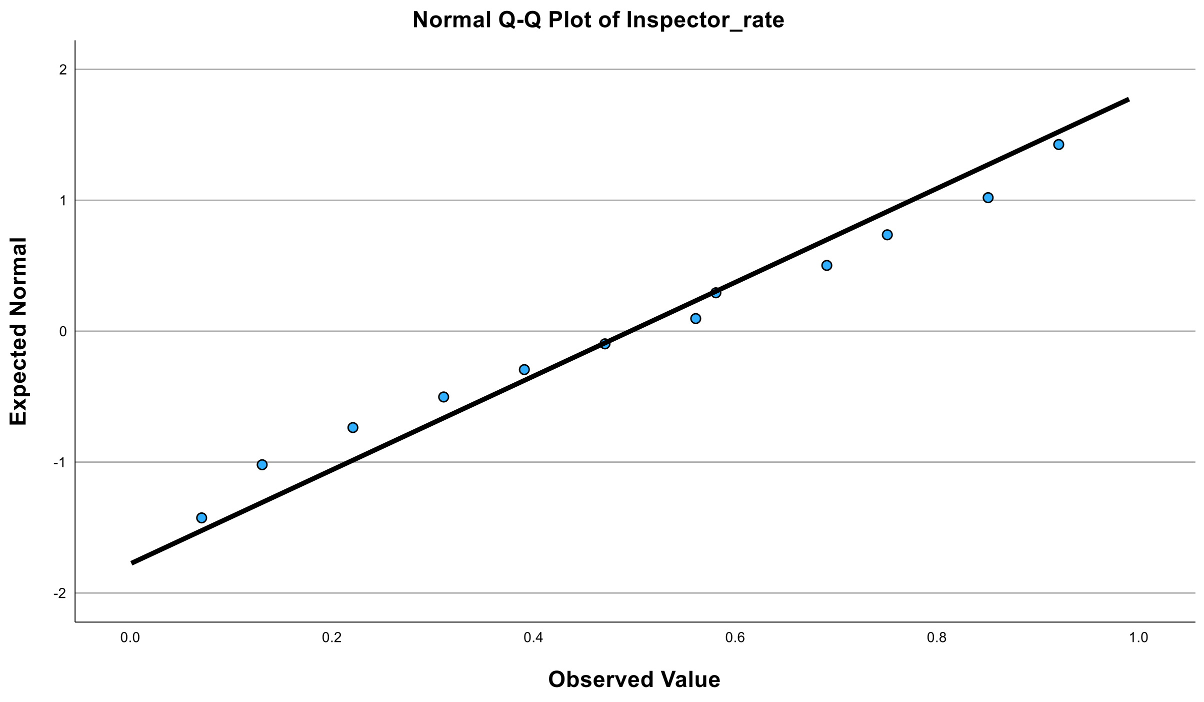

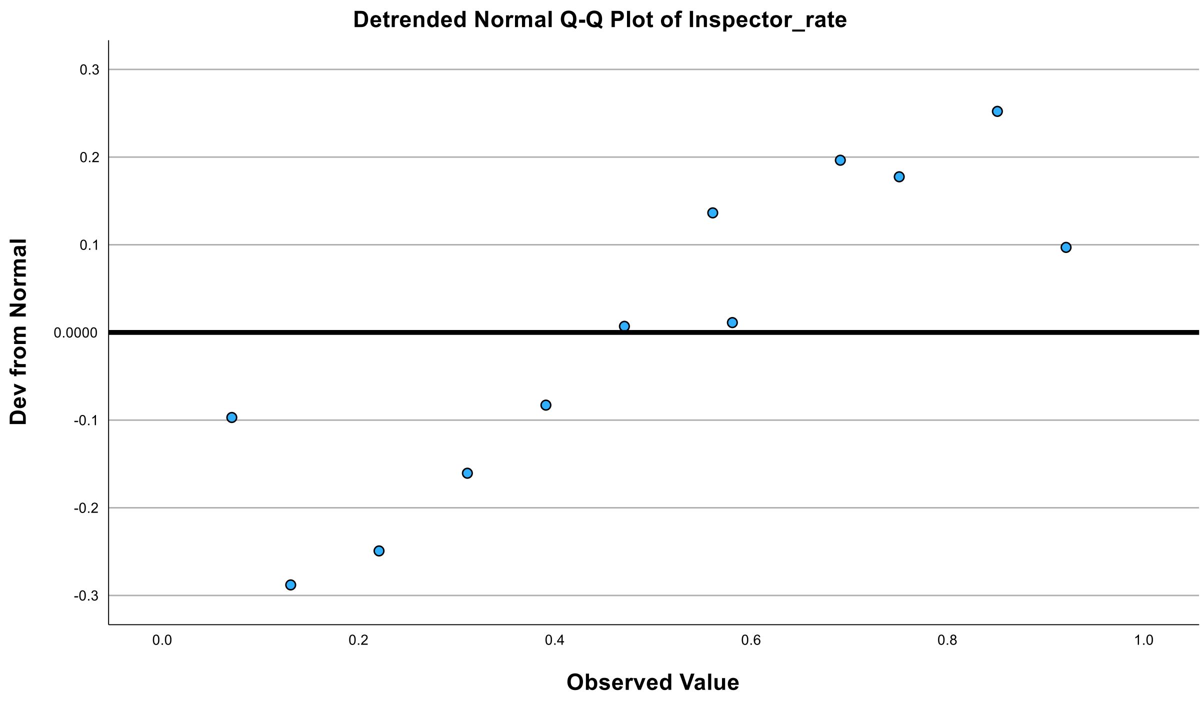
Detrended Normal Q-Q Plot of Inspector_rate
Dev from Normal
0.3
0.2
0.1
0.0000
-0.1
-0.2
-0.3
0.0
0.2
0.4
0.6
0.8
1.0
Observed Value

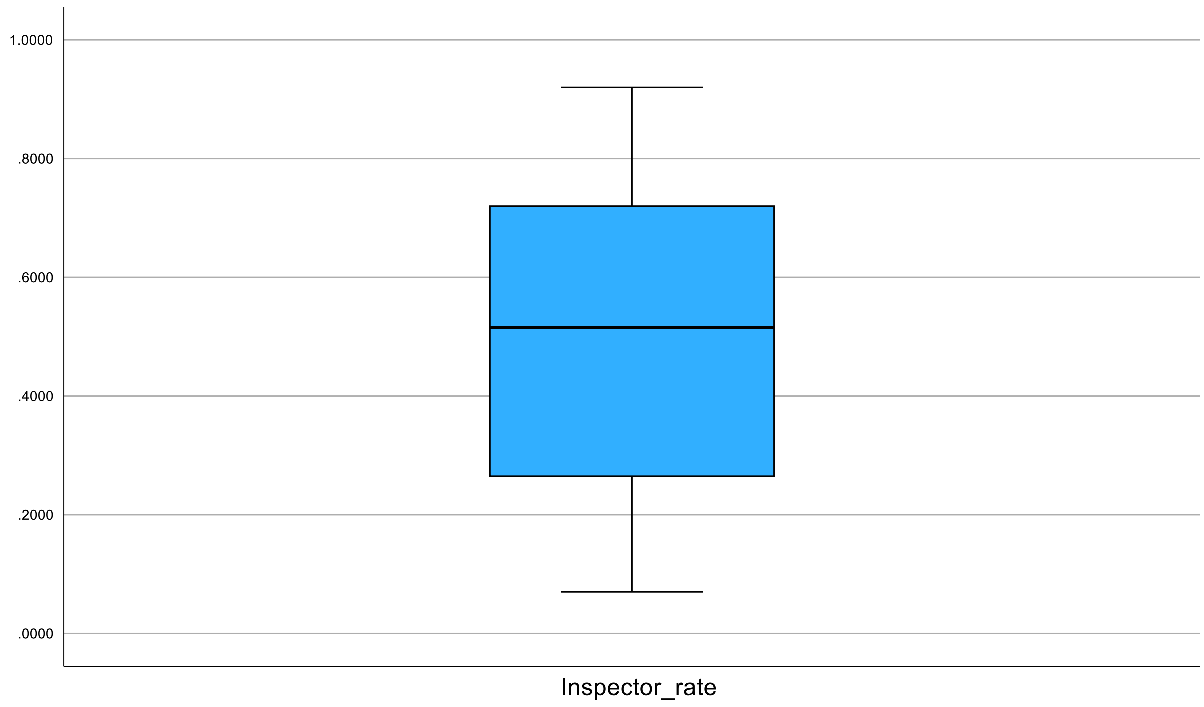
1.0000
.8000
.6000
.4000
.2000
.0000
Inspector_rate

Log_fatalrate

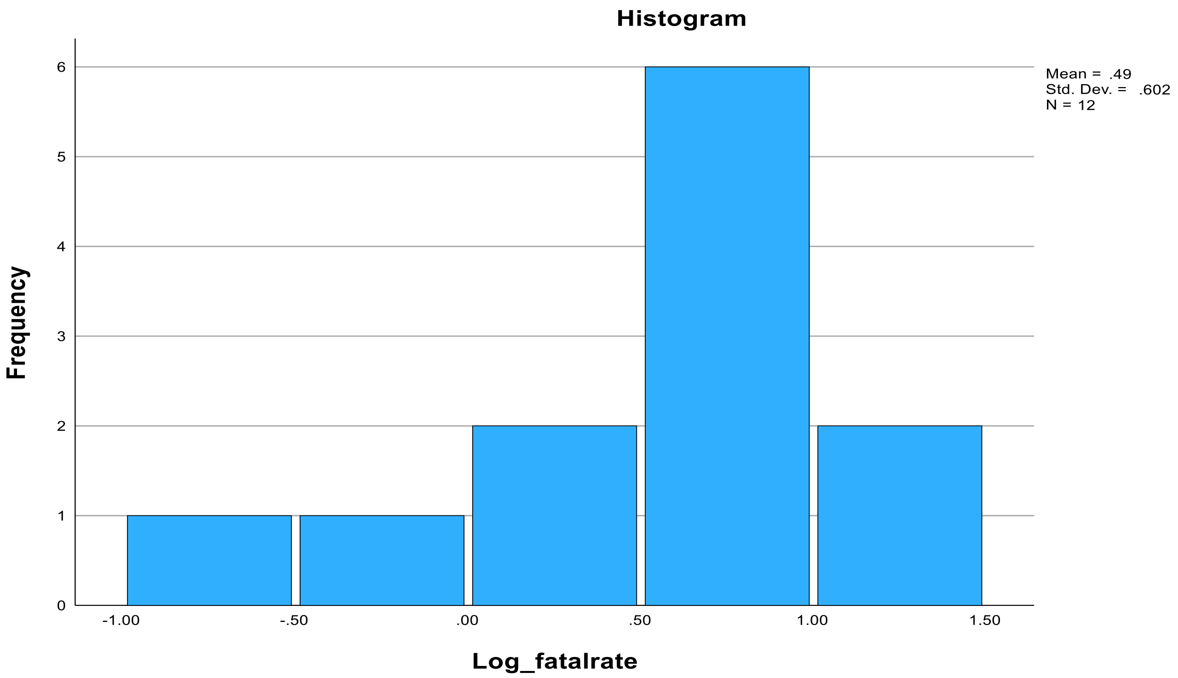

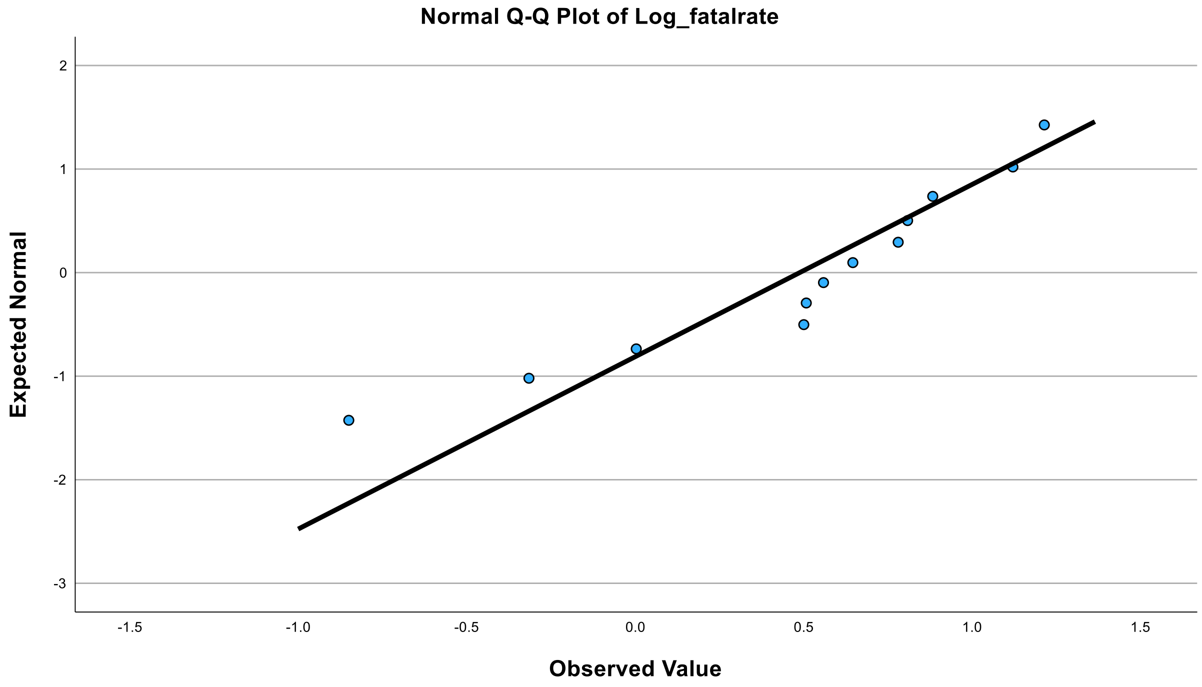

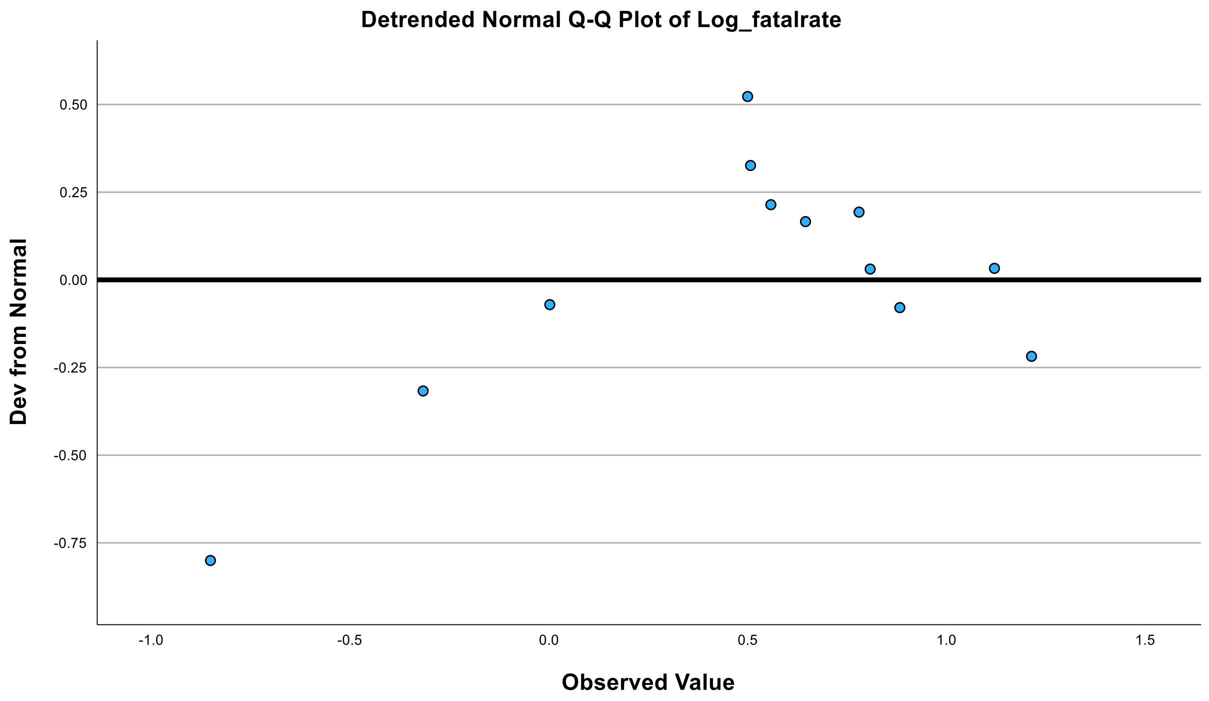
Detrended Normal Q-Q Plot of Log_fatalrate
Dev from Normal
Observed Value
0.50
0.25
0.00
-0.25
-0.50
-0.75
-1.0
-0.5
0.0
0.5
1.0
1.5

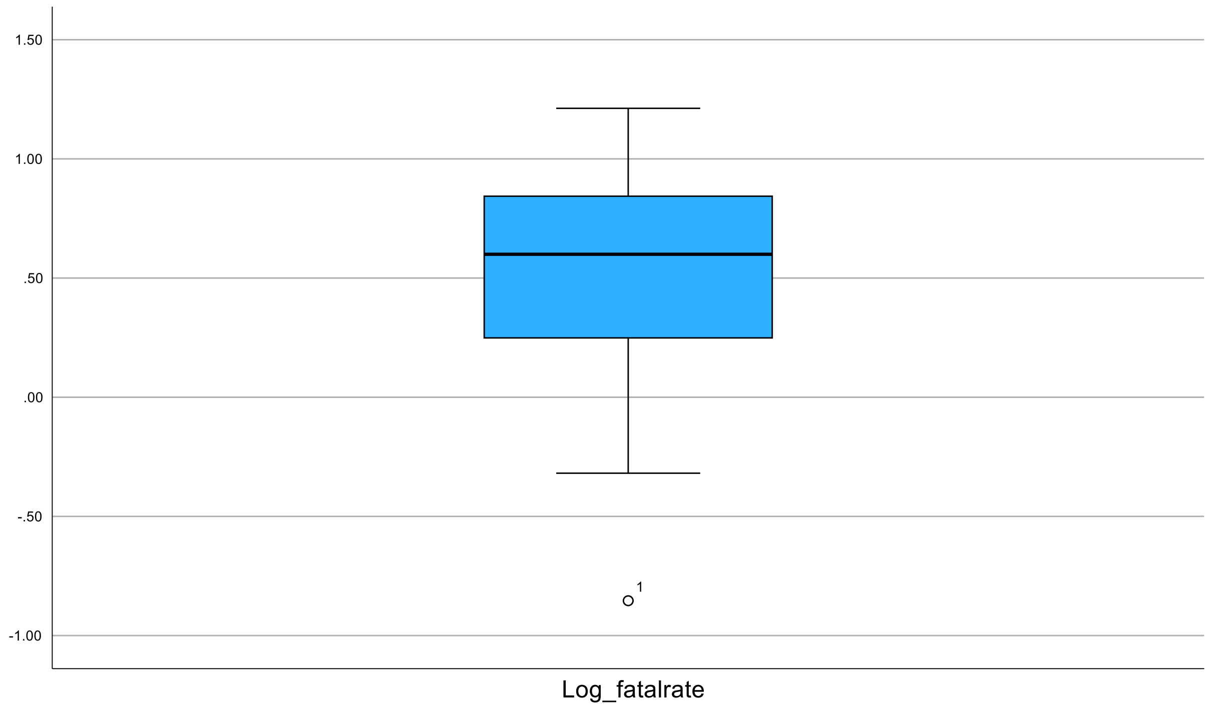
1.50
1.00
.50
.00
-.50
-1.00
1
Log_fatalrate

Log_nonfatalrate

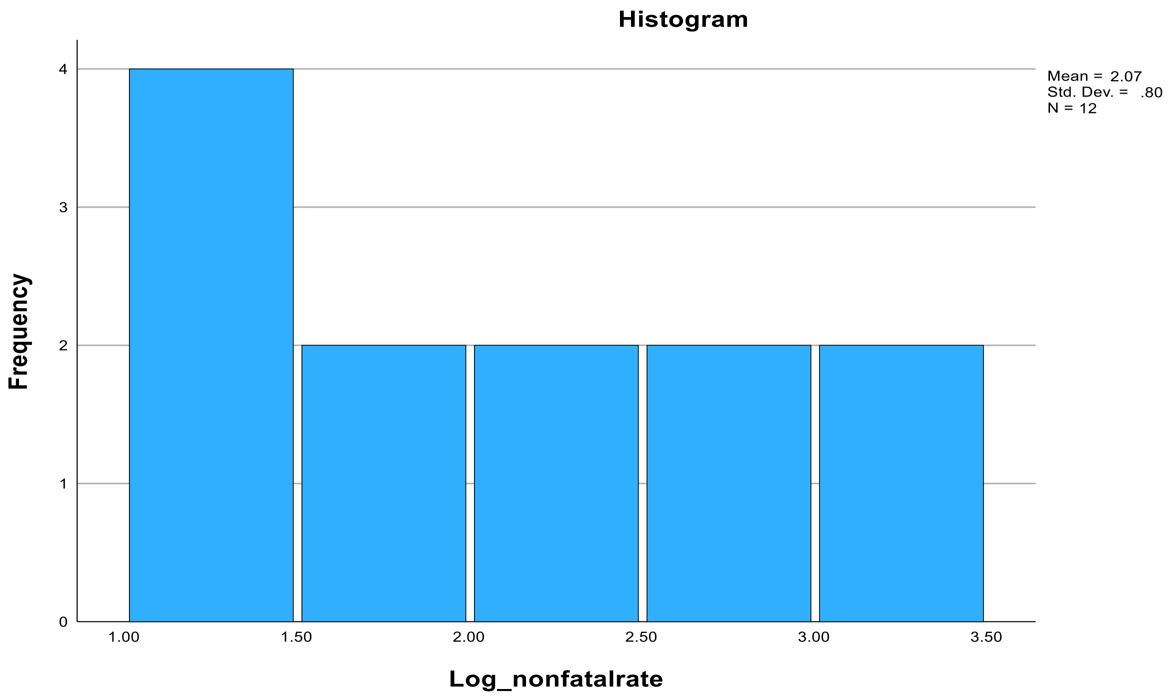

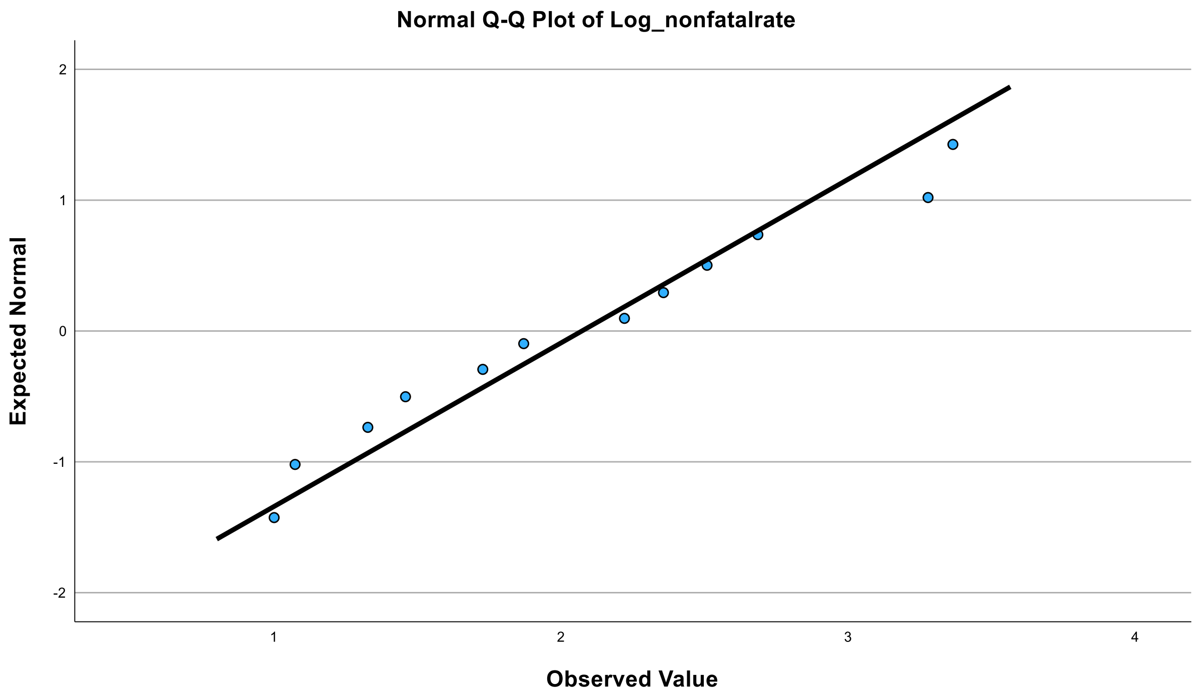

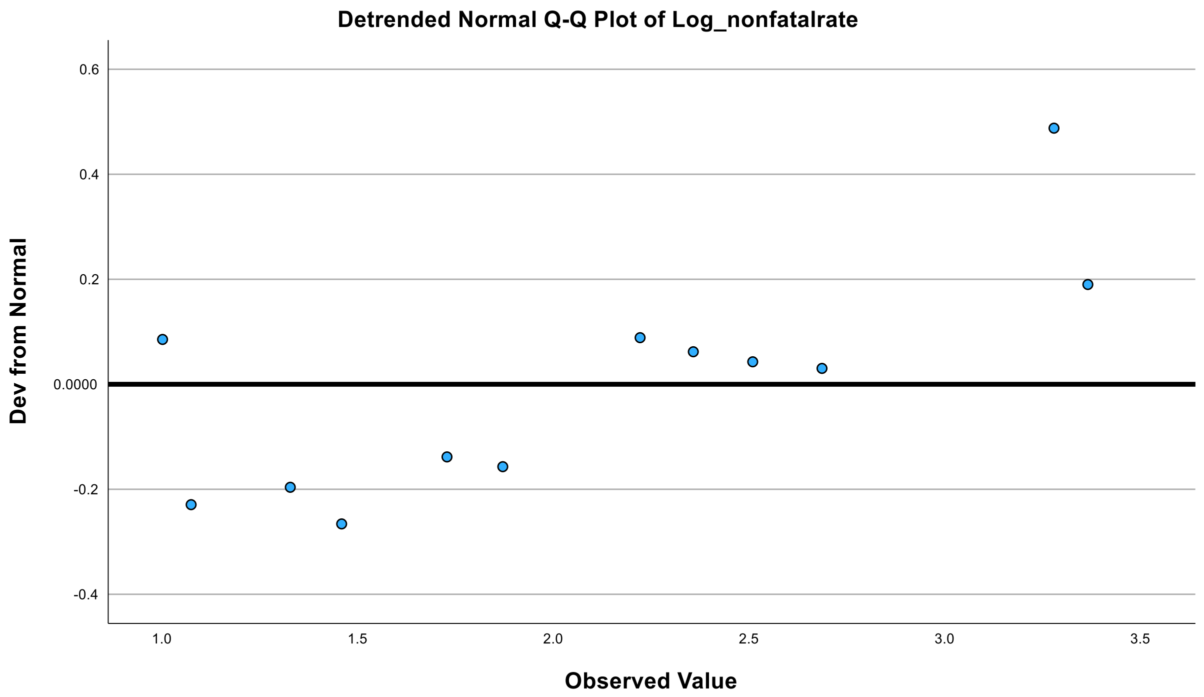
Detrended Normal Q-Q Plot of Log_nonfatalrate
Dev from Normal
0.6
0.4
0.2
0.0000
-0.2
-0.4
1.0
1.5
2.0
2.5
3.0
3.5
Observed Value

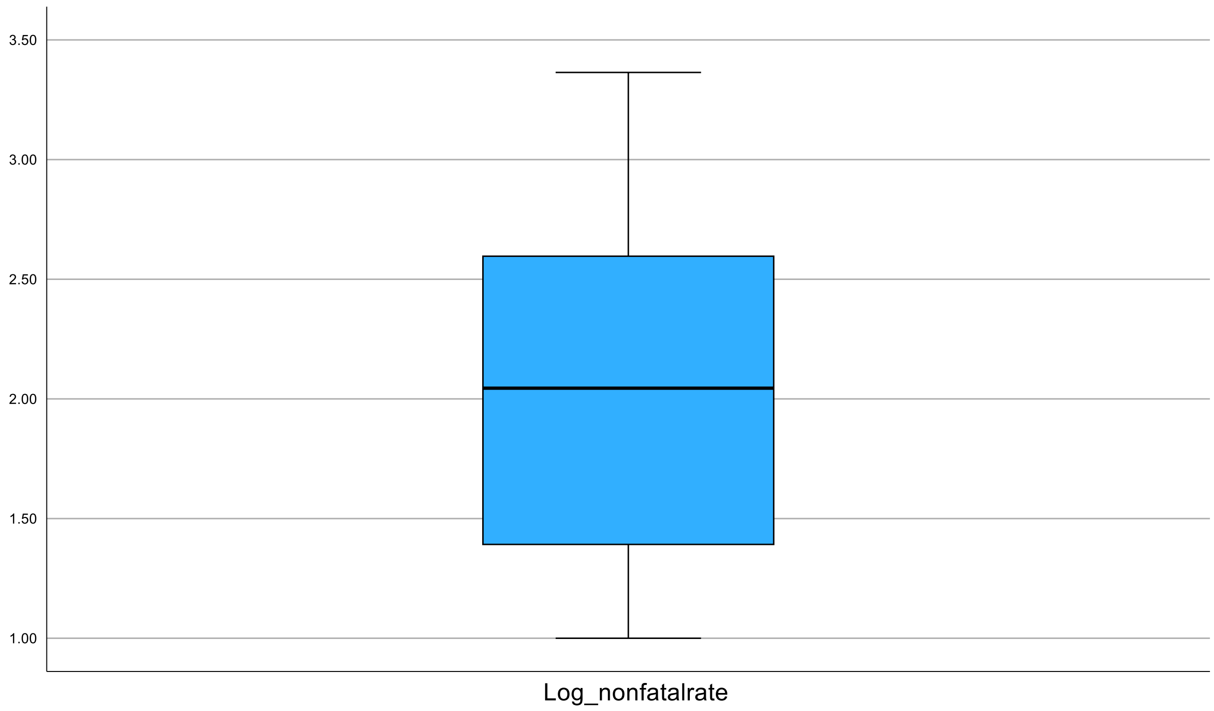
3.50
3.00
2.50
2.00
1.50
1.00
Log_nonfatalrate

Log-general linear multivariate regression with bootstrap-2000 with Confidence Intervals of 95% (PP. 34-40)

Bootstrap

Notes

Output Created		24-FEB-2026 09:43:08
Comments		
Input	Data	E:\Group 3 and 4 - Feb 24\Groups 3 and 4 - Descriptive and regression analysis.sav
	Active Dataset	DataSet0
	Filter	<none>
	Weight	<none>
	Split File	<none>
	N of Rows in Working Data File	12
Syntax		BOOTSTRAP<br>/SAMPLING METHOD=SIMPLE<br>/VARIABLES TARGET=Log_fatalrate Log_nonfatalrate INPUT= Inspector_ratio<br>/CRITERIA CILEVEL=95 CITYPE=PERCENTILE NSAMPLES=2000<br>/MISSING USERMISSING=EXCLUDE.
Resources	Processor Time	00:00:00.02
	Elapsed Time	00:00:00.01

Bootstrap Specifications

Sampling Method	Simple
Number of Samples	2000
Confidence Interval Level	95.0%
Confidence Interval Type	Percentile

General Linear Model

Notes

Output Created		24-FEB-2026 09:43:09
Comments		
Input	Data	E:\Group 3 and 4 - Feb 24\Groups 3 and 4 - Descriptive and regression analysis.sav
	Active Dataset	DataSet0
	Filter	<none>
	Weight	<none>
	Split File	<none>
	N of Rows in Working Data File	24012
Missing Value Handling	Definition of Missing	User-defined missing values are treated as missing.

	Cases Used	Statistics are based on all cases with valid data for all variables in the model.
Syntax		GLM Log_fatalrate Log_nonfatalrate<br>WITH Inspector_ratio<br>/METHOD=SSTYPE(3)<br>/INTERCEPT=INCLUDE<br>/SAVE=PRED SEPRED RESID ZRESID COOK LEVER<br>/EMMEANS=TABLES(OVERALL) WITH(Inspector_ratio=MEAN)<br>/PRINT=DESCRIPTIVE ETASQ OPOWER PARAMETER TEST(SSCP) RSSCP HOMOGENEITY LOF<br>/PLOT=SPREADLEVEL RESIDUALS<br>/CRITERIA=ALPHA(.05)<br>/DESIGN=Inspector_ratio.
Resources	Processor Time	00:00:26.34
	Elapsed Time	00:00:26.22

Warnings

The HOMOGENEITY specification in the PRINT subcommand will be ignored because there are no between-subjects factors.
The SPREADLEVEL specification has been found in the PLOT subcommand, but the model includes no factors. The spread versus level plot will not be produced.
This command is trying to save new variables while bootstrapping is in effect. The new variables will be lost.<br>Execution of this command stops.

Descriptive Statistics

	Mean	Std. Deviation	N
Log_fatalrate	.4855	.60159	12
Log_nonfatalrate	2.0719	.79955	12

Bartlett's Test of Sphericity[a]

Likelihood Ratio	.061
Approx. Chi-Square	4.194
df	2
Sig.	.123

Tests the null hypothesis that the residual covariance matrix is proportional to an identity matrix.[a]

a. Design: Intercept + Inspector_ratio

Multivariate Tests[a]

Effect		Value	F	Hypothesis df	Error df	Sig.
Intercept	Pillai's Trace	.590	6.481[b]	2.000	9.000	.018
	Wilks' Lambda	.410	6.481[b]	2.000	9.000	.018
	Hotelling's Trace	1.440	6.481[b]	2.000	9.000	.018
	Roy's Largest Root	1.440	6.481[b]	2.000	9.000	.018
Inspector_ratio	Pillai's Trace	.040	.187[b]	2.000	9.000	.832
	Wilks' Lambda	.960	.187[b]	2.000	9.000	.832
	Hotelling's Trace	.042	.187[b]	2.000	9.000	.832
	Roy's Largest Root	.042	.187[b]	2.000	9.000	.832

Multivariate Tests[a]

Effect		Partial Eta Squared	Noncent. Parameter	Observed Power[c]
Intercept	Pillai's Trace	.590	12.962	.777
	Wilks' Lambda	.590	12.962	.777
	Hotelling's Trace	.590	12.962	.777
	Roy's Largest Root	.590	12.962	.777
Inspector_ratio	Pillai's Trace	.040	.375	.071
	Wilks' Lambda	.040	.375	.071
	Hotelling's Trace	.040	.375	.071
	Roy's Largest Root	.040	.375	.071

a. Design: Intercept + Inspector_ratio
b. Exact statistic
c. Computed using alpha = .05

Tests of Between-Subjects Effects

Source	Dependent Variable	Type III Sum of Squares	df	Mean Square	F	Sig.
Corrected Model	Log_fatalrate	.001[a]	1	.001	.001	.971
	Log_nonfatalrate	.177[b]	1	.177	.259	.622
Intercept	Log_fatalrate	.671	1	.671	1.686	.223
	Log_nonfatalrate	9.232	1	9.232	13.468	.004
Inspector_ratio	Log_fatalrate	.001	1	.001	.001	.971
	Log_nonfatalrate	.177	1	.177	.259	.622
Error	Log_fatalrate	3.980	10	.398		
	Log_nonfatalrate	6.855	10	.685		
Total	Log_fatalrate	6.810	12			
	Log_nonfatalrate	58.548	12			
Corrected Total	Log_fatalrate	3.981	11			
	Log_nonfatalrate	7.032	11			

Tests of Between-Subjects Effects

Source	Dependent Variable	Partial Eta Squared	Noncent. Parameter	Observed Power[c]
Corrected Model	Log_fatalrate	.000	.001	.050
	Log_nonfatalrate	.025	.259	.075
Intercept	Log_fatalrate	.144	1.686	.217

	Log_nonfatalrate	.574	13.468	.910
Inspector_ratio	Log_fatalrate	.000	.001	.050
	Log_nonfatalrate	.025	.259	.075
Error	Log_fatalrate			
	Log_nonfatalrate			
Total	Log_fatalrate			
	Log_nonfatalrate			
Corrected Total	Log_fatalrate			
	Log_nonfatalrate			

a. R Squared = .000 (Adjusted R Squared = -.100)
b. R Squared = .025 (Adjusted R Squared = -.072)
c. Computed using alpha = .05

Parameter Estimates

Dependent Variable	Parameter	B	Std. Error	t	Sig.	95% Confidence Interval Lower Bound
Log_fatalrate	Intercept	.498	.383	1.298	.223	-.357
	Inspector_ratio	-.025	.682	-.037	.971	-1.544
Log_nonfatalrate	Intercept	1.847	.503	3.670	.004	.726
	Inspector_ratio	.455	.895	.508	.622	-1.538

Parameter Estimates

Dependent Variable	Parameter	95% Confidence Interval Upper Bound	Partial Eta Squared	Noncent. Parameter	Observed Power[a]
Log_fatalrate	Intercept	1.352	.144	1.298	.217
	Inspector_ratio	1.494	.000	.037	.050
Log_nonfatalrate	Intercept	2.968	.574	3.670	.910
	Inspector_ratio	2.448	.025	.508	.075

a. Computed using alpha = .05

Bootstrap for Parameter Estimates

			Bootstrap[a]			
Dependent Variable	Parameter	B	Bias	Std. Error	Sig. (2-tailed)	95% Confidence Interval Lower
Log_fatalrate	Intercept	.498	-.012	.261	.047	-.102
	Inspector_ratio	-.025	.025	.474	.953	-.917
Log_nonfatalrate	Intercept	1.847	.028	.522	.015	.965
	Inspector_ratio	.455	-.004	.853	.582	-1.354

Bootstrap for Parameter Estimates

Dependent Variable	Parameter	Bootstrap 95% Confidence Interval Upper
Log_fatalrate	Intercept	.943
	Inspector_ratio	.894
Log_nonfatalrate	Intercept	3.018
	Inspector_ratio	2.199

a. Unless otherwise noted, bootstrap results are based on 2000 bootstrap samples

Between-Subjects SSCP Matrix

			Log_fatalrate	Log_nonfatalrate
Hypothesis	Intercept	Log_fatalrate	.671	2.489
		Log_nonfatalrate	2.489	9.232
	Inspector_ratio	Log_fatalrate	.001	-.010
		Log_nonfatalrate	-.010	.177
Error		Log_fatalrate	3.980	2.978
		Log_nonfatalrate	2.978	6.855

Based on Type III Sum of Squares

Residual SSCP Matrix

		Log_fatalrate	Log_nonfatalrate
Sum-of-Squares and Cross-Products	Log_fatalrate	3.980	2.978
	Log_nonfatalrate	2.978	6.855
Covariance	Log_fatalrate	.398	.298
	Log_nonfatalrate	.298	.685
Correlation	Log_fatalrate	1.000	.570
	Log_nonfatalrate	.570	1.000

Based on Type III Sum of Squares

Lack of Fit

Multivariate Tests

Dependent Variables		Value	F	Hypothesis df	Error df
Log_fatalrate, Log_nonfatalrate	Pillai's Trace	.[a]	.	.	.
	Wilks' Lambda	.[a]	.	.	.
	Hotelling's Trace	.[a]	.	.	.
	Roy's Largest Root	.[a]	.	.	.
Log_fatalrate	Pillai's Trace	.[a]	.	.	.
	Wilks' Lambda	.[a]	.	.	.
	Hotelling's Trace	.[a]	.	.	.
	Roy's Largest Root	.[a]	.	.	.
Log_nonfatalrate	Pillai's Trace	.[a]	.	.	.
	Wilks' Lambda	.[a]	.	.	.
	Hotelling's Trace	.[a]	.	.	.
	Roy's Largest Root	.[a]	.	.	.

Multivariate Tests

Dependent Variables		Sig.	Partial Eta Squared	Noncent. Parameter
Log_fatalrate, Log_nonfatalrate	Pillai's Trace	.	.	.
	Wilks' Lambda	.	.	.
	Hotelling's Trace	.	.	.
	Roy's Largest Root	.	.	.
Log_fatalrate	Pillai's Trace	.	.	.
	Wilks' Lambda	.	.	.
	Hotelling's Trace	.	.	.
	Roy's Largest Root	.	.	.
Log_nonfatalrate	Pillai's Trace	.	.	.
	Wilks' Lambda	.	.	.
	Hotelling's Trace	.	.	.
	Roy's Largest Root	.	.	.

Multivariate Tests

Dependent Variables		Observed Power[b]
Log_fatalrate, Log_nonfatalrate	Pillai's Trace	.
	Wilks' Lambda	.
	Hotelling's Trace	.
	Roy's Largest Root	.
Log_fatalrate	Pillai's Trace	.
	Wilks' Lambda	.
	Hotelling's Trace	.
	Roy's Largest Root	.
Log_nonfatalrate	Pillai's Trace	.
	Wilks' Lambda	.
	Hotelling's Trace	.
	Roy's Largest Root	.

a. The pure error sub-matrix corresponding to the selected dependent variables is a zero matrix.
b. Computed using alpha = .05

Univariate Tests

Dependent Variable	Source	Sum of Squares	df	Mean Square	F	Sig.
Log_fatalrate	Lack of Fit	3.980	10	.398	.	.
	Pure Error	.000	0	.		
Log_nonfatalrate	Lack of Fit	6.855	10	.685	.	.
	Pure Error	.000	0	.		

Univariate Tests

Dependent Variable	Source	Partial Eta Squared	Noncent. Parameter	Observed Power[a]
Log_fatalrate	Lack of Fit	1.000	.	.
	Pure Error			
Log_nonfatalrate	Lack of Fit	1.000	.	.
	Pure Error			

a. Computed using alpha = .05

SSCP Matrix

		Log_fatalrate	Log_nonfatalrate
Lack of Fit	Log_fatalrate	3.980	2.978
	Log_nonfatalrate	2.978	6.855
Pure Error	Log_fatalrate	.000	.000
	Log_nonfatalrate	.000	.000

Estimated Marginal Means

Grand Mean

Dependent Variable	Mean	Std. Error	95% Confidence Interval	
			Lower Bound	Upper Bound
Log_fatalrate	.486[a]	.182	.080	.891
Log_nonfatalrate	2.072[a]	.239	1.539	2.604

a. Covariates appearing in the model are evaluated at the following values: Inspector_ratio = .495000.

Continued.

Log-general linear multivariate regression analysis (PP. 41-47)

Notes

Output Created		24-FEB-2026 09:45:39
Comments		
Input	Data	E:\Group 3 and 4 - Feb 24\Groups 3 and 4 - Descriptive and regression analysis.sav
	Active Dataset	DataSet0
	Filter	<none>
	Weight	<none>
	Split File	<none>
	N of Rows in Working Data File	12
Missing Value Handling	Definition of Missing	User-defined missing values are treated as missing.
	Cases Used	Statistics are based on all cases with valid data for all variables in the model.
Syntax		GLM Log_fatalrate Log_nonfatalrate WITH Inspector_ratio /METHOD=SSTYPE(3) /INTERCEPT=INCLUDE /SAVE=PRED SEPRED RESID ZRESID COOK LEVER /EMMEANS=TABLES(OVERALL) WITH(Inspector_ratio=MEAN) /PRINT=DESCRIPTIVE ETASQ OPOWER PARAMETER TEST(SSCP) RSSCP HOMOGENEITY LOF /PLOT=SPREADLEVEL RESIDUALS /CRITERIA=ALPHA(.05) /DESIGN=Inspector_ratio.
Resources	Processor Time	00:00:00.34
	Elapsed Time	00:00:00.26
Variables Created or Modified	PRE_3	Predicted Value for Log_fatalrate
	PRE_4	Predicted Value for Log_nonfatalrate
	SEP_3	Standard Error of Predicted Value for Log_fatalrate
	SEP_4	Standard Error of Predicted Value for Log_nonfatalrate
	RES_3	Residual for Log_fatalrate
	RES_4	Residual for Log_nonfatalrate
	ZRE_3	Standardized Residual for Log_fatalrate
	ZRE_4	Standardized Residual for Log_nonfatalrate
	COO_3	Cook's Distance for Log_fatalrate
	COO_4	Cook's Distance for Log_nonfatalrate
	LEV_3	Uncentered Leverage Value for

	Log_fatalrate
LEV_4	Uncentered Leverage Value for Log_nonfatalrate

Warnings

The HOMOGENEITY specification in the PRINT subcommand will be ignored because there are no between-subjects factors.
The SPREADLEVEL specification has been found in the PLOT subcommand, but the model includes no factors. The spread versus level plot will not be produced.

Descriptive Statistics

	Mean	Std. Deviation	N
Log_fatalrate	.4855	.60159	12
Log_nonfatalrate	2.0719	.79955	12

Bartlett's Test of Sphericity[a]

Likelihood Ratio	.061
Approx. Chi-Square	4.194
df	2
Sig.	.123

Tests the null hypothesis that the residual covariance matrix is proportional to an identity matrix.[a]
a. Design: Intercept + Inspector_ratio

Multivariate Tests[a]

Effect		Value	F	Hypothesis df	Error df	Sig.
Intercept	Pillai's Trace	.590	6.481[b]	2.000	9.000	.018
	Wilks' Lambda	.410	6.481[b]	2.000	9.000	.018
	Hotelling's Trace	1.440	6.481[b]	2.000	9.000	.018
	Roy's Largest Root	1.440	6.481[b]	2.000	9.000	.018
Inspector_ratio	Pillai's Trace	.040	.187[b]	2.000	9.000	.832
	Wilks' Lambda	.960	.187[b]	2.000	9.000	.832
	Hotelling's Trace	.042	.187[b]	2.000	9.000	.832
	Roy's Largest Root	.042	.187[b]	2.000	9.000	.832

Multivariate Tests[a]

Effect		Partial Eta Squared	Noncent. Parameter	Observed Power[c]
Intercept	Pillai's Trace	.590	12.962	.777
	Wilks' Lambda	.590	12.962	.777
	Hotelling's Trace	.590	12.962	.777
	Roy's Largest Root	.590	12.962	.777
Inspector_ratio	Pillai's Trace	.040	.375	.071
	Wilks' Lambda	.040	.375	.071
	Hotelling's Trace	.040	.375	.071
	Roy's Largest Root	.040	.375	.071

a. Design: Intercept + Inspector_ratio
b. Exact statistic

c. Computed using alpha = .05

Tests of Between-Subjects Effects

Source	Dependent Variable	Type III Sum of Squares	df	Mean Square	F	Sig.
Corrected Model	Log_fatalrate	.001[a]	1	.001	.001	.971
	Log_nonfatalrate	.177[b]	1	.177	.259	.622
Intercept	Log_fatalrate	.671	1	.671	1.686	.223
	Log_nonfatalrate	9.232	1	9.232	13.468	.004
Inspector_ratio	Log_fatalrate	.001	1	.001	.001	.971
	Log_nonfatalrate	.177	1	.177	.259	.622
Error	Log_fatalrate	3.980	10	.398		
	Log_nonfatalrate	6.855	10	.685		
Total	Log_fatalrate	6.810	12			
	Log_nonfatalrate	58.548	12			
Corrected Total	Log_fatalrate	3.981	11			
	Log_nonfatalrate	7.032	11			

Tests of Between-Subjects Effects

Source	Dependent Variable	Partial Eta Squared	Noncent. Parameter	Observed Power[c]
Corrected Model	Log_fatalrate	.000	.001	.050
	Log_nonfatalrate	.025	.259	.075
Intercept	Log_fatalrate	.144	1.686	.217
	Log_nonfatalrate	.574	13.468	.910
Inspector_ratio	Log_fatalrate	.000	.001	.050
	Log_nonfatalrate	.025	.259	.075
Error	Log_fatalrate			
	Log_nonfatalrate			
Total	Log_fatalrate			
	Log_nonfatalrate			
Corrected Total	Log_fatalrate			
	Log_nonfatalrate			

a. R Squared = .000 (Adjusted R Squared = -.100)
b. R Squared = .025 (Adjusted R Squared = -.072)
c. Computed using alpha = .05

Parameter Estimates

Dependent Variable	Parameter	B	Std. Error	t	Sig.	95% Confidence Interval Lower Bound
Log_fatalrate	Intercept	.498	.383	1.298	.223	-.357
	Inspector_ratio	-.025	.682	-.037	.971	-1.544
Log_nonfatalrate	Intercept	1.847	.503	3.670	.004	.726
	Inspector_ratio	.455	.895	.508	.622	-1.538

Parameter Estimates

Dependent Variable	Parameter	95% Confidence Interval Upper Bound	Partial Eta Squared	Noncent. Parameter	Observed Power[a]
Log_fatalrate	Intercept	1.352	.144	1.298	.217
	Inspector_ratio	1.494	.000	.037	.050
Log_nonfatalrate	Intercept	2.968	.574	3.670	.910
	Inspector_ratio	2.448	.025	.508	.075

a. Computed using alpha = .05

Between-Subjects SSCP Matrix

			Log_fatalrate	Log_nonfatalrate
Hypothesis	Intercept	Log_fatalrate	.671	2.489
		Log_nonfatalrate	2.489	9.232
	Inspector_ratio	Log_fatalrate	.001	-.010
		Log_nonfatalrate	-.010	.177
Error		Log_fatalrate	3.980	2.978
		Log_nonfatalrate	2.978	6.855

Based on Type III Sum of Squares

Residual SSCP Matrix

		Log_fatalrate	Log_nonfatalrate
Sum-of-Squares and Cross-Products	Log_fatalrate	3.980	2.978
	Log_nonfatalrate	2.978	6.855
Covariance	Log_fatalrate	.398	.298
	Log_nonfatalrate	.298	.685
Correlation	Log_fatalrate	1.000	.570
	Log_nonfatalrate	.570	1.000

Based on Type III Sum of Squares

Lack of Fit

Multivariate Tests

Dependent Variables		Value	F	Hypothesis df	Error df
Log_fatalrate, Log_nonfatalrate	Pillai's Trace	.[a]	.	.	.
	Wilks' Lambda	.[a]	.	.	.
	Hotelling's Trace	.[a]	.	.	.
	Roy's Largest Root	.[a]	.	.	.
Log_fatalrate	Pillai's Trace	.[a]	.	.	.
	Wilks' Lambda	.[a]	.	.	.
	Hotelling's Trace	.[a]	.	.	.
	Roy's Largest Root	.[a]	.	.	.
Log_nonfatalrate	Pillai's Trace	.[a]	.	.	.
	Wilks' Lambda	.[a]	.	.	.
	Hotelling's Trace	.[a]	.	.	.
	Roy's Largest Root	.[a]	.	.	.

Multivariate Tests

Dependent Variables		Sig.	Partial Eta Squared	Noncent. Parameter
Log_fatalrate, Log_nonfatalrate	Pillai's Trace	.	.	.
	Wilks' Lambda	.	.	.
	Hotelling's Trace	.	.	.
	Roy's Largest Root	.	.	.
Log_fatalrate	Pillai's Trace	.	.	.
	Wilks' Lambda	.	.	.
	Hotelling's Trace	.	.	.
	Roy's Largest Root	.	.	.
Log_nonfatalrate	Pillai's Trace	.	.	.
	Wilks' Lambda	.	.	.
	Hotelling's Trace	.	.	.
	Roy's Largest Root	.	.	.

Multivariate Tests

Dependent Variables		Observed Power[b]
Log_fatalrate, Log_nonfatalrate	Pillai's Trace	.
	Wilks' Lambda	.
	Hotelling's Trace	.
	Roy's Largest Root	.
Log_fatalrate	Pillai's Trace	.
	Wilks' Lambda	.
	Hotelling's Trace	.
	Roy's Largest Root	.
Log_nonfatalrate	Pillai's Trace	.
	Wilks' Lambda	.
	Hotelling's Trace	.
	Roy's Largest Root	.

a. The pure error sub-matrix corresponding to the selected dependent variables is a zero matrix.
b. Computed using alpha = .05

Univariate Tests

Dependent Variable	Source	Sum of Squares	df	Mean Square	F	Sig.
Log_fatalrate	Lack of Fit	3.980	10	.398	.	.
	Pure Error	.000	0	.		
Log_nonfatalrate	Lack of Fit	6.855	10	.685	.	.
	Pure Error	.000	0	.		

Univariate Tests

Dependent Variable	Source	Partial Eta Squared	Noncent. Parameter	Observed Power[a]
Log_fatalrate	Lack of Fit	1.000	.	.
	Pure Error			
Log_nonfatalrate	Lack of Fit	1.000	.	.
	Pure Error			

a. Computed using alpha = .05

SSCP Matrix

		Log_fatalrate	Log_nonfatalrate
Lack of Fit	Log_fatalrate	3.980	2.978
	Log_nonfatalrate	2.978	6.855
Pure Error	Log_fatalrate	.000	.000
	Log_nonfatalrate	.000	.000

Estimated Marginal Means

Grand Mean

Dependent Variable	Mean	Std. Error	95% Confidence Interval Lower Bound	95% Confidence Interval Upper Bound
Log_fatalrate	.486[a]	.182	.080	.891
Log_nonfatalrate	2.072[a]	.239	1.539	2.604

a. Covariates appearing in the model are evaluated at the following values: Inspector_ratio = .495000.

Observed * Predicted * Std. Residual Plots

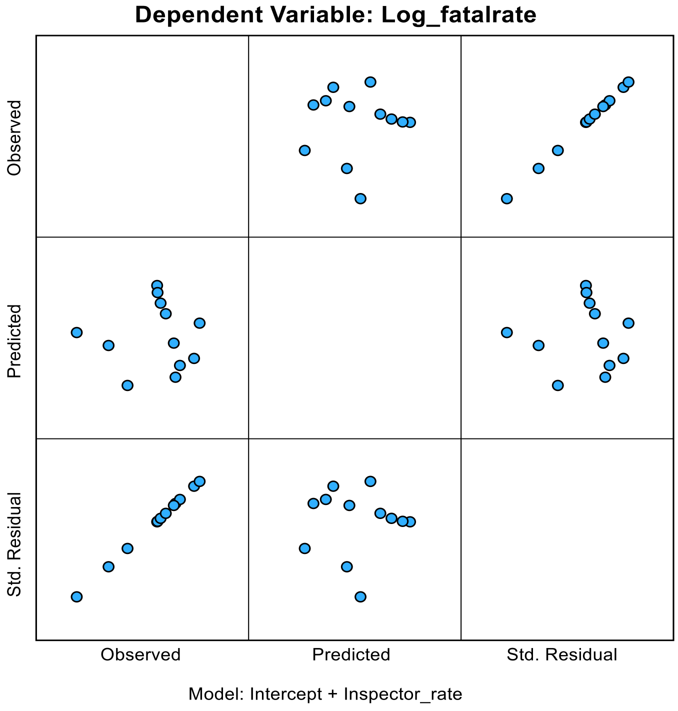

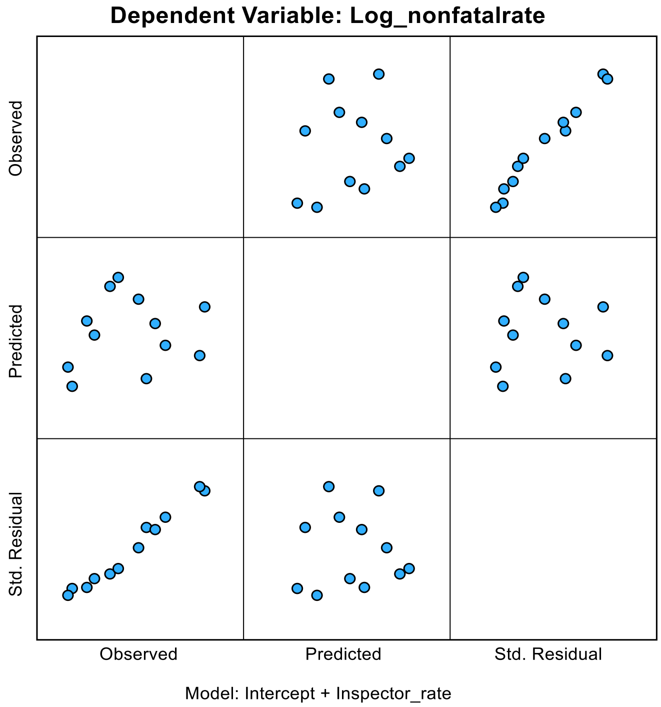

Thus, following analysis have been completed with IBM SPSS 30.0 for ILO's groups 3 and 4, as hereunder-

- Descriptive statistics with original data associated with the research variables,
- General linear multivariate regression,
- General linear multivariate regression with bootstrap-2000 with Confidence Intervals of 95%,
- Descriptive statistics with log-transformation of dependent variables,
- Log-general linear multivariate regression with bootstrap-2000 with Confidence Intervals of 95% and
- Log-general linear multivariate regression analysis.
