# Supplementary material for: OSH inspector ratio to strengthen decent workplace safety and health: a cross-regional trend analysis research
Source: Front Public Health. 2026 Apr 8;14:1806383. doi: 10.3389/fpubh.2026.1806383 (PMC13099828; doi:10.3389/fpubh.2026.1806383)
Supplement: Supplementary file 1 [file Data_Sheet_1.zip › Supplementary document Malaysia - Descriptive and multivariate regression.docx]

Supplementary Material

**Malaysia – Descriptive and multivariate regression analysis**

Malaysia is a member nation of International Labour Organization (ILO). IBM SPSS 30.0 software was used to complete descriptive statistics and multivariate regression analysis for Malaysia, with longitudinal data for eight different years. There are six major sections in this supplementary material as listed hereunder:

1. Descriptive statistics with original data associated with the research variables (PP. 2-10),
2. General linear multivariate regression (PP. 11-17),
3. General linear multivariate regression with bootstrap-2000 with Confidence Intervals of 95% (PP. 18-24),
4. Descriptive statistics with log-transformation of dependent variables (PP. 25-33),
5. Log-general linear multivariate regression with bootstrap-2000 with Confidence Intervals of 95% (PP. 34-40), and
6. Log-general linear multivariate regression analysis (PP. 41-47).

**Note:**

- Inspector ratio or inspector_ratio, or inspector_rate = Independent Variable (IV),
- Falta injury rate or fatal_rate = Dependent Variable (DV1), and
- Nonfatal injury rate or nonfatal_rate = Dependent Variable (DV2).

**Descriptive statistics with original research data associated with research variables (PP. 2-10)**

**Explore**

| **Notes** | | |
| --- | --- | --- |
| Output Created | | 24-FEB-2026 08:54:12 |
| Comments | |  |
| Input | Data | E:\Malaysia - Feb 24\Malaysia - Descriptive and multivariate regression.sav |
|  | Active Dataset | DataSet2 |
|  | Filter | <none> |
|  | Weight | <none> |
|  | Split File | <none> |
|  | N of Rows in Working Data File | 8 |
| Missing Value Handling | Definition of Missing | User-defined missing values for dependent variables are treated as missing. |
|  | Cases Used | Statistics are based on cases with no missing values for any dependent variable or factor used. |
| Syntax | | EXAMINE VARIABLES=Inspector_ratio Fatal_rate Nonfatal_rate /PLOT BOXPLOT HISTOGRAM NPPLOT /COMPARE GROUPS /STATISTICS DESCRIPTIVES EXTREME /CINTERVAL 95 /MISSING LISTWISE /NOTOTAL. |
| Resources | Processor Time | 00:00:05.31 |
|  | Elapsed Time | 00:00:01.90 |

| **Case Processing Summary** | | | | | | |
| --- | --- | --- | --- | --- | --- | --- |
|  | Cases | | | | | |
|  | Valid | | Missing | | Total | |
|  | N | Percent | N | Percent | N | Percent |
| Inspector_ratio | 8 | 100.0% | 0 | 0.0% | 8 | 100.0% |
| Fatal_rate | 8 | 100.0% | 0 | 0.0% | 8 | 100.0% |
| Nonfatal_rate | 8 | 100.0% | 0 | 0.0% | 8 | 100.0% |

| **Descriptives** | | | | |
| --- | --- | --- | --- | --- |
|  | | | Statistic | Std. Error |
| Inspector_ratio | Mean | | .751300 | .0081439 |
|  | 95% Confidence Interval for Mean | Lower Bound | .732043 |  |
|  |  | Upper Bound | .770557 |  |
|  | 5% Trimmed Mean | | .751217 |  |
|  | Median | | .748150 |  |
|  | Variance | | .001 |  |
|  | Std. Deviation | | .0230344 |  |
|  | Minimum | | .7147 |  |
|  | Maximum | | .7894 |  |
|  | Range | | .0747 |  |
|  | Interquartile Range | | .0312 |  |
|  | Skewness | | .165 | .752 |
|  | Kurtosis | | .144 | 1.481 |
| Fatal_rate | Mean | | 3.242500 | .4577731 |
|  | 95% Confidence Interval for Mean | Lower Bound | 2.160039 |  |
|  |  | Upper Bound | 4.324961 |  |
|  | 5% Trimmed Mean | | 3.221111 |  |
|  | Median | | 2.975000 |  |
|  | Variance | | 1.676 |  |
|  | Std. Deviation | | 1.2947780 |  |
|  | Minimum | | 2.0300 |  |
|  | Maximum | | 4.8400 |  |
|  | Range | | 2.8100 |  |
|  | Interquartile Range | | 2.6050 |  |
|  | Skewness | | .233 | .752 |
|  | Kurtosis | | -2.298 | 1.481 |
| Nonfatal_rate | Mean | | 236.632500 | 15.7712924 |
|  | 95% Confidence Interval for Mean | Lower Bound | 199.339320 |  |
|  |  | Upper Bound | 273.925680 |  |
|  | 5% Trimmed Mean | | 239.251111 |  |
|  | Median | | 239.905000 |  |
|  | Variance | | 1989.869 |  |
|  | Std. Deviation | | 44.6079512 |  |
|  | Minimum | | 142.9700 |  |
|  | Maximum | | 283.1600 |  |
|  | Range | | 140.1900 |  |
|  | Interquartile Range | | 53.0250 |  |
|  | Skewness | | -1.367 | .752 |
|  | Kurtosis | | 2.466 | 1.481 |

| **Extreme Values**^a^ | | | | |
| --- | --- | --- | --- | --- |
|  | | | Case Number | Value |
| Inspector_ratio | Highest | 1 | 1 | .7894 |
|  |  | 2 | 6 | .7715 |
|  |  | 3 | 2 | .7615 |
|  |  | 4 | 3 | .7536 |
|  | Lowest | 1 | 8 | .7147 |
|  |  | 2 | 4 | .7371 |
|  |  | 3 | 7 | .7399 |
|  |  | 4 | 5 | .7427 |
| Fatal_rate | Highest | 1 | 1 | 4.8400 |
|  |  | 2 | 2 | 4.8400 |
|  |  | 3 | 3 | 4.1400 |
|  |  | 4 | 4 | 3.8300 |
|  | Lowest | 1 | 6 | 2.0300 |
|  |  | 2 | 8 | 2.0500 |
|  |  | 3 | 7 | 2.0900 |
|  |  | 4 | 5 | 2.1200 |
| Nonfatal_rate | Highest | 1 | 2 | 283.1600 |
|  |  | 2 | 1 | 276.1600 |
|  |  | 3 | 4 | 267.1700 |
|  |  | 4 | 8 | 243.9500 |
|  | Lowest | 1 | 6 | 142.9700 |
|  |  | 2 | 5 | 219.8800 |
|  |  | 3 | 7 | 223.9100 |
|  |  | 4 | 3 | 235.8600 |
| a. The requested number of extreme values exceeds the number of data points. A smaller number of extremes is displayed. | | | | |

| **Tests of Normality** | | | | | | |
| --- | --- | --- | --- | --- | --- | --- |
|  | Kolmogorov-Smirnov^a^ | | | Shapiro-Wilk | | |
|  | Statistic | df | Sig. | Statistic | df | Sig. |
| Inspector_ratio | .146 | 8 | .200^*^ | .985 | 8 | .983 |
| Fatal_rate | .307 | 8 | .025 | .787 | 8 | .021 |
| Nonfatal_rate | .229 | 8 | .200^*^ | .879 | 8 | .185 |
| *. This is a lower bound of the true significance. | | | | | | |
| a. Lilliefors Significance Correction | | | | | | |

**Inspector_ratio**

**Fatal_rate**

**Nonfatal_rate**

**General linear multivariate regression (PP. 11-17)**

| **Notes** | | |
| --- | --- | --- |
| Output Created | | 24-FEB-2026 08:58:39 |
| Comments | |  |
| Input | Data | E:\Malaysia - Feb 24\Malaysia - Descriptive and multivariate regression.sav |
|  | Active Dataset | DataSet2 |
|  | Filter | <none> |
|  | Weight | <none> |
|  | Split File | <none> |
|  | N of Rows in Working Data File | 8 |
| Missing Value Handling | Definition of Missing | User-defined missing values are treated as missing. |
|  | Cases Used | Statistics are based on all cases with valid data for all variables in the model. |
| Syntax | | GLM Fatal_rate Nonfatal_rate WITH Inspector_ratio /METHOD=SSTYPE(3) /INTERCEPT=INCLUDE /SAVE=PRED SEPRED RESID ZRESID COOK LEVER /EMMEANS=TABLES(OVERALL) WITH(Inspector_ratio=MEAN) /PRINT=DESCRIPTIVE ETASQ OPOWER PARAMETER TEST(SSCP) RSSCP HOMOGENEITY LOF /PLOT=SPREADLEVEL RESIDUALS /CRITERIA=ALPHA(.05) /DESIGN=Inspector_ratio. |
| Resources | Processor Time | 00:00:00.53 |
|  | Elapsed Time | 00:00:00.29 |
| Variables Created or Modified | PRE_1 | Predicted Value for Fatal_rate |
|  | PRE_2 | Predicted Value for Nonfatal_rate |
|  | SEP_1 | Standard Error of Predicted Value for Fatal_rate |
|  | SEP_2 | Standard Error of Predicted Value for Nonfatal_rate |
|  | RES_1 | Residual for Fatal_rate |
|  | RES_2 | Residual for Nonfatal_rate |
|  | ZRE_1 | Standardized Residual for Fatal_rate |
|  | ZRE_2 | Standardized Residual for Nonfatal_rate |
|  | COO_1 | Cook's Distance for Fatal_rate |
|  | COO_2 | Cook's Distance for Nonfatal_rate |
|  | LEV_1 | Uncentered Leverage Value for Fatal_rate |
|  | LEV_2 | Uncentered Leverage Value for Nonfatal_rate |

| **Warnings** |
| --- |
| The HOMOGENEITY specification in the PRINT subcommand will be ignored because there are no between-subjects factors. |
| The SPREADLEVEL specification has been found in the PLOT subcommand, but the model includes no factors. The spread versus level plot will not be produced. |

| **Descriptive Statistics** | | | |
| --- | --- | --- | --- |
|  | Mean | Std. Deviation | N |
| Fatal_rate | 3.242500 | 1.2947780 | 8 |
| Nonfatal_rate | 236.632500 | 44.6079512 | 8 |

| **Bartlett's Test of Sphericity**^a^ | |
| --- | --- |
| Likelihood Ratio | .000 |
| Approx. Chi-Square | 38.100 |
| df | 2 |
| Sig. | <.001 |
| Tests the null hypothesis that the residual covariance matrix is proportional to an identity matrix.^a^  a. Design: Intercept + Inspector_ratio | |

| **Multivariate Tests**^a^ | | | | | | |
| --- | --- | --- | --- | --- | --- | --- |
| Effect | | Value | F | Hypothesis df | Error df | Sig. |
| Intercept | Pillai's Trace | .728 | 6.705^b^ | 2.000 | 5.000 | .038 |
|  | Wilks' Lambda | .272 | 6.705^b^ | 2.000 | 5.000 | .038 |
|  | Hotelling's Trace | 2.682 | 6.705^b^ | 2.000 | 5.000 | .038 |
|  | Roy's Largest Root | 2.682 | 6.705^b^ | 2.000 | 5.000 | .038 |
| Inspector_ratio | Pillai's Trace | .694 | 5.677^b^ | 2.000 | 5.000 | .052 |
|  | Wilks' Lambda | .306 | 5.677^b^ | 2.000 | 5.000 | .052 |
|  | Hotelling's Trace | 2.271 | 5.677^b^ | 2.000 | 5.000 | .052 |
|  | Roy's Largest Root | 2.271 | 5.677^b^ | 2.000 | 5.000 | .052 |

| **Multivariate Tests**^a^ | | | | |
| --- | --- | --- | --- | --- |
| Effect | | Partial Eta Squared | Noncent. Parameter | Observed Power^c^ |
| Intercept | Pillai's Trace | .728 | 13.410 | .660 |
|  | Wilks' Lambda | .728 | 13.410 | .660 |
|  | Hotelling's Trace | .728 | 13.410 | .660 |
|  | Roy's Largest Root | .728 | 13.410 | .660 |
| Inspector_ratio | Pillai's Trace | .694 | 11.354 | .587 |
|  | Wilks' Lambda | .694 | 11.354 | .587 |
|  | Hotelling's Trace | .694 | 11.354 | .587 |
|  | Roy's Largest Root | .694 | 11.354 | .587 |
| a. Design: Intercept + Inspector_ratio | | | | |
| b. Exact statistic | | | | |
| c. Computed using alpha = .05 | | | | |

| **Tests of Between-Subjects Effects** | | | | | | |
| --- | --- | --- | --- | --- | --- | --- |
| Source | Dependent Variable | Type III Sum of Squares | df | Mean Square | F | Sig. |
| Corrected Model | Fatal_rate | 3.427^a^ | 1 | 3.427 | 2.475 | .167 |
|  | Nonfatal_rate | 28.531^b^ | 1 | 28.531 | .012 | .915 |
| Intercept | Fatal_rate | 2.521 | 1 | 2.521 | 1.820 | .226 |
|  | Nonfatal_rate | 601.542 | 1 | 601.542 | .260 | .629 |
| Inspector_ratio | Fatal_rate | 3.427 | 1 | 3.427 | 2.475 | .167 |
|  | Nonfatal_rate | 28.531 | 1 | 28.531 | .012 | .915 |
| Error | Fatal_rate | 8.308 | 6 | 1.385 |  |  |
|  | Nonfatal_rate | 13900.554 | 6 | 2316.759 |  |  |
| Total | Fatal_rate | 95.846 | 8 |  |  |  |
|  | Nonfatal_rate | 461888.606 | 8 |  |  |  |
| Corrected Total | Fatal_rate | 11.735 | 7 |  |  |  |
|  | Nonfatal_rate | 13929.085 | 7 |  |  |  |

| **Tests of Between-Subjects Effects** | | | | |
| --- | --- | --- | --- | --- |
| Source | Dependent Variable | Partial Eta Squared | Noncent. Parameter | Observed Power^c^ |
| Corrected Model | Fatal_rate | .292 | 2.475 | .264 |
|  | Nonfatal_rate | .002 | .012 | .051 |
| Intercept | Fatal_rate | .233 | 1.820 | .207 |
|  | Nonfatal_rate | .041 | .260 | .072 |
| Inspector_ratio | Fatal_rate | .292 | 2.475 | .264 |
|  | Nonfatal_rate | .002 | .012 | .051 |
| Error | Fatal_rate |  |  |  |
|  | Nonfatal_rate |  |  |  |
| Total | Fatal_rate |  |  |  |
|  | Nonfatal_rate |  |  |  |
| Corrected Total | Fatal_rate |  |  |  |
|  | Nonfatal_rate |  |  |  |
| a. R Squared = .292 (Adjusted R Squared = .174) | | | | |
| b. R Squared = .002 (Adjusted R Squared = -.164) | | | | |
| c. Computed using alpha = .05 | | | | |

| **Parameter Estimates** | | | | | | |
| --- | --- | --- | --- | --- | --- | --- |
| Dependent Variable | Parameter | B | Std. Error | t | Sig. | 95% Confidence Interval |
|  |  |  |  |  |  | Lower Bound |
| Fatal_rate | Intercept | -19.580 | 14.512 | -1.349 | .226 | -55.090 |
|  | Inspector_ratio | 30.378 | 19.308 | 1.573 | .167 | -16.867 |
| Nonfatal_rate | Intercept | 302.481 | 593.616 | .510 | .629 | -1150.045 |
|  | Inspector_ratio | -87.646 | 789.794 | -.111 | .915 | -2020.201 |

| **Parameter Estimates** | | | | | |
| --- | --- | --- | --- | --- | --- |
| Dependent Variable | Parameter | 95% Confidence Interval | Partial Eta Squared | Noncent. Parameter | Observed Power^a^ |
|  |  | Upper Bound |  |  |  |
| Fatal_rate | Intercept | 15.930 | .233 | 1.349 | .207 |
|  | Inspector_ratio | 77.623 | .292 | 1.573 | .264 |
| Nonfatal_rate | Intercept | 1755.007 | .041 | .510 | .072 |
|  | Inspector_ratio | 1844.909 | .002 | .111 | .051 |
| a. Computed using alpha = .05 | | | | | |

| **Between-Subjects SSCP Matrix** | | | | |
| --- | --- | --- | --- | --- |
|  | | | Fatal_rate | Nonfatal_rate |
| Hypothesis | Intercept | Fatal_rate | 2.521 | -38.939 |
|  |  | Nonfatal_rate | -38.939 | 601.542 |
|  | Inspector_ratio | Fatal_rate | 3.427 | -9.889 |
|  |  | Nonfatal_rate | -9.889 | 28.531 |
| Error | | Fatal_rate | 8.308 | 302.916 |
|  |  | Nonfatal_rate | 302.916 | 13900.554 |
| Based on Type III Sum of Squares | | | | |

| **Residual SSCP Matrix** | | | |
| --- | --- | --- | --- |
|  | | Fatal_rate | Nonfatal_rate |
| Sum-of-Squares and Cross-Products | Fatal_rate | 8.308 | 302.916 |
|  | Nonfatal_rate | 302.916 | 13900.554 |
| Covariance | Fatal_rate | 1.385 | 50.486 |
|  | Nonfatal_rate | 50.486 | 2316.759 |
| Correlation | Fatal_rate | 1.000 | .891 |
|  | Nonfatal_rate | .891 | 1.000 |
| Based on Type III Sum of Squares | | | |

**Lack of Fit**

| **Multivariate Tests** | | | | | |
| --- | --- | --- | --- | --- | --- |
| Dependent Variables | | Value | F | Hypothesis df | Error df |
| Fatal_rate, Nonfatal_rate | Pillai's Trace | .^a^ | . | . | . |
|  | Wilks' Lambda | .^a^ | . | . | . |
|  | Hotelling's Trace | .^a^ | . | . | . |
|  | Roy's Largest Root | .^a^ | . | . | . |
| Fatal_rate | Pillai's Trace | .^a^ | . | . | . |
|  | Wilks' Lambda | .^a^ | . | . | . |
|  | Hotelling's Trace | .^a^ | . | . | . |
|  | Roy's Largest Root | .^a^ | . | . | . |
| Nonfatal_rate | Pillai's Trace | .^a^ | . | . | . |
|  | Wilks' Lambda | .^a^ | . | . | . |
|  | Hotelling's Trace | .^a^ | . | . | . |
|  | Roy's Largest Root | .^a^ | . | . | . |

| **Multivariate Tests** | | | | |
| --- | --- | --- | --- | --- |
| Dependent Variables | | Sig. | Partial Eta Squared | Noncent. Parameter |
| Fatal_rate, Nonfatal_rate | Pillai's Trace | . | . | . |
|  | Wilks' Lambda | . | . | . |
|  | Hotelling's Trace | . | . | . |
|  | Roy's Largest Root | . | . | . |
| Fatal_rate | Pillai's Trace | . | . | . |
|  | Wilks' Lambda | . | . | . |
|  | Hotelling's Trace | . | . | . |
|  | Roy's Largest Root | . | . | . |
| Nonfatal_rate | Pillai's Trace | . | . | . |
|  | Wilks' Lambda | . | . | . |
|  | Hotelling's Trace | . | . | . |
|  | Roy's Largest Root | . | . | . |

| **Multivariate Tests** | | |
| --- | --- | --- |
| Dependent Variables | | Observed Power^b^ |
| Fatal_rate, Nonfatal_rate | Pillai's Trace | . |
|  | Wilks' Lambda | . |
|  | Hotelling's Trace | . |
|  | Roy's Largest Root | . |
| Fatal_rate | Pillai's Trace | . |
|  | Wilks' Lambda | . |
|  | Hotelling's Trace | . |
|  | Roy's Largest Root | . |
| Nonfatal_rate | Pillai's Trace | . |
|  | Wilks' Lambda | . |
|  | Hotelling's Trace | . |
|  | Roy's Largest Root | . |

| a. The pure error sub-matrix corresponding to the selected dependent variables is a zero matrix. |
| --- |
| b. Computed using alpha = .05 |

| **Univariate Tests** | | | | | | |
| --- | --- | --- | --- | --- | --- | --- |
| Dependent Variable | Source | Sum of Squares | df | Mean Square | F | Sig. |
| Fatal_rate | Lack of Fit | 8.308 | 6 | 1.385 | . | . |
|  | Pure Error | .000 | 0 | . |  |  |
| Nonfatal_rate | Lack of Fit | 13900.554 | 6 | 2316.759 | . | . |
|  | Pure Error | .000 | 0 | . |  |  |

| **Univariate Tests** | | | | |
| --- | --- | --- | --- | --- |
| Dependent Variable | Source | Partial Eta Squared | Noncent. Parameter | Observed Power^a^ |
| Fatal_rate | Lack of Fit | 1.000 | . | . |
|  | Pure Error |  |  |  |
| Nonfatal_rate | Lack of Fit | 1.000 | . | . |
|  | Pure Error |  |  |  |
| a. Computed using alpha = .05 | | | | |

| **SSCP Matrix** | | | |
| --- | --- | --- | --- |
|  | | Fatal_rate | Nonfatal_rate |
| Lack of Fit | Fatal_rate | 8.308 | 302.916 |
|  | Nonfatal_rate | 302.916 | 13900.554 |
| Pure Error | Fatal_rate | .000 | .000 |
|  | Nonfatal_rate | .000 | .000 |

**Estimated Marginal Means**

| **Grand Mean** | | | | |
| --- | --- | --- | --- | --- |
| Dependent Variable | Mean | Std. Error | 95% Confidence Interval | |
|  |  |  | Lower Bound | Upper Bound |
| Fatal_rate | 3.242^a^ | .416 | 2.225 | 4.260 |
| Nonfatal_rate | 236.633^a^ | 17.017 | 194.992 | 278.273 |
| a. Covariates appearing in the model are evaluated at the following values: Inspector_ratio = .751300. | | | | |

**Observed * Predicted * Std. Residual Plots**

**Continued.**

**General linear multivariate regression with bootstrap-2000 with Confidence Intervals of 95% (PP. 18-24)**

**Bootstrap**

| **Notes** | | |
| --- | --- | --- |
| Output Created | | 24-FEB-2026 09:02:26 |
| Comments | |  |
| Input | Data | E:\Malaysia - Feb 24\Malaysia - Descriptive and multivariate regression.sav |
|  | Active Dataset | DataSet2 |
|  | Filter | <none> |
|  | Weight | <none> |
|  | Split File | <none> |
|  | N of Rows in Working Data File | 8 |
| Syntax | | BOOTSTRAP /SAMPLING METHOD=SIMPLE /VARIABLES TARGET=Fatal_rate Nonfatal_rate INPUT= Inspector_ratio /CRITERIA CILEVEL=95 CITYPE=PERCENTILE NSAMPLES=2000 /MISSING USERMISSING=EXCLUDE. |
| Resources | Processor Time | 00:00:00.03 |
|  | Elapsed Time | 00:00:00.02 |

| **Bootstrap Specifications** | |
| --- | --- |
| Sampling Method | Simple |
| Number of Samples | 2000 |
| Confidence Interval Level | 95.0% |
| Confidence Interval Type | Percentile |

**General Linear Model**

| **Notes** | | |
| --- | --- | --- |
| Output Created | | 24-FEB-2026 09:02:26 |
| Comments | |  |
| Input | Data | E:\Malaysia - Feb 24\Malaysia - Descriptive and multivariate regression.sav |
|  | Active Dataset | DataSet2 |
|  | Filter | <none> |
|  | Weight | <none> |
|  | Split File | <none> |
|  | N of Rows in Working Data File | 16008 |
| Missing Value Handling | Definition of Missing | User-defined missing values are treated as missing. |
|  | Cases Used | Statistics are based on all cases with valid data for all variables in the model. |
| Syntax | | GLM Fatal_rate Nonfatal_rate WITH Inspector_ratio /METHOD=SSTYPE(3) /INTERCEPT=INCLUDE /SAVE=PRED SEPRED RESID ZRESID COOK LEVER /EMMEANS=TABLES(OVERALL) WITH(Inspector_ratio=MEAN) /PRINT=DESCRIPTIVE ETASQ OPOWER PARAMETER TEST(SSCP) RSSCP HOMOGENEITY LOF /PLOT=SPREADLEVEL RESIDUALS /CRITERIA=ALPHA(.05) /DESIGN=Inspector_ratio. |
| Resources | Processor Time | 00:00:26.11 |
|  | Elapsed Time | 00:00:25.47 |

| **Warnings** |
| --- |
| The HOMOGENEITY specification in the PRINT subcommand will be ignored because there are no between-subjects factors. |
| The SPREADLEVEL specification has been found in the PLOT subcommand, but the model includes no factors. The spread versus level plot will not be produced. |
| This command is trying to save new variables while bootstrapping is in effect. The new variables will be lost. Execution of this command stops. |

| **Descriptive Statistics** | | | |
| --- | --- | --- | --- |
|  | Mean | Std. Deviation | N |
| Fatal_rate | 3.242500 | 1.2947780 | 8 |
| Nonfatal_rate | 236.632500 | 44.6079512 | 8 |

| **Bartlett's Test of Sphericity**^a^ | |
| --- | --- |
| Likelihood Ratio | .000 |
| Approx. Chi-Square | 38.100 |
| df | 2 |
| Sig. | <.001 |
| Tests the null hypothesis that the residual covariance matrix is proportional to an identity matrix.^a^  a. Design: Intercept + Inspector_ratio | |

| **Multivariate Tests**^a^ | | | | | | |
| --- | --- | --- | --- | --- | --- | --- |
| Effect | | Value | F | Hypothesis df | Error df | Sig. |
| Intercept | Pillai's Trace | .728 | 6.705^b^ | 2.000 | 5.000 | .038 |
|  | Wilks' Lambda | .272 | 6.705^b^ | 2.000 | 5.000 | .038 |
|  | Hotelling's Trace | 2.682 | 6.705^b^ | 2.000 | 5.000 | .038 |
|  | Roy's Largest Root | 2.682 | 6.705^b^ | 2.000 | 5.000 | .038 |
| Inspector_ratio | Pillai's Trace | .694 | 5.677^b^ | 2.000 | 5.000 | .052 |
|  | Wilks' Lambda | .306 | 5.677^b^ | 2.000 | 5.000 | .052 |
|  | Hotelling's Trace | 2.271 | 5.677^b^ | 2.000 | 5.000 | .052 |
|  | Roy's Largest Root | 2.271 | 5.677^b^ | 2.000 | 5.000 | .052 |

| **Multivariate Tests**^a^ | | | | |
| --- | --- | --- | --- | --- |
| Effect | | Partial Eta Squared | Noncent. Parameter | Observed Power^c^ |
| Intercept | Pillai's Trace | .728 | 13.410 | .660 |
|  | Wilks' Lambda | .728 | 13.410 | .660 |
|  | Hotelling's Trace | .728 | 13.410 | .660 |
|  | Roy's Largest Root | .728 | 13.410 | .660 |
| Inspector_ratio | Pillai's Trace | .694 | 11.354 | .587 |
|  | Wilks' Lambda | .694 | 11.354 | .587 |
|  | Hotelling's Trace | .694 | 11.354 | .587 |
|  | Roy's Largest Root | .694 | 11.354 | .587 |
| a. Design: Intercept + Inspector_ratio | | | | |
| b. Exact statistic | | | | |
| c. Computed using alpha = .05 | | | | |

| **Tests of Between-Subjects Effects** | | | | | | |
| --- | --- | --- | --- | --- | --- | --- |
| Source | Dependent Variable | Type III Sum of Squares | df | Mean Square | F | Sig. |
| Corrected Model | Fatal_rate | 3.427^a^ | 1 | 3.427 | 2.475 | .167 |
|  | Nonfatal_rate | 28.531^b^ | 1 | 28.531 | .012 | .915 |
| Intercept | Fatal_rate | 2.521 | 1 | 2.521 | 1.820 | .226 |
|  | Nonfatal_rate | 601.542 | 1 | 601.542 | .260 | .629 |
| Inspector_ratio | Fatal_rate | 3.427 | 1 | 3.427 | 2.475 | .167 |
|  | Nonfatal_rate | 28.531 | 1 | 28.531 | .012 | .915 |
| Error | Fatal_rate | 8.308 | 6 | 1.385 |  |  |
|  | Nonfatal_rate | 13900.554 | 6 | 2316.759 |  |  |
| Total | Fatal_rate | 95.846 | 8 |  |  |  |
|  | Nonfatal_rate | 461888.606 | 8 |  |  |  |
| Corrected Total | Fatal_rate | 11.735 | 7 |  |  |  |
|  | Nonfatal_rate | 13929.085 | 7 |  |  |  |

| **Tests of Between-Subjects Effects** | | | | |
| --- | --- | --- | --- | --- |
| Source | Dependent Variable | Partial Eta Squared | Noncent. Parameter | Observed Power^c^ |
| Corrected Model | Fatal_rate | .292 | 2.475 | .264 |
|  | Nonfatal_rate | .002 | .012 | .051 |
| Intercept | Fatal_rate | .233 | 1.820 | .207 |
|  | Nonfatal_rate | .041 | .260 | .072 |
| Inspector_ratio | Fatal_rate | .292 | 2.475 | .264 |
|  | Nonfatal_rate | .002 | .012 | .051 |
| Error | Fatal_rate |  |  |  |
|  | Nonfatal_rate |  |  |  |
| Total | Fatal_rate |  |  |  |
|  | Nonfatal_rate |  |  |  |
| Corrected Total | Fatal_rate |  |  |  |
|  | Nonfatal_rate |  |  |  |
| a. R Squared = .292 (Adjusted R Squared = .174) | | | | |
| b. R Squared = .002 (Adjusted R Squared = -.164) | | | | |
| c. Computed using alpha = .05 | | | | |

| **Parameter Estimates** | | | | | | |
| --- | --- | --- | --- | --- | --- | --- |
| Dependent Variable | Parameter | B | Std. Error | t | Sig. | 95% Confidence Interval |
|  |  |  |  |  |  | Lower Bound |
| Fatal_rate | Intercept | -19.580 | 14.512 | -1.349 | .226 | -55.090 |
|  | Inspector_ratio | 30.378 | 19.308 | 1.573 | .167 | -16.867 |
| Nonfatal_rate | Intercept | 302.481 | 593.616 | .510 | .629 | -1150.045 |
|  | Inspector_ratio | -87.646 | 789.794 | -.111 | .915 | -2020.201 |

| **Parameter Estimates** | | | | | |
| --- | --- | --- | --- | --- | --- |
| Dependent Variable | Parameter | 95% Confidence Interval | Partial Eta Squared | Noncent. Parameter | Observed Power^a^ |
|  |  | Upper Bound |  |  |  |
| Fatal_rate | Intercept | 15.930 | .233 | 1.349 | .207 |
|  | Inspector_ratio | 77.623 | .292 | 1.573 | .264 |
| Nonfatal_rate | Intercept | 1755.007 | .041 | .510 | .072 |
|  | Inspector_ratio | 1844.909 | .002 | .111 | .051 |
| a. Computed using alpha = .05 | | | | | |

| **Bootstrap for Parameter Estimates** | | | | | | |
| --- | --- | --- | --- | --- | --- | --- |
| Dependent Variable | Parameter | B | Bootstrap^a^ | | | |
|  |  |  | Bias | Std. Error | Sig. (2-tailed) | 95% Confidence Interval |
|  |  |  |  |  |  | Lower |
| Fatal_rate | Intercept | -19.580 | .094 | 16.562 | .163 | -47.057 |
|  | Inspector_ratio | 30.378 | -.116 | 22.294 | .118 | -12.853 |
| Nonfatal_rate | Intercept | 302.481 | 78.181 | 717.299 | .669 | -589.153 |
|  | Inspector_ratio | -87.646 | -105.619 | 969.380 | .920 | -2508.278 |

| **Bootstrap for Parameter Estimates** | | |
| --- | --- | --- |
| Dependent Variable | Parameter | Bootstrap |
|  |  | 95% Confidence Interval |
|  |  | Upper |
| Fatal_rate | Intercept | 12.600 |
|  | Inspector_ratio | 67.650 |
| Nonfatal_rate | Intercept | 2105.711 |
|  | Inspector_ratio | 1109.680 |
| a. Unless otherwise noted, bootstrap results are based on 2000 bootstrap samples | | |

| **Between-Subjects SSCP Matrix** | | | | |
| --- | --- | --- | --- | --- |
|  | | | Fatal_rate | Nonfatal_rate |
| Hypothesis | Intercept | Fatal_rate | 2.521 | -38.939 |
|  |  | Nonfatal_rate | -38.939 | 601.542 |
|  | Inspector_ratio | Fatal_rate | 3.427 | -9.889 |
|  |  | Nonfatal_rate | -9.889 | 28.531 |
| Error | | Fatal_rate | 8.308 | 302.916 |
|  |  | Nonfatal_rate | 302.916 | 13900.554 |
| Based on Type III Sum of Squares | | | | |

| **Residual SSCP Matrix** | | | |
| --- | --- | --- | --- |
|  | | Fatal_rate | Nonfatal_rate |
| Sum-of-Squares and Cross-Products | Fatal_rate | 8.308 | 302.916 |
|  | Nonfatal_rate | 302.916 | 13900.554 |
| Covariance | Fatal_rate | 1.385 | 50.486 |
|  | Nonfatal_rate | 50.486 | 2316.759 |
| Correlation | Fatal_rate | 1.000 | .891 |
|  | Nonfatal_rate | .891 | 1.000 |
| Based on Type III Sum of Squares | | | |

**Lack of Fit**

| **Multivariate Tests** | | | | | |
| --- | --- | --- | --- | --- | --- |
| Dependent Variables | | Value | F | Hypothesis df | Error df |
| Fatal_rate, Nonfatal_rate | Pillai's Trace | .^a^ | . | . | . |
|  | Wilks' Lambda | .^a^ | . | . | . |
|  | Hotelling's Trace | .^a^ | . | . | . |
|  | Roy's Largest Root | .^a^ | . | . | . |
| Fatal_rate | Pillai's Trace | .^a^ | . | . | . |
|  | Wilks' Lambda | .^a^ | . | . | . |
|  | Hotelling's Trace | .^a^ | . | . | . |
|  | Roy's Largest Root | .^a^ | . | . | . |
| Nonfatal_rate | Pillai's Trace | .^a^ | . | . | . |
|  | Wilks' Lambda | .^a^ | . | . | . |
|  | Hotelling's Trace | .^a^ | . | . | . |
|  | Roy's Largest Root | .^a^ | . | . | . |

| **Multivariate Tests** | | | | |
| --- | --- | --- | --- | --- |
| Dependent Variables | | Sig. | Partial Eta Squared | Noncent. Parameter |
| Fatal_rate, Nonfatal_rate | Pillai's Trace | . | . | . |
|  | Wilks' Lambda | . | . | . |
|  | Hotelling's Trace | . | . | . |
|  | Roy's Largest Root | . | . | . |
| Fatal_rate | Pillai's Trace | . | . | . |
|  | Wilks' Lambda | . | . | . |
|  | Hotelling's Trace | . | . | . |
|  | Roy's Largest Root | . | . | . |
| Nonfatal_rate | Pillai's Trace | . | . | . |
|  | Wilks' Lambda | . | . | . |
|  | Hotelling's Trace | . | . | . |
|  | Roy's Largest Root | . | . | . |

| **Multivariate Tests** | | |
| --- | --- | --- |
| Dependent Variables | | Observed Power^b^ |
| Fatal_rate, Nonfatal_rate | Pillai's Trace | . |
|  | Wilks' Lambda | . |
|  | Hotelling's Trace | . |
|  | Roy's Largest Root | . |
| Fatal_rate | Pillai's Trace | . |
|  | Wilks' Lambda | . |
|  | Hotelling's Trace | . |
|  | Roy's Largest Root | . |
| Nonfatal_rate | Pillai's Trace | . |
|  | Wilks' Lambda | . |
|  | Hotelling's Trace | . |
|  | Roy's Largest Root | . |
| a. The pure error sub-matrix corresponding to the selected dependent variables is a zero matrix. | | |
| b. Computed using alpha = .05 | | |

| **Univariate Tests** | | | | | | |
| --- | --- | --- | --- | --- | --- | --- |
| Dependent Variable | Source | Sum of Squares | df | Mean Square | F | Sig. |
| Fatal_rate | Lack of Fit | 8.308 | 6 | 1.385 | . | . |
|  | Pure Error | .000 | 0 | . |  |  |
| Nonfatal_rate | Lack of Fit | 13900.554 | 6 | 2316.759 | . | . |
|  | Pure Error | .000 | 0 | . |  |  |

| **Univariate Tests** | | | | |
| --- | --- | --- | --- | --- |
| Dependent Variable | Source | Partial Eta Squared | Noncent. Parameter | Observed Power^a^ |
| Fatal_rate | Lack of Fit | 1.000 | . | . |
|  | Pure Error |  |  |  |
| Nonfatal_rate | Lack of Fit | 1.000 | . | . |
|  | Pure Error |  |  |  |
| a. Computed using alpha = .05 | | | | |

| **SSCP Matrix** | | | |
| --- | --- | --- | --- |
|  | | Fatal_rate | Nonfatal_rate |
| Lack of Fit | Fatal_rate | 8.308 | 302.916 |
|  | Nonfatal_rate | 302.916 | 13900.554 |
| Pure Error | Fatal_rate | .000 | .000 |
|  | Nonfatal_rate | .000 | .000 |

**Estimated Marginal Means**

| **Grand Mean** | | | | |
| --- | --- | --- | --- | --- |
| Dependent Variable | Mean | Std. Error | 95% Confidence Interval | |
|  |  |  | Lower Bound | Upper Bound |
| Fatal_rate | 3.242^a^ | .416 | 2.225 | 4.260 |
| Nonfatal_rate | 236.633^a^ | 17.017 | 194.992 | 278.273 |
| a. Covariates appearing in the model are evaluated at the following values: Inspector_ratio = .751300. | | | | |

**Descriptive statistics with log-transformation of dependent variables (PP. 25-33)**

**Explore**

| **Notes** | | |
| --- | --- | --- |
| Output Created | | 24-FEB-2026 09:05:42 |
| Comments | |  |
| Input | Data | E:\Malaysia - Feb 24\Malaysia - Descriptive and multivariate regression.sav |
|  | Active Dataset | DataSet2 |
|  | Filter | <none> |
|  | Weight | <none> |
|  | Split File | <none> |
|  | N of Rows in Working Data File | 8 |
| Missing Value Handling | Definition of Missing | User-defined missing values for dependent variables are treated as missing. |
|  | Cases Used | Statistics are based on cases with no missing values for any dependent variable or factor used. |
| Syntax | | EXAMINE VARIABLES=Inspector_ratio Log_fatalrate Log_nonfatalrate /PLOT BOXPLOT HISTOGRAM NPPLOT /COMPARE GROUPS /STATISTICS DESCRIPTIVES EXTREME /CINTERVAL 95 /MISSING LISTWISE /NOTOTAL. |
| Resources | Processor Time | 00:00:01.66 |
|  | Elapsed Time | 00:00:01.31 |

| **Case Processing Summary** | | | | | | |
| --- | --- | --- | --- | --- | --- | --- |
|  | Cases | | | | | |
|  | Valid | | Missing | | Total | |
|  | N | Percent | N | Percent | N | Percent |
| Inspector_ratio | 8 | 100.0% | 0 | 0.0% | 8 | 100.0% |
| Log_fatalrate | 8 | 100.0% | 0 | 0.0% | 8 | 100.0% |
| Log_nonfatalrate | 8 | 100.0% | 0 | 0.0% | 8 | 100.0% |

| **Descriptives** | | | | |
| --- | --- | --- | --- | --- |
|  | | | Statistic | Std. Error |
| Inspector_ratio | Mean | | .751300 | .0081439 |
|  | 95% Confidence Interval for Mean | Lower Bound | .732043 |  |
|  |  | Upper Bound | .770557 |  |
|  | 5% Trimmed Mean | | .751217 |  |
|  | Median | | .748150 |  |
|  | Variance | | .001 |  |
|  | Std. Deviation | | .0230344 |  |
|  | Minimum | | .7147 |  |
|  | Maximum | | .7894 |  |
|  | Range | | .0747 |  |
|  | Interquartile Range | | .0312 |  |
|  | Skewness | | .165 | .752 |
|  | Kurtosis | | .144 | 1.481 |
| Log_fatalrate | Mean | | .4795 | .06276 |
|  | 95% Confidence Interval for Mean | Lower Bound | .3311 |  |
|  |  | Upper Bound | .6279 |  |
|  | 5% Trimmed Mean | | .4776 |  |
|  | Median | | .4548 |  |
|  | Variance | | .032 |  |
|  | Std. Deviation | | .17751 |  |
|  | Minimum | | .31 |  |
|  | Maximum | | .68 |  |
|  | Range | | .38 |  |
|  | Interquartile Range | | .35 |  |
|  | Skewness | | .123 | .752 |
|  | Kurtosis | | -2.520 | 1.481 |
| Log_nonfatalrate | Mean | | 2.3659 | .03340 |
|  | 95% Confidence Interval for Mean | Lower Bound | 2.2869 |  |
|  |  | Upper Bound | 2.4449 |  |
|  | 5% Trimmed Mean | | 2.3729 |  |
|  | Median | | 2.3800 |  |
|  | Variance | | .009 |  |
|  | Std. Deviation | | .09448 |  |
|  | Minimum | | 2.16 |  |
|  | Maximum | | 2.45 |  |
|  | Range | | .30 |  |
|  | Interquartile Range | | .09 |  |
|  | Skewness | | -1.823 | .752 |
|  | Kurtosis | | 4.015 | 1.481 |

| **Extreme Values**^a^ | | | | |
| --- | --- | --- | --- | --- |
|  | | | Case Number | Value |
| Inspector_ratio | Highest | 1 | 1 | .7894 |
|  |  | 2 | 6 | .7715 |
|  |  | 3 | 2 | .7615 |
|  |  | 4 | 3 | .7536 |
|  | Lowest | 1 | 8 | .7147 |
|  |  | 2 | 4 | .7371 |
|  |  | 3 | 7 | .7399 |
|  |  | 4 | 5 | .7427 |
| Log_fatalrate | Highest | 1 | 1 | .68 |
|  |  | 2 | 2 | .68 |
|  |  | 3 | 3 | .62 |
|  |  | 4 | 4 | .58 |
|  | Lowest | 1 | 6 | .31 |
|  |  | 2 | 8 | .31 |
|  |  | 3 | 7 | .32 |
|  |  | 4 | 5 | .33 |
| Log_nonfatalrate | Highest | 1 | 2 | 2.45 |
|  |  | 2 | 1 | 2.44 |
|  |  | 3 | 4 | 2.43 |
|  |  | 4 | 8 | 2.39 |
|  | Lowest | 1 | 6 | 2.16 |
|  |  | 2 | 5 | 2.34 |
|  |  | 3 | 7 | 2.35 |
|  |  | 4 | 3 | 2.37 |
| a. The requested number of extreme values exceeds the number of data points. A smaller number of extremes is displayed. | | | | |

| **Tests of Normality** | | | | | | |
| --- | --- | --- | --- | --- | --- | --- |
|  | Kolmogorov-Smirnov^a^ | | | Shapiro-Wilk | | |
|  | Statistic | df | Sig. | Statistic | df | Sig. |
| Inspector_ratio | .146 | 8 | .200^*^ | .985 | 8 | .983 |
| Log_fatalrate | .306 | 8 | .027 | .773 | 8 | .014 |
| Log_nonfatalrate | .276 | 8 | .074 | .811 | 8 | .038 |
| *. This is a lower bound of the true significance. | | | | | | |
| a. Lilliefors Significance Correction | | | | | | |

**Inspector_ratio**

 **Log_fatalrate**

**Log_nonfatalrate**

**Log-general linear multivariate regression with bootstrap-2000 with Confidence Intervals of 95% (PP. 34-40)**

**Bootstrap**

| **Notes** | | |
| --- | --- | --- |
| Output Created | | 24-FEB-2026 09:06:59 |
| Comments | |  |
| Input | Data | E:\Malaysia - Feb 24\Malaysia - Descriptive and multivariate regression.sav |
|  | Active Dataset | DataSet2 |
|  | Filter | <none> |
|  | Weight | <none> |
|  | Split File | <none> |
|  | N of Rows in Working Data File | 8 |
| Syntax | | BOOTSTRAP /SAMPLING METHOD=SIMPLE /VARIABLES TARGET=Log_fatalrate Log_nonfatalrate INPUT= Inspector_ratio /CRITERIA CILEVEL=95 CITYPE=PERCENTILE NSAMPLES=2000 /MISSING USERMISSING=EXCLUDE. |
| Resources | Processor Time | 00:00:00.02 |
|  | Elapsed Time | 00:00:00.02 |

| **Bootstrap Specifications** | |
| --- | --- |
| Sampling Method | Simple |
| Number of Samples | 2000 |
| Confidence Interval Level | 95.0% |
| Confidence Interval Type | Percentile |

**General Linear Model**

| **Notes** | | |
| --- | --- | --- |
| Output Created | | 24-FEB-2026 09:06:59 |
| Comments | |  |
| Input | Data | E:\Malaysia - Feb 24\Malaysia - Descriptive and multivariate regression.sav |
|  | Active Dataset | DataSet2 |
|  | Filter | <none> |
|  | Weight | <none> |
|  | Split File | <none> |
|  | N of Rows in Working Data File | 16008 |
| Missing Value Handling | Definition of Missing | User-defined missing values are treated as missing. |
|  | Cases Used | Statistics are based on all cases with valid data for all variables in the model. |
| Syntax | | GLM Log_fatalrate Log_nonfatalrate WITH Inspector_ratio /METHOD=SSTYPE(3) /INTERCEPT=INCLUDE /SAVE=PRED SEPRED RESID ZRESID COOK LEVER /EMMEANS=TABLES(OVERALL) WITH(Inspector_ratio=MEAN) /PRINT=DESCRIPTIVE ETASQ OPOWER PARAMETER TEST(SSCP) RSSCP HOMOGENEITY LOF /PLOT=SPREADLEVEL RESIDUALS /CRITERIA=ALPHA(.05) /DESIGN=Inspector_ratio. |
| Resources | Processor Time | 00:00:24.89 |
|  | Elapsed Time | 00:00:24.99 |

| **Warnings** |
| --- |
| The HOMOGENEITY specification in the PRINT subcommand will be ignored because there are no between-subjects factors. |
| The SPREADLEVEL specification has been found in the PLOT subcommand, but the model includes no factors. The spread versus level plot will not be produced. |
| This command is trying to save new variables while bootstrapping is in effect. The new variables will be lost. Execution of this command stops. |

| **Descriptive Statistics** | | | |
| --- | --- | --- | --- |
|  | Mean | Std. Deviation | N |
| Log_fatalrate | .4795 | .17751 | 8 |
| Log_nonfatalrate | 2.3659 | .09448 | 8 |

| **Bartlett's Test of Sphericity**^a^ | |
| --- | --- |
| Likelihood Ratio | .002 |
| Approx. Chi-Square | 7.785 |
| df | 2 |
| Sig. | .020 |
| Tests the null hypothesis that the residual covariance matrix is proportional to an identity matrix.^a^  a. Design: Intercept + Inspector_ratio | |

| **Multivariate Tests**^a^ | | | | | | |
| --- | --- | --- | --- | --- | --- | --- |
| Effect | | Value | F | Hypothesis df | Error df | Sig. |
| Intercept | Pillai's Trace | .871 | 16.867^b^ | 2.000 | 5.000 | .006 |
|  | Wilks' Lambda | .129 | 16.867^b^ | 2.000 | 5.000 | .006 |
|  | Hotelling's Trace | 6.747 | 16.867^b^ | 2.000 | 5.000 | .006 |
|  | Roy's Largest Root | 6.747 | 16.867^b^ | 2.000 | 5.000 | .006 |
| Inspector_ratio | Pillai's Trace | .644 | 4.531^b^ | 2.000 | 5.000 | .075 |
|  | Wilks' Lambda | .356 | 4.531^b^ | 2.000 | 5.000 | .075 |
|  | Hotelling's Trace | 1.813 | 4.531^b^ | 2.000 | 5.000 | .075 |
|  | Roy's Largest Root | 1.813 | 4.531^b^ | 2.000 | 5.000 | .075 |

| **Multivariate Tests**^a^ | | | | |
| --- | --- | --- | --- | --- |
| Effect | | Partial Eta Squared | Noncent. Parameter | Observed Power^c^ |
| Intercept | Pillai's Trace | .871 | 33.735 | .964 |
|  | Wilks' Lambda | .871 | 33.735 | .964 |
|  | Hotelling's Trace | .871 | 33.735 | .964 |
|  | Roy's Largest Root | .871 | 33.735 | .964 |
| Inspector_ratio | Pillai's Trace | .644 | 9.063 | .493 |
|  | Wilks' Lambda | .644 | 9.063 | .493 |
|  | Hotelling's Trace | .644 | 9.063 | .493 |
|  | Roy's Largest Root | .644 | 9.063 | .493 |
| a. Design: Intercept + Inspector_ratio | | | | |
| b. Exact statistic | | | | |
| c. Computed using alpha = .05 | | | | |

| **Tests of Between-Subjects Effects** | | | | | | |
| --- | --- | --- | --- | --- | --- | --- |
| Source | Dependent Variable | Type III Sum of Squares | df | Mean Square | F | Sig. |
| Corrected Model | Log_fatalrate | .057^a^ | 1 | .057 | 2.095 | .198 |
|  | Log_nonfatalrate | .001^b^ | 1 | .001 | .081 | .786 |
| Intercept | Log_fatalrate | .040 | 1 | .040 | 1.467 | .271 |
|  | Log_nonfatalrate | .049 | 1 | .049 | 4.739 | .072 |
| Inspector_ratio | Log_fatalrate | .057 | 1 | .057 | 2.095 | .198 |
|  | Log_nonfatalrate | .001 | 1 | .001 | .081 | .786 |
| Error | Log_fatalrate | .163 | 6 | .027 |  |  |
|  | Log_nonfatalrate | .062 | 6 | .010 |  |  |
| Total | Log_fatalrate | 2.060 | 8 |  |  |  |
|  | Log_nonfatalrate | 44.843 | 8 |  |  |  |
| Corrected Total | Log_fatalrate | .221 | 7 |  |  |  |
|  | Log_nonfatalrate | .062 | 7 |  |  |  |

| **Tests of Between-Subjects Effects** | | | | |
| --- | --- | --- | --- | --- |
| Source | Dependent Variable | Partial Eta Squared | Noncent. Parameter | Observed Power^c^ |
| Corrected Model | Log_fatalrate | .259 | 2.095 | .231 |
|  | Log_nonfatalrate | .013 | .081 | .057 |
| Intercept | Log_fatalrate | .196 | 1.467 | .177 |
|  | Log_nonfatalrate | .441 | 4.739 | .448 |
| Inspector_ratio | Log_fatalrate | .259 | 2.095 | .231 |
|  | Log_nonfatalrate | .013 | .081 | .057 |
| Error | Log_fatalrate |  |  |  |
|  | Log_nonfatalrate |  |  |  |
| Total | Log_fatalrate |  |  |  |
|  | Log_nonfatalrate |  |  |  |
| Corrected Total | Log_fatalrate |  |  |  |
|  | Log_nonfatalrate |  |  |  |
| a. R Squared = .259 (Adjusted R Squared = .135) | | | | |
| b. R Squared = .013 (Adjusted R Squared = -.151) | | | | |
| c. Computed using alpha = .05 | | | | |

| **Parameter Estimates** | | | | | | |
| --- | --- | --- | --- | --- | --- | --- |
| Dependent Variable | Parameter | B | Std. Error | t | Sig. | 95% Confidence Interval |
|  |  |  |  |  |  | Lower Bound |
| Log_fatalrate | Intercept | -2.466 | 2.036 | -1.211 | .271 | -7.447 |
|  | Inspector_ratio | 3.920 | 2.709 | 1.447 | .198 | -2.707 |
| Log_nonfatalrate | Intercept | 2.721 | 1.250 | 2.177 | .072 | -.338 |
|  | Inspector_ratio | -.473 | 1.663 | -.284 | .786 | -4.543 |

| **Parameter Estimates** | | | | | |
| --- | --- | --- | --- | --- | --- |
| Dependent Variable | Parameter | 95% Confidence Interval | Partial Eta Squared | Noncent. Parameter | Observed Power^a^ |
|  |  | Upper Bound |  |  |  |
| Log_fatalrate | Intercept | 2.515 | .196 | 1.211 | .177 |
|  | Inspector_ratio | 10.548 | .259 | 1.447 | .231 |
| Log_nonfatalrate | Intercept | 5.780 | .441 | 2.177 | .448 |
|  | Inspector_ratio | 3.597 | .013 | .284 | .057 |
| a. Computed using alpha = .05 | | | | | |

| **Bootstrap for Parameter Estimates** | | | | | | |
| --- | --- | --- | --- | --- | --- | --- |
| Dependent Variable | Parameter | B | Bootstrap^a^ | | | |
|  |  |  | Bias | Std. Error | Sig. (2-tailed) | 95% Confidence Interval |
|  |  |  |  |  |  | Lower |
| Log_fatalrate | Intercept | -2.466 | .100 | 2.148 | .213 | -5.942 |
|  | Inspector_ratio | 3.920 | -.128 | 2.881 | .146 | -3.160 |
| Log_nonfatalrate | Intercept | 2.721 | .213 | 1.569 | .252 | .959 |
|  | Inspector_ratio | -.473 | -.285 | 2.118 | .808 | -6.308 |

| **Bootstrap for Parameter Estimates** | | |
| --- | --- | --- |
| Dependent Variable | Parameter | Bootstrap |
|  |  | 95% Confidence Interval |
|  |  | Upper |
| Log_fatalrate | Intercept | 2.780 |
|  | Inspector_ratio | 8.577 |
| Log_nonfatalrate | Intercept | 7.089 |
|  | Inspector_ratio | 1.890 |
| a. Unless otherwise noted, bootstrap results are based on 2000 bootstrap samples | | |

| **Between-Subjects SSCP Matrix** | | | | |
| --- | --- | --- | --- | --- |
|  | | | Log_fatalrate | Log_nonfatalrate |
| Hypothesis | Intercept | Log_fatalrate | .040 | -.044 |
|  |  | Log_nonfatalrate | -.044 | .049 |
|  | Inspector_ratio | Log_fatalrate | .057 | -.007 |
|  |  | Log_nonfatalrate | -.007 | .001 |
| Error | | Log_fatalrate | .163 | .086 |
|  |  | Log_nonfatalrate | .086 | .062 |
| Based on Type III Sum of Squares | | | | |

| **Residual SSCP Matrix** | | | |
| --- | --- | --- | --- |
|  | | Log_fatalrate | Log_nonfatalrate |
| Sum-of-Squares and Cross-Products | Log_fatalrate | .163 | .086 |
|  | Log_nonfatalrate | .086 | .062 |
| Covariance | Log_fatalrate | .027 | .014 |
|  | Log_nonfatalrate | .014 | .010 |
| Correlation | Log_fatalrate | 1.000 | .857 |
|  | Log_nonfatalrate | .857 | 1.000 |
| Based on Type III Sum of Squares | | | |

**Lack of Fit**

| **Multivariate Tests** | | | | | |
| --- | --- | --- | --- | --- | --- |
| Dependent Variables | | Value | F | Hypothesis df | Error df |
| Log_fatalrate, Log_nonfatalrate | Pillai's Trace | .^a^ | . | . | . |
|  | Wilks' Lambda | .^a^ | . | . | . |
|  | Hotelling's Trace | .^a^ | . | . | . |
|  | Roy's Largest Root | .^a^ | . | . | . |
| Log_fatalrate | Pillai's Trace | .^a^ | . | . | . |
|  | Wilks' Lambda | .^a^ | . | . | . |
|  | Hotelling's Trace | .^a^ | . | . | . |
|  | Roy's Largest Root | .^a^ | . | . | . |
| Log_nonfatalrate | Pillai's Trace | .^a^ | . | . | . |
|  | Wilks' Lambda | .^a^ | . | . | . |
|  | Hotelling's Trace | .^a^ | . | . | . |
|  | Roy's Largest Root | .^a^ | . | . | . |

| **Multivariate Tests** | | | | |
| --- | --- | --- | --- | --- |
| Dependent Variables | | Sig. | Partial Eta Squared | Noncent. Parameter |
| Log_fatalrate, Log_nonfatalrate | Pillai's Trace | . | . | . |
|  | Wilks' Lambda | . | . | . |
|  | Hotelling's Trace | . | . | . |
|  | Roy's Largest Root | . | . | . |
| Log_fatalrate | Pillai's Trace | . | . | . |
|  | Wilks' Lambda | . | . | . |
|  | Hotelling's Trace | . | . | . |
|  | Roy's Largest Root | . | . | . |
| Log_nonfatalrate | Pillai's Trace | . | . | . |
|  | Wilks' Lambda | . | . | . |
|  | Hotelling's Trace | . | . | . |
|  | Roy's Largest Root | . | . | . |

| **Multivariate Tests** | | |
| --- | --- | --- |
| Dependent Variables | | Observed Power^b^ |
| Log_fatalrate, Log_nonfatalrate | Pillai's Trace | . |
|  | Wilks' Lambda | . |
|  | Hotelling's Trace | . |
|  | Roy's Largest Root | . |
| Log_fatalrate | Pillai's Trace | . |
|  | Wilks' Lambda | . |
|  | Hotelling's Trace | . |
|  | Roy's Largest Root | . |
| Log_nonfatalrate | Pillai's Trace | . |
|  | Wilks' Lambda | . |
|  | Hotelling's Trace | . |
|  | Roy's Largest Root | . |
| a. The pure error sub-matrix corresponding to the selected dependent variables is a zero matrix. | | |
| b. Computed using alpha = .05 | | |

| **Univariate Tests** | | | | | | |
| --- | --- | --- | --- | --- | --- | --- |
| Dependent Variable | Source | Sum of Squares | df | Mean Square | F | Sig. |
| Log_fatalrate | Lack of Fit | .163 | 6 | .027 | . | . |
|  | Pure Error | .000 | 0 | . |  |  |
| Log_nonfatalrate | Lack of Fit | .062 | 6 | .010 | . | . |
|  | Pure Error | .000 | 0 | . |  |  |

| **Univariate Tests** | | | | |
| --- | --- | --- | --- | --- |
| Dependent Variable | Source | Partial Eta Squared | Noncent. Parameter | Observed Power^a^ |
| Log_fatalrate | Lack of Fit | 1.000 | . | . |
|  | Pure Error |  |  |  |
| Log_nonfatalrate | Lack of Fit | 1.000 | . | . |
|  | Pure Error |  |  |  |
| a. Computed using alpha = .05 | | | | |

| **SSCP Matrix** | | | |
| --- | --- | --- | --- |
|  | | Log_fatalrate | Log_nonfatalrate |
| Lack of Fit | Log_fatalrate | .163 | .086 |
|  | Log_nonfatalrate | .086 | .062 |
| Pure Error | Log_fatalrate | .000 | .000 |
|  | Log_nonfatalrate | .000 | .000 |

**Estimated Marginal Means**

| **Grand Mean** | | | | |
| --- | --- | --- | --- | --- |
| Dependent Variable | Mean | Std. Error | 95% Confidence Interval | |
|  |  |  | Lower Bound | Upper Bound |
| Log_fatalrate | .479^a^ | .058 | .337 | .622 |
| Log_nonfatalrate | 2.366^a^ | .036 | 2.278 | 2.454 |
| a. Covariates appearing in the model are evaluated at the following values: Inspector_ratio = .751300. | | | | |

**Log-general linear multivariate regression analysis (PP. 41-47)**

| **Notes** | | |
| --- | --- | --- |
| Output Created | | 24-FEB-2026 09:08:23 |
| Comments | |  |
| Input | Data | E:\Malaysia - Feb 24\Malaysia - Descriptive and multivariate regression.sav |
|  | Active Dataset | DataSet2 |
|  | Filter | <none> |
|  | Weight | <none> |
|  | Split File | <none> |
|  | N of Rows in Working Data File | 8 |
| Missing Value Handling | Definition of Missing | User-defined missing values are treated as missing. |
|  | Cases Used | Statistics are based on all cases with valid data for all variables in the model. |
| Syntax | | GLM Log_fatalrate Log_nonfatalrate WITH Inspector_ratio /METHOD=SSTYPE(3) /INTERCEPT=INCLUDE /SAVE=PRED SEPRED RESID ZRESID COOK LEVER /EMMEANS=TABLES(OVERALL) WITH(Inspector_ratio=MEAN) /PRINT=DESCRIPTIVE ETASQ OPOWER PARAMETER TEST(SSCP) RSSCP HOMOGENEITY LOF /PLOT=SPREADLEVEL RESIDUALS /CRITERIA=ALPHA(.05) /DESIGN=Inspector_ratio. |
| Resources | Processor Time | 00:00:00.27 |
|  | Elapsed Time | 00:00:00.26 |
| Variables Created or Modified | PRE_3 | Predicted Value for Log_fatalrate |
|  | PRE_4 | Predicted Value for Log_nonfatalrate |
|  | SEP_3 | Standard Error of Predicted Value for Log_fatalrate |
|  | SEP_4 | Standard Error of Predicted Value for Log_nonfatalrate |
|  | RES_3 | Residual for Log_fatalrate |
|  | RES_4 | Residual for Log_nonfatalrate |
|  | ZRE_3 | Standardized Residual for Log_fatalrate |
|  | ZRE_4 | Standardized Residual for Log_nonfatalrate |
|  | COO_3 | Cook's Distance for Log_fatalrate |
|  | COO_4 | Cook's Distance for Log_nonfatalrate |
|  | LEV_3 | Uncentered Leverage Value for Log_fatalrate |
|  | LEV_4 | Uncentered Leverage Value for Log_nonfatalrate |

| **Warnings** |
| --- |
| The HOMOGENEITY specification in the PRINT subcommand will be ignored because there are no between-subjects factors. |
| The SPREADLEVEL specification has been found in the PLOT subcommand, but the model includes no factors. The spread versus level plot will not be produced. |

| **Descriptive Statistics** | | | |
| --- | --- | --- | --- |
|  | Mean | Std. Deviation | N |
| Log_fatalrate | .4795 | .17751 | 8 |
| Log_nonfatalrate | 2.3659 | .09448 | 8 |

| **Bartlett's Test of Sphericity**^a^ | |
| --- | --- |
| Likelihood Ratio | .002 |
| Approx. Chi-Square | 7.785 |
| df | 2 |
| Sig. | .020 |
| Tests the null hypothesis that the residual covariance matrix is proportional to an identity matrix.^a^  a. Design: Intercept + Inspector_ratio | |

| **Multivariate Tests**^a^ | | | | | | |
| --- | --- | --- | --- | --- | --- | --- |
| Effect | | Value | F | Hypothesis df | Error df | Sig. |
| Intercept | Pillai's Trace | .871 | 16.867^b^ | 2.000 | 5.000 | .006 |
|  | Wilks' Lambda | .129 | 16.867^b^ | 2.000 | 5.000 | .006 |
|  | Hotelling's Trace | 6.747 | 16.867^b^ | 2.000 | 5.000 | .006 |
|  | Roy's Largest Root | 6.747 | 16.867^b^ | 2.000 | 5.000 | .006 |
| Inspector_ratio | Pillai's Trace | .644 | 4.531^b^ | 2.000 | 5.000 | .075 |
|  | Wilks' Lambda | .356 | 4.531^b^ | 2.000 | 5.000 | .075 |
|  | Hotelling's Trace | 1.813 | 4.531^b^ | 2.000 | 5.000 | .075 |
|  | Roy's Largest Root | 1.813 | 4.531^b^ | 2.000 | 5.000 | .075 |

| **Multivariate Tests**^a^ | | | | |
| --- | --- | --- | --- | --- |
| Effect | | Partial Eta Squared | Noncent. Parameter | Observed Power^c^ |
| Intercept | Pillai's Trace | .871 | 33.735 | .964 |
|  | Wilks' Lambda | .871 | 33.735 | .964 |
|  | Hotelling's Trace | .871 | 33.735 | .964 |
|  | Roy's Largest Root | .871 | 33.735 | .964 |
| Inspector_ratio | Pillai's Trace | .644 | 9.063 | .493 |
|  | Wilks' Lambda | .644 | 9.063 | .493 |
|  | Hotelling's Trace | .644 | 9.063 | .493 |
|  | Roy's Largest Root | .644 | 9.063 | .493 |
| a. Design: Intercept + Inspector_ratio | | | | |
| b. Exact statistic | | | | |
| c. Computed using alpha = .05 | | | | |

| **Tests of Between-Subjects Effects** | | | | | | |
| --- | --- | --- | --- | --- | --- | --- |
| Source | Dependent Variable | Type III Sum of Squares | df | Mean Square | F | Sig. |
| Corrected Model | Log_fatalrate | .057^a^ | 1 | .057 | 2.095 | .198 |
|  | Log_nonfatalrate | .001^b^ | 1 | .001 | .081 | .786 |
| Intercept | Log_fatalrate | .040 | 1 | .040 | 1.467 | .271 |
|  | Log_nonfatalrate | .049 | 1 | .049 | 4.739 | .072 |
| Inspector_ratio | Log_fatalrate | .057 | 1 | .057 | 2.095 | .198 |
|  | Log_nonfatalrate | .001 | 1 | .001 | .081 | .786 |
| Error | Log_fatalrate | .163 | 6 | .027 |  |  |
|  | Log_nonfatalrate | .062 | 6 | .010 |  |  |
| Total | Log_fatalrate | 2.060 | 8 |  |  |  |
|  | Log_nonfatalrate | 44.843 | 8 |  |  |  |
| Corrected Total | Log_fatalrate | .221 | 7 |  |  |  |
|  | Log_nonfatalrate | .062 | 7 |  |  |  |

| **Tests of Between-Subjects Effects** | | | | |
| --- | --- | --- | --- | --- |
| Source | Dependent Variable | Partial Eta Squared | Noncent. Parameter | Observed Power^c^ |
| Corrected Model | Log_fatalrate | .259 | 2.095 | .231 |
|  | Log_nonfatalrate | .013 | .081 | .057 |
| Intercept | Log_fatalrate | .196 | 1.467 | .177 |
|  | Log_nonfatalrate | .441 | 4.739 | .448 |
| Inspector_ratio | Log_fatalrate | .259 | 2.095 | .231 |
|  | Log_nonfatalrate | .013 | .081 | .057 |
| Error | Log_fatalrate |  |  |  |
|  | Log_nonfatalrate |  |  |  |
| Total | Log_fatalrate |  |  |  |
|  | Log_nonfatalrate |  |  |  |
| Corrected Total | Log_fatalrate |  |  |  |
|  | Log_nonfatalrate |  |  |  |
| a. R Squared = .259 (Adjusted R Squared = .135) | | | | |
| b. R Squared = .013 (Adjusted R Squared = -.151) | | | | |
| c. Computed using alpha = .05 | | | | |

| **Parameter Estimates** | | | | | | |
| --- | --- | --- | --- | --- | --- | --- |
| Dependent Variable | Parameter | B | Std. Error | t | Sig. | 95% Confidence Interval |
|  |  |  |  |  |  | Lower Bound |
| Log_fatalrate | Intercept | -2.466 | 2.036 | -1.211 | .271 | -7.447 |
|  | Inspector_ratio | 3.920 | 2.709 | 1.447 | .198 | -2.707 |
| Log_nonfatalrate | Intercept | 2.721 | 1.250 | 2.177 | .072 | -.338 |
|  | Inspector_ratio | -.473 | 1.663 | -.284 | .786 | -4.543 |

| **Parameter Estimates** | | | | | |
| --- | --- | --- | --- | --- | --- |
| Dependent Variable | Parameter | 95% Confidence Interval | Partial Eta Squared | Noncent. Parameter | Observed Power^a^ |
|  |  | Upper Bound |  |  |  |
| Log_fatalrate | Intercept | 2.515 | .196 | 1.211 | .177 |
|  | Inspector_ratio | 10.548 | .259 | 1.447 | .231 |
| Log_nonfatalrate | Intercept | 5.780 | .441 | 2.177 | .448 |
|  | Inspector_ratio | 3.597 | .013 | .284 | .057 |
| a. Computed using alpha = .05 | | | | | |

| **Between-Subjects SSCP Matrix** | | | | |
| --- | --- | --- | --- | --- |
|  | | | Log_fatalrate | Log_nonfatalrate |
| Hypothesis | Intercept | Log_fatalrate | .040 | -.044 |
|  |  | Log_nonfatalrate | -.044 | .049 |
|  | Inspector_ratio | Log_fatalrate | .057 | -.007 |
|  |  | Log_nonfatalrate | -.007 | .001 |
| Error | | Log_fatalrate | .163 | .086 |
|  |  | Log_nonfatalrate | .086 | .062 |
| Based on Type III Sum of Squares | | | | |

| **Residual SSCP Matrix** | | | |
| --- | --- | --- | --- |
|  | | Log_fatalrate | Log_nonfatalrate |
| Sum-of-Squares and Cross-Products | Log_fatalrate | .163 | .086 |
|  | Log_nonfatalrate | .086 | .062 |
| Covariance | Log_fatalrate | .027 | .014 |
|  | Log_nonfatalrate | .014 | .010 |
| Correlation | Log_fatalrate | 1.000 | .857 |
|  | Log_nonfatalrate | .857 | 1.000 |
| Based on Type III Sum of Squares | | | |

**Lack of Fit**

| **Multivariate Tests** | | | | | |
| --- | --- | --- | --- | --- | --- |
| Dependent Variables | | Value | F | Hypothesis df | Error df |
| Log_fatalrate, Log_nonfatalrate | Pillai's Trace | .^a^ | . | . | . |
|  | Wilks' Lambda | .^a^ | . | . | . |
|  | Hotelling's Trace | .^a^ | . | . | . |
|  | Roy's Largest Root | .^a^ | . | . | . |
| Log_fatalrate | Pillai's Trace | .^a^ | . | . | . |
|  | Wilks' Lambda | .^a^ | . | . | . |
|  | Hotelling's Trace | .^a^ | . | . | . |
|  | Roy's Largest Root | .^a^ | . | . | . |
| Log_nonfatalrate | Pillai's Trace | .^a^ | . | . | . |
|  | Wilks' Lambda | .^a^ | . | . | . |
|  | Hotelling's Trace | .^a^ | . | . | . |
|  | Roy's Largest Root | .^a^ | . | . | . |

| **Multivariate Tests** | | | | |
| --- | --- | --- | --- | --- |
| Dependent Variables | | Sig. | Partial Eta Squared | Noncent. Parameter |
| Log_fatalrate, Log_nonfatalrate | Pillai's Trace | . | . | . |
|  | Wilks' Lambda | . | . | . |
|  | Hotelling's Trace | . | . | . |
|  | Roy's Largest Root | . | . | . |
| Log_fatalrate | Pillai's Trace | . | . | . |
|  | Wilks' Lambda | . | . | . |
|  | Hotelling's Trace | . | . | . |
|  | Roy's Largest Root | . | . | . |
| Log_nonfatalrate | Pillai's Trace | . | . | . |
|  | Wilks' Lambda | . | . | . |
|  | Hotelling's Trace | . | . | . |
|  | Roy's Largest Root | . | . | . |

| **Multivariate Tests** | | |
| --- | --- | --- |
| Dependent Variables | | Observed Power^b^ |
| Log_fatalrate, Log_nonfatalrate | Pillai's Trace | . |
|  | Wilks' Lambda | . |
|  | Hotelling's Trace | . |
|  | Roy's Largest Root | . |
| Log_fatalrate | Pillai's Trace | . |
|  | Wilks' Lambda | . |
|  | Hotelling's Trace | . |
|  | Roy's Largest Root | . |
| Log_nonfatalrate | Pillai's Trace | . |
|  | Wilks' Lambda | . |
|  | Hotelling's Trace | . |
|  | Roy's Largest Root | . |
| a. The pure error sub-matrix corresponding to the selected dependent variables is a zero matrix. | | |
| b. Computed using alpha = .05 | | |

| **Univariate Tests** | | | | | | |
| --- | --- | --- | --- | --- | --- | --- |
| Dependent Variable | Source | Sum of Squares | df | Mean Square | F | Sig. |
| Log_fatalrate | Lack of Fit | .163 | 6 | .027 | . | . |
|  | Pure Error | .000 | 0 | . |  |  |
| Log_nonfatalrate | Lack of Fit | .062 | 6 | .010 | . | . |
|  | Pure Error | .000 | 0 | . |  |  |

| **Univariate Tests** | | | | |
| --- | --- | --- | --- | --- |
| Dependent Variable | Source | Partial Eta Squared | Noncent. Parameter | Observed Power^a^ |
| Log_fatalrate | Lack of Fit | 1.000 | . | . |
|  | Pure Error |  |  |  |
| Log_nonfatalrate | Lack of Fit | 1.000 | . | . |
|  | Pure Error |  |  |  |
| a. Computed using alpha = .05 | | | | |

| **SSCP Matrix** | | | |
| --- | --- | --- | --- |
|  | | Log_fatalrate | Log_nonfatalrate |
| Lack of Fit | Log_fatalrate | .163 | .086 |
|  | Log_nonfatalrate | .086 | .062 |
| Pure Error | Log_fatalrate | .000 | .000 |
|  | Log_nonfatalrate | .000 | .000 |

**Estimated Marginal Means**

| **Grand Mean** | | | | |
| --- | --- | --- | --- | --- |
| Dependent Variable | Mean | Std. Error | 95% Confidence Interval | |
|  |  |  | Lower Bound | Upper Bound |
| Log_fatalrate | .479^a^ | .058 | .337 | .622 |
| Log_nonfatalrate | 2.366^a^ | .036 | 2.278 | 2.454 |
| a. Covariates appearing in the model are evaluated at the following values: Inspector_ratio = .751300. | | | | |

**Observed * Predicted * Std. Residual Plots**

Thus, following analysis have been completed with IBM SPSS 30.0 for Malaysia, as hereunder-

- Descriptive statistics with original data associated with the research variables,
- General linear multivariate regression,
- General linear multivariate regression with bootstrap-2000 with Confidence Intervals of 95%,
- Descriptive statistics with log-transformation of dependent variables,
- Log-general linear multivariate regression with bootstrap-2000 with Confidence Intervals of 95%, and
- Log-general linear multivariate regression analysis.
