# Supplementary material for: OSH inspector ratio to strengthen decent workplace safety and health: a cross-regional trend analysis research
Source: Front Public Health. 2026 Apr 8;14:1806383. doi: 10.3389/fpubh.2026.1806383 (PMC13099828; doi:10.3389/fpubh.2026.1806383)
Supplement: Supplementary file 1 [file Data_Sheet_1.zip › Supplementary document Malaysia - Descriptive and multivariate regression.pdf]

Supplementary Material

Malaysia – Descriptive and multivariate regression analysis

Malaysia is a member nation of International Labour Organization (ILO). IBM SPSS 30.0 software was used to complete descriptive statistics and multivariate regression analysis for Malaysia, with longitudinal data for eight different years. There are six major sections in this supplementary material as listed hereunder:

1. Descriptive statistics with original data associated with the research variables (PP. 2-10),
2. General linear multivariate regression (PP. 11-17),
3. General linear multivariate regression with bootstrap-2000 with Confidence Intervals of 95% (PP. 18-24),
4. Descriptive statistics with log-transformation of dependent variables (PP. 25-33),
5. Log-general linear multivariate regression with bootstrap-2000 with Confidence Intervals of 95% (PP. 34-40), and
6. Log-general linear multivariate regression analysis (PP. 41-47).

Note:

- Inspector ratio or inspector_ratio, or inspector_rate = Independent Variable (IV),
- Falta injury rate or fatal_rate = Dependent Variable (DV1), and
- Nonfatal injury rate or nonfatal_rate = Dependent Variable (DV2).

Descriptive statistics with original research data associated with research variables (PP. 2-10)

Explore

Notes

Output Created		24-FEB-2026 08:54:12
Comments		
Input	Data	E:\Malaysia - Feb 24\Malaysia - Descriptive and multivariate regression.sav
	Active Dataset	DataSet2
	Filter	<none>
	Weight	<none>
	Split File	<none>
	N of Rows in Working Data File	8
Missing Value Handling	Definition of Missing	User-defined missing values for dependent variables are treated as missing.
	Cases Used	Statistics are based on cases with no missing values for any dependent variable or factor used.
Syntax		EXAMINE VARIABLES=Inspector_ratio Fatal_rate Nonfatal_rate<br>/PLOT BOXPLOT HISTOGRAM NPPLOT<br>/COMPARE GROUPS<br>/STATISTICS DESCRIPTIVES EXTREME<br>/CINTERVAL 95<br>/MISSING LISTWISE<br>/NOTOTAL.
Resources	Processor Time	00:00:05.31
	Elapsed Time	00:00:01.90

Case Processing Summary

	Cases					
	Valid		Missing		Total	
	N	Percent	N	Percent	N	Percent
Inspector_ratio	8	100.0%	0	0.0%	8	100.0%
Fatal_rate	8	100.0%	0	0.0%	8	100.0%
Nonfatal_rate	8	100.0%	0	0.0%	8	100.0%

Descriptives

			Statistic	Std. Error
Inspector_ratio	Mean		.751300	.0081439
	95% Confidence Interval for Mean	Lower Bound	.732043	
		Upper Bound	.770557	
	5% Trimmed Mean		.751217	
	Median		.748150	
	Variance		.001	
	Std. Deviation		.0230344	
	Minimum		.7147	
	Maximum		.7894	
	Range		.0747	
	Interquartile Range		.0312	
	Skewness		.165	.752
	Kurtosis		.144	1.481
Fatal_rate	Mean		3.242500	.4577731
	95% Confidence Interval for Mean	Lower Bound	2.160039	
		Upper Bound	4.324961	
	5% Trimmed Mean		3.221111	
	Median		2.975000	
	Variance		1.676	
	Std. Deviation		1.2947780	
	Minimum		2.0300	
	Maximum		4.8400	
	Range		2.8100	
	Interquartile Range		2.6050	
	Skewness		.233	.752
	Kurtosis		-2.298	1.481
Nonfatal_rate	Mean		236.632500	15.7712924
	95% Confidence Interval for Mean	Lower Bound	199.339320	
		Upper Bound	273.925680	
	5% Trimmed Mean		239.251111	
	Median		239.905000	
	Variance		1989.869	
	Std. Deviation		44.6079512	
	Minimum		142.9700	
	Maximum		283.1600	
	Range		140.1900	
	Interquartile Range		53.0250	
	Skewness		-1.367	.752
	Kurtosis		2.466	1.481

Extreme Values[a]

			Case Number	Value
Inspector_ratio	Highest	1	1	.7894
		2	6	.7715
		3	2	.7615
		4	3	.7536
	Lowest	1	8	.7147
		2	4	.7371
		3	7	.7399
		4	5	.7427
Fatal_rate	Highest	1	1	4.8400
		2	2	4.8400
		3	3	4.1400
		4	4	3.8300
	Lowest	1	6	2.0300
		2	8	2.0500
		3	7	2.0900
		4	5	2.1200
Nonfatal_rate	Highest	1	2	283.1600
		2	1	276.1600
		3	4	267.1700
		4	8	243.9500
	Lowest	1	6	142.9700
		2	5	219.8800
		3	7	223.9100
		4	3	235.8600

a. The requested number of extreme values exceeds the number of data points. A smaller number of extremes is displayed.

Tests of Normality

	Kolmogorov-Smirnov[a]			Shapiro-Wilk		
	Statistic	df	Sig.	Statistic	df	Sig.
Inspector_ratio	.146	8	.200*	.985	8	.983
Fatal_rate	.307	8	.025	.787	8	.021
Nonfatal_rate	.229	8	.200*	.879	8	.185

*. This is a lower bound of the true significance.

a. Lilliefors Significance Correction

Inspector_ratio

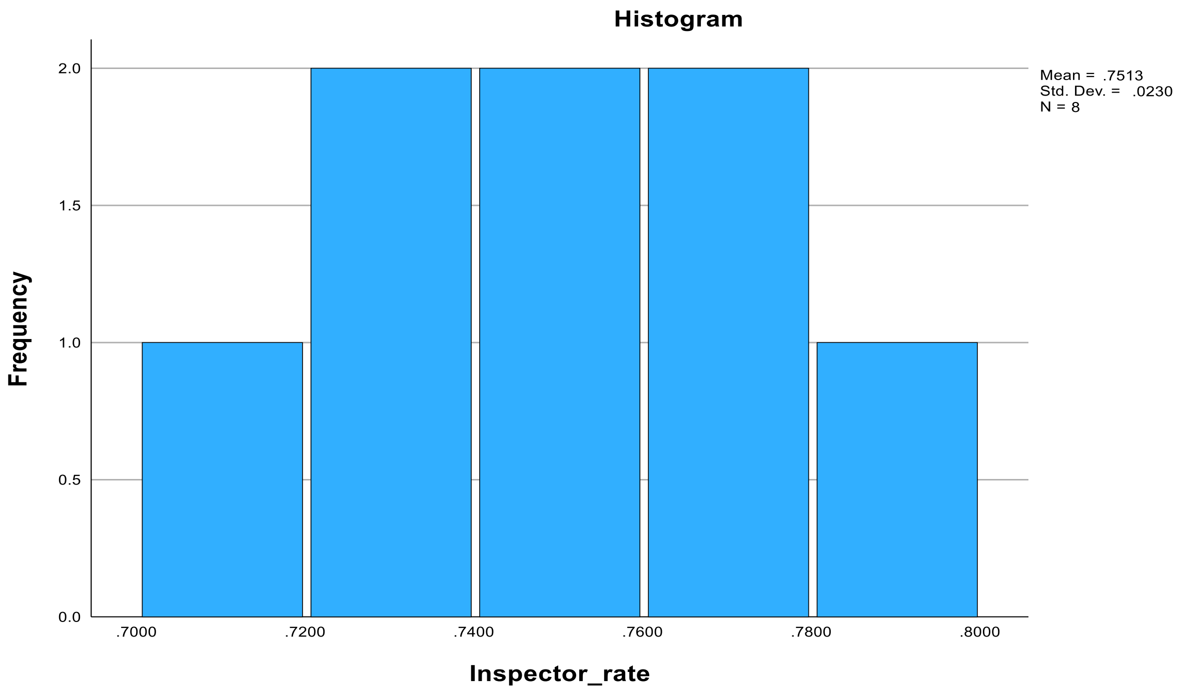

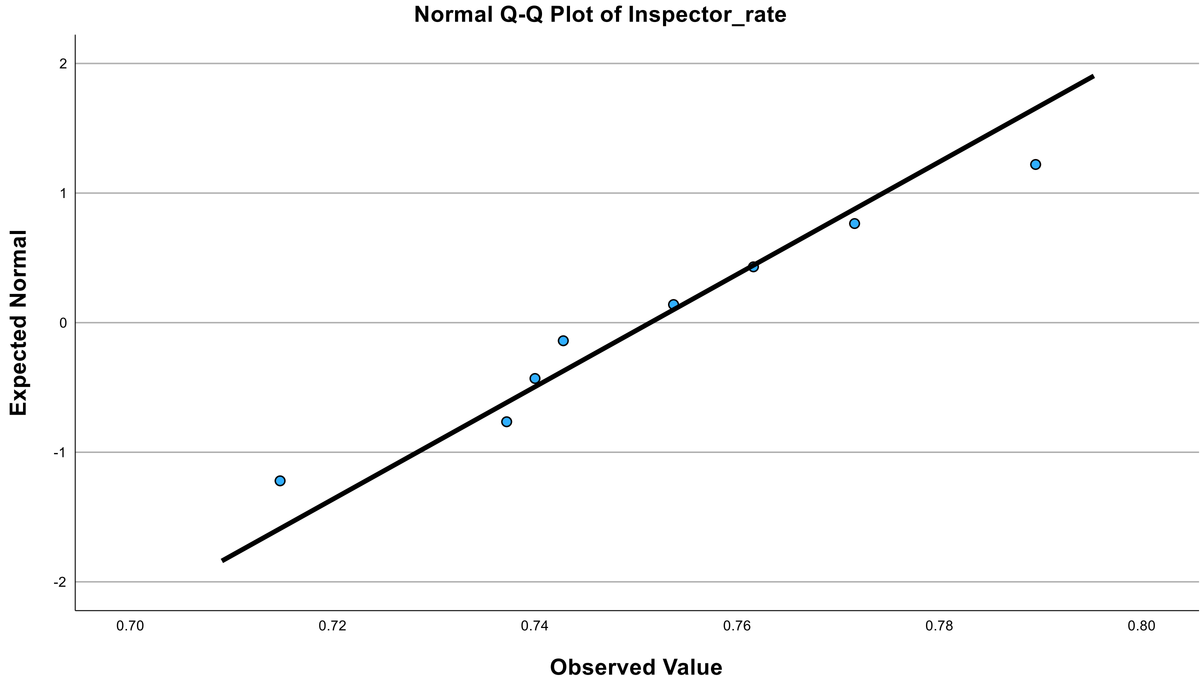

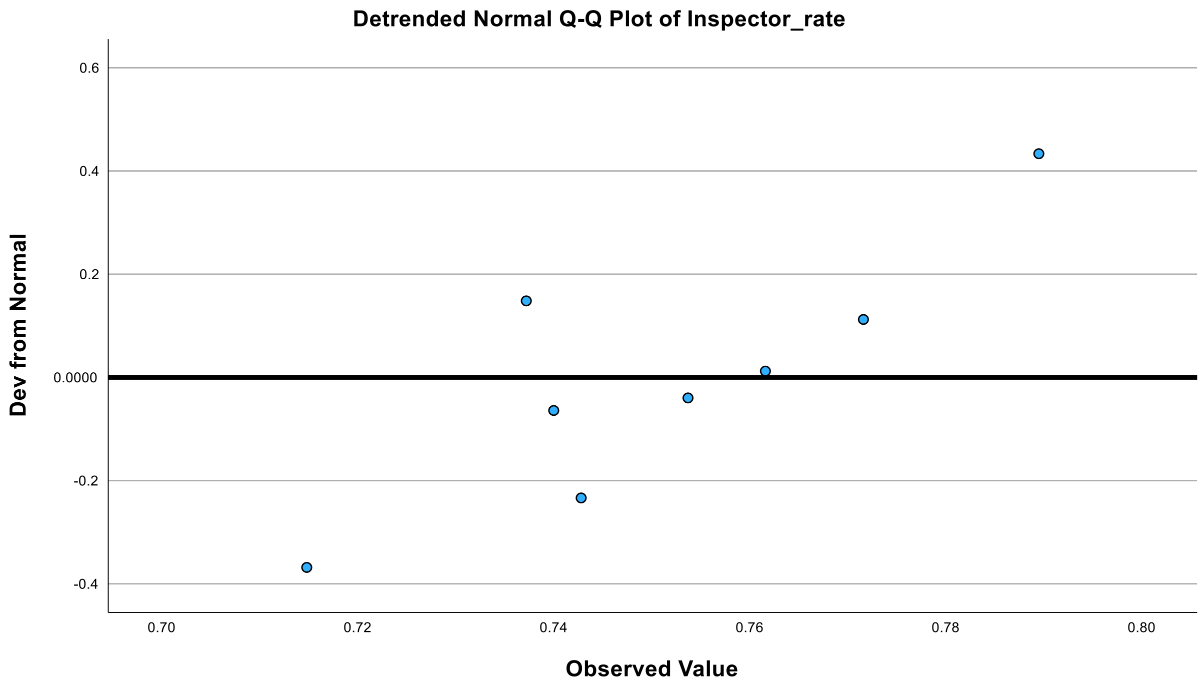
Detrended Normal Q-Q Plot of Inspector_rate
Dev from Normal
0.6
0.4
0.2
0.0000
-0.2
-0.4
0.70
0.72
0.74
0.76
0.78
0.80
Observed Value

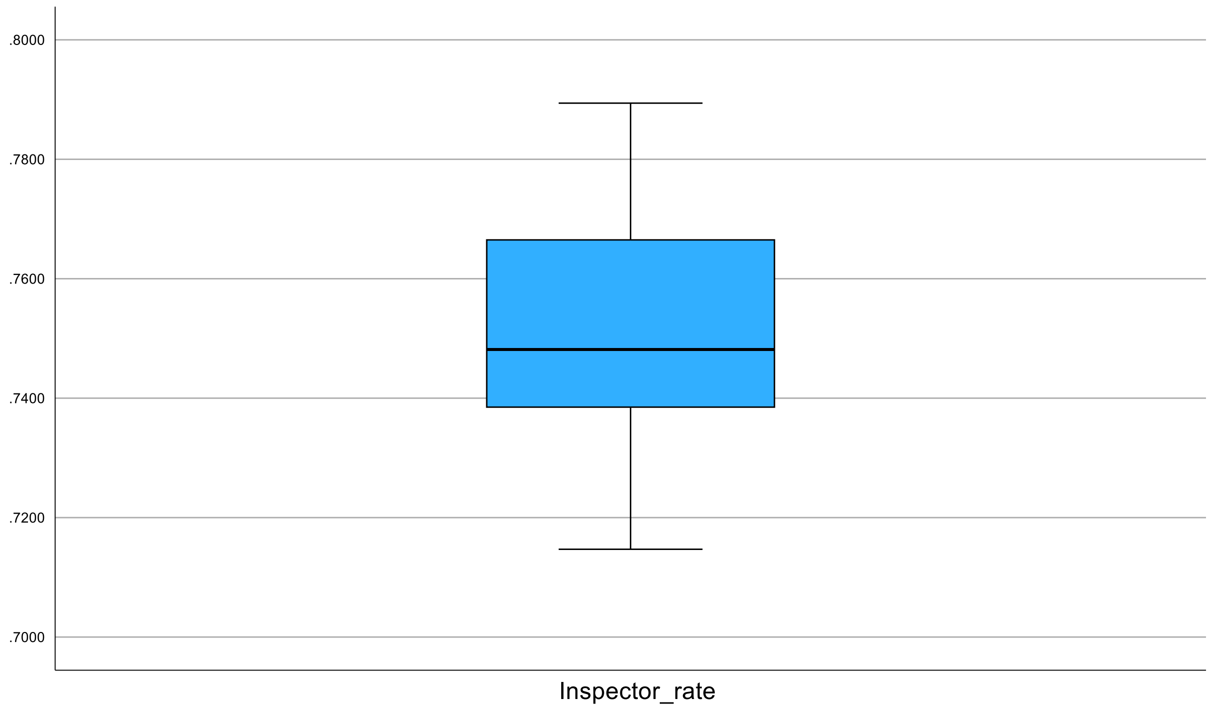
.8000
.7800
.7600
.7400
.7200
.7000
Inspector_rate

Fatal_rate

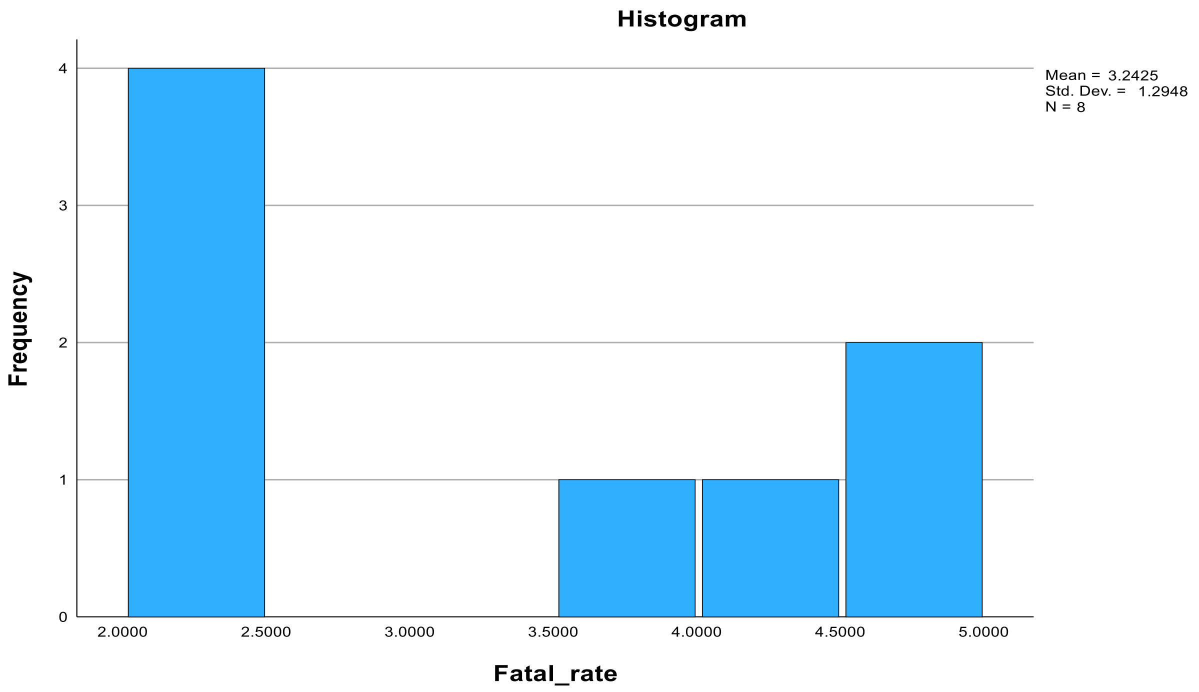
Histogram
Mean = 3.2425
Std. Dev. = 1.2948
N = 8
Frequency
0
1
2
3
4
2.0000
2.5000
3.0000
3.5000
4.0000
4.5000
5.0000
Fatal_rate

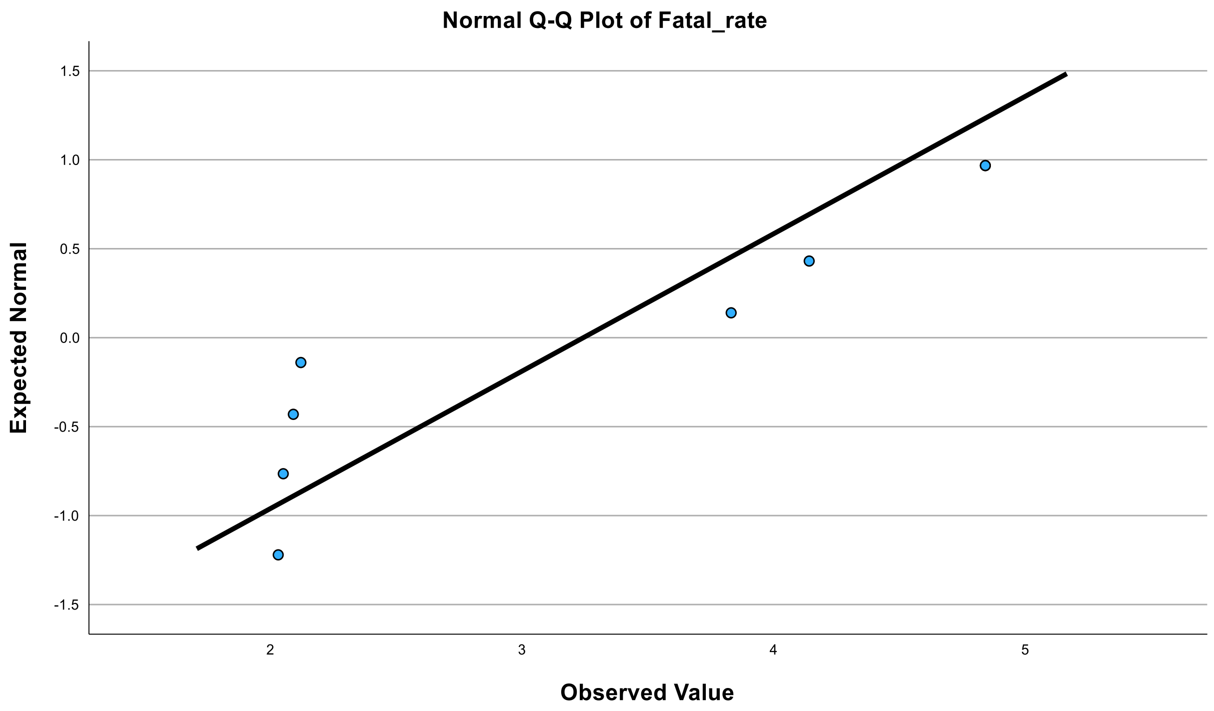
Normal Q-Q Plot of Fatal_rate
Expected Normal
1.5
1.0
0.5
0.0
-0.5
-1.0
-1.5
2
3
4
5
Observed Value

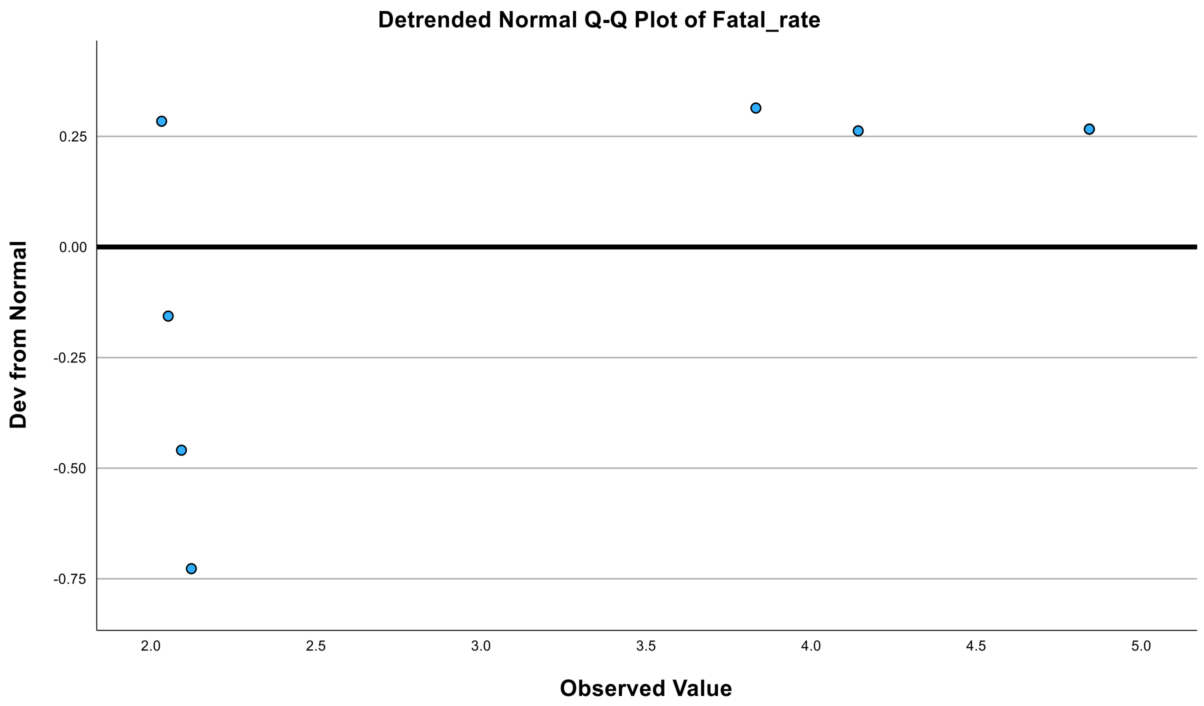

Detrended Normal Q-Q Plot of Fatal_rate
Dev from Normal
0.25
0.00
-0.25
-0.50
-0.75
2.0
2.5
3.0
3.5
4.0
4.5
5.0
Observed Value

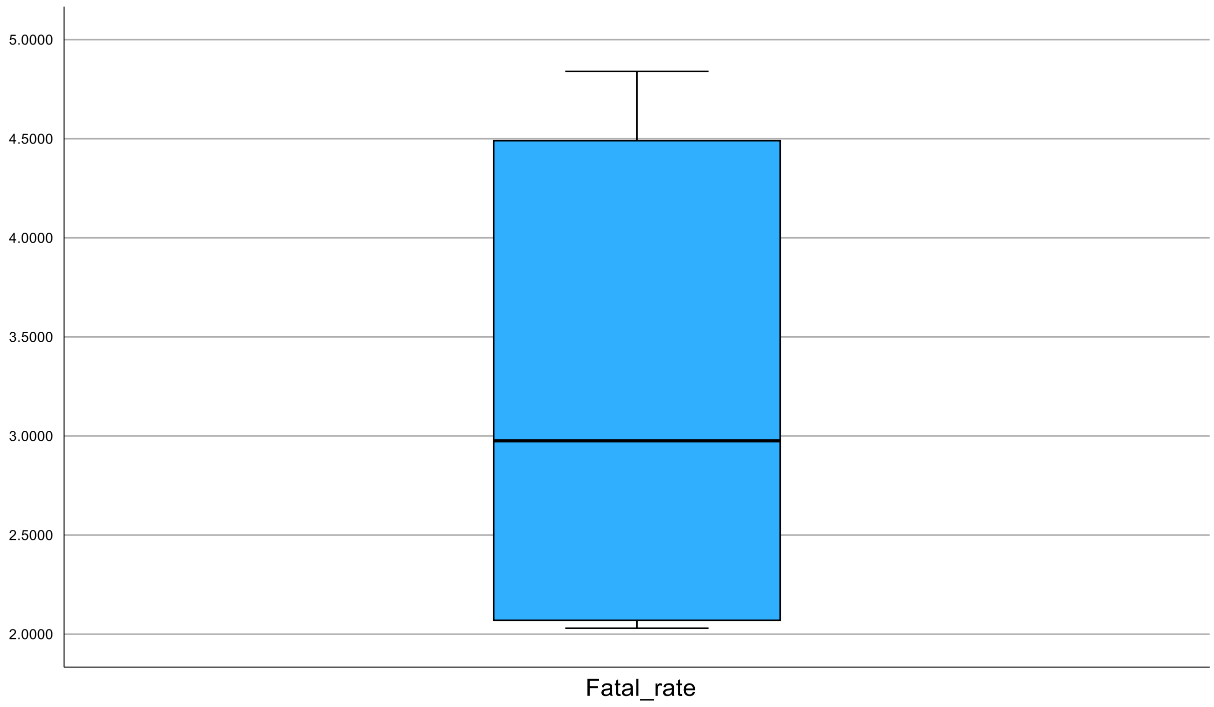

5.0000
4.5000
4.0000
3.5000
3.0000
2.5000
2.0000
Fatal_rate

Nonfatal_rate

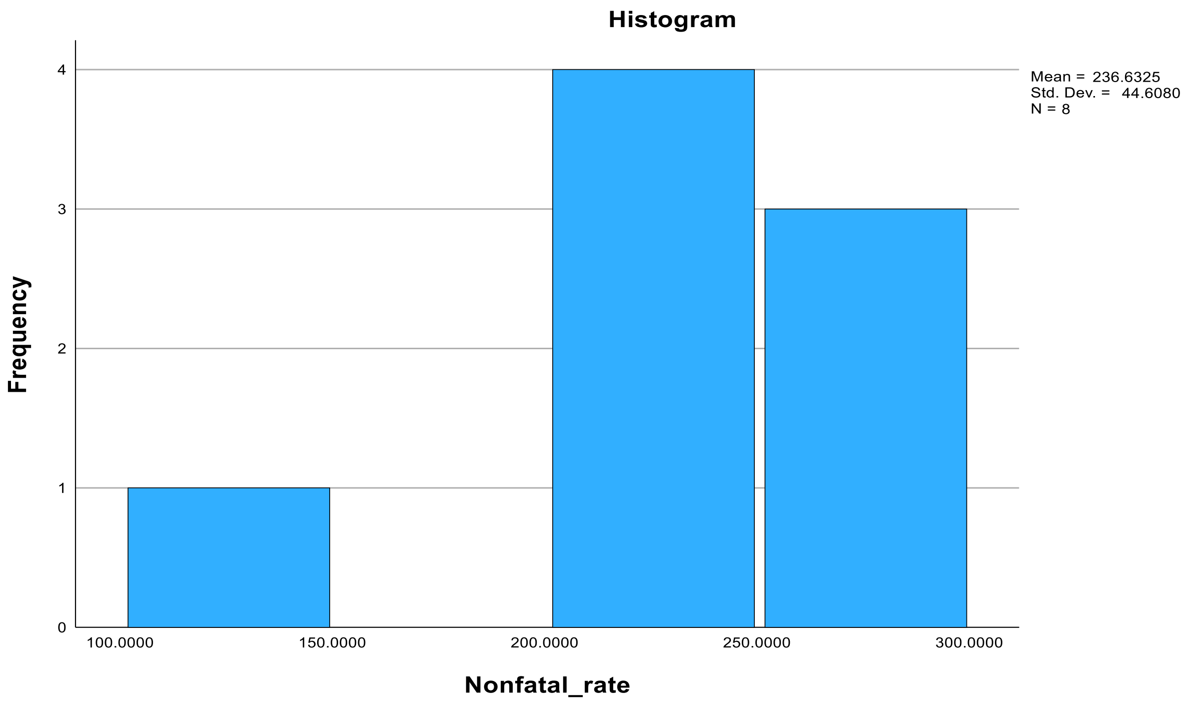

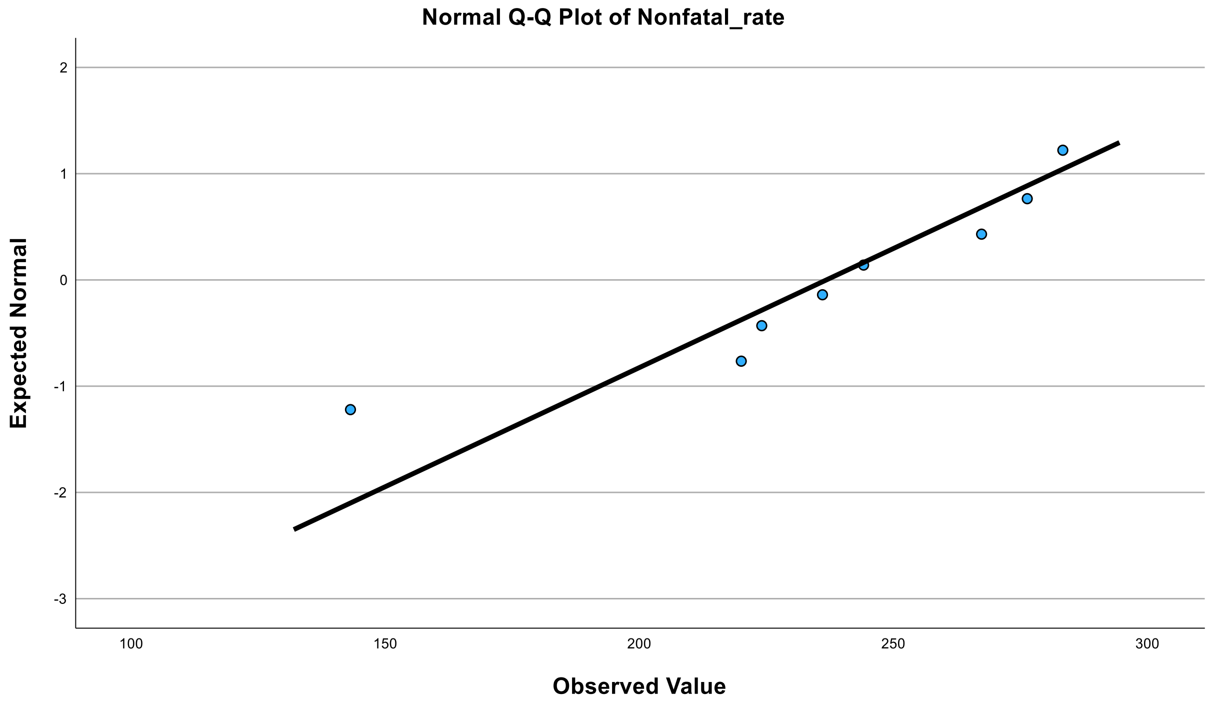

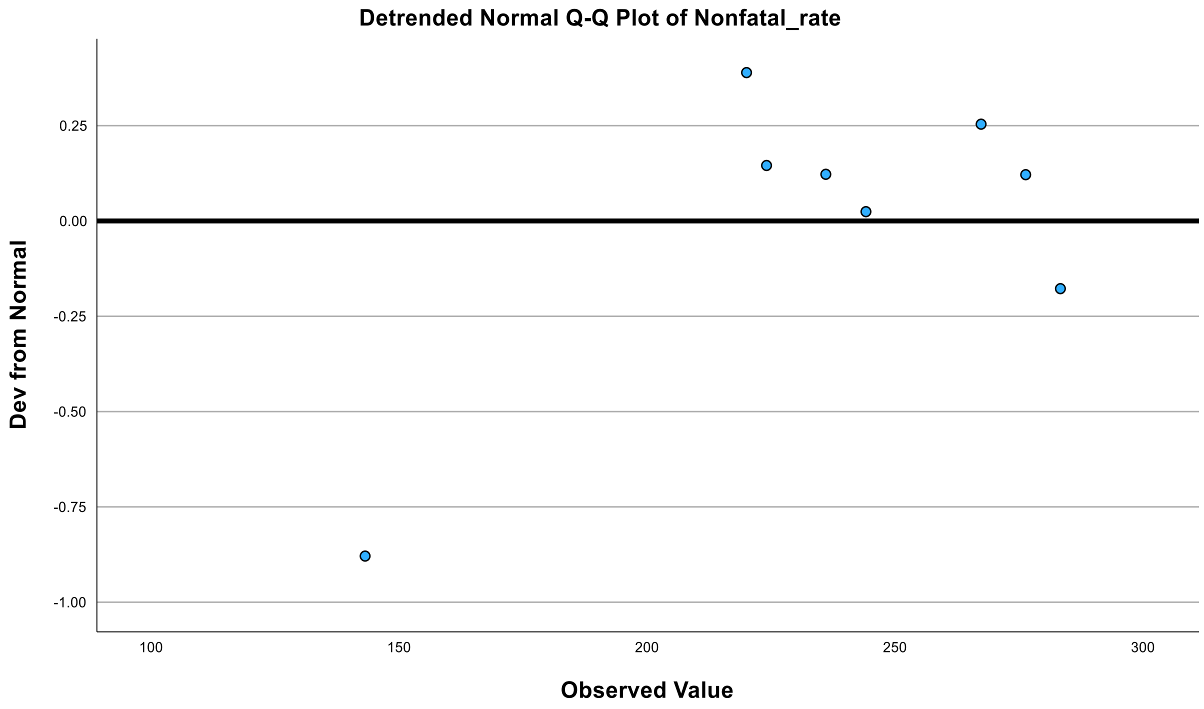
Detrended Normal Q-Q Plot of Nonfatal_rate
Dev from Normal
0.25
0.00
-0.25
-0.50
-0.75
-1.00
100
150
200
250
300
Observed Value

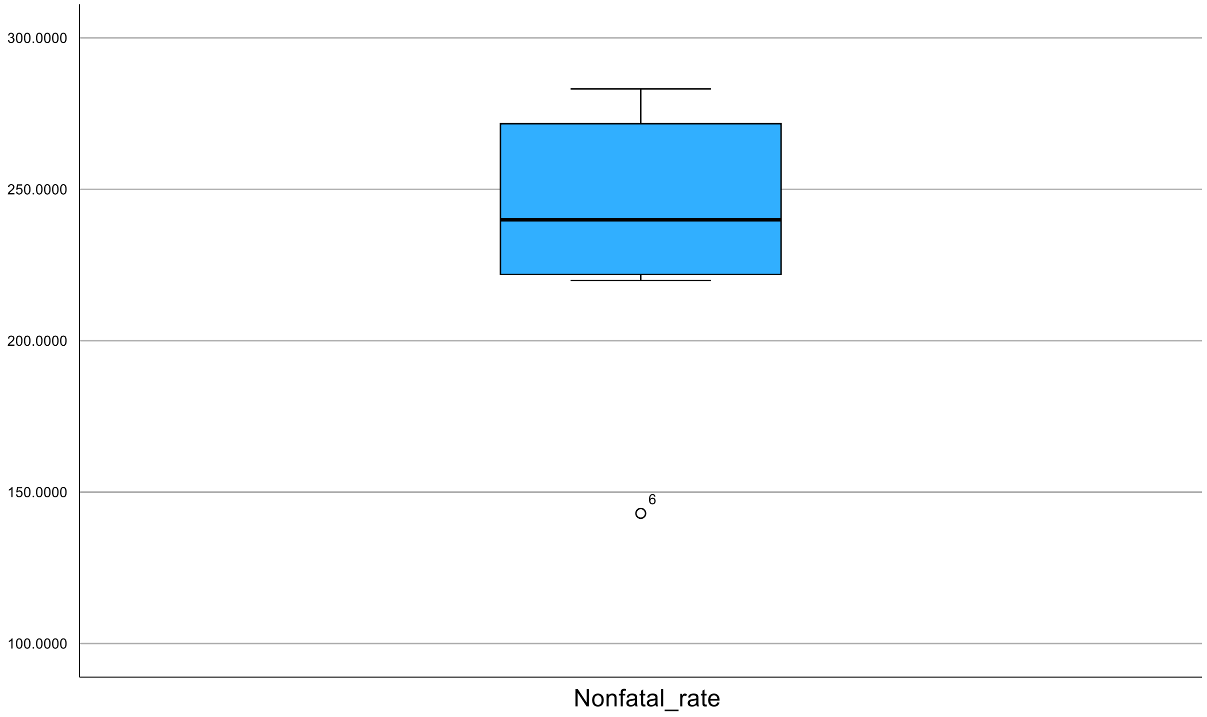
300.0000
250.0000
200.0000
150.0000
100.0000
6
Nonfatal_rate

General linear multivariate regression (PP. 11-17)

Notes

Output Created		24-FEB-2026 08:58:39
Comments		
Input	Data	E:\Malaysia - Feb 24\Malaysia - Descriptive and multivariate regression.sav
	Active Dataset	DataSet2
	Filter	<none>
	Weight	<none>
	Split File	<none>
	N of Rows in Working Data File	8
Missing Value Handling	Definition of Missing	User-defined missing values are treated as missing.
	Cases Used	Statistics are based on all cases with valid data for all variables in the model.
Syntax		GLM Fatal_rate Nonfatal_rate WITH Inspector_ratio<br>/METHOD=SSTYPE(3)<br>/INTERCEPT=INCLUDE<br>/SAVE=PRED SEPRED RESID ZRESID COOK LEVER<br>/EMMEANS=TABLES(OVERALL) WITH(Inspector_ratio=MEAN)<br>/PRINT=DESCRIPTIVE ETASQ OPOWER PARAMETER TEST(SSCP) RSSCP HOMOGENEITY LOF<br>/PLOT=SPREADLEVEL RESIDUALS<br>/CRITERIA=ALPHA(.05)<br>/DESIGN=Inspector_ratio.
Resources	Processor Time	00:00:00.53
	Elapsed Time	00:00:00.29
Variables Created or Modified	PRE_1	Predicted Value for Fatal_rate
	PRE_2	Predicted Value for Nonfatal_rate
	SEP_1	Standard Error of Predicted Value for Fatal_rate
	SEP_2	Standard Error of Predicted Value for Nonfatal_rate
	RES_1	Residual for Fatal_rate
	RES_2	Residual for Nonfatal_rate
	ZRE_1	Standardized Residual for Fatal_rate
	ZRE_2	Standardized Residual for Nonfatal_rate
	COO_1	Cook's Distance for Fatal_rate
	COO_2	Cook's Distance for Nonfatal_rate
	LEV_1	Uncentered Leverage Value for Fatal_rate
	LEV_2	Uncentered Leverage Value for Nonfatal_rate

Warnings

Warnings
The HOMOGENEITY specification in the PRINT subcommand will be ignored because there are no between-subjects factors.
The SPREADLEVEL specification has been found in the PLOT subcommand, but the model includes no factors. The spread versus level plot will not be produced.

Descriptive Statistics

	Mean	Std. Deviation	N
Fatal_rate	3.242500	1.2947780	8
Nonfatal_rate	236.632500	44.6079512	8

Bartlett's Test of Sphericity[a]

Likelihood Ratio	.000
Approx. Chi-Square	38.100
df	2
Sig.	<.001

Tests the null hypothesis that the residual covariance matrix is proportional to an identity matrix.[a]
a. Design: Intercept + Inspector_ratio

Multivariate Tests[a]

Effect		Value	F	Hypothesis df	Error df	Sig.
Intercept	Pillai's Trace	.728	6.705[b]	2.000	5.000	.038
	Wilks' Lambda	.272	6.705[b]	2.000	5.000	.038
	Hotelling's Trace	2.682	6.705[b]	2.000	5.000	.038
	Roy's Largest Root	2.682	6.705[b]	2.000	5.000	.038
Inspector_ratio	Pillai's Trace	.694	5.677[b]	2.000	5.000	.052
	Wilks' Lambda	.306	5.677[b]	2.000	5.000	.052
	Hotelling's Trace	2.271	5.677[b]	2.000	5.000	.052
	Roy's Largest Root	2.271	5.677[b]	2.000	5.000	.052

Multivariate Tests[a]

Effect		Partial Eta Squared	Noncent. Parameter	Observed Power[c]
Intercept	Pillai's Trace	.728	13.410	.660
	Wilks' Lambda	.728	13.410	.660
	Hotelling's Trace	.728	13.410	.660
	Roy's Largest Root	.728	13.410	.660
Inspector_ratio	Pillai's Trace	.694	11.354	.587
	Wilks' Lambda	.694	11.354	.587
	Hotelling's Trace	.694	11.354	.587
	Roy's Largest Root	.694	11.354	.587

a. Design: Intercept + Inspector_ratio
b. Exact statistic
c. Computed using alpha = .05

Tests of Between-Subjects Effects

Source	Dependent Variable	Type III Sum of Squares	df	Mean Square	F	Sig.
Corrected Model	Fatal_rate	3.427[a]	1	3.427	2.475	.167
	Nonfatal_rate	28.531[b]	1	28.531	.012	.915
Intercept	Fatal_rate	2.521	1	2.521	1.820	.226
	Nonfatal_rate	601.542	1	601.542	.260	.629
Inspector_ratio	Fatal_rate	3.427	1	3.427	2.475	.167
	Nonfatal_rate	28.531	1	28.531	.012	.915
Error	Fatal_rate	8.308	6	1.385		
	Nonfatal_rate	13900.554	6	2316.759		
Total	Fatal_rate	95.846	8			
	Nonfatal_rate	461888.606	8			
Corrected Total	Fatal_rate	11.735	7			
	Nonfatal_rate	13929.085	7			

Tests of Between-Subjects Effects

Source	Dependent Variable	Partial Eta Squared	Noncent. Parameter	Observed Power[c]
Corrected Model	Fatal_rate	.292	2.475	.264
	Nonfatal_rate	.002	.012	.051
Intercept	Fatal_rate	.233	1.820	.207
	Nonfatal_rate	.041	.260	.072
Inspector_ratio	Fatal_rate	.292	2.475	.264
	Nonfatal_rate	.002	.012	.051
Error	Fatal_rate			
	Nonfatal_rate			
Total	Fatal_rate			
	Nonfatal_rate			
Corrected Total	Fatal_rate			
	Nonfatal_rate			

a. R Squared = .292 (Adjusted R Squared = .174)
b. R Squared = .002 (Adjusted R Squared = -.164)
c. Computed using alpha = .05

Parameter Estimates

Dependent Variable	Parameter	B	Std. Error	t	Sig.	95% Confidence Interval Lower Bound
Fatal_rate	Intercept	-19.580	14.512	-1.349	.226	-55.090
	Inspector_ratio	30.378	19.308	1.573	.167	-16.867
Nonfatal_rate	Intercept	302.481	593.616	.510	.629	-1150.045
	Inspector_ratio	-87.646	789.794	-.111	.915	-2020.201

Parameter Estimates

Dependent Variable	Parameter	95% Confidence Interval Upper Bound	Partial Eta Squared	Noncent. Parameter	Observed Power[a]
Fatal_rate	Intercept	15.930	.233	1.349	.207
	Inspector_ratio	77.623	.292	1.573	.264
Nonfatal_rate	Intercept	1755.007	.041	.510	.072
	Inspector_ratio	1844.909	.002	.111	.051

a. Computed using alpha = .05

Between-Subjects SSCP Matrix

			Fatal_rate	Nonfatal_rate
Hypothesis	Intercept	Fatal_rate	2.521	-38.939
		Nonfatal_rate	-38.939	601.542
	Inspector_ratio	Fatal_rate	3.427	-9.889
		Nonfatal_rate	-9.889	28.531
Error		Fatal_rate	8.308	302.916
		Nonfatal_rate	302.916	13900.554

Based on Type III Sum of Squares

Residual SSCP Matrix

		Fatal_rate	Nonfatal_rate
Sum-of-Squares and Cross-Products	Fatal_rate	8.308	302.916
	Nonfatal_rate	302.916	13900.554
Covariance	Fatal_rate	1.385	50.486
	Nonfatal_rate	50.486	2316.759
Correlation	Fatal_rate	1.000	.891
	Nonfatal_rate	.891	1.000

Based on Type III Sum of Squares

Lack of Fit

Multivariate Tests

Dependent Variables		Value	F	Hypothesis df	Error df
Fatal_rate, Nonfatal_rate	Pillai's Trace	.[a]	.	.	.
	Wilks' Lambda	.[a]	.	.	.
	Hotelling's Trace	.[a]	.	.	.
	Roy's Largest Root	.[a]	.	.	.
Fatal_rate	Pillai's Trace	.[a]	.	.	.
	Wilks' Lambda	.[a]	.	.	.
	Hotelling's Trace	.[a]	.	.	.
	Roy's Largest Root	.[a]	.	.	.
Nonfatal_rate	Pillai's Trace	.[a]	.	.	.
	Wilks' Lambda	.[a]	.	.	.
	Hotelling's Trace	.[a]	.	.	.
	Roy's Largest Root	.[a]	.	.	.

Multivariate Tests

Dependent Variables		Sig.	Partial Eta Squared	Noncent. Parameter
Fatal_rate, Nonfatal_rate	Pillai's Trace	.	.	.
	Wilks' Lambda	.	.	.
	Hotelling's Trace	.	.	.
	Roy's Largest Root	.	.	.
Fatal_rate	Pillai's Trace	.	.	.
	Wilks' Lambda	.	.	.
	Hotelling's Trace	.	.	.
	Roy's Largest Root	.	.	.
Nonfatal_rate	Pillai's Trace	.	.	.
	Wilks' Lambda	.	.	.
	Hotelling's Trace	.	.	.
	Roy's Largest Root	.	.	.

Multivariate Tests

Dependent Variables		Observed Power[b]
Fatal_rate, Nonfatal_rate	Pillai's Trace	.
	Wilks' Lambda	.
	Hotelling's Trace	.
	Roy's Largest Root	.
Fatal_rate	Pillai's Trace	.
	Wilks' Lambda	.
	Hotelling's Trace	.
	Roy's Largest Root	.
Nonfatal_rate	Pillai's Trace	.
	Wilks' Lambda	.
	Hotelling's Trace	.
	Roy's Largest Root	.

a. The pure error sub-matrix corresponding to the selected dependent variables is a zero matrix.
b. Computed using alpha = .05

Univariate Tests

Dependent Variable	Source	Sum of Squares	df	Mean Square	F	Sig.
Fatal_rate	Lack of Fit	8.308	6	1.385	.	.
	Pure Error	.000	0	.		
Nonfatal_rate	Lack of Fit	13900.554	6	2316.759	.	.
	Pure Error	.000	0	.		

Univariate Tests

Dependent Variable	Source	Partial Eta Squared	Noncent. Parameter	Observed Power[a]
Fatal_rate	Lack of Fit	1.000	.	.
	Pure Error			
Nonfatal_rate	Lack of Fit	1.000	.	.
	Pure Error			

a. Computed using alpha = .05

SSCP Matrix

		Fatal_rate	Nonfatal_rate
Lack of Fit	Fatal_rate	8.308	302.916
	Nonfatal_rate	302.916	13900.554
Pure Error	Fatal_rate	.000	.000
	Nonfatal_rate	.000	.000

Estimated Marginal Means

Grand Mean

Dependent Variable	Mean	Std. Error	95% Confidence Interval Lower Bound	95% Confidence Interval Upper Bound
Fatal_rate	3.242[a]	.416	2.225	4.260
Nonfatal_rate	236.633[a]	17.017	194.992	278.273

a. Covariates appearing in the model are evaluated at the following values: Inspector_ratio = .751300.

Observed * Predicted * Std. Residual Plots

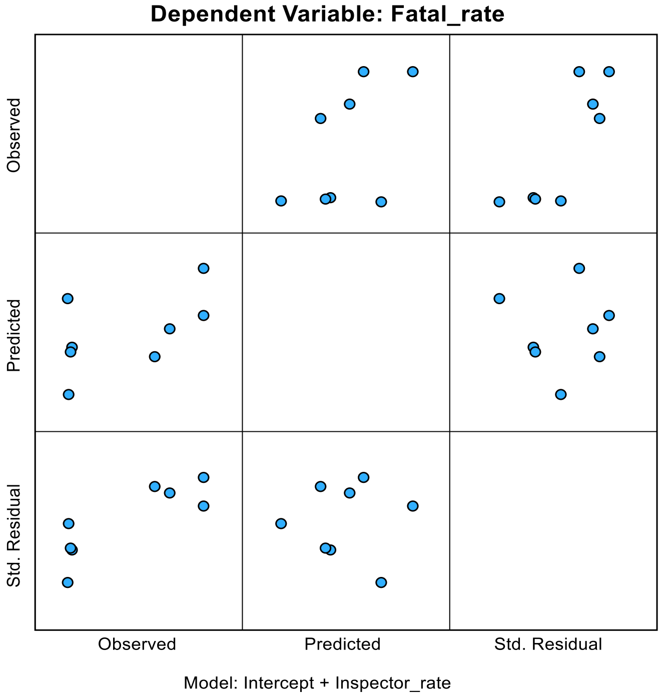

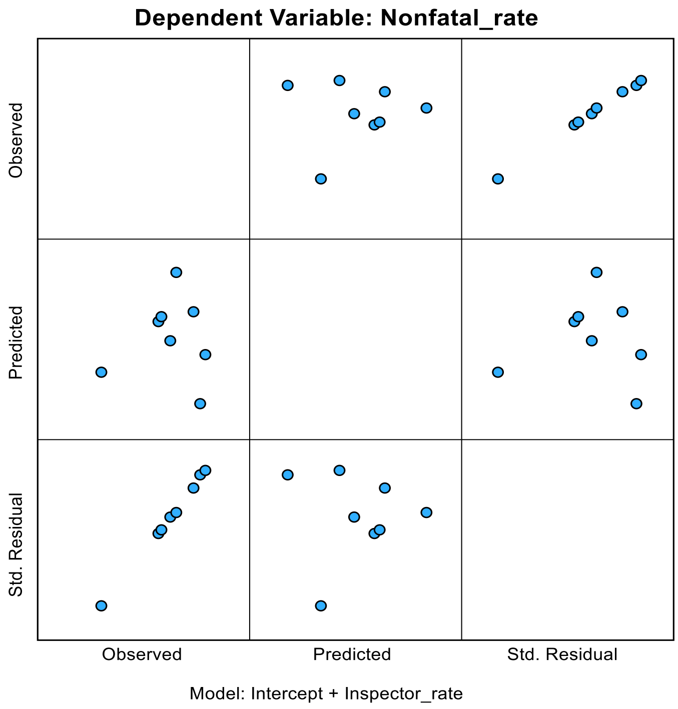
Dependent Variable: Nonfatal_rate
Observed
Predicted
Std. Residual
Observed
Predicted
Std. Residual
Model: Intercept + Inspector_rate

Continued.

General linear multivariate regression with bootstrap-2000 with Confidence Intervals of 95% (PP. 18-24)

Bootstrap

Notes

Output Created		24-FEB-2026 09:02:26
Comments		
Input	Data	E:\Malaysia - Feb 24\Malaysia - Descriptive and multivariate regression.sav
	Active Dataset	DataSet2
	Filter	<none>
	Weight	<none>
	Split File	<none>
	N of Rows in Working Data File	8
Syntax		BOOTSTRAP<br>/SAMPLING METHOD=SIMPLE<br>/VARIABLES TARGET=Fatal_rate Nonfatal_rate INPUT= Inspector_ratio<br>/CRITERIA CILEVEL=95 CITYPE=PERCENTILE NSAMPLES=2000<br>/MISSING USERMISSING=EXCLUDE.
Resources	Processor Time	00:00:00.03
	Elapsed Time	00:00:00.02

Bootstrap Specifications

Sampling Method	Simple
Number of Samples	2000
Confidence Interval Level	95.0%
Confidence Interval Type	Percentile

General Linear Model

Notes

Output Created		24-FEB-2026 09:02:26
Comments		
Input	Data	E:\Malaysia - Feb 24\Malaysia - Descriptive and multivariate regression.sav
	Active Dataset	DataSet2
	Filter	<none>
	Weight	<none>
	Split File	<none>
	N of Rows in Working Data File	16008

Missing Value Handling	Definition of Missing	User-defined missing values are treated as missing.
	Cases Used	Statistics are based on all cases with valid data for all variables in the model.
Syntax		GLM Fatal_rate Nonfatal_rate WITH Inspector_ratio<br>/METHOD=SSTYPE(3)<br>/INTERCEPT=INCLUDE<br>/SAVE=PRED SEPRED RESID ZRESID COOK LEVER<br>/EMMEANS=TABLES(OVERALL) WITH(Inspector_ratio=MEAN)<br>/PRINT=DESCRIPTIVE ETASQ OPOWER PARAMETER TEST(SSCP) RSSCP HOMOGENEITY LOF<br>/PLOT=SPREADLEVEL RESIDUALS<br>/CRITERIA=ALPHA(.05)<br>/DESIGN=Inspector_ratio.
Resources	Processor Time	00:00:26.11
	Elapsed Time	00:00:25.47

Warnings

The HOMOGENEITY specification in the PRINT subcommand will be ignored because there are no between-subjects factors.
The SPREADLEVEL specification has been found in the PLOT subcommand, but the model includes no factors. The spread versus level plot will not be produced.
This command is trying to save new variables while bootstrapping is in effect. The new variables will be lost.<br>Execution of this command stops.

Descriptive Statistics

	Mean	Std. Deviation	N
Fatal_rate	3.242500	1.2947780	8
Nonfatal_rate	236.632500	44.6079512	8

Bartlett's Test of Sphericity[a]

Likelihood Ratio	.000
Approx. Chi-Square	38.100
df	2
Sig.	<.001

Tests the null hypothesis that the residual covariance matrix is proportional to an identity matrix.[a]

a. Design: Intercept + Inspector_ratio

Multivariate Tests[a]

Effect		Value	F	Hypothesis df	Error df	Sig.
Intercept	Pillai's Trace	.728	6.705[b]	2.000	5.000	.038
	Wilks' Lambda	.272	6.705[b]	2.000	5.000	.038
	Hotelling's Trace	2.682	6.705[b]	2.000	5.000	.038
	Roy's Largest Root	2.682	6.705[b]	2.000	5.000	.038
Inspector_ratio	Pillai's Trace	.694	5.677[b]	2.000	5.000	.052
	Wilks' Lambda	.306	5.677[b]	2.000	5.000	.052
	Hotelling's Trace	2.271	5.677[b]	2.000	5.000	.052
	Roy's Largest Root	2.271	5.677[b]	2.000	5.000	.052

Multivariate Tests[a]

Effect		Partial Eta Squared	Noncent. Parameter	Observed Power[c]
Intercept	Pillai's Trace	.728	13.410	.660
	Wilks' Lambda	.728	13.410	.660
	Hotelling's Trace	.728	13.410	.660
	Roy's Largest Root	.728	13.410	.660
Inspector_ratio	Pillai's Trace	.694	11.354	.587
	Wilks' Lambda	.694	11.354	.587
	Hotelling's Trace	.694	11.354	.587
	Roy's Largest Root	.694	11.354	.587

a. Design: Intercept + Inspector_ratio
b. Exact statistic
c. Computed using alpha = .05

Tests of Between-Subjects Effects

Source	Dependent Variable	Type III Sum of Squares	df	Mean Square	F	Sig.
Corrected Model	Fatal_rate	3.427[a]	1	3.427	2.475	.167
	Nonfatal_rate	28.531[b]	1	28.531	.012	.915
Intercept	Fatal_rate	2.521	1	2.521	1.820	.226
	Nonfatal_rate	601.542	1	601.542	.260	.629
Inspector_ratio	Fatal_rate	3.427	1	3.427	2.475	.167
	Nonfatal_rate	28.531	1	28.531	.012	.915
Error	Fatal_rate	8.308	6	1.385		
	Nonfatal_rate	13900.554	6	2316.759		
Total	Fatal_rate	95.846	8			
	Nonfatal_rate	461888.606	8			
Corrected Total	Fatal_rate	11.735	7			
	Nonfatal_rate	13929.085	7			

Tests of Between-Subjects Effects

Source	Dependent Variable	Partial Eta Squared	Noncent. Parameter	Observed Power[c]
Corrected Model	Fatal_rate	.292	2.475	.264
	Nonfatal_rate	.002	.012	.051
Intercept	Fatal_rate	.233	1.820	.207
	Nonfatal_rate	.041	.260	.072
Inspector_ratio	Fatal_rate	.292	2.475	.264
	Nonfatal_rate	.002	.012	.051
Error	Fatal_rate			
	Nonfatal_rate			
Total	Fatal_rate			
	Nonfatal_rate			
Corrected Total	Fatal_rate			
	Nonfatal_rate			

a. R Squared = .292 (Adjusted R Squared = .174)
b. R Squared = .002 (Adjusted R Squared = -.164)
c. Computed using alpha = .05

Parameter Estimates

Dependent Variable	Parameter	B	Std. Error	t	Sig.	95% Confidence Interval Lower Bound
Fatal_rate	Intercept	-19.580	14.512	-1.349	.226	-55.090
	Inspector_ratio	30.378	19.308	1.573	.167	-16.867
Nonfatal_rate	Intercept	302.481	593.616	.510	.629	-1150.045
	Inspector_ratio	-87.646	789.794	-.111	.915	-2020.201

Parameter Estimates

Dependent Variable	Parameter	95% Confidence Interval Upper Bound	Partial Eta Squared	Noncent. Parameter	Observed Power[a]
Fatal_rate	Intercept	15.930	.233	1.349	.207
	Inspector_ratio	77.623	.292	1.573	.264
Nonfatal_rate	Intercept	1755.007	.041	.510	.072
	Inspector_ratio	1844.909	.002	.111	.051

a. Computed using alpha = .05

Bootstrap for Parameter Estimates

Dependent Variable	Parameter	B	Bootstrap[a] Bias	Std. Error	Sig. (2-tailed)	95% Confidence Interval Lower
Fatal_rate	Intercept	-19.580	.094	16.562	.163	-47.057
	Inspector_ratio	30.378	-.116	22.294	.118	-12.853
Nonfatal_rate	Intercept	302.481	78.181	717.299	.669	-589.153
	Inspector_ratio	-87.646	-105.619	969.380	.920	-2508.278

Bootstrap for Parameter Estimates

Dependent Variable	Parameter	Bootstrap 95% Confidence Interval Upper
Fatal_rate	Intercept	12.600
	Inspector_ratio	67.650
Nonfatal_rate	Intercept	2105.711
	Inspector_ratio	1109.680

a. Unless otherwise noted, bootstrap results are based on 2000 bootstrap samples

Between-Subjects SSCP Matrix

			Fatal_rate	Nonfatal_rate
Hypothesis	Intercept	Fatal_rate	2.521	-38.939
		Nonfatal_rate	-38.939	601.542
	Inspector_ratio	Fatal_rate	3.427	-9.889
		Nonfatal_rate	-9.889	28.531
Error		Fatal_rate	8.308	302.916
		Nonfatal_rate	302.916	13900.554

Based on Type III Sum of Squares

Residual SSCP Matrix

		Fatal_rate	Nonfatal_rate
Sum-of-Squares and Cross-Products	Fatal_rate	8.308	302.916
	Nonfatal_rate	302.916	13900.554
Covariance	Fatal_rate	1.385	50.486
	Nonfatal_rate	50.486	2316.759
Correlation	Fatal_rate	1.000	.891
	Nonfatal_rate	.891	1.000

Based on Type III Sum of Squares

Lack of Fit

Multivariate Tests

Dependent Variables		Value	F	Hypothesis df	Error df
Fatal_rate, Nonfatal_rate	Pillai's Trace	.[a]	.	.	.
	Wilks' Lambda	.[a]	.	.	.
	Hotelling's Trace	.[a]	.	.	.
	Roy's Largest Root	.[a]	.	.	.
Fatal_rate	Pillai's Trace	.[a]	.	.	.
	Wilks' Lambda	.[a]	.	.	.
	Hotelling's Trace	.[a]	.	.	.
	Roy's Largest Root	.[a]	.	.	.
Nonfatal_rate	Pillai's Trace	.[a]	.	.	.
	Wilks' Lambda	.[a]	.	.	.
	Hotelling's Trace	.[a]	.	.	.
	Roy's Largest Root	.[a]	.	.	.

Multivariate Tests

Dependent Variables		Sig.	Partial Eta Squared	Noncent. Parameter
Fatal_rate, Nonfatal_rate	Pillai's Trace	.	.	.
	Wilks' Lambda	.	.	.
	Hotelling's Trace	.	.	.
	Roy's Largest Root	.	.	.
Fatal_rate	Pillai's Trace	.	.	.
	Wilks' Lambda	.	.	.
	Hotelling's Trace	.	.	.
	Roy's Largest Root	.	.	.
Nonfatal_rate	Pillai's Trace	.	.	.
	Wilks' Lambda	.	.	.
	Hotelling's Trace	.	.	.
	Roy's Largest Root	.	.	.

Multivariate Tests

Dependent Variables		Observed Power[b]
Fatal_rate, Nonfatal_rate	Pillai's Trace	.
	Wilks' Lambda	.
	Hotelling's Trace	.
	Roy's Largest Root	.
Fatal_rate	Pillai's Trace	.
	Wilks' Lambda	.
	Hotelling's Trace	.
	Roy's Largest Root	.
Nonfatal_rate	Pillai's Trace	.
	Wilks' Lambda	.
	Hotelling's Trace	.
	Roy's Largest Root	.

a. The pure error sub-matrix corresponding to the selected dependent variables is a zero matrix.
b. Computed using alpha = .05

Univariate Tests

Dependent Variable	Source	Sum of Squares	df	Mean Square	F	Sig.
Fatal_rate	Lack of Fit	8.308	6	1.385	.	.
	Pure Error	.000	0	.		
Nonfatal_rate	Lack of Fit	13900.554	6	2316.759	.	.
	Pure Error	.000	0	.		

Univariate Tests

Dependent Variable	Source	Partial Eta Squared	Noncent. Parameter	Observed Power[a]
Fatal_rate	Lack of Fit	1.000	.	.
	Pure Error			
Nonfatal_rate	Lack of Fit	1.000	.	.
	Pure Error			

a. Computed using alpha = .05

SSCP Matrix

		Fatal_rate	Nonfatal_rate
Lack of Fit	Fatal_rate	8.308	302.916
	Nonfatal_rate	302.916	13900.554
Pure Error	Fatal_rate	.000	.000
	Nonfatal_rate	.000	.000

Estimated Marginal Means

Grand Mean

Dependent Variable	Mean	Std. Error	95% Confidence Interval Lower Bound	95% Confidence Interval Upper Bound
Fatal_rate	3.242[a]	.416	2.225	4.260
Nonfatal_rate	236.633[a]	17.017	194.992	278.273

a. Covariates appearing in the model are evaluated at the following values: Inspector_ratio = .751300.

Descriptive statistics with log-transformation of dependent variables (PP. 25-33)

Explore

Notes

Output Created		24-FEB-2026 09:05:42
Comments		
Input	Data	E:\Malaysia - Feb 24\Malaysia - Descriptive and multivariate regression.sav
	Active Dataset	DataSet2
	Filter	<none>
	Weight	<none>
	Split File	<none>
	N of Rows in Working Data File	8
Missing Value Handling	Definition of Missing	User-defined missing values for dependent variables are treated as missing.
	Cases Used	Statistics are based on cases with no missing values for any dependent variable or factor used.
Syntax		EXAMINE VARIABLES=Inspector_ratio Log_fatalrate Log_nonfatalrate<br>/PLOT BOXPLOT HISTOGRAM NPPLOT<br>/COMPARE GROUPS<br>/STATISTICS DESCRIPTIVES EXTREME<br>/CINTERVAL 95<br>/MISSING LISTWISE<br>/NOTOTAL.
Resources	Processor Time	00:00:01.66
	Elapsed Time	00:00:01.31

Case Processing Summary

	Cases					
	Valid		Missing		Total	
	N	Percent	N	Percent	N	Percent
Inspector_ratio	8	100.0%	0	0.0%	8	100.0%
Log_fatalrate	8	100.0%	0	0.0%	8	100.0%
Log_nonfatalrate	8	100.0%	0	0.0%	8	100.0%

Descriptives

			Statistic	Std. Error
Inspector_ratio	Mean		.751300	.0081439
	95% Confidence Interval for Mean	Lower Bound	.732043	
		Upper Bound	.770557	
	5% Trimmed Mean		.751217	
	Median		.748150	
	Variance		.001	
	Std. Deviation		.0230344	
	Minimum		.7147	

	Maximum		.7894	
	Range		.0747	
	Interquartile Range		.0312	
	Skewness		.165	.752
	Kurtosis		.144	1.481
Log_fatalrate	Mean		.4795	.06276
	95% Confidence Interval for Mean	Lower Bound	.3311	
		Upper Bound	.6279	
	5% Trimmed Mean		.4776	
	Median		.4548	
	Variance		.032	
	Std. Deviation		.17751	
	Minimum		.31	
	Maximum		.68	
	Range		.38	
	Interquartile Range		.35	
	Skewness		.123	.752
	Kurtosis		-2.520	1.481
Log_nonfatalrate	Mean		2.3659	.03340
	95% Confidence Interval for Mean	Lower Bound	2.2869	
		Upper Bound	2.4449	
	5% Trimmed Mean		2.3729	
	Median		2.3800	
	Variance		.009	
	Std. Deviation		.09448	
	Minimum		2.16	
	Maximum		2.45	
	Range		.30	
	Interquartile Range		.09	
	Skewness		-1.823	.752
	Kurtosis		4.015	1.481

Extreme Values[a]

			Case Number	Value
Inspector_ratio	Highest	1	1	.7894
		2	6	.7715
		3	2	.7615
		4	3	.7536
	Lowest	1	8	.7147
		2	4	.7371
		3	7	.7399
		4	5	.7427
Log_fatalrate	Highest	1	1	.68
		2	2	.68
		3	3	.62
		4	4	.58
	Lowest	1	6	.31
		2	8	.31
		3	7	.32
		4	5	.33

Log_nonfatalrate	Highest	1	2	2.45
		2	1	2.44
		3	4	2.43
		4	8	2.39
	Lowest	1	6	2.16
		2	5	2.34
		3	7	2.35
		4	3	2.37

a. The requested number of extreme values exceeds the number of data points. A smaller number of extremes is displayed.

Tests of Normality

	Kolmogorov-Smirnov[a]			Shapiro-Wilk		
	Statistic	df	Sig.	Statistic	df	Sig.
Inspector_ratio	.146	8	.200*	.985	8	.983
Log_fatalrate	.306	8	.027	.773	8	.014
Log_nonfatalrate	.276	8	.074	.811	8	.038

*. This is a lower bound of the true significance.
a. Lilliefors Significance Correction

Inspector_ratio

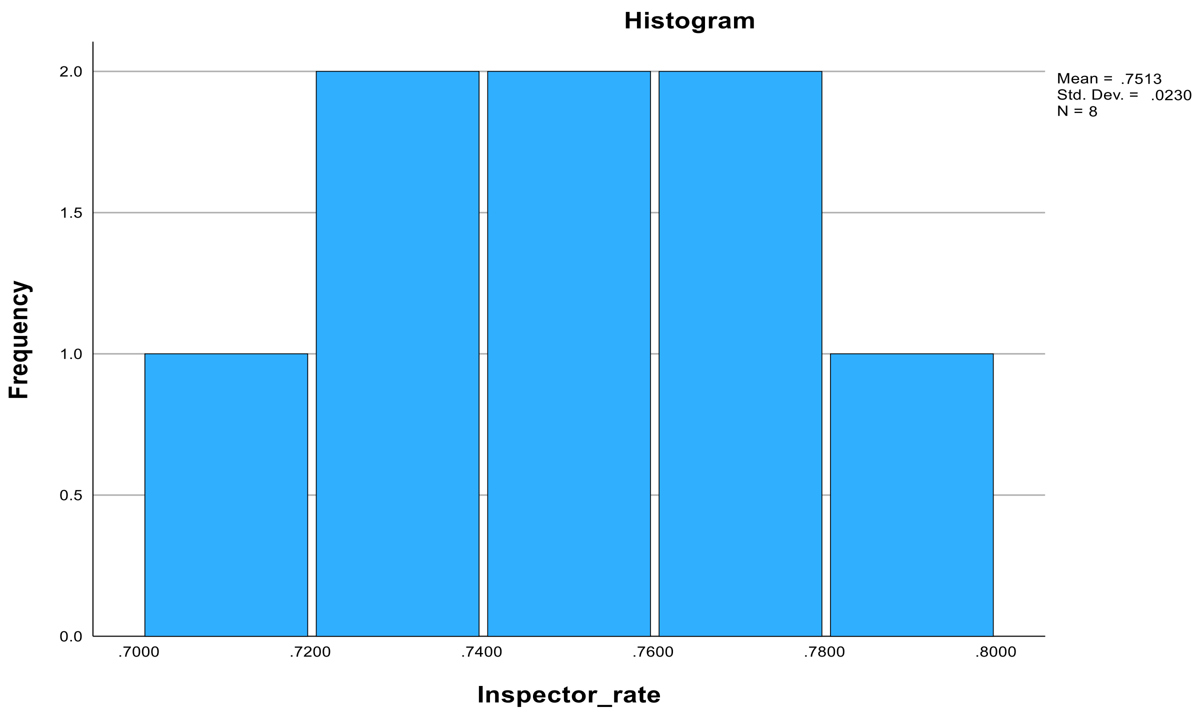

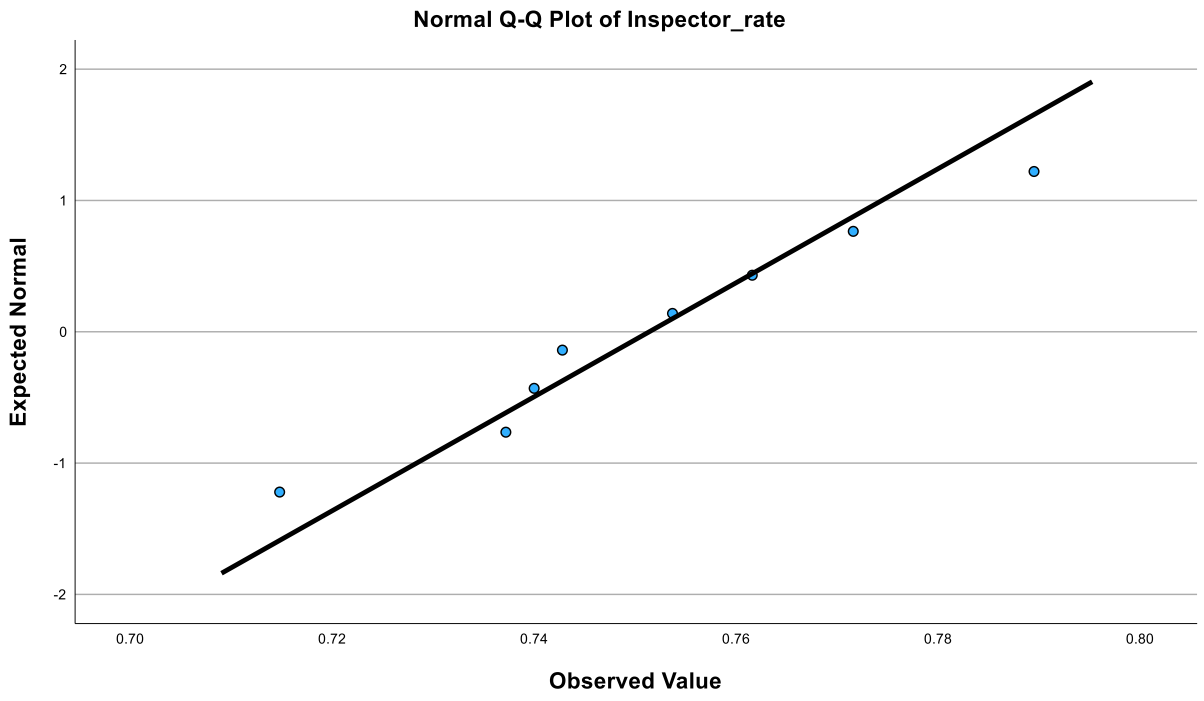
Normal Q-Q Plot of Inspector_rate
Expected Normal
Observed Value
2
1
0
-1
-2
0.70
0.72
0.74
0.76
0.78
0.80

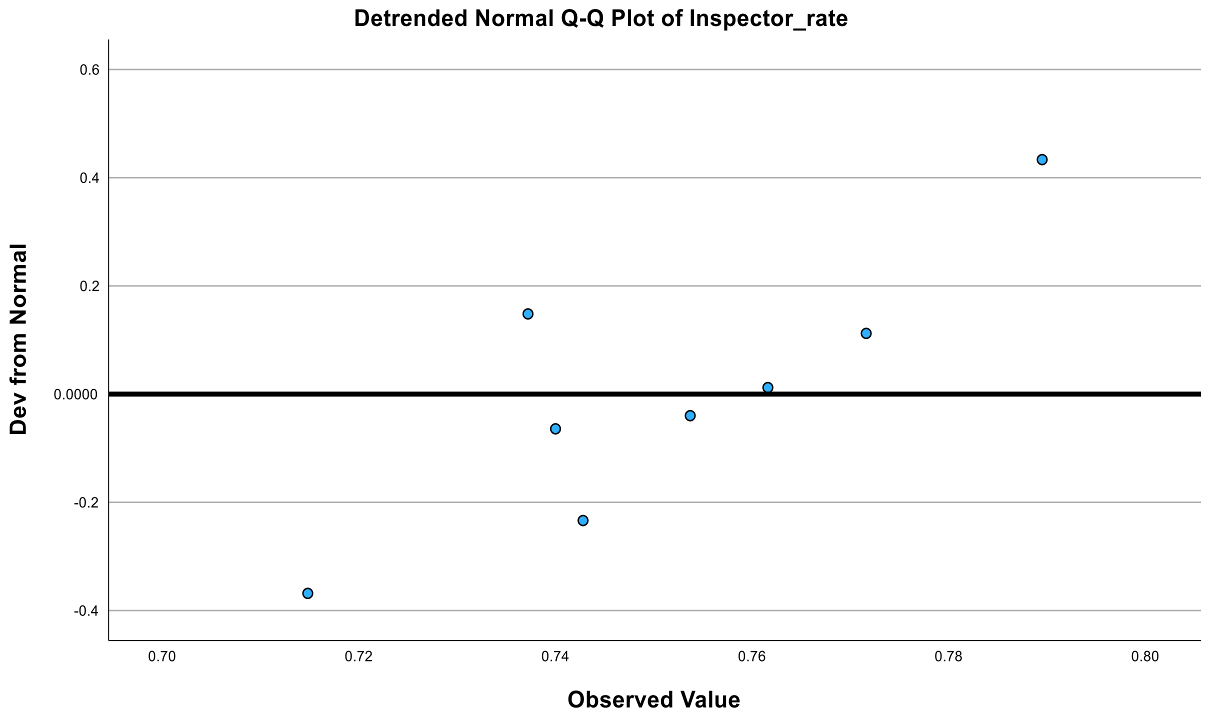
Detrended Normal Q-Q Plot of Inspector_rate
Dev from Normal
Observed Value
0.6
0.4
0.2
0.0000
-0.2
-0.4
0.70
0.72
0.74
0.76
0.78
0.80

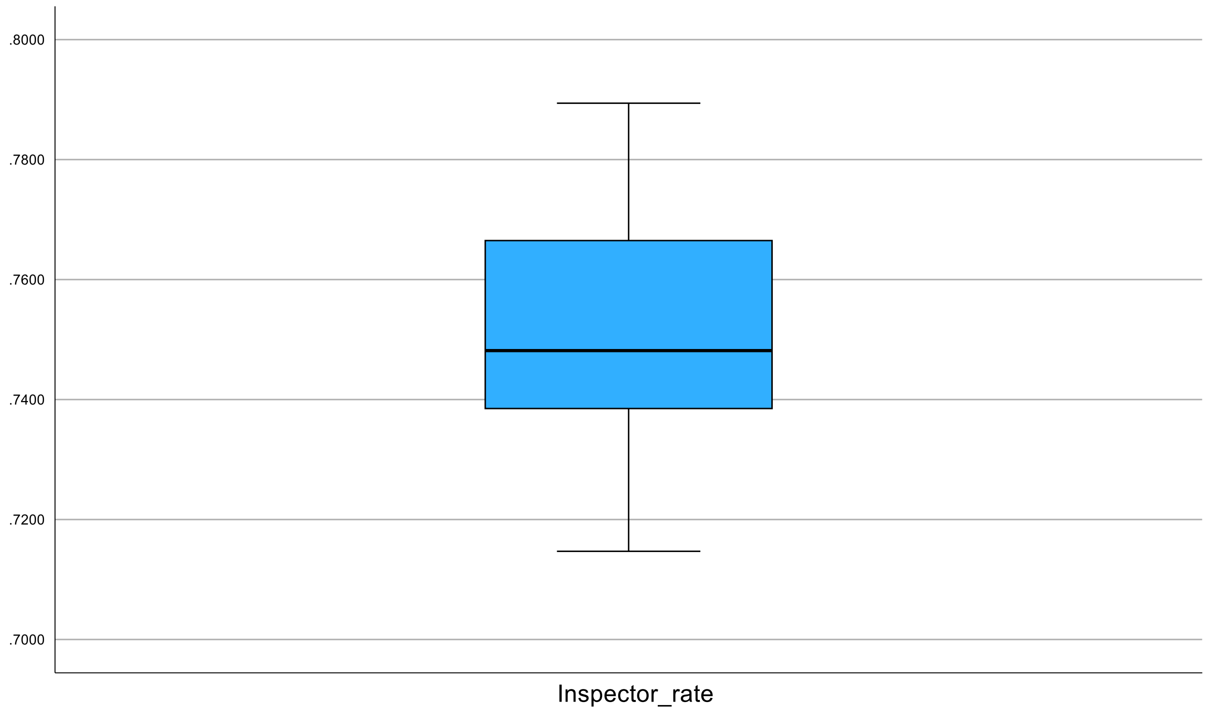

Log_fatalrate

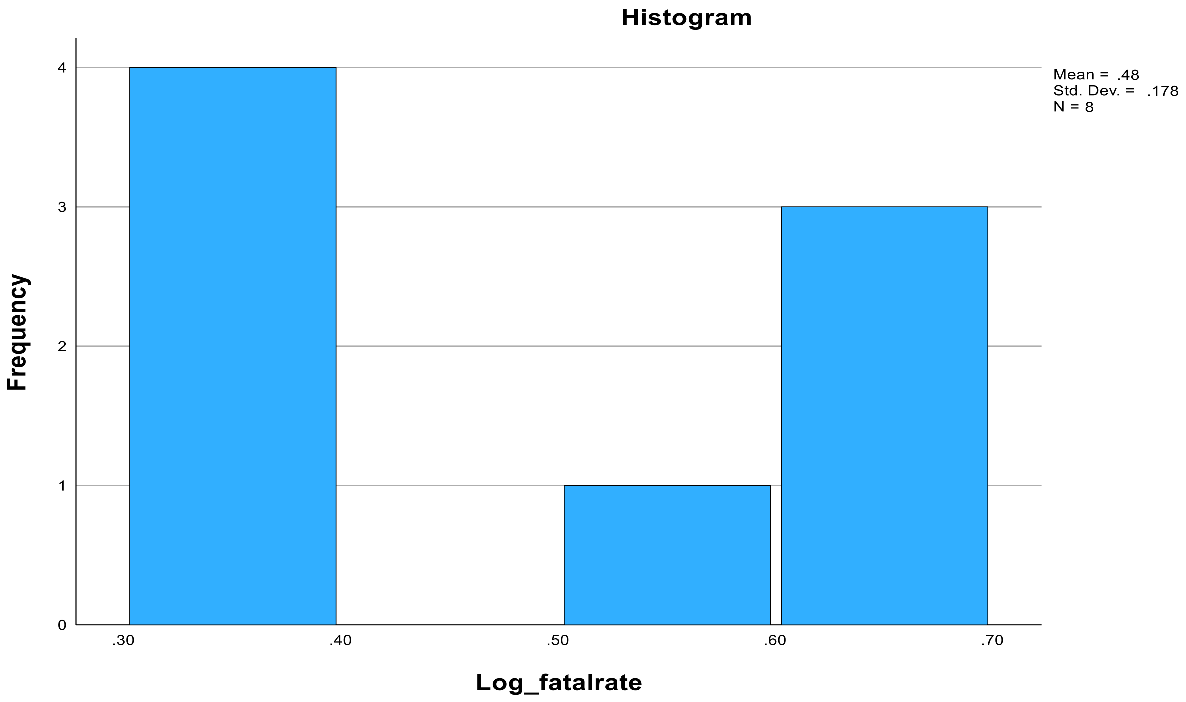

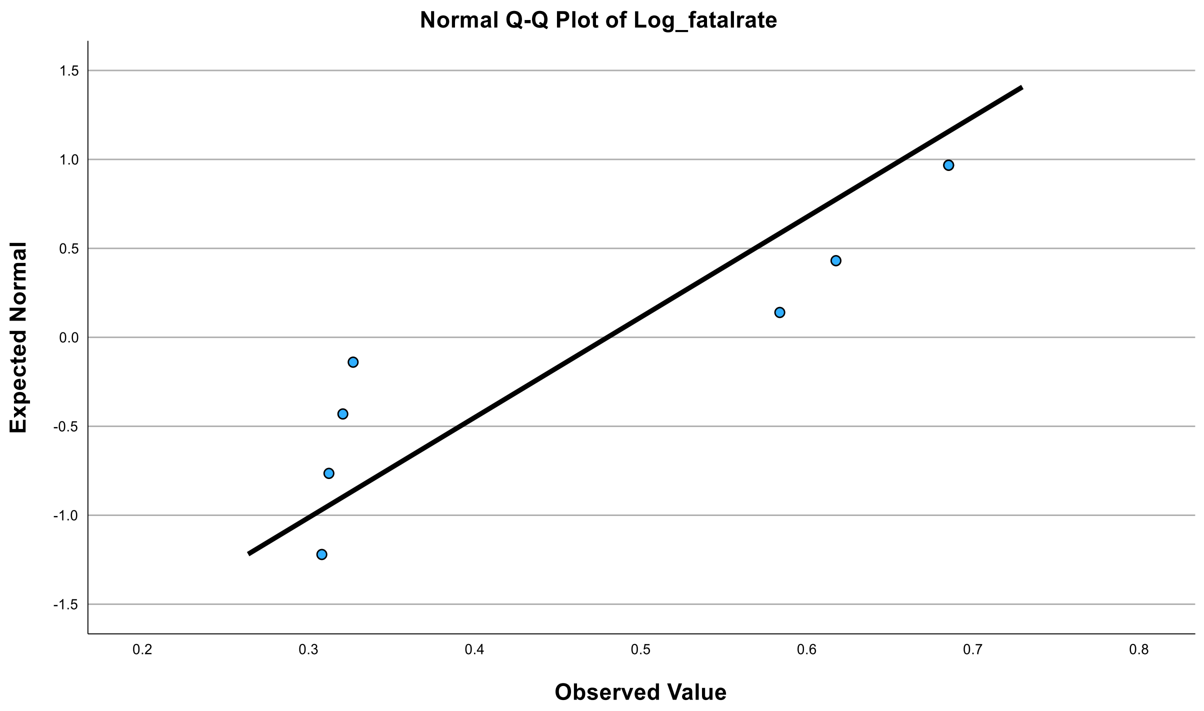
Normal Q-Q Plot of Log_fatalrate
Expected Normal
Observed Value
1.5
1.0
0.5
0.0
-0.5
-1.0
-1.5
0.2
0.3
0.4
0.5
0.6
0.7
0.8

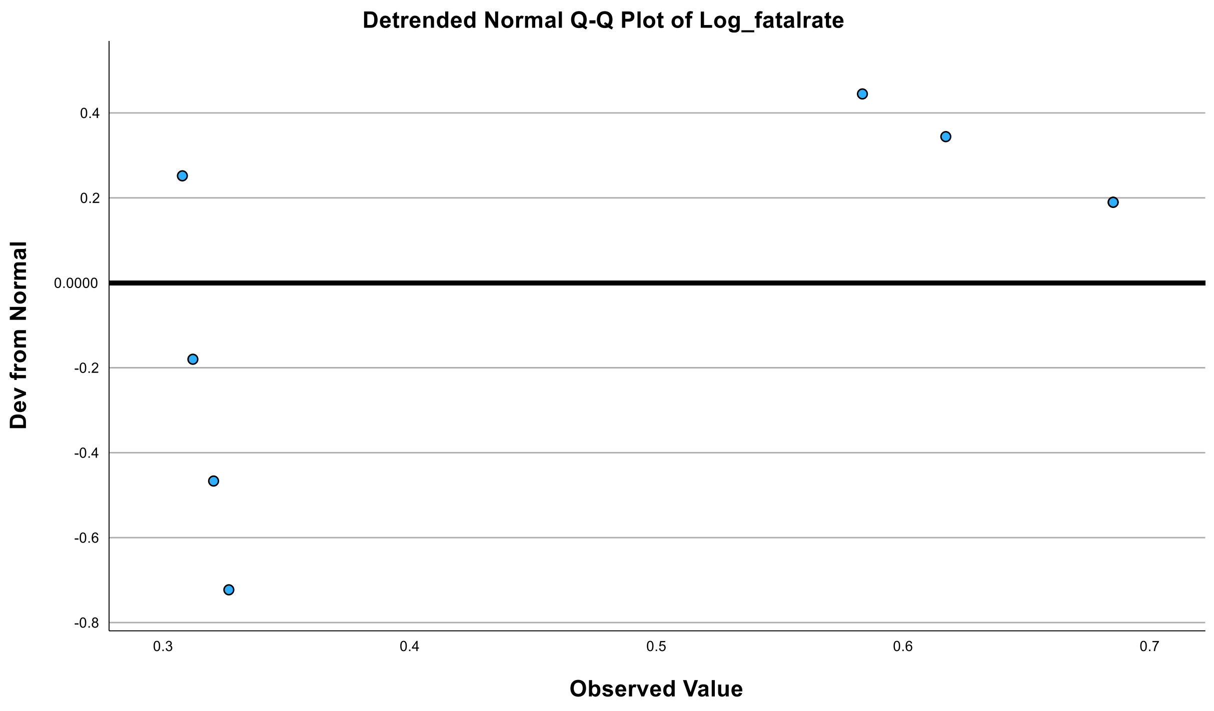
Detrended Normal Q-Q Plot of Log_fatalrate
Dev from Normal
Observed Value
0.4
0.2
0.0000
-0.2
-0.4
-0.6
-0.8
0.3
0.4
0.5
0.6
0.7

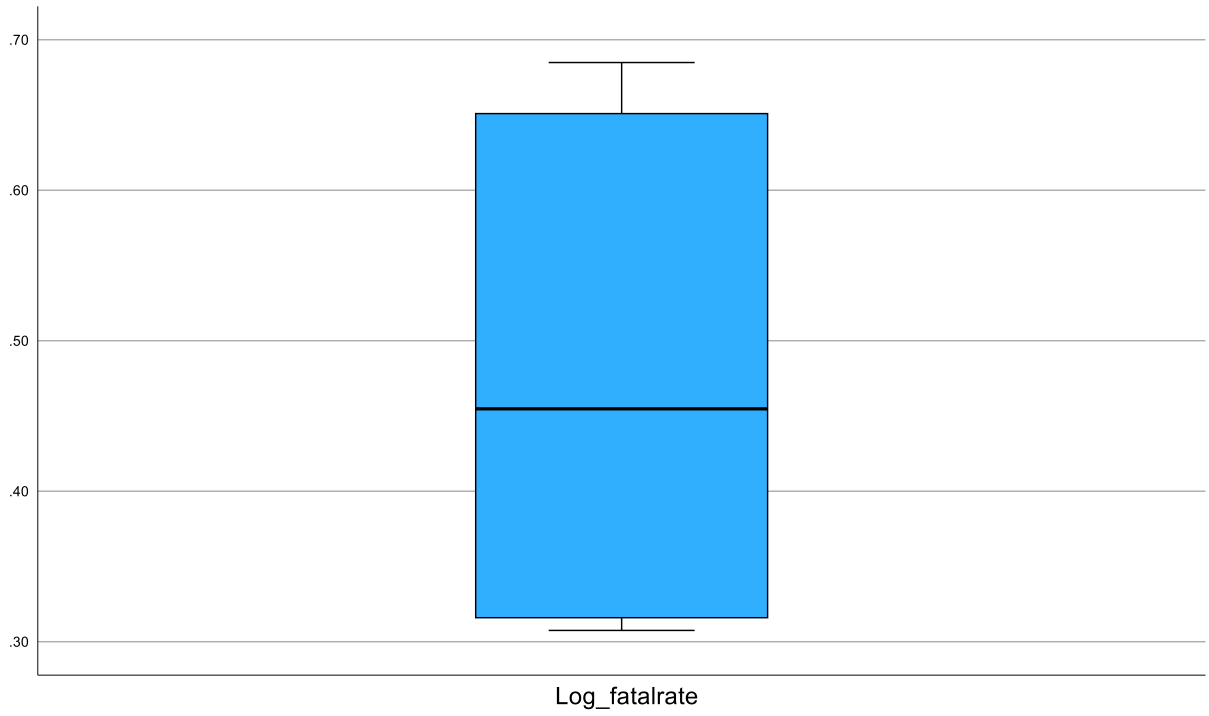

Log_nonfatalrate

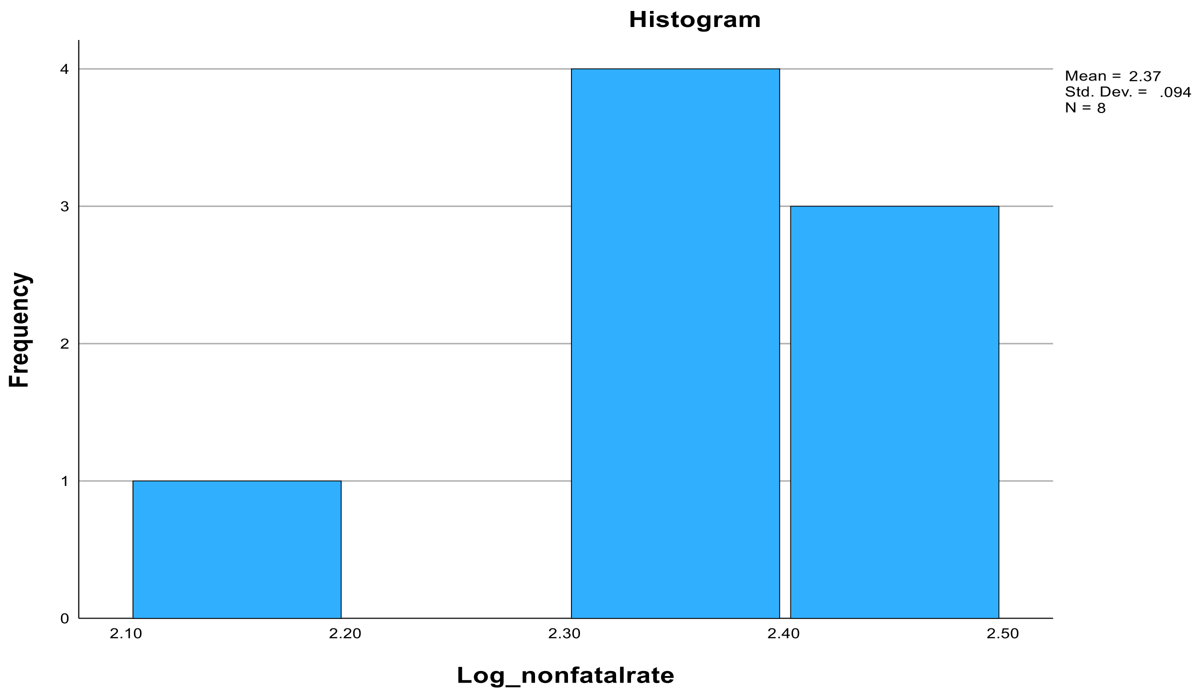

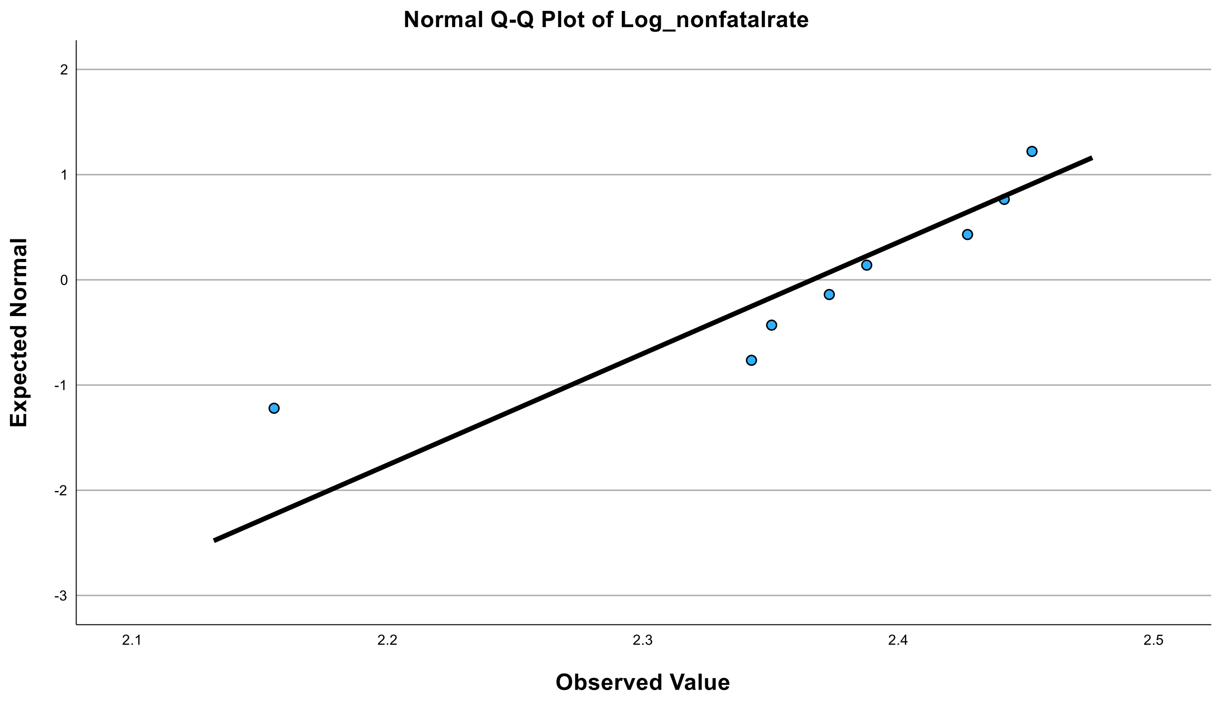
Normal Q-Q Plot of Log_nonfatalrate
Expected Normal
Observed Value
2
1
0
-1
-2
-3
2.1
2.2
2.3
2.4
2.5

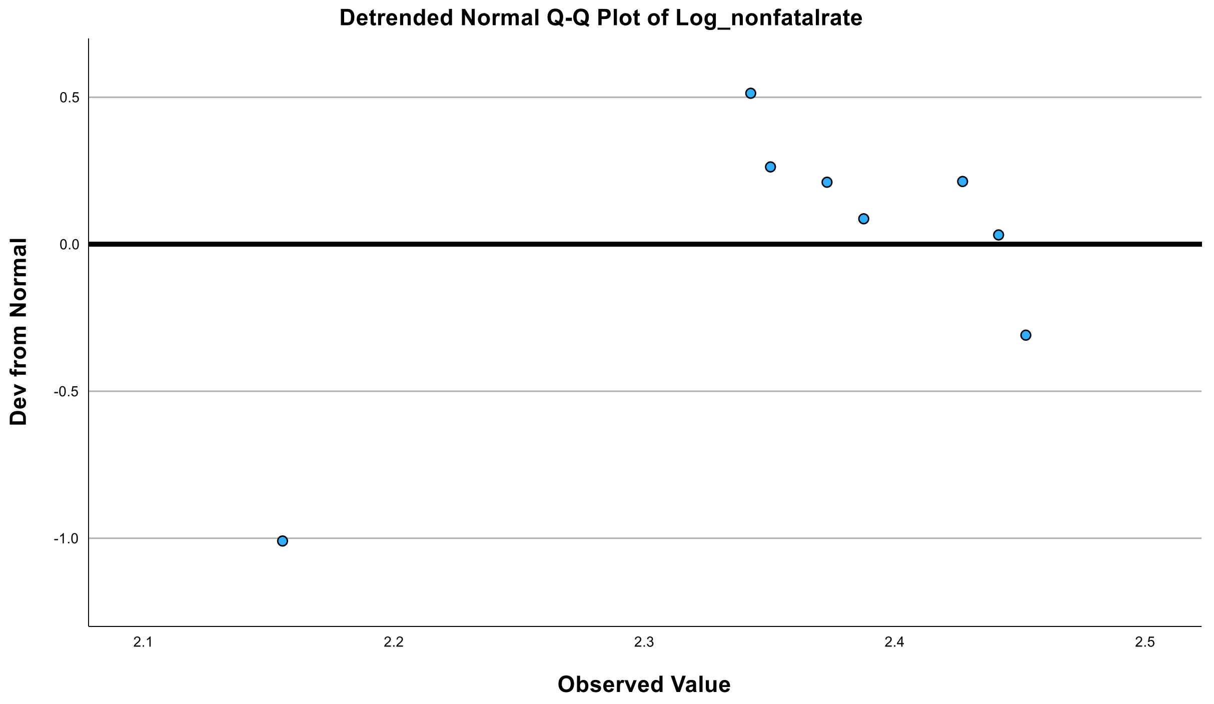
Detrended Normal Q-Q Plot of Log_nonfatalrate
Dev from Normal
Observed Value
0.5
0.0
-0.5
-1.0
2.1
2.2
2.3
2.4
2.5

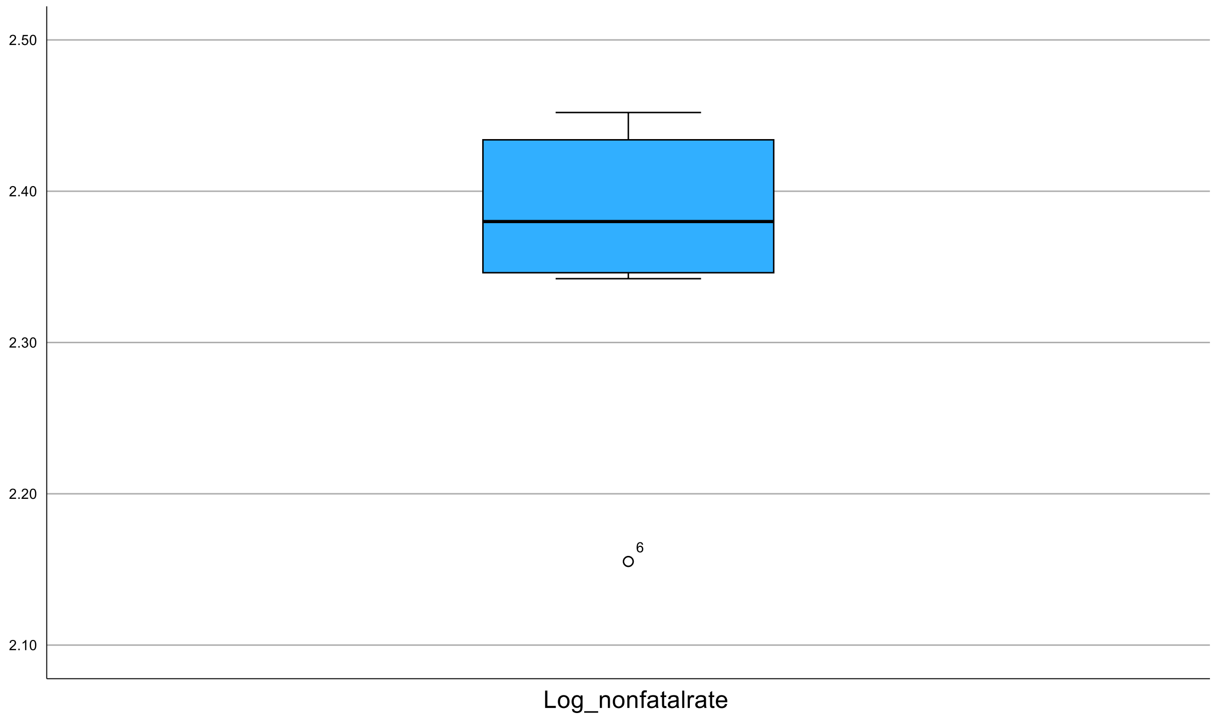
2.50
2.40
2.30
2.20
2.10
6
Log_nonfatalrate

Log-general linear multivariate regression with bootstrap-2000 with Confidence Intervals of 95% (PP. 34-40)

Bootstrap

Notes

Output Created		24-FEB-2026 09:06:59
Comments		
Input	Data	E:\Malaysia - Feb 24\Malaysia - Descriptive and multivariate regression.sav
	Active Dataset	DataSet2
	Filter	<none>
	Weight	<none>
	Split File	<none>
	N of Rows in Working Data File	8
Syntax		BOOTSTRAP<br>/SAMPLING METHOD=SIMPLE<br>/VARIABLES TARGET=Log_fatalrate Log_nonfatalrate INPUT= Inspector_ratio<br>/CRITERIA CILEVEL=95 CITYPE=PERCENTILE NSAMPLES=2000<br>/MISSING USERMISSING=EXCLUDE.
Resources	Processor Time	00:00:00.02
	Elapsed Time	00:00:00.02

Bootstrap Specifications

Sampling Method	Simple
Number of Samples	2000
Confidence Interval Level	95.0%
Confidence Interval Type	Percentile

General Linear Model

Notes

Output Created		24-FEB-2026 09:06:59
Comments		
Input	Data	E:\Malaysia - Feb 24\Malaysia - Descriptive and multivariate regression.sav
	Active Dataset	DataSet2
	Filter	<none>
	Weight	<none>
	Split File	<none>
	N of Rows in Working Data File	16008
Missing Value Handling	Definition of Missing	User-defined missing values are treated as missing.

	Cases Used	Statistics are based on all cases with valid data for all variables in the model.
Syntax		GLM Log_fatalrate Log_nonfatalrate WITH Inspector_ratio<br>/METHOD=SSTYPE(3)<br>/INTERCEPT=INCLUDE<br>/SAVE=PRED SEPRED RESID ZRESID COOK LEVER<br>/EMMEANS=TABLES(OVERALL) WITH(Inspector_ratio=MEAN)<br>/PRINT=DESCRIPTIVE ETASQ OPOWER PARAMETER TEST(SSCP) RSSCP HOMOGENEITY LOF<br>/PLOT=SPREADLEVEL RESIDUALS<br>/CRITERIA=ALPHA(.05)<br>/DESIGN=Inspector_ratio.
Resources	Processor Time	00:00:24.89
	Elapsed Time	00:00:24.99

Warnings

The HOMOGENEITY specification in the PRINT subcommand will be ignored because there are no between-subjects factors.
The SPREADLEVEL specification has been found in the PLOT subcommand, but the model includes no factors. The spread versus level plot will not be produced.
This command is trying to save new variables while bootstrapping is in effect. The new variables will be lost.<br>Execution of this command stops.

Descriptive Statistics

	Mean	Std. Deviation	N
Log_fatalrate	.4795	.17751	8
Log_nonfatalrate	2.3659	.09448	8

Bartlett's Test of Sphericity[a]

Likelihood Ratio	.002
Approx. Chi-Square	7.785
df	2
Sig.	.020

Tests the null hypothesis that the residual covariance matrix is proportional to an identity matrix.[a]

a. Design: Intercept + Inspector_ratio

Multivariate Tests[a]

Effect		Value	F	Hypothesis df	Error df	Sig.
Intercept	Pillai's Trace	.871	16.867[b]	2.000	5.000	.006
	Wilks' Lambda	.129	16.867[b]	2.000	5.000	.006
	Hotelling's Trace	6.747	16.867[b]	2.000	5.000	.006
	Roy's Largest Root	6.747	16.867[b]	2.000	5.000	.006
Inspector_ratio	Pillai's Trace	.644	4.531[b]	2.000	5.000	.075
	Wilks' Lambda	.356	4.531[b]	2.000	5.000	.075
	Hotelling's Trace	1.813	4.531[b]	2.000	5.000	.075
	Roy's Largest Root	1.813	4.531[b]	2.000	5.000	.075

Multivariate Tests[a]

Effect		Partial Eta Squared	Noncent. Parameter	Observed Power[c]
Intercept	Pillai's Trace	.871	33.735	.964
	Wilks' Lambda	.871	33.735	.964
	Hotelling's Trace	.871	33.735	.964
	Roy's Largest Root	.871	33.735	.964
Inspector_ratio	Pillai's Trace	.644	9.063	.493
	Wilks' Lambda	.644	9.063	.493
	Hotelling's Trace	.644	9.063	.493
	Roy's Largest Root	.644	9.063	.493

a. Design: Intercept + Inspector_ratio
b. Exact statistic
c. Computed using alpha = .05

Tests of Between-Subjects Effects

Source	Dependent Variable	Type III Sum of Squares	df	Mean Square	F	Sig.
Corrected Model	Log_fatalrate	.057[a]	1	.057	2.095	.198
	Log_nonfatalrate	.001[b]	1	.001	.081	.786
Intercept	Log_fatalrate	.040	1	.040	1.467	.271
	Log_nonfatalrate	.049	1	.049	4.739	.072
Inspector_ratio	Log_fatalrate	.057	1	.057	2.095	.198
	Log_nonfatalrate	.001	1	.001	.081	.786
Error	Log_fatalrate	.163	6	.027		
	Log_nonfatalrate	.062	6	.010		
Total	Log_fatalrate	2.060	8			
	Log_nonfatalrate	44.843	8			
Corrected Total	Log_fatalrate	.221	7			
	Log_nonfatalrate	.062	7			

Tests of Between-Subjects Effects

Source	Dependent Variable	Partial Eta Squared	Noncent. Parameter	Observed Power[c]
Corrected Model	Log_fatalrate	.259	2.095	.231
	Log_nonfatalrate	.013	.081	.057
Intercept	Log_fatalrate	.196	1.467	.177
	Log_nonfatalrate	.441	4.739	.448
Inspector_ratio	Log_fatalrate	.259	2.095	.231
	Log_nonfatalrate	.013	.081	.057
Error	Log_fatalrate			
	Log_nonfatalrate			
Total	Log_fatalrate			
	Log_nonfatalrate			
Corrected Total	Log_fatalrate			
	Log_nonfatalrate			

a. R Squared = .259 (Adjusted R Squared = .135)
b. R Squared = .013 (Adjusted R Squared = -.151)
c. Computed using alpha = .05

Parameter Estimates

Dependent Variable	Parameter	B	Std. Error	t	Sig.	95% Confidence Interval Lower Bound
Log_fatalrate	Intercept	-2.466	2.036	-1.211	.271	-7.447
	Inspector_ratio	3.920	2.709	1.447	.198	-2.707
Log_nonfatalrate	Intercept	2.721	1.250	2.177	.072	-.338
	Inspector_ratio	-.473	1.663	-.284	.786	-4.543

Parameter Estimates

Dependent Variable	Parameter	95% Confidence Interval Upper Bound	Partial Eta Squared	Noncent. Parameter	Observed Power[a]
Log_fatalrate	Intercept	2.515	.196	1.211	.177
	Inspector_ratio	10.548	.259	1.447	.231
Log_nonfatalrate	Intercept	5.780	.441	2.177	.448
	Inspector_ratio	3.597	.013	.284	.057

a. Computed using alpha = .05

Bootstrap for Parameter Estimates

Dependent Variable	Parameter	B	Bootstrap[a] Bias	Std. Error	Sig. (2-tailed)	95% Confidence Interval Lower
Log_fatalrate	Intercept	-2.466	.100	2.148	.213	-5.942
	Inspector_ratio	3.920	-.128	2.881	.146	-3.160
Log_nonfatalrate	Intercept	2.721	.213	1.569	.252	.959
	Inspector_ratio	-.473	-.285	2.118	.808	-6.308

Bootstrap for Parameter Estimates

Dependent Variable	Parameter	Bootstrap 95% Confidence Interval Upper
Log_fatalrate	Intercept	2.780
	Inspector_ratio	8.577
Log_nonfatalrate	Intercept	7.089
	Inspector_ratio	1.890

a. Unless otherwise noted, bootstrap results are based on 2000 bootstrap samples

Between-Subjects SSCP Matrix

			Log_fatalrate	Log_nonfatalrate
Hypothesis	Intercept	Log_fatalrate	.040	-.044
		Log_nonfatalrate	-.044	.049
	Inspector_ratio	Log_fatalrate	.057	-.007
		Log_nonfatalrate	-.007	.001
Error		Log_fatalrate	.163	.086
		Log_nonfatalrate	.086	.062

Based on Type III Sum of Squares

Residual SSCP Matrix

		Log_fatalrate	Log_nonfatalrate
Sum-of-Squares and Cross-Products	Log_fatalrate	.163	.086
	Log_nonfatalrate	.086	.062
Covariance	Log_fatalrate	.027	.014
	Log_nonfatalrate	.014	.010
Correlation	Log_fatalrate	1.000	.857
	Log_nonfatalrate	.857	1.000

Based on Type III Sum of Squares

Lack of Fit

Multivariate Tests

Dependent Variables		Value	F	Hypothesis df	Error df
Log_fatalrate, Log_nonfatalrate	Pillai's Trace	.[a]	.	.	.
	Wilks' Lambda	.[a]	.	.	.
	Hotelling's Trace	.[a]	.	.	.
	Roy's Largest Root	.[a]	.	.	.
Log_fatalrate	Pillai's Trace	.[a]	.	.	.
	Wilks' Lambda	.[a]	.	.	.
	Hotelling's Trace	.[a]	.	.	.
	Roy's Largest Root	.[a]	.	.	.
Log_nonfatalrate	Pillai's Trace	.[a]	.	.	.
	Wilks' Lambda	.[a]	.	.	.
	Hotelling's Trace	.[a]	.	.	.
	Roy's Largest Root	.[a]	.	.	.

Multivariate Tests

Dependent Variables		Sig.	Partial Eta Squared	Noncent. Parameter
Log_fatalrate, Log_nonfatalrate	Pillai's Trace	.	.	.
	Wilks' Lambda	.	.	.
	Hotelling's Trace	.	.	.
	Roy's Largest Root	.	.	.
Log_fatalrate	Pillai's Trace	.	.	.
	Wilks' Lambda	.	.	.
	Hotelling's Trace	.	.	.
	Roy's Largest Root	.	.	.
Log_nonfatalrate	Pillai's Trace	.	.	.
	Wilks' Lambda	.	.	.
	Hotelling's Trace	.	.	.
	Roy's Largest Root	.	.	.

Multivariate Tests

Dependent Variables		Observed Power[b]
Log_fatalrate, Log_nonfatalrate	Pillai's Trace	.
	Wilks' Lambda	.
	Hotelling's Trace	.
	Roy's Largest Root	.
Log_fatalrate	Pillai's Trace	.
	Wilks' Lambda	.
	Hotelling's Trace	.
	Roy's Largest Root	.
Log_nonfatalrate	Pillai's Trace	.
	Wilks' Lambda	.
	Hotelling's Trace	.
	Roy's Largest Root	.

a. The pure error sub-matrix corresponding to the selected dependent variables is a zero matrix.
b. Computed using alpha = .05

Univariate Tests

Dependent Variable	Source	Sum of Squares	df	Mean Square	F	Sig.
Log_fatalrate	Lack of Fit	.163	6	.027	.	.
	Pure Error	.000	0	.		
Log_nonfatalrate	Lack of Fit	.062	6	.010	.	.
	Pure Error	.000	0	.		

Univariate Tests

Dependent Variable	Source	Partial Eta Squared	Noncent. Parameter	Observed Power[a]
Log_fatalrate	Lack of Fit	1.000	.	.
	Pure Error			
Log_nonfatalrate	Lack of Fit	1.000	.	.
	Pure Error			

a. Computed using alpha = .05

SSCP Matrix

		Log_fatalrate	Log_nonfatalrate
Lack of Fit	Log_fatalrate	.163	.086
	Log_nonfatalrate	.086	.062
Pure Error	Log_fatalrate	.000	.000
	Log_nonfatalrate	.000	.000

Estimated Marginal Means

Grand Mean

Dependent Variable	Mean	Std. Error	95% Confidence Interval Lower Bound	95% Confidence Interval Upper Bound
Log_fatalrate	.479[a]	.058	.337	.622
Log_nonfatalrate	2.366[a]	.036	2.278	2.454

a. Covariates appearing in the model are evaluated at the following values: Inspector_ratio = .751300.

Log-general linear multivariate regression analysis (PP. 41-47)

Notes

Output Created		24-FEB-2026 09:08:23
Comments		
Input	Data	E:\Malaysia - Feb 24\Malaysia - Descriptive and multivariate regression.sav
	Active Dataset	DataSet2
	Filter	<none>
	Weight	<none>
	Split File	<none>
	N of Rows in Working Data File	8
Missing Value Handling	Definition of Missing	User-defined missing values are treated as missing.
	Cases Used	Statistics are based on all cases with valid data for all variables in the model.
Syntax		GLM Log_fatalrate Log_nonfatalrate WITH Inspector_ratio<br>/METHOD=SSTYPE(3)<br>/INTERCEPT=INCLUDE<br>/SAVE=PRED SEPRED RESID ZRESID COOK LEVER<br>/EMMEANS=TABLES(OVERALL) WITH(Inspector_ratio=MEAN)<br>/PRINT=DESCRIPTIVE ETASQ OPOWER PARAMETER TEST(SSCP) RSSCP HOMOGENEITY LOF<br>/PLOT=SPREADLEVEL RESIDUALS<br>/CRITERIA=ALPHA(.05)<br>/DESIGN=Inspector_ratio.
Resources	Processor Time	00:00:00.27
	Elapsed Time	00:00:00.26
Variables Created or Modified	PRE_3	Predicted Value for Log_fatalrate
	PRE_4	Predicted Value for Log_nonfatalrate
	SEP_3	Standard Error of Predicted Value for Log_fatalrate
	SEP_4	Standard Error of Predicted Value for Log_nonfatalrate
	RES_3	Residual for Log_fatalrate
	RES_4	Residual for Log_nonfatalrate
	ZRE_3	Standardized Residual for Log_fatalrate
	ZRE_4	Standardized Residual for Log_nonfatalrate
	COO_3	Cook's Distance for Log_fatalrate
	COO_4	Cook's Distance for Log_nonfatalrate
	LEV_3	Uncentered Leverage Value for Log_fatalrate
	LEV_4	Uncentered Leverage Value for Log_nonfatalrate

Warnings

The HOMOGENEITY specification in the PRINT subcommand will be ignored because there are no between-subjects factors.
The SPREADLEVEL specification has been found in the PLOT subcommand, but the model includes no factors. The spread versus level plot will not be produced.

Descriptive Statistics

	Mean	Std. Deviation	N
Log_fatalrate	.4795	.17751	8
Log_nonfatalrate	2.3659	.09448	8

Bartlett's Test of Sphericity[a]

Likelihood Ratio	.002
Approx. Chi-Square	7.785
df	2
Sig.	.020

Tests the null hypothesis that the residual covariance matrix is proportional to an identity matrix.[a]
a. Design: Intercept + Inspector_ratio

Multivariate Tests[a]

Effect		Value	F	Hypothesis df	Error df	Sig.
Intercept	Pillai's Trace	.871	16.867[b]	2.000	5.000	.006
	Wilks' Lambda	.129	16.867[b]	2.000	5.000	.006
	Hotelling's Trace	6.747	16.867[b]	2.000	5.000	.006
	Roy's Largest Root	6.747	16.867[b]	2.000	5.000	.006
Inspector_ratio	Pillai's Trace	.644	4.531[b]	2.000	5.000	.075
	Wilks' Lambda	.356	4.531[b]	2.000	5.000	.075
	Hotelling's Trace	1.813	4.531[b]	2.000	5.000	.075
	Roy's Largest Root	1.813	4.531[b]	2.000	5.000	.075

Multivariate Tests[a]

Effect		Partial Eta Squared	Noncent. Parameter	Observed Power[c]
Intercept	Pillai's Trace	.871	33.735	.964
	Wilks' Lambda	.871	33.735	.964
	Hotelling's Trace	.871	33.735	.964
	Roy's Largest Root	.871	33.735	.964
Inspector_ratio	Pillai's Trace	.644	9.063	.493
	Wilks' Lambda	.644	9.063	.493
	Hotelling's Trace	.644	9.063	.493
	Roy's Largest Root	.644	9.063	.493

a. Design: Intercept + Inspector_ratio
b. Exact statistic
c. Computed using alpha = .05

Tests of Between-Subjects Effects

Source	Dependent Variable	Type III Sum of Squares	df	Mean Square	F	Sig.
Corrected Model	Log_fatalrate	.057[a]	1	.057	2.095	.198
	Log_nonfatalrate	.001[b]	1	.001	.081	.786
Intercept	Log_fatalrate	.040	1	.040	1.467	.271
	Log_nonfatalrate	.049	1	.049	4.739	.072
Inspector_ratio	Log_fatalrate	.057	1	.057	2.095	.198
	Log_nonfatalrate	.001	1	.001	.081	.786
Error	Log_fatalrate	.163	6	.027		
	Log_nonfatalrate	.062	6	.010		
Total	Log_fatalrate	2.060	8			
	Log_nonfatalrate	44.843	8			
Corrected Total	Log_fatalrate	.221	7			
	Log_nonfatalrate	.062	7			

Tests of Between-Subjects Effects

Source	Dependent Variable	Partial Eta Squared	Noncent. Parameter	Observed Power[c]
Corrected Model	Log_fatalrate	.259	2.095	.231
	Log_nonfatalrate	.013	.081	.057
Intercept	Log_fatalrate	.196	1.467	.177
	Log_nonfatalrate	.441	4.739	.448
Inspector_ratio	Log_fatalrate	.259	2.095	.231
	Log_nonfatalrate	.013	.081	.057
Error	Log_fatalrate			
	Log_nonfatalrate			
Total	Log_fatalrate			
	Log_nonfatalrate			
Corrected Total	Log_fatalrate			
	Log_nonfatalrate			

a. R Squared = .259 (Adjusted R Squared = .135)
b. R Squared = .013 (Adjusted R Squared = -.151)
c. Computed using alpha = .05

Parameter Estimates

Dependent Variable	Parameter	B	Std. Error	t	Sig.	95% Confidence Interval Lower Bound
Log_fatalrate	Intercept	-2.466	2.036	-1.211	.271	-7.447
	Inspector_ratio	3.920	2.709	1.447	.198	-2.707
Log_nonfatalrate	Intercept	2.721	1.250	2.177	.072	-.338
	Inspector_ratio	-.473	1.663	-.284	.786	-4.543

Parameter Estimates

Dependent Variable	Parameter	95% Confidence Interval Upper Bound	Partial Eta Squared	Noncent. Parameter	Observed Power[a]
Log_fatalrate	Intercept	2.515	.196	1.211	.177
	Inspector_ratio	10.548	.259	1.447	.231
Log_nonfatalrate	Intercept	5.780	.441	2.177	.448
	Inspector_ratio	3.597	.013	.284	.057

a. Computed using alpha = .05

Between-Subjects SSCP Matrix

			Log_fatalrate	Log_nonfatalrate
Hypothesis	Intercept	Log_fatalrate	.040	-.044
		Log_nonfatalrate	-.044	.049
	Inspector_ratio	Log_fatalrate	.057	-.007
		Log_nonfatalrate	-.007	.001
Error		Log_fatalrate	.163	.086
		Log_nonfatalrate	.086	.062

Based on Type III Sum of Squares

Residual SSCP Matrix

		Log_fatalrate	Log_nonfatalrate
Sum-of-Squares and Cross-Products	Log_fatalrate	.163	.086
	Log_nonfatalrate	.086	.062
Covariance	Log_fatalrate	.027	.014
	Log_nonfatalrate	.014	.010
Correlation	Log_fatalrate	1.000	.857
	Log_nonfatalrate	.857	1.000

Based on Type III Sum of Squares

Lack of Fit

Multivariate Tests

Dependent Variables		Value	F	Hypothesis df	Error df
Log_fatalrate, Log_nonfatalrate	Pillai's Trace	.[a]	.	.	.
	Wilks' Lambda	.[a]	.	.	.
	Hotelling's Trace	.[a]	.	.	.
	Roy's Largest Root	.[a]	.	.	.
Log_fatalrate	Pillai's Trace	.[a]	.	.	.
	Wilks' Lambda	.[a]	.	.	.
	Hotelling's Trace	.[a]	.	.	.
	Roy's Largest Root	.[a]	.	.	.
Log_nonfatalrate	Pillai's Trace	.[a]	.	.	.
	Wilks' Lambda	.[a]	.	.	.
	Hotelling's Trace	.[a]	.	.	.
	Roy's Largest Root	.[a]	.	.	.

Multivariate Tests

Dependent Variables		Sig.	Partial Eta Squared	Noncent. Parameter
Log_fatalrate, Log_nonfatalrate	Pillai's Trace	.	.	.
	Wilks' Lambda	.	.	.
	Hotelling's Trace	.	.	.
	Roy's Largest Root	.	.	.
Log_fatalrate	Pillai's Trace	.	.	.
	Wilks' Lambda	.	.	.
	Hotelling's Trace	.	.	.
	Roy's Largest Root	.	.	.
Log_nonfatalrate	Pillai's Trace	.	.	.
	Wilks' Lambda	.	.	.
	Hotelling's Trace	.	.	.
	Roy's Largest Root	.	.	.

Multivariate Tests

Dependent Variables		Observed Power[b]
Log_fatalrate, Log_nonfatalrate	Pillai's Trace	.
	Wilks' Lambda	.
	Hotelling's Trace	.
	Roy's Largest Root	.
Log_fatalrate	Pillai's Trace	.
	Wilks' Lambda	.
	Hotelling's Trace	.
	Roy's Largest Root	.
Log_nonfatalrate	Pillai's Trace	.
	Wilks' Lambda	.
	Hotelling's Trace	.
	Roy's Largest Root	.

a. The pure error sub-matrix corresponding to the selected dependent variables is a zero matrix.
b. Computed using alpha = .05

Univariate Tests

Dependent Variable	Source	Sum of Squares	df	Mean Square	F	Sig.
Log_fatalrate	Lack of Fit	.163	6	.027	.	.
	Pure Error	.000	0	.		
Log_nonfatalrate	Lack of Fit	.062	6	.010	.	.
	Pure Error	.000	0	.		

Univariate Tests

Dependent Variable	Source	Partial Eta Squared	Noncent. Parameter	Observed Power[a]
Log_fatalrate	Lack of Fit	1.000	.	.
	Pure Error			
Log_nonfatalrate	Lack of Fit	1.000	.	.
	Pure Error			

a. Computed using alpha = .05

SSCP Matrix

		Log_fatalrate	Log_nonfatalrate
Lack of Fit	Log_fatalrate	.163	.086
	Log_nonfatalrate	.086	.062
Pure Error	Log_fatalrate	.000	.000
	Log_nonfatalrate	.000	.000

Estimated Marginal Means

Grand Mean

Dependent Variable	Mean	Std. Error	95% Confidence Interval Lower Bound	95% Confidence Interval Upper Bound
Log_fatalrate	.479[a]	.058	.337	.622
Log_nonfatalrate	2.366[a]	.036	2.278	2.454

a. Covariates appearing in the model are evaluated at the following values: Inspector_ratio = .751300.

Observed * Predicted * Std. Residual Plots

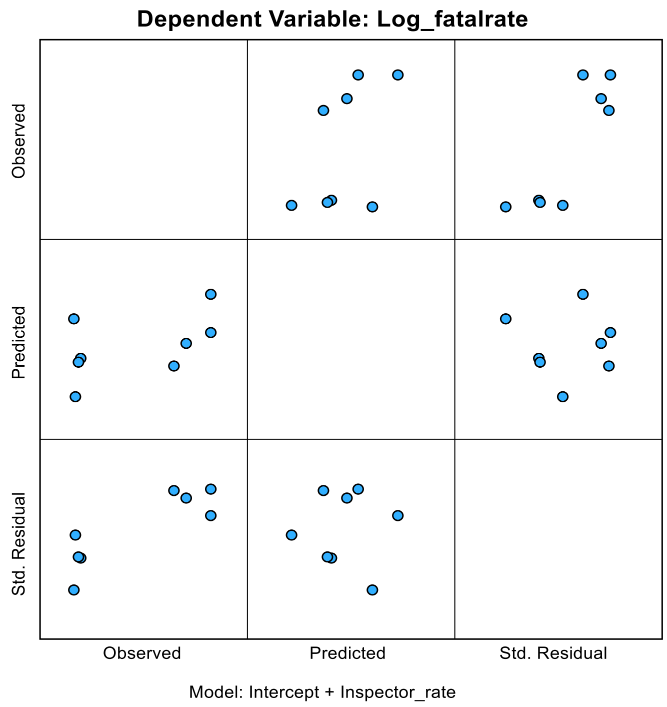

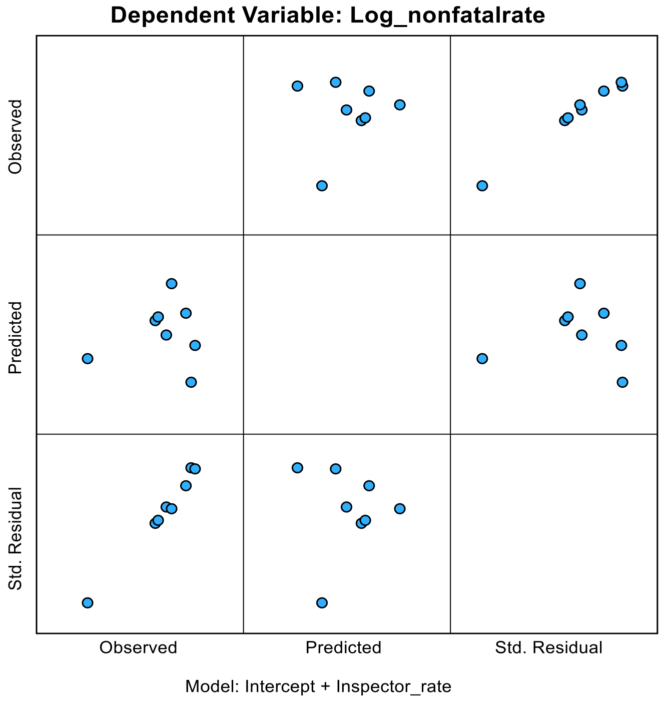

Thus, following analysis have been completed with IBM SPSS 30.0 for Malaysia, as hereunder-

- Descriptive statistics with original data associated with the research variables,
- General linear multivariate regression,
- General linear multivariate regression with bootstrap-2000 with Confidence Intervals of 95%,
- Descriptive statistics with log-transformation of dependent variables,
- Log-general linear multivariate regression with bootstrap-2000 with Confidence Intervals of 95%, and
- Log-general linear multivariate regression analysis.
