# Supplementary material for: OSH inspector ratio to strengthen decent workplace safety and health: a cross-regional trend analysis research
Source: Front Public Health. 2026 Apr 8;14:1806383. doi: 10.3389/fpubh.2026.1806383 (PMC13099828; doi:10.3389/fpubh.2026.1806383)
Supplement: Supplementary file 1 [file Data_Sheet_1.zip › Supplementary document Saskatchewan - Descriptive and multivariate regression analysis.pdf]

Supplementary Material

Saskatchewan – Descriptive and multivariate regression analysis

Saskatchewan is a province of Canada, which is an International Labour Organization (ILO) member nation. IBM SPSS 30.0 software was used to complete descriptive statistics and multivariate regression analysis for Saskatchewan, with longitudinal data for eight different years. There are six major sections in this supplementary material as listed hereunder:

1. Descriptive statistics with original data associated with the research variables (PP. 2-10),
2. General linear multivariate regression (PP. 11-17),
3. General linear multivariate regression with bootstrap-2000 with Confidence Intervals of 95% (PP. 18-24),
4. Descriptive statistics with log-transformation of dependent variables (PP. 25-33),
5. Log-general linear multivariate regression with bootstrap-2000 with Confidence Intervals of 95% (PP. 34-40), and
6. Log-general linear multivariate regression analysis (PP. 41-47).

Note:

- Inspector ratio or inspector_ratio, or inspector_rate = Independent Variable (IV),
- Falta injury rate or fatal_rate = Dependent Variable (DV1), and
- Nonfatal injury rate or nonfatal_rate = Dependent Variable (DV2).

Descriptive statistics with original data associated with the research variables (PP. 2-10)

Explore

Notes

Output Created		24-FEB-2026 08:28:32
Comments		
Input	Active Dataset	DataSet2
	Filter	<none>
	Weight	<none>
	Split File	<none>
	N of Rows in Working Data File	8
Missing Value Handling	Definition of Missing	User-defined missing values for dependent variables are treated as missing.
	Cases Used	Statistics are based on cases with no missing values for any dependent variable or factor used.
Syntax		EXAMINE VARIABLES=Inspector_ratio Fatal_rate Nonfatal_rate<br>/PLOT BOXPLOT HISTOGRAM NPPLOT<br>/COMPARE GROUPS<br>/STATISTICS DESCRIPTIVES EXTREME<br>/CINTERVAL 95<br>/MISSING LISTWISE<br>/NOTOTAL.
Resources	Processor Time	00:00:05.62
	Elapsed Time	00:00:01.90

Case Processing Summary

	Cases					
	Valid		Missing		Total	
	N	Percent	N	Percent	N	Percent
Inspector_ratio	8	100.0%	0	0.0%	8	100.0%
Fatal_rate	8	100.0%	0	0.0%	8	100.0%
Nonfatal_rate	8	100.0%	0	0.0%	8	100.0%

Descriptives

			Statistic	Std. Error
Inspector_ratio	Mean		1.157200	.0234691
	95% Confidence Interval for Mean	Lower Bound	1.101705	
		Upper Bound	1.212695	
	5% Trimmed Mean		1.157500	
	Median		1.138950	
	Variance		.004	
	Std. Deviation		.0663805	
	Minimum		1.0488	
	Maximum		1.2602	
	Range		.2114	
	Interquartile Range		.0906	
	Skewness		.087	.752
	Kurtosis		.029	1.481
Fatal_rate	Mean		9.136250	.6815972
	95% Confidence Interval for Mean	Lower Bound	7.524529	
		Upper Bound	10.747971	
	5% Trimmed Mean		9.027500	
	Median		8.765000	
	Variance		3.717	
	Std. Deviation		1.9278481	
	Minimum		7.2800	
	Maximum		12.9500	
	Range		5.6700	
	Interquartile Range		2.9025	
	Skewness		1.137	.752
	Kurtosis		1.117	1.481
Nonfatal_rate	Mean		4597.113750	199.7784371
	95% Confidence Interval for Mean	Lower Bound	4124.712813	
		Upper Bound	5069.514687	
	5% Trimmed Mean		4589.583611	
	Median		4501.235000	
	Variance		319291.392	
	Std. Deviation		565.0587506	
	Minimum		3902.7200	
	Maximum		5427.0500	
	Range		1524.3300	
	Interquartile Range		1130.6075	
	Skewness		.254	.752
	Kurtosis		-1.251	1.481

Extreme Values[a]

			Case Number	Value
Inspector_ratio	Highest	1	4	1.2602
		2	5	1.2284
		3	6	1.1865
		4	7	1.1482
	Lowest	1	1	1.0488
		2	3	1.1267

		3	8	1.1291
		4	2	1.1297
Fatal_rate	Highest	1	2	12.9500
		2	6	10.5200
		3	3	9.7100
		4	4	9.1700
	Lowest	1	8	7.2800
		2	1	7.2800
		3	7	7.8200
		4	5	8.3600
Nonfatal_rate	Highest	1	2	5427.0500
		2	1	5242.7200
		3	3	4940.2900
		4	5	4551.6400
	Lowest	1	8	3902.7200
		2	7	3942.1800
		3	6	4319.4800
		4	4	4450.8300

a. The requested number of extreme values exceeds the number of data points. A smaller number of extremes is displayed.

Tests of Normality

	Kolmogorov-Smirnov[a]			Shapiro-Wilk		
	Statistic	df	Sig.	Statistic	df	Sig.
Inspector_ratio	.198	8	.200*	.950	8	.711
Fatal_rate	.168	8	.200*	.898	8	.274
Nonfatal_rate	.157	8	.200*	.938	8	.594

*. This is a lower bound of the true significance.

a. Lilliefors Significance Correction

Inspector_ratio

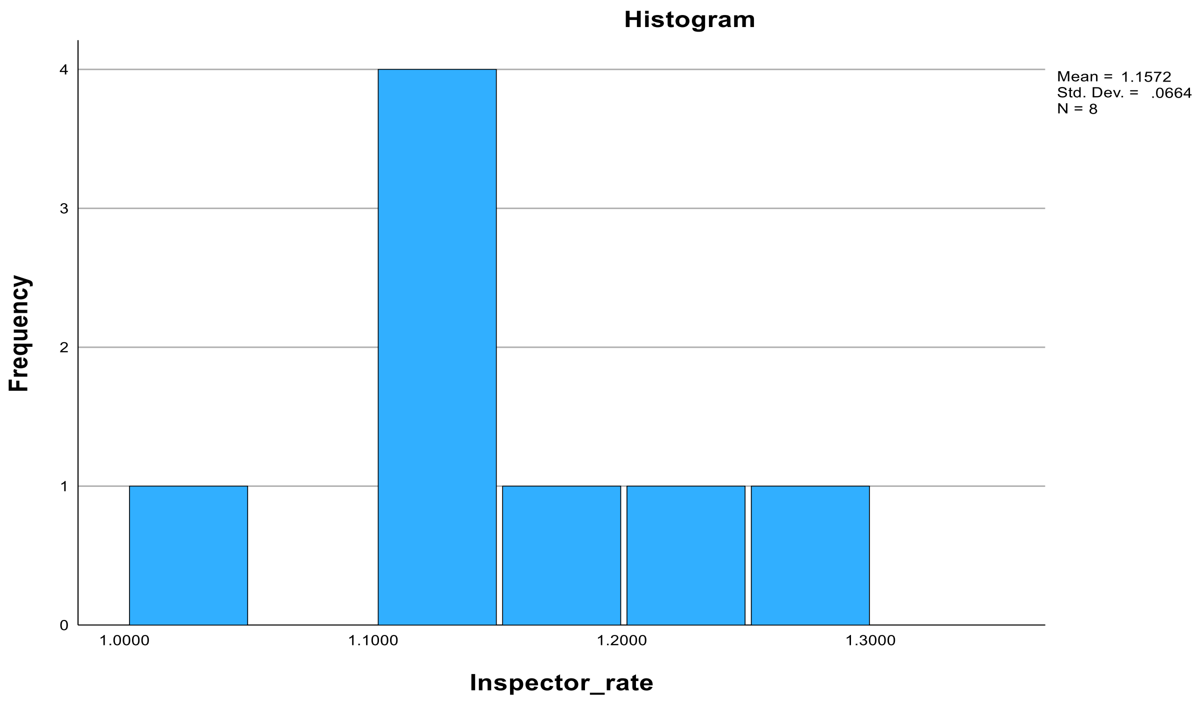

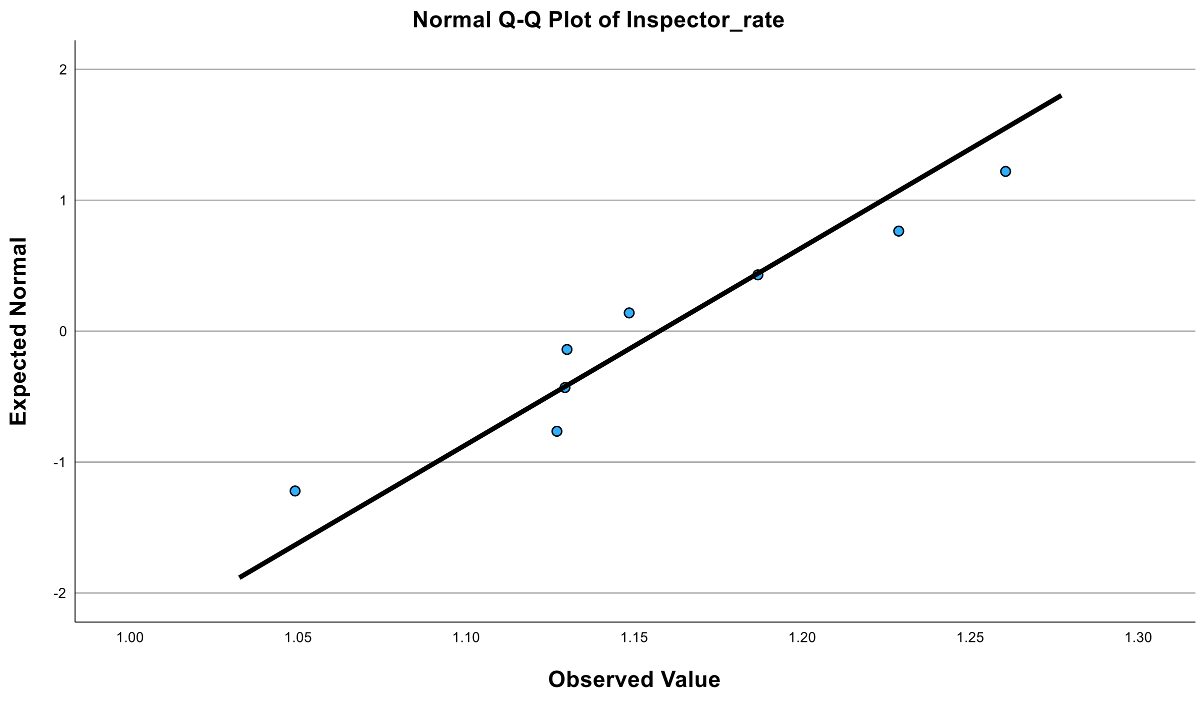

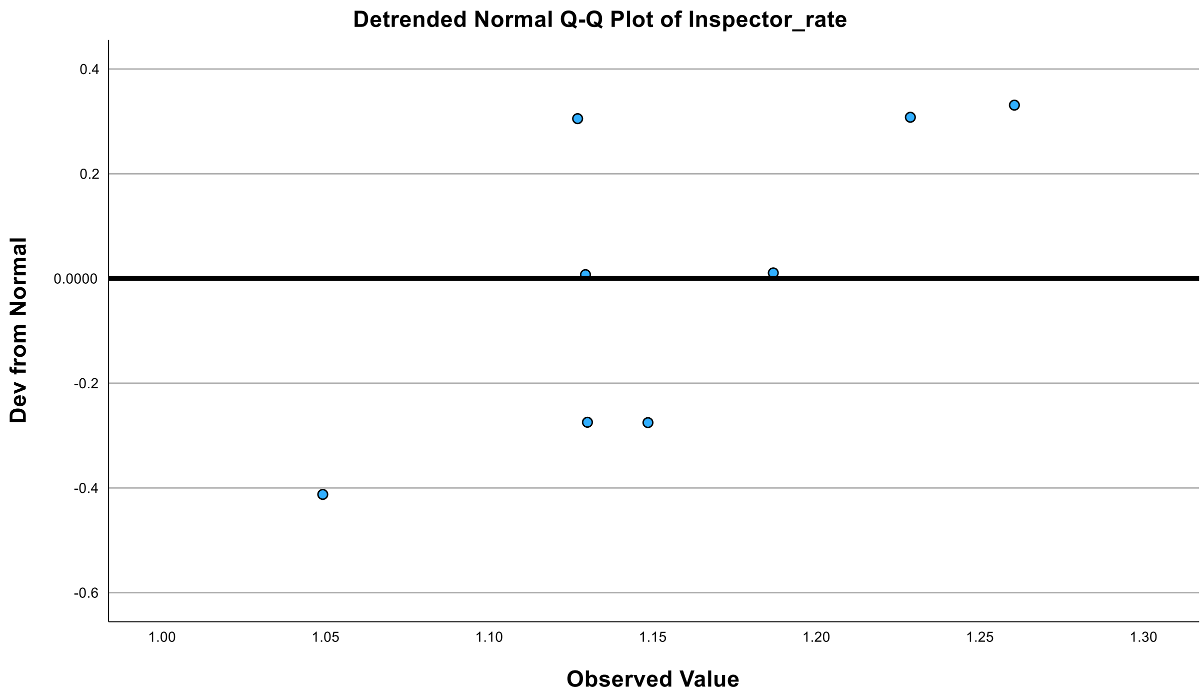
Detrended Normal Q-Q Plot of Inspector_rate
Dev from Normal
0.4
0.2
0.0000
-0.2
-0.4
-0.6
1.00
1.05
1.10
1.15
1.20
1.25
1.30
Observed Value

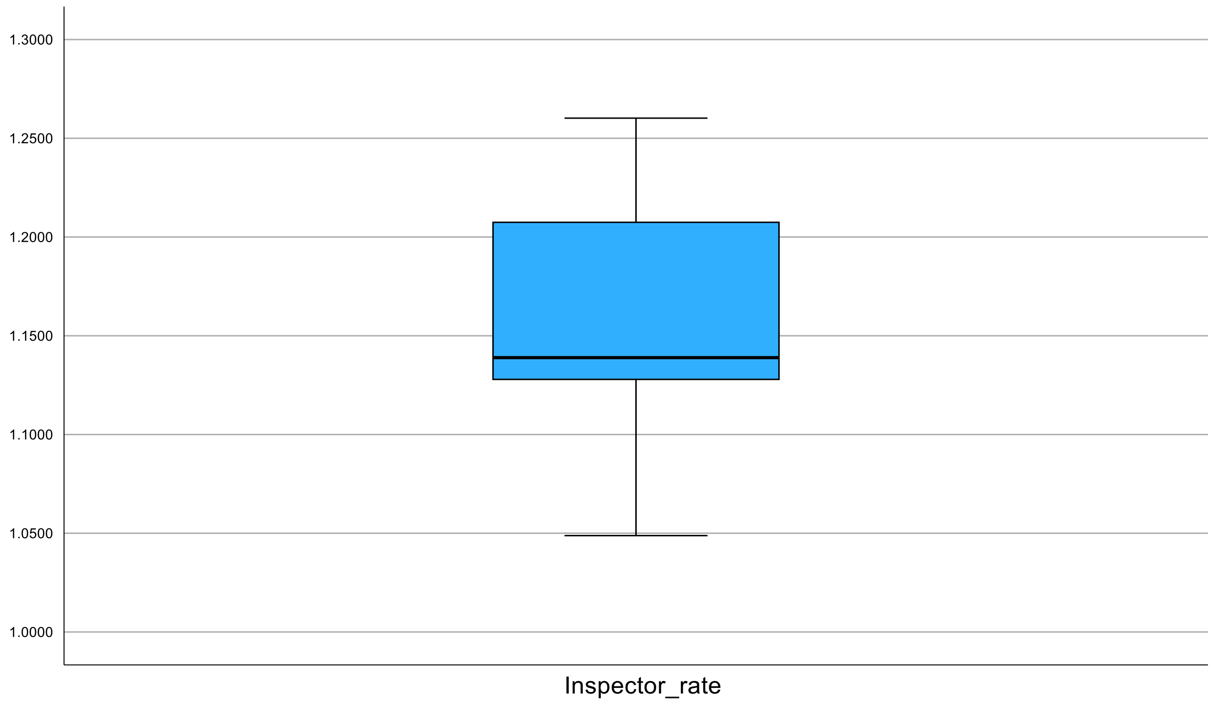
1.3000
1.2500
1.2000
1.1500
1.1000
1.0500
1.0000
Inspector_rate

Fatal_rate

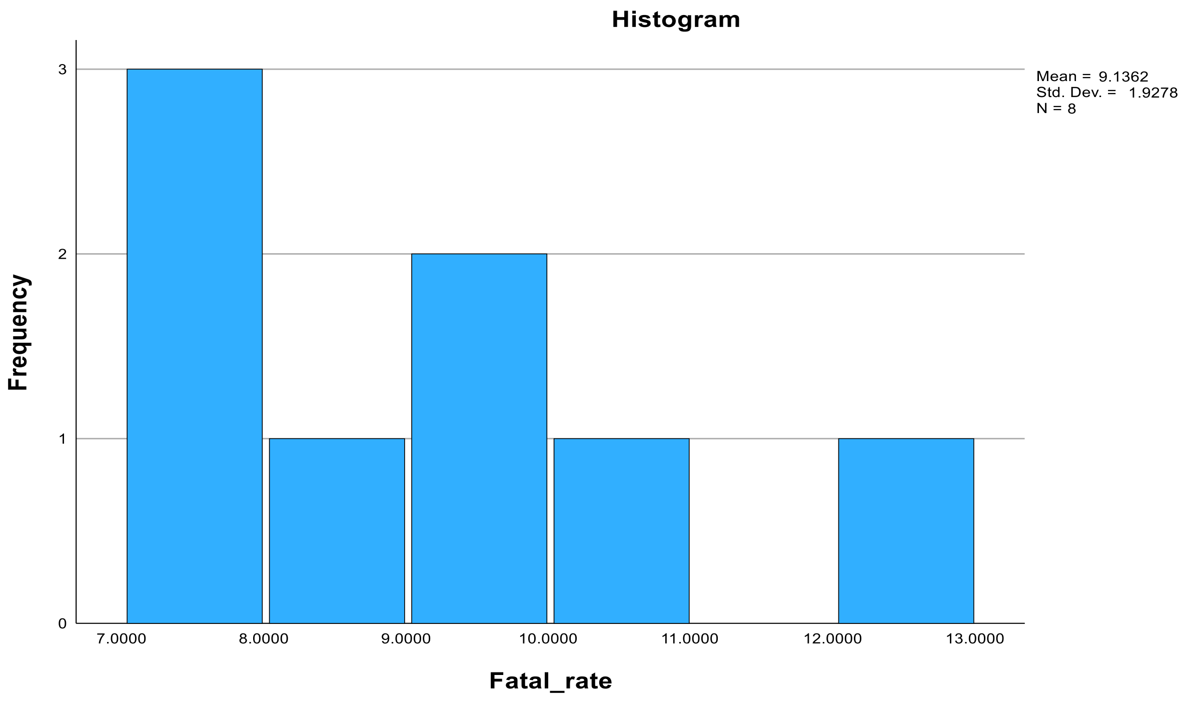
Histogram
Mean = 9.1362
Std. Dev. = 1.9278
N = 8
Frequency
0
1
2
3
7.0000
8.0000
9.0000
10.0000
11.0000
12.0000
13.0000
Fatal_rate

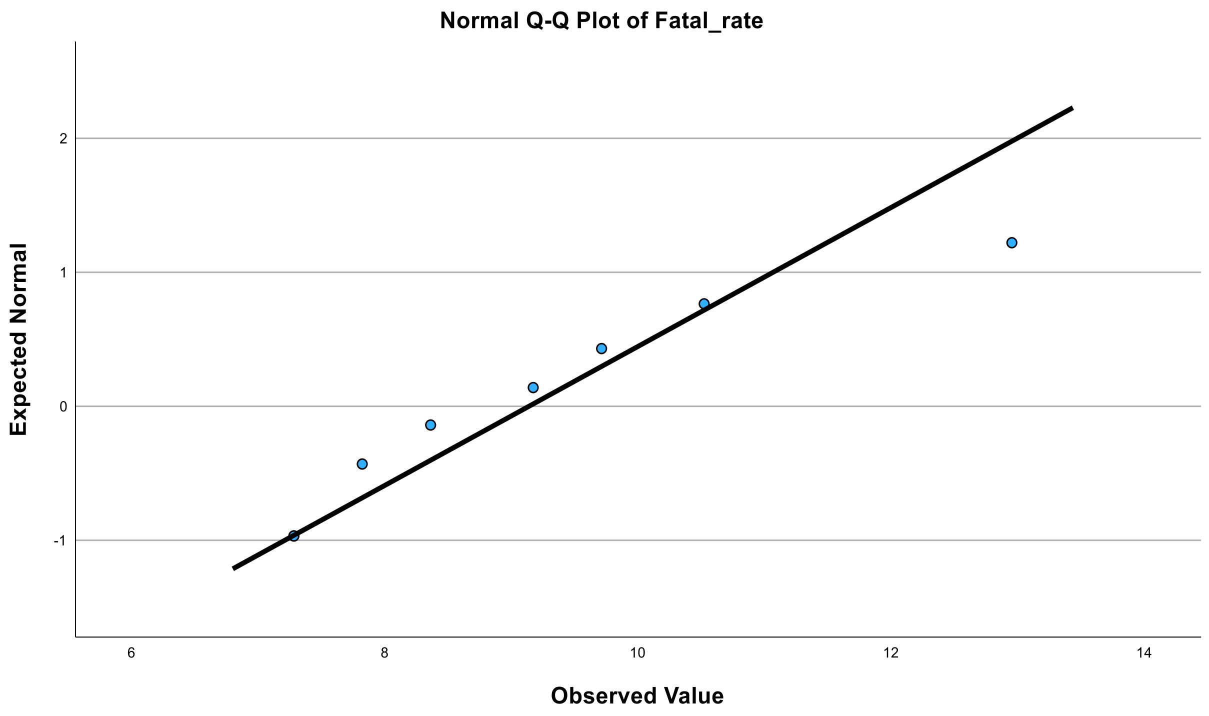
Normal Q-Q Plot of Fatal_rate
Expected Normal
-1
0
1
2
6
8
10
12
14
Observed Value

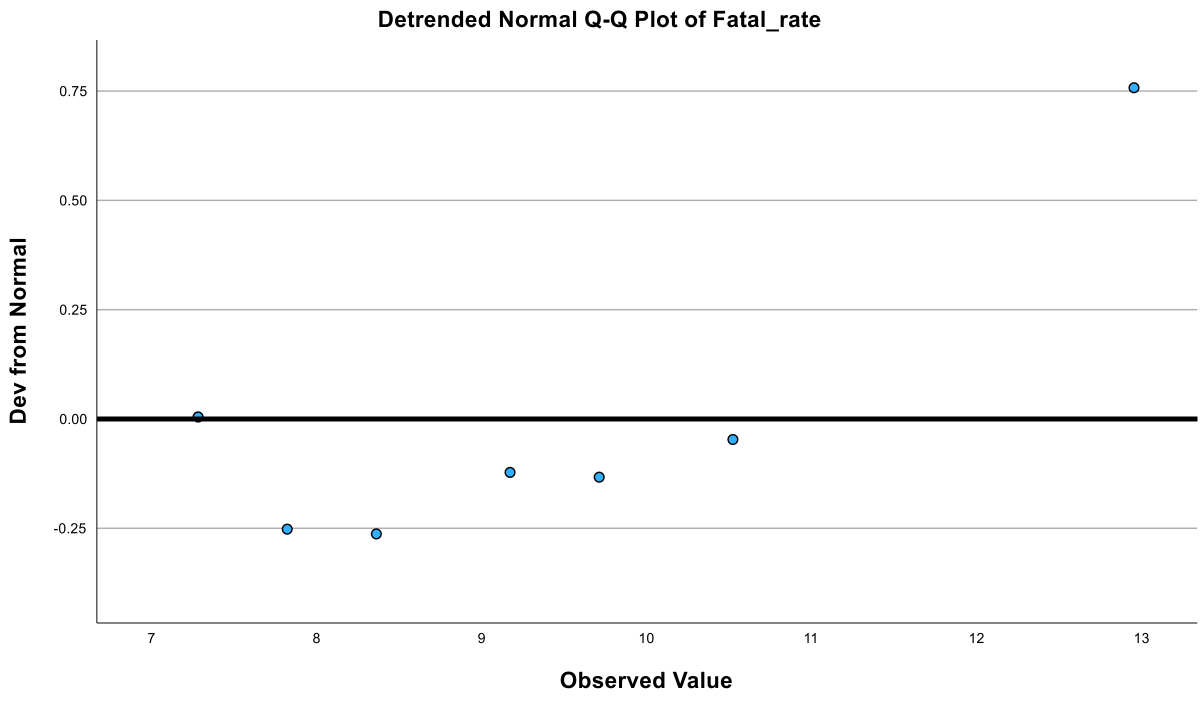
Detrended Normal Q-Q Plot of Fatal_rate
Dev from Normal
0.75
0.50
0.25
0.00
-0.25
7
8
9
10
11
12
13
Observed Value

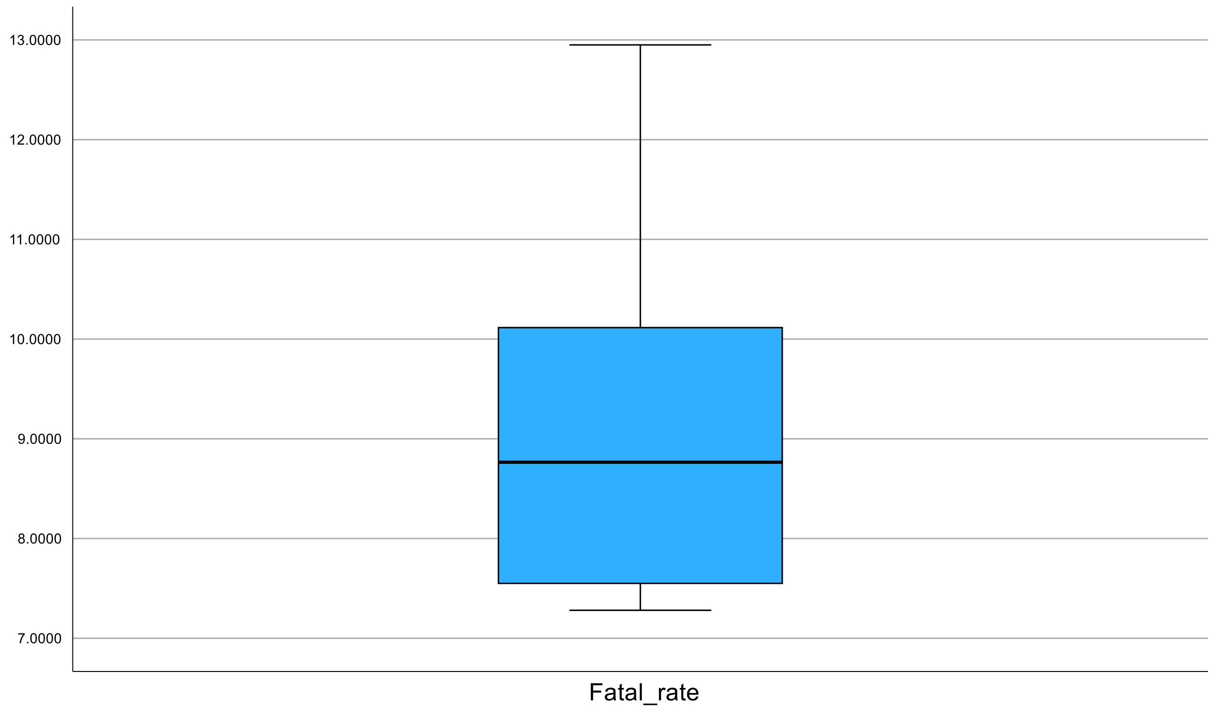
13.0000
12.0000
11.0000
10.0000
9.0000
8.0000
7.0000
Fatal_rate

Nonfatal_rate

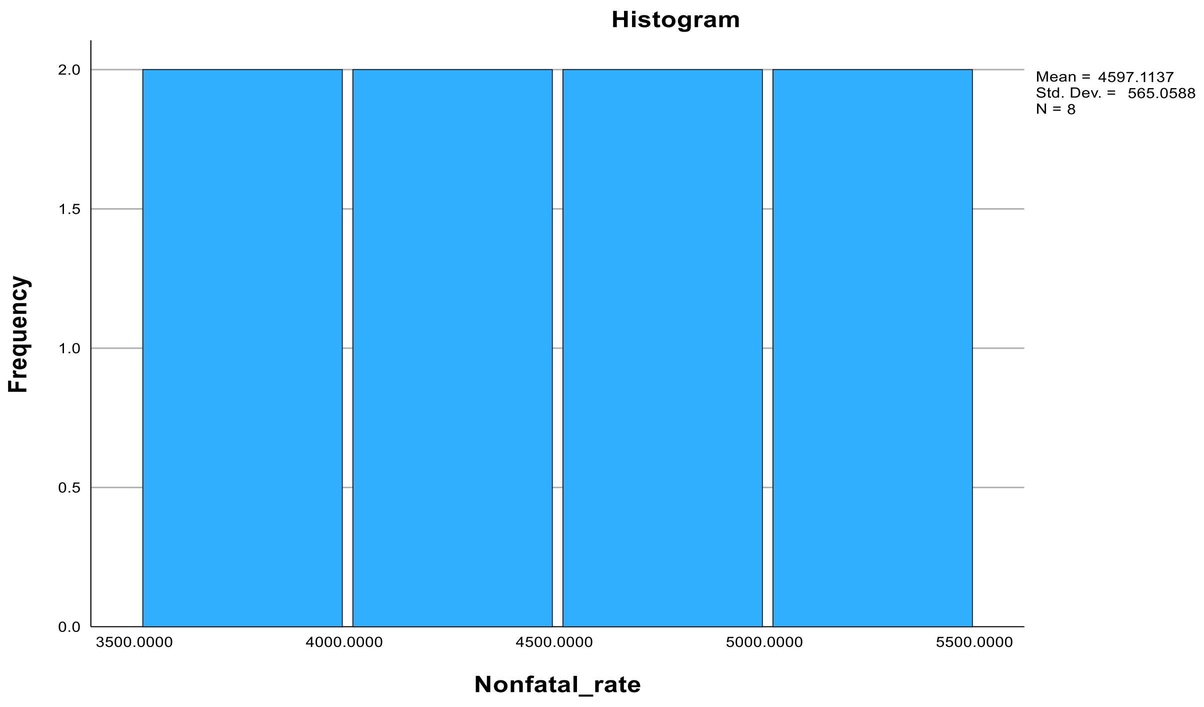

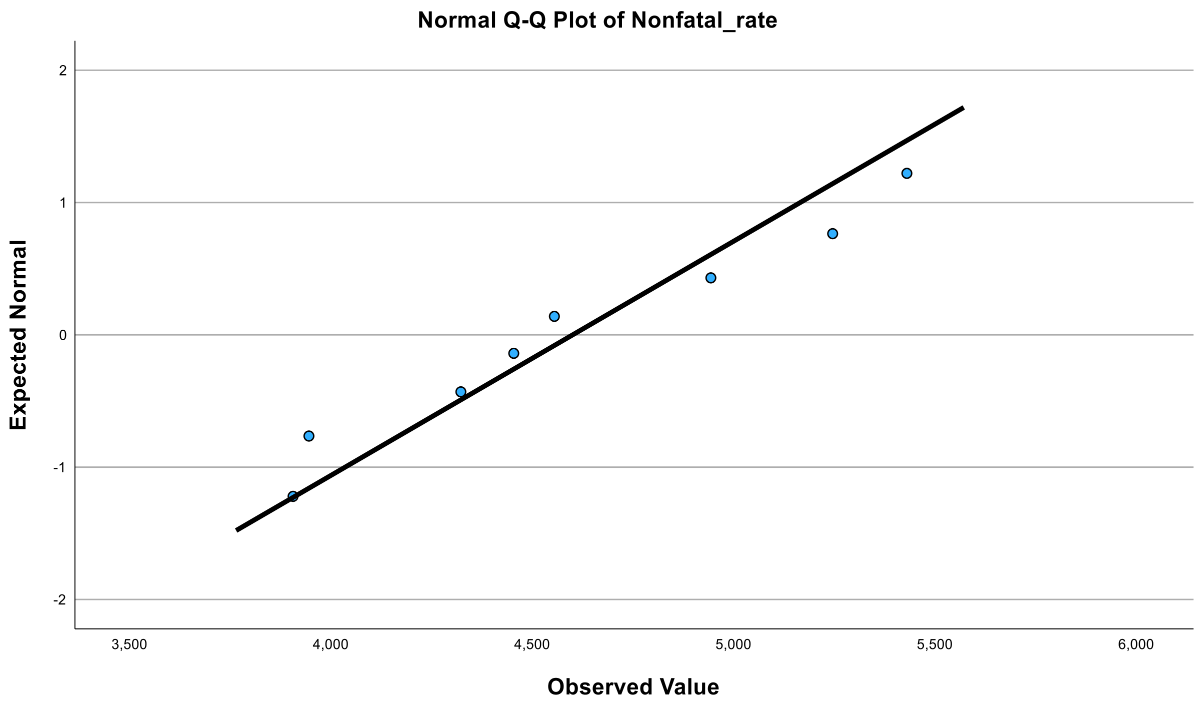

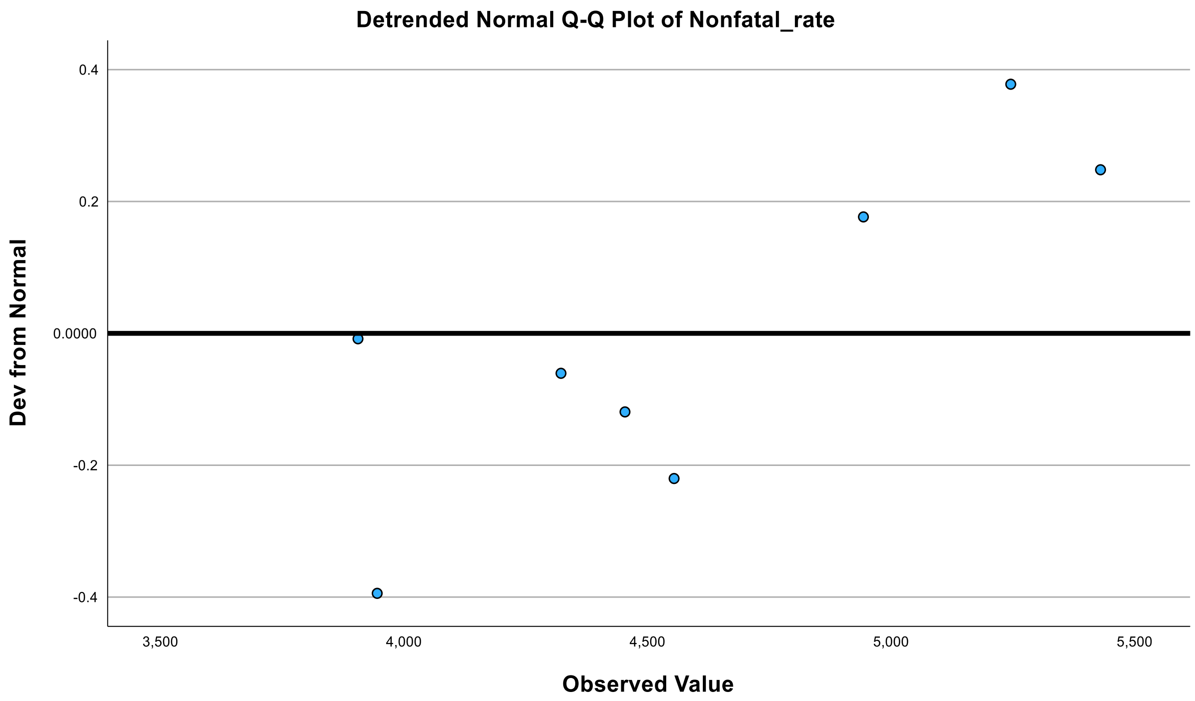
Detrended Normal Q-Q Plot of Nonfatal_rate
Dev from Normal
0.4
0.2
0.0000
-0.2
-0.4
3,500
4,000
4,500
5,000
5,500
Observed Value

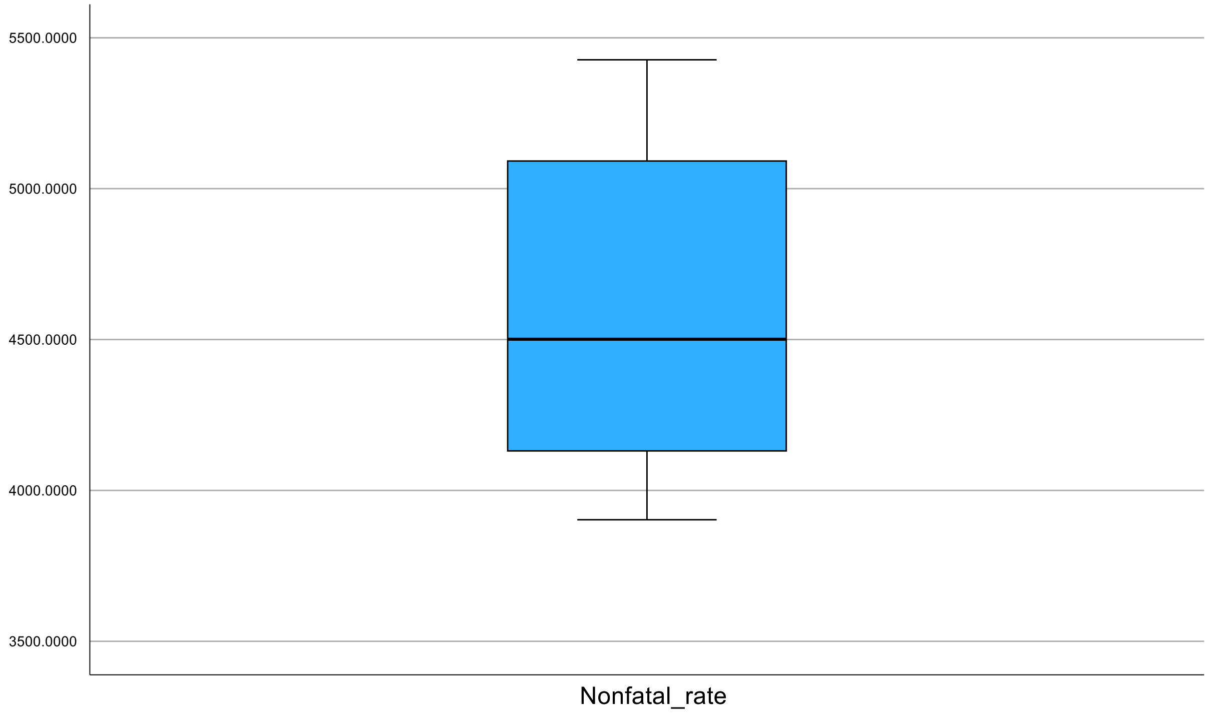
5500.0000
5000.0000
4500.0000
4000.0000
3500.0000
Nonfatal_rate

General linear multivariate regression (PP. 11-17)

Notes

Output Created		24-FEB-2026 08:32:14
Comments		
Input	Active Dataset	DataSet2
	Filter	<none>
	Weight	<none>
	Split File	<none>
	N of Rows in Working Data File	8
Missing Value Handling	Definition of Missing	User-defined missing values are treated as missing.
	Cases Used	Statistics are based on all cases with valid data for all variables in the model.
Syntax		GLM Fatal_rate Nonfatal_rate WITH Inspector_ratio /METHOD=SSTYPE(3) /INTERCEPT=INCLUDE /SAVE=PRED SEPRED RESID ZRESID COOK LEVER /EMMEANS=TABLES(OVERALL) WITH(Inspector_ratio=MEAN) /PRINT=DESCRIPTIVE ETASQ OPOWER PARAMETER TEST(SSCP) RSSCP HOMOGENEITY LOF /PLOT=SPREADLEVEL RESIDUALS /CRITERIA=ALPHA(.05) /DESIGN=Inspector_ratio.
Resources	Processor Time	00:00:00.48
	Elapsed Time	00:00:00.27
Variables Created or Modified	PRE_1	Predicted Value for Fatal_rate
	PRE_2	Predicted Value for Nonfatal_rate
	SEP_1	Standard Error of Predicted Value for Fatal_rate
	SEP_2	Standard Error of Predicted Value for Nonfatal_rate
	RES_1	Residual for Fatal_rate
	RES_2	Residual for Nonfatal_rate
	ZRE_1	Standardized Residual for Fatal_rate
	ZRE_2	Standardized Residual for Nonfatal_rate
	COO_1	Cook's Distance for Fatal_rate
	COO_2	Cook's Distance for Nonfatal_rate
	LEV_1	Uncentered Leverage Value for Fatal_rate
	LEV_2	Uncentered Leverage Value for Nonfatal_rate

Warnings

The HOMOGENEITY specification in the PRINT subcommand will be ignored because there are no between-subjects factors.
The SPREADLEVEL specification has been found in the PLOT subcommand, but the model includes no factors. The spread versus level plot will not be produced.

Descriptive Statistics

	Mean	Std. Deviation	N
Fatal_rate	9.136250	1.9278481	8
Nonfatal_rate	4597.113750	565.0587506	8

Bartlett's Test of Sphericity[a]

Likelihood Ratio	.000
Approx. Chi-Square	51.721
df	2
Sig.	<.001

Tests the null hypothesis that the residual covariance matrix is proportional to an identity matrix.[a]
a. Design: Intercept + Inspector_ratio

Multivariate Tests[a]

Effect		Value	F	Hypothesis df	Error df	Sig.
Intercept	Pillai's Trace	.557	3.147[b]	2.000	5.000	.130
	Wilks' Lambda	.443	3.147[b]	2.000	5.000	.130
	Hotelling's Trace	1.259	3.147[b]	2.000	5.000	.130
	Roy's Largest Root	1.259	3.147[b]	2.000	5.000	.130
Inspector_ratio	Pillai's Trace	.329	1.224[b]	2.000	5.000	.369
	Wilks' Lambda	.671	1.224[b]	2.000	5.000	.369
	Hotelling's Trace	.490	1.224[b]	2.000	5.000	.369
	Roy's Largest Root	.490	1.224[b]	2.000	5.000	.369

Multivariate Tests[a]

Effect		Partial Eta Squared	Noncent. Parameter	Observed Power[c]
Intercept	Pillai's Trace	.557	6.293	.364
	Wilks' Lambda	.557	6.293	.364
	Hotelling's Trace	.557	6.293	.364
	Roy's Largest Root	.557	6.293	.364
Inspector_ratio	Pillai's Trace	.329	2.448	.167
	Wilks' Lambda	.329	2.448	.167
	Hotelling's Trace	.329	2.448	.167
	Roy's Largest Root	.329	2.448	.167

a. Design: Intercept + Inspector_ratio
b. Exact statistic
c. Computed using alpha = .05

Tests of Between-Subjects Effects

Source	Dependent Variable	Type III Sum of Squares	df	Mean Square	F	Sig.
Corrected Model	Fatal_rate	.561[a]	1	.561	.132	.728
	Nonfatal_rate	352731.641[b]	1	352731.641	1.124	.330
Intercept	Fatal_rate	.405	1	.405	.095	.768
	Nonfatal_rate	1663462.842	1	1663462.842	5.302	.061
Inspector_ratio	Fatal_rate	.561	1	.561	.132	.728
	Nonfatal_rate	352731.641	1	352731.641	1.124	.330
Error	Fatal_rate	25.455	6	4.242		
	Nonfatal_rate	1882308.100	6	313718.017		
Total	Fatal_rate	693.785	8			
	Nonfatal_rate	171302678.385	8			
Corrected Total	Fatal_rate	26.016	7			
	Nonfatal_rate	2235039.741	7			

Tests of Between-Subjects Effects

Source	Dependent Variable	Partial Eta Squared	Noncent. Parameter	Observed Power[c]
Corrected Model	Fatal_rate	.022	.132	.061
	Nonfatal_rate	.158	1.124	.146
Intercept	Fatal_rate	.016	.095	.058
	Nonfatal_rate	.469	5.302	.489
Inspector_ratio	Fatal_rate	.022	.132	.061
	Nonfatal_rate	.158	1.124	.146
Error	Fatal_rate			
	Nonfatal_rate			
Total	Fatal_rate			
	Nonfatal_rate			
Corrected Total	Fatal_rate			
	Nonfatal_rate			

a. R Squared = .022 (Adjusted R Squared = -.141)
b. R Squared = .158 (Adjusted R Squared = .017)
c. Computed using alpha = .05

Parameter Estimates

Dependent Variable	Parameter	B	Std. Error	t	Sig.	95% Confidence Interval Lower Bound
Fatal_rate	Intercept	4.199	13.591	.309	.768	-29.057
	Inspector_ratio	4.266	11.728	.364	.728	-24.431
Nonfatal_rate	Intercept	8510.395	3695.838	2.303	.061	-532.994
	Inspector_ratio	-3381.681	3189.188	-1.060	.330	-11185.344

Parameter Estimates

Dependent Variable	Parameter	95% Confidence Interval Upper Bound	Partial Eta Squared	Noncent. Parameter	Observed Power[a]
Fatal_rate	Intercept	37.455	.016	.309	.058
	Inspector_ratio	32.964	.022	.364	.061
Nonfatal_rate	Intercept	17553.784	.469	2.303	.489
	Inspector_ratio	4421.982	.158	1.060	.146

a. Computed using alpha = .05

Between-Subjects SSCP Matrix

			Fatal_rate	Nonfatal_rate
Hypothesis	Intercept	Fatal_rate	.405	820.761
		Nonfatal_rate	820.761	1663462.842
	Inspector_ratio	Fatal_rate	.561	-445.022
		Nonfatal_rate	-445.022	352731.641
Error		Fatal_rate	25.455	4405.894
		Nonfatal_rate	4405.894	1882308.100

Based on Type III Sum of Squares

Residual SSCP Matrix

		Fatal_rate	Nonfatal_rate
Sum-of-Squares and Cross-Products	Fatal_rate	25.455	4405.894
	Nonfatal_rate	4405.894	1882308.100
Covariance	Fatal_rate	4.242	734.316
	Nonfatal_rate	734.316	313718.017
Correlation	Fatal_rate	1.000	.637
	Nonfatal_rate	.637	1.000

Based on Type III Sum of Squares

Lack of Fit

Multivariate Tests

Dependent Variables		Value	F	Hypothesis df	Error df
Fatal_rate, Nonfatal_rate	Pillai's Trace	.[a]	.	.	.
	Wilks' Lambda	.[a]	.	.	.
	Hotelling's Trace	.[a]	.	.	.
	Roy's Largest Root	.[a]	.	.	.
Fatal_rate	Pillai's Trace	.[a]	.	.	.
	Wilks' Lambda	.[a]	.	.	.
	Hotelling's Trace	.[a]	.	.	.
	Roy's Largest Root	.[a]	.	.	.
Nonfatal_rate	Pillai's Trace	.[a]	.	.	.
	Wilks' Lambda	.[a]	.	.	.
	Hotelling's Trace	.[a]	.	.	.
	Roy's Largest Root	.[a]	.	.	.

Multivariate Tests

Dependent Variables		Sig.	Partial Eta Squared	Noncent. Parameter
Fatal_rate, Nonfatal_rate	Pillai's Trace	.	.	.
	Wilks' Lambda	.	.	.
	Hotelling's Trace	.	.	.
	Roy's Largest Root	.	.	.
Fatal_rate	Pillai's Trace	.	.	.
	Wilks' Lambda	.	.	.
	Hotelling's Trace	.	.	.
	Roy's Largest Root	.	.	.
Nonfatal_rate	Pillai's Trace	.	.	.
	Wilks' Lambda	.	.	.
	Hotelling's Trace	.	.	.
	Roy's Largest Root	.	.	.

Multivariate Tests

Dependent Variables		Observed Power[b]
Fatal_rate, Nonfatal_rate	Pillai's Trace	.
	Wilks' Lambda	.
	Hotelling's Trace	.
	Roy's Largest Root	.
Fatal_rate	Pillai's Trace	.
	Wilks' Lambda	.
	Hotelling's Trace	.
	Roy's Largest Root	.
Nonfatal_rate	Pillai's Trace	.
	Wilks' Lambda	.
	Hotelling's Trace	.
	Roy's Largest Root	.

a. The pure error sub-matrix corresponding to the selected dependent variables is a zero matrix.
b. Computed using alpha = .05

Univariate Tests

Dependent Variable	Source	Sum of Squares	df	Mean Square	F	Sig.
Fatal_rate	Lack of Fit	25.455	6	4.242	.	.
	Pure Error	.000	0	.		
Nonfatal_rate	Lack of Fit	1882308.100	6	313718.017	.	.
	Pure Error	.000	0	.		

Univariate Tests

Dependent Variable	Source	Partial Eta Squared	Noncent. Parameter	Observed Power[a]
Fatal_rate	Lack of Fit	1.000	.	.
	Pure Error			
Nonfatal_rate	Lack of Fit	1.000	.	.
	Pure Error			

a. Computed using alpha = .05

SSCP Matrix

		Fatal_rate	Nonfatal_rate
Lack of Fit	Fatal_rate	25.455	4405.894
	Nonfatal_rate	4405.894	1882308.100
Pure Error	Fatal_rate	.000	.000
	Nonfatal_rate	.000	.000

Estimated Marginal Means

Grand Mean

Dependent Variable	Mean	Std. Error	95% Confidence Interval Lower Bound	95% Confidence Interval Upper Bound
Fatal_rate	9.136[a]	.728	7.354	10.918
Nonfatal_rate	4597.114[a]	198.027	4112.559	5081.669

a. Covariates appearing in the model are evaluated at the following values: Inspector_ratio = 1.157200.

Observed * Predicted * Std. Residual Plots

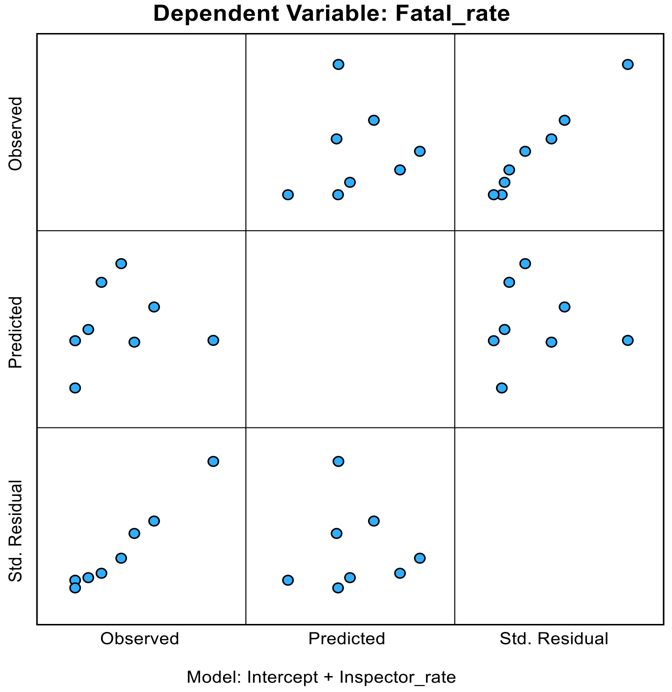

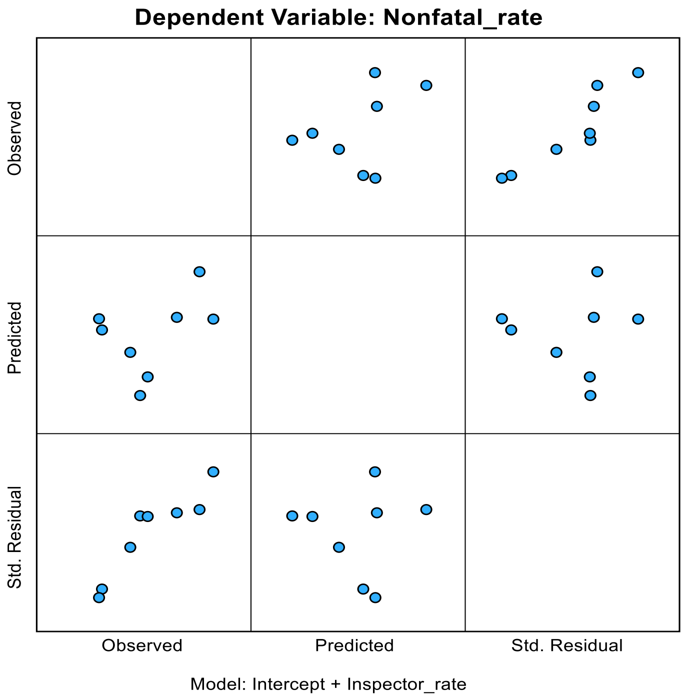
Dependent Variable: Nonfatal_rate
Observed
Predicted
Std. Residual
Observed
Predicted
Std. Residual
Model: Intercept + Inspector_rate

General linear multivariate regression with bootstrap-2000 with Confidence Intervals of 95% (PP. 18-24)

Bootstrap

Notes

Output Created		24-FEB-2026 08:36:56
Comments		
Input	Active Dataset	DataSet2
	Filter	<none>
	Weight	<none>
	Split File	<none>
Syntax		BOOTSTRAP<br>/SAMPLING METHOD=SIMPLE<br>/VARIABLES TARGET=Fatal_rate Nonfatal_rate INPUT= Inspector_ratio<br>/CRITERIA CILEVEL=95 CITYPE=PERCENTILE NSAMPLES=2000<br>/MISSING USERMISSING=EXCLUDE.
Resources	Processor Time	00:00:00.02
	Elapsed Time	00:00:00.02

Bootstrap Specifications

Sampling Method	Simple
Number of Samples	2000
Confidence Interval Level	95.0%
Confidence Interval Type	Percentile

General Linear Model

Notes

Output Created		24-FEB-2026 08:36:56
Comments		
Input	Active Dataset	DataSet2
	Filter	<none>
	Weight	<none>
	Split File	<none>
	N of Rows in Working Data File	16008
Missing Value Handling	Definition of Missing	User-defined missing values are treated as missing.
	Cases Used	Statistics are based on all cases with valid data for all variables in the model.
Syntax		GLM Fatal_rate Nonfatal_rate WITH

		Inspector_ratio<br>/METHOD=SSTYPE(3)<br>/INTERCEPT=INCLUDE<br>/SAVE=PRED SEPRED RESID ZRESID COOK LEVER<br>/EMMEANS=TABLES(OVERALL) WITH(Inspector_ratio=MEAN)<br>/PRINT=DESCRIPTIVE ETASQ OPOWER PARAMETER TEST(SSCP) RSSCP HOMOGENEITY LOF<br>/PLOT=SPREADLEVEL RESIDUALS<br>/CRITERIA=ALPHA(.05)<br>/DESIGN=Inspector_ratio.
Resources	Processor Time	00:00:25.98
	Elapsed Time	00:00:25.56

Warnings

The HOMOGENEITY specification in the PRINT subcommand will be ignored because there are no between-subjects factors.
The SPREADLEVEL specification has been found in the PLOT subcommand, but the model includes no factors. The spread versus level plot will not be produced.
This command is trying to save new variables while bootstrapping is in effect. The new variables will be lost.<br>Execution of this command stops.

Descriptive Statistics

	Mean	Std. Deviation	N
Fatal_rate	9.136250	1.9278481	8
Nonfatal_rate	4597.113750	565.0587506	8

Bartlett's Test of Sphericity[a]

Likelihood Ratio	.000
Approx. Chi-Square	51.721
df	2
Sig.	<.001

Tests the null hypothesis that the residual covariance matrix is proportional to an identity matrix.[a]
a. Design: Intercept + Inspector_ratio

Multivariate Tests[a]

Effect		Value	F	Hypothesis df	Error df	Sig.
Intercept	Pillai's Trace	.557	3.147[b]	2.000	5.000	.130
	Wilks' Lambda	.443	3.147[b]	2.000	5.000	.130
	Hotelling's Trace	1.259	3.147[b]	2.000	5.000	.130
	Roy's Largest Root	1.259	3.147[b]	2.000	5.000	.130
Inspector_ratio	Pillai's Trace	.329	1.224[b]	2.000	5.000	.369
	Wilks' Lambda	.671	1.224[b]	2.000	5.000	.369

	Hotelling's Trace	.490	1.224[b]	2.000	5.000	.369
	Roy's Largest Root	.490	1.224[b]	2.000	5.000	.369

Multivariate Tests[a]

Effect		Partial Eta Squared	Noncent. Parameter	Observed Power[c]
Intercept	Pillai's Trace	.557	6.293	.364
	Wilks' Lambda	.557	6.293	.364
	Hotelling's Trace	.557	6.293	.364
	Roy's Largest Root	.557	6.293	.364
Inspector_ratio	Pillai's Trace	.329	2.448	.167
	Wilks' Lambda	.329	2.448	.167
	Hotelling's Trace	.329	2.448	.167
	Roy's Largest Root	.329	2.448	.167

a. Design: Intercept + Inspector_ratio
b. Exact statistic
c. Computed using alpha = .05

Tests of Between-Subjects Effects

Source	Dependent Variable	Type III Sum of Squares	df	Mean Square	F	Sig.
Corrected Model	Fatal_rate	.561[a]	1	.561	.132	.728
	Nonfatal_rate	352731.641[b]	1	352731.641	1.124	.330
Intercept	Fatal_rate	.405	1	.405	.095	.768
	Nonfatal_rate	1663462.842	1	1663462.842	5.302	.061
Inspector_ratio	Fatal_rate	.561	1	.561	.132	.728
	Nonfatal_rate	352731.641	1	352731.641	1.124	.330
Error	Fatal_rate	25.455	6	4.242		
	Nonfatal_rate	1882308.100	6	313718.017		
Total	Fatal_rate	693.785	8			
	Nonfatal_rate	171302678.385	8			
Corrected Total	Fatal_rate	26.016	7			
	Nonfatal_rate	2235039.741	7			

Tests of Between-Subjects Effects

Source	Dependent Variable	Partial Eta Squared	Noncent. Parameter	Observed Power[c]
Corrected Model	Fatal_rate	.022	.132	.061
	Nonfatal_rate	.158	1.124	.146
Intercept	Fatal_rate	.016	.095	.058
	Nonfatal_rate	.469	5.302	.489
Inspector_ratio	Fatal_rate	.022	.132	.061
	Nonfatal_rate	.158	1.124	.146
Error	Fatal_rate			
	Nonfatal_rate			
Total	Fatal_rate			
	Nonfatal_rate			
Corrected Total	Fatal_rate			

Nonfatal_rate			

a. R Squared = .022 (Adjusted R Squared = -.141)
b. R Squared = .158 (Adjusted R Squared = .017)
c. Computed using alpha = .05

Parameter Estimates

Dependent Variable	Parameter	B	Std. Error	t	Sig.	95% Confidence Interval Lower Bound
Fatal_rate	Intercept	4.199	13.591	.309	.768	-29.057
	Inspector_ratio	4.266	11.728	.364	.728	-24.431
Nonfatal_rate	Intercept	8510.395	3695.838	2.303	.061	-532.994
	Inspector_ratio	-3381.681	3189.188	-1.060	.330	-11185.344

Parameter Estimates

Dependent Variable	Parameter	95% Confidence Interval Upper Bound	Partial Eta Squared	Noncent. Parameter	Observed Power[a]
Fatal_rate	Intercept	37.455	.016	.309	.058
	Inspector_ratio	32.964	.022	.364	.061
Nonfatal_rate	Intercept	17553.784	.469	2.303	.489
	Inspector_ratio	4421.982	.158	1.060	.146

a. Computed using alpha = .05

Bootstrap for Parameter Estimates

			Bootstrap[a]			
Dependent Variable	Parameter	B	Bias	Std. Error	Sig. (2-tailed)	95% Confidence Interval Lower
Fatal_rate	Intercept	4.199	1.882	16.997	.717	-17.853
	Inspector_ratio	4.266	-1.541	14.622	.655	-25.981
Nonfatal_rate	Intercept	8510.395	276.660	6909.890	.041	-29.514
	Inspector_ratio	-3381.681	-282.423	6051.708	.183	-9819.345

Bootstrap for Parameter Estimates

Dependent Variable	Parameter	Bootstrap 95% Confidence Interval Upper
Fatal_rate	Intercept	40.498
	Inspector_ratio	23.832
Nonfatal_rate	Intercept	15802.889
	Inspector_ratio	3699.057

a. Unless otherwise noted, bootstrap results are based on 2000 bootstrap samples

Between-Subjects SSCP Matrix

			Fatal_rate	Nonfatal_rate
Hypothesis	Intercept	Fatal_rate	.405	820.761
		Nonfatal_rate	820.761	1663462.842
	Inspector_ratio	Fatal_rate	.561	-445.022
		Nonfatal_rate	-445.022	352731.641
Error		Fatal_rate	25.455	4405.894
		Nonfatal_rate	4405.894	1882308.100

Based on Type III Sum of Squares

Residual SSCP Matrix

		Fatal_rate	Nonfatal_rate
Sum-of-Squares and Cross-Products	Fatal_rate	25.455	4405.894
	Nonfatal_rate	4405.894	1882308.100
Covariance	Fatal_rate	4.242	734.316
	Nonfatal_rate	734.316	313718.017
Correlation	Fatal_rate	1.000	.637
	Nonfatal_rate	.637	1.000

Based on Type III Sum of Squares

Lack of Fit

Multivariate Tests

Dependent Variables		Value	F	Hypothesis df	Error df
Fatal_rate, Nonfatal_rate	Pillai's Trace	.[a]	.	.	.
	Wilks' Lambda	.[a]	.	.	.
	Hotelling's Trace	.[a]	.	.	.
	Roy's Largest Root	.[a]	.	.	.
Fatal_rate	Pillai's Trace	.[a]	.	.	.
	Wilks' Lambda	.[a]	.	.	.
	Hotelling's Trace	.[a]	.	.	.
	Roy's Largest Root	.[a]	.	.	.
Nonfatal_rate	Pillai's Trace	.[a]	.	.	.
	Wilks' Lambda	.[a]	.	.	.
	Hotelling's Trace	.[a]	.	.	.
	Roy's Largest Root	.[a]	.	.	.

Multivariate Tests

Dependent Variables		Sig.	Partial Eta Squared	Noncent. Parameter
Fatal_rate, Nonfatal_rate	Pillai's Trace	.	.	.
	Wilks' Lambda	.	.	.
	Hotelling's Trace	.	.	.
	Roy's Largest Root	.	.	.
Fatal_rate	Pillai's Trace	.	.	.
	Wilks' Lambda	.	.	.

	Hotelling's Trace	.	.	.
	Roy's Largest Root	.	.	.
Nonfatal_rate	Pillai's Trace	.	.	.
	Wilks' Lambda	.	.	.
	Hotelling's Trace	.	.	.
	Roy's Largest Root	.	.	.

Multivariate Tests

Dependent Variables		Observed Power[b]
Fatal_rate, Nonfatal_rate	Pillai's Trace	.
	Wilks' Lambda	.
	Hotelling's Trace	.
	Roy's Largest Root	.
Fatal_rate	Pillai's Trace	.
	Wilks' Lambda	.
	Hotelling's Trace	.
	Roy's Largest Root	.
Nonfatal_rate	Pillai's Trace	.
	Wilks' Lambda	.
	Hotelling's Trace	.
	Roy's Largest Root	.

a. The pure error sub-matrix corresponding to the selected dependent variables is a zero matrix.
b. Computed using alpha = .05

Univariate Tests

Dependent Variable	Source	Sum of Squares	df	Mean Square	F	Sig.
Fatal_rate	Lack of Fit	25.455	6	4.242	.	.
	Pure Error	.000	0	.		
Nonfatal_rate	Lack of Fit	1882308.100	6	313718.017	.	.
	Pure Error	.000	0	.		

Univariate Tests

Dependent Variable	Source	Partial Eta Squared	Noncent. Parameter	Observed Power[a]
Fatal_rate	Lack of Fit	1.000	.	.
	Pure Error			
Nonfatal_rate	Lack of Fit	1.000	.	.
	Pure Error			

a. Computed using alpha = .05

SSCP Matrix

		Fatal_rate	Nonfatal_rate
Lack of Fit	Fatal_rate	25.455	4405.894
	Nonfatal_rate	4405.894	1882308.100
Pure Error	Fatal_rate	.000	.000
	Nonfatal_rate	.000	.000

Estimated Marginal Means

Grand Mean

Dependent Variable	Mean	Std. Error	95% Confidence Interval Lower Bound	95% Confidence Interval Upper Bound
Fatal_rate	9.136[a]	.728	7.354	10.918
Nonfatal_rate	4597.114[a]	198.027	4112.559	5081.669

a. Covariates appearing in the model are evaluated at the following values: Inspector_ratio = 1.157200.

Continued.

Descriptive statistics with log-transformation of dependent variables (PP. 25-33)

Explore

Notes

Output Created		24-FEB-2026 08:42:08
Comments		
Input	Active Dataset	DataSet2
	Filter	<none>
	Weight	<none>
	Split File	<none>
	N of Rows in Working Data File	8
Missing Value Handling	Definition of Missing	User-defined missing values for dependent variables are treated as missing.
	Cases Used	Statistics are based on cases with no missing values for any dependent variable or factor used.
Syntax		EXAMINE VARIABLES=Inspector_ratio Log_fatalrate Log_nonfatalrate<br>/PLOT BOXPLOT HISTOGRAM NPPLOT<br>/COMPARE GROUPS<br>/STATISTICS DESCRIPTIVES EXTREME<br>/CINTERVAL 95<br>/MISSING LISTWISE<br>/NOTOTAL.
Resources	Processor Time	00:00:01.51
	Elapsed Time	00:00:01.27

Case Processing Summary

	Cases					
	Valid		Missing		Total	
	N	Percent	N	Percent	N	Percent
Inspector_ratio	8	100.0%	0	0.0%	8	100.0%
Log_fatalrate	8	100.0%	0	0.0%	8	100.0%
Log_nonfatalrate	8	100.0%	0	0.0%	8	100.0%

Descriptives

			Statistic	Std. Error
Inspector_ratio	Mean		1.157200	.0234691
	95% Confidence Interval for Mean	Lower Bound	1.101705	
		Upper Bound	1.212695	

	5% Trimmed Mean		1.157500	
	Median		1.138950	
	Variance		.004	
	Std. Deviation		.0663805	
	Minimum		1.0488	
	Maximum		1.2602	
	Range		.2114	
	Interquartile Range		.0906	
	Skewness		.087	.752
	Kurtosis		.029	1.481
Log_fatalrate	Mean		.9529	.03060
	95% Confidence Interval for Mean	Lower Bound	.8806	
		Upper Bound	1.0253	
	5% Trimmed Mean		.9491	
	Median		.9423	
	Variance		.007	
	Std. Deviation		.08654	
	Minimum		.86	
	Maximum		1.11	
	Range		.25	
	Interquartile Range		.14	
	Skewness		.786	.752
	Kurtosis		.101	1.481
Log_nonfatalrate	Mean		3.6596	.01882
	95% Confidence Interval for Mean	Lower Bound	3.6151	
		Upper Bound	3.7041	
	5% Trimmed Mean		3.6593	
	Median		3.6533	
	Variance		.003	
	Std. Deviation		.05322	
	Minimum		3.59	
	Maximum		3.73	
	Range		.14	
	Interquartile Range		.11	
	Skewness		.103	.752
	Kurtosis		-1.287	1.481

Extreme Values[a]

			Case Number	Value
Inspector_ratio	Highest	1	4	1.2602
		2	5	1.2284
		3	6	1.1865
		4	7	1.1482
	Lowest	1	1	1.0488
		2	3	1.1267
		3	8	1.1291
		4	2	1.1297
Log_fatalrate	Highest	1	2	1.11
		2	6	1.02
		3	3	.99

		4	4	.96
	Lowest	1	8	.86
		2	1	.86
		3	7	.89
		4	5	.92
Log_nonfatalrate	Highest	1	2	3.73
		2	1	3.72
		3	3	3.69
		4	5	3.66
	Lowest	1	8	3.59
		2	7	3.60
		3	6	3.64
		4	4	3.65

a. The requested number of extreme values exceeds the number of data points. A smaller number of extremes is displayed.

Tests of Normality

	Kolmogorov-Smirnov[a]			Shapiro-Wilk		
	Statistic	df	Sig.	Statistic	df	Sig.
Inspector_ratio	.198	8	.200*	.950	8	.711
Log_fatalrate	.147	8	.200*	.929	8	.504
Log_nonfatalrate	.136	8	.200*	.941	8	.619

*. This is a lower bound of the true significance.
a. Lilliefors Significance Correction

Inspector_ratio

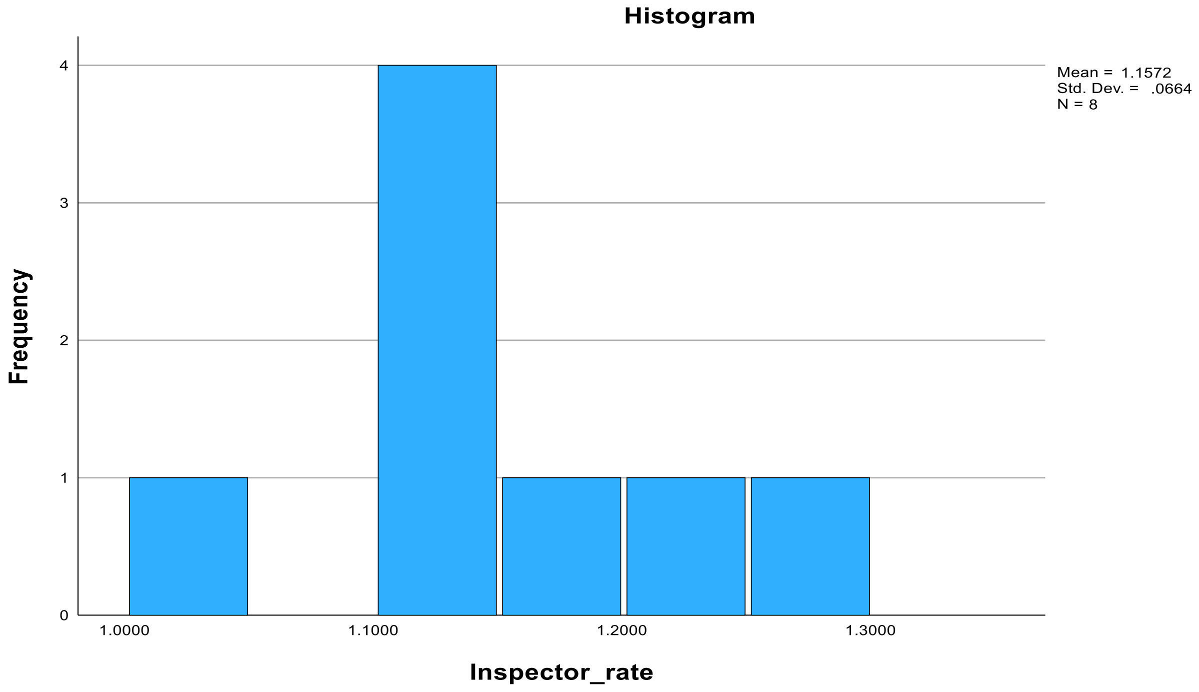

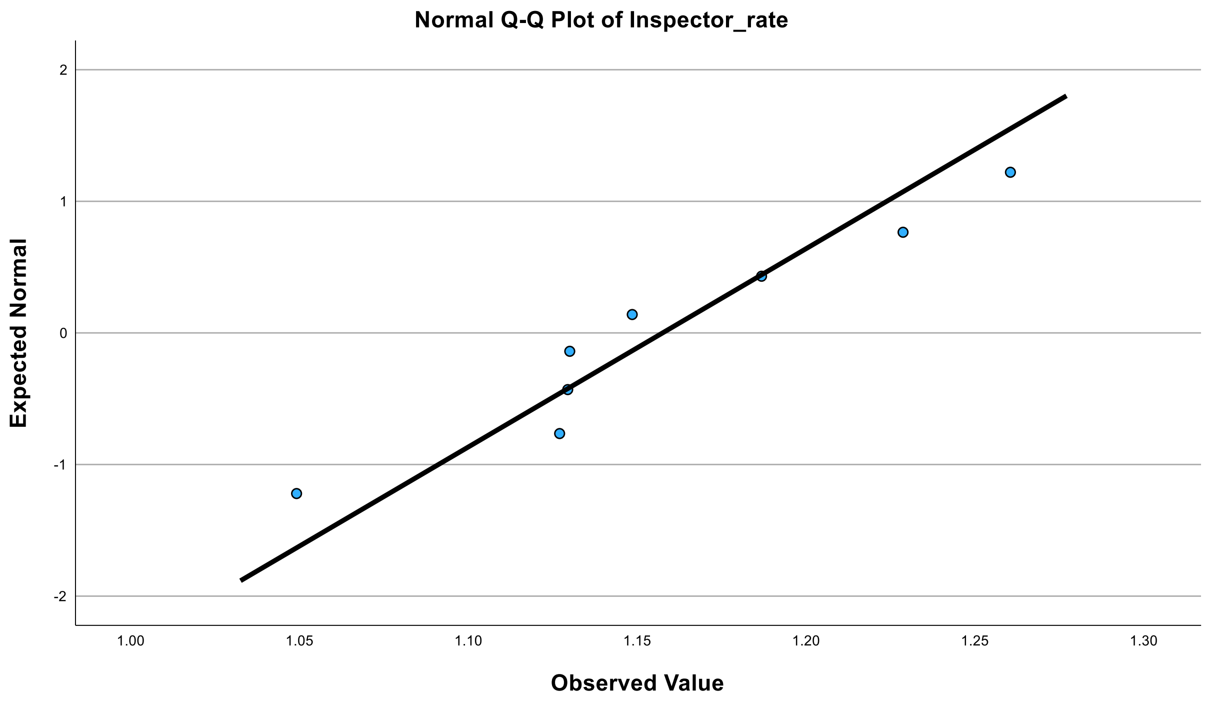
Normal Q-Q Plot of Inspector_rate
Expected Normal
Observed Value
2
1
0
-1
-2
1.00
1.05
1.10
1.15
1.20
1.25
1.30

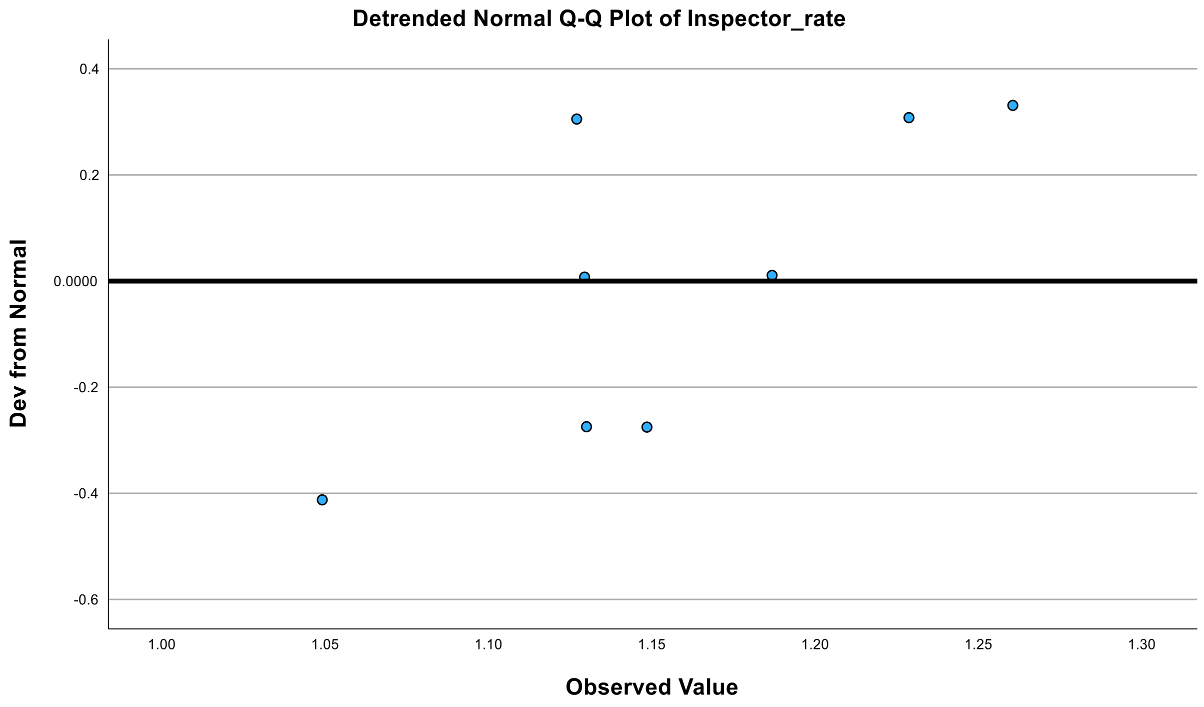
Detrended Normal Q-Q Plot of Inspector_rate
Dev from Normal
Observed Value
0.4
0.2
0.0000
-0.2
-0.4
-0.6
1.00
1.05
1.10
1.15
1.20
1.25
1.30

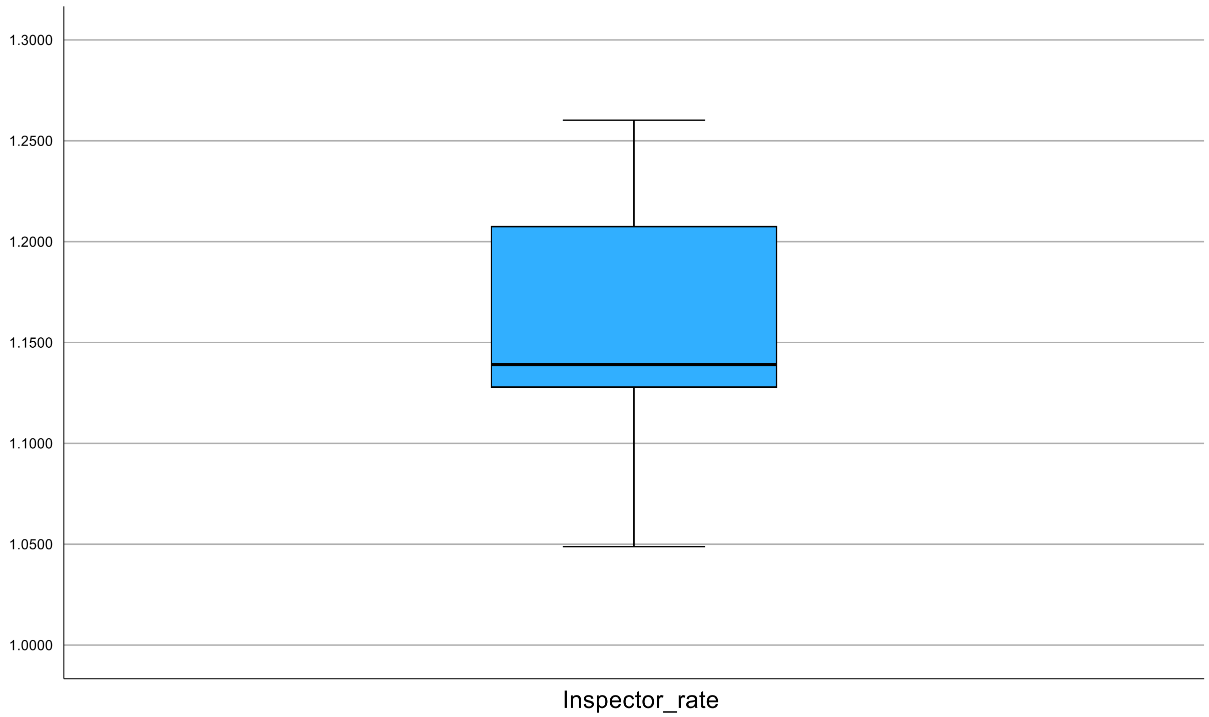

Log_fatalrate

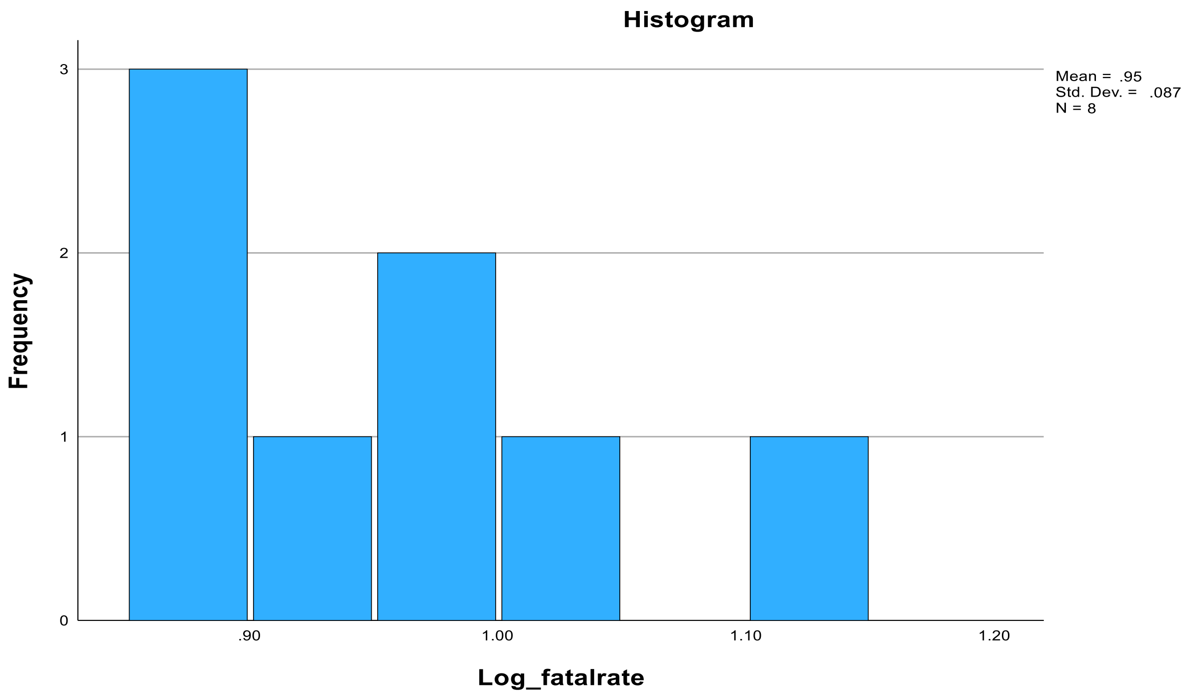

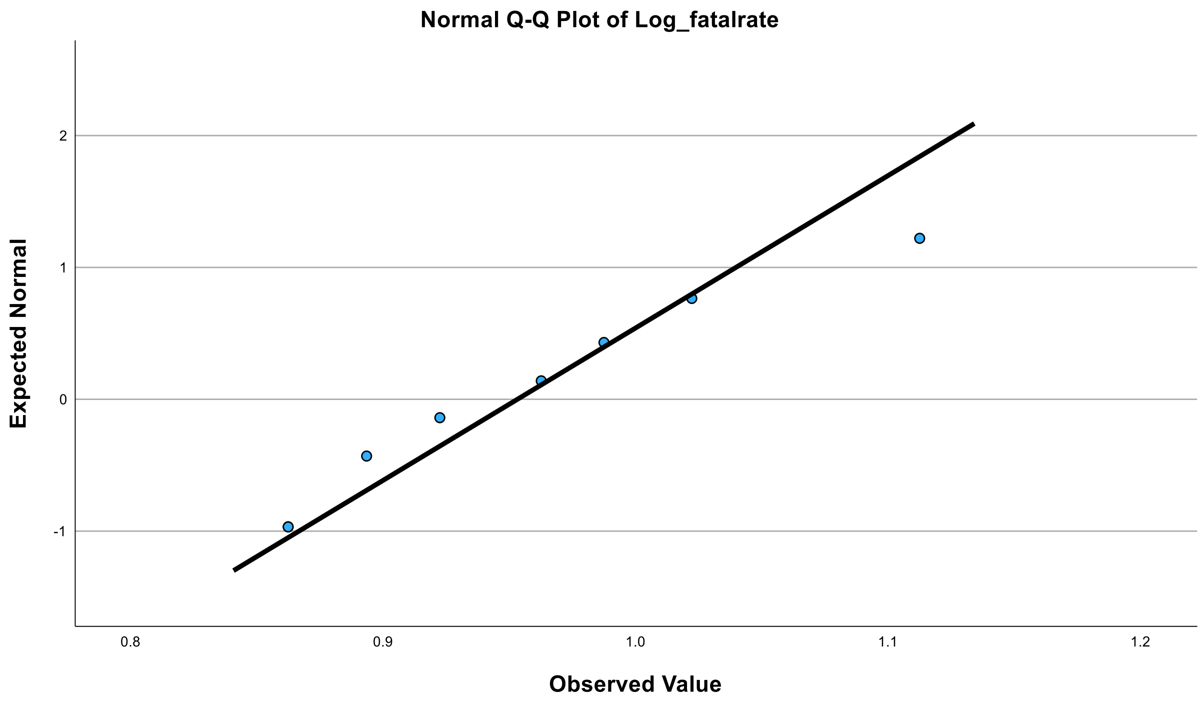
Normal Q-Q Plot of Log_fatalrate
Expected Normal
2
1
0
-1
0.8
0.9
1.0
1.1
1.2
Observed Value

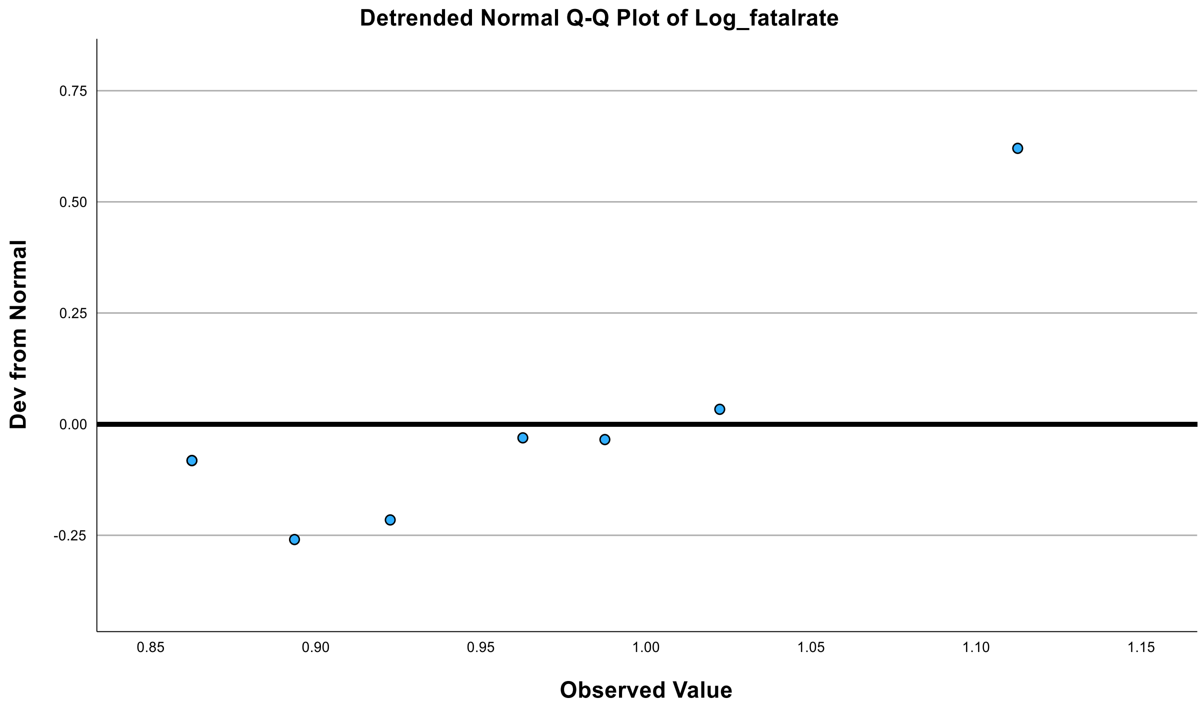
Detrended Normal Q-Q Plot of Log_fatalrate
Dev from Normal
0.75
0.50
0.25
0.00
-0.25
0.85
0.90
0.95
1.00
1.05
1.10
1.15
Observed Value

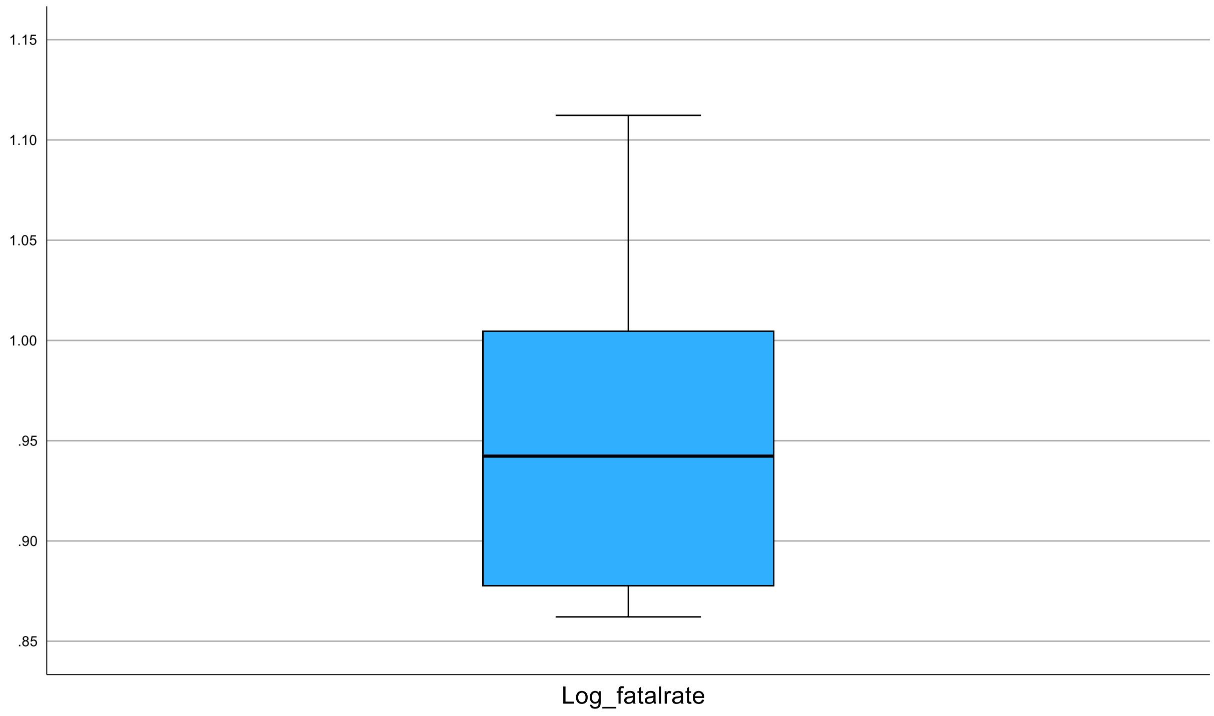

Log_nonfatalrate

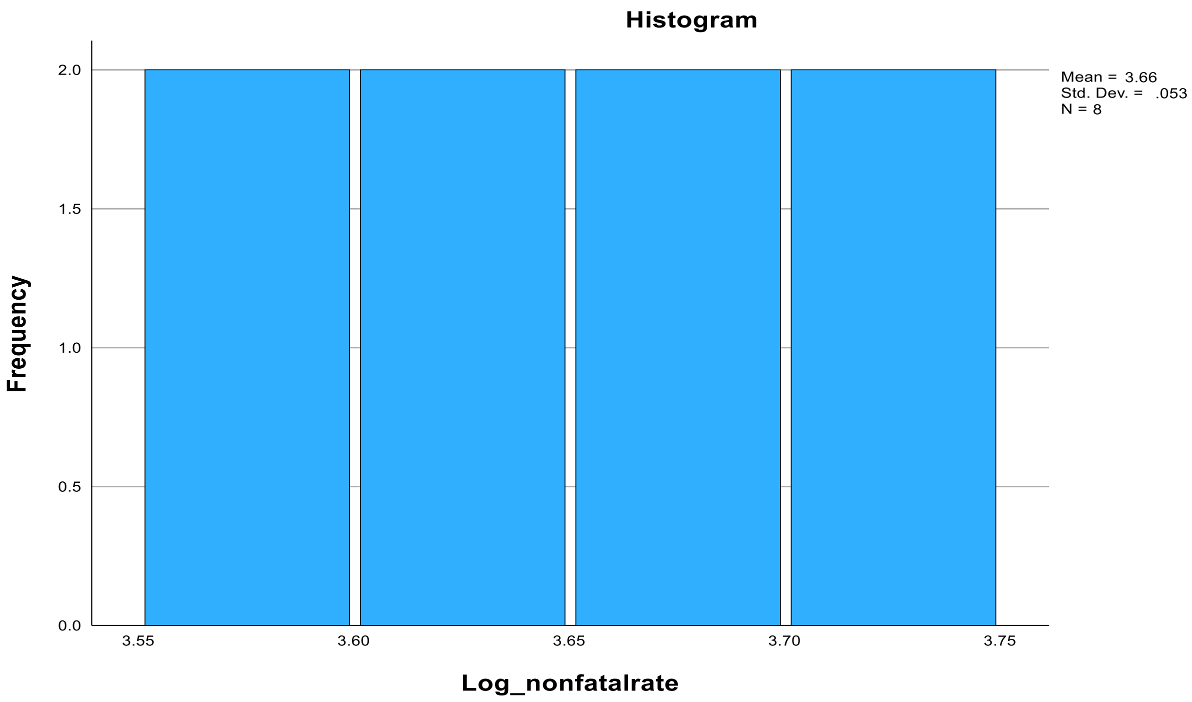

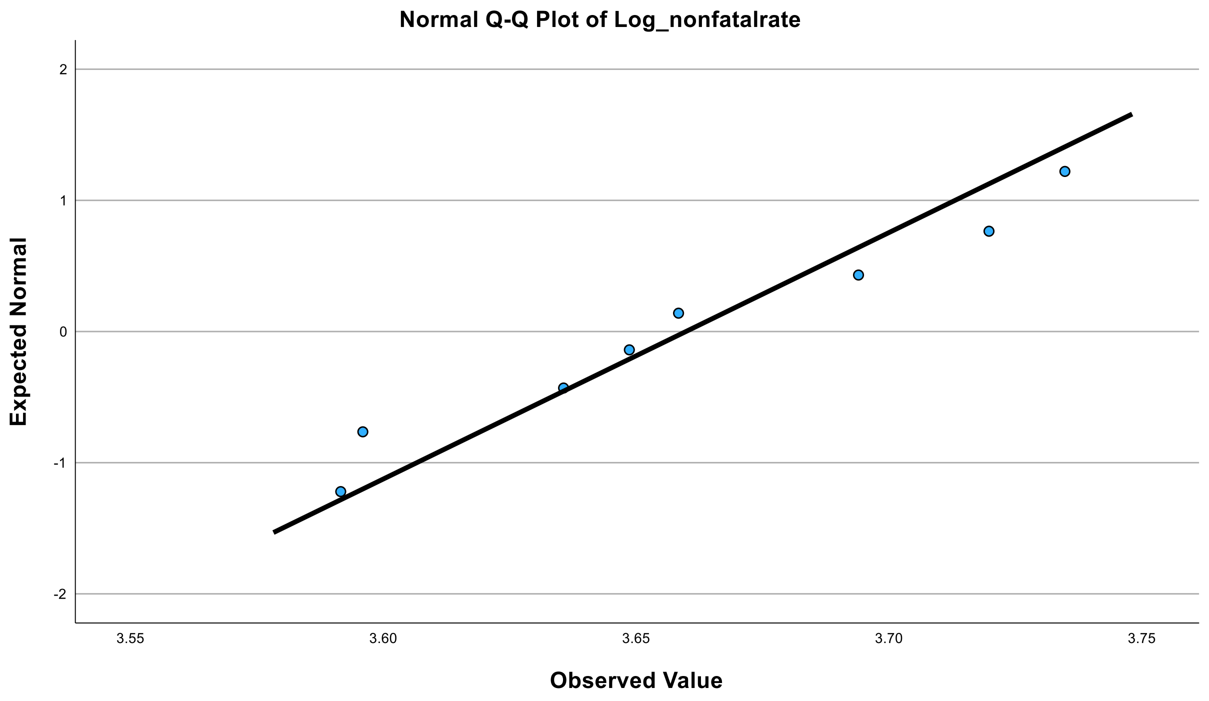
Normal Q-Q Plot of Log_nonfatalrate
Expected Normal
2
1
0
-1
-2
3.55
3.60
3.65
3.70
3.75
Observed Value

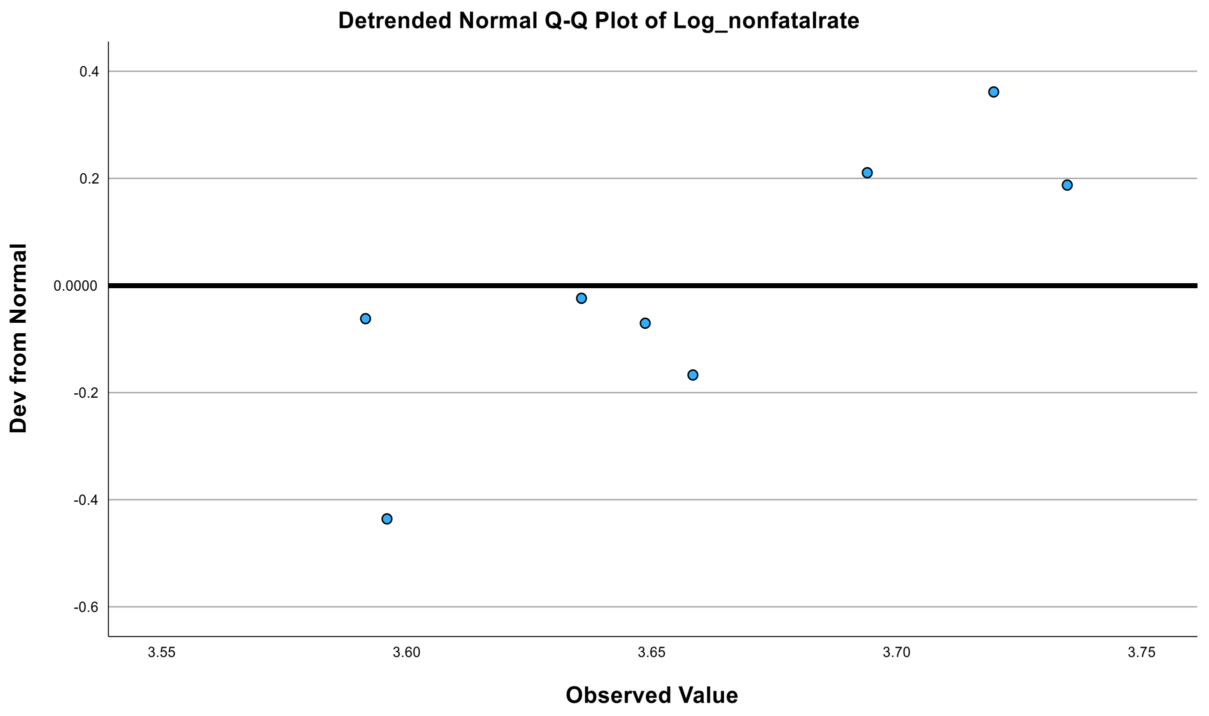
Detrended Normal Q-Q Plot of Log_nonfatalrate
Dev from Normal
0.4
0.2
0.0000
-0.2
-0.4
-0.6
3.55
3.60
3.65
3.70
3.75
Observed Value

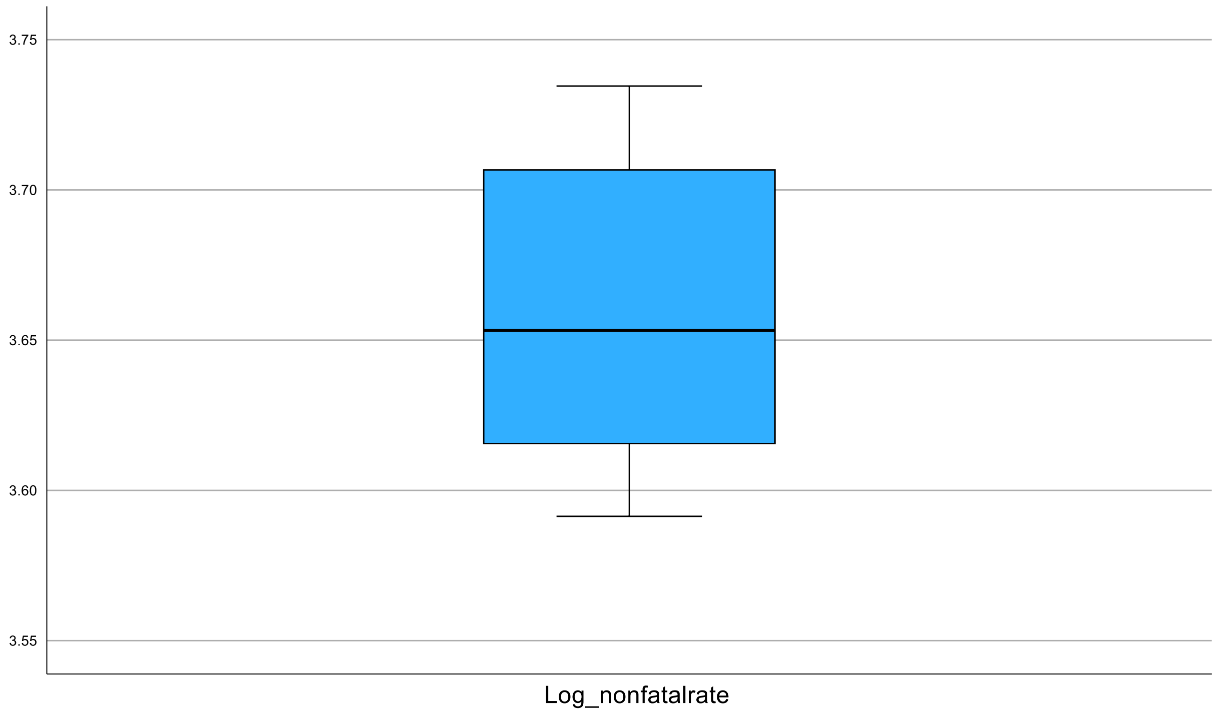
3.75
3.70
3.65
3.60
3.55
Log_nonfatalrate

Log-general linear multivariate regression with bootstrap-2000 with Confidence Intervals of 95% (PP. 34-40)

Bootstrap

Notes

Output Created		24-FEB-2026 08:43:22
Comments		
Input	Active Dataset	DataSet2
	Filter	<none>
	Weight	<none>
	Split File	<none>
	N of Rows in Working Data File	8
Syntax		BOOTSTRAP<br>/SAMPLING METHOD=SIMPLE<br>/VARIABLES TARGET=Log_fatalrate Log_nonfatalrate INPUT= Inspector_ratio<br>/CRITERIA CILEVEL=95 CITYPE=PERCENTILE NSAMPLES=2000<br>/MISSING USERMISSING=EXCLUDE.
Resources	Processor Time	00:00:00.02
	Elapsed Time	00:00:00.02

Bootstrap Specifications

Sampling Method	Simple
Number of Samples	2000
Confidence Interval Level	95.0%
Confidence Interval Type	Percentile

General Linear Model

Notes

Output Created		24-FEB-2026 08:43:22
Comments		
Input	Active Dataset	DataSet2
	Filter	<none>
	Weight	<none>
	Split File	<none>
	N of Rows in Working Data File	16008
Missing Value Handling	Definition of Missing	User-defined missing values are treated as missing.
	Cases Used	Statistics are based on all cases with valid

		data for all variables in the model.
Syntax		GLM Log_fatalrate Log_nonfatalrate WITH Inspector_ratio /METHOD=SSTYPE(3) /INTERCEPT=INCLUDE /SAVE=PRED SEPRED RESID ZRESID COOK LEVER /EMMEANS=TABLES(OVERALL) WITH(Inspector_ratio=MEAN) /PRINT=DESCRIPTIVE ETASQ OPOWER PARAMETER TEST(SSCP) RSSCP HOMOGENEITY LOF /PLOT=SPREADLEVEL RESIDUALS /CRITERIA=ALPHA(.05) /DESIGN=Inspector_ratio.
Resources	Processor Time	00:00:25.30
	Elapsed Time	00:00:25.38

Warnings

The HOMOGENEITY specification in the PRINT subcommand will be ignored because there are no between-subjects factors.
The SPREADLEVEL specification has been found in the PLOT subcommand, but the model includes no factors. The spread versus level plot will not be produced.
This command is trying to save new variables while bootstrapping is in effect. The new variables will be lost.<br>Execution of this command stops.

Descriptive Statistics

	Mean	Std. Deviation	N
Log_fatalrate	.9529	.08654	8
Log_nonfatalrate	3.6596	.05322	8

Bartlett's Test of Sphericity[a]

Likelihood Ratio	.045
Approx. Chi-Square	3.871
df	2
Sig.	.144

Tests the null hypothesis that the residual covariance matrix is proportional to an identity matrix.[a]

a. Design: Intercept + Inspector_ratio

Multivariate Tests[a]

Effect		Value	F	Hypothesis df	Error df	Sig.
Intercept	Pillai's Trace	.969	78.605[b]	2.000	5.000	<.001
	Wilks' Lambda	.031	78.605[b]	2.000	5.000	<.001
	Hotelling's Trace	31.442	78.605[b]	2.000	5.000	<.001
	Roy's Largest Root	31.442	78.605[b]	2.000	5.000	<.001
Inspector_ratio	Pillai's Trace	.334	1.254[b]	2.000	5.000	.362
	Wilks' Lambda	.666	1.254[b]	2.000	5.000	.362
	Hotelling's Trace	.502	1.254[b]	2.000	5.000	.362
	Roy's Largest Root	.502	1.254[b]	2.000	5.000	.362

Multivariate Tests[a]

Effect		Partial Eta Squared	Noncent. Parameter	Observed Power[c]
Intercept	Pillai's Trace	.969	157.210	1.000
	Wilks' Lambda	.969	157.210	1.000
	Hotelling's Trace	.969	157.210	1.000
	Roy's Largest Root	.969	157.210	1.000
Inspector_ratio	Pillai's Trace	.334	2.508	.170
	Wilks' Lambda	.334	2.508	.170
	Hotelling's Trace	.334	2.508	.170
	Roy's Largest Root	.334	2.508	.170

a. Design: Intercept + Inspector_ratio
b. Exact statistic
c. Computed using alpha = .05

Tests of Between-Subjects Effects

Source	Dependent Variable	Type III Sum of Squares	df	Mean Square	F	Sig.
Corrected Model	Log_fatalrate	.002[a]	1	.002	.268	.623
	Log_nonfatalrate	.003[b]	1	.003	.933	.371
Intercept	Log_fatalrate	.009	1	.009	1.128	.329
	Log_nonfatalrate	.367	1	.367	128.493	<.001
Inspector_ratio	Log_fatalrate	.002	1	.002	.268	.623
	Log_nonfatalrate	.003	1	.003	.933	.371
Error	Log_fatalrate	.050	6	.008		
	Log_nonfatalrate	.017	6	.003		
Total	Log_fatalrate	7.317	8			
	Log_nonfatalrate	107.163	8			
Corrected Total	Log_fatalrate	.052	7			
	Log_nonfatalrate	.020	7			

Tests of Between-Subjects Effects

Source	Dependent Variable	Partial Eta Squared	Noncent. Parameter	Observed Power[c]
Corrected Model	Log_fatalrate	.043	.268	.072
	Log_nonfatalrate	.135	.933	.130
Intercept	Log_fatalrate	.158	1.128	.147
	Log_nonfatalrate	.955	128.493	1.000
Inspector_ratio	Log_fatalrate	.043	.268	.072
	Log_nonfatalrate	.135	.933	.130
Error	Log_fatalrate			
	Log_nonfatalrate			
Total	Log_fatalrate			
	Log_nonfatalrate			
Corrected Total	Log_fatalrate			
	Log_nonfatalrate			

a. R Squared = .043 (Adjusted R Squared = -.117)
b. R Squared = .135 (Adjusted R Squared = -.010)
c. Computed using alpha = .05

Parameter Estimates

Dependent Variable	Parameter	B	Std. Error	t	Sig.	95% Confidence Interval Lower Bound
Log_fatalrate	Intercept	.641	.603	1.062	.329	-.836
	Inspector_ratio	.270	.521	.518	.623	-1.005
Log_nonfatalrate	Intercept	4.000	.353	11.335	<.001	3.136
	Inspector_ratio	-.294	.304	-.966	.371	-1.039

Parameter Estimates

Dependent Variable	Parameter	95% Confidence Interval Upper Bound	Partial Eta Squared	Noncent. Parameter	Observed Power[a]
Log_fatalrate	Intercept	2.118	.158	1.062	.147
	Inspector_ratio	1.544	.043	.518	.072
Log_nonfatalrate	Intercept	4.863	.955	11.335	1.000
	Inspector_ratio	.451	.135	.966	.130

a. Computed using alpha = .05

Bootstrap for Parameter Estimates

			Bootstrap[a]			
Dependent Variable	Parameter	B	Bias	Std. Error	Sig. (2-tailed)	95% Confidence Interval Lower
Log_fatalrate	Intercept	.641	.053	.833	.245	-.352
	Inspector_ratio	.270	-.041	.722	.500	-.951
Log_nonfatalrate	Intercept	4.000	.017	.422	.010	3.216
	Inspector_ratio	-.294	-.018	.364	.211	-.925

Bootstrap for Parameter Estimates

Dependent Variable	Parameter	Bootstrap 95% Confidence Interval Upper
Log_fatalrate	Intercept	2.116
	Inspector_ratio	1.159
Log_nonfatalrate	Intercept	4.724
	Inspector_ratio	.365

a. Unless otherwise noted, bootstrap results are based on 2000 bootstrap samples

Between-Subjects SSCP Matrix

			Log_fatalrate	Log_nonfatalrate
Hypothesis	Intercept	Log_fatalrate	.009	.059
		Log_nonfatalrate	.059	.367
	Inspector_ratio	Log_fatalrate	.002	-.002
		Log_nonfatalrate	-.002	.003
Error		Log_fatalrate	.050	.018
		Log_nonfatalrate	.018	.017

Based on Type III Sum of Squares

Residual SSCP Matrix

		Log_fatalrate	Log_nonfatalrate
Sum-of-Squares and Cross-Products	Log_fatalrate	.050	.018
	Log_nonfatalrate	.018	.017
Covariance	Log_fatalrate	.008	.003
	Log_nonfatalrate	.003	.003
Correlation	Log_fatalrate	1.000	.627
	Log_nonfatalrate	.627	1.000

Based on Type III Sum of Squares

Lack of Fit

Multivariate Tests

Dependent Variables		Value	F	Hypothesis df	Error df
Log_fatalrate, Log_nonfatalrate	Pillai's Trace	.[a]	.	.	.
	Wilks' Lambda	.[a]	.	.	.
	Hotelling's Trace	.[a]	.	.	.
	Roy's Largest Root	.[a]	.	.	.
Log_fatalrate	Pillai's Trace	.[a]	.	.	.
	Wilks' Lambda	.[a]	.	.	.
	Hotelling's Trace	.[a]	.	.	.
	Roy's Largest Root	.[a]	.	.	.
Log_nonfatalrate	Pillai's Trace	.[a]	.	.	.
	Wilks' Lambda	.[a]	.	.	.
	Hotelling's Trace	.[a]	.	.	.
	Roy's Largest Root	.[a]	.	.	.

Multivariate Tests

Dependent Variables		Sig.	Partial Eta Squared	Noncent. Parameter
Log_fatalrate, Log_nonfatalrate	Pillai's Trace	.	.	.
	Wilks' Lambda	.	.	.
	Hotelling's Trace	.	.	.
	Roy's Largest Root	.	.	.
Log_fatalrate	Pillai's Trace	.	.	.
	Wilks' Lambda	.	.	.
	Hotelling's Trace	.	.	.
	Roy's Largest Root	.	.	.
Log_nonfatalrate	Pillai's Trace	.	.	.
	Wilks' Lambda	.	.	.
	Hotelling's Trace	.	.	.
	Roy's Largest Root	.	.	.

Multivariate Tests

Dependent Variables		Observed Power[b]
Log_fatalrate, Log_nonfatalrate	Pillai's Trace	.
	Wilks' Lambda	.
	Hotelling's Trace	.
	Roy's Largest Root	.
Log_fatalrate	Pillai's Trace	.
	Wilks' Lambda	.
	Hotelling's Trace	.
	Roy's Largest Root	.
Log_nonfatalrate	Pillai's Trace	.
	Wilks' Lambda	.
	Hotelling's Trace	.
	Roy's Largest Root	.

a. The pure error sub-matrix corresponding to the selected dependent variables is a zero matrix.

b. Computed using alpha = .05

Univariate Tests

Dependent Variable	Source	Sum of Squares	df	Mean Square	F	Sig.
Log_fatalrate	Lack of Fit	.050	6	.008	.	.
	Pure Error	.000	0	.		
Log_nonfatalrate	Lack of Fit	.017	6	.003	.	.
	Pure Error	.000	0	.		

Univariate Tests

Dependent Variable	Source	Partial Eta Squared	Noncent. Parameter	Observed Power[a]
Log_fatalrate	Lack of Fit	1.000	.	.
	Pure Error			
Log_nonfatalrate	Lack of Fit	1.000	.	.
	Pure Error			

a. Computed using alpha = .05

SSCP Matrix

		Log_fatalrate	Log_nonfatalrate
Lack of Fit	Log_fatalrate	.050	.018
	Log_nonfatalrate	.018	.017
Pure Error	Log_fatalrate	.000	.000
	Log_nonfatalrate	.000	.000

Estimated Marginal Means

Grand Mean

Dependent Variable	Mean	Std. Error	95% Confidence Interval Lower Bound	95% Confidence Interval Upper Bound
Log_fatalrate	.953[a]	.032	.874	1.032
Log_nonfatalrate	3.660[a]	.019	3.613	3.706

a. Covariates appearing in the model are evaluated at the following values: Inspector_ratio = 1.157200.

Log-linear multivariate regression analysis (PP. 41-47)

Notes

Output Created		24-FEB-2026 08:45:18
Comments		
Input	Active Dataset	DataSet2
	Filter	<none>
	Weight	<none>
	Split File	<none>
	N of Rows in Working Data File	8
Missing Value Handling	Definition of Missing	User-defined missing values are treated as missing.
	Cases Used	Statistics are based on all cases with valid data for all variables in the model.
Syntax		GLM Log_fatalrate Log_nonfatalrate WITH Inspector_ratio /METHOD=SSTYPE(3) /INTERCEPT=INCLUDE /SAVE=PRED SEPRED RESID ZRESID COOK LEVER /EMMEANS=TABLES(OVERALL) WITH(Inspector_ratio=MEAN) /PRINT=DESCRIPTIVE ETASQ OPOWER PARAMETER TEST(SSCP) RSSCP HOMOGENEITY LOF /PLOT=SPREADLEVEL RESIDUALS /CRITERIA=ALPHA(.05) /DESIGN=Inspector_ratio.
Resources	Processor Time	00:00:00.28
	Elapsed Time	00:00:00.25
Variables Created or Modified	PRE_3	Predicted Value for Log_fatalrate
	PRE_4	Predicted Value for Log_nonfatalrate
	SEP_3	Standard Error of Predicted Value for Log_fatalrate
	SEP_4	Standard Error of Predicted Value for Log_nonfatalrate
	RES_3	Residual for Log_fatalrate
	RES_4	Residual for Log_nonfatalrate
	ZRE_3	Standardized Residual for Log_fatalrate
	ZRE_4	Standardized Residual for Log_nonfatalrate
	COO_3	Cook's Distance for Log_fatalrate
	COO_4	Cook's Distance for Log_nonfatalrate
	LEV_3	Uncentered Leverage Value for Log_fatalrate
	LEV_4	Uncentered Leverage Value for Log_nonfatalrate

Warnings

The HOMOGENEITY specification in the PRINT subcommand will be ignored because there are no between-subjects factors.
The SPREADLEVEL specification has been found in the PLOT subcommand, but the model includes no factors. The spread versus level plot will not be produced.

Descriptive Statistics

	Mean	Std. Deviation	N
Log_fatalrate	.9529	.08654	8
Log_nonfatalrate	3.6596	.05322	8

Bartlett's Test of Sphericity[a]

Likelihood Ratio	.045
Approx. Chi-Square	3.871
df	2
Sig.	.144

Tests the null hypothesis that the residual covariance matrix is proportional to an identity matrix.[a]

a. Design: Intercept + Inspector_ratio

Multivariate Tests[a]

Effect		Value	F	Hypothesis df	Error df	Sig.
Intercept	Pillai's Trace	.969	78.605[b]	2.000	5.000	<.001
	Wilks' Lambda	.031	78.605[b]	2.000	5.000	<.001
	Hotelling's Trace	31.442	78.605[b]	2.000	5.000	<.001
	Roy's Largest Root	31.442	78.605[b]	2.000	5.000	<.001
Inspector_ratio	Pillai's Trace	.334	1.254[b]	2.000	5.000	.362
	Wilks' Lambda	.666	1.254[b]	2.000	5.000	.362
	Hotelling's Trace	.502	1.254[b]	2.000	5.000	.362
	Roy's Largest Root	.502	1.254[b]	2.000	5.000	.362

Multivariate Tests[a]

Effect		Partial Eta Squared	Noncent. Parameter	Observed Power[c]
Intercept	Pillai's Trace	.969	157.210	1.000
	Wilks' Lambda	.969	157.210	1.000
	Hotelling's Trace	.969	157.210	1.000
	Roy's Largest Root	.969	157.210	1.000
Inspector_ratio	Pillai's Trace	.334	2.508	.170
	Wilks' Lambda	.334	2.508	.170
	Hotelling's Trace	.334	2.508	.170
	Roy's Largest Root	.334	2.508	.170

a. Design: Intercept + Inspector_ratio
b. Exact statistic
c. Computed using alpha = .05

Tests of Between-Subjects Effects

Source	Dependent Variable	Type III Sum of Squares	df	Mean Square	F	Sig.
Corrected Model	Log_fatalrate	.002[a]	1	.002	.268	.623
	Log_nonfatalrate	.003[b]	1	.003	.933	.371
Intercept	Log_fatalrate	.009	1	.009	1.128	.329
	Log_nonfatalrate	.367	1	.367	128.493	<.001
Inspector_ratio	Log_fatalrate	.002	1	.002	.268	.623
	Log_nonfatalrate	.003	1	.003	.933	.371
Error	Log_fatalrate	.050	6	.008		
	Log_nonfatalrate	.017	6	.003		
Total	Log_fatalrate	7.317	8			
	Log_nonfatalrate	107.163	8			
Corrected Total	Log_fatalrate	.052	7			
	Log_nonfatalrate	.020	7			

Tests of Between-Subjects Effects

Source	Dependent Variable	Partial Eta Squared	Noncent. Parameter	Observed Power[c]
Corrected Model	Log_fatalrate	.043	.268	.072
	Log_nonfatalrate	.135	.933	.130
Intercept	Log_fatalrate	.158	1.128	.147
	Log_nonfatalrate	.955	128.493	1.000
Inspector_ratio	Log_fatalrate	.043	.268	.072
	Log_nonfatalrate	.135	.933	.130
Error	Log_fatalrate			
	Log_nonfatalrate			
Total	Log_fatalrate			
	Log_nonfatalrate			
Corrected Total	Log_fatalrate			
	Log_nonfatalrate			

a. R Squared = .043 (Adjusted R Squared = -.117)
b. R Squared = .135 (Adjusted R Squared = -.010)
c. Computed using alpha = .05

Parameter Estimates

Dependent Variable	Parameter	B	Std. Error	t	Sig.	95% Confidence Interval Lower Bound
Log_fatalrate	Intercept	.641	.603	1.062	.329	-.836
	Inspector_ratio	.270	.521	.518	.623	-1.005
Log_nonfatalrate	Intercept	4.000	.353	11.335	<.001	3.136
	Inspector_ratio	-.294	.304	-.966	.371	-1.039

Parameter Estimates

Dependent Variable	Parameter	95% Confidence Interval Upper Bound	Partial Eta Squared	Noncent. Parameter	Observed Power[a]
Log_fatalrate	Intercept	2.118	.158	1.062	.147
	Inspector_ratio	1.544	.043	.518	.072
Log_nonfatalrate	Intercept	4.863	.955	11.335	1.000
	Inspector_ratio	.451	.135	.966	.130

a. Computed using alpha = .05

Between-Subjects SSCP Matrix

			Log_fatalrate	Log_nonfatalrate
Hypothesis	Intercept	Log_fatalrate	.009	.059
		Log_nonfatalrate	.059	.367
	Inspector_ratio	Log_fatalrate	.002	-.002
		Log_nonfatalrate	-.002	.003
Error		Log_fatalrate	.050	.018
		Log_nonfatalrate	.018	.017

Based on Type III Sum of Squares

Residual SSCP Matrix

		Log_fatalrate	Log_nonfatalrate
Sum-of-Squares and Cross-Products	Log_fatalrate	.050	.018
	Log_nonfatalrate	.018	.017
Covariance	Log_fatalrate	.008	.003
	Log_nonfatalrate	.003	.003
Correlation	Log_fatalrate	1.000	.627
	Log_nonfatalrate	.627	1.000

Based on Type III Sum of Squares

Lack of Fit

Multivariate Tests

Dependent Variables		Value	F	Hypothesis df	Error df
Log_fatalrate, Log_nonfatalrate	Pillai's Trace	.[a]	.	.	.
	Wilks' Lambda	.[a]	.	.	.
	Hotelling's Trace	.[a]	.	.	.
	Roy's Largest Root	.[a]	.	.	.
Log_fatalrate	Pillai's Trace	.[a]	.	.	.
	Wilks' Lambda	.[a]	.	.	.
	Hotelling's Trace	.[a]	.	.	.
	Roy's Largest Root	.[a]	.	.	.
Log_nonfatalrate	Pillai's Trace	.[a]	.	.	.
	Wilks' Lambda	.[a]	.	.	.
	Hotelling's Trace	.[a]	.	.	.
	Roy's Largest Root	.[a]	.	.	.

Multivariate Tests

Dependent Variables		Sig.	Partial Eta Squared	Noncent. Parameter
Log_fatalrate, Log_nonfatalrate	Pillai's Trace	.	.	.
	Wilks' Lambda	.	.	.
	Hotelling's Trace	.	.	.
	Roy's Largest Root	.	.	.
Log_fatalrate	Pillai's Trace	.	.	.
	Wilks' Lambda	.	.	.
	Hotelling's Trace	.	.	.
	Roy's Largest Root	.	.	.
Log_nonfatalrate	Pillai's Trace	.	.	.
	Wilks' Lambda	.	.	.
	Hotelling's Trace	.	.	.
	Roy's Largest Root	.	.	.

Multivariate Tests

Dependent Variables		Observed Power[b]
Log_fatalrate, Log_nonfatalrate	Pillai's Trace	.
	Wilks' Lambda	.
	Hotelling's Trace	.
	Roy's Largest Root	.
Log_fatalrate	Pillai's Trace	.
	Wilks' Lambda	.
	Hotelling's Trace	.
	Roy's Largest Root	.
Log_nonfatalrate	Pillai's Trace	.
	Wilks' Lambda	.
	Hotelling's Trace	.
	Roy's Largest Root	.

a. The pure error sub-matrix corresponding to the selected dependent variables is a zero matrix.
b. Computed using alpha = .05

Univariate Tests

Dependent Variable	Source	Sum of Squares	df	Mean Square	F	Sig.
Log_fatalrate	Lack of Fit	.050	6	.008	.	.
	Pure Error	.000	0	.		
Log_nonfatalrate	Lack of Fit	.017	6	.003	.	.
	Pure Error	.000	0	.		

Univariate Tests

Dependent Variable	Source	Partial Eta Squared	Noncent. Parameter	Observed Power[a]
Log_fatalrate	Lack of Fit	1.000	.	.
	Pure Error			
Log_nonfatalrate	Lack of Fit	1.000	.	.
	Pure Error			

a. Computed using alpha = .05

SSCP Matrix

		Log_fatalrate	Log_nonfatalrate
Lack of Fit	Log_fatalrate	.050	.018
	Log_nonfatalrate	.018	.017
Pure Error	Log_fatalrate	.000	.000
	Log_nonfatalrate	.000	.000

Estimated Marginal Means

Grand Mean

Dependent Variable	Mean	Std. Error	95% Confidence Interval Lower Bound	95% Confidence Interval Upper Bound
Log_fatalrate	.953[a]	.032	.874	1.032
Log_nonfatalrate	3.660[a]	.019	3.613	3.706

a. Covariates appearing in the model are evaluated at the following values: Inspector_ratio = 1.157200.

Observed * Predicted * Std. Residual Plots

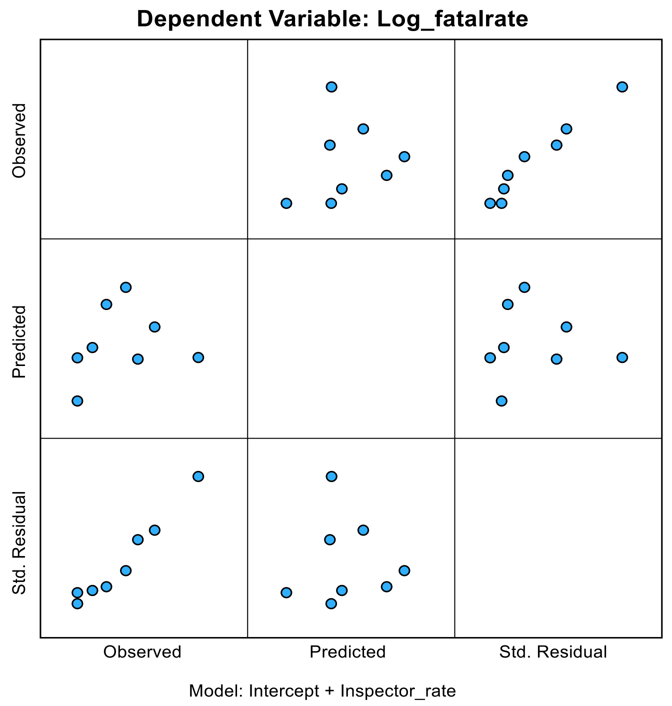

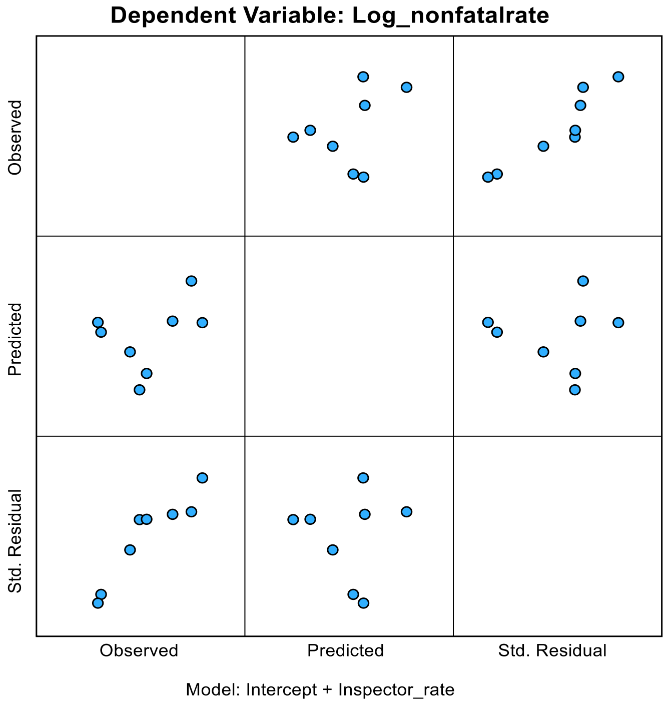

Thus, following analysis have been completed with IBM SPSS 30.0 for Saskatchewan, as hereunder-

- Descriptive statistics with original data associated with the research variables,
- General linear multivariate regression,
- General linear multivariate regression with bootstrap-2000 with Confidence Intervals of 95%,
- Descriptive statistics with log-transformation of dependent variables,
- Log-general linear multivariate regression with bootstrap-2000 with Confidence Intervals of 95% and
- Log-general linear multivariate regression analysis.
